# Comprehensive Identification and Modified-Site Mapping of *S-*Nitrosylated Targets in Prostate Epithelial Cells

**DOI:** 10.1371/journal.pone.0009075

**Published:** 2010-02-05

**Authors:** Ying Wai Lam, Yong Yuan, Jared Isaac, C. V. Suresh Babu, Jarek Meller, Shuk-Mei Ho

**Affiliations:** 1 Department of Environmental Health, University of Cincinnati College of Medicine, Cincinnati, Ohio, United States of America; 2 Center for Environmental Genetics, University of Cincinnati College of Medicine, Cincinnati, Ohio, United States of America; 3 Cincinnati Cancer Consortium, University of Cincinnati College of Medicine, Cincinnati, Ohio, United States of America; University of Minnesota, United States of America

## Abstract

**Background:**

Although overexpression of nitric oxide synthases (NOSs) has been found associated with prostate diseases, the underlying mechanisms for NOS*-*related prostatic diseases remain unclear. One proposed mechanism is related to the *S-*nitrosylation of key regulatory proteins in cell-signaling pathways due to elevated levels of NO in the prostate. Thus, our primary objective was to identify *S-*nitrosylated targets in an immortalized normal prostate epithelial cell line, NPrEC.

**Methodology/Principal Findings:**

We treated NPrEC with nitroso-cysteine and used the biotin switch technique followed by gel-based separation and mass spectrometry protein identification (using the LTQ-Orbitrap) to discover *S-*nitrosylated (SNO) proteins in the treated cells. In parallel, we adapted a peptide pull-down methodology to locate the site(s) of *S-*nitrosylation on the protein SNO targets identified by the first technique. This combined approach identified 116 SNO proteins and determined the sites of modification for 82 of them. Over 60% of these proteins belong to four functional groups: cell structure/cell motility/protein trafficking, protein folding/protein response/protein assembly, mRNA splicing/processing/transcriptional regulation, and metabolism. Western blot analysis validated a subset of targets related to disease development (proliferating cell nuclear antigen, maspin, integrin β4, α-catenin, karyopherin [importin] β1, and elongation factor 1A1). We analyzed the SNO sequences for their primary and secondary structures, solvent accessibility, and three-dimensional structural context. We found that about 80% of the SNO sites that can be mapped into resolved structures are buried, of which approximately half have charged amino acids in their three-dimensional neighborhood, and the other half residing within primarily hydrophobic pockets.

**Conclusions/Significance:**

We here identified 116 potential SNO targets and mapped their putative SNO sites in NPrEC. Elucidation of how this post-translational modification alters the function of these proteins should shed light on the role of NO in prostate pathologies. To our knowledge, this is the first report identifying SNO targets in prostate epithelial cells.

## Introduction

Evidence suggesting that an impaired nitric oxide (NO)-signaling contributes to the pathogenesis of benign prostatic hyperplasia (BPH) and prostate cancer (PCa) is accumulating. This view lends supports to the postulate that chronic prostatic inflammation is an inciting factor for BPH and PCa [1], [2] or that proliferative inflammatory atrophy is the precursor of PCa [3]. Nevertheless, studies of this relationship remain sparse and the major findings are limited to a few reports of an aberrant expression of the inducible nitric oxide synthase (iNOS or NOS*-*2) in diseased prostate tissues [4]–[7].

While endothelial NOS and neuronal NOS constantly generate a basal level of NO, iNOS produces NO upon stimulation with the inflammatory cytokines, IL-2, TNF-α and IL-1β; hypoxia; and other stimuli. Thus, the induced levels of NO are highly dependent on the redox environment and the signals received by the cell. Immunohistologic studies demonstrated that iNOS is not expressed in normal prostate [8] but that the enzyme is expressed in all specimens with BPH, low- or high-grade prostatic intraepithelial neoplasia (PIN), and PCa. iNOS immunoreactivity was found to be higher in high-grade PIN and PCa than in BPH and low-grade PIN. In both BPH and PIN, immunopositivity was localized to both basal epithelial cells and secretory cells of the glandular epithelium, along with weak staining in smooth muscle cells [8], whereas both PCa and its surrounding inflammatory cells expressed high levels of iNOS. Production of high levels of NO by iNOS causes nitrosative stress (NS), which is consistent with a role of inflammation in the induction of NS. Although NS has been proposed to promote the development of prostate disease, partly by imparting damage to DNA, proteins, and lipids [3], [9]–[12], we lack a clear understanding of the mode of action of NS, despite recent reports suggesting that the aggressiveness of PCa cell lines can partly be determined by NO [13], [14]. In particular, the impact of NS on normal prostatic epithelial cells with regard to early stages of disease development is unknown and warrants investigation.

Traditionally, NO has been demonstrated to act through the guanylate cyclase/cGMP signaling pathway to regulate many physiological processes [15], [16]. However, through reversible *S-*nitrosylation of cysteine residues on specific regulatory proteins, NO has the unique function of affecting cell survival and death, mostly reported for endothelial cells and neurons [17], [18]. NO generated by NOS reacts with intracellular glutathione to form nitrosoglutathione, an intracellular reservoir, which in turn transnitrosylate protein thiol to form nitrocysteine, thus modifying protein functions [19]. A number of signal transduction molecules, including those participating in apoptosis (e.g., Bcl-2, caspase-3, GAPDH, TRAIL receptor DR4, NFκB, RAC/p21, and Ras), have been identified as targets of *S-*nitrosylation and their activity and/or stability is affected by overproduction of NO [17], [20]. Additionally, NO has been shown to inhibit 8-oxodeoxyguanosine DNA glycosylase, a DNA repair enzyme, via *S-*nitrosylation, which may allow DNA damage to accumulate during cell proliferation, linking chronic inflammation to carcinogenesis [21], [22]. Therefore, high output of NO due to iNOS overexpression in the normal prostatic epithelial cells and/or their neighboring inflammatory cells will likely nitrosylate key prostatic proteins responsible for the pathogenesis of prostate diseases, including BPH and PCa.

The biotin switch technique (BST) developed by Jaffrey et al. [23] is now an established method for analyzing protein *S-*nitrosylation. BST allows *S-*nitrosylated proteins (protein-SNOs) to be detected in a complex mixture such as a cell lysate. However, most profiling studies using BST identified the protein-SNOs but did not report the site of modification. A few recent studies have incorporated an additional procedure involving a peptide pull-down step followed by LC-MS/MS sequencing to identify the site of modification on the target proteins in various tissues and cell types [24]–[27]. Yet, protein-SNO has not been identified in prostate epithelial cells.

Our goal is to gain new insights into the role of NS in the development of prostate diseases by comprehensively identifying these targets and their modification sites in an immortalized normal prostate epithelial cell line (NPrEC) using a protein and peptide pull-down combination protocol. We here identified 116 protein-SNO in the NPrEC and determined the sites of modification for 82 of them. Over 60% of the proteins belong to the functional categories of cell structure/cell motility/protein trafficking, protein folding/protein response/protein assembly, mRNA splicing/processing/transcriptional regulation, and metabolisms. We also have used bioinformatic approaches to analyze the SNO sequences in terms of primary and secondary structures, solvent accessibility, and three-dimensional (3-D) structural analysis for those whose protein structures are available. Western-blot analysis validated the *S-*nitrosylation status of a subset of targets related to cancer development. Bioinformatics revealed that 79% of the SNO sites that can be mapped into resolved structures are fully buried, among which half have charged amino acids in the 3-D neighborhood. Future functional elucidation of these targets will yield insights into the role of NO, NS, and/or inflammation in the development of prostate diseases.

## Materials and Methods

### Cell Culture

An immortalized normal prostate cell line (NPrEC) was maintained in Defined Keratinocyte-SFM medium with growth-promoting supplement (Invitrogen, Carlsbad, CA). Prostate cancer cell line (PC3) was maintained in F12 medium with Kaighn's modification (F-12K), 4 mM L + glutamine (ATCC, Manassas, VA) and 10% fetal bovine serum. Cell cultures were maintained at 37°C in a humidified incubator with 5% CO_2_. *S-*nitroso-L-cysteine (CysNO) was freshly synthesized by mixing equimolar concentrations of NaNO_2_ in water and cysteine in 1 N HCl followed by incubation at room temperature for 30 min and neutralization with an equal volume of HEN buffer (250 mM HEPE*S-*NaOH, 1 mM EDTA, and 0.1 mM neocuproine, pH 7.8) [28]. NPrEC were seeded in 150-mm dishes and treated with 1 mM CysNO from a 250 mM CysNO stock solution for 15 min in the dark. The dose of CysNO (1 mM) selected was based on previous studies of endothelial cells [28]. After 15 min, the cells were harvested by scraping. The cell pellets were washed twice with 1x phosphate buffer saline (PBS) and stored frozen until protein extraction.

### Cell Transfection

PC3 cells (1×10^6^) were transfected in 10-cm dishes using Mirus *Trans*IT®-Prostate Transfection Kit (MirusBio Corp. Madison, WI) with 24 µg of iNOS expression plasmid (BC130283; TOPO pcDNA 3.1) for 24 hr prior to harvest. Cells were harvested with scraping using 1x PBS/1 mM EDTA/0.1 mM neocuproine and the pellet stored at −80°C.

### Biotin Switch Technique (BST)

The biotin switch technique was performed as previously described [23], [29], [30]. The cell pellet was lysed with HEN buffer/1% Triton X-100, with the addition of protease inhibitor (Roche, Basel, Switzerland) except for those samples used for biotinylated peptide capture. The protein concentration was adjusted to 0.7–1 mg/mL. As the first step of BST, free cysteine thiols were blocked by *S-*methylthiolation by adding methyl-methane thiosulfonate (MMTS) [2 M in dimethylformide (DMF)] to a final concentration of 100 mM in the presence of 2.5% SDS at 50°C for 30 min. Two volumes of acetone were added, followed by incubation at −20°C for 20 min to precipitate the protein. The pellet was then washed four times with 70% acetone and then resuspended in 1% SDS in HEN/10 buffer. The SNOs were then converted to thiols via transnitrosation with ascorbate before the nascent thiols were biotinylated with biotin-HPDP, a reactive mixed disulfide of biotin. Specifically, biotinylation was performed by adding 1/10 volume of 4 mM biotin-HPDP (Pierce) in DMSO (prepared from a 50 mM stock in DMF) in the presence or absence of 100 mM freshly prepared sodium ascorbate (NaAsc; Fluka) in HEN/10 buffer [29], and incubated at room temperature for 1 h. The assay was performed in amber tubes with minimal light exposure. Gel electrophoresis was performed after biotinylation with non-reducing sample buffer to detect biotinylated proteins.

### Purification of Biotinylated Proteins

After biotinylation, proteins were precipitated with acetone; protein pellets were washed with 70% acetone four times and resuspended in 250 µL of 25 mM HEPES/1 mM EDTA/1% SDS; 750 µL of neutralization buffer (25 mM HEPES/1 mM EDTA/1% Triton X/100 mM NaCl) was added to the samples, followed by centrifugation to remove insoluble aggregates. Prewashed high-capacity NeutrAvidin agarose resin (Pierce, Rockford, IL) was used to pull down biotinylated proteins at room temperature for 2–3 h (Pierce; 50 µL dried beads per 1 mg of initial starting material). After pull-down, the beads were washed four times with five volumes of neutralization buffer with 500 mM NaCl, followed by two washes with five volumes of neutralization buffer. The protein was eluted with two dry-bead volumes of 25 mM HEPES/1 mM EDTA/100 mM β-mercaptoethanol at room temperature for 30 min with shaking. The protein was then concentrated by Microcon (10,000 MWCO, Millipore, MA), heated at 95°C with reducing sample buffer, loaded onto SD*S-*PAGE (Ready Gel, Biorad), followed by western-blot analysis or stained with silver (Invitrogen, Carlsbad, CA) or Coomassie Blue (Biorad, Hercules, CA).

### Capture of Biotinylated Peptides

*S-*nitrosylated peptides were captured as previously described [25]. In brief, 1 mg of protein was used for BST and after MMTS blocking, biotinylation, and acetone precipitation, protein pellets were resuspended in 500 µL of 100 mM NH_4_HCO_3_/10% ACN and incubated with 50 µg (per 1 mg of protein) of trypsin at 37°C for 18 h. The resulting digest was passed through a Microcon (10,000 MWCO, Millipore, MA), followed by pull-down with NeutrAvidin agarose resin (50-µL beads) at room temperature for 30 min. The precipitate was washed five times with 10 volumes of 1 M NH_4_HCO_3_ and five times with 10 volumes of water. Elution was performed by incubating the beads with 75 µL of 70% formic acid for 30 min. The eluate was evaporated to 2 µL and resuspended to 10 µL with 2% ACN/0.1% FA. Half of the sample was subjected to LC/MS.

### Gel Electrophoresis and Western Blot Analysis

SD*S-*PAGE was performed with ReadyGel 4–15% (Biorad, Hercules, CA) or Precise protein gel 8 or 12% (Pierce, Rockford, IL). After electrophoresis, gels were transferred onto a PVDF membrane (Immobilon FL, Millipore, MA) with a blotting cell (Invitrogen, Carlsbad, CA) at 30 V for 2 h. Primary antibodies were used with the following conditions: mouse monoclonal proliferating cell nuclear antigen (PCNA) (Santa Cruz sc-56; 1∶1000, 1 h at RT); mouse monoclonal maspin (BD Pharmingen; 1∶500, O/N at 4°C); integrin β4 (Santa Cruz; 1∶1000, O/N at 4°C); α-catenin (BD Pharmingen; 1∶500, O/N at 4°C); karyopherin β1 (Santa Cruz (H-300), 1∶1000, O/N at 4°C]; mouse monoclonal elongation factor 1 alpha (EF1α) (Upstate, CA; 1∶1000, O/N at 4°C), which recognizes both EF1A1 and EF1A2; streptavidin Alexa Fluor 680 (Invitrogen; 1∶1000, 1 h at RT). IRDye antibiotin was used at 1∶1000 overnight, and corresponding IRDye conjugated secondary antibodies (Rockland, PA) were used at 1∶5000 dilutions. Starting material of 1 mg was used for western-blot confirmation.

### Tryptic Digestion and LC-MS/MS

Tryptic digestion was performed as described previously [31], [32]. In brief, silver-stained gel bands were excised and destained with a 50 µL of a 1∶1 mixture of 30 mM potassium ferricyanide (Sigma, MO) and 100 mM sodium thiosulfate (Sigma) in a siliconized tube until the brownish stain disappeared (∼5 min), whereas CBB-stained gel bands were destained with 50% ACN/MeOH/5% acetic acid for 4 h, followed by 50% ACN/50 mM NH_4_HCO_3_ for 2–3 h. After the gel pieces were washed with 40 mM NH_4_HCO_3_, they were minced, dehydrated with ACN, dried in a SpeedVac, and subjected to digestion with Trypsin Gold (Promega, Madison, WI) for 18 h at 37°C. Peptides were extracted successively with 1% FA/50% ACN, 80% ACN/1% FA, and 100% ACN and then purified by ZipTip (Millipore, MA). Each digest was analyzed by capillary LC-MS/MS with a Finnigan LTQ-Orbitrap (Thermo Fisher Scientific, MA). Half of the digest was loaded directly onto the 75 µm×100 mm PicoFrit capillary column (New Objective, MA) packed with MAGIC C18 (100 Å 5 µ, Michrom Bioresources, CA) at a flow rate of ∼300 nL/min, and peptides were separated by a gradient comprising 2–60% ACN/0.1% FA in 30 min, 60–98% ACN/0.1% FA in 4 min, and held at 98% ACN/0.1%FA for 2 min. The LTQ-Orbitrap was operated in standard data-dependent “top-three” mode with lock mass function activated (protonated polydimethylcyclosiloxane [Si(CH3)2O))_6_; *m*/*z* 445.120025)]. A survey scan from *m/z* 300–1600 at 60,000 resolution in the Orbitrap was paralleled by 3 MS/MS scans in the LTQ. Exclusion duration was set for 3 min. The minimum signal threshold was 250. Singly charged ions were excluded for MS/MS.

### Data Analysis

The product ion spectra were searched against the latest version of the human subset of the International Protein Index (IPI) database containing sequences in forward and reverse orientations (v3.56, Mar 09, target-decoy) using the SEQUEST search engine in Bioworks 3.3. The database was indexed with fully enzymatic activity and two missed cleavage sites allowed for trypsin; peptides MW of 600–6000. SEQUEST search parameters were as follows: mass tolerance of 15 ppm and 1 amu for precursor and fragment ions, respectively; three differential/post-translational modifications allowed per peptide; variable modification on methionine [+15.9949 amu for oxidized methionine and +45.98772 amu (MMTS) on cysteine]. For biotinylated peptides, variable modifications of +428.191567 amu (HPDP-biotin) were included to allow identification of the site of modification. Analysis was performed in Bioworks 3.3 by applying filters of XCorr [2.0 (2+), 2.5(3+)]; DelCN (≥0.1)], SP>/ = 300, and precursor mass accuracy ≤15 ppm. Protein identifications were ranked by protein probability P (pro), and the false positive rate was limited to <1% FP. The MS/MS spectra of biotinylated peptides were evaluated by Scaffold (Proteome Software, OR).

### Protein Classification

Identified *S-*nitrosylated proteins were then classified by the PANTHER system (www.pantherdb.com) on the basis of their unique gene IDs. The classification system provides information on the candidates regarding their molecular function and the biological processes and signal transduction pathways to which they belong.

### Mapping *S-*Nitrosylated Sites into High-Resolution PDB Structures

The initial set comprising 82 proteins with 141 *S-*nitrosylated cysteines was analyzed to identify sites that can be mapped into structurally resolved proteins and consequently assessed by their structural context. This process was facilitated by automated mapping into the PFAM database of protein domains and the Protein Data Bank (PDB) of structurally resolved proteins, using SCORPPION (http://scorppion.cchmc.org) and POLYVIEW-3D (http://polyview.cchmc.org/polyview3d.html) servers [33]. Structurally resolved fragments or domains of 35 proteins, or their sufficiently close homologs (we used a BLASTp E-value of <10E-50 and sequence identity of >90%) with conserved SNO sites, have been identified in PDB. Since PDB structures often cover only part of the target sequence, 43 SNO sites in 24 proteins were subsequently mapped into resolved fragments. Further validation of sequence-to-structure mapping was performed with PROSITE (http://ca.expasy.org/prosite/) searches. Residues and atoms in contact with SNO cysteines were identified with the iMolTalk server (http://i.moltalk.org/) and the Loopp program [34], with a cutoff distance of 5 Å from the sulfur atom of the cysteine. These protein structures, together with the location of *S-*nitrosylated cysteines, are listed in Supporting Information Table S2.

### Sequence-Based Bioinformatic Analysis of Cysteine-Containing Peptides

To further assess sequence motifs and propensity for secondary structure and solvent exposure states for all (and not only structurally resolved) SNO proteins, we used sequence-based analysis and prediction methods. In particular, we used the SABLE server (http://sable.cchmc.org) [35] to predict from sequence secondary structures and solvent accessibilities, with the goal of identifying potential characteristics of SNO sites in terms of structural profiles. We analyzed sequence motifs around *S-*nitrosylated cysteines with WebLogo (http://weblogo.berkeley.edu/). In addition, we computed frequencies of amino acid residues with reduced alphabets (e.g., hydrophobic vs. hydrophilic) within windows of up to 21 residues centered at the *S-*nitrosylated cysteines. The search for putative sequence motifs was performed with the MEME (http://meme.nbcr.net) and PRATT (http://www.ebi.ac.uk/Tools/pratt/index.html) servers.

## Results

### 1. Identification of Protein Targets of *S*-Nitrosylation

**Figure 1:**
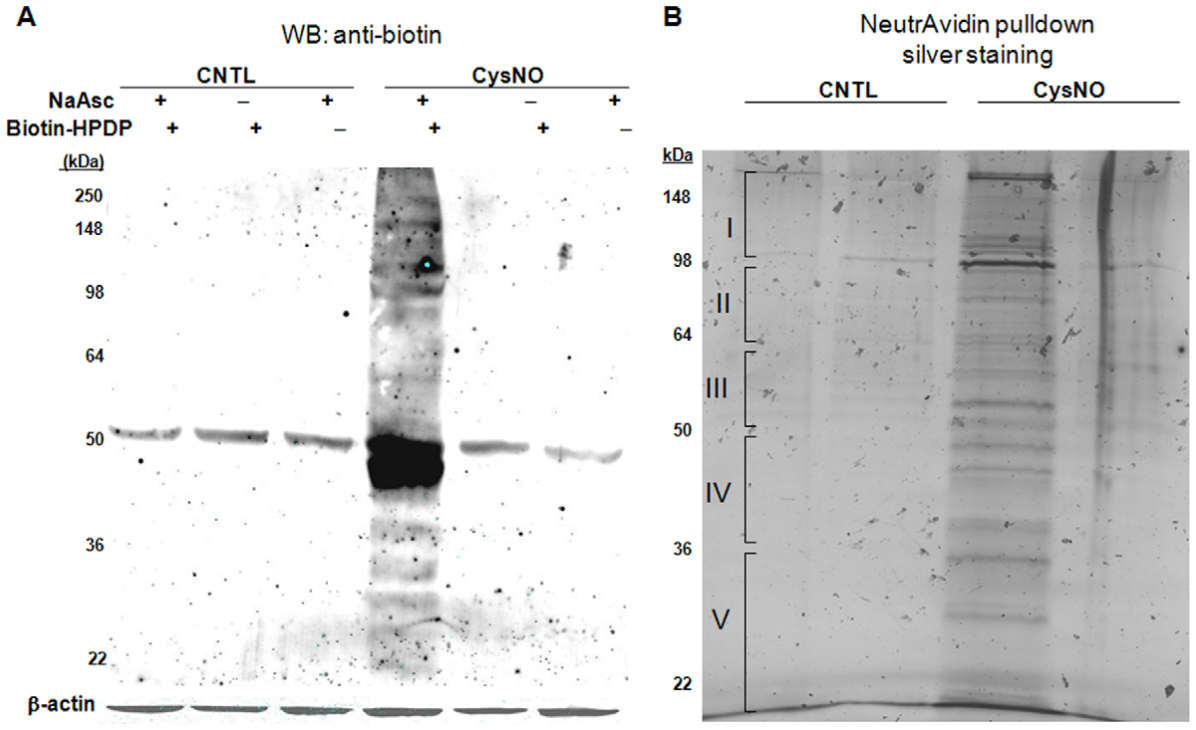
*S-*nitrosylation in NPrEC. **A**) Normal prostate cells, NPrEC, were treated with or without 1 mM CysNO followed by a biotin BST. Protein extract (100 µg) was loaded onto an SDS*-*PAGE (10%). Western-blot analysis was carried out, and the membrane was probed with anti-biotin. **B**) After cell treatment (1 mM CysNO) and the biotin switch assay, neutravidin pull-down and SD*S-*PAGE (4–15%) was performed (3 mg was used for IP in each lane). Each lane (CNTL, CNTLΔNaAsc, CysNO, CysNOΔNaAsc) was divided into five portions (I–V) as indicated and subjected to tryptic digestion and mass spectrometry protein identification.

The BST was performed, and its specificity in NPrEC was demonstrated as described below. Western-blot analysis (Figure 1) of protein *S-*nitrosylation in NPrEC noted a strong anti-biotin immunoreactivity for biotinylated proteins only in cells treated with CysNO but not in untreated control cells (CNTL), indicating a complete blocking of free cysteine thiols by MMTS and minimal endogenous *S-*nitrosylation in untreated cells. The absence of a signal in reactions without NaAsc demonstrates that the modification is SNO-specific [29]. After pull-down of the protein-SNOs and SD*S-*PAGE, distinct bands were clearly visible on the silver-stained gels in the CysNO lane but not in the CysNO minus NaAsc and control lanes (Figure 1B). These data are in accordance with results of the western-blot analysis (Figure 1A), indicating specific pull-down of biotinylated proteins.

**Figure 2:**
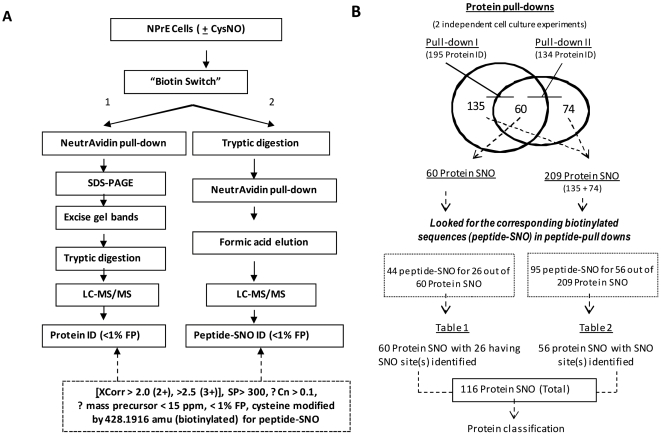
Identification of *S-*nitrosylated proteins. **A) Schematic of the workflow.** *Approach 1*. Protein extracted from NPrEC treated with CysNO (1 mM) was subjected to a BST, and nitrosylated proteins were purified by Neutravidin pull-down, followed by SD*S-*PAGE. Nitrosylated proteins were identified by tryptic digestion and LC-MS/MS in the five gel bands (I–V) of lanes (CNTL, CNTLΔNaAsc, CysNO, CysNOΔNaAsc) according to the defined peptide selection criteria (XCorr 1.5, 2.0, 2.5, ΔCn>0.1, Sp>300, precursor <15 ppm, with <1% FP). After the protein-SNOs were identified, their sites of modification in peptide (peptide SNO) were identified in approach 2: after biotin switching, 1 mg of protein was digested and the nitrosylated peptides were pulled down and subjected to mass spectrometric site mapping, according to the same peptide selection criteria. **B**) Number of protein-SNO and peptide-SNO identification: Protein pull-downs from two independent experiments were performed to identify protein-SNOs, followed by the identification of the corresponding peptide-SNOs from peptide pull-downs. A total of 116 proteins were identified, with the sites of modification determined for 82.

After establishing the efficacy and specificity of the BST in NPrEC, we used the scheme depicted in Figure 2A in an effort to comprehensively identify *S-*nitrosylation targets at the protein (Approach 1) and peptide (Approach 2) levels. The identification (ID) was deemed to be a target of *S-*nitrosylation 1) if it was identified in the CysNO-treated samples but not in the untreated control samples in two dependent protein pull-down experiments, or 2) if it was identified in one of the two protein pull-down experiments and the corresponding biotinylated peptide(s) were identified in the peptide pull-down. With the mass tolerance on peptide precursor <15 ppm and the false positive (FP) rate <1% in database searching, as well as the conventional peptide filters (XCorr 1.5, 2.0, 2.5, ΔCn>0.1, Sp>300), we identified 195 and 134 unique protein-SNOs in the two protein pull-downs from two independent cell culture experiments (Pull-Down I and Pull-Down II), respectively, with 60 of them shared by the two data sets (Figure 2B, Supporting Information Table S1A). After identifying the nitrosylated proteins by Approach 1, we looked for the corresponding peptide-SNOs from multiple peptide pull-downs (Approach 2). Of the 60 IDs common to Pull-down I and Pull-down II, 24 had the corresponding biotinylated sequences (one or multiple biotinylated peptides) determined in the peptide pull-down experiments (Figure 2B, Supporting Information Table S1B). Moreover, we were able to obtain from the peptide pull-downs biotinylated sequences for 56 protein-SNOs that were identified in either one of the protein pull-downs (Figure 2B, Supporting Information Table S1B). All together, we identified 116 distinct protein SNOs [60 IDs (that are present in both protein pull-downs) and 56 IDs (that are present in one protein pull-down with a corresponding biotinylated peptide(s) found in peptide pull-downs)]. All the biotinylated peptides identified have a precursor mass accuracy <5 ppm (Supporting Information Tables S1A and S1B). The MS/MS spectra for all the biotinylated peptides identified are provided in Supporting Information Figure S1. Literature references are included in Table S1A and S1B if the protein and/or the cysteine sites have been reported to be *S-*nitrosylated.

### 2. Classification of the Nitrosylated Proteins Identified

**Figure 3:**
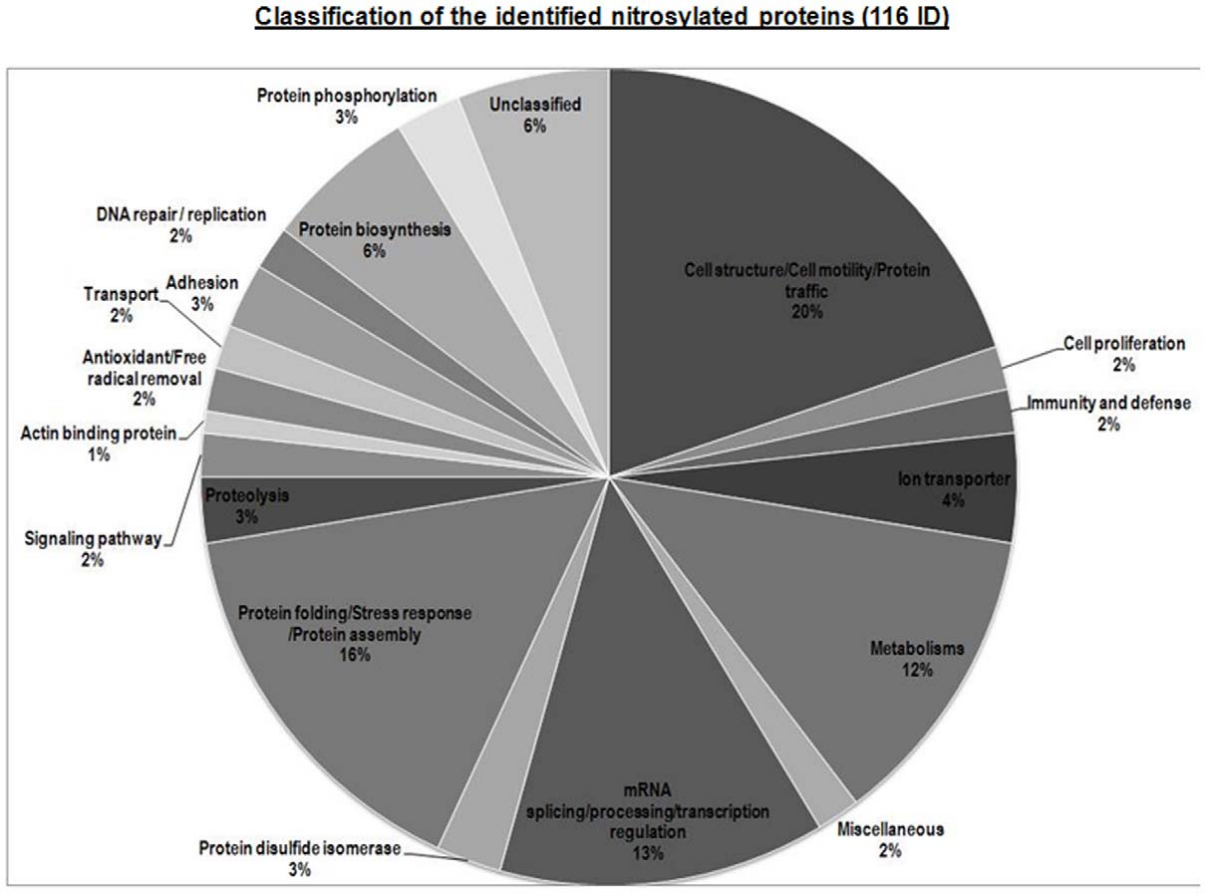
Protein-SNO classification. Proteins that were identified as nitrosylated are grouped according to the biological processes they belong to according to the Panther classification system and listed in Table S1A and S1B with their biotinylated peptides, if available.

The identified proteins were classified into different categories on the basis of their function according to the Panther classification system (Figure 3). Over 60% of the *S-*nitrosylated proteins belong to one of the four major functional categories: cell structure/cell motility/intracellular protein trafficking (20%), protein folding/stress response/protein assembly (16%), RNA splicing/processing/transcription regulation (13%), and metabolisms (12%).

*Cell structure/cell motility/intracellular protein trafficking* represents the largest category with proteins having distinct molecular functions, e.g., cell-structure components [plectin 1 (PLEC1), vimentin (VIM), actinin 1, 4 (ACTN1, ACTN4)], intracellular protein trafficking and motility [tubulin beta 2c (TUBB2C), annexin A1, 2 (ANXA1, ANXA2), reticulon 4 (RTN4), PDZ and LIM domain, elfin (PDLIM1), tropomyosin 1 (TPM1)], nucleotransport [karyopherin (importin)β1 (KPNB1)], and exocytosis/endocytosis/transport [valosin-containing protein (VCP), actin beta (ACTB), and actin alpha 2 (ACTA2)].

Under *protein folding/stress response/protein complex assembly* are chaperonin-containing TCP subunits (CCT3, CCT4, CCT5, CCT7), t-complex 1 (TCP1), heat-shock 60-kDa protein 1 (HSPD1), heat-shock 70-kDa protein 4 (HSPA4), heat-shock protein 70-kDa protein (HSPA5, HSPA8, and HSPA9), heat-shock protein 90 kDa alpha (HSP90AA1), heat-shock protein 90-kDa alpha class B member 1(HSPAB1), heat-shock protein 90-kDa beta member 1 (HSP90B1), and calnexin (CANX), among which HSPA5, A8, and A9 are involved in an apoptotic pathway and HSPB1, in the p38 MAPK pathway.

Grouped under *RNA splicing/processing/transcription regulation* are those proteins related to mRNA splicing [heterogeneous nuclear ribonucleoprotein (HNRPDL, HNRNPR, HNRNPA1), RNA-binding motif protein 39 (RBM39), DEAH box polypeptide 9 (DHX9), U2 small nuclear RNA auxiliary factor 2 (U2AF2), KH-type splicing regulatory protein (KHSRP)], mRNA transcription and processing [general transcription factor II, I (GTF2I), RuvB-like 1 (RUVBL1), TAR DNA-binding protein (TARDBP), heterogeneous nuclear ribonucleoprotein (HNRNPK, HNRNPM)], and chromatin remodeling [SWI/SNF regulator of chromatin, subfamily c, member 2 (SMARCC2)].

Under the category of *metabolisms*, we found proteins belonging to the glycolysis pathway [phosphoglycerate kinase 1 (PGK1), enolase 1 (ENO1), aldolase A (ALDOA), pyruvate kinase (PKM2), glucose phosphate isomerase (GPI), triosephosphate isomerase, (TPI1)], tricarboxylic acid cycle [citrate synthase (CS), aconitase 2 (ACO2), malate dehydrogenase 2 (MDH2)], amino acid metabolism [(aldehyde dehydrogenase 18 family, member A1 (ALDH18A1)], purine metabolism [IMP dehydrogenase (IMPDH2), adenylate kinase 2(AK2)], porphyrin metabolism [HMOX2 (heme oxygenase)], and fatty acid/lipid/steroid metabolism [farnesyl-diphosphate farnesyltransferase 1 (FDFT1), annexin A1 (ANXA1)].

Worth noting is a group of signal transduction proteins. Among them, although only 14-3-3 theta polypeptide (YWHAQ) is classified exclusively in signal transduction; a number of proteins are classified in other categories but often are associated with various signaling transduction pathways, including integrin signaling [integrin (ITGB4, ITGA6) filamin (FLNA, FLNB, FLNC), actinin alpha 1, 4 (ACTN1, ACTN4)], cadherin signaling [α-catenin (CTNNA1)], EGF signaling (epidermal growth factor receptor (EGFR), FAS signaling [lamin (LMNA, LMNB1)], as well as the and the tumor suppressor maspin (SERPINB5). Some are involved in DNA replication [proliferating cell nuclear antigen (PCNA), X-ray repair complementing defective repair (XRCC5)], cell-cycle regulation and cell proliferation [N-myc downstream-regulated 1 (NDRG1)], protein phosphorylation [protein phosphatase 1G (2C) (PPM1G)], as well as cell adhesion and induction of apoptosis [galectin 1 (LGALS1), glyceraldehyde-3-phosphate dehydrogenase GAPDH].

Finally, other classified proteins include those that share common functions in antioxidation and free-radical removal include isomerases [peroxiredoxin (PRDX1, PRDX5), protein disulfide-isomerases (PDIA3, PDIA6), proline 4-hydroxylase, beta peptide (P4HB)], those related to protein biosynthesis and translation regulation [eukaryotic translation initiation factor 2 subunit 3 (EIF2S3), eukaryotic translational elongation factor 2 (EEF2), ribosomal protein L5 (RPL5), Tu translation elongation factor, mitochondrial (TUFM), ribosomal protein S3 (RPS3), ribophorin I (RPN1), eukaryotic translation elongation factor 1 alpha 1 (eEF1A1)], and those belong to proteolysis [(proteasome activator subunit 1 (PSME1), calpastatin (CAST)], serpin peptidase inhibitor clade H (SERPINH1)] and ion transporter category [voltage-dependent anion channel 1, 2, and 3 (VDAC1,VDAC2, VDAC3), chloride intracellular channel 1 (CLIC1), and ATPase Ca^++^ transporting (ATP2A2)].

### 3. Confirmation of Nitrosylated Targets by Western Blot Analysis

**Figure 4:**
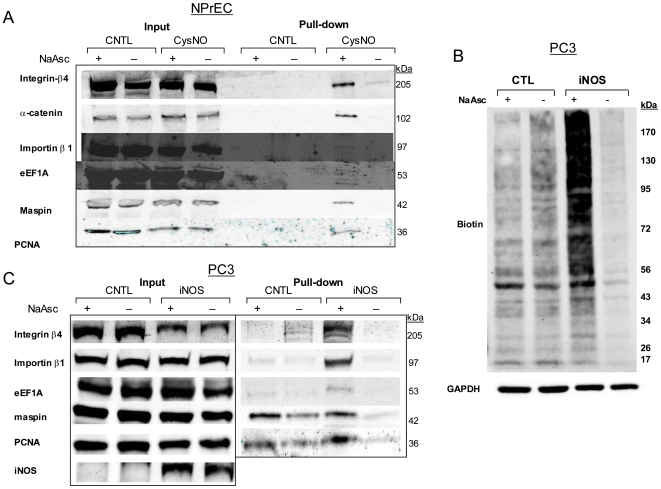
Verification of nitrosylated proteins. **A**) **Western blot analysis of targets nitrosylated by CysNO.** NPrEC were treated with 1 mM CysNO, and 1 mg of protein extract was subjected to BST. Biotinylated proteins were pulled down with Neutravidin beads, eluted with β-mercaptoethanol, concentrated and detected using western blot. 8% of total protein was loaded as input, and the same membrane was probed with respective antibodies with stripping and re-probing. **B**) **Protein nitrosylation in iNOS expressing PC3 cells.** PC3 cells were transiently transfected with an iNOS expressing plasmid. Cells were harvested 24 h post-transfection. 3.5 mg of total protein was subjected to BST, as described in Materials and Methods. After BST, the overall protein nitrosylation was assessed by western blot using an anti-biotin antibody. The membrane was stripped and re-probed with GAPDH to show equal loading. **C**). **Endogenous nitrosylation of protein targets.** Biotinylated proteins were pulled down with Neutravidin and analyzed by Western blot. Seventy µg was loaded as input, and the same membrane was probed with respective antibodies with stripping and re-probing.

We selected a subset of the identified targets that are related to cancer development (promotion or progression) and for which antibodies are commercially available for western-blot analysis (Figure 4). We confirmed the *S-*nitrosylation status of proliferating cell nuclear antigen (PCNA), maspin (serpin B4), integrin β4, α-catenin, karyopherin (importin) β1, and elongation factor 1A (eEF1A). The targets were pulled down and recognized by their respective antibodies in cell lysates prepared from NPrEC treated with CysNO but not in those prepared in the absence of NaAsc or in lysates of untreated controls.

### 4. Confirmed Targets Are Endogenously Nitrosylated

We further investigated if the protein-SNOs identified could be nitrosylated by iNOS overexpression. We transiently transfected an iNOS expression plasmid into PC3 cells and assessed the nitrosylation status of the protein targets previously identified with CysNO treatment. PC3 cells were used due to its higher transfection efficiency than NPrEC. The overall increased protein nitrosylation in PC3 cells expressing iNOS is shown in Figure 4B. The anti-biotin reactivity is specific to iNOS expressing PC3 cells (as compared to untransfected PC3 cells), and is SNO-specific (as the anti-biotin signal was diminished in the reaction without NaAsc). All the proteins selected for confirmation, except for alpha catenin (which does not express in PC3 cells), were shown to be nitrosylated after iNOS overexpression. The extent of nitrosylation was reduced in reactions without NaAsc (Figure 4C) demonstrating its SNO specificity.

### 5. Analysis of SNO Sequences by Motif Detection Methods

We performed bioinformatic analysis of the 141 SNO sites (from the 82 protein-SNOs) by several sequence motif detection methods, including secondary structure and solvent accessibility predictions and hydrophobicity analysis (as described in Materials and Methods). These analyses did not reveal any clear signatures of SNO sites as compared with a set of cysteine residues derived from a representative set of ∼1,300 proteins (data not shown).

### 6. Mapping SNO Sites into High-Resolution PDB Structures and Analysis of Structural Features

Since some of the 141 SNO sites identified in this (and previous global site-mapping studies) could represent *S-*nitrosylation events without clear functional relevance, we performed a comprehensive mapping of SNO sites into structurally resolved protein domains with the use of PFAM and PDB databases to obtain a representative data set for further bioinformatics analyses. As proteins (and their domains) that are a focus of intensive experimental (and in particular structural) studies are more likely to occur in PDB, SNO sites within these domains/proteins are also more likely to represent functionally relevant modifications. We mapped 42 SNO sites in 24 different proteins into high-resolution structures, with some of them having been reported to be nitrosylated and/or the sites having been located (Supporting Information Table S2).

**Figure 5:**
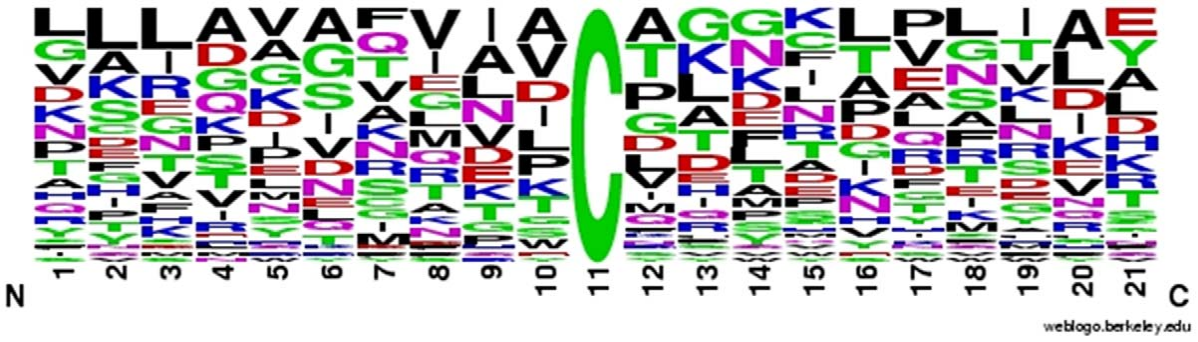
Frequency of amino acids surrounding *S-*nitrosylated cysteines in 42 sites mapped into structurally resolved domains from 24 different proteins. Hydrophobic residues are shown in black; charged residues in red and blue, respectively; and hydrophilic residues in magenta and green (for mixed-character residues).

For the 42 SNO sites that can be mapped into PDB structures, we found no clear sequence motif around the sites (shown at the center of the window of 21 residues) (Figure 5). Similarly, we observed no clear biases in terms of secondary structures. Specifically, we observed all three states (helix–H, beta-strand–E, other–C), with frequencies of ∼0.24, 0.32, and 0.45, for H, E, and C, respectively, values similar to the frequencies of 0.24, 0.36 and 0.40 found in a large set of ∼1,300 representative structures derived from PDB (Supporting Information Table S3). Regarding solvent accessibility, ∼79% (34 sites) of SNO cysteines that can be mapped into resolved structures are fully buried (even though SH groups are just under the surface in some cases), in contrast with ∼56% for a set of cysteines derived from ∼1,300 representative structures (Supporting Information Table S3). About half of these buried sites (see Supporting Information Table S4 for detailed annotation of each case) are characterized by a purely hydrophobic environment without charged residues in the direct vicinity.

### 7. Localization of Neighboring Charged Residues in the Vicinity of the SNO

**Figure 6:**
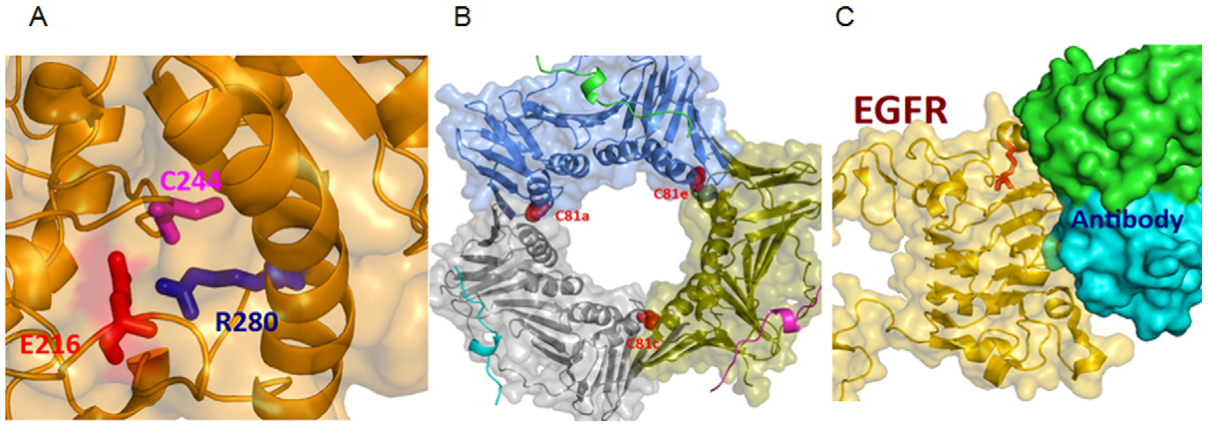
Crystal structure of the representative protein-SNOs. **A**) An example of a buried *S-*nitrosylation site in a crystal structure of the human protein disulfide-isomerase, PDIA3 (PDB code 3F8U), with positively charged (R280) and negatively charged (E216) residues in direct spatial proximity of the nitrosylated cysteine (C244). **B**) An example of an *S-*nitrosylation site in direct contact with a ligand and thus likely disrupting/affecting ligand binding upon nitrosylation. One of such examples is EGFR, which is shown here in complex with the monoclonal antibody inhibitor cetuximab (PDB structure 1YY9). Binding of the antibody partially occludes the ligand-binding site in EGFR and keeps it in an inactive conformation. As can be seen from the figure, the targeted cysteine (highlighted using red stick model) directly supports the protein interaction interface in EGFR, suggesting that *S-*nitrolysation may attenuate its interactions with ligands and inhibitors. **C**) An example of *S-*nitrosylation site(s) located within protein-protein (oligomerization) interfaces and that thus may disrupt/attenuate complex formation in PCNA and its function in DNA replication. Note that DNA, which occupies the central hole in the PCNA trimer, is not included in this complex (PDB structure 1VYJ). On the other hand, p21 peptides, which mediate regulatory interactions with CDK/cyclin complexes, are shown using cartoon models.

On the basis of previous reports that nitrosylation may be facilitated by the presence of charged residues in the vicinity of an SH group [36], [37], we then determined the neighboring amino acids within 5 Å of the cysteine nitrosothiols. We found that 17 sites, of the 34 that are fully buried, are characterized by the presence of positively and/or negatively charged residues within the 3-D neighborhood of SNO. An example of such a case (Figure 6A) is that in which a buried cysteine (C244) in protein disulfide-isomerase, PDIA3 (PDB code 3F8U) is found in close proximity with positively charged (R280) and negatively charged (E216) residues. This site in PDIA3 is buried in the core of the protein, implying that diffusion of NO through a thermally fluctuating protein matrix would be required to *S-*nitrosylate the buried cysteine residue. A likely diffusion path would involve the E216 residue (shown in red in Figure 6A), which is partially exposed and separates C244 from the solvent.

### 8. Structural Analysis Predicts That Nitrosylation Might Affect the Function of Some Proteins

Although the functions of several mapped structures (e.g., GAPDH [38], peroxiredoxin [39]) have been shown to be affected by nitrosylation (Supporting Information Table S2), structural analysis on the mapped structures revealed that nitrosylation of some of the newly found SNO sites might regulate the function of a number of proteins. One of the representative examples is EGFR, in which one of the targeted cysteines is located at the ligand-binding interface in the extracellular domain. This domain has been resolved in a complex with a monoclonal antibody inhibitor (see Figure 6B) that partially occludes ligand-binding sites and keeps the receptor in inactive conformation. *S-*nitrosylation of this cysteine thiol in EGFR might therefore directly affect its interaction with ligands and signal transduction in the EGFR pathway.

On the other hand, we found a number of SNO sites (including three in direct contact with ligands, and five at protein-protein interaction interfaces) that are accessible on the protein surface in unbound forms. One example (Figure 6C) is that in which the structure of PCNA complexed with p21 peptides that mediate regulatory interaction with cdk/cyclin complexes; the *S-*nitrosylated cysteine residues can be seen to be located within trimerization interfaces of the PCNA trimer. *S-*nitrosylation of PCNA might have an effect on the trimer formation and/or affect protein interactions with cdk/cyclins and other proteins that regulate DNA replication.

## Discussion

In NPrEC, we identified 116 protein-SNOs, with modification sites located for 86 of them by the use of a combined protocol involving both protein and peptide pull-down following the BST. Of the proteins identified, 60% belong to four functional categories: cell structure/cell motility/intracellular protein trafficking, protein folding/stress response/protein assembly, RNA splicing/processing/transcription regulation, and metabolisms. Thirty percent of the proteins identified have previously been reported to be nitrosylated in non-prostate tissues, and certain proteins have been shown to cause aberrant signaling processes and/or been associated with disease development. For example, several isomerases (triosephosphate isomerase 1 TIP1; glucose phosphate isomerase GPI, protein disulfide isomerase family A, member 3 and 6, PDI, proline 4-hydroxyase, P4HB) and oxidative stress proteins (e.g., peroxiredoxin) were identified as nitrosylated, among which SNO-PDI [40] and SNO-peroxiredoxin [39], [41] have been reported to serve as direct links between nitrosative/oxidative stress and neurodegenerative diseases. Although NS has been implicated in the pathogenesis of prostatic diseases [3], [9]–[14], its mechanism of action has not been established. The discovery of these protein-SNOs in this study support the notion that, depending on the level of NS, NO can induce modification on a specific subset of proteins that may play a role in the pathogenesis of prostate diseases.

A number of recent studies have described similar peptide pull-down approaches for the profiling of nitrocysteine-containing peptides in cells or lysates treated with either CysNO or nitrosoglutathione (GSNO) [24]–[27], [42]. Compared with these studies, which identified nitrosylated targets on the basis of one single biotinylated peptide, our study strengthened the stringency/confidence of the peptide-SNO identifications by also identifying the corresponding proteins in the protein pull-down. In fact, by taking advantage of the high mass accuracy measurement of the LTQ-Orbitrap, in our study, all the sequenced biotinylated peptides (Table S1A and S1B) have a low precursor mass error <5 ppm (although a peptide mass tolerance was set to <15 ppm during database searching), strongly supporting our peptide-SNO identifications. Of the sites of modification we identified in the study, 16% have been reported in the literature. These sites, together with the newly located sites, serve as a foundation for future investigations of how NO regulates the functions of the target proteins.

In our analysis of the 141 SNO sites, we did not find any consensus sequence motif in terms of primary or secondary structures. This is consistent with the previously reported results of primary sequence analysis of SNO proteins, including an attempt to derive predictive signatures by using machine-learning approaches by Hao and colleagues [27]. On the other hand, hydrophobicity analysis indicates that half of the SNO sites are located within a hydrophobic pocket, which is also in agreement with the findings of Greco and colleagues [25], who found that most of the 18 SNO sites they analyzed were located in hydrophobic pockets. We observed an enrichment of buried cysteine residues (79%) in SNO proteins, which may be indicative of the primary role of *S-*nitrosylation in regulating protein stability. In fact, many studies have demonstrated that nitrosylation can affect protein stability (e.g., HIF-1α [43], Bcl-2 [44])—some through the ubiquitin-proteasome system. For the SNO sites that can be mapped to structurally resolved structures, half of the buried cysteines were surrounded by spatially adjacent charged amino acids. Perez-Mato et al. first proposed an autocatalytic mechanism of nitrosylation that is facilitated by the charged amino acids in the vicinity of the cysteine thiol [37]. While this phenomenon has been demonstrated in a few proteins (e.g., MAT, 14-3-3θ, CLIC4) [19], [25], [36], [37], our data, with its larger collection of structures, further enhances the idea of an autocatalytic mechanism, at least in half of the cases. Finally, analysis on the mapped structures revealed that nitrosylation might affect the specific function of some proteins. In the 24 mapped structures, the function of several proteins has previously been demonstrated to be affected by nitrosylation. For example, in GAPDH, nitrosylation of C152, which is in the NAD binding pocket, has been shown to abolish the catalytic activity of GAPDH by recruiting the E3-ubiquitin-ligase Siah1 [38], [45]. Moreover, we also identified some new SNO sites, including those in EGFR and PCNA. The kinase activity of EGFR has previously been shown to be affected by NO [46], [47]. Structural analysis revealed that one of the SNO sites is involved in the ligand-binding surface of EGFR, and nitrosylation of this site may affect its ligand binding. For PCNA, since C81 is located at the trimer interface of PCNA, nitrosylation of this site may affect the trimerization/stability of the trimer ring formation. Although functional assays are necessary to confirm these hypotheses, mapping of SNOs into structures represents a strategy for predicting functional SNOs.

Considering that several groups have demonstrated the effects of NO on cell proliferation, migration, motility, adhesion, and aggressiveness in PCa cells [13], [14], [48], we decided to focus on a subset of protein-SNOs we identified that are related to cancer initiation and progression. We confirmed, by western-blot analysis, a total of six representative proteins, including the proliferating cell nuclear antigen (PCNA), karyopherin-β, α-catenin, integrin β4, maspin, and eEF1A1. PCNA is critical during chromosomal DNA replication; it has been found to be overexpressed in various types of cancers and that its expression is associated with poor survival outcomes [49]. Karyopherin-β1 is instrumental in nucleo-cytoplasmic transport of signaling molecules. It is overexpressed in cervical cancer [50], and its down-regulation impairs cell proliferation [51]. Integrin β4 is a transmembrane protein expressed predominantly on hemidesmosomes of epithelial cells. Similar to other members of the integrin family, integrin β4 mediates anchorage and migration of normal and cancer cells via influencing cell-matrix and cell-cell interactions [52]. α-Catenin forms the link between the β-catenin/E-cadherin complex and the actin cytoskeleton, hence playing a key role in maintaining cell adhesion [53]. Loss of integrins and catenins has been found in primary and metastatic PCa [54]. Maspin is a 42-kDa serine protease inhibitor with multifaceted tumor suppressive activities in breast, prostate, colon, and oral squamous cancers. Of interest is its biphasic expression pattern during carcinogenesis, with a loss of expression during early steps of tumorigenicity and re-expression in metastatic cancer [55]. Maspin-transfected PCa cells exhibit reduced tumorigenicity, vasculature, and metastatic potential under hypoxic conditions [56]. The eEF1 subunit 1A1 is involved in the binding of tRNA to ribosomes during protein synthesis. It has been found to be overexpressed in PCa [57] and implicated in tumorigenesis, signal transduction, and apoptosis [58]. While the effect of NO in the pathogenesis of BPH and PCa is unclear, our findings are concordant with the fact that signaling molecules important in cancer development are targets of nitrosylation and therefore may be involved in NS*-*induced initiation of the NPrEC. Although a number of apoptotic or cell cycle–related proteins whose activity or stability can be modulated by *S-*nitrosylation [17], SNO-Bcl-2 was shown to promote the malignant transformation of lung epithelial cells [59], supporting the role of NS in tumorigenesis [59], [60]. It is conceivable that the previously reported over-expression of iNOS [4]–[7], together with other oxidative insults [61], in the prostate epithelial and stromal compartments is likely to cause nitrosylation of the above mentioned protein targets, leading to modulation of cell growth, disruption of cellular architecture, and/or transformation of normal epithelial cells of the prostate.

Over-expression of NOS has been shown to promote tumorigenicity in other cancers, and the therapeutic application of NOS inhibitors for chemopreventive purposes has been the subject of intense research for the past decade [62]. Inhibition of NOS was recently shown to have tumor antivascular activity in patients with PCa [63], and the efficacy of NO-releasing drugs for BPH and lower urinary tract symptomatology was evaluated in a clinical trial based on the rationale that NO can relax muscular tone [64]. Given that the emerging evidence suggesting the importance of NS in carcinogenesis and inflammation-related diseases [59], [60], further elucidation of the functional significance of the targets identified in the present study should not only yield insights into details of the complex role of NO and inflammation-related prostatic diseases but also provide the experimental basis for NOS-related therapeutics.

## Supporting Information

Table S1 Table S1A identifies protein SNOs in both independent protein pull-down experiments. Table S1B identifies protein SNOs in one of the two protein pull-down experiments with the corresponding peptide SNO(s) identified in peptide pull-down.
(0.14 MB PDF)

Table S2 Mapping SNO sites into high-resolution Protein Data Bank (PDB) structures. Entrez gene symbols, gene names, PDB codes (and chain letters, assumed to be A if not given), and the number of *S*-nitrosylated cysteine residues in those structures are given, respectively.
(0.03 MB PDF)

Table S3 Analysis of structural features (secondary structure and solvent accessibility) of the 42 SNO sites mapped into 24 Protein Data Bank structures.
(0.05 MB PDF)

Table S4 Localization of neighboring charged residues in the vicinity of the SNO. Residues and atoms in contact with SNO cysteines in the mapped structures were identified with the iMolTalk server (http://i.moltalk.org) and the Loopp program, using a cutoff distance of 5 Å from the sulfur atom of the cysteine.
(2.47 MB PDF)

Figure S1 MS/MS spectra for all the biotinylated peptides identified. The MS/MS spectra of biotinylated peptides were evaluated by Scaffold (Proteome Software).
(1.67 MB PDF)
