# Supplementary material for: Comprehensive Identification and Modified-Site Mapping of S-Nitrosylated Targets in Prostate Epithelial Cells
Source: PLoS One. 2010 Feb 5;5(2):e9075. doi: 10.1371/journal.pone.0009075 (PMC2816712; doi:10.1371/journal.pone.0009075)
Supplement: Table S1 — A identifies protein SNOs in both independent protein pull-down experiments. Table S1B identifies protein SNOs in one of the two protein pull-down experiments with the corresponding peptide SNO(s) identified in peptide pull-down. (0.14 MB PDF) [file pone.0009075.s001.pdf]

Table S1A

IPI. No	Gene ID	Protein name	Protein Pull-down: Pull-Down I (< 1%FP) Prob/ (pro) # of pep[a)]	Protein Pull-down: Pull-Down II (< 1%FP) Prob (pro) # of pep	Peptide Pull-down: Biotinylated peptide [b,c,d)]	MH+	ΔM	z	Prob (pep)	XC	ΔCn	Ref
		Adhesion										
IPI00027422.1	ITGB4	integrin, beta 4	3.4E-09 (3)	1.5E-13 (6)								
		Cell structure / cell motility / intracellular protein traffic										
			3.9E-04 (2)	2.1E-05 (1)	K.GC#TDNLTLTVAR.S	1691.83	1.92	2	4.98E-06	3.12	0.42	
IPI00010414.4	PDLIM1	PDZ and LIM domain 1 (elfin)	1.8E-07 (4)	3.8E-07 (1)								
IPI00000230.6	TPM1	tropomyosin 1 (alpha)	5.5E-07 (7)	5.3E-07 (1)	R.EIVHLQAGQC#GNQIGAK.F [1]	2194.09	1.50	2	3.23E-07	3.31	0.59	[1,3]]
IPI00007752.1	TUBB2C	tubulin, beta 2C			K.LTTPTYGDLNHLVSATM*SGVTTC#LR.F[1]	3095.50	-1.49	3	1.96E-04	5.02	0.58	
			5.9E-10 (4)	1.6E-04 (1)	R.AVC#MLSNTTAVAEAWAR.L	2222.06	0.47	3	1.42E-03	2.60	0.31	[1] [2]
IPI00166768.3	TUBA1C	tubulin, alpha 1c			R.TIQFVDWC#PTGFK.V[1;2]	1969.94	3.23	2	6.73E-09	3.22	0.49	[2]
					K.AYHEQLTVAEITNAC#FEPANQM*VK.C[1;2]	3151.47	1.28	4	1.57E-03	2.74	0.15	
IPI00218918.5	ANXA1	annexin A1	2.3E-11 (7)	2.1E-07 (2)	K.M*YGISLC#QAILDETKGDYEK.I	2721.26	1.94	3	1.85E-07	3.24	0.58	
					K.ILVALC#GGN	1287.66	1.51	2	2.24E-03	2.20	0.48	[2,4,21]
IPI00418169.3	ANXA2	annexin A2	5.8E-07 (5)	3.8E-07 (3)	KGLGTDEDSLIEIIC*SRT [21]							[2,4]
IPI00334627.3	ANXA2P2	annexin A2 pseudogene 2	4.5E-08 (5)	4.3E-07 (2)	KGLGTDEDSLIEIIC*SRT	2149.03	3.40	2	1.16E-07	4.17	0.46	
						2149.03	0.35	3	6.80E-03	3.52	0.41	[13]
IPI00021766.5	RTN4	reticulon 4	3.5E-06 (1)	6.0E-13 (2)	K.YSNSALGHVNC#TIK.E	1934.93	3.47	2	1.19E-05	3.29	0.51	
IPI00014898.3	PLEC1	plectin 1, intermediate filament binding protein 500kDa	1.1E-06 (4)	8.9E-08 (17)	R.GC#LDEETSR.A	1437.62	-0.31	2	6.10E-05	2.59	0.43	
					R.C#VEDPETGLR.L	1546.71	1.74	2	1.31E-05	2.07	0.34	
					R.VPLDVAC#AR.G	1371.69	1.32	2	6.37E-07	2.30	0.36	
					R.C#RPDQLTGLSLLPLSEK.A	2298.20	0.85	3	1.05E-03	3.65	0.39	
					R.EYGSC#SHHYQQLLQSLEQGAQEESR.C	3335.49	1.14	4	9.56E-05	4.01	0.48	
					R.LPLLAVC#DYK.Q	1562.81	3.87	2	2.31E-05	2.40	0.40	
					R.LQLEAC#ETR.T	1490.72	4.24	2	9.21E-07	3.26	0.42	
IPI00013508.5	ACTN1	actinin, alpha 1	4.0E-05 (2)	8.0E-09 (5)	K.IC#DQWDNLGALTQK.R	2032.97	6.47	2	1.60E-08	4.83	0.44	
					K.C#QLEINFNTLQTK.L	1979.98	0.20	2	3.43E-06	3.09	0.42	[16]
IPI00418471.6	VIM	vimentin	4.0E-10 (11)	2.3E-05 (2)								
IPI00008603.1	ACTA2	actin, alpha 2, smooth muscle, aorta	1.4E-05 (2)	4.1E-04 (1)								[16,17]
IPI00021439.1	ACTB	actin, beta	8.2E-09 (9)	7.7E-09 (2)								
IPI00003269.1	ACTBL2	actin, beta-like 2	1.5E-10 (2)	9.0E-05 (1)								[2]
IPI00302592.2	FLNA	filamin A, alpha (actin binding protein 280)	3.7E-09 (15)	6.0E-07 (11)	K.ATC#APQHGAPGPGPADASK.V	2161.00	6.32	3	5.16E-07	3.64	0.52	
					R.C#SYQPTMEGVHTVHVTFAGVPIPR.S	3054.48	2.01	4	1.36E-04	3.25	0.31	
					K.C#APGVVGPAEADIDFDIIR.N	2386.16	2.05	3	2.85E-08	3.60	0.36	
					K.LQVEPAVDTSGVQC#YGPGIEGQGVFR.E	3134.51	4.78	3	3.95E-09	3.94	0.57	[2]
IPI00289334.1	FLNB	filamin B, beta (actin binding protein 278)	3.3E-05 (7)	1.2E-06 (6)	K.VAVTEGC#QPSR.V	1574.75	2.61	2	5.79E-07	2.46	0.47	
IPI00021405.3	LMNA	lamin A/C	2.4E-05 (4)	1.6E-06 (5)								
IPI00217975.4	LMNB1	lamin B1	3.5E-05 (3)	2.9E-05 (2)								
IPI00215948.4	CTNNA1	catenin (cadherin-associated protein), alpha 1, 102kDa	1.0E+00 (2)	9.2E-09 (6)								
		Cell proliferation										
IPI00022078.3	NDRG1	N-myc downstream regulated gene 1	2.5E-08 (1)	6.6E-07 (1)								[5]
IPI00001639.2	KPNB1	karyopherin (importin) beta 1	9.3E-05 (1)	5.6E-04 (1)								
		Immunity and defense										
IPI00005198.2	ILF2	interleukin enhancer binding factor 2, 45kDa	1.6E-06 (5)	4.9E-05 (1)								
IPI00396321.1	LRRC59	leucine rich repeat containing 59	4.5E-04 (2)	5.7E-08 (2)								
		Ion Transporter										
IPI00216308.5	VDAC1	voltage-dependent anion channel 1	1.2E-04 (1)	1.9E-06 (1)								
		Metabolism										
IPI00025366.4	CS	citrate synthase	5.1E-06 (1)	8.5E-05 (2)	K.LPC#VAAK.I	1129.59	0.14	2	9.79E-01	2.62	0.35	
IPI00172460.4	AK2	adenylate kinase 2	2.5E-05 (1)	4.9E-06 (1)								[6]
IPI00008982.1	ALDH18A1	aldehyde dehydrogenase 18 family, member A1	5.8E-04 (1)	1.1E-04 (1)	K.YQIDPDAC#FSAK.V	1785.80	1.08	2	2.11E-06	3.24	0.53	[7]
IPI00169383.3	PGK1	phosphoglycerate kinase 1	4.3E-11 (6)	2.8E-09 (1)	K.DC#VGPEVEK.A	1403.64	1.84	2	4.81E-03	2.14	0.39	[13, 18]
					K.AC#ANPAAGSVILLENLR.F	2140.11	-0.66	3	1.07E-04	3.17	0.48	
					K.AC#ANPAAGSVILLENLR.F	2140.11	2.02	2	4.52E-10	5.31	0.58	
IPI00465248.5	ENO1	enolase 1, (alpha)	2.1E-06 (6)	7.2E-09 (2)								[8]
IPI00020944.1	FDFT1	farnesyl-diphosphate farnesyltransferase 1	2.0E-05 (1)	6.7E-08 (1)								[1]
		Miscellaneous										

IPI00017297.1	MATR3	matrin 3	3.1E-04 (1)	3.0E-04 (2)	K.LC#SLFYTNEEVAK.N	1944.93	2.46	2	2.12E-06	3.43	0.39	
IPI00658109.1	CKMT1A	creatine kinase, mitochondrial 1A	5.0E-07 (2)	6.9E-10 (1)								[19]
		mRNA splicing / processing / transcription regulation										
IPI00012074.3	HNRNPR	heterogeneous nuclear ribonucleoprotein R	1.5E-05 (1)	3.3E-04 (1)	R.GFC#FLEYEDHK.S	1815.79	3.82	3	1.24E-04	3.53	0.31	
IPI00011274.3	HNRPDL	heterogeneous nuclear ribonucleoprotein D-like	1.9E-04 (1)	4.1E-05 (1)	R.FGEVVDC#TIK.T	1538.74	4.42	2	7.86E-04	2.39	0.40	
IPI00031556.7	U2AF2	U2 small nuclear RNA auxiliary factor 2	5.5E-07 (1)	1.5E-05 (1)								
IPI00479786.5	KHSRP	KH-type splicing regulatory protein (FUSE binding protein 2)	2.5E-07 (3)	8.1E-09 (5)	K.IQQDSGC#K.V	1306.60	0.60	2	1.00E+00	2.59	0.33	
					K.C#GLVIGR.G	1145.60	3.51	2	1.00E+00	2.02	0.18	
IPI00021187.4	RUVBL1	RuvB-like 1	3.7E-08 (4)	1.1E-06 (1)								
IPI00025815.2	TARDBP	TAR DNA binding protein	2.6E-10 (1)	6.4E-08 (1)								
IPI00216049.1	HNRNPK	heterogeneous nuclear ribonucleoprotein K	2.6E-07 (6)	1.4E-08 (3)								
IPI00171903.2	HNRNPM	heterogeneous nuclear ribonucleoprotein M	1.0E+00 (1)	4.5E-06 (2)	K.DKFNEC#GHVLYADIK.M	2180.03	3.88	3	5.31E-09	2.79	0.36	
		Protein disulfide-isomerase reaction										
IPI00010796.1	P4HB	proline 4-hydroxylase, beta polypeptide	4.8E-07 (4)	1.6E-09 (3)	K.KEEC#PAVR.L	1359.66	1.60	2	1.00E+00	2.64	0.11	
IPI00025252.1	PDIA3	protein disulfide isomerase family A, member 3	1.8E-08 (1)	1.4E-07 (4)	K.KFIQENIFGIC#PHMTEDNKDLIQGK.D	3346.65	1.88	4	1.35E-05	4.43	0.32	[9]
					K.FIQENIFGIC#PHM*TEDNKDLIQGK.D	3234.55	-0.41	4	5.62E-03	2.56	0.25	
					K.KFIQENIFGIC#PHM*TEDNKDLIQGK.D	3362.64	-0.11	4	5.37E-07	4.82	0.53	
					K.FIQENIFGIC#PHMTEDNKDLIQGK.D	3218.55	2.25	4	4.29E-06	2.58	0.20	
IPI00299571.5	PDIA6	protein disulfide isomerase family A, member 6	1.7E-05 (2)	2.4E-05 (1)								[9]
		Protein folding / stress response / Protein assembly										
IPI00020984.2	CANX	calnexin	8.1E-06 (3)	2.9E-08 (2)								
IPI00010720.1	CCT5	chaperonin containing TCP1, subunit 5 (epsilon)	2.6E-04 (2)	2.6E-08 (1)								
IPI00013122.1	CDC37	cell division cycle 37 homolog (S. cerevisiae)	6.2E-04 (1)	2.7E-09 (1)								
IPI00784154.1	HSPD1	heat shock 60kDa protein 1 (chaperonin)	8.7E-10 (2)	5.9E-08 (3)	K.C#EFQDAYVLLSEK.K	1972.92	0.27	3	2.35E-05	2.66	0.17	
					K.C#EFQDAYVLLSEK.K	1972.92	0.68	2	3.93E-06	4.15	0.52	
					R.<u>AAVEEGIVLGGGC#ALLR</u>.C [1]	2056.08	3.21	3	1.24E-05	2.75	0.44	[1]
IPI00382470.3	HSP90AA1	heat shock protein 90kDa alpha (cytosolic), class A member 1	6.8E-11 (4)	9.5E-06 (3)	K.C#LELFTELAEDKENYKK.F	2501.21	0.62	4	7.19E-04	3.34	0.29	[10]
IPI00030275.5	TRAP1	TNF receptor-associated protein 1	1.1E-08 (1)	3.2E-05 (4)								
IPI00027230.3	HSP90B1	heat shock protein 90kDa beta (Grp94), member 1	1.2E-08 (4)	2.1E-05 (6)								[10]
IPI00013894.1	STIP1	stress-induced-phosphoprotein 1 (Hsp70/Hsp90-organizing protein)	3.8E-04 (6)	3.1E-05 (1)								
IPI00003362.2	HSPA5	heat shock 70kDa protein 5 (glucose-regulated protein, 78kDa)	3.1E-09 (2)	1.2E-11 (6)								
IPI00003865.1	HSPA8	heat shock 70kDa protein 8	1.0E+00 (3)	9.3E-06 (1)	K.C#NEIINWLDKNQTAEKEEFEHQQK.E	3402.59	3.35	4	2.33E-03	3.21	0.21	[10]
					K.C#NEIINWLDK.N	1675.80	2.56	2	1.51E-05	3.18	0.32	
					K.VC#NPIITK.L	1315.69	4.22	2	3.79E-04	2.79	0.31	
IPI00007765.5	HSPA9	heat shock 70kDa protein 9 (mortalin)	3.8E-12 (13)	1.9E-10 (15)	K.MEEFKDQLPADEC#NK.L	2224.97	2.41	3	1.11E-08	2.73	0.24	[20]
					K.VC#QGER.E	1119.51	-0.95	2	9.99E-01	2.34	0.11	
IPI00025512.2	HSPB1	heat shock 27kDa protein 1	4.9E-07 (4)	1.2E-08 (1)								
		Proteolysis										
IPI00479722.2	PSME1	proteasome (prosome, macropain) activator subunit 1 (PA28 alpha)	6.0E-05 (2)	7.5E-07 (1)								
IPI00032140.4	SERPINH1	serpin peptidase inhibitor, clade H (heat shock protein 47), member 1	6.2E-12 (7)	6.1E-08 (7)								
IPI00644196.1	SERPINB5	serpin peptidase inhibitor, clade B (ovalbumin), member 5	2.9E-08 (3)	9.1E-10 (1)								
		Transport										
IPI00001639.2	KPNB1	karyopherin (importin) beta 1	9.3E-05 (1)	5.6E-04 (1)	K.GVLFYGPPGC#GK.T	1622.79	1.25	2	1.30E-07	2.45	0.41	
IPI00022774.3	VCP	valosin-containing protein	8.3E-05 (5)	6.7E-05 (4)	R.LGDVISIQPC#PDVK.Y	1911.97	-0.20	2	6.61E-06	3.75	0.56	

a) # of pep: number of peptides identified for each protein according to the criteria of XCorr values of 2.0 (2+) and 2.5 (3+), ΔCn $\geq$ 0.1 and Δ mass precursor < 10 ppm (including the same peptide with different charge states.

b) C#: Cysteine modified by biotin-HPDP (+428.191567 amu); C@: Cysteine modified by MMTS (+45.98772 amu).

c) For each protein, only the biotinylated peptides with the highest score from the multiple peptide pull-downs are shown.

d) Underlined are the sequences have been reported in the literature.

Table S1B

IPI. No	Gene ID	Protein name	Protein Pull-down Run I (< 1%FP) Prob/(pro) (# of pep)	Protein Pull-down Run II (< 1%FP) Prob (pro) (# of pep)	Peptide Pull-down Biotinylated peptide	MH+	ΔM	z	Prob (pep)	XC	ΔCn	Ref
		Actin binding protein										
IPI00015262.10	CNN2	calponin 2		9.27E-11(1)	K.AGQC#VIGLQMGTNK.C	1847.90	4.68	2	1.24E-06	2.98	0.40	
					K.DGTILC#TLMNK.L	1636.79	-2.18	2	4.93E-02	2.43	0.25	
		Antioxidant / free radical removal										
IPI00024915.2	PRDX5	Peroxiredoxin 5		1.09E-04 (1)	K.GVLFGVPGAFTPGC#SK.T	1964.98	0.65	3	7.78E-04	2.79	0.31	
IPI00000874.1	PRDX1	peroxiredoxin 1	2.88E-04 (2)		K.HGEVC#PAGWK.P	1511.70	-0.63	3	2.70E-02	3.24	0.49	
					K.HGEVC#PAGWKPGSDTIKPDVQK.S [11]	2777.36	4.19	3	1.85E-04	3.46	0.41	[11]
		Adhesion										
IPI00010697.2	ITGA6	integrin, alpha 6		6.58E-07 (3)	K.VVTC#AHR.Y	1213.60	-0.48	2	2.39E-04	2.12	0.20	
					R.TGGLYSC#DITAR.G	1684.79	2.85	2	6.89E-09	2.77	0.53	
					K.SC#FEYTANPAGYNPSISIVGTLEAEK.E	3189.49	0.42	3	9.22E-02	3.53	0.35	
IPI00219219.3	LGALS1	lectin, galactoside-binding, soluble, 1 (galectin 1)		2.83E-05 (1)	R.FNAHGDANTIVC#NSK.D	2018.92	3.51	2	2.24E-11	3.67	0.66	
					K.DSNNLC#LHFNPR.F	1857.86	1.16	3	1.91E-05	3.00	0.40	
		DNA Repair / DNA replication										
IPI00220834.8	XRCC5	X-ray repair complementing defective repair	3.88E-05 (1)		R.HSIHWPC#R.L	1463.69	3.61	3	3.03E-05	2.82	0.42	
		in Chinese hamster cells 5 (double-strand-break rejoining			R.LFQC#LLHR.A	1457.76	3.89	2	1.16E-03	2.27	0.35	
IPI00021700.3	PCNA	proliferating cell nuclear antigen	2.14E-07 (2)		K.C#AGNEDIITLR.A	1632.79	2.07	2	6.51E-05	3.11	0.52	
					R.DLSHIGDAVVISC#AK.D [2]	1955.98	0.07	3	1.37E-02	2.96	0.21	[2]
		Ion Transport										
IPI00024145.2	VDAC2	voltage-dependent anion channel 2		8.89E-09 (3)	K.SC#SGVEFSTSGSSNTDTGK.V	2278.96	1.82	2	2.99E-07	4.56	0.67	[13]
IPI00031804.1	VDAC3	voltage-dependent anion channel 3		4.77E-06 (1)	K.VC#NYGLTFTQK.W	1701.82	1.14	2	1.89E-02	2.44	0.22	[13]
IPI00010896.3	CLIC1	chloride intracellular channel 1	2.74E-08 (3)		K.LHIVQVVC#K.K	1466.80	3.25	3	3.09E-04	2.51	0.46	
IPI00177817.4	ATP2A2	ATPase, Ca++ transporting, cardiac muscle, slow twitch 2		7.80E-10 (3)	R.C#LALATHDNPLR.R	1751.88	-2.55	3	7.63E-05	4.23	0.51	[1]
		Metabolism										
IPI00465439.5	ALDOA	aldolase A, fructose-bisphosphate	1.75E-10 (6)		R.ALANSLAC#QGK.Y	1503.75	0.62	2	6.67E-07	2.67	0.43	[10]
					R.VNPC#IGGVILFHETLYQK.A	2459.26	1.28	3	2.76E-10	3.59	0.46	
					K.ALSDHHIYLEGTLLKPNM*VTPGHAC#TQK.F	3518.74	-0.58	5	7.57E-04	2.77	0.14	
					K.ALSDHHIYLEGTLLKPNMVTPGHAC#TQK.F	3502.75	-5.80	5	1.16E-06	3.53	0.28	
IPI00847989.2	PKM2	pyruvate kinase, muscle	1.95E-10 (3)		R.NTGIIC#TIGPASR.S [11]	1730.88	2.70	2	1.15E-05	3.09	0.35	[1, 10,
					R.AGKPVIC#ATQM*LESM*IK.K	2280.13	1.44	3	2.52E-04	4.08	0.57	11]
					K.PVIC#ATQMLESMIK.K	1991.99	3.86	2	1.75E-06	3.51	0.60	
					R.AGKPVIC#ATQMLESMIK.K	2248.14	1.72	3	1.29E-08	4.30	0.59	
					R.AEGSDVANAVLDGADC#IMLSGETAK.G [1]	2865.31	2.41	3	2.80E-07	3.77	0.56	
					K.C#DENILWLDYK.N	1839.85	1.41	2	1.24E-01	2.22	0.36	
					R.AGKPVIC#ATQMLESM*IK.K	2264.13	2.95	3	4.65E-08	4.46	0.36	
					R.AGKPVIC#ATQM*LESMIK.K	2264.13	2.95	3	9.50E-04	2.85	0.55	
IPI00383071.1	TPI1	triosephosphate isomerase 1	5.69E-05 (1)		K.DC#GATWVVLGHSER.R	1957.91	2.16	3	2.15E-06	3.43	0.52	[1,10]
					K.VPADTEVVC#APPTAYIDFAR.Q	2563.24	4.85	2	9.72E-06	3.57	0.53	
					K.VAHALAEGLGVIAC#IGEK.L	2179.14	1.88	3	3.68E-03	3.35	0.42	
					K.GALEGSSC#PFR.T	1551.71	3.99	2	6.49E-05	2.33	0.32	
IPI00026824.2	HMOX2	heme oxygenase (decycling) 2		5.88E-04 (1)	R.DIDFLKEEEHDC#FLEEIM*TK.R	2928.32	3.10	3	2.57E-06	3.59	0.37	
IPI00291510.3	IMPDH2	IMP (inosine monophosphate) dehydrogenase 2	1.31E-05 (4)		K.M*IPC#DFLIPVQTQHPIR.K	2452.24	0.10	3	7.28E-06	3.14	0.37	
IPI00027497.5	GPI	glucose phosphate isomerase	1.00E+00 (1)		R.FNHFSLTLNTNHGHILVDYSK.N	2457.23	1.25	4	1.15E-04	3.87	0.52	[10]
					K.MIPC#DFLIPVQTQHPIR.K	2436.24	-0.09	3	9.72E-04	2.84	0.38	
					K.C#TTDHISAAGPWLK.F	1927.92	4.31	3	4.40E-05	3.86	0.46	
IPI00017855.1	ACO2	aconitase 2, mitochondrial		2.44E-06 (2)	K.GC#DVVVIPAGVPR.K	1709.89	2.14	2	1.27E-02	2.70	0.45	
IPI00291006.2	MDH2	malate dehydrogenase 2, NAD (mitochondrial)	1.00E+00 (1)		K.GYLGPEQLPDC#LK.G	1860.91	4.35	2	2.68E-08	2.47	0.51	[1,
					K.SQETEC#TYFSTPLLLGK.K	2345.12	0.12	2	5.35E-03	2.59	0.50	10,11,12]
					K.VAVLGASGGIGQPLSLLLK.N	1793.09	1.32	2	2.02E-09	5.27	0.80	
		mRNA splicing / processing / transcription										
IPI00163505.2	RBM39	RNA binding motif protein 39	8.99E-08 (2)		K.C#PSIAAAIAAVNALHGR.W	2063.07	0.71	3	2.65E-07	3.85	0.63	
IPI00844578.1	DHX9	DEAH (Asp-Glu-Ala-His) box polypeptide 9	1.00E+00 (1)		K.SSVNC#PFSSQDMK.Y	1857.80	2.18	2	1.44E-03	3.02	0.34	
					K.NFLYAWC#GK.R	1529.71	1.80	2	1.08E-04	2.34	0.11	
IPI00215965.2	HNRNPA1	heterogeneous nuclear ribonucleoprotein A1	1.50E-08 (1)		R.SHFEQWGTLTDC#VVM*R.D	2353.06	1.25	3	5.10E-06	3.49	0.51	
					R.SHFEQWGTLTDC#VVMR.D	2337.06	0.05	3	4.61E-02	3.32	0.38	
IPI00054042.1	GTF2I	general transcription factor II, I		2.14E-08 (1)	K.MVDQLFC#K.K	1411.66	4.15	2	1.45E-04	2.68	0.45	
IPI00150057.6	SMARCC2	SWI/SNF regulator of chromatin, subfamily c, member 2	1.00E+00 (1)		R.PNIFLC#PEIEPK.L	1827.92	0.27	2	2.88E-04	2.77	0.33	
IPI00013881.6	HNRNPH1	heterogeneous nuclear ribonucleoprotein H1 (H)		5.62E-05 (1)	R.DLNYC#FSGMSDHR.Y	1972.82	3.65	3	2.76E-04	2.63	0.51	

IPI00027834.3	HNRNPL	heterogeneous nuclear ribonucleoprotein L	7.68E-06 (1)		K.LC#FSTAQHAS	1492.67	4.47	2	4.79E-07	2.25	0.49	[2]
		Proteolysis										
IPI00220857.3	CAST	calpastatin (proteolysis)	1.98E-04 (1)		K.AAAPAPVSEAVC#R.T	1669.82	-0.25	2	2.02E-09	2.83	0.59	
					K.KPADDQDPIDALSGDLDSC#PSTTETSQNTAK.D	3648.63	2.91	3	5.34E-08	6.15	0.66	
		Protein biosynthesis/ translational regulation										
IPI00297982.7	EIF2S3	eukaryotic translation initiation factor 2, subunit 3 gamma, 52kDa	6.18E-08 (1)		K.YNIEVVC#EYIVK.K	1899.94	4.18	2	2.67E-04	2.83	0.46	
IPI00186290.6	EEF2	eukaryotic translation elongation factor 2	2.03E-10 (3)		K.STLTDSLVC#K.A	1494.74	0.60	2	1.60E-05	2.41	0.38	[8]
					K.IWC#FGPDGTGPNILTDITK.G	2476.21	7.35	3	6.70E-01	3.03	0.38	
					R.C#ELLYEGPPDDEAAMGIK.S	2379.07	4.48	3	4.89E-04	3.06	0.38	
IPI00000494.6	RPL5	ribosomal protein L5	1.00E+00 (1)		R.IEGDM*IVC#AAYAHELPK.Y	2304.09	2.87	3	6.43E-02	3.63	0.31	
					K.VGLTNYAAAYC#TGLLLAR.R	2298.18	-0.08	3	2.27E-04	2.69	0.35	
					R.IEGDMIVC#AAYAHELPK.Y	2288.09	1.33	3	3.69E-07	3.02	0.27	
IPI00027107.5	TUFM	Tu translation elongation factor, mitochondrial		5.06E-06 (2)	K.KGDEC#ELLGHSK.N	1743.82	1.20	4	6.09E-06	3.05	0.40	
IPI00011253.3	RPS3	ribosomal protein S3	1.00E+00 (1)		R.GLC#AIAQAESLR.Y [1, 11]	1659.84	2.45	2	1.83E-06	3.29	0.47	[1] [11]
IPI00025874.2	RPN1	ribophorin I	2.34E-05 (2)		K.VAC#ITEQVLTLVNK.R	1959.05	2.36	2	9.15E-05	3.99	0.45	
IPI00025447.8	EEF1A1	eukaryotic translation elongation factor 1 alpha 1	9.96E-01 (1)		K.SGDAAIVDMVPGKPM*C#VESFSDYPPLGR.F[2]	3382.56	-1.31	3	6.16E-07	3.01	0.36	[2,8,
					K.SGDAAIVDM*VPGKPMC#VESFSDYPPLGR.F[2]	3382.56	-3.18	4	1.31E-02	2.95	0.41	10,11]
					K.SGDAAIVDM*VPGKPM*C#VESFSDYPPLGR.F[2]	3398.56	5.18	3	1.29E-09	3.61	0.53	
		Protein folding / stress reponse / complex assembly										
IPI00002966.2	HSPA4	heat shock 70kDa protein 4	1.17E-09 (2)		R.KFDEVLVNHFC#EEFGK.K	2369.11	-0.41	4	2.35E-08	3.58	0.40	[10]
					R.KFDEVLVNHFC#EEFGK.K	2369.11	2.00	3	1.35E-04	3.11	0.20	
IPI00414676.6	HSP90AB1	heat shock protein 90kDa alpha (cytosolic), class B member 1	6.26E-13 (5)		K.C#LELFSELAEDKENYK.K	2359.10	0.10	3	2.35E-05	3.56	0.44	[10]
					K.C#LELFSELAEDKENYKK.F	2487.20	-0.95	3	4.89E-05	4.29	0.43	
IPI00302927.6	CCT4	chaperonin containing TCP1, subunit 4 (delta)	1.93E-04 (1)		K.TGC#NVLLIQK.S	1516.80	0.51	2	1.07E-04	2.46	0.11	[11]
					K.ITGC#ASPGK.T	1261.61	2.63	2	8.33E-01	2.40	0.17	
IPI00290566.1	TCP1	t-complex 1	1.50E-08 (4)		K.VLC#ELADLQDK.E	1674.83	2.56	2	1.36E-06	3.49	0.31	[2,11]
					R.IC#DDELILIK.N [11]	1602.83	2.37	2	3.79E-03	2.31	0.24	
IPI00290770.3	CCT3	chaperonin containing TCP1, subunit 3 (gamma)	2.65E-05 (2)		R.WSSLAC#NIALDAVK.M	1918.96	3.01	2	3.79E-05	2.15	0.38	
					K.IPGGIIEDSC#VLR.G [2]	1799.92	2.81	2	2.64E-07	3.25	0.48	[2]
IPI00018465.1	CCT7	chaperonin containing TCP1, subunit 7 (eta)	1.24E-05 (1)		R.YNFFTGC#PK.A	1504.68	1.12	2	3.58E-03	2.20	0.22	[11]
					R.QLC#DNAGFDATNILNK.L	2165.02	1.66	3	2.09E-05	3.95	0.32	
		Protein phosphorylation										
IPI00783271.1	LRPPRC	leucine-rich PPR-motif containing		2.01E-06 (4)	R.C#VANNQVETLEK.L	1775.85	-0.65	2	8.73E-04	4.04	0.52	
IPI00006167.1	PPM1G	protein phosphatase 1G (formerly 2C),	2.54E-04 (1)		K.C#SGDGVGAPR.L	1346.60	2.82	2	1.00E+00	2.37	0.27	
IPI00018274.1	EGFR	epidermal growth factor receptor		1.17E-07 (5)	R.AC#GADSYEM*EEDGVR.K	2075.82	5.17	2	3.64E-08	4.44	0.72	[22]
					K.YSFGATC#VK.K	1403.65	1.59	2	8.67E-07	2.39	0.46	
					K.NLC#YANTINWK.K	1767.84	4.03	2	9.26E-06	3.47	0.41	
		Structure and motility										
IPI00215743.3	RRBP1	ribosome binding protein 1 homolog 180kDa		3.76E-06 (4)	R.ELC#HTQSSHASLR.A	1896.89	-0.69	3	7.59E-04	2.59	0.44	[13]
					K.LTAEFEEAQTSAC#R.L	1983.90	-0.31	3	1.38E-04	3.07	0.41	
IPI00010779.4	TPM4	tropomyosin 4	1.23E-07 (2)		K.C#GDLEEELK.N	1463.66	2.26	2	8.08E-04	2.79	0.35	[14, 23]
IPI00013808.1	ACTN4	actinin, alpha 4		3.06E-04 (1)	K.IC#DQWDALGSLTHSR.R	2129.99	1.69	3	1.71E-08	5.37	0.65	[10,11]
					K.AC#LISLGYDVENDR.Q	1995.93	5.44	2	2.80E-06	3.22	0.67	
IPI00178352.5	FLNC	filamin C, gamma (actin binding protein 280)	1.00E+00 (1)		K.GAGTGGLGLTVEGPC#EAK.I	2044.99	-0.59	3	4.69E-02	2.54	0.41	[2]
					K.GAGTGGLGLTVEGPC#EAK.I	2044.99	2.85	2	9.40E-04	3.60	0.53	
IPI00007750.1	TUBA4A	tubulin, alpha 4a	3.20E-08 (7)		R.SIQFVDWC#PTGFK.V [1]	1955.92	0.64	3	3.01E-07	4.07	0.54	[1,8,10]
					R.SIQFVDWC#PTGFK.V [1]	1955.92	1.27	2	1.07E-07	3.25	0.62	
					R.AVC#M*LSNTTAIAEAWAR.L[1]	2252.07	5.12	3	4.66E-09	3.15	0.38	
					K.AYHEQLSVAEITNAC#FEPANQM*VK.C [1]	3137.46	3.34	3	2.67E-05	3.66	0.48	
					K.TIGGGDDSFTTFFC#ETGAGK.H	2439.07	0.68	2	6.50E-04	2.76	0.50	
					R.AVC#MLSNTTAIAEAWAR.L[1]	2236.07	1.60	3	4.60E-05	3.92	0.35	
					R.AVC#MLSNTTAIAEAWAR.L [1]	2236.07	2.40	2	6.00E-10	4.61	0.55	
					R.AVC#M*LSNTTAIAEAWAR.L [1]	2252.07	3.01	2	1.56E-06	3.63	0.50	
		Signal transduction										
IPI00018146.1	YWHAQ	tyrosine 3-monooxygenase/tryptophan 5-monooxygenase activation protein, theta polypeptide	4.20E-08 (2)		R.YLAEVAC#GDDR.K	1639.73	3.22	2	3.20E-06	2.76	0.51	[3]
		Unclassified										
IPI00550069.3	RNH1	ribonuclease/angiogenin inhibitor 1	2.93E-05 (1)		R.LDDC#GLTEAR.C	1520.69	5.32	2	6.06E-05	2.88	0.41	[2]
					K.LGDVGMAELC#PGLLHPSSR.L	2380.16	-1.02	3	3.68E-08	3.25	0.44	
IPI00021812.2	AHNAK	AHNAK nucleoprotein (desmoyokin)		6.40E-05 (4)	K.VDVEC#PDVNIEGPEGK.W	2127.98	2.62	2	4.83E-07	3.76	0.56	
					K.LEGDLTGPSVGVEVPDVELEC#PDAK.L	2996.43	2.64	3	3.72E-09	4.49	0.49	
					K.GPFVEAEVPDVDLEC#PDAK.L	2458.13	-0.56	3	2.40E-01	2.90	0.34	
IPI00008530.1	RPLP0	ribosomal protein, large, P0	8.44E-06 (6)		K.C#FIVGADNVGSK.Q	1637.78	2.48	2	2.23E-06	3.95	0.43	[2,11]
IPI00219018.7	GAPDH	glyceraldehyde-3-phosphate dehydrogenase	4.62E-08 (1)		R.VPTANVSVVDLTC#R.L [2]	1901.96	-0.06	3	4.86E-03	2.62	0.44	[1,2,8,
					R.VPTANVSVVDLTC#R.L[2]	1901.96	2.74	2	5.57E-13	4.11	0.58	11,13,15]

IPI00141318.2	CKAP4	cytoskeleton-associated protein 4		1.04E-09 (1)	K.IISNASC#TTNC#LAPLAK.V [1,2,11]	2194.06	5.95	2	2.38E-07	2.67	0.50	[2]
					K.SSSSSSASAAAAAAAASSSASC#SR.R	2461.08	4.29	2	7.89E-11	3.79	0.65	
					K.SSSSSSASAAAAAAAASSSASC#SR.R	2461.08	3.34	3	1.02E-12	3.89	0.60	[1]
IPI00645078.1	UBA1	ubiquitin-like modifier activating enzyme 1	1.48E-09 (4)		K.DNPGVVTC#LDEAR.H	1816.84	2.23	2	1.69E-06	3.52	0.62	
IPI00029656.1	DFNA5	deafness, autosomal dominant 5	3.82E-05 (3)		R.NEVLC#VLTQK.I	1574.81	3.61	2	3.85E-05	2.80	0.44	

a) # of pep: number of peptides identified for each protein according to the criteria of XCorr values of 2.0 (2+) and 2.5 (3+), ΔCn ≥ 0.1 and Δ mass precursor < 10 ppm (including the same peptide with different charge states.

b) C#: Cysteine modified by biotin-HPDP (+428.191567 amu); C@: Cysteine modified by MMTS (+45.98772 amu).

c) For each protein, only the biotinylated peptides with the highest score from the multiple peptide pull-downs are shown.

d) Underlined are the sequences have been reported in the literature.

Reference List

1. Hao,G., Derakhshan,B., Shi,L., Campagne,F., and Gross,S.S. (2006) SNOSID, a proteomic method for identification of cysteine S-nitrosylation sites in complex protein mixtures. *Proc.Natl.Acad.Sci.U.S.A*, **103**, 1012-1017.

2. Forrester,M.T., Thompson,J.W., Foster,M.W., Nogueira,L., Moseley,M.A., and Stamler,J.S. (2009) Proteomic analysis of S-nitrosylation and denitrosylation by resin-assisted capture. *Nat.Biotechnol.*, **27**, 557-559.

3. Chen,S.C., Huang,B., Liu,Y.C., Shyu,K.G., Lin,P.Y., and Wang,D.L. (2008) Acute hypoxia enhances proteins' S-nitrosylation in endothelial cells. *Biochem.Biophys.Res.Commun.*, **377**, 1274-1278.

4. Liu,L., Enright,E., Sun,P., Tsai,S.Y., Mehta,P., Beckman,D.L., and Terrian,D.M. (2002) Inactivation of annexin II tetramer by S-nitrosoglutathione. *Eur.J.Biochem.*, **269**, 4277-4286.

5. Contestabile,A. (2008) Regulation of transcription factors by nitric oxide in neurons and in neural-derived tumor cells. *Prog.Neurobiol.*, **84**, 317-328.

6. Shi,Q., Feng,J., Qu,H., and Cheng,Y.Y. (2008) A proteomic study of S-nitrosylation in the rat cardiac proteins in vitro. *Biol.Pharm.Bull.*, **31**, 1536-1540.

7. Moon,K.H., Kim,B.J., and Song,B.J. (2005) Inhibition of mitochondrial aldehyde dehydrogenase by nitric oxide-mediated S-nitrosylation. *FEBS Lett.*, **579**, 6115-6120.

8. Gao,C., Guo,H., Wei,J., Mi,Z., Wai,P.Y., and Kuo,P.C. (2005) Identification of S-nitrosylated proteins in endotoxin-stimulated RAW264.7 murine macrophages. *Nitric.Oxide.*, **12**, 121-126.

9. Uehara,T., Nakamura,T., Yao,D., Shi,Z.Q., Gu,Z., Ma,Y., Masliah,E., Nomura,Y., and Lipton,S.A. (2006) S-nitrosylated protein-disulphide isomerase links protein misfolding to neurodegeneration. *Nature*, **441**, 513-517.

10. Camerini,S., Polci,M.L., Restuccia,U., Usuelli,V., Malgaroli,A., and Bachi,A. (2007) A novel approach to identify proteins modified by nitric oxide: the HIS-TAG switch method. *J.Proteome.Res.*, **6**, 3224-3231.

11. Han,P. and Chen,C. (2008) Detergent-free biotin switch combined with liquid chromatography/tandem mass spectrometry in the analysis of S-nitrosylated proteins. *Rapid Commun.Mass Spectrom.*, **22**, 1137-1145.

12. Sun,J., Morgan,M., Shen,R.F., Steenbergen,C., and Murphy,E. (2007) Preconditioning results in S-nitrosylation of proteins involved in regulation of mitochondrial energetics and calcium transport. *Circ.Res.*, **101**, 1155-1163.

13. Lefievre,L., Chen,Y., Conner,S.J., Scott,J.L., Publicover,S.J., Ford,W.C., and Barratt,C.L. (2007) Human spermatozoa contain multiple targets for protein S-nitrosylation: an alternative mechanism of the modulation of sperm function by nitric oxide? *Proteomics.*, **7**, 3066-3084.

14. Kuncewicz,T., Sheta,E.A., Goldknopf,I.L., and Kone,B.C. (2003) Proteomic analysis of S-nitrosylated proteins in mesangial cells. *Mol.Cell Proteomics.*, **2**, 156-163.

15. Hara,M.R., Agrawal,N., Kim,S.F., Cascio,M.B., Fujimuro,M., Ozeki,Y., Takahashi,M., Cheah,J.H., Tankou,S.K., Hester,L.D., Ferris,C.D., Hayward,S.D., Snyder,S.H., and Sawa,A. (2005) S-nitrosylated GAPDH initiates apoptotic cell death by nuclear translocation following Siah1 binding. *Nat.Cell Biol.*, **7**, 665-674.

16. Huang, B., Chen, S. C. & Wang, D. L. Shear flow increases S-nitrosylation of proteins in endothelial cells. *Cardiovasc. Res.* (2009).

17. Thom, S. R., Bhopale, V. M., Mancini, D. J. & Milovanova, T. N. Actin S-Nitrosylation Inhibits Neutrophil {beta} 2 Integrin Function. *J. Biol. Chem.* **283**, 10822 (2008).

18. Fratelli, M. *et al*. Identification by redox proteomics of glutathionylated proteins in oxidatively stressed human T lymphocytes. *Proceedings of the National Academy of Sciences* **99**, 3505 (2002).

19. Jaffrey, S. R., Erdjument-Bromage, H., Ferris, C. D., Tempst, P. & Snyder, S. H. Protein S-nitrosylation: a physiological signal for neuronal nitric oxide. *Nat. Cell Biol.* **3**, 193-197 (2001).

20. Lopez-Sanchez, L. M. *et al*. Alteration of S-nitrosothiol homeostasis and targets for protein S-nitrosation in human hepatocytes. *Proteomics* **8** (2008).

21. Greco, T. M. *et al*. Identification of S-nitrosylation motifs by site-specific mapping of the S-nitrosocysteine proteome in human vascular smooth muscle cells. *Proceedings of the National Academy of Sciences* **103**, 7420 (2006).

22. Murillo-Carretero, M., Torroglosa, A., Castro, C., Villalobo, A. & Estrada, C. S-Nitrosylation of the epidermal growth factor receptor: A regulatory mechanism of receptor tyrosine kinase activity. *Free Radical Biology and Medicine* **46**, 471-479 (2009).
23. Martínez-Ruiz, A. & Lamas, S. Detection and proteomic identification of S-nitrosylated proteins in endothelial cells. *Arch. Biochem. Biophys.* **423**, 192-199 (2004).
