# Supplementary material for: Comprehensive Identification and Modified-Site Mapping of S-Nitrosylated Targets in Prostate Epithelial Cells
Source: PLoS One. 2010 Feb 5;5(2):e9075. doi: 10.1371/journal.pone.0009075 (PMC2816712; doi:10.1371/journal.pone.0009075)
Supplement: Table S2 — Mapping SNO sites into high-resolution Protein Data Bank (PDB) structures. Entrez gene symbols, gene names, PDB codes (and chain letters, assumed to be A if not given), and the number of S-nitrosylated cysteine residues in those structures are given, respectively. (0.03 MB PDF) [file pone.0009075.s002.pdf]

Entrez	Name	PDB	Cys residue(s)
PDLIM1	PDZ and LIM domain 1 (elfin)	2pkt	74
TUBB2C	tubulin, beta 2C	1tub B	241
TUBA1C	tubulin, alpha 1c	3du7 A	375
TUBA1C	tubulin, alpha 1c	3du7 A	346
ACTN1	actinin, alpha 1	1sjj A	456
ACTN1	actinin, alpha 1	1sjj A	308
CS	citrate synthase	1cts	184
PDIA3	protein disulfide isomerase family A, member 3	3f8u A	220
VCP	valosin-containing protein	3cf1 A	85
PRDX5	peroxiredoxin 5	2vl2 A	48
LGALS1	lectin, galactoside-binding, soluble, 1 (galectin 1)	1gzw	60
LGALS1	lectin, galactoside-binding, soluble, 1 (galectin 1)	1gzw A	42
PCNA	proliferating cell nuclear antigen	1vyj	81
PCNA	proliferating cell nuclear antigen	1vyj	162
CLIC1	chloride intracellular channel 1	1rk4 A	170
ALDOA	aldolase A, fructose-bisphosphate	2ald	338
ALDOA	aldolase A, fructose-bisphosphate	2ald	72
ALDOA	aldolase A, fructose-bisphosphate	2ald	239
PKM2	pyruvate kinase, muscle	3gr4 A	37
PKM2	pyruvate kinase, muscle	3gr4 A	314
PKM2	pyruvate kinase, muscle	3gr4 A	346
PKM2	pyruvate kinase, muscle	3gr4 A	140
TPI1	triosephosphate isomerase 1	1wyi A	86
TPI1	triosephosphate isomerase 1	1wyi A	41
TPI1	triosephosphate isomerase 1	1wyi A	126
GPI	glucose phosphate isomerase	1iri A	404
MDH2	malate dehydrogenase 2, NAD (mitochondrial)	2dfd A	66
MDH2	malate dehydrogenase 2, NAD (mitochondrial)	2dfd A	262
HNRNPA1	heterogeneous nuclear ribonucleoprotein A1	1l3k	43
RPL5	ribosomal protein L5	2zkr N	61
RPL5	ribosomal protein L5	2zkr	75
RPS3	ribosomal protein S3	2zkq C	96
EGFR	epidermal growth factor receptor	1yy9 A	286
EGFR	epidermal growth factor receptor	1yy9	266
EGFR	epidermal growth factor receptor	1yy9	445
TUBA4A	tubulin, alpha 4a	1z2b A	346
TUBA4A	tubulin, alpha 4a	1z2b	375
TUBA4A	tubulin, alpha 4a	1z2b	294
YWHAQ	tyrosine 3-monooxygenase/tryptophan 5-monooxygenase activation protein, theta	2btp A	156
RNH1	ribonuclease/angiogenin inhibitor 1	1z7x W	37
RNH1	ribonuclease/angiogenin inhibitor 1	1z7x W	247
GAPDH	glyceraldehyde-3-phosphate dehydrogenase	1znq	248
GAPDH	glyceraldehyde-3-phosphate dehydrogenase	1znq	152
