# Supplementary material for: Comprehensive Identification and Modified-Site Mapping of S-Nitrosylated Targets in Prostate Epithelial Cells
Source: PLoS One. 2010 Feb 5;5(2):e9075. doi: 10.1371/journal.pone.0009075 (PMC2816712; doi:10.1371/journal.pone.0009075)
Supplement: Table S3 — Analysis of structural features (secondary structure and solvent accessibility) of the 42 SNO sites mapped into 24 Protein Data Bank structures. (0.05 MB PDF) [file pone.0009075.s003.pdf]

PDB structures with SNO sites:

>2pkt A
SMTTQQIDLQGPGPWGFRLVGGKDFEQPLAISRVTPGSKAALANLCIGDVITAIDGENTS
NMTHLEAQNRIKGCTDNLTLTVARSEHESDL
CEEEEEEEEECCCCCCEEEEEEHHHCEEEEEEEECCCCHHHHCCCCCCCEEEEECCEECC
CCCHHHHHHHHHC**C**CCEEEEEEEEECCCCCC
663776170526480215130046575601045238813028270466020300575616
7032530354077**1**68601020028385699

>1tub B
MREIVHIQAGQCGNQIGAKFWEVISDEHGIDPTGSYHGDSDLQLERINVYYNEAAGNKYV
PRAILVDLEPGTMDSVRSGPFGQIFRPDNFVFGQSGAGNNWAKGHYTEGAELVDSVLDVV
RKESESCDCLQGFQLTHSLGGGTGSGMGTLLISKIREEYPDRIMNTFSVVPSPKVSDTVV
EPYNATLSVHQLVENTDETYCIDNEALYDICFRTLKLTTPTYGDLNHLVSATMSGVTTCL
RFPGQLNADLRKLAVNMVPFPRLHFFMPGFAPLTSRGSQQYRALTVPELTQQMFDAKNMM
AACDPRHGRYLTVAAVFRGRMSMKEVDEQMLNVQNKNSSYFVEWIPNNVKTAVCDIPPRG
LKMSATFIGNSTAIQELFKRISEQFTAMFRRKAFLHWYTGEGMDEMEFTEAESNMNDLVS
EYQQYQD
CCEEEEEECHHHHHHHHHHHHHHCCCCCCCCCCCCCCCCCCCCCCCCCCCCCCCCCCCCC
CCCEECCCCHHHHHHHCCCCCCCCCCCCCCCCCCCCCCCCCHHHHCCHHHHHHHHHHHHH
HHHHHCCCCEEEEEEECCCCCCCCCCCHHHHHHHHHHHCCCCCEEECCCCCCCCCCCCCC
CHHHHHHHHHHHCCCEEEECCCCHHHHHHHCCCCCCCCCCCHHHHHHHHHHHHHHHHC**E**C
CECCCCCCCHHHHHHHCCCCCCCCCCCCEECCCCCCCHHHCCCCHHHHHHHHHHCHHHCC
CCCCCCCCCEEEEEEECCCCCCCCHHHHHHHHHHCCCHHHECCCCCCCCEEECCCCCCCC
CCCCCEEEEEEHHHHHHHHCHHHHHHHCHHHHHCCHHHHCCCCCCCCCHHHHHHHHHHHH
HHHCCCC
210000000052003001301330230000023101696544801286755141010410
100000003330075135933241083011317727115000101222027005201500
550170733000000000101000000001001204751582100000001145854700
1000000000003200100000022002300562272772453200700000000000**0**0
002022000010000000015400000001010519746876822032005100336000
000503622000000000082000101120250268164200700200000000331179
171000000000001200220264034104694406604826067420350142034004
2035128

>3du7 A
RECISIHVGQAGVQIGNACWELYCLEHGIQPDGQMPXXXXXXXXXXSFNTFFSETGAGKH
VPRAVFVDLEPTVIDEVRTGTYRQLFHPEQLISGKEDAANNYARGHYTIGKEIIDLVLDR
VRKLADQCTGLQGFLVFHSFGGGTGSGFTSLLMERLSVDYGKKSKLEFSIYPAPQVSTAV
VEPYNSILTTHTTLEHSDCAFMVDNEAIYDICRRNLDIERPTYTNLNRLMSQIVSSITAS
LRFDGALNVDLTEFQTNLVPYPRIHFPLATYAPVISAEKAYHEQLSVAEITNACFEPANQ
MVKCDPRHGKYMACCLLYRGDVVPKDVNAAIATIKTKRTIQFVDW**C**PTGFKVGINYQPPT
VVPGGDLAKVQRAV**C**MLSNTTAVAEAWARLDHKFDLMYAKRAFVHWYVGEGMEEGEFSEA
REDMAALEKDYEEVGADS
CEEEEEEECHHHHHHHHHHHHHHHHHHCCCCCCCCCXXXXXXXXXXCCCCCECCCCCCCC
CECEEEECCCCCCCCCCCCCCCCCCCHHHEECCCCCCCCCHHHHHHCCCCCCHHHHHHH
HHHHHCCCCCEEEEEEEEEECCCHHHHHHHHHHHHHHHHHHCCCCEEEEEEEECCCCCCCCC
CHHHHHHHHHHHHHCCCCCEEEECCHHHHHHHHHHHCCCCCCCCHHHHHHHHHHHHHHHHCCH
HHHHCCCCCCHHHHHHHCCCCCCCCCCECCCCCCCECCCCCCCCCCHHHHHHHHHCCHHHC
CCCCCCCCCCECCCCCCECEECCCHHHHHHHHHCHHHHCCCCCECCC**C**CCCCEEEECCCCCC
CCCCCCECCCCEEC**C**CCECECCCCHHHHHHHHHHHHHHHHCCCCCCHHHHHHCCCHHHHHHH
HHHHHHHHHHHHHHHCCCC
700000000220030020001000302503230229999999999981310014299533
000000000034004204748175005630004171100200000001203620550063
045118519501000000100110000000000023004306720000000000002315100
010000000011006002000000110012004720815605340002000000000000
100301003305303720145820000000001121454297143614400420244310
0041302320000000000060514502610150095274110431**1**30103000042504
108933007151000**0**00000100140023006104200534000210231005551045O
172042026306520669

>1sjj A
LDPAWEKQQRKTFTAWCNSHLRKAGTQIENIEEDFRDGLKLMLLLEVISGERLAKPERGK
MRVHKISNVNKALDFIASKGVKLVSIGAEEIVDGNVKMTLGMIWTIILRFAIQDISVEET
SAKEGLLLWYQRKTAPYKNVNIQNFHISWKDGLGFCALIHRHRPELIDYGKLRKDDPLTN
LNTAFDVAEKYLDIPKMLDAEDIVGTARPDEKAIMTYVSSFYHAFSGAQKAETAANRICK
VLAVNQENEQLMEDYEKLASDLLEWIRRTIPWLENRAPENTMQAMQQKLEDFRDYRRLHK
PPKVQEK**C**QLEINFNTLQTKLRLSNRPAFMPSEGKMVSDINNAWGGLEQAEKGYEEWLLN
EIRRLERLDHLAEKFRQKASIHESWTDGKEAMLQQKDYETATLSEIKALLKKHEAFESDL
AAHQDRVEQIAAIAQELNELDYYDSPSVNARCQKI**C**DQWDNLGALTQKRREALERTEKLL
ETIDQLYLEYAKRAAPFNNWMEGAMEDLQDTFIVHTIEEIQGLTTAHEQFKATLPDADKE
RQAILGIHNEVSKIVQTYHVNMAGTNPYTTITPQEINGKWEHVRQLVPRRDQALMEEHAR
QQQNERLRKQFGAQANVIGPWIQTKMEEIGRISIEMHGTLEDQLNHLRQYEKSIVNYKPK
IDQLEGDHQQIQEALIFDNKHTNYTMEHIRVGWEQLLTTIARTINEVENQILTRDAKGIS
QEQMNEFRASFNHFDRKKTGMMDCEDFRACLISMGYNMGEAEFARIMSIVDPNRMGVVTF
QAFIDFMSRETADTDTADQVMASFKILAGDKNYITVDELRRELPPDQAEYCIARMAPYNG
RDAVPGALDYMSFSTALYGESDL
CCCCCHHHHHHHHHHHHHHHHHHHCCCCCCCCCCCCCCHHHHHHHHHCCCCCCCCCCCCC
CHHHHHHHHHHHHHHHHHCCCCCCCCCCHHHHCCCHHHHHHHHHHHHCCCCCCCCCCCCC
CCCCCCHHHHHHHHCCCCCCCCCCCCHHHCCCHHHHHHHHCCCCCCCCCHHHHCCCCHHH
CCHHHHHHHHHHCCCCCCHHHHCCCCCCCCHHHHHHHHHHCCCCCCCCCCCCCCCCCCCC
CCCHHHHHHHHHHHHHHHHHHHHHHHHHHHHHCCCCCCCCCHHHHHHHHHHHHHHHHCCC
HHHHHHH**H**HHHHCCCHHHHHCCCCCCCCCCCCCCCCHHHHHHHHHHHHHHHCCCHHHHHH
HHEEHHHHHHHHHHHHHHHHHHHHHHHHHHHHHHHHCCCCCCCHHHHHHHHHHHHHHHHHHH
CCHHHHHHHHHHHHHHHHHCCCCCHHHHHHHHHHH**H**HHHHHHHHHHHHHHHHHHHHHHHH
HHHHHHHHHHHHHCCCHHHHHHHHHHHHHCCCCCCCCHHHHHHHHHHHHHHCCHHHHHHH
HHHHHHHHHHHHHHHHCCCCCCCCCCCCCCCCHHHHHHHHHHHHHHHHHHHHHHHHHHHH
HHHHHHHHHHHHHHHHHHHHHHHHHHHHCCCCCCCCCCCCHHHHHHHHHHHHHHHCCHHH
HHHHHHHHHHHHCCCCCCCCCCCCCHHHHHHHHHHHHHHHHHHHHHHHHHHHHCCCCCCC
HHHHHHHHHHHCCCCCCCCCEEECCCHHHHHHHHCCCCCCHHHHHHHHHHCCCCCCEEEC
CHHHHCHHHHCCCCCCCHHHHHHHHHHHCCCCEEEHHHHHHHCCHHHHHHHHHHCEECCC
CCCCCCEEECHHHHHHHCCCCCC
977474952273015202502753366436065303203300500220030905372859
856411200420162033071618702053005235521130042016054253702662
618801241033101111404060034203202000000120135443027135495447
136004203540905351607200236915472024003203613523847765461201
050022002003300110040014109612730464455943810252034013013550
0513301**0**3020012101100301222113066713185046214404700730341014
304412404310410451043027107513660665312633125020215205122212
64047005503410520263817216502430670**3**731850433055146104402700
220152032016201501120230226053422146274046324505713521650173
072024216304611755704358612106140840341045045105511520551543
143024304600430350022053134218407619643132025204423620571353
047014222710433034273093503601520250221123211505252246436344
376216123103511641223041520210253558485664147046512678742032
210340174655963427521461442078582021750131000420550473035486
97529401205210543554968

>1cts
ASSTNLKDILADLIPKEQARIKTFRQQHGNTVVGQITVDMMYGGMRGMKGLVYETSVLDP
DEGIRFRGYSIPECQKMLPKAKGGEEPLPEGLFWLLVTGQIPTEEQVSWLSKEWAKRAAL
PSHVVTMLDNFPTNLHPMSQLSAAITALNSESNFARAYAEGIHRTKYWELIYEDCMDLIA
KLP**C**VAAKIYRNLYREGSSIGAIDSKLDWSHNFTNMLGYTDAQFTELMRLYLTIHSDHEG
GNVSAHTSHLVGSALSDPYLSFAAAMNGLAGPLHGLANQEVLVWLTQLQKEVGKDVSDEK
LRDYIWNTLNSGRVVPGYGHAVLRKTDPRYTCQREFALKHLPHDPMFKLVAQLYKIVPNV
LLEQGKAKNPWPNVDAHSGVLLQYYGMTEMNYYTVLFGVSRALGVLAQLIWSRALGFPLE
RPKSMSTDGLIKLVDSK
CCCCCCHHHHHHHHHHHHHHHHHHHHHHHHCCCCCCHHHHHHCCCCCCCCCCCCCCEEEC
CCEEECCCCEHHHHHHHCCECCCCCCECHHHHHHHHHCCCCCHHHHHHHHHHHHHHHCCC
CHHHHHHHHCCCCCCHHHHHHHHHHHHHHHHCHHHHHHHHCCCHHHHHHHHHHHHHHHHH
HHH**H**HHHHHHHHHCCCCCCCCCCCCCCHHHHHHHHHHCCCHHHHHHHHHHHHHHCCCCCC
CCHHHHHHHHHHHCCCCHHHHHHHHHHHHCCCCCCCHHHHHHHHHHHHHHHCCCCCCCCH
HHHHHHHHHHCCCCCCCECCCCCCCCHHHHHHHHHHHHHHCCCHHHHHHHHHHHHHHHHH
HHHCCCCCCCCECCHHHHHHHHHCCCCCCHHHHHHHHHHHHHHHHHHHHHHHHHCCCCCC
CCCCCCHHHHHHHHCCC
954440261037105734381755787449676696538245295892882614005122
530020721004302750140993400000000000000452056310310152018115 2
184025305814692300400020042036302037047871577400320020001000
000**0**000000211239638187336821002000400409453000000000000000240
624002200330142200020002006103259103200500510340276036915574
036103601649650220329406400000300250044203814005001102700050
046293490020202000000004414061240000000000000000000000162762
75774547306735979

>3f8u A
SDVLELTDDNFESRISDTGSAGLMLVEFFAPWCGHAKRLAPEYEAAATRLKGIVPLAKVD
CTANTNTCNKYGVSGYPTLKIFRDGEEAGAYDGPRTADGIVSHLKKQAGPASVPLRTEEE
FKKFISDKDASIVGFFDDSFSEAHSEFLKAASNLRDNYRFAHTNVESLVNEYDDNGEGII
LFRPSHLTNKFEDKTVAYTEQKMTSGKIKKFIQENIFGI**C**PHMTEDNKDLIQGKDLLIAY
YDVDYEKNAKGSNYWRNRVMMVAKKFLDAGHKLNFAVASRKTFSHELSDFGLESTAGEIP
VVAIRTAKGEKFVMQEEFSRDGKALERFLQDYFDGNLKRYLKSEPIPESNDGPVKVVVAE
NFDEIVNNENKDVLIEFYAPWCGHCKNLEPKYKELGEKLSKDPNIVIAKMDATANDVPSP
YEVRGFPTIYFSPANKKLNPKKYEGGRELSDFISYLQREATNPPVIQEE
CCCEEECCCCHHHHCCCCCCCCEEEEEEECCCCHHHHHHHHHHHHHHHHCCCCCCEEEEE
CCCCHHHHHHCCCCEECEEEEEECCEEEEECCCCCCHHHHHHHHHHCCCCEEEEECCHHH
HHHHCCCCCEEEEEEECCCCCHHHHHHHHHHHHCCCCCEEEEECCHHHHHHHCCCCEEEE
EECCHHHCECCECCEEECCCCCCHHHHHHHHHHHHCCCC**C**CEECCCCHHHHCCCCEEEEE
ECCCCCCCHHHHHHHHHHHHHHHHHHHHCCCCCEEEEEECCCCHHHHHHHCCCCCCCCCC
EEEECCCCCEEECCCCCCCCCHHHHHHHHHHHHCCCCCCCCCCCCCCCCCCCCCEEECCC
CHHHHHCCCCCEEEEEEEECCCEHHHHHHHHHHHHHHHCCCCCCEEEEEEECCCCCCCCC
CCCCCCCEEEEECCCCCCCEECCCCCCCHHHHHHHHHHHCCCCCCCCCC
930360339105730561592000000010410110670353023005507840200203
045045002617070002010048284354093533071006205221350032074363
067126464200000064880601410450066028412001061650066229703000
001153141911843140849403233035104720232**0**010347118202743000000
030417933810320022003003502765380400000084055007405175684320
200031495321008470266060035004312697295232237529728531330006
007710234720000002030004024055305400410660520100201122042294
0716410000000065087132075444164014006631448231479

>3cf1 A
NRPNRLIVDEAINEDNSVVSLSQPKMDELQLFRGDTVLLKGKKRREAVCIVLSDDTCSDE
KIRMNRVVRNNLRVRLGDVISIQPCPDVKYGKRIHVLPIDDTVEGITGNLFEVYLKPYFL
EAYRPIRKGDIFLVRGGMRAVEFKVVETDPSPYCIVAPDTVIHCEGEPIKREDEEESLNE
VGYDDIGGCRKQLAQIKEMVELPLRHPALFKAIGVKPPRGILLYGPPGTGKTLIARAVAN
ETGAFFFLINGPEIMSKLAGESESNLRKAFEEAEKNAPAIIFIDELDAIAPKREKTHGEV
ERRIVSQLLTLMDGLKQRAHVIVMAATNRPNSIDPALRRFGRFDREVDIGIPDATGRLEI
LQIHTKNMKLADDVDLEQVANETHGHVGADLAALCSEAALQAIRKKMDLIDLEXETIDAE
VMNSLAVTMDDFRWALSQSNPSALRETVVEVPQVTWEDIGGLEDVKRELQELVQYPVEHP
DKFLKFGMTPSKGVLFYGPPGCGKTLLAKAIANECQANFISIKGPELLTMWFGESEANVR
EIFDKARQAAPCVLFFDELDSIAKARXXXXGDGGGAADRVINQILTEMDGMSTKKNVFII
GATNRPDIIDPAILRPGRLDQLIYIPLPDEKSRVAILKANLRKSPVAKDVDLEFLAKMTN
GFSGADLTEICQRACKLAIRESIESEIXXXXXXXXXXXXXXXXXXXXVPEIRRDHFEEAM
RFARRSVSDNDIRKYEMFAQTLQ
CCCCECCEECCCCCCCCCCCCECHHHHHHHCCCCCCEECECCCCCCCCECCECCECCCCCCCC
CEECCHHHHHCCCCCCCCCCCECCE**C**CCCCCCCEEEEEECHHHCCCCCCCHHHHCHHHHHCC
CCCCEEECCCCCEEEECCEEEEEEEEEECCCCCEEECCCCCEEECCCCCCCCCCCCCCCC
CCHHHCCCCHHHHHHHHHHHCCCCCCCCCCCCCCCCCCCCEEEECCCCCCCHHHHHHHHHH
HHCCEEEEEEECCHHHHCCCCHHHHHHHHHHHHHHHHCCCEEEEEEECHHHCCCCCCCCCCCHH
HHHHHHHHHHHHCCCCCCCCCCCEEEECCCCCCCCCHHHCCCCCCCEEECCCCCCHHHHHHH
HHHHCCCCCCCCCCCHHHHHCCCCCCCCCHHHHHHHHHHHHHHHHHHHHHHCCCXCCCCHH
HHHHCCCCHHHHHHHHHHHCCCCCCCCCCCCCCCCCCCCCCCCHHHHHHHHHHHCHHHHCH
HHHHHHCCCCCCCEEEECCCCCCHHHHHHHHHHHHCCCEEEECCHHHHHHHHCCCCCHHHH
HHHHHCCCCCCEEEEECCCCHHHHHCXXXXCCCCCCCCHHHHHHHHHHHHCCCCCCCCCEEE
EEECCHHHCCHHHCCCCCCCEEEECCCCCHHHHHHHHHHHHCCCCCECCCCCHHHHCCCCC
CCCHHHHHHHHHHHHCCHHHHHHHHHCXXXXXXXXXXXXXXXXXXXXCCCECHHHHHHHH
CCCCCCCCHHHHHHHHHHHHHHC
874410002225382210000055007519076811000316591300010230950542
2010100001001021232010340**0**750750530201004301831554144000045006
634200141100003102210001044062522010149141335763282494122073
200421222463133023002000123200501905101000000153130241030002
015131240404402747544006205400440375220000024022102144717423
421010110110450667210000000443630161032320020201041022600230
061503206229515264005406010232024005300420343506875579951204
122613033700420184487242753322307131741212560042011011214623
600550516401000000043130260030002438020030604302702666416405
310320141430000033000024439999955235001000110300430374231000
000221431151034560011211021054810131061404935429414044106516
000230024005303721573456769999999999999999999999750335004502
86384603472264047218525

>2vl2 A
SAPIKVGDAIPAVEVFEGEPGNKVNLAELFKGKKGVLFGVPGAFTPGCSKTHLPGFVEQA
EALKAKGVQVVACLSVNDAFVTGEWGRAHKAEGKVRLLADPTGAFGKETDLLLDDSLVSI
FGNRRLKRFSMVVQDGIVKALNVEPDGTGLTXSLAPNIISQL
CCCCCCCCECCCCEEECCCCCCEEEHHHHHCCCEEEEEEEECCCCCHH**H**HHCHHHHHHHCH
HHHHCCCEEEEEEEEECCCHHHHHHHHHHCCCCCCCEEEECCCCHHHHHHCCECCHHHHHH
HCCCCECCEEEEEEECCEEEEEEEECCCCCCCCCCCHHHHHHCC
954055645015040222547471102710773200000000005152**0**173002300730
530574406000000014062005016528099303000037020065040003720263
27441020000000461205120204001245902053027333

>1gzw A
ACGLVASNLNLKPGEXLRVRGEVAPDAKSFVLNLGKDSNNL**C**LHFNPRFNAHGDANTIV**C**
NSKDGGAWGTEQREAVFPFQPGSVAEVCITFDQANLTVKLPDGYEFKFPNRLNLEAINYM
AADGDFKIKCVAFD
CCCCEEEEEEECCCCEEEEEEEECCCCCECCEEEEEECCEE**E**EEEEEEEEECCEEEEEE**E**
EEEECCEECCCEECCCCCCCCCCEEEEEEEECCCEEEEECCCCCEEEEECCCCCCCEEEE
EECCCEEEEEEEEC
57100034150547190404040268061000100535520**0**00000000617916510 0**0**
002674544742417212043545020102045630004055545050204170810310
00273050210207

>1w60 A
MFEARLVQGSILKKVLEALKDLINEACWDISSSGVNLQSMDSSHVSLVQLTLRSEGFDTY
RCDRNLAMGVNLTSMSKILK**C**AGNEDIITLRAEDNADTLALVFEAPNQEKVSDYEMKLMD
LDVEQLGIPEQEYSCVVKMPSGEFARICRDLSHIGDAVVIS**C**AKDGVKFSASGELGNGNI
KLSQTSNVDKEEEAVTIEMNEPVQLTFALRYLNFFTKATPLSSTVTLSMSADVPLVVEYK
IADMGHLKYYLAPKI
CEEEEECCHHHHHHHHCCCCCCCCEEEEEECCCEEEEEEECCCCCEEEEEEEECCCCCEE
EECCCEEEEEEHHHHHHHCC**C**CCCCCEEEEECCCCCEEEEEEECCCCCCEEEEEEEECCC
CCCCCCCCCCCCCCEEEEEEHHHHHHHHHHHHCCCCEEEEE**E**CCCCEEEEECCCCEEEEE
EECCCCCCCCCCCCCCECCCCCCEEEEEHHHHHHHHHHCCCCCEEEEEEECCEEEEEEEC
CCCCEEEEEEECCCC
202020460110230030033005400030276002021209553000101041720642
51578150102033006107**4**036701000305292720000010576855163708149
29465393694910020401064026006303720920201**0**277003010759837252
516689596775440416275607030107102201402511820201014913000106
079001020103349

>1rk4 A
GNCPFSQRLFMVLWLKGVTFNVTTVDTKRRTETVQKLCPGGQLPFLLYGTEVHTDTNKIE
EFLEAVLCPPRYPKLAALNPESNTAGLDIFAKFSAYIKNSNPALNDNLEKGLLKALKVLD
NYLTSPLPEEVDETSAEDEGVSQRKFLDGNELTLADCNLLPKLHIVQVV**C**KKYRGFTIPE
AFRGVHRYLSNAYAREEFASTCPDDEEIELAYE
CCCCCCHHHHHHHHHHCCCCCCHHHHHHHHHHHHHHHCCCCCCCCCCCCCCCCCCCCHHH
HHHHHHCCCCCCCCCCCCHHHHCCCCCCHHHHHHHHHHCCCHHHHHHHHHHHHHHHHHHH
HHHHCCCHHHCCCCCCCCCCCCCCCCCCCCCCCHHHHHHHHHHHHHHHH**H**HHHHCCCCCC
CCHHHHHHHHHHCCHHHHCCCCCHHHHHHHHCC
920201010000001070200547404650362047007823214217994636270500
300130034751460318165046103200130030141947641673362027003500
4102540660794827969630404001147001000100010100200**0**7633806026
614001300620372620430215564016419

```
>2ald A
PYQYPALTPEQKKELSDIAHRIVAPGKGILAADESTGSIAKRLQSIGTENTEENRRFYRQ
LLLTADDRVNPCIGGVILFHETLYQKADDGRPFPQVIKSKGGVVGIKVDKGVVPLAGTNG
ETTTQGLDGLSERCAQYKKDGADFAKWRCVLKIGEHTPSALAIMENANVLARYASICQQN
GIVPIVEPEILPDGDHDLKRCQYVTEKVLAAVYKALSDHHIYLEGTLLKPNMVTPGHACT
QKFSHEEIAMATVTALRRTVPPAVTGITFLSGGQSEEEASINLNAINKCPLLKPWALTFS
YGRALQASALKAWGGKKENLKAAQEEYVKRALANSLACQGKYTPSGQAGAAASESLFVSN
HAY
CCCCCCCCHHHHHHHHHHHHHHCCCCCEEEEECCCHHHHHHHHHHCCCCCHHHHHHHHHH
HHHCCCHHHCCCEEEEEECHHHHCCECCCCCEHHHHHHHCCCEEEEEECCCCEEECCCCC
CEEECCCCHHHHHHHHHHHCCCCEEEEEEEECCCCCCCHHHHHHHHHHHHHHHHHHHHHC
CCEEEEEEEECCCCCCHHHHHHHHHHHHHHHHHHHHHHCCCHHHCEECCCCCCCCCCCCC
CCCHHHHHHHHHHHHHHCCCCCCCEEEECCCCCCHHHHHHHHHHHHHCCCCCCCCEEEEE
ECHHHHHHHHHHHCCCHHHHHHHHHHHHHHHHHHHHHHCCCCCCCCCCCCCCCCCCCCCC
CCC
978873046734640140055002802000000033730152067191733652111000
000304740250000000241003050766330050046161000010153437189277
011050285046202402731010000200020387012740163003000400210052
100000000001248040640060016003200600652701230000000001105519
692434400300010057102630100000032110130020000026072724020000
023000110062032466328401420010040014014260547866640195602353
967

>3gr4 A
IQTQQLHAAMADTFLEHMCRLDIDSPPITARNTGIICTIGPASRSVETLKEMIKSGMNVA
RLNFSHGTHEYHAETIKNVRTATESFASDPILYRPVAVALDTKGPEIRTGLIKGSGTAEV
ELKKGATLKITLDNAYMEKCDENILWLDYKNICKVVEVGSKIYVDDGLISLQVKQKGADF
LVTEVENGGSLGSKKGVNLPGAAVDLPAVSEKDIQDLKFGVEQDVDMVFASFIRKASDVH
EVRKVLGEKGKNIKIISKIENHEGVRRFDEILEASDGIMVARGDLGIEIPAEKVFLAQKM
MIGRCNRAGKPVICATQMLESMIKKPRPTRAEGSDVANAVLDGADCIMLSGETAKGDYPL
EAVRMQHLIAREAEAAIYHLQLFEELRRLAPITSDPTEATAVGAVEASFKCCSGAIIVLT
KSGRSAHQVARYRPRAPIIAVTRNPQTARQAHLYRGIFPVLCKDPVQEAWAEDVDLRVNF
AMNVGKARGFFKKGDVVIVLTGWRPGSGFTNTMRVVPVP
CCCCHHHHCCCCHHHHHHCCCCCCCCCCCCCCCEEEEECCCCCCCHHHHHHHHHHCCCEE
EEECCCCCHHHHHHHHHHHHHHHHCCCCCCCCCCCCEEEEECCCCCCEECCECCECCCCE
EECCCCEEEEECCHHHCCCECCCEEEECCCCHHHHCCCCCEEEECCCCEEEEEEEECCCE
EEEEEEECEEECCCCEEECCCCCCCCCCCCHHHHHHHHHHHHCCCCEEEECCCCCHHHHH
HHHHHHCCCCCCEEEEEEECCHHHHHCHHHHHHHCCEEEEEHHHHHHHCCHHHHHHHHHH
HHHHHHHHCCCEEEECCCCHHHHCCCCCCHHHHHHHHHHHHHCCCEEEECHHHHCCCCHH
HHHHHHHHHHHHHHHCCHHHHHHHHHHHHCCCCCCHHHHHHHHHHHHHHHCCCCCEEEEC
CCCHHHHHHHCCCCCCEEEEECCHHHHHHHHHHCCCEEEEECCCCCCCHHHHHHHHHHHH
HHHHHHCCCCCCCCEEEEEEEECCCCCCCEEEEEEEECC
634201401606140100020405041250110000000072014282043005110100
002015563830240061034004422932240300000000101101001065469260
403793601001466436202261000305401320654220203311000403232843
030203224500063200029273534312730450060026250000000101315004
403730365065020000001330063064007002000000030112042110000000
000200232200000200022014244001001000000031000000021001707311
300410230020000001042202101331552633020000000000200502000000
240200120020001000000024410020000100020000646639336600321040
004003416104564100000264465721000001506
```

>1wyi A
APSRKFFVGGNWKMNGRKQSLGELIGTLNAAKVPADTEVV**C**APPTAYIDFARQKLDPKIA
VAAQNCYKVTNGAFTGEISPGMIKD**C**GATWVVLGHSERRHVFGESDELIGQKVAHALAEG
LGVIA**C**IGEKLDEREAGITEKVVFEQTKVIADNVKDWSKVVLAYEPVWAIGTGKTATPQQ
AQEVHEKLRGWLKSNVSDAVAQSTRIIYGGSVTGATCKELASQPDVDGFLVGGASLKPEF
VDIINAKQ
CCCCCEEEEEECCECCCHHHHHHHHHHHHHCCCCCCCEEEE**E**ECCHHHHHHHHHHCCCCCE
EEECCCCCCCCECCCCCCCHHHHHH**C**CCCEEEECCHHHHCCCCCCHHHHHHHHHHHHHCC
CCEEE**E**ECCCHHHHHHCCHHHHHHHHHHHHHHCCCCCCCEEEEECCHHHCCCCCCCCHHH
HHHHHHHHHHHHHHCCHHHHHHCCEEECCCCCCCCHHHHHHCCCCCEEEECHHHHCCHH
HHHHCCCC
95914000000010212284037004303718208502000**0**000000001016603740 1
00000003343241010000000043**0**40500000000000342715371003003101725
00000**0**00033621767314500240052016209507500000001024939482316 2
004005401510585337510350100011803263055007151000000270035620
15002046

>1iri A
MAALTRDPQFQKLQQWYREHRSELNLRRLFDANKDRFNHFSLTLNTNHGHILVDYSKNLV
TEDVMRMLVDLAKSRGVEAARERMFNGEKINYTEGRAVLHVALRNRSNTPILVDGKDVMP
EVNKVLDKMKSFCQRVRSGDWKGYTGKTITDVINIGIGGSDLGPLMVTEALKPYSSGGPR
VWYVSNIDGTHIAKTLAQLNPESSLFIIASKTFTTQETITNAETAKEWFLQAAKDPSAVA
KHFVALSTNTTKVKEFGIDPQNMFEFWDWVGGRYSLWSAIGLSIALHVGFDNFEQLLSGA
HWMDQHFRTTPLEKNAPVLLALLGIWYINCFGCETHAMLPYDQYLHRFAAYFQQGDMESN
GKYITKSGTRVDHQTGPIVWGEPGTNGQHAFYQLIHQGTKMIP**C**DFLIPVQTQHPIRKGL
HHKILLANFLAQTEALMRGKSTEEARKELQAAGKSPEDLERLLPHKVFEGNRPTNSIVFT
KLTPFMLGALVAMYEHKIFVQGIIWDINSFDQWGVELGKQLAKKIEPELDGSAQVTSHDA
STNGLINFIKQQREARV
CCHHHHCHHHHHHHHHHHHHHHHHCCHHHHHHHCCCHHHHCEEEEECCCCEEEEEECCCCCC
CHHHHHHHHHHHHHHHCHHHHHHHHHHCCCCCECCCCECCCHHHHCCCCCCCCCEECCEECHH
HHHHHHHHHHHHHHHHHHCCCECCCCCCECCEEEEEECCHHHCHHHHHHHHHHCHHHCCCCCE
EEEECCCCHHHHHHHHCCCCHHHEEEEEEECCCCCCHHHHHHHHHHHHHHHHHHHHCCHHHHH
HCEEEEEECCHHHHHHHHCCCHHHEEECCCCCCHHHCCCCHHHHHHHHHHHCHHHHHHHHHHHH
HHHHHHHHHCCHHHCHHHHHHHHHHHHHHHHHCCCCCEEEEEEECCHHHCCHHHHHHHHHHHHHH
CCCECCCCCECCCCCCCEEECCCCCHHHHCCHHHHHHHCCCCCC**E**EEEEEEECCCCHHHHH
HHHHHHHHHHHHHHHHHHHCECHHHHHHHHHHHCCCCHHHHHHHHHHHHCECCCCCCEEEEEEE
ECCHHHHHHHHHHHHHHHHHHHHHHHCCCCCCCCHHHHHHHHHHHHHHHHHHHCCCCCCCCCCH
HHHHHHHHHHHHHCCCCC
702017052054035117525950202720873740164010515074110000000000
155005000300541304400330050441021252000000000364650518972005
402600740450064036061402454503000000001001002000200322198306
111000000000130076141200000000014051200100040024202731556700 2
500000033354057060257000301500220000000000000000000720240020 0
120030037140450000000000000011150100000000100210000000000000
000005733404130000001100010010000000000010200**0**000000212262392 2
002000000000000003003452026306869437750671020100402100000000
200000000000000000000100200000210120042105600520669570861 12
00110010006037295

>2dfd A
NAKVAVLGASGGIGQPLSLLLKNSPLVSRLTLYDIAHTPGVAADLSHIETKAAVKGYLGP
EQLPD**C**LKGCDVVVIPAGVPRKPGMTRDDLFNTNATIVATLTAACAQHCPEAMICVIANP
VNSTIPITAEVFKKHGVYNPNKIFGVTTLDIVRANTFVAELKGLDPARVNVPVIGGHAGK
TIIPLISQCTPKVDFPQDQLTALTGRIQEAGTEVVKAKAGAGSATLSMAYAGARFVFSLV
DAMNGKEGVVECSFVKSQETE**C**TYFSTPLLLGKKGIEKNLGIGKVSSFEEKMISDAIPEL
KASIKKGEDFVKTL
CEEEEEECCCCCCHHHHHHHHHCCCCEEEEEEEEECCCHHHHHHHHHCCCCCCEEEEEEECC
CCHHH**H**HCCCCEEEECCCCCCCCCCCHHHHHHHHHHHHHHHHHHHHHHHHCCCCEEEECCCC
HHHHHHHHHHHHHHCCCCCCCCEEECCHHHHHHHHHHHHHHHHHCCCHHHCECCEEECCCHH
HEEECHHHCECCCCCCHHHHHHHHHHHHHHHHHHHHHHHHHHCCCCCCHHHHHHHHHHHHHHH
HHHCCCCCCEEEEEEEECCCCC**C**CEEEEEEEEEECCEEEEECCCCCCCHHHHHHHHHHHHHH
HHHHHHHHHHHHHCC
703000010146102000000021720320000173504000200200013041531316
74025**0**056030000014140436030200030004200600100063037000000020
00000000020046383233310000000000000000000544933047040100000126
0000005305451814583155003500300250053062522201000010000001000
2025469704000002074270**0**40000102008500562411772392055106601530
35005402410676

>1l3k A
KEPEQLRKLFIGGLSFETTDESLRSHFEQWGTLTD**C**VVMRDPNTKRSRGFGFVTYATVEE
VDAAMNARPHKVDGRVVEPKRAVSXXXXXXXXXXXXTVKKIFVGGIKEDTEEHHLRDYFEQ
YGKIEVIEIMTDRGSGKKRGFAFVTFDDHDSVDKIVIQKYHTVNGHNCEVRKAL
CCCHHHHEEEEECCCCCCCHHHHHHHHHHHHCCEEEE**E**EEEECCCCCCEEEEEEEEEECCHHH
HHHHHHCCCCEECCEECEEEECCCXXXXXXXXXXXXCCCEEEEEECCCCCCCHHHHHHHHHCC
CCCEEEEEEEEECCCCCCEEEEEEEEEECCHHHHHHHHHCCCCEECCEECEEEEECC
945543030102502670346203610462260560**0**104337867705130100023281
0330062270604865050212899999999999996153000040657034520352058
126054142254996662611010203200000102359606039170515409

>2zkr n
AYFKRYQVKFRRRREGKTDYYARKRLVIQDKNKYNTPKYRMIVRVTNRDIICQIAYARIE
GDMIV**C**ARYAHELPKYGVKVGLTNYAAAY**C**TGLLLARRLLNRFGMDKIYEGQVEVTGDEY
NVESIDGQPGAFTCYLDAGLARTTTGNKVFGALKGAVDGGLSIPHSTKRFPGYDSESKEF
XAEVHRKHIMGQNVADYMRYLMEEDEDAYKKQFSQYIKNSVTPDMMEEMYKKAHAAI
CCCCCCCCCCHHHHCCCCCHHHHHHHHHCCCCCCCCCCCEEEEEEEEECCEEEEEEEEECCCC
CCEEE**E**EEEHHHHHHHCCCCCCCCCHHHHHH**H**HHHHHHHHHHHCCCCCCCCCCCCCCCCCCC
CCCCCCCCCCCCEEEEECCCCCCCCCCHHHHHHHHHHHCCCECCCCHHHCCCCCCCCCCC
XCCCCCCCCCCCHHHHHHHCCCCCCCCCCCCCCEEEEEECCEEECCCCCHHHHHHHHHC
994815535765444451538224830211003323731000034577102020011378
33251**0**2040700451425113302000000**0**0000000420257878768597947233638
864550362506101102484864522201100200251503065468522528144495
922000530005401431874975772029851318398310340100003602631

>2zkq c
AVQISKKRKFVADGIFKAELNEFLTRELAEDGYSGVEVRVTPTRTEIIILATRTQNVLGE
KGRRIRELTAVVQKRFGFPEGSVELYAEKVATRGL**C**AIAQAESLRYKLLGGLAVRRACYG
VLRFIMESGAKGCEVVVSGKLRGQRAKSMKFVDGLMIHSGDPVNYYVDTAVRHVLLRQGV
LGIKVKIMLPWD
CECCCCCCCHHHHHHHHHHHHHHHHHHHCCCCCEEEEEECEEECCEECCEEEECCHHHHHCH
HHHHHHHHHHHHHHHHCCCCCCCCCCEEEEECCCHHH**C**HHHHHHHHHHHHHHHCCCHHHHHHHH
HHHHHHCCCCEEEEEEECCCHHHCCCCCECCEEECCHHHHCCCCCCEEEEEECCCCCCCC
CCEEEEEEECCCC
320001593063003022102220375026002321501135760401010053550217
928326601240475518682506151462646230**0**021001402430488450380023
006501747140000003240947956515133641660783750523504160629536
020401003559

>1yy9 A
EEKKVCQGTSNKLTQLGTFEDHFLSLQRMFNNCEVVLGNLEITYVQRNYDLSFLKTIQEV
AGYVLIALNTVERIPLENLQIIRGNMYYENSYALAVLSNYDANKTGLKELPMRNLQEILH
GAVRFSNNPALCNVESIQWRDIVSSDFLSNMSMDFQNHLGSCQKCDPSCPNGSCWGAGEE
NCQKLTKIICAQQCSGRCRGKSPSDCCHNQCAAGCTGPRESDCLVCRKFRDEATCKDTCP
PLMLYNPTTYQMDVNPEGKYSFGAT**C**VKKCPRNYVVTDHGSCVRA**C**GADSYEMEEDGVRK
CKKCEGPCRKVCNGIGIGEFKDSLSINATNIKHFKNCTSISGDLHILPVAFRGDSFTHTP
PLDPQELDILKTVKEITGFLLIQAWPENRTDLHAFENLEIIRGRTKQHGQFSLAVVSLNI
TSLGLRSLKEISDGDVIISGNKNL**C**YANTINWKKLFGTSGQKTKIISNRGENKCKATGQV
CHALCSPEGCWGPEPRDCVSCRNVSRGRECVDKCKLLEGEPREFVENSECIQCHPECLPQ
AMNITCTGRGPDNCIQCAHYIDGPHCVKTCPAGVMGENNTLVWKYADAGHVCHLCHPNCT
YGCTGPGLRGCPT
CCCCEECCCCCCCCCCCCCHHHHHHHHHHHHCCCCEECCCEEEEECCCCCCCCHHHHHCCEE
CCCEEEEECCCCCECCCCCCCCEECCCCCCCCCCEEEEEECCECCCCCECCECCCCCCCCEECC
CEEEEECCCCCCHHHCCHHHCECCCCHHHCEECCCCCCCCCCCCCCCCCCHHHCECCCCHH
HECCCCCCCCCCCCCCCCECCCCHHHECCCCECCCECCCCCCCECCECCEECCCCEECCCC
CCEEEEECCCCEEEEECCCCCEEECCE**E**ECCCCCCCCEECCCCCEEECC**C**CCCEEEEEECCCCEE
EEECCCCCCCCEEECCECHHHECCCCCCCCCHHHHCCCCEEECCEEECHHHHHCEHHHCEC

CCCHHHHHHHHHCCEECCCEEECCCCCCCCCCHHHCCCCEECCCCCECCCEEEEEEECCCC
CCCCCCCCCEECCCEEEEEECCCCC**C**CCCCCCHHHCECCCCCCCEEEECECCHHHHHHCCCC
CCCCECCCCECCCCHHHECCECCEECCCCEECCCCCCCCCCCCEEEECCEEEECCCCECCC
CCCCCECCCCCCCECCECCEEECCEEECCCCEEECCHHHCCEEEEECCCCEEEECCCCCC
CCCCCCCHHHCCC
841731302734363465345003204830560210220000020239270400430310
100000020003300013020000753047210000020428881003200011001014
000300501000106301080002881395233425364493692387029500002426
010410343235417400526368120350000007068153140034240753036401
32410358333437184111216110**0**27613392001780313666**2**68825627597112
038196427440300445507926001150065065000040102010102622885814
506363031032041010000001017935100002304103074213830000001030
20000120640230200000032 0**0**00430506501849606220000305550765834
238201930000341301132251125940055034536430003486401712700463
686300616225315522432034103761153230596541201238752046038607
8118133690289

>1z2b A
RECISIHVGQAGVQIGNACWELYCLEHGIQPDGQMPXXXXXXXXXDSFNTFFSETGAGKH
VPRAVFVDLEPTVIDEVRTGTYRQLFHPEQLITGKEDAANNYARGHYTIGKEIIDLVLDR
IRKLADQCTGLQGFLVFHSFGGGTGSGFTSLLMERLSVDYGKKSKLEFSIYPAPQVSTAV
VEPYNSILTTHTTLEHSDCAFMVDNEAIYDICRRNLDIERPTYTNLNRLIGQIVSSITAS
LRFDGALNVDLTEFQTNLVPYPRIHFPLATYAPVISAEKAYHEQLSVAEITNA**C**FEPANQ
MVKCDPRHGKYMACCLLYRGDVVPKDVNAAIATIKTKRSIQFVDW**C**PTGFKVGINYQPPT
VVPGGDLAKVQRAV**C**MLSNTTAIAEAWARLDHKFDLMYAKRAFVHWYVGEGMEEGEFSEA
REDMAALEKDYEEVGI
CEEEEEEEHHHHHHHHHHHHHHHHHHHHCCCCCCCCCXXXXXXXXXCCCCCCECCCCCCCC
CECEEEEECCCCHHHHHCCCCCCCCCCHHHEEECCCCCCCCHHHHHCCCHHHHHHHHHHH
HHHHCCCCCCEEEEEEEEECCCHHHHHHHHHHHHHHHHHHCCCCEEEEEEEECCCCCCCCC
CHHHHHHHHHHHHHCCCCEEEEECCHHHHHHHHHHHCCCCCCCCHHHHHHHHHHHHHHHHCH
HHHCCCCCCCCCHHHHHHHCCCCCCCCCEECCCCCCECCCCCCCCCCHHHHHHH**H**CCHHHC
CCCCCCCCCCEEECEEEEEEECCHHHHHHHHHHHHHHCCCCCECCC**C**CCCCEEEEECCCCC
CCCCCCECCCCEEC**C**CEEEEHHHHHHHHHHHHHHHHHHHHCCCCCCHHHHCCCCCHHHHHHH
HHHHHHHHHHHHHCCCC
500000000320030021001000301505350259999999999968230001319964 1
000000000025104303747154004740002261000100000001102720640062
055017518601000000100110000000000022013407721000000010331410 0
000000000011006003000000010011005510826205350002000000000000
101400003305102610044820000000000003027339683340360062**0**235110
004130361400100000006051730241016107425511052**1**31211000042503
108923007142000**0**000001001200240062042005230001103310054400560
172033036308705 4

>2btp A
MHHHHHHSSGVDLGTENLYFQSMEKTELIQKAKLAEQAERYDDMATCMKAVTEQGAELSN
EERNLLSVAYKNVVGGRRSAWRVISSIEQKTXXXXKKLQLIKDYREKVESELRSICTTVL
ELLDKYLIANATNPESKVFYLKMKGDYFRYLAEVA**C**GDDRKQTIDNSQGAYQEAFDISKK
EMQPTHPIRLGLALNFSVFYYEILNNPELACTLAKTAFDEAIAELDTLNEDSYKDSTLIM
QLLRDNLTLWTS
CCCCCECCCCCECCCCCHHHHCCCHHHHHHHHHHHHHHCCCHHHHHHHHHHHHHHCCCCCCH
HHHHHHHHHHHHHHHHHHHHHHHHHHHHHHHHHCXXXXCHHHHHHHHHHHHHHHHHHHHHHHHHH
HHHHHCCCCCCCCHHHHHHHHHHHHHHHHHHHHHHHC**C**HHHHHHHHHHHHHHHHHHHHHHHHHH
HCCCCCHHHHHHHHHHHHHHHHHHCCCCHHHHHHHHHHHHHHHHHHHCCCCCCHHHHHHHHHHH
HHHHHHHHHCCC
036152443353620437307716364003003003506124000000010002434304 5
510400120033103510301520262225889999624320161144006003300520 0
600542025408432010000002000100101006**6**896322017402500350152054
607102211000000000001230264375016103411550561097269424250250 0
500550165079

>1z7x W
SLDIQSLDIQCEELSDARWAELLPLLQQCQVVRLDDCGLTEARCKDISSALRVNPALAEL
NLRSNELGDVGVHCVLQGLQTPSCKIQKLSLQNCCLTGAGCGVLSSTLRTLPTLQELHLS
DNLLGDAGLQLLCEGLLDPQCRLEKLQLEYCSLSAASCEPLASVLRAKPDFKELTVSNND
INEAGVRVLCQGLKDSPCQLEALKLESCGVTSDNCRDLCGIVASKASLRELALGSNKLGD
VGMAELCPGLLHPSSRLRTLWIWECGITAKGCGDLCRVLRAKESLKELSLAGNELGDEGA
RLLCETLLEPGCQLESLWVKSCSFTAACCSHFSSVLAQNRFLLELQISNNRLEDAGVREL
CQGLGQPGSVLRVLWLADCDVSDSSCSSLAATLLANHSLRELDLSNNCLGDAGILQLVES
VRQPGCLLEQLVLYDIYWSEEMEDRLQALEKDKPSLRVIS
CEEEEEEEEECCCCCHHHHHHHHHHHCCCCEEEEE**C**CCCCHHHHHHHHHHHHCCCCCCEE
ECCCCCCHHHHHHHHHHCCCCCCCCCCEEECCCCCCEHHHHHHHHHHCCCCCCCCEEECC
CCECHHHHHHHHHHHHCCCCCCCCEEECCCCCCEHHHHHHHHHHHHHCCCCCEEECCCCE
CHHHHHHHHHHHHHHCCCCCCEEECCCCCCECCHHHHHHHHHHHCCCCCEEECCCCECHH
HHHHHH**H**HHHCCCCCCCCEEECCCCCCCHHHHHHHHHHHHHCCCCCEEECCCCCCHHHHH
HHHHHHHCCCCCCCCEEECCCCCCEHHHHHHHHHHHHHCCCCCEEECCCCECHHHHHHHH
HHHCCCCCCCCEEECCCCCCCHHHHHHHHHHHHHHCCCCCEEECCCCCCCHHHHHHHHHH
HCCCCCCCCEEECCCCCCCHHHHHHHHHHHHHCCCCEEEC
533202010111805851056024004404103001030445105400600450540110
102208022500310040052931504400013040337004200500450220210100
206022600430050041740200301025060234005100200540120100000206
043300410050055050202002012040225006301100150310420000309021
500320**0**40044740105000003070426002100600540200110000307023400
320040033830003000003050425004301300160420010000207032500520
050025861104001003030326005100510350320120000103003500330040
0646300031000020001270154053037603204102

>1znq O
KVKVGVNGFGRIGRLVTRAAFNSGKVDIVAINDPFIDLNYMVYMFQYDSTHGKFHGTVKA
ENGKLVINGNPITIFQERDPSKIKWGDAGAEYVVESTGVFTTMEKAGAHLQGGAKRVIIS
APSADAPMFVMGVNHEKYDNSLKIISNASCTTNCLAPLAKVIHDNFGIVEGLMTTVHAIT
ATQKTVDGPSGKLWRDGRGALQNIIPASTGAAKAVGKVIPELNGKLTGMAFRVPTANVSV
VDLTCRLEKPAKYDDIKKVVKQASEGPLKGILGYTEHQVVSSDFNSDTHSSTFDAGAGIA
LNDHFVKLISWYDNEFGYSNRVVDLMAHMASKE
CCEEEEECCCHHHHHHHHHHHHHCCCEEEEEECCCCCHHHHHHHHHCCCCCCCCCCCEEE
ECCEEEECCEEEEEECCCCHHHCCHHHHCCCEEEECCCCCCCHHHHCHHHHCCCCEEEEC
CCCCCCCECCCCCCHHHCCCCCCEEECCC**H**HHHHHHHHHHHHHHHHCEEEEEEEEEEECC
CCCCCCCCCCCCCHHHHCCCCCCCEEEECCHHHHHHHHCHHHCCCEEEEEEEECCCCCEE
EEEE**E**EECCCCHHHHHHHHHHHHHHCCCCCCEEEECCCCHHHHCCCCCCEEEEECCCCEE
CCCCEEEEEEEECCCHHHHHHHHHHHHHHHCCC
805000000312000001001843404010001260416001000100010450944053
597401047450211447402605048140300001375132485011038230500001
241850310012101660447251000012000000000030015401020000000002
660400022289420100001200001726007100600560574010000100010000
0000**0**20766061640261047116480740001154300012043232000000060123
22320000000000001000100010021027466
