# Supplementary material for: Comprehensive Identification and Modified-Site Mapping of S-Nitrosylated Targets in Prostate Epithelial Cells
Source: PLoS One. 2010 Feb 5;5(2):e9075. doi: 10.1371/journal.pone.0009075 (PMC2816712; doi:10.1371/journal.pone.0009075)
Supplement: Table S4 — Localization of neighboring charged residues in the vicinity of the SNO. Residues and atoms in contact with SNO cysteines in the mapped structures were identified with the iMolTalk server (http://i.moltalk.org) and the Loopp program, using a cutoff distance of 5 Å from the sulfur atom of the cysteine. (2.47 MB PDF) [file pone.0009075.s004.pdf]

chain1	res1/atm1	distance	res2/atm2	chain2	H-bonding	Charge interaction
2PKTA	CYS73(C)	4.36	TRP14(CZ2)	2PKTA		C[ ] ... C[ ]
2PKTA	CYS73(O)	4.29	TRP14(NE1)	2PKTA	[D-A-AA]:147.7° [A-D-DD]:167.1° d_planarity:-62.1° a_planarity:-28.4°, maximum distance exceeded, bad d_planarity	O[ ] ... N[ ]
2PKTA	CYS73(O)	4.21	TRP14(CE2)	2PKTA		O[ ] ... C[ ]
2PKTA	CYS73(O)	3.42	TRP14(CZ2)	2PKTA		O[ ] ... C[ ]
2PKTA	CYS73(O)	4.36	TRP14(CH2)	2PKTA		O[ ] ... C[ ]
2PKTA	CYS73(CB)	4.46	TRP14(CZ2)	2PKTA		C[ ] ... C[ ]
2PKTA	CYS73(SG)	4.41	ASP54(CG)	2PKTA		S[ ] ... C[ ]
2PKTA	CYS73(SG)	4.65	ASP54(OD1)	2PKTA	negative	
2PKTA	CYS73(SG)	3.55	ASP54(OD2)	2PKTA		S[ ] ... O[-]
2PKTA	CYS73(N)	4.72	ARG69(C)	2PKTA		
2PKTA	CYS73(N)	3.69	ARG69(O)	2PKTA	[D-A-AA]:141.5° [A-D-DD]:120.2° d_planarity:-53.4° a_planarity:55.9°, maximum distance exceeded	N[ ] ... O[ ]
2PKTA	CYS73(CA)	4.60	ARG69(O)	2PKTA		
2PKTA	CYS73(CB)	4.35	ARG69(O)	2PKTA		C[ ] ... O[ ]
2PKTA	CYS73(SG)	4.33	ARG69(C)	2PKTA		S[ ] ... C[ ]
2PKTA	CYS73(SG)	3.27	ARG69(O)	2PKTA	Positive	S[ ] ... O[ ]
2PKTA	CYS73(N)	4.74	ILE70(CA)	2PKTA		
2PKTA	CYS73(N)	3.82	ILE70(C)	2PKTA		N[ ] ... C[ ]
2PKTA	CYS73(N)	3.13	ILE70(O)	2PKTA	**H-bond** [D-A-AA]:115.7° [A-D-DD]:103.3° d_planarity:-12.0° a_planarity:78.1°	N[ ] ... O[ ]
2PKTA	CYS73(CA)	4.68	ILE70(C)	2PKTA		
2PKTA	CYS73(CA)	3.74	ILE70(O)	2PKTA		C[ ] ... O[ ]
2PKTA	CYS73(C)	4.22	ILE70(O)	2PKTA		C[ ] ... O[ ]
2PKTA	CYS73(O)	3.82	ILE70(O)	2PKTA		O[ ] ... O[ ]
2PKTA	CYS73(CB)	4.80	ILE70(CA)	2PKTA		
2PKTA	CYS73(CB)	4.37	ILE70(C)	2PKTA		C[ ] ... C[ ]
2PKTA	CYS73(CB)	3.42	ILE70(O)	2PKTA		C[ ] ... O[ ]
2PKTA	CYS73(SG)	4.79	ILE70(N)	2PKTA	[D-A-AA]:108.7° [A-D-DD]:61.8° d_planarity:17.7°, maximum distance exceeded, bad d_angle(sp2)	
2PKTA	CYS73(SG)	4.30	ILE70(CA)	2PKTA		S[ ] ... C[ ]
2PKTA	CYS73(SG)	4.38	ILE70(C)	2PKTA		S[ ] ... C[ ]
2PKTA	CYS73(SG)	3.85	ILE70(O)	2PKTA		S[ ] ... O[ ]
2PKTA	CYS73(N)	4.33	LYS71(N)	2PKTA		N[ ] ... N[ ]
2PKTA	CYS73(N)	4.25	LYS71(CA)	2PKTA		N[ ] ... C[ ]
2PKTA	CYS73(N)	3.20	LYS71(C)	2PKTA		N[ ] ... C[ ]
2PKTA	CYS73(N)	3.43	LYS71(O)	2PKTA	[D-A-AA]:69.0° [A-D-DD]:131.4° d_planarity:-88.3° a_planarity:50.3°, bad a_angle(sp2), bad d_planarity	N[ ] ... O[ ]
2PKTA	CYS73(CA)	4.49	LYS71(C)	2PKTA		C[ ] ... C[ ]
2PKTA	CYS73(CA)	4.53	LYS71(O)	2PKTA		
2PKTA	CYS73(C)	4.74	LYS71(C)	2PKTA		
2PKTA	CYS73(C)	4.43	LYS71(O)	2PKTA		C[ ] ... O[ ]
2PKTA	CYS73(O)	4.68	LYS71(C)	2PKTA		
2PKTA	CYS73(O)	4.34	LYS71(O)	2PKTA		O[ ] ... O[ ]
2PKTA	CYS73(N)	2.74	GLY72(N)	2PKTA		N[ ] ... N[ ]
2PKTA	CYS73(N)	2.43	GLY72(CA)	2PKTA		N[ ] ... C[ ]
2PKTA	CYS73(N)	2.24	GLY72(O)	2PKTA	[D-A-AA]:30.0° [A-D-DD]:94.3° d_planarity:0.4° a_planarity:0.6°, bad a_angle(sp2)	N[ ] ... O[ ]

2PKTA	CYS73(CA)	4.19	GLY72(N)	2PKTA		C[ ] ... N[ ]
2PKTA	CYS73(CA)	3.81	GLY72(CA)	2PKTA		C[ ] ... C[ ]
2PKTA	CYS73(CA)	2.44	GLY72(C)	2PKTA		C[ ] ... C[ ]
2PKTA	CYS73(CA)	2.77	GLY72(O)	2PKTA		C[ ] ... O[ ]
2PKTA	CYS73(C)	4.84	GLY72(N)	2PKTA		
2PKTA	CYS73(C)	4.52	GLY72(CA)	2PKTA		
2PKTA	CYS73(C)	3.20	GLY72(C)	2PKTA		C[ ] ... C[ ]
2PKTA	CYS73(C)	3.33	GLY72(O)	2PKTA		C[ ] ... O[ ]
2PKTA	CYS73(O)	3.97	GLY72(C)	2PKTA		O[ ] ... C[ ]
2PKTA	CYS73(O)	4.33	GLY72(O)	2PKTA		O[ ] ... O[ ]
2PKTA	CYS73(CB)	4.88	GLY72(N)	2PKTA		
2PKTA	CYS73(CB)	4.83	GLY72(CA)	2PKTA		
2PKTA	CYS73(CB)	3.67	GLY72(C)	2PKTA		C[ ] ... C[ ]
2PKTA	CYS73(CB)	4.18	GLY72(O)	2PKTA		C[ ] ... O[ ]
2PKTA	CYS73(SG)	4.99	GLY72(CA)	2PKTA		
2PKTA	CYS73(SG)	4.12	GLY72(C)	2PKTA		S[ ] ... C[ ]
2PKTA	CYS73(SG)	4.71	GLY72(O)	2PKTA		
2PKTA	CYS73(N)	3.30	THR74(N)	2PKTA		N[ ] ... N[ ]
2PKTA	CYS73(N)	4.59	THR74(CA)	2PKTA		
2PKTA	CYS73(N)	4.91	THR74(OG1)	2PKTA	[D-A-AA]:100.6° [A-D-DD]:75.8° d_planarity:46.2°, maximum distance exceeded, bad d_angle(sp2)	
2PKTA	CYS73(CA)	2.43	THR74(N)	2PKTA		C[ ] ... N[ ]
2PKTA	CYS73(CA)	3.81	THR74(CA)	2PKTA		C[ ] ... C[ ]
2PKTA	CYS73(CA)	4.59	THR74(C)	2PKTA		
2PKTA	CYS73(CA)	4.82	THR74(CB)	2PKTA		
2PKTA	CYS73(CA)	4.76	THR74(OG1)	2PKTA		
2PKTA	CYS73(C)	2.44	THR74(CA)	2PKTA		C[ ] ... C[ ]
2PKTA	CYS73(C)	3.29	THR74(C)	2PKTA		C[ ] ... C[ ]
2PKTA	CYS73(C)	3.98	THR74(O)	2PKTA		C[ ] ... O[ ]
2PKTA	CYS73(C)	3.66	THR74(CB)	2PKTA		C[ ] ... C[ ]
2PKTA	CYS73(C)	3.81	THR74(OG1)	2PKTA		C[ ] ... O[ ]
2PKTA	CYS73(C)	4.89	THR74(CG2)	2PKTA		
2PKTA	CYS73(O)	2.25	THR74(N)	2PKTA	[D-A-AA]:29.6° [A-D-DD]:95.1° d_planarity:0.1° a_planarity:0.4°, bad a_angle(sp2)	O[ ] ... N[ ]
2PKTA	CYS73(O)	2.79	THR74(CA)	2PKTA		O[ ] ... C[ ]
2PKTA	CYS73(O)	3.50	THR74(C)	2PKTA		O[ ] ... C[ ]
2PKTA	CYS73(O)	3.82	THR74(O)	2PKTA		O[ ] ... O[ ]
2PKTA	CYS73(O)	4.16	THR74(CB)	2PKTA		O[ ] ... C[ ]
2PKTA	CYS73(O)	4.34	THR74(OG1)	2PKTA	[D-A-AA]:57.0° [A-D-DD]:73.2° a_planarity:36.9°, maximum distance exceeded, bad a_angle(sp2)	O[ ] ... O[ ]
2PKTA	CYS73(CB)	3.59	THR74(N)	2PKTA		C[ ] ... N[ ]
2PKTA	CYS73(CB)	4.83	THR74(CA)	2PKTA		
2PKTA	CYS73(CA)	4.83	ASP75(N)	2PKTA		
2PKTA	CYS73(C)	3.84	ASP75(N)	2PKTA		C[ ] ... N[ ]
2PKTA	CYS73(O)	4.35	ASP75(N)	2PKTA	[D-A-AA]:58.0° [A-D-DD]:139.0° d_planarity:54.5° a_planarity:35.3°, maximum distance exceeded, bad a_angle(sp2)	O[ ] ... N[ ]
2PKTA	CYS73(CA)	4.16	ASN76(O)	2PKTA		C[ ] ... O[ ]
2PKTA	CYS73(C)	4.74	ASN76(N)	2PKTA		
2PKTA	CYS73(C)	4.44	ASN76(O)	2PKTA		C[ ] ... O[ ]

2PKTA	CYS73(O)	4.86	ASN76(O)	2PKTA		
2PKTA	CYS73(CB)	4.19	ASN76(C)	2PKTA		C[ ] ... C[ ]
2PKTA	CYS73(CB)	3.39	ASN76(O)	2PKTA		C[ ] ... O[ ]
2PKTA	CYS73(SG)	4.99	ASN76(C)	2PKTA		
2PKTA	CYS73(SG)	4.04	ASN76(O)	2PKTA		S[ ] ... O[ ]
2PKTA	CYS73(CB)	4.62	LEU77(N)	2PKTA		
2PKTA	CYS73(CB)	4.31	LEU77(CA)	2PKTA		C[ ] ... C[ ]
2PKTA	CYS73(CB)	4.16	LEU77(CB)	2PKTA		C[ ] ... C[ ]
2PKTA	CYS73(CB)	4.69	LEU77(CG)	2PKTA		
2PKTA	CYS73(CB)	4.06	LEU77(CD1)	2PKTA		C[ ] ... C[ ]
2PKTA	CYS73(SG)	4.54	LEU77(CA)	2PKTA		
2PKTA	CYS73(SG)	4.57	LEU77(CB)	2PKTA		
2PKTA	CYS73(SG)	4.66	LEU77(CG)	2PKTA		
2PKTA	CYS73(SG)	3.53	LEU77(CD1)	2PKTA		S[ ] ... C[ ]
2PKTA	CYS73(CA)	4.91	CA201(CA)	2PKTA		
2PKTA	CYS73(CB)	4.52	CA201(CA)	2PKTA		
2PKTA	CYS73(SG)	4.55	CA201(CA)	2PKTA	ligand	

Site: weak positive, negative, ligand

chain1	res1/atm1	distance	res2/atm2	chain2	H-bonding	Charge interaction
1TUBB	CYS241(N)	4.83	SER236(CA)	1TUBB		
1TUBB	CYS241(N)	3.69	SER236(C)	1TUBB		N[ ] ... C[ ]
1TUBB	CYS241(N)	2.50	SER236(O)	1TUBB	**H-bond** [D-A-AA]:164.4° [A-D-DD]:124.8° d_planarity:-29.1° a_planarity:20.8°	N[ ] ... O[ ]
1TUBB	CYS241(CA)	4.61	SER236(C)	1TUBB		
1TUBB	CYS241(CA)	3.53	SER236(O)	1TUBB		C[ ] ... O[ ]
1TUBB	CYS241(C)	4.69	SER236(O)	1TUBB		
1TUBB	CYS241(O)	4.80	SER236(O)	1TUBB		
1TUBB	CYS241(CB)	4.27	SER236(C)	1TUBB		C[ ] ... C[ ]
1TUBB	CYS241(CB)	3.48	SER236(O)	1TUBB		C[ ] ... O[ ]
1TUBB	CYS241(SG)	4.81	SER236(CA)	1TUBB		
1TUBB	CYS241(SG)	3.44	SER236(C)	1TUBB		S[ ] ... C[ ]
1TUBB	CYS241(SG)	2.92	SER236(O)	1TUBB		S[ ] ... O[ ]
1TUBB	CYS241(SG)	4.88	SER236(OG)	1TUBB	[D-A-AA]:139.9° [A-D-DD]:87.8°, maximum distance exceeded	
1TUBB	CYS241(N)	4.35	GLY237(N)	1TUBB		N[ ] ... N[ ]
1TUBB	CYS241(N)	4.07	GLY237(CA)	1TUBB		N[ ] ... C[ ]
1TUBB	CYS241(N)	3.68	GLY237(C)	1TUBB		N[ ] ... C[ ]
1TUBB	CYS241(N)	3.12	GLY237(O)	1TUBB	[D-A-AA]:107.8° [A-D-DD]:73.8° d_planarity:-22.9° a_planarity:-71.4°, bad d_angle(sp2)	N[ ] ... O[ ]
1TUBB	CYS241(CA)	4.97	GLY237(N)	1TUBB		
1TUBB	CYS241(CA)	4.29	GLY237(CA)	1TUBB		C[ ] ... C[ ]
1TUBB	CYS241(CA)	3.92	GLY237(C)	1TUBB		C[ ] ... C[ ]
1TUBB	CYS241(CA)	3.05	GLY237(O)	1TUBB		C[ ] ... O[ ]
1TUBB	CYS241(C)	4.48	GLY237(C)	1TUBB		C[ ] ... C[ ]
1TUBB	CYS241(C)	3.38	GLY237(O)	1TUBB		C[ ] ... O[ ]
1TUBB	CYS241(O)	4.10	GLY237(C)	1TUBB		O[ ] ... C[ ]
1TUBB	CYS241(O)	2.97	GLY237(O)	1TUBB		O[ ] ... O[ ]

1TUBB	CYS241(CB)	4.24	GLY237(N)	1TUBB		C[ ] ... N[ ]
1TUBB	CYS241(CB)	3.25	GLY237(CA)	1TUBB		C[ ] ... C[ ]
1TUBB	CYS241(CB)	3.07	GLY237(C)	1TUBB		C[ ] ... C[ ]
1TUBB	CYS241(CB)	2.26	GLY237(O)	1TUBB		C[ ] ... O[ ]
1TUBB	CYS241(SG)	3.37	GLY237(N)	1TUBB	[D-A-AA]:106.9° [A-D-DD]:50.2° d_planarity:44.1°, bad d_angle(sp2)	S[ ] ... N[ ]
1TUBB	CYS241(SG)	2.68	GLY237(CA)	1TUBB		S[ ] ... C[ ]
1TUBB	CYS241(SG)	3.31	GLY237(C)	1TUBB		S[ ] ... C[ ]
1TUBB	CYS241(SG)	3.14	GLY237(O)	1TUBB		S[ ] ... O[ ]
1TUBB	CYS241(N)	4.40	VAL238(N)	1TUBB		N[ ] ... N[ ]
1TUBB	CYS241(N)	4.81	VAL238(CA)	1TUBB		
1TUBB	CYS241(N)	4.08	VAL238(C)	1TUBB		N[ ] ... C[ ]
1TUBB	CYS241(N)	4.28	VAL238(O)	1TUBB	[D-A-AA]:72.2° [A-D-DD]:101.9° d_planarity:-67.1° a_planarity:65.9°, maximum distance exceeded, bad a_angle(sp2), bad d_planarity	N[ ] ... O[ ]
1TUBB	CYS241(CA)	4.91	VAL238(N)	1TUBB		
1TUBB	CYS241(CA)	4.77	VAL238(C)	1TUBB		
1TUBB	CYS241(CA)	4.79	VAL238(O)	1TUBB		
1TUBB	CYS241(C)	4.70	VAL238(C)	1TUBB		
1TUBB	CYS241(C)	4.34	VAL238(O)	1TUBB		C[ ] ... O[ ]
1TUBB	CYS241(O)	4.75	VAL238(N)	1TUBB	[D-A-AA]:113.1° [A-D-DD]:70.3° d_planarity:24.4° a_planarity:-12.0°, maximum distance exceeded, bad d_angle(sp2)	
1TUBB	CYS241(O)	4.47	VAL238(CA)	1TUBB		O[ ] ... C[ ]
1TUBB	CYS241(O)	3.82	VAL238(C)	1TUBB		O[ ] ... C[ ]
1TUBB	CYS241(O)	3.29	VAL238(O)	1TUBB		O[ ] ... O[ ]
1TUBB	CYS241(CB)	4.27	VAL238(N)	1TUBB		C[ ] ... N[ ]
1TUBB	CYS241(CB)	4.87	VAL238(CA)	1TUBB		
1TUBB	CYS241(CB)	4.80	VAL238(C)	1TUBB		
1TUBB	CYS241(SG)	4.50	VAL238(N)	1TUBB	[D-A-AA]:71.3° [A-D-DD]:129.1° d_planarity:19.6°, maximum distance exceeded	S[ ] ... N[ ]
1TUBB	CYS241(N)	4.22	THR239(N)	1TUBB		N[ ] ... N[ ]
1TUBB	CYS241(N)	4.40	THR239(CA)	1TUBB		N[ ] ... C[ ]
1TUBB	CYS241(N)	3.34	THR239(C)	1TUBB		N[ ] ... C[ ]
1TUBB	CYS241(N)	3.85	THR239(O)	1TUBB	[D-A-AA]:57.1° [A-D-DD]:135.4° d_planarity:60.1° a_planarity:36.2°, maximum distance exceeded, bad a_angle(sp2), bad d_planarity	N[ ] ... O[ ]
1TUBB	CYS241(CA)	4.62	THR239(C)	1TUBB		
1TUBB	CYS241(CA)	4.98	THR239(O)	1TUBB		
1TUBB	CYS241(C)	4.82	THR239(C)	1TUBB		
1TUBB	CYS241(C)	4.93	THR239(O)	1TUBB		
1TUBB	CYS241(O)	4.78	THR239(N)	1TUBB	[D-A-AA]:119.9° [A-D-DD]:94.7° d_planarity:34.6° a_planarity:-25.8°, maximum distance exceeded	
1TUBB	CYS241(O)	4.46	THR239(C)	1TUBB		O[ ] ... C[ ]
1TUBB	CYS241(O)	4.59	THR239(O)	1TUBB		
1TUBB	CYS241(N)	2.86	THR240(N)	1TUBB		N[ ] ... N[ ]
1TUBB	CYS241(N)	2.43	THR240(CA)	1TUBB		N[ ] ... C[ ]
1TUBB	CYS241(N)	2.21	THR240(O)	1TUBB	[D-A-AA]:31.2° [A-D-DD]:88.9° d_planarity:1.4° a_planarity:11.5°, bad a_angle(sp2), bad d_angle(sp2)	N[ ] ... O[ ]
1TUBB	CYS241(N)	3.06	THR240(CB)	1TUBB		N[ ] ... C[ ]
1TUBB	CYS241(N)	4.37	THR240(OG1)	1TUBB	[D-A-AA]:18.5° [A-D-DD]:147.2° d_planarity:63.1°, maximum distance exceeded, bad a_angle(sp3),	N[ ] ... O[ ]

					bad d_planarity	
1TUBB	CYS241(N)	3.58	THR240(CG2)	1TUBB		N[ ] ... C[ ]
1TUBB	CYS241(CA)	4.29	THR240(N)	1TUBB		C[ ] ... N[ ]
1TUBB	CYS241(CA)	3.75	THR240(CA)	1TUBB		C[ ] ... C[ ]
1TUBB	CYS241(CA)	2.37	THR240(C)	1TUBB		C[ ] ... C[ ]
1TUBB	CYS241(CA)	2.62	THR240(O)	1TUBB		C[ ] ... O[ ]
1TUBB	CYS241(CA)	4.26	THR240(CB)	1TUBB		C[ ] ... C[ ]
1TUBB	CYS241(CA)	4.39	THR240(CG2)	1TUBB		C[ ] ... C[ ]
1TUBB	CYS241(C)	4.88	THR240(N)	1TUBB		
1TUBB	CYS241(C)	4.48	THR240(CA)	1TUBB		C[ ] ... C[ ]
1TUBB	CYS241(C)	3.03	THR240(C)	1TUBB		C[ ] ... C[ ]
1TUBB	CYS241(C)	2.94	THR240(O)	1TUBB		C[ ] ... O[ ]
1TUBB	CYS241(O)	4.80	THR240(N)	1TUBB	[D-A-AA]:86.7° [A-D-DD]:78.8° d_planarity:53.4° a_planarity:-43.3°, maximum distance exceeded, bad a_angle(sp2), bad d_angle(sp2)	
1TUBB	CYS241(O)	4.74	THR240(CA)	1TUBB		
1TUBB	CYS241(O)	3.47	THR240(C)	1TUBB		O[ ] ... C[ ]
1TUBB	CYS241(O)	3.61	THR240(O)	1TUBB		O[ ] ... O[ ]
1TUBB	CYS241(CB)	4.79	THR240(CA)	1TUBB		
1TUBB	CYS241(CB)	3.62	THR240(C)	1TUBB		C[ ] ... C[ ]
1TUBB	CYS241(CB)	4.08	THR240(O)	1TUBB		C[ ] ... O[ ]
1TUBB	CYS241(SG)	4.93	THR240(CA)	1TUBB		
1TUBB	CYS241(SG)	4.14	THR240(C)	1TUBB		S[ ] ... C[ ]
1TUBB	CYS241(SG)	4.78	THR240(O)	1TUBB		
1TUBB	CYS241(SG)	4.71	THR240(CB)	1TUBB		
1TUBB	CYS241(SG)	4.96	THR240(CG2)	1TUBB		
1TUBB	CYS241(N)	3.43	LEU242(N)	1TUBB		N[ ] ... N[ ]
1TUBB	CYS241(N)	4.61	LEU242(CA)	1TUBB		
1TUBB	CYS241(N)	4.98	LEU242(C)	1TUBB		
1TUBB	CYS241(N)	4.92	LEU242(CB)	1TUBB		
1TUBB	CYS241(CA)	2.37	LEU242(N)	1TUBB		C[ ] ... N[ ]
1TUBB	CYS241(CA)	3.70	LEU242(CA)	1TUBB		C[ ] ... C[ ]
1TUBB	CYS241(CA)	4.41	LEU242(C)	1TUBB		C[ ] ... C[ ]
1TUBB	CYS241(CA)	4.10	LEU242(CB)	1TUBB		C[ ] ... C[ ]
1TUBB	CYS241(CA)	4.19	LEU242(CG)	1TUBB		C[ ] ... C[ ]
1TUBB	CYS241(C)	2.34	LEU242(CA)	1TUBB		C[ ] ... C[ ]
1TUBB	CYS241(C)	3.16	LEU242(C)	1TUBB		C[ ] ... C[ ]
1TUBB	CYS241(C)	4.25	LEU242(O)	1TUBB		C[ ] ... O[ ]
1TUBB	CYS241(C)	2.62	LEU242(CB)	1TUBB		C[ ] ... C[ ]
1TUBB	CYS241(C)	2.96	LEU242(CG)	1TUBB		C[ ] ... C[ ]
1TUBB	CYS241(C)	4.09	LEU242(CD1)	1TUBB		C[ ] ... C[ ]
1TUBB	CYS241(C)	4.00	LEU242(CD2)	1TUBB		C[ ] ... C[ ]
1TUBB	CYS241(O)	2.21	LEU242(N)	1TUBB	[D-A-AA]:29.1° [A-D-DD]:91.1° d_planarity:0.6° a_planarity:3.0°, bad a_angle(sp2)	O[ ] ... N[ ]
1TUBB	CYS241(O)	2.66	LEU242(CA)	1TUBB		O[ ] ... C[ ]
1TUBB	CYS241(O)	3.37	LEU242(C)	1TUBB		O[ ] ... C[ ]
1TUBB	CYS241(O)	4.25	LEU242(O)	1TUBB		O[ ] ... O[ ]
1TUBB	CYS241(O)	2.27	LEU242(CB)	1TUBB		O[ ] ... C[ ]
1TUBB	CYS241(O)	2.64	LEU242(CG)	1TUBB		O[ ] ... C[ ]
1TUBB	CYS241(O)	3.41	LEU242(CD1)	1TUBB		O[ ] ... C[ ]

1TUBB	CYS241(O)	4.00	LEU242(CD2)	1TUBB		O[ ] ... C[ ]
1TUBB	CYS241(CB)	3.33	LEU242(N)	1TUBB		C[ ] ... N[ ]
1TUBB	CYS241(CB)	4.53	LEU242(CA)	1TUBB		
1TUBB	CYS241(CB)	4.60	LEU242(CB)	1TUBB		
1TUBB	CYS241(CB)	4.20	LEU242(CG)	1TUBB		C[ ] ... C[ ]
1TUBB	CYS241(CB)	4.97	LEU242(CD1)	1TUBB		
1TUBB	CYS241(SG)	4.85	LEU242(N)	1TUBB	[D-A-AA]:24.8° [A-D-DD]:153.8° d_planarity:38.7°, maximum distance exceeded, bad a_angle(sp3)	
1TUBB	CYS241(N)	4.20	ARG243(N)	1TUBB		N[ ] ... N[ ]
1TUBB	CYS241(N)	4.96	ARG243(CA)	1TUBB		
1TUBB	CYS241(N)	4.61	ARG243(CB)	1TUBB		
1TUBB	CYS241(N)	4.76	ARG243(CG)	1TUBB		
1TUBB	CYS241(CA)	3.94	ARG243(N)	1TUBB		C[ ] ... N[ ]
1TUBB	CYS241(C)	2.93	ARG243(N)	1TUBB		C[ ] ... N[ ]
1TUBB	CYS241(C)	4.23	ARG243(CA)	1TUBB		C[ ] ... C[ ]
1TUBB	CYS241(C)	4.89	ARG243(C)	1TUBB		
1TUBB	CYS241(C)	4.55	ARG243(CB)	1TUBB		
1TUBB	CYS241(C)	4.60	ARG243(CG)	1TUBB		
1TUBB	CYS241(O)	3.18	ARG243(N)	1TUBB	[D-A-AA]:67.1° [A-D-DD]:145.3° d_planarity:-30.7° a_planarity:51.0°, bad a_angle(sp2)	O[ ] ... N[ ]
1TUBB	CYS241(O)	4.45	ARG243(CA)	1TUBB		O[ ] ... C[ ]
1TUBB	CYS241(O)	4.59	ARG243(CB)	1TUBB		
1TUBB	CYS241(O)	4.24	ARG243(CG)	1TUBB		O[ ] ... C[ ]
1TUBB	CYS241(N)	4.64	PHE244(N)	1TUBB		
1TUBB	CYS241(CA)	4.57	PHE244(N)	1TUBB		
1TUBB	CYS241(C)	4.31	PHE244(N)	1TUBB		C[ ] ... N[ ]
1TUBB	CYS241(C)	4.64	PHE244(O)	1TUBB		
1TUBB	CYS241(O)	4.36	ALA250(O)	1TUBB		O[ ] ... O[ ]
1TUBB	CYS241(CB)	4.13	VAL318(CG1)	1TUBB		C[ ] ... C[ ]
1TUBB	CYS241(SG)	4.84	VAL318(CG1)	1TUBB		
1TUBB	CYS241(N)	4.98	ARG320(CZ)	1TUBB		
1TUBB	CYS241(N)	3.75	ARG320(NH1)	1TUBB		N[ ] ... N[+]
1TUBB	CYS241(CA)	4.76	ARG320(CD)	1TUBB		
1TUBB	CYS241(CA)	3.96	ARG320(NH1)	1TUBB		C[ ] ... N[+]
1TUBB	CYS241(CB)	4.08	ARG320(CD)	1TUBB		C[ ] ... C[ ]
1TUBB	CYS241(CB)	4.72	ARG320(NE)	1TUBB		
1TUBB	CYS241(CB)	4.59	ARG320(CZ)	1TUBB		
1TUBB	CYS241(CB)	3.71	ARG320(NH1)	1TUBB		C[ ] ... N[+]
1TUBB	CYS241(SG)	4.58	ARG320(CB)	1TUBB		
1TUBB	CYS241(SG)	4.08	ARG320(CG)	1TUBB		S[ ] ... C[ ]
1TUBB	CYS241(SG)	2.84	ARG320(CD)	1TUBB	Positive	S[ ] ... C[ ]
1TUBB	CYS241(SG)	3.10	ARG320(NE)	1TUBB	[D-A-AA]:150.2° [A-D-DD]:68.3° d_planarity:34.9°, bad d_angle(sp2)	S[ ] ... N[ ]
1TUBB	CYS241(SG)	2.89	ARG320(CZ)	1TUBB		S[ ] ... C[ ]
1TUBB	CYS241(SG)	2.20	ARG320(NH1)	1TUBB	**H-bond** [D-A-AA]:138.1° [A-D-DD]:109.3° d_planarity:-52.5°	S[ ] ... N[+]
1TUBB	CYS241(SG)	3.94	ARG320(NH2)	1TUBB	[D-A-AA]:164.6° [A-D-DD]:30.3° d_planarity:56.2°, bad d_angle(sp2)	S[ ] ... N[+]
1TUBB	CYS241(N)	4.97	CYS356(SG)	1TUBB	[D-A-AA]:103.7° [A-D-DD]:13.5° d_planarity:15.3°, maximum distance exceeded, bad d_angle(sp2)	
1TUBB	CYS241(CA)	4.32	CYS356(CB)	1TUBB		C[ ] ... C[ ]

1TUBB	CYS241(CA)	3.58	CYS356(SG)	1TUBB		C[ ] ... S[ ]
1TUBB	CYS241(C)	4.37	CYS356(CB)	1TUBB		C[ ] ... C[ ]
1TUBB	CYS241(C)	3.10	CYS356(SG)	1TUBB		C[ ] ... S[ ]
1TUBB	CYS241(O)	4.00	CYS356(SG)	1TUBB		O[ ] ... S[ ]
1TUBB	CYS241(CB)	4.49	CYS356(CB)	1TUBB		C[ ] ... C[ ]
1TUBB	CYS241(CB)	3.86	CYS356(SG)	1TUBB	Possible disulphide	C[ ] ... S[ ]
1TUBB	CYS241(SG)	4.86	THR376(OG1)	1TUBB	[D-A-AA]:109.1° [A-D-DD]:121.8°, maximum distance exceeded	

chain1	res1/atm1	distance	res2/atm2	chain2	H-bonding	Charge interaction
3DU7A	CYS347(CB)	4.82	TYR312(O)	3DU7A		
3DU7A	CYS347(SG)	5.00	TYR312(O)	3DU7A		
3DU7A	CYS347(CB)	4.16	MET313(CA)	3DU7A		C[ ] ... C[ ]
3DU7A	CYS347(CB)	4.20	MET313(C)	3DU7A		C[ ] ... C[ ]
3DU7A	CYS347(CB)	4.01	MET313(O)	3DU7A		C[ ] ... O[ ]
3DU7A	CYS347(CB)	4.66	MET313(CB)	3DU7A		
3DU7A	CYS347(SG)	4.88	MET313(N)	3DU7A	[D-A-AA]:93.0° [A-D-DD]:35.0° d_planarity:68.0°, maximum distance exceeded, bad d_angle(sp2), bad d_planarity	
3DU7A	CYS347(SG)	3.77	MET313(CA)	3DU7A		S[ ] ... C[ ]
3DU7A	CYS347(SG)	3.21	MET313(C)	3DU7A		S[ ] ... C[ ]
3DU7A	CYS347(SG)	3.04	MET313(O)	3DU7A		S[ ] ... O[ ]
3DU7A	CYS347(SG)	4.64	MET313(CB)	3DU7A		
3DU7A	CYS347(SG)	3.70	ALA314(N)	3DU7A	[D-A-AA]:126.4° [A-D-DD]:86.0° d_planarity:46.8°, bad d_angle(sp2)	S[ ] ... N[ ]
3DU7A	CYS347(SG)	3.89	ALA314(CA)	3DU7A		S[ ] ... C[ ]
3DU7A	CYS347(SG)	4.32	ALA314(CB)	3DU7A		S[ ] ... C[ ]
3DU7A	CYS347(C)	4.90	PHE343(CD2)	3DU7A		
3DU7A	CYS347(O)	4.25	PHE343(CB)	3DU7A		O[ ] ... C[ ]
3DU7A	CYS347(O)	3.92	PHE343(CG)	3DU7A		O[ ] ... C[ ]
3DU7A	CYS347(O)	4.47	PHE343(CD1)	3DU7A		O[ ] ... C[ ]
3DU7A	CYS347(O)	3.79	PHE343(CD2)	3DU7A		O[ ] ... C[ ]
3DU7A	CYS347(O)	4.86	PHE343(CE1)	3DU7A		
3DU7A	CYS347(O)	4.24	PHE343(CE2)	3DU7A		O[ ] ... C[ ]
3DU7A	CYS347(O)	4.75	PHE343(CZ)	3DU7A		
3DU7A	CYS347(CB)	4.72	PHE343(CD2)	3DU7A		
3DU7A	CYS347(CB)	4.90	PHE343(CE2)	3DU7A		
3DU7A	CYS347(SG)	4.47	PHE343(CD2)	3DU7A		S[ ] ... C[ ]
3DU7A	CYS347(SG)	4.12	PHE343(CE2)	3DU7A		S[ ] ... C[ ]
3DU7A	CYS347(SG)	4.88	PHE343(CZ)	3DU7A		
3DU7A	CYS347(N)	4.29	VAL344(C)	3DU7A		N[ ] ... C[ ]
3DU7A	CYS347(N)	3.27	VAL344(O)	3DU7A	**H-bond** [D-A-AA]:141.1° [A-D-DD]:134.5° d_planarity:-21.8° a_planarity:84.1°	N[ ] ... O[ ]
3DU7A	CYS347(N)	4.78	VAL344(CG1)	3DU7A		
3DU7A	CYS347(CA)	4.42	VAL344(O)	3DU7A		C[ ] ... O[ ]
3DU7A	CYS347(CB)	4.43	VAL344(O)	3DU7A		C[ ] ... O[ ]
3DU7A	CYS347(CB)	4.49	VAL344(CG1)	3DU7A		C[ ] ... C[ ]
3DU7A	CYS347(N)	3.98	ASP345(C)	3DU7A		N[ ] ... C[ ]

3DU7A	CYS347(N)	4.48	ASP345(O)	3DU7A	[D-A-AA]:59.0° [A-D-DD]:153.7° d_planarity:88.8° a_planarity:27.6°, maximum distance exceeded, bad a_angle(sp2), bad d_planarity	N[ ] ... O[ ]
3DU7A	CYS347(N)	2.89	TRP346(N)	3DU7A		N[ ] ... N[ ]
3DU7A	CYS347(N)	2.48	TRP346(CA)	3DU7A		N[ ] ... C[ ]
3DU7A	CYS347(N)	2.24	TRP346(O)	3DU7A	[D-A-AA]:30.4° [A-D-DD]:92.8° d_planarity:1.3° a_planarity:1.3°, bad a_angle(sp2)	N[ ] ... O[ ]
3DU7A	CYS347(N)	3.54	TRP346(CB)	3DU7A		N[ ] ... C[ ]
3DU7A	CYS347(N)	3.83	TRP346(CG)	3DU7A		N[ ] ... C[ ]
3DU7A	CYS347(N)	4.94	TRP346(CD1)	3DU7A		
3DU7A	CYS347(N)	3.62	TRP346(CD2)	3DU7A		N[ ] ... C[ ]
3DU7A	CYS347(N)	4.75	TRP346(CE2)	3DU7A		
3DU7A	CYS347(N)	3.16	TRP346(CE3)	3DU7A		N[ ] ... C[ ]
3DU7A	CYS347(N)	4.04	TRP346(CZ3)	3DU7A		N[ ] ... C[ ]
3DU7A	CYS347(CA)	4.36	TRP346(N)	3DU7A		C[ ] ... N[ ]
3DU7A	CYS347(CA)	3.85	TRP346(CA)	3DU7A		C[ ] ... C[ ]
3DU7A	CYS347(CA)	2.44	TRP346(C)	3DU7A		C[ ] ... C[ ]
3DU7A	CYS347(CA)	2.74	TRP346(O)	3DU7A		C[ ] ... O[ ]
3DU7A	CYS347(CA)	4.78	TRP346(CB)	3DU7A		
3DU7A	CYS347(CA)	4.49	TRP346(CD2)	3DU7A		C[ ] ... C[ ]
3DU7A	CYS347(CA)	3.60	TRP346(CE3)	3DU7A		C[ ] ... C[ ]
3DU7A	CYS347(CA)	4.10	TRP346(CZ3)	3DU7A		C[ ] ... C[ ]
3DU7A	CYS347(C)	4.65	TRP346(CA)	3DU7A		
3DU7A	CYS347(C)	3.34	TRP346(C)	3DU7A		C[ ] ... C[ ]
3DU7A	CYS347(C)	3.58	TRP346(O)	3DU7A		C[ ] ... O[ ]
3DU7A	CYS347(O)	4.18	TRP346(C)	3DU7A		O[ ] ... C[ ]
3DU7A	CYS347(O)	4.65	TRP346(O)	3DU7A		
3DU7A	CYS347(CB)	4.83	TRP346(CA)	3DU7A		
3DU7A	CYS347(CB)	3.60	TRP346(C)	3DU7A		C[ ] ... C[ ]
3DU7A	CYS347(CB)	4.01	TRP346(O)	3DU7A		C[ ] ... O[ ]
3DU7A	CYS347(CB)	4.56	TRP346(CD2)	3DU7A		
3DU7A	CYS347(CB)	3.57	TRP346(CE3)	3DU7A		C[ ] ... C[ ]
3DU7A	CYS347(CB)	3.55	TRP346(CZ3)	3DU7A		C[ ] ... C[ ]
3DU7A	CYS347(CB)	4.53	TRP346(CH2)	3DU7A		
3DU7A	CYS347(SG)	4.75	TRP346(CZ3)	3DU7A		
3DU7A	CYS347(N)	3.32	PRO348(N)	3DU7A		N[ ] ... N[ ]
3DU7A	CYS347(N)	4.45	PRO348(CA)	3DU7A		N[ ] ... C[ ]
3DU7A	CYS347(N)	4.92	PRO348(C)	3DU7A		
3DU7A	CYS347(N)	4.72	PRO348(O)	3DU7A	[D-A-AA]:91.8° [A-D-DD]:73.9° d_planarity:-74.8° a_planarity:-43.1°, maximum distance exceeded, bad d_angle(sp2), bad d_planarity	
3DU7A	CYS347(N)	3.95	PRO348(CD)	3DU7A		N[ ] ... C[ ]
3DU7A	CYS347(CA)	2.47	PRO348(N)	3DU7A		C[ ] ... N[ ]
3DU7A	CYS347(CA)	3.82	PRO348(CA)	3DU7A		C[ ] ... C[ ]
3DU7A	CYS347(CA)	4.39	PRO348(C)	3DU7A		C[ ] ... C[ ]
3DU7A	CYS347(CA)	4.54	PRO348(O)	3DU7A		
3DU7A	CYS347(CA)	4.76	PRO348(CB)	3DU7A		
3DU7A	CYS347(CA)	4.39	PRO348(CG)	3DU7A		C[ ] ... C[ ]
3DU7A	CYS347(CA)	3.01	PRO348(CD)	3DU7A		C[ ] ... C[ ]
3DU7A	CYS347(C)	2.46	PRO348(CA)	3DU7A		C[ ] ... C[ ]

3DU7A	CYS347(C)	2.88	PRO348(C)	3DU7A		C[ ] ... C[ ]
3DU7A	CYS347(C)	3.17	PRO348(O)	3DU7A		C[ ] ... O[ ]
3DU7A	CYS347(C)	3.50	PRO348(CB)	3DU7A		C[ ] ... C[ ]
3DU7A	CYS347(C)	3.55	PRO348(CG)	3DU7A		C[ ] ... C[ ]
3DU7A	CYS347(C)	2.52	PRO348(CD)	3DU7A		C[ ] ... C[ ]
3DU7A	CYS347(O)	2.25	PRO348(N)	3DU7A		O[ ] ... N[ ]
3DU7A	CYS347(O)	2.83	PRO348(CA)	3DU7A		O[ ] ... C[ ]
3DU7A	CYS347(O)	2.55	PRO348(C)	3DU7A		O[ ] ... C[ ]
3DU7A	CYS347(O)	2.64	PRO348(O)	3DU7A		O[ ] ... O[ ]
3DU7A	CYS347(O)	3.92	PRO348(CB)	3DU7A		O[ ] ... C[ ]
3DU7A	CYS347(O)	4.35	PRO348(CG)	3DU7A		O[ ] ... C[ ]
3DU7A	CYS347(O)	3.59	PRO348(CD)	3DU7A		O[ ] ... C[ ]
3DU7A	CYS347(CB)	3.67	PRO348(N)	3DU7A		C[ ] ... N[ ]
3DU7A	CYS347(CB)	4.99	PRO348(CA)	3DU7A		
3DU7A	CYS347(CB)	4.18	PRO348(CD)	3DU7A		C[ ] ... C[ ]
3DU7A	CYS347(SG)	4.14	PRO348(N)	3DU7A		S[ ] ... N[ ]
3DU7A	CYS347(SG)	4.53	PRO348(CD)	3DU7A		
3DU7A	CYS347(C)	3.72	THR349(N)	3DU7A		C[ ] ... N[ ]
3DU7A	CYS347(C)	4.64	THR349(CA)	3DU7A		
3DU7A	CYS347(O)	3.23	THR349(N)	3DU7A	**H-bond** [D-A-AA]:103.9° [A-D-DD]:102.7° d_planarity:47.6° a_planarity:5.8°	O[ ] ... N[ ]
3DU7A	CYS347(O)	3.82	THR349(CA)	3DU7A		O[ ] ... C[ ]
3DU7A	CYS347(O)	4.63	THR349(C)	3DU7A		
3DU7A	CYS347(O)	4.91	THR349(OG1)	3DU7A	[D-A-AA]:124.6° [A-D-DD]:86.8° a_planarity:27.7°, maximum distance exceeded	
3DU7A	CYS347(O)	4.33	GLY350(N)	3DU7A	[D-A-AA]:117.5° [A-D-DD]:129.7° d_planarity:-46.0° a_planarity:47.9°, maximum distance exceeded	O[ ] ... N[ ]
3DU7A	CYS347(N)	4.17	PRO27(O)	3DU7E	[D-A-AA]:131.8° [A-D-DD]:117.8° d_planarity:63.2° a_planarity:-28.4°, maximum distance exceeded, bad d_planarity	N[ ] ... O[ ]
3DU7A	CYS347(C)	4.71	PRO27(CA)	3DU7E		
3DU7A	CYS347(C)	4.77	PRO27(O)	3DU7E		

chain1	res1/atm1	distance	res2/atm2	chain2	H-bonding	Charge interaction
3DU7A	CYS376(SG)	4.55	ILE234(O)	3DU7A		
3DU7A	CYS376(CB)	4.22	SER237(CB)	3DU7A		C[ ] ... C[ ]
3DU7A	CYS376(SG)	4.74	SER237(CA)	3DU7A		
3DU7A	CYS376(SG)	4.57	SER237(C)	3DU7A		
3DU7A	CYS376(SG)	4.90	SER237(O)	3DU7A		
3DU7A	CYS376(SG)	3.73	SER237(CB)	3DU7A		S[ ] ... C[ ]
3DU7A	CYS376(SG)	4.70	SER237(OG)	3DU7A	[D-A-AA]:89.9° [A-D-DD]:40.6°, maximum distance exceeded, bad d_angle(sp3)	
3DU7A	CYS376(SG)	4.68	ILE238(N)	3DU7A	[D-A-AA]:116.9° [A-D-DD]:102.1° d_planarity:81.8°, maximum distance exceeded, bad d_planarity	
3DU7A	CYS376(SG)	4.48	ILE238(CG1)	3DU7A		S[ ] ... C[ ]
3DU7A	CYS376(O)	4.08	ALA270(CA)	3DU7A		O[ ] ... C[ ]
3DU7A	CYS376(O)	3.86	ALA270(C)	3DU7A		O[ ] ... C[ ]
3DU7A	CYS376(O)	4.95	ALA270(O)	3DU7A		

3DU7A	CYS376(O)	4.13	ALA270(CB)	3DU7A		O[ ] ... C[ ]
3DU7A	CYS376(SG)	4.89	ALA270(CB)	3DU7A		
3DU7A	CYS376(N)	4.62	THR271(O)	3DU7A	[D-A-AA]:114.0° [A-D-DD]:64.6° d_planarity:70.7° a_planarity:71.8°, maximum distance exceeded, bad d_angle(sp2), bad d_planarity	
3DU7A	CYS376(CA)	4.51	THR271(C)	3DU7A		
3DU7A	CYS376(CA)	4.20	THR271(O)	3DU7A		C[ ] ... O[ ]
3DU7A	CYS376(C)	4.08	THR271(N)	3DU7A		C[ ] ... N[ ]
3DU7A	CYS376(C)	4.58	THR271(CA)	3DU7A		
3DU7A	CYS376(C)	4.23	THR271(C)	3DU7A		C[ ] ... C[ ]
3DU7A	CYS376(C)	4.06	THR271(O)	3DU7A		C[ ] ... O[ ]
3DU7A	CYS376(C)	4.91	THR271(CB)	3DU7A		
3DU7A	CYS376(O)	2.90	THR271(N)	3DU7A	**H-bond** [D-A-AA]:158.6° [A-D-DD]:106.5° d_planarity:-25.7° a_planarity:-90.0°	O[ ] ... N[ ]
3DU7A	CYS376(O)	3.60	THR271(CA)	3DU7A		O[ ] ... C[ ]
3DU7A	CYS376(O)	3.52	THR271(C)	3DU7A		O[ ] ... C[ ]
3DU7A	CYS376(O)	3.67	THR271(O)	3DU7A		O[ ] ... O[ ]
3DU7A	CYS376(O)	4.13	THR271(CB)	3DU7A		O[ ] ... C[ ]
3DU7A	CYS376(O)	4.54	THR271(OG1)	3DU7A	[D-A-AA]:129.1° [A-D-DD]:64.6° a_planarity:55.5°, maximum distance exceeded	
3DU7A	CYS376(CA)	4.72	TYR272(N)	3DU7A		
3DU7A	CYS376(CA)	4.50	TYR272(CA)	3DU7A		C[ ] ... C[ ]
3DU7A	CYS376(C)	4.75	TYR272(N)	3DU7A		
3DU7A	CYS376(C)	4.99	TYR272(CA)	3DU7A		
3DU7A	CYS376(O)	4.04	TYR272(N)	3DU7A	[D-A-AA]:118.2° [A-D-DD]:103.0° d_planarity:60.3° a_planarity:-37.5°, maximum distance exceeded, bad d_planarity	O[ ] ... N[ ]
3DU7A	CYS376(O)	4.60	TYR272(CA)	3DU7A		
3DU7A	CYS376(SG)	4.94	TYR272(N)	3DU7A	[D-A-AA]:100.6° [A-D-DD]:78.1° d_planarity:-89.9°, maximum distance exceeded, bad d_angle(sp2), bad d_planarity	
3DU7A	CYS376(SG)	4.86	TYR272(CA)	3DU7A		
3DU7A	CYS376(SG)	4.80	TYR272(CD1)	3DU7A		
3DU7A	CYS376(N)	4.75	ALA273(N)	3DU7A		
3DU7A	CYS376(N)	4.76	ALA273(CB)	3DU7A		
3DU7A	CYS376(CA)	4.91	ALA273(N)	3DU7A		
3DU7A	CYS376(N)	4.17	LEU318(N)	3DU7A		N[ ] ... N[ ]
3DU7A	CYS376(N)	4.46	LEU318(CA)	3DU7A		N[ ] ... C[ ]
3DU7A	CYS376(N)	3.82	LEU318(C)	3DU7A		N[ ] ... C[ ]
3DU7A	CYS376(N)	2.74	LEU318(O)	3DU7A	[D-A-AA]:145.8° [A-D-DD]:106.6° d_planarity:-76.9° a_planarity:-53.0°, bad d_planarity	N[ ] ... O[ ]
3DU7A	CYS376(N)	4.83	LEU318(CB)	3DU7A		
3DU7A	CYS376(CA)	4.37	LEU318(N)	3DU7A		C[ ] ... N[ ]
3DU7A	CYS376(CA)	4.70	LEU318(CA)	3DU7A		
3DU7A	CYS376(CA)	4.44	LEU318(C)	3DU7A		C[ ] ... C[ ]
3DU7A	CYS376(CA)	3.45	LEU318(O)	3DU7A		C[ ] ... O[ ]
3DU7A	CYS376(CA)	4.65	LEU318(CB)	3DU7A		
3DU7A	CYS376(C)	4.44	LEU318(N)	3DU7A		C[ ] ... N[ ]
3DU7A	CYS376(C)	4.50	LEU318(O)	3DU7A		C[ ] ... O[ ]
3DU7A	CYS376(CB)	4.07	LEU318(N)	3DU7A		C[ ] ... N[ ]
3DU7A	CYS376(CB)	4.03	LEU318(CA)	3DU7A		C[ ] ... C[ ]
3DU7A	CYS376(CB)	3.92	LEU318(C)	3DU7A		C[ ] ... C[ ]

3DU7A	CYS376(CB)	3.07	LEU318(O)	3DU7A		C[ ] ... O[ ]
3DU7A	CYS376(CB)	3.57	LEU318(CB)	3DU7A		C[ ] ... C[ ]
3DU7A	CYS376(CB)	4.95	LEU318(CG)	3DU7A		
3DU7A	CYS376(SG)	4.76	LEU318(O)	3DU7A		
3DU7A	CYS376(SG)	4.64	LEU318(CB)	3DU7A		
3DU7A	CYS376(N)	4.86	TYR319(N)	3DU7A		
3DU7A	CYS376(N)	4.57	ALA374(C)	3DU7A		
3DU7A	CYS376(N)	4.64	ALA374(O)	3DU7A	[D-A-AA]:79.3° [A-D-DD]:135.7° d_planarity:44.3° a_planarity:18.5°, maximum distance exceeded, bad a_angle(sp2)	
3DU7A	CYS376(N)	3.68	VAL375(N)	3DU7A		N[ ] ... N[ ]
3DU7A	CYS376(N)	2.42	VAL375(CA)	3DU7A		N[ ] ... C[ ]
3DU7A	CYS376(N)	2.24	VAL375(O)	3DU7A	[D-A-AA]:30.0° [A-D-DD]:91.1° d_planarity:1.6° a_planarity:2.0°, bad a_angle(sp2)	N[ ] ... O[ ]
3DU7A	CYS376(N)	2.86	VAL375(CB)	3DU7A		N[ ] ... C[ ]
3DU7A	CYS376(N)	2.91	VAL375(CG1)	3DU7A		N[ ] ... C[ ]
3DU7A	CYS376(N)	4.35	VAL375(CG2)	3DU7A		N[ ] ... C[ ]
3DU7A	CYS376(CA)	4.85	VAL375(N)	3DU7A		
3DU7A	CYS376(CA)	3.77	VAL375(CA)	3DU7A		C[ ] ... C[ ]
3DU7A	CYS376(CA)	2.39	VAL375(C)	3DU7A		C[ ] ... C[ ]
3DU7A	CYS376(CA)	2.69	VAL375(O)	3DU7A		C[ ] ... O[ ]
3DU7A	CYS376(CA)	4.20	VAL375(CB)	3DU7A		C[ ] ... C[ ]
3DU7A	CYS376(CA)	4.23	VAL375(CG1)	3DU7A		C[ ] ... C[ ]
3DU7A	CYS376(C)	4.98	VAL375(CA)	3DU7A		
3DU7A	CYS376(C)	3.73	VAL375(C)	3DU7A		C[ ] ... C[ ]
3DU7A	CYS376(C)	4.09	VAL375(O)	3DU7A		C[ ] ... O[ ]
3DU7A	CYS376(C)	4.79	VAL375(CG1)	3DU7A		
3DU7A	CYS376(O)	4.60	VAL375(C)	3DU7A		
3DU7A	CYS376(O)	4.69	VAL375(O)	3DU7A		
3DU7A	CYS376(CB)	4.46	VAL375(CA)	3DU7A		C[ ] ... C[ ]
3DU7A	CYS376(CB)	3.15	VAL375(C)	3DU7A		C[ ] ... C[ ]
3DU7A	CYS376(CB)	3.28	VAL375(O)	3DU7A		C[ ] ... O[ ]
3DU7A	CYS376(SG)	4.46	VAL375(C)	3DU7A		S[ ] ... C[ ]
3DU7A	CYS376(SG)	4.16	VAL375(O)	3DU7A		S[ ] ... O[ ]
3DU7A	CYS376(N)	3.01	MET377(N)	3DU7A		N[ ] ... N[ ]
3DU7A	CYS376(N)	4.40	MET377(CA)	3DU7A		N[ ] ... C[ ]
3DU7A	CYS376(N)	4.83	MET377(CB)	3DU7A		
3DU7A	CYS376(CA)	2.44	MET377(N)	3DU7A		C[ ] ... N[ ]
3DU7A	CYS376(CA)	3.82	MET377(CA)	3DU7A		C[ ] ... C[ ]
3DU7A	CYS376(CA)	4.49	MET377(C)	3DU7A		C[ ] ... C[ ]
3DU7A	CYS376(CA)	4.65	MET377(O)	3DU7A		
3DU7A	CYS376(CA)	4.43	MET377(CB)	3DU7A		C[ ] ... C[ ]
3DU7A	CYS376(C)	2.44	MET377(CA)	3DU7A		C[ ] ... C[ ]
3DU7A	CYS376(C)	3.01	MET377(C)	3DU7A		C[ ] ... C[ ]
3DU7A	CYS376(C)	3.17	MET377(O)	3DU7A		C[ ] ... O[ ]
3DU7A	CYS376(C)	3.13	MET377(CB)	3DU7A		C[ ] ... C[ ]
3DU7A	CYS376(C)	4.53	MET377(CG)	3DU7A		
3DU7A	CYS376(O)	2.26	MET377(N)	3DU7A	[D-A-AA]:29.6° [A-D-DD]:94.5° d_planarity:4.7° a_planarity:3.0°, bad a_angle(sp2)	O[ ] ... N[ ]
3DU7A	CYS376(O)	2.79	MET377(CA)	3DU7A		O[ ] ... C[ ]

chain1	res1/atm1	distance	res2/atm2	chain2	H-bonding	Charge interaction
3DU7A	CYS376(O)	2.80	MET377(C)	3DU7A		O[ ] ... C[ ]
3DU7A	CYS376(O)	2.47	MET377(O)	3DU7A		O[ ] ... O[ ]
3DU7A	CYS376(O)	3.27	MET377(CB)	3DU7A		O[ ] ... C[ ]
3DU7A	CYS376(O)	4.43	MET377(CG)	3DU7A		O[ ] ... C[ ]
3DU7A	CYS376(CB)	3.20	MET377(N)	3DU7A		C[ ] ... N[ ]
3DU7A	CYS376(CB)	4.54	MET377(CA)	3DU7A		
3DU7A	CYS376(CB)	4.93	MET377(C)	3DU7A		
3DU7A	CYS376(SG)	4.13	MET377(N)	3DU7A	[D-A-AA]:47.0° [A-D-DD]:129.9° d_planarity:39.8°, maximum distance exceeded, bad a_angle(sp3)	S[ ] ... N[ ]
3DU7A	CYS376(SG)	4.93	MET377(O)	3DU7A		
3DU7A	CYS376(C)	4.00	LEU378(N)	3DU7A		C[ ] ... N[ ]
3DU7A	CYS376(C)	4.96	LEU378(CD2)	3DU7A		
3DU7A	CYS376(O)	3.94	LEU378(N)	3DU7A	[D-A-AA]:84.1° [A-D-DD]:121.3° d_planarity:30.1° a_planarity:28.3°, maximum distance exceeded, bad a_angle(sp2)	O[ ] ... N[ ]
3DU7A	CYS376(O)	4.84	LEU378(CA)	3DU7A		
3DU7A	CYS376(O)	4.90	LEU378(CG)	3DU7A		
3DU7A	CYS376(O)	4.46	LEU378(CD2)	3DU7A		O[ ] ... C[ ]
3DU7A	CYS376(SG)	4.55	LEU378(CD2)	3DU7A		

chain1	res1/atm1	distance	res2/atm2	chain2	H-bonding	Charge interaction
1CTSA	CYS184(SG)	4.83	ALA118(C)	1CTSA		
1CTSA	CYS184(SG)	4.97	ALA118(O)	1CTSA		
1CTSA	CYS184(SG)	4.54	ALA118(CB)	1CTSA		
1CTSA	CYS184(O)	4.78	LEU120(CD2)	1CTSA		
1CTSA	CYS184(CB)	4.73	LEU120(CG)	1CTSA		
1CTSA	CYS184(CB)	3.94	LEU120(CD1)	1CTSA		C[ ] ... C[ ]
1CTSA	CYS184(CB)	4.47	LEU120(CD2)	1CTSA		C[ ] ... C[ ]
1CTSA	CYS184(SG)	4.63	LEU120(CD1)	1CTSA		
1CTSA	CYS184(O)	4.98	LEU128(CD1)	1CTSA		
1CTSA	CYS184(N)	4.73	ALA180(O)	1CTSA	[D-A-AA]:118.4° [A-D-DD]:116.1° d_planarity:-8.2° a_planarity:47.9°, maximum distance exceeded	
1CTSA	CYS184(SG)	4.04	ALA180(O)	1CTSA		S[ ] ... O[ ]
1CTSA	CYS184(N)	4.73	LYS181(CA)	1CTSA		
1CTSA	CYS184(N)	3.75	LYS181(C)	1CTSA		N[ ] ... C[ ]
1CTSA	CYS184(N)	3.09	LYS181(O)	1CTSA	**H-bond** [D-A-AA]:112.3° [A-D-DD]:90.8° d_planarity:-46.2° a_planarity:72.7°	N[ ] ... O[ ]
1CTSA	CYS184(CA)	4.41	LYS181(C)	1CTSA		C[ ] ... C[ ]
1CTSA	CYS184(CA)	3.44	LYS181(O)	1CTSA		C[ ] ... O[ ]
1CTSA	CYS184(C)	4.81	LYS181(C)	1CTSA		
1CTSA	CYS184(C)	3.65	LYS181(O)	1CTSA		C[ ] ... O[ ]
1CTSA	CYS184(O)	4.77	LYS181(O)	1CTSA		
1CTSA	CYS184(CB)	4.61	LYS181(CA)	1CTSA		
1CTSA	CYS184(CB)	4.15	LYS181(C)	1CTSA		C[ ] ... C[ ]
1CTSA	CYS184(CB)	3.20	LYS181(O)	1CTSA		C[ ] ... O[ ]
1CTSA	CYS184(SG)	4.45	LYS181(CA)	1CTSA	Weakly positive	S[ ] ... C[ ]

1CTSA	CYS184(SG)	4.49	LYS181(C)	1CTSA		S[ ] ... C[ ]
1CTSA	CYS184(SG)	3.98	LYS181(O)	1CTSA		S[ ] ... O[ ]
1CTSA	CYS184(N)	4.30	LEU182(N)	1CTSA		N[ ] ... N[ ]
1CTSA	CYS184(N)	4.29	LEU182(CA)	1CTSA		N[ ] ... C[ ]
1CTSA	CYS184(N)	3.18	LEU182(C)	1CTSA		N[ ] ... C[ ]
1CTSA	CYS184(N)	3.20	LEU182(O)	1CTSA	[D-A-AA]:77.7° [A-D-DD]:115.8° d_planarity:71.6° a_planarity:52.6°, bad a_angle(sp2), bad d_planarity	N[ ] ... O[ ]
1CTSA	CYS184(CA)	4.33	LEU182(C)	1CTSA		C[ ] ... C[ ]
1CTSA	CYS184(CA)	4.06	LEU182(O)	1CTSA		C[ ] ... O[ ]
1CTSA	CYS184(C)	4.42	LEU182(C)	1CTSA		C[ ] ... C[ ]
1CTSA	CYS184(C)	3.75	LEU182(O)	1CTSA		C[ ] ... O[ ]
1CTSA	CYS184(O)	4.82	LEU182(O)	1CTSA		
1CTSA	CYS184(N)	2.84	PRO183(N)	1CTSA		N[ ] ... N[ ]
1CTSA	CYS184(N)	2.41	PRO183(CA)	1CTSA		N[ ] ... C[ ]
1CTSA	CYS184(N)	2.28	PRO183(O)	1CTSA	[D-A-AA]:27.7° [A-D-DD]:96.3° d_planarity:2.3° a_planarity:5.3°, bad a_angle(sp2)	N[ ] ... O[ ]
1CTSA	CYS184(N)	3.00	PRO183(CB)	1CTSA		N[ ] ... C[ ]
1CTSA	CYS184(N)	3.82	PRO183(CG)	1CTSA		N[ ] ... C[ ]
1CTSA	CYS184(N)	3.72	PRO183(CD)	1CTSA		N[ ] ... C[ ]
1CTSA	CYS184(CA)	4.24	PRO183(N)	1CTSA		C[ ] ... N[ ]
1CTSA	CYS184(CA)	3.80	PRO183(CA)	1CTSA		C[ ] ... C[ ]
1CTSA	CYS184(CA)	2.44	PRO183(C)	1CTSA		C[ ] ... C[ ]
1CTSA	CYS184(CA)	2.84	PRO183(O)	1CTSA		C[ ] ... O[ ]
1CTSA	CYS184(CA)	4.34	PRO183(CB)	1CTSA		C[ ] ... C[ ]
1CTSA	CYS184(C)	4.74	PRO183(N)	1CTSA		
1CTSA	CYS184(C)	4.42	PRO183(CA)	1CTSA		C[ ] ... C[ ]
1CTSA	CYS184(C)	3.03	PRO183(C)	1CTSA		C[ ] ... C[ ]
1CTSA	CYS184(C)	3.00	PRO183(O)	1CTSA		C[ ] ... O[ ]
1CTSA	CYS184(O)	4.03	PRO183(C)	1CTSA		O[ ] ... C[ ]
1CTSA	CYS184(O)	3.72	PRO183(O)	1CTSA		O[ ] ... O[ ]
1CTSA	CYS184(CB)	4.89	PRO183(CA)	1CTSA		
1CTSA	CYS184(CB)	3.74	PRO183(C)	1CTSA		C[ ] ... C[ ]
1CTSA	CYS184(CB)	4.35	PRO183(O)	1CTSA		C[ ] ... O[ ]
1CTSA	CYS184(SG)	4.48	PRO183(C)	1CTSA		S[ ] ... C[ ]
1CTSA	CYS184(N)	2.80	VAL185(N)	1CTSA		N[ ] ... N[ ]
1CTSA	CYS184(N)	4.24	VAL185(CA)	1CTSA		N[ ] ... C[ ]
1CTSA	CYS184(N)	4.84	VAL185(C)	1CTSA		
1CTSA	CYS184(CA)	2.40	VAL185(N)	1CTSA		C[ ] ... N[ ]
1CTSA	CYS184(CA)	3.75	VAL185(CA)	1CTSA		C[ ] ... C[ ]
1CTSA	CYS184(CA)	4.44	VAL185(C)	1CTSA		C[ ] ... C[ ]
1CTSA	CYS184(CA)	4.84	VAL185(CB)	1CTSA		
1CTSA	CYS184(C)	2.36	VAL185(CA)	1CTSA		C[ ] ... C[ ]
1CTSA	CYS184(C)	3.01	VAL185(C)	1CTSA		C[ ] ... C[ ]
1CTSA	CYS184(C)	3.80	VAL185(O)	1CTSA		C[ ] ... O[ ]
1CTSA	CYS184(C)	3.67	VAL185(CB)	1CTSA		C[ ] ... C[ ]
1CTSA	CYS184(C)	4.94	VAL185(CG1)	1CTSA		
1CTSA	CYS184(C)	4.13	VAL185(CG2)	1CTSA		C[ ] ... C[ ]
1CTSA	CYS184(O)	2.22	VAL185(N)	1CTSA	[D-A-AA]:29.6° [A-D-DD]:89.4° d_planarity:1.4° a_planarity:13.1°, bad a_angle(sp2), bad d_angle(sp2)	O[ ] ... N[ ]
1CTSA	CYS184(O)	2.65	VAL185(CA)	1CTSA		O[ ] ... C[ ]

1CTSA	CYS184(O)	3.02	VAL185(C)	1CTSA		O[ ] ... C[ ]
1CTSA	CYS184(O)	3.40	VAL185(O)	1CTSA		O[ ] ... O[ ]
1CTSA	CYS184(O)	4.14	VAL185(CB)	1CTSA		O[ ] ... C[ ]
1CTSA	CYS184(O)	4.66	VAL185(CG2)	1CTSA		
1CTSA	CYS184(CB)	3.14	VAL185(N)	1CTSA		C[ ] ... N[ ]
1CTSA	CYS184(CB)	4.35	VAL185(CA)	1CTSA		C[ ] ... C[ ]
1CTSA	CYS184(CB)	4.93	VAL185(CG2)	1CTSA		
1CTSA	CYS184(SG)	4.81	VAL185(N)	1CTSA	[D-A-AA]:18.3° [A-D-DD]:148.1° d_planarity:66.6°, maximum distance exceeded, bad a_angle(sp3), bad d_planarity	
1CTSA	CYS184(N)	4.56	ALA186(N)	1CTSA		
1CTSA	CYS184(CA)	4.54	ALA186(N)	1CTSA		
1CTSA	CYS184(C)	3.36	ALA186(N)	1CTSA		C[ ] ... N[ ]
1CTSA	CYS184(C)	4.60	ALA186(CA)	1CTSA		
1CTSA	CYS184(C)	4.74	ALA186(C)	1CTSA		
1CTSA	CYS184(O)	3.66	ALA186(N)	1CTSA	[D-A-AA]:66.5° [A-D-DD]:130.2° d_planarity:66.2° a_planarity:34.3°, maximum distance exceeded, bad a_angle(sp2), bad d_planarity	O[ ] ... N[ ]
1CTSA	CYS184(O)	4.74	ALA186(CA)	1CTSA		
1CTSA	CYS184(O)	4.62	ALA186(C)	1CTSA		
1CTSA	CYS184(N)	4.86	ALA187(N)	1CTSA		
1CTSA	CYS184(N)	4.70	ALA187(CB)	1CTSA		
1CTSA	CYS184(CA)	4.75	ALA187(N)	1CTSA		
1CTSA	CYS184(CA)	4.39	ALA187(CB)	1CTSA		C[ ] ... C[ ]
1CTSA	CYS184(C)	4.03	ALA187(N)	1CTSA		C[ ] ... N[ ]
1CTSA	CYS184(C)	4.67	ALA187(CA)	1CTSA		
1CTSA	CYS184(C)	4.95	ALA187(C)	1CTSA		
1CTSA	CYS184(C)	4.31	ALA187(CB)	1CTSA		C[ ] ... C[ ]
1CTSA	CYS184(O)	3.95	ALA187(N)	1CTSA	[D-A-AA]:84.8° [A-D-DD]:95.6° d_planarity:-58.2° a_planarity:75.0°, maximum distance exceeded, bad a_angle(sp2)	O[ ] ... N[ ]
1CTSA	CYS184(O)	4.35	ALA187(CA)	1CTSA		O[ ] ... C[ ]
1CTSA	CYS184(O)	4.26	ALA187(C)	1CTSA		O[ ] ... C[ ]
1CTSA	CYS184(O)	4.10	ALA187(CB)	1CTSA		O[ ] ... C[ ]
1CTSA	CYS184(C)	4.30	LYS188(N)	1CTSA		C[ ] ... N[ ]
1CTSA	CYS184(C)	4.36	LYS188(CB)	1CTSA		C[ ] ... C[ ]
1CTSA	CYS184(O)	3.46	LYS188(N)	1CTSA	**H-bond** [D-A-AA]:125.7° [A-D-DD]:100.2° d_planarity:-44.6° a_planarity:72.3°	O[ ] ... N[ ]
1CTSA	CYS184(O)	3.97	LYS188(CA)	1CTSA		O[ ] ... C[ ]
1CTSA	CYS184(O)	3.17	LYS188(CB)	1CTSA		O[ ] ... C[ ]
1CTSA	CYS184(O)	4.35	LYS188(CG)	1CTSA		O[ ] ... C[ ]
1CTSA	CYS184(O)	4.30	LYS188(CD)	1CTSA		O[ ] ... C[ ]
1CTSA	CYS184(CA)	4.39	ILE200(CD1)	1CTSA		C[ ] ... C[ ]
1CTSA	CYS184(C)	4.78	ILE200(CD1)	1CTSA		
1CTSA	CYS184(O)	4.53	ILE200(CG2)	1CTSA		
1CTSA	CYS184(O)	4.16	ILE200(CD1)	1CTSA		O[ ] ... C[ ]
1CTSA	CYS184(CB)	4.41	ILE200(CD1)	1CTSA		C[ ] ... C[ ]
1CTSA	CYS184(SG)	4.90	ILE200(CD1)	1CTSA		
1CTSA	CYS184(SG)	4.86	ILE203(CG1)	1CTSA		
1CTSA	CYS184(SG)	4.23	ILE203(CD1)	1CTSA		S[ ] ... C[ ]
1CTSA	CYS184(N)	4.56	MET216(SD)	1CTSA	[D-A-AA]:138.8° [A-D-DD]:70.2° d_planarity:76.3°,	

					maximum distance exceeded, bad d_angle(sp2), bad d_planarity	
1CTSA	CYS184(N)	4.00	MET216(CE)	1CTSA		N[ ] ... C[ ]
1CTSA	CYS184(CA)	4.29	MET216(SD)	1CTSA		C[ ] ... S[ ]
1CTSA	CYS184(CA)	4.33	MET216(CE)	1CTSA		C[ ] ... C[ ]
1CTSA	CYS184(CB)	4.79	MET216(SD)	1CTSA		
1CTSA	CYS184(CB)	4.94	MET216(CE)	1CTSA		
1CTSA	CYS184(SG)	4.48	MET216(SD)	1CTSA		S[ ] ... S[ ]
1CTSA	CYS184(SG)	4.42	MET216(CE)	1CTSA		S[ ] ... C[ ]

chain1	res1/atm1	distance	res2/atm2	chain2	H-bonding	Charge interaction
3F8UA	CYS244(N)	4.70	GLU216(OE1)	3F8UA	[D-A-AA]:107.1° [A-D-DD]:128.1° d_planarity:-24.1° a_planarity:-33.1°, maximum distance exceeded	
3F8UA	CYS244(N)	4.95	GLU216(OE2)	3F8UA	[D-A-AA]:95.0° [A-D-DD]:153.3° d_planarity:-37.2° a_planarity:-29.9°, maximum distance exceeded	
3F8UA	CYS244(N)	4.79	PHE241(C)	3F8UA		
3F8UA	CYS244(N)	3.99	PHE241(O)	3F8UA	[D-A-AA]:124.2° [A-D-DD]:105.1° d_planarity:-38.6° a_planarity:53.3°, maximum distance exceeded	N[ ] ... O[ ]
3F8UA	CYS244(CA)	4.59	PHE241(O)	3F8UA		
3F8UA	CYS244(C)	4.09	PHE241(O)	3F8UA		C[ ] ... O[ ]
3F8UA	CYS244(O)	4.66	PHE241(O)	3F8UA		
3F8UA	CYS244(N)	4.36	GLY242(CA)	3F8UA		N[ ] ... C[ ]
3F8UA	CYS244(N)	3.25	GLY242(C)	3F8UA		N[ ] ... C[ ]
3F8UA	CYS244(N)	3.34	GLY242(O)	3F8UA	[D-A-AA]:75.0° [A-D-DD]:132.9° d_planarity:-89.5° a_planarity:48.6°, bad a_angle(sp2), bad d_planarity	N[ ] ... O[ ]
3F8UA	CYS244(CA)	4.57	GLY242(C)	3F8UA		
3F8UA	CYS244(CA)	4.47	GLY242(O)	3F8UA		C[ ] ... O[ ]
3F8UA	CYS244(N)	2.82	ILE243(N)	3F8UA		N[ ] ... N[ ]
3F8UA	CYS244(N)	2.49	ILE243(CA)	3F8UA		N[ ] ... C[ ]
3F8UA	CYS244(N)	2.25	ILE243(O)	3F8UA	[D-A-AA]:30.5° [A-D-DD]:93.4° d_planarity:1.7° a_planarity:3.2°, bad a_angle(sp2)	N[ ] ... O[ ]
3F8UA	CYS244(N)	3.51	ILE243(CB)	3F8UA		N[ ] ... C[ ]
3F8UA	CYS244(N)	3.48	ILE243(CG1)	3F8UA		N[ ] ... C[ ]
3F8UA	CYS244(N)	4.80	ILE243(CG2)	3F8UA		
3F8UA	CYS244(N)	4.83	ILE243(CD1)	3F8UA		
3F8UA	CYS244(CA)	4.28	ILE243(N)	3F8UA		C[ ] ... N[ ]
3F8UA	CYS244(CA)	3.86	ILE243(CA)	3F8UA		C[ ] ... C[ ]
3F8UA	CYS244(CA)	2.44	ILE243(C)	3F8UA		C[ ] ... C[ ]
3F8UA	CYS244(CA)	2.75	ILE243(O)	3F8UA		C[ ] ... O[ ]
3F8UA	CYS244(CA)	4.76	ILE243(CB)	3F8UA		
3F8UA	CYS244(CA)	4.56	ILE243(CG1)	3F8UA		
3F8UA	CYS244(C)	4.98	ILE243(CA)	3F8UA		
3F8UA	CYS244(C)	3.75	ILE243(C)	3F8UA		C[ ] ... C[ ]
3F8UA	CYS244(C)	4.23	ILE243(O)	3F8UA		C[ ] ... O[ ]
3F8UA	CYS244(O)	4.60	ILE243(C)	3F8UA		
3F8UA	CYS244(CB)	4.49	ILE243(CA)	3F8UA		C[ ] ... C[ ]
3F8UA	CYS244(CB)	3.10	ILE243(C)	3F8UA		C[ ] ... C[ ]
3F8UA	CYS244(CB)	3.15	ILE243(O)	3F8UA		C[ ] ... O[ ]

3F8UA	CYS244(CB)	4.94	ILE243(CB)	3F8UA		
3F8UA	CYS244(CB)	4.39	ILE243(CG1)	3F8UA		C[ ] ... C[ ]
3F8UA	CYS244(SG)	4.78	ILE243(C)	3F8UA		
3F8UA	CYS244(SG)	4.52	ILE243(O)	3F8UA		
3F8UA	CYS244(N)	3.21	PRO245(N)	3F8UA		N[ ] ... N[ ]
3F8UA	CYS244(N)	4.55	PRO245(CA)	3F8UA		
3F8UA	CYS244(N)	4.88	PRO245(CG)	3F8UA		
3F8UA	CYS244(N)	3.38	PRO245(CD)	3F8UA		N[ ] ... C[ ]
3F8UA	CYS244(CA)	2.45	PRO245(N)	3F8UA		C[ ] ... N[ ]
3F8UA	CYS244(CA)	3.82	PRO245(CA)	3F8UA		C[ ] ... C[ ]
3F8UA	CYS244(CA)	4.54	PRO245(C)	3F8UA		
3F8UA	CYS244(CA)	4.33	PRO245(O)	3F8UA		C[ ] ... O[ ]
3F8UA	CYS244(CA)	4.73	PRO245(CB)	3F8UA		
3F8UA	CYS244(CA)	4.34	PRO245(CG)	3F8UA		C[ ] ... C[ ]
3F8UA	CYS244(CA)	2.95	PRO245(CD)	3F8UA		C[ ] ... C[ ]
3F8UA	CYS244(C)	2.45	PRO245(CA)	3F8UA		C[ ] ... C[ ]
3F8UA	CYS244(C)	3.16	PRO245(C)	3F8UA		C[ ] ... C[ ]
3F8UA	CYS244(C)	3.21	PRO245(O)	3F8UA		C[ ] ... O[ ]
3F8UA	CYS244(C)	3.61	PRO245(CB)	3F8UA		C[ ] ... C[ ]
3F8UA	CYS244(C)	3.62	PRO245(CG)	3F8UA		C[ ] ... C[ ]
3F8UA	CYS244(C)	2.52	PRO245(CD)	3F8UA		C[ ] ... C[ ]
3F8UA	CYS244(O)	2.25	PRO245(N)	3F8UA		O[ ] ... N[ ]
3F8UA	CYS244(O)	2.76	PRO245(CA)	3F8UA		O[ ] ... C[ ]
3F8UA	CYS244(O)	3.17	PRO245(C)	3F8UA		O[ ] ... C[ ]
3F8UA	CYS244(O)	3.31	PRO245(O)	3F8UA		O[ ] ... O[ ]
3F8UA	CYS244(O)	4.18	PRO245(CB)	3F8UA		O[ ] ... C[ ]
3F8UA	CYS244(O)	4.52	PRO245(CG)	3F8UA		
3F8UA	CYS244(O)	3.64	PRO245(CD)	3F8UA		O[ ] ... C[ ]
3F8UA	CYS244(CB)	3.68	PRO245(N)	3F8UA		C[ ] ... N[ ]
3F8UA	CYS244(CB)	4.89	PRO245(CA)	3F8UA		
3F8UA	CYS244(CB)	4.88	PRO245(O)	3F8UA		
3F8UA	CYS244(CB)	4.39	PRO245(CD)	3F8UA		C[ ] ... C[ ]
3F8UA	CYS244(SG)	4.25	PRO245(N)	3F8UA		S[ ] ... N[ ]
3F8UA	CYS244(SG)	4.30	PRO245(O)	3F8UA		S[ ] ... O[ ]
3F8UA	CYS244(C)	4.30	HIS246(N)	3F8UA		C[ ] ... N[ ]
3F8UA	CYS244(O)	4.06	HIS246(N)	3F8UA	[D-A-AA]:92.7° [A-D-DD]:128.4° d_planarity:50.2° a_planarity:19.2°, maximum distance exceeded	O[ ] ... N[ ]
3F8UA	CYS244(N)	4.98	ARG280(NH2)	3F8UA		
3F8UA	CYS244(CA)	4.89	ARG280(NE)	3F8UA		
3F8UA	CYS244(CA)	4.85	ARG280(NH2)	3F8UA		
3F8UA	CYS244(C)	3.98	ARG280(NE)	3F8UA		C[ ] ... N[ ]
3F8UA	CYS244(C)	4.48	ARG280(CZ)	3F8UA		C[ ] ... C[ ]
3F8UA	CYS244(C)	4.02	ARG280(NH2)	3F8UA		C[ ] ... N[+]
3F8UA	CYS244(O)	4.51	ARG280(CG)	3F8UA		
3F8UA	CYS244(O)	3.87	ARG280(CD)	3F8UA		O[ ] ... C[ ]
3F8UA	CYS244(O)	2.74	ARG280(NE)	3F8UA	**H-bond** [D-A-AA]:176.2° [A-D-DD]:103.6° d_planarity:-10.4° a_planarity:-0.4°	O[ ] ... N[ ]
3F8UA	CYS244(O)	3.32	ARG280(CZ)	3F8UA		O[ ] ... C[ ]
3F8UA	CYS244(O)	4.63	ARG280(NH1)	3F8UA	[D-A-AA]:157.6° [A-D-DD]:7.5° d_planarity:-50.9° a_planarity:-70.5°, maximum distance exceeded,	

					bad d_angle(sp2)	
3F8UA	CYS244(O)	3.07	ARG280(NH2)	3F8UA	[D-A-AA]:133.6° [A-D-DD]:89.0° d_planarity:-8.7° a_planarity:-87.6°, bad d_angle(sp2)	O[ ] ... N[+]
3F8UA	CYS244(CB)	4.52	ARG280(NE)	3F8UA		
3F8UA	CYS244(CB)	4.90	ARG280(NH2)	3F8UA		
3F8UA	CYS244(SG)	4.54	ARG280(CG)	3F8UA		
3F8UA	CYS244(SG)	4.83	ARG280(CD)	3F8UA	Positive	
3F8UA	CYS244(SG)	4.56	ARG280(NE)	3F8UA	[D-A-AA]:77.1° [A-D-DD]:129.7° d_planarity:-52.4°, maximum distance exceeded	
3F8UA	CYS244(SG)	4.73	VAL283(CB)	3F8UA		
3F8UA	CYS244(SG)	4.13	VAL283(CG1)	3F8UA		S[ ] ... C[ ]
3F8UA	CYS244(N)	4.93	MET284(CG)	3F8UA		
3F8UA	CYS244(N)	3.98	MET284(CE)	3F8UA		N[ ] ... C[ ]
3F8UA	CYS244(CA)	4.74	MET284(CG)	3F8UA		
3F8UA	CYS244(CA)	4.39	MET284(CE)	3F8UA		C[ ] ... C[ ]
3F8UA	CYS244(C)	4.67	MET284(CE)	3F8UA		
3F8UA	CYS244(O)	4.85	MET284(CG)	3F8UA		
3F8UA	CYS244(O)	4.58	MET284(SD)	3F8UA		
3F8UA	CYS244(O)	4.23	MET284(CE)	3F8UA		O[ ] ... C[ ]
3F8UA	CYS244(CB)	4.96	MET284(CA)	3F8UA		
3F8UA	CYS244(CB)	4.65	MET284(CB)	3F8UA		
3F8UA	CYS244(CB)	3.45	MET284(CG)	3F8UA		C[ ] ... C[ ]
3F8UA	CYS244(CB)	4.22	MET284(SD)	3F8UA		C[ ] ... S[ ]
3F8UA	CYS244(CB)	3.93	MET284(CE)	3F8UA		C[ ] ... C[ ]
3F8UA	CYS244(SG)	4.11	MET284(CG)	3F8UA		S[ ] ... C[ ]
3F8UA	CYS244(N)	4.45	PHE299(O)	3F8UA	[D-A-AA]:113.2° [A-D-DD]:47.5° d_planarity:51.4° a_planarity:-79.2°, maximum distance exceeded, bad d_angle(sp2)	N[ ] ... O[ ]
3F8UA	CYS244(N)	4.99	PHE299(CB)	3F8UA		
3F8UA	CYS244(CA)	4.83	PHE299(CA)	3F8UA		
3F8UA	CYS244(CA)	4.11	PHE299(C)	3F8UA		C[ ] ... C[ ]
3F8UA	CYS244(CA)	3.62	PHE299(O)	3F8UA		C[ ] ... O[ ]
3F8UA	CYS244(CA)	4.39	PHE299(CB)	3F8UA		C[ ] ... C[ ]
3F8UA	CYS244(C)	4.75	PHE299(C)	3F8UA		
3F8UA	CYS244(C)	4.13	PHE299(O)	3F8UA		C[ ] ... O[ ]
3F8UA	CYS244(CB)	4.97	PHE299(CA)	3F8UA		
3F8UA	CYS244(CB)	4.45	PHE299(C)	3F8UA		C[ ] ... C[ ]
3F8UA	CYS244(CB)	4.38	PHE299(O)	3F8UA		C[ ] ... O[ ]
3F8UA	CYS244(CB)	4.14	PHE299(CB)	3F8UA		C[ ] ... C[ ]
3F8UA	CYS244(SG)	4.75	PHE299(CA)	3F8UA		
3F8UA	CYS244(SG)	4.07	PHE299(C)	3F8UA		S[ ] ... C[ ]
3F8UA	CYS244(SG)	4.36	PHE299(O)	3F8UA		S[ ] ... O[ ]
3F8UA	CYS244(SG)	4.06	PHE299(CB)	3F8UA		S[ ] ... C[ ]
3F8UA	CYS244(CA)	4.59	ALA300(N)	3F8UA		
3F8UA	CYS244(CA)	4.61	ALA300(CA)	3F8UA		
3F8UA	CYS244(C)	4.97	ALA300(N)	3F8UA		
3F8UA	CYS244(C)	4.53	ALA300(CA)	3F8UA		
3F8UA	CYS244(CB)	4.67	ALA300(N)	3F8UA		
3F8UA	CYS244(CB)	4.81	ALA300(CA)	3F8UA		
3F8UA	CYS244(SG)	3.75	ALA300(N)	3F8UA	[D-A-AA]:108.1° [A-D-DD]:78.6° d_planarity:88.1°,	S[ ] ... N[ ]

					bad d_angle(sp2), bad d_planarity	
3F8UA	CYS244(SG)	3.75	ALA300(CA)	3F8UA		S[ ] ... C[ ]
3F8UA	CYS244(SG)	3.94	ALA300(C)	3F8UA		S[ ] ... C[ ]
3F8UA	CYS244(SG)	4.53	ALA300(O)	3F8UA		
3F8UA	CYS244(SG)	4.03	VAL301(N)	3F8UA	[D-A-AA]:138.5° [A-D-DD]:120.2° d_planarity:-88.1°, maximum distance exceeded, bad d_planarity	S[ ] ... N[ ]
3F8UA	CYS244(SG)	4.92	VAL301(CA)	3F8UA		
3F8UA	CYS244(SG)	4.77	VAL301(CB)	3F8UA		
3F8UA	CYS244(SG)	3.75	VAL301(CG1)	3F8UA		S[ ] ... C[ ]

chain1	res1/atm1	distance	res2/atm2	chain2	H-bonding	Charge interaction
3CF1A	CYS105(N)	4.13	ASP55(OD1)	3CF1A	[D-A-AA]:140.9° [A-D-DD]:138.1° d_planarity:-30.3° a_planarity:-25.3°, maximum distance exceeded	N[ ] ... O[-]
3CF1A	CYS105(N)	4.43	THR56(C)	3CF1A		N[ ] ... C[ ]
3CF1A	CYS105(N)	3.36	THR56(O)	3CF1A	**H-bond** [D-A-AA]:145.6° [A-D-DD]:115.9° d_planarity:-13.0° a_planarity:53.5°	N[ ] ... O[ ]
3CF1A	CYS105(N)	4.97	THR56(OG1)	3CF1A	[D-A-AA]:121.5° [A-D-DD]:79.3° d_planarity:-3.3°, maximum distance exceeded, bad d_angle(sp2)	
3CF1A	CYS105(CA)	4.20	THR56(O)	3CF1A		C[ ] ... O[ ]
3CF1A	CYS105(CA)	4.91	THR56(OG1)	3CF1A		
3CF1A	CYS105(C)	4.19	THR56(O)	3CF1A		C[ ] ... O[ ]
3CF1A	CYS105(C)	4.04	THR56(OG1)	3CF1A		C[ ] ... O[ ]
3CF1A	CYS105(O)	4.90	THR56(C)	3CF1A		
3CF1A	CYS105(O)	4.30	THR56(O)	3CF1A		O[ ] ... O[ ]
3CF1A	CYS105(O)	4.66	THR56(CB)	3CF1A		
3CF1A	CYS105(O)	3.34	THR56(OG1)	3CF1A	[D-A-AA]:116.5° [A-D-DD]:153.4° a_planarity:81.0°, maximum distance exceeded	O[ ] ... O[ ]
3CF1A	CYS105(CB)	4.50	THR56(O)	3CF1A		
3CF1A	CYS105(N)	4.96	VAL57(N)	3CF1A		
3CF1A	CYS105(N)	4.55	VAL57(CA)	3CF1A		
3CF1A	CYS105(N)	4.97	VAL57(C)	3CF1A		
3CF1A	CYS105(CB)	4.97	VAL57(CA)	3CF1A		
3CF1A	CYS105(CB)	4.69	VAL57(C)	3CF1A		
3CF1A	CYS105(N)	4.76	LEU58(N)	3CF1A		
3CF1A	CYS105(CB)	4.40	LEU58(N)	3CF1A		C[ ] ... N[ ]
3CF1A	CYS105(CB)	4.76	LEU58(CA)	3CF1A		
3CF1A	CYS105(CB)	4.02	LEU58(CB)	3CF1A		C[ ] ... C[ ]
3CF1A	CYS105(CB)	4.98	LEU58(CG)	3CF1A		
3CF1A	CYS105(CB)	4.89	LEU58(CD2)	3CF1A		
3CF1A	CYS105(SG)	4.61	LEU58(CB)	3CF1A		
3CF1A	CYS105(SG)	4.63	LEU58(CD2)	3CF1A		
3CF1A	CYS105(N)	3.84	GLN103(C)	3CF1A		N[ ] ... C[ ]
3CF1A	CYS105(N)	3.40	GLN103(O)	3CF1A	[D-A-AA]:101.7° [A-D-DD]:123.1° d_planarity:72.7° a_planarity:23.3°, bad d_planarity	N[ ] ... O[ ]
3CF1A	CYS105(CA)	4.91	GLN103(C)	3CF1A		
3CF1A	CYS105(CA)	4.36	GLN103(O)	3CF1A		C[ ] ... O[ ]
3CF1A	CYS105(CB)	4.91	GLN103(C)	3CF1A		
3CF1A	CYS105(CB)	4.13	GLN103(O)	3CF1A		C[ ] ... O[ ]

3CF1A	CYS105(N)	3.51	PRO104(N)	3CF1A		N[ ] ... N[ ]
3CF1A	CYS105(N)	2.42	PRO104(CA)	3CF1A		N[ ] ... C[ ]
3CF1A	CYS105(N)	2.24	PRO104(O)	3CF1A	[D-A-AA]:29.8° [A-D-DD]:92.9° d_planarity:0.0° a_planarity:0.5°, bad a_angle(sp2)	N[ ] ... O[ ]
3CF1A	CYS105(N)	3.45	PRO104(CB)	3CF1A		N[ ] ... C[ ]
3CF1A	CYS105(N)	4.28	PRO104(CG)	3CF1A		N[ ] ... C[ ]
3CF1A	CYS105(N)	4.60	PRO104(CD)	3CF1A		
3CF1A	CYS105(CA)	4.72	PRO104(N)	3CF1A		
3CF1A	CYS105(CA)	3.78	PRO104(CA)	3CF1A		C[ ] ... C[ ]
3CF1A	CYS105(CA)	2.41	PRO104(C)	3CF1A		C[ ] ... C[ ]
3CF1A	CYS105(CA)	2.73	PRO104(O)	3CF1A		C[ ] ... O[ ]
3CF1A	CYS105(CA)	4.71	PRO104(CB)	3CF1A		
3CF1A	CYS105(C)	4.80	PRO104(CA)	3CF1A		
3CF1A	CYS105(C)	3.62	PRO104(C)	3CF1A		C[ ] ... C[ ]
3CF1A	CYS105(C)	4.08	PRO104(O)	3CF1A		C[ ] ... O[ ]
3CF1A	CYS105(O)	4.61	PRO104(C)	3CF1A		
3CF1A	CYS105(CB)	4.53	PRO104(CA)	3CF1A		
3CF1A	CYS105(CB)	3.25	PRO104(C)	3CF1A		C[ ] ... C[ ]
3CF1A	CYS105(CB)	3.45	PRO104(O)	3CF1A		C[ ] ... O[ ]
3CF1A	CYS105(SG)	4.78	PRO104(C)	3CF1A		
3CF1A	CYS105(SG)	4.64	PRO104(O)	3CF1A		
3CF1A	CYS105(N)	3.01	PRO106(N)	3CF1A		N[ ] ... N[ ]
3CF1A	CYS105(N)	4.38	PRO106(CA)	3CF1A		N[ ] ... C[ ]
3CF1A	CYS105(N)	4.92	PRO106(CB)	3CF1A		
3CF1A	CYS105(N)	4.49	PRO106(CG)	3CF1A		N[ ] ... C[ ]
3CF1A	CYS105(N)	2.99	PRO106(CD)	3CF1A		N[ ] ... C[ ]
3CF1A	CYS105(CA)	2.46	PRO106(N)	3CF1A		C[ ] ... N[ ]
3CF1A	CYS105(CA)	3.85	PRO106(CA)	3CF1A		C[ ] ... C[ ]
3CF1A	CYS105(CA)	4.64	PRO106(C)	3CF1A		
3CF1A	CYS105(CA)	4.74	PRO106(CB)	3CF1A		
3CF1A	CYS105(CA)	4.37	PRO106(CG)	3CF1A		C[ ] ... C[ ]
3CF1A	CYS105(CA)	2.93	PRO106(CD)	3CF1A		C[ ] ... C[ ]
3CF1A	CYS105(C)	2.47	PRO106(CA)	3CF1A		C[ ] ... C[ ]
3CF1A	CYS105(C)	3.28	PRO106(C)	3CF1A		C[ ] ... C[ ]
3CF1A	CYS105(C)	4.48	PRO106(O)	3CF1A		C[ ] ... O[ ]
3CF1A	CYS105(C)	3.62	PRO106(CB)	3CF1A		C[ ] ... C[ ]
3CF1A	CYS105(C)	3.65	PRO106(CG)	3CF1A		C[ ] ... C[ ]
3CF1A	CYS105(C)	2.52	PRO106(CD)	3CF1A		C[ ] ... C[ ]
3CF1A	CYS105(O)	2.26	PRO106(N)	3CF1A		O[ ] ... N[ ]
3CF1A	CYS105(O)	2.80	PRO106(CA)	3CF1A		O[ ] ... C[ ]
3CF1A	CYS105(O)	3.33	PRO106(C)	3CF1A		O[ ] ... C[ ]
3CF1A	CYS105(O)	4.54	PRO106(O)	3CF1A		
3CF1A	CYS105(O)	4.20	PRO106(CB)	3CF1A		O[ ] ... C[ ]
3CF1A	CYS105(O)	4.56	PRO106(CG)	3CF1A		
3CF1A	CYS105(O)	3.63	PRO106(CD)	3CF1A		O[ ] ... C[ ]
3CF1A	CYS105(CB)	3.75	PRO106(N)	3CF1A		C[ ] ... N[ ]
3CF1A	CYS105(CB)	4.98	PRO106(CA)	3CF1A		
3CF1A	CYS105(CB)	4.44	PRO106(CD)	3CF1A		C[ ] ... C[ ]
3CF1A	CYS105(SG)	4.52	PRO106(N)	3CF1A		

3CF1A	CYS105(CA)	4.35	ASP107(N)	3CF1A		C[ ] ... N[ ]
3CF1A	CYS105(CA)	3.95	ASP107(OD1)	3CF1A		C[ ] ... O[-]
3CF1A	CYS105(C)	3.11	ASP107(N)	3CF1A		C[ ] ... N[ ]
3CF1A	CYS105(C)	4.46	ASP107(CA)	3CF1A		C[ ] ... C[ ]
3CF1A	CYS105(C)	4.92	ASP107(CB)	3CF1A		
3CF1A	CYS105(C)	4.69	ASP107(CG)	3CF1A		
3CF1A	CYS105(C)	3.69	ASP107(OD1)	3CF1A		C[ ] ... O[-]
3CF1A	CYS105(O)	2.95	ASP107(N)	3CF1A	[D-A-AA]:85.5° [A-D-DD]:140.6° d_planarity:-24.9° a_planarity:46.4°, bad a_angle(sp2)	O[ ] ... N[ ]
3CF1A	CYS105(O)	4.19	ASP107(CA)	3CF1A		O[ ] ... C[ ]
3CF1A	CYS105(O)	4.41	ASP107(C)	3CF1A		O[ ] ... C[ ]
3CF1A	CYS105(O)	4.88	ASP107(CB)	3CF1A		
3CF1A	CYS105(O)	4.60	ASP107(CG)	3CF1A		
3CF1A	CYS105(O)	3.71	ASP107(OD1)	3CF1A		O[ ] ... O[-]
3CF1A	CYS105(CB)	4.00	ASP107(OD1)	3CF1A		C[ ] ... O[-]
3CF1A	CYS105(SG)	4.70	ASP107(N)	3CF1A	[D-A-AA]:89.0° [A-D-DD]:107.1° d_planarity:-8.6°, maximum distance exceeded	
3CF1A	CYS105(SG)	3.98	ASP107(CG)	3CF1A		S[ ] ... C[ ]
3CF1A	CYS105(SG)	2.84	ASP107(OD1)	3CF1A	Negative	S[ ] ... O[-]
3CF1A	CYS105(SG)	4.49	ASP107(OD2)	3CF1A		S[ ] ... O[-]
3CF1A	CYS105(C)	4.52	VAL108(N)	3CF1A		
3CF1A	CYS105(C)	4.51	VAL108(CG2)	3CF1A		
3CF1A	CYS105(O)	3.64	VAL108(N)	3CF1A	[D-A-AA]:129.3° [A-D-DD]:117.4° d_planarity:-21.1° a_planarity:61.4°, maximum distance exceeded	O[ ] ... N[ ]
3CF1A	CYS105(O)	4.50	VAL108(CA)	3CF1A		O[ ] ... C[ ]
3CF1A	CYS105(O)	4.11	VAL108(CB)	3CF1A		O[ ] ... C[ ]
3CF1A	CYS105(O)	3.41	VAL108(CG2)	3CF1A		O[ ] ... C[ ]
3CF1A	CYS105(CB)	4.32	VAL108(CG2)	3CF1A		C[ ] ... C[ ]
3CF1A	CYS105(SG)	4.83	VAL108(N)	3CF1A	[D-A-AA]:106.0° [A-D-DD]:103.5° d_planarity:-63.7°, maximum distance exceeded, bad d_planarity	
3CF1A	CYS105(SG)	4.99	VAL108(CB)	3CF1A		
3CF1A	CYS105(SG)	3.59	VAL108(CG2)	3CF1A		S[ ] ... C[ ]
3CF1A	CYS105(SG)	4.31	TYR173(CE1)	3CF1A		S[ ] ... C[ ]
3CF1A	CYS105(SG)	4.67	TYR173(CE2)	3CF1A		
3CF1A	CYS105(SG)	4.08	TYR173(CZ)	3CF1A		S[ ] ... C[ ]
3CF1A	CYS105(SG)	3.89	TYR173(OH)	3CF1A	[D-A-AA]:155.7° [A-D-DD]:87.6° d_planarity:-80.3°, maximum distance exceeded, bad d_angle(sp2), bad d_planarity	S[ ] ... O[ ]

chain1	res1/atm1	distance	res2/atm2	chain2	H-bonding	Charge interaction
2VL2A	CYS47(CA)	4.79	VAL39(CB)	2VL2A		
2VL2A	CYS47(CA)	4.78	VAL39(CG1)	2VL2A		
2VL2A	CYS47(CB)	3.83	VAL39(CB)	2VL2A		C[ ] ... C[ ]
2VL2A	CYS47(CB)	4.20	VAL39(CG1)	2VL2A		C[ ] ... C[ ]
2VL2A	CYS47(CB)	4.26	VAL39(CG2)	2VL2A		C[ ] ... C[ ]
2VL2A	CYS47(SG)	4.31	VAL39(CA)	2VL2A		S[ ] ... C[ ]
2VL2A	CYS47(SG)	4.37	VAL39(C)	2VL2A		S[ ] ... C[ ]
2VL2A	CYS47(SG)	3.59	VAL39(CB)	2VL2A		S[ ] ... C[ ]

2VL2A	CYS47(SG)	4.44	VAL39(CG1)	2VL2A		S[ ] ... C[ ]
2VL2A	CYS47(SG)	4.23	VAL39(CG2)	2VL2A		S[ ] ... C[ ]
2VL2A	CYS47(SG)	3.86	PRO40(N)	2VL2A		S[ ] ... N[ ]
2VL2A	CYS47(SG)	4.54	PRO40(CA)	2VL2A		
2VL2A	CYS47(SG)	4.60	PRO40(C)	2VL2A		
2VL2A	CYS47(SG)	4.38	PRO40(CB)	2VL2A		S[ ] ... C[ ]
2VL2A	CYS47(SG)	4.31	PRO40(CG)	2VL2A		S[ ] ... C[ ]
2VL2A	CYS47(SG)	3.55	PRO40(CD)	2VL2A		S[ ] ... C[ ]
2VL2A	CYS47(CA)	4.80	GLY41(O)	2VL2A		
2VL2A	CYS47(CB)	4.82	GLY41(N)	2VL2A		
2VL2A	CYS47(CB)	4.44	GLY41(C)	2VL2A		C[ ] ... C[ ]
2VL2A	CYS47(CB)	3.35	GLY41(O)	2VL2A		C[ ] ... O[ ]
2VL2A	CYS47(SG)	3.71	GLY41(N)	2VL2A	**H-bond** [D-A-AA]:117.3° [A-D-DD]:113.2° d_planarity:-15.9°	S[ ] ... N[ ]
2VL2A	CYS47(SG)	4.49	GLY41(CA)	2VL2A		S[ ] ... C[ ]
2VL2A	CYS47(SG)	4.25	GLY41(C)	2VL2A		S[ ] ... C[ ]
2VL2A	CYS47(SG)	3.37	GLY41(O)	2VL2A		S[ ] ... O[ ]
2VL2A	CYS47(N)	4.16	THR44(C)	2VL2A		N[ ] ... C[ ]
2VL2A	CYS47(N)	3.56	THR44(O)	2VL2A	[D-A-AA]:111.0° [A-D-DD]:95.2° d_planarity:-61.2° a_planarity:74.4°, maximum distance exceeded, bad d_planarity	N[ ] ... O[ ]
2VL2A	CYS47(N)	4.92	THR44(CB)	2VL2A		
2VL2A	CYS47(N)	3.76	THR44(OG1)	2VL2A	[D-A-AA]:138.4° [A-D-DD]:95.4° d_planarity:-9.3°, maximum distance exceeded	N[ ] ... O[ ]
2VL2A	CYS47(CA)	4.80	THR44(C)	2VL2A		
2VL2A	CYS47(CA)	3.97	THR44(O)	2VL2A		C[ ] ... O[ ]
2VL2A	CYS47(CA)	4.16	THR44(OG1)	2VL2A		C[ ] ... O[ ]
2VL2A	CYS47(C)	4.92	THR44(C)	2VL2A		
2VL2A	CYS47(C)	3.83	THR44(O)	2VL2A		C[ ] ... O[ ]
2VL2A	CYS47(O)	4.95	THR44(O)	2VL2A		
2VL2A	CYS47(CB)	4.79	THR44(N)	2VL2A		
2VL2A	CYS47(CB)	4.93	THR44(CA)	2VL2A		
2VL2A	CYS47(CB)	4.63	THR44(C)	2VL2A		
2VL2A	CYS47(CB)	3.96	THR44(O)	2VL2A		C[ ] ... O[ ]
2VL2A	CYS47(CB)	4.63	THR44(CB)	2VL2A		
2VL2A	CYS47(CB)	3.32	THR44(OG1)	2VL2A		C[ ] ... O[ ]
2VL2A	CYS47(SG)	4.98	THR44(C)	2VL2A		
2VL2A	CYS47(SG)	4.74	THR44(O)	2VL2A		
2VL2A	CYS47(SG)	4.16	THR44(CB)	2VL2A		S[ ] ... C[ ]
2VL2A	CYS47(SG)	2.75	THR44(OG1)	2VL2A	**H-bond** [D-A-AA]:91.1° [A-D-DD]:169.5°	S[ ] ... O[ ]
2VL2A	CYS47(SG)	4.73	THR44(CG2)	2VL2A		
2VL2A	CYS47(N)	4.57	PRO45(N)	2VL2A		
2VL2A	CYS47(N)	4.50	PRO45(CA)	2VL2A		N[ ] ... C[ ]
2VL2A	CYS47(N)	3.29	PRO45(C)	2VL2A		N[ ] ... C[ ]
2VL2A	CYS47(N)	3.32	PRO45(O)	2VL2A	[D-A-AA]:77.7° [A-D-DD]:119.2° d_planarity:67.8° a_planarity:44.3°, bad a_angle(sp2), bad d_planarity	N[ ] ... O[ ]
2VL2A	CYS47(CA)	4.45	PRO45(C)	2VL2A		C[ ] ... C[ ]
2VL2A	CYS47(CA)	4.23	PRO45(O)	2VL2A		C[ ] ... O[ ]
2VL2A	CYS47(C)	4.55	PRO45(C)	2VL2A		
2VL2A	CYS47(C)	3.97	PRO45(O)	2VL2A		C[ ] ... O[ ]
2VL2A	CYS47(O)	4.97	PRO45(O)	2VL2A		
2VL2A	CYS47(N)	2.86	GLY46(N)	2VL2A		N[ ] ... N[ ]

2VL2A	CYS47(N)	2.42	GLY46(CA)	2VL2A		N[ ] ... C[ ]
2VL2A	CYS47(N)	2.26	GLY46(O)	2VL2A	[D-A-AA]:29.5° [A-D-DD]:95.6° d_planarity:0.3° a_planarity:0.5°, bad a_angle(sp2)	N[ ] ... O[ ]
2VL2A	CYS47(CA)	4.26	GLY46(N)	2VL2A		C[ ] ... N[ ]
2VL2A	CYS47(CA)	3.81	GLY46(CA)	2VL2A		C[ ] ... C[ ]
2VL2A	CYS47(CA)	2.45	GLY46(C)	2VL2A		C[ ] ... C[ ]
2VL2A	CYS47(CA)	2.81	GLY46(O)	2VL2A		C[ ] ... O[ ]
2VL2A	CYS47(C)	4.78	GLY46(N)	2VL2A		
2VL2A	CYS47(C)	4.47	GLY46(CA)	2VL2A		C[ ] ... C[ ]
2VL2A	CYS47(C)	3.08	GLY46(C)	2VL2A		C[ ] ... C[ ]
2VL2A	CYS47(C)	3.04	GLY46(O)	2VL2A		C[ ] ... O[ ]
2VL2A	CYS47(O)	4.02	GLY46(C)	2VL2A		O[ ] ... C[ ]
2VL2A	CYS47(O)	3.66	GLY46(O)	2VL2A		O[ ] ... O[ ]
2VL2A	CYS47(CB)	5.00	GLY46(N)	2VL2A		
2VL2A	CYS47(CB)	4.83	GLY46(CA)	2VL2A		
2VL2A	CYS47(CB)	3.72	GLY46(C)	2VL2A		C[ ] ... C[ ]
2VL2A	CYS47(CB)	4.29	GLY46(O)	2VL2A		C[ ] ... O[ ]
2VL2A	CYS47(SG)	4.93	GLY46(CA)	2VL2A		
2VL2A	CYS47(SG)	4.22	GLY46(C)	2VL2A		S[ ] ... C[ ]
2VL2A	CYS47(N)	2.79	SER48(N)	2VL2A		N[ ] ... N[ ]
2VL2A	CYS47(N)	4.23	SER48(CA)	2VL2A		N[ ] ... C[ ]
2VL2A	CYS47(N)	4.91	SER48(C)	2VL2A		
2VL2A	CYS47(N)	4.98	SER48(CB)	2VL2A		
2VL2A	CYS47(CA)	2.42	SER48(N)	2VL2A		C[ ] ... N[ ]
2VL2A	CYS47(CA)	3.80	SER48(CA)	2VL2A		C[ ] ... C[ ]
2VL2A	CYS47(CA)	4.69	SER48(C)	2VL2A		
2VL2A	CYS47(CA)	4.75	SER48(CB)	2VL2A		
2VL2A	CYS47(CA)	4.66	SER48(OG)	2VL2A		
2VL2A	CYS47(C)	2.44	SER48(CA)	2VL2A		C[ ] ... C[ ]
2VL2A	CYS47(C)	3.34	SER48(C)	2VL2A		C[ ] ... C[ ]
2VL2A	CYS47(C)	4.27	SER48(O)	2VL2A		C[ ] ... O[ ]
2VL2A	CYS47(C)	3.63	SER48(CB)	2VL2A		C[ ] ... C[ ]
2VL2A	CYS47(C)	3.77	SER48(OG)	2VL2A		C[ ] ... O[ ]
2VL2A	CYS47(O)	2.26	SER48(N)	2VL2A	[D-A-AA]:29.5° [A-D-DD]:94.9° d_planarity:3.5° a_planarity:0.8°, bad a_angle(sp2)	O[ ] ... N[ ]
2VL2A	CYS47(O)	2.79	SER48(CA)	2VL2A		O[ ] ... C[ ]
2VL2A	CYS47(O)	3.50	SER48(C)	2VL2A		O[ ] ... C[ ]
2VL2A	CYS47(O)	4.13	SER48(O)	2VL2A		O[ ] ... O[ ]
2VL2A	CYS47(O)	4.16	SER48(CB)	2VL2A		O[ ] ... C[ ]
2VL2A	CYS47(O)	4.35	SER48(OG)	2VL2A	[D-A-AA]:55.0° [A-D-DD]:72.9° a_planarity:40.5°, maximum distance exceeded, bad a_angle(sp2)	O[ ] ... O[ ]
2VL2A	CYS47(CB)	3.19	SER48(N)	2VL2A		C[ ] ... N[ ]
2VL2A	CYS47(CB)	4.43	SER48(CA)	2VL2A		C[ ] ... C[ ]
2VL2A	CYS47(CB)	4.50	SER48(OG)	2VL2A		
2VL2A	CYS47(SG)	4.73	SER48(N)	2VL2A	[D-A-AA]:25.4° [A-D-DD]:153.0° d_planarity:77.7°, maximum distance exceeded, bad a_angle(sp3), bad d_planarity	
2VL2A	CYS47(N)	4.54	LYS49(N)	2VL2A		
2VL2A	CYS47(CA)	4.69	LYS49(N)	2VL2A		
2VL2A	CYS47(C)	3.61	LYS49(N)	2VL2A		C[ ] ... N[ ]
2VL2A	CYS47(C)	4.94	LYS49(CA)	2VL2A		

2VL2A	CYS47(O)	4.01	LYS49(N)	2VL2A	[D-A-AA]:63.0° [A-D-DD]:139.4° d_planarity:75.2° a_planarity:37.1°, maximum distance exceeded, bad a_angle(sp2), bad d_planarity	O[ ] ... N[ ]
2VL2A	CYS47(C)	4.40	THR50(N)	2VL2A		C[ ] ... N[ ]
2VL2A	CYS47(C)	4.97	THR50(C)	2VL2A		
2VL2A	CYS47(O)	4.36	THR50(N)	2VL2A	[D-A-AA]:83.6° [A-D-DD]:108.9° d_planarity:-31.5° a_planarity:69.2°, maximum distance exceeded, bad a_angle(sp2)	O[ ] ... N[ ]
2VL2A	CYS47(O)	4.46	THR50(C)	2VL2A		O[ ] ... C[ ]
2VL2A	CYS47(N)	4.89	HIS51(N)	2VL2A		
2VL2A	CYS47(N)	4.75	HIS51(CB)	2VL2A		
2VL2A	CYS47(CA)	4.44	HIS51(N)	2VL2A		C[ ] ... N[ ]
2VL2A	CYS47(CA)	4.75	HIS51(CA)	2VL2A		
2VL2A	CYS47(CA)	3.98	HIS51(CB)	2VL2A		C[ ] ... C[ ]
2VL2A	CYS47(C)	4.09	HIS51(N)	2VL2A		C[ ] ... N[ ]
2VL2A	CYS47(C)	4.58	HIS51(CA)	2VL2A		
2VL2A	CYS47(C)	4.87	HIS51(C)	2VL2A		
2VL2A	CYS47(C)	4.26	HIS51(CB)	2VL2A		C[ ] ... C[ ]
2VL2A	CYS47(O)	3.61	HIS51(N)	2VL2A	[D-A-AA]:103.9° [A-D-DD]:90.0° d_planarity:-52.0° a_planarity:-73.3°, maximum distance exceeded, bad d_angle(sp2)	O[ ] ... N[ ]
2VL2A	CYS47(O)	3.89	HIS51(CA)	2VL2A		O[ ] ... C[ ]
2VL2A	CYS47(O)	3.86	HIS51(C)	2VL2A		O[ ] ... C[ ]
2VL2A	CYS47(O)	3.78	HIS51(CB)	2VL2A		O[ ] ... C[ ]
2VL2A	CYS47(CB)	4.93	HIS51(CB)	2VL2A		
2VL2A	CYS47(CA)	4.80	LEU52(N)	2VL2A		
2VL2A	CYS47(C)	4.09	LEU52(N)	2VL2A		C[ ] ... N[ ]
2VL2A	CYS47(C)	4.63	LEU52(CB)	2VL2A		
2VL2A	CYS47(O)	2.94	LEU52(N)	2VL2A	**H-bond** [D-A-AA]:155.1° [A-D-DD]:113.7° d_planarity:-5.0° a_planarity:-48.0°	O[ ] ... N[ ]
2VL2A	CYS47(O)	3.77	LEU52(CA)	2VL2A		O[ ] ... C[ ]
2VL2A	CYS47(O)	4.79	LEU52(C)	2VL2A		
2VL2A	CYS47(O)	3.46	LEU52(CB)	2VL2A		O[ ] ... C[ ]
2VL2A	CYS47(O)	4.83	LEU52(CG)	2VL2A		
2VL2A	CYS47(C)	4.64	PRO53(CD)	2VL2A		
2VL2A	CYS47(O)	4.66	PRO53(N)	2VL2A		
2VL2A	CYS47(O)	4.80	PRO53(CG)	2VL2A		
2VL2A	CYS47(O)	3.67	PRO53(CD)	2VL2A		O[ ] ... C[ ]
2VL2A	CYS47(CA)	4.48	TRP84(CZ2)	2VL2A		C[ ] ... C[ ]
2VL2A	CYS47(CA)	4.70	TRP84(CH2)	2VL2A		
2VL2A	CYS47(C)	4.89	TRP84(CE2)	2VL2A		
2VL2A	CYS47(C)	3.75	TRP84(CZ2)	2VL2A		C[ ] ... C[ ]
2VL2A	CYS47(C)	4.29	TRP84(CH2)	2VL2A		C[ ] ... C[ ]
2VL2A	CYS47(O)	4.71	TRP84(CE2)	2VL2A		
2VL2A	CYS47(O)	3.53	TRP84(CZ2)	2VL2A		O[ ] ... C[ ]
2VL2A	CYS47(O)	3.94	TRP84(CH2)	2VL2A		O[ ] ... C[ ]
2VL2A	CYS47(CB)	3.89	TRP84(CZ2)	2VL2A		C[ ] ... C[ ]
2VL2A	CYS47(CB)	3.92	TRP84(CH2)	2VL2A		C[ ] ... C[ ]
2VL2A	CYS47(N)	4.26	ARG127(CZ)	2VL2A		N[ ] ... C[ ]
2VL2A	CYS47(N)	3.95	ARG127(NH1)	2VL2A		N[ ] ... N[+]
2VL2A	CYS47(N)	3.83	ARG127(NH2)	2VL2A		N[ ] ... N[+]

2VL2A	CYS47(CA)	4.52	ARG127(CZ)	2VL2A		
2VL2A	CYS47(CA)	3.78	ARG127(NH1)	2VL2A		C[ ] ... N[+]
2VL2A	CYS47(CA)	4.44	ARG127(NH2)	2VL2A		C[ ] ... N[+]
2VL2A	CYS47(CB)	4.84	ARG127(CZ)	2VL2A		
2VL2A	CYS47(CB)	4.01	ARG127(NH1)	2VL2A		C[ ] ... N[+]
2VL2A	CYS47(CB)	4.70	ARG127(NH2)	2VL2A		
2VL2A	CYS47(SG)	3.97	ARG127(CZ)	2VL2A		S[ ] ... C[ ]
2VL2A	CYS47(SG)	3.43	ARG127(NH1)	2VL2A	**H-bond** [D-A-AA]:94.6° [A-D-DD]:104.6° d_planarity:12.2°	S[ ] ... N[+]
2VL2A	CYS47(SG)	3.61	ARG127(NH2)	2VL2A	**H-bond** [D-A-AA]:116.0° [A-D-DD]:96.3° d_planarity:11.3°	S[ ] ... N[+]
2VL2A	CYS47(N)	4.46	BEZ1162(C)	2VL2A		N[ ] ... C[ ]
2VL2A	CYS47(N)	3.58	BEZ1162(O1)	2VL2A	**H-bond** [D-A-AA]:128.2° [A-D-DD]:135.2° d_planarity:-4.5°	N[ ] ... O[ ]
2VL2A	CYS47(N)	4.72	BEZ1162(O2)	2VL2A	[D-A-AA]:70.5° [A-D-DD]:159.7° d_planarity:-22.4°, maximum distance exceeded	
2VL2A	CYS47(CA)	4.73	BEZ1162(O1)	2VL2A		
2VL2A	CYS47(CB)	4.64	BEZ1162(O1)	2VL2A		
2VL2A	CYS47(SG)	4.77	BEZ1162(C)	2VL2A		
2VL2A	CYS47(SG)	3.82	BEZ1162(O1)	2VL2A	[D-A-AA]:105.1° [A-D-DD]:132.9°, maximum distance exceeded	S[ ] ... O[ ]
2VL2A	CYS47(SG)	4.79	BEZ1162(C6)	2VL2A		

chain1	res1/atm1	distance	res2/atm2	chain2	H-bonding	Charge interaction
1GZWA	CYS42(N)	3.90	LEU34(C)	1GZWA		N[ ] ... C[ ]
1GZWA	CYS42(N)	2.85	LEU34(O)	1GZWA	**H-bond** [D-A-AA]:143.0° [A-D-DD]:123.8° d_planarity:-43.5° a_planarity:33.0°	N[ ] ... O[ ]
1GZWA	CYS42(CA)	4.84	LEU34(C)	1GZWA		
1GZWA	CYS42(CA)	3.85	LEU34(O)	1GZWA		C[ ] ... O[ ]
1GZWA	CYS42(C)	4.98	LEU34(C)	1GZWA		
1GZWA	CYS42(C)	3.95	LEU34(O)	1GZWA		C[ ] ... O[ ]
1GZWA	CYS42(CB)	4.96	LEU34(C)	1GZWA		
1GZWA	CYS42(CB)	4.27	LEU34(O)	1GZWA		C[ ] ... O[ ]
1GZWA	CYS42(SG)	4.74	LEU34(CA)	1GZWA		
1GZWA	CYS42(SG)	3.85	LEU34(C)	1GZWA		S[ ] ... C[ ]
1GZWA	CYS42(SG)	3.56	LEU34(O)	1GZWA		S[ ] ... O[ ]
1GZWA	CYS42(SG)	4.26	LEU34(CB)	1GZWA		S[ ] ... C[ ]
1GZWA	CYS42(N)	4.33	GLY35(N)	1GZWA		N[ ] ... N[ ]
1GZWA	CYS42(N)	3.77	GLY35(CA)	1GZWA		N[ ] ... C[ ]
1GZWA	CYS42(CA)	4.83	GLY35(CA)	1GZWA		
1GZWA	CYS42(CB)	4.68	GLY35(CA)	1GZWA		
1GZWA	CYS42(SG)	4.02	GLY35(N)	1GZWA	[D-A-AA]:121.2° [A-D-DD]:73.8° d_planarity:59.8°, maximum distance exceeded, bad d_angle(sp2)	S[ ] ... N[ ]
1GZWA	CYS42(SG)	3.87	GLY35(CA)	1GZWA		S[ ] ... C[ ]
1GZWA	CYS42(SG)	4.94	GLY35(C)	1GZWA		
1GZWA	CYS42(N)	4.23	ASN40(C)	1GZWA		N[ ] ... C[ ]
1GZWA	CYS42(N)	4.16	ASN40(O)	1GZWA	[D-A-AA]:84.9° [A-D-DD]:148.6° d_planarity:88.8° a_planarity:24.4°, maximum distance exceeded, bad a_angle(sp2), bad d_planarity	N[ ] ... O[ ]
1GZWA	CYS42(N)	3.54	LEU41(N)	1GZWA		N[ ] ... N[ ]
1GZWA	CYS42(N)	2.43	LEU41(CA)	1GZWA		N[ ] ... C[ ]

1GZWA	CYS42(N)	2.25	LEU41(O)	1GZWA	[D-A-AA]:29.8° [A-D-DD]:92.6° d_planarity:0.8° a_planarity:0.5°, bad a_angle(sp2)	N[ ] ... O[ ]
1GZWA	CYS42(N)	3.38	LEU41(CB)	1GZWA		N[ ] ... C[ ]
1GZWA	CYS42(N)	4.74	LEU41(CG)	1GZWA		
1GZWA	CYS42(N)	4.97	LEU41(CD2)	1GZWA		
1GZWA	CYS42(CA)	4.80	LEU41(N)	1GZWA		
1GZWA	CYS42(CA)	3.79	LEU41(CA)	1GZWA		C[ ] ... C[ ]
1GZWA	CYS42(CA)	2.41	LEU41(C)	1GZWA		C[ ] ... C[ ]
1GZWA	CYS42(CA)	2.73	LEU41(O)	1GZWA		C[ ] ... O[ ]
1GZWA	CYS42(CA)	4.60	LEU41(CB)	1GZWA		
1GZWA	CYS42(C)	4.42	LEU41(CA)	1GZWA		C[ ] ... C[ ]
1GZWA	CYS42(C)	3.13	LEU41(C)	1GZWA		C[ ] ... C[ ]
1GZWA	CYS42(C)	3.28	LEU41(O)	1GZWA		C[ ] ... O[ ]
1GZWA	CYS42(C)	4.76	LEU41(CB)	1GZWA		
1GZWA	CYS42(O)	4.15	LEU41(C)	1GZWA		O[ ] ... C[ ]
1GZWA	CYS42(O)	4.02	LEU41(O)	1GZWA		O[ ] ... O[ ]
1GZWA	CYS42(CB)	4.85	LEU41(CA)	1GZWA		
1GZWA	CYS42(CB)	3.67	LEU41(C)	1GZWA		C[ ] ... C[ ]
1GZWA	CYS42(CB)	4.16	LEU41(O)	1GZWA		C[ ] ... O[ ]
1GZWA	CYS42(SG)	4.49	LEU41(C)	1GZWA		S[ ] ... C[ ]
1GZWA	CYS42(N)	2.59	LEU43(N)	1GZWA		N[ ] ... N[ ]
1GZWA	CYS42(N)	4.03	LEU43(CA)	1GZWA		N[ ] ... C[ ]
1GZWA	CYS42(N)	4.75	LEU43(C)	1GZWA		
1GZWA	CYS42(N)	5.00	LEU43(O)	1GZWA	[D-A-AA]:71.3° [A-D-DD]:98.3° d_planarity:-83.2° a_planarity:-49.1°, maximum distance exceeded, bad a_angle(sp2), bad d_planarity	
1GZWA	CYS42(N)	4.95	LEU43(CB)	1GZWA		
1GZWA	CYS42(CA)	2.44	LEU43(N)	1GZWA		C[ ] ... N[ ]
1GZWA	CYS42(CA)	3.80	LEU43(CA)	1GZWA		C[ ] ... C[ ]
1GZWA	CYS42(CA)	4.91	LEU43(C)	1GZWA		
1GZWA	CYS42(CA)	4.53	LEU43(CB)	1GZWA		
1GZWA	CYS42(C)	2.42	LEU43(CA)	1GZWA		C[ ] ... C[ ]
1GZWA	CYS42(C)	3.71	LEU43(C)	1GZWA		C[ ] ... C[ ]
1GZWA	CYS42(C)	4.40	LEU43(O)	1GZWA		C[ ] ... O[ ]
1GZWA	CYS42(C)	3.18	LEU43(CB)	1GZWA		C[ ] ... C[ ]
1GZWA	CYS42(C)	4.56	LEU43(CG)	1GZWA		
1GZWA	CYS42(C)	4.76	LEU43(CD1)	1GZWA		
1GZWA	CYS42(O)	2.25	LEU43(N)	1GZWA	[D-A-AA]:30.0° [A-D-DD]:92.6° d_planarity:0.4° a_planarity:0.7°, bad a_angle(sp2)	O[ ] ... N[ ]
1GZWA	CYS42(O)	2.73	LEU43(CA)	1GZWA		O[ ] ... C[ ]
1GZWA	CYS42(O)	4.17	LEU43(C)	1GZWA		O[ ] ... C[ ]
1GZWA	CYS42(O)	3.27	LEU43(CB)	1GZWA		O[ ] ... C[ ]
1GZWA	CYS42(O)	4.41	LEU43(CG)	1GZWA		O[ ] ... C[ ]
1GZWA	CYS42(O)	4.27	LEU43(CD1)	1GZWA		O[ ] ... C[ ]
1GZWA	CYS42(CB)	3.38	LEU43(N)	1GZWA		C[ ] ... N[ ]
1GZWA	CYS42(CB)	4.66	LEU43(CA)	1GZWA		
1GZWA	CYS42(CB)	4.95	LEU43(CB)	1GZWA		
1GZWA	CYS42(SG)	3.59	LEU43(N)	1GZWA	[D-A-AA]:68.7° [A-D-DD]:132.4° d_planarity:87.0°, bad d_planarity	S[ ] ... N[ ]
1GZWA	CYS42(SG)	4.70	LEU43(CA)	1GZWA		

1GZWA	CYS42(SG)	4.60	LEU43(CB)	1GZWA		
1GZWA	CYS42(C)	4.43	HIS44(N)	1GZWA		C[ ] ... N[ ]
1GZWA	CYS42(O)	4.69	HIS44(N)	1GZWA	[D-A-AA]:70.6° [A-D-DD]:172.4° d_planarity:-79.8° a_planarity:24.1°, maximum distance exceeded, bad a_angle(sp2), bad d_planarity	
1GZWA	CYS42(C)	4.57	ASN61(O)	1GZWA		
1GZWA	CYS42(O)	4.40	ASN61(O)	1GZWA		O[ ] ... O[ ]
1GZWA	CYS42(CA)	5.00	SER62(CB)	1GZWA		
1GZWA	CYS42(C)	4.74	SER62(CA)	1GZWA		
1GZWA	CYS42(C)	4.84	SER62(CB)	1GZWA		
1GZWA	CYS42(O)	4.66	SER62(CA)	1GZWA		
1GZWA	CYS42(O)	4.58	SER62(CB)	1GZWA		
1GZWA	CYS42(CA)	4.69	LEU96(CD1)	1GZWA		
1GZWA	CYS42(C)	4.22	LEU96(CD1)	1GZWA		C[ ] ... C[ ]
1GZWA	CYS42(C)	4.91	LEU96(CD2)	1GZWA		
1GZWA	CYS42(O)	4.79	LEU96(CG)	1GZWA		
1GZWA	CYS42(O)	3.61	LEU96(CD1)	1GZWA		O[ ] ... C[ ]
1GZWA	CYS42(O)	4.75	LEU96(CD2)	1GZWA		
1GZWA	CYS42(CB)	4.91	LEU96(CG)	1GZWA		
1GZWA	CYS42(CB)	4.00	LEU96(CD1)	1GZWA		C[ ] ... C[ ]
1GZWA	CYS42(CB)	4.71	LEU96(CD2)	1GZWA		
1GZWA	CYS42(SG)	4.64	LEU96(CG)	1GZWA		
1GZWA	CYS42(SG)	4.27	LEU96(CD1)	1GZWA		S[ ] ... C[ ]
1GZWA	CYS42(SG)	3.95	LEU96(CD2)	1GZWA		S[ ] ... C[ ]
1GZWA	CYS42(C)	4.62	PHE108(CD2)	1GZWA		
1GZWA	CYS42(C)	4.55	PHE108(CE2)	1GZWA		
1GZWA	CYS42(O)	4.73	PHE108(CG)	1GZWA		
1GZWA	CYS42(O)	3.44	PHE108(CD2)	1GZWA		O[ ] ... C[ ]
1GZWA	CYS42(O)	3.50	PHE108(CE2)	1GZWA		O[ ] ... C[ ]
1GZWA	CYS42(O)	4.82	PHE108(CZ)	1GZWA		
1GZWA	CYS42(O)	4.46	PRO109(O)	1GZWA		O[ ] ... O[ ]
1GZWA	CYS42(CB)	4.11	ASN110(OD1)	1GZWA		C[ ] ... O[ ]
1GZWA	CYS42(N)	4.28	LEU112(CD2)	1GZWA		N[ ] ... C[ ]
1GZWA	CYS42(CA)	4.74	LEU112(CD1)	1GZWA		
1GZWA	CYS42(CA)	4.62	LEU112(CD2)	1GZWA		
1GZWA	CYS42(N)	4.38	LEU114(CD2)	1GZWA		N[ ] ... C[ ]
1GZWA	CYS42(CA)	4.54	LEU114(CD2)	1GZWA		
1GZWA	CYS42(CB)	4.81	LEU114(CB)	1GZWA		
1GZWA	CYS42(CB)	4.75	LEU114(CG)	1GZWA		
1GZWA	CYS42(CB)	3.63	LEU114(CD2)	1GZWA		C[ ] ... C[ ]
1GZWA	CYS42(SG)	3.76	LEU114(CD2)	1GZWA		S[ ] ... C[ ]
1GZWA	CYS42(SG)	4.92	ILE117(CB)	1GZWA		
1GZWA	CYS42(SG)	3.79	ILE117(CG1)	1GZWA		S[ ] ... C[ ]
1GZWA	CYS42(SG)	4.89	ILE117(CG2)	1GZWA		
1GZWA	CYS42(SG)	3.69	ILE117(CD1)	1GZWA		S[ ] ... C[ ]

chain1	res1/atm1	distance	res2/atm2	chain2	H-bonding	Charge interaction

1GZWA	CYS60(CA)	4.94	LEU43(CD1)	1GZWA		
1GZWA	CYS60(CB)	4.11	LEU43(CD1)	1GZWA		C[ ] ... C[ ]
1GZWA	CYS60(SG)	4.04	LEU43(CD1)	1GZWA		S[ ] ... C[ ]
1GZWA	CYS60(N)	4.16	HIS44(O)	1GZWA	[D-A-AA]:164.2° [A-D-DD]:37.8° d_planarity:11.0° a_planarity:4.5°, maximum distance exceeded, bad d_angle(sp2)	N[ ] ... O[ ]
1GZWA	CYS60(CA)	4.34	HIS44(C)	1GZWA		C[ ] ... C[ ]
1GZWA	CYS60(CA)	3.13	HIS44(O)	1GZWA		C[ ] ... O[ ]
1GZWA	CYS60(C)	4.77	HIS44(C)	1GZWA		
1GZWA	CYS60(C)	3.60	HIS44(O)	1GZWA		C[ ] ... O[ ]
1GZWA	CYS60(O)	4.80	HIS44(O)	1GZWA		
1GZWA	CYS60(CB)	4.86	HIS44(C)	1GZWA		
1GZWA	CYS60(CB)	3.82	HIS44(O)	1GZWA		C[ ] ... O[ ]
1GZWA	CYS60(SG)	4.95	HIS44(C)	1GZWA	Weakly positive	
1GZWA	CYS60(SG)	4.17	HIS44(O)	1GZWA		S[ ] ... O[ ]
1GZWA	CYS60(CA)	4.92	PHE45(CA)	1GZWA		
1GZWA	CYS60(SG)	4.35	PHE45(CA)	1GZWA		S[ ] ... C[ ]
1GZWA	CYS60(SG)	4.86	PHE45(CB)	1GZWA		
1GZWA	CYS60(SG)	4.76	PHE45(CG)	1GZWA		
1GZWA	CYS60(SG)	3.83	PHE45(CD1)	1GZWA		S[ ] ... C[ ]
1GZWA	CYS60(SG)	4.45	PHE45(CE1)	1GZWA		S[ ] ... C[ ]
1GZWA	CYS60(N)	4.92	ASN46(N)	1GZWA		
1GZWA	CYS60(CA)	4.90	ASN46(N)	1GZWA		
1GZWA	CYS60(SG)	4.99	ASN46(N)	1GZWA	[D-A-AA]:100.4° [A-D-DD]:143.7° d_planarity:-15.3°, maximum distance exceeded	
1GZWA	CYS60(N)	4.15	ILE58(C)	1GZWA		N[ ] ... C[ ]
1GZWA	CYS60(N)	4.14	ILE58(O)	1GZWA	[D-A-AA]:81.6° [A-D-DD]:146.9° d_planarity:88.2° a_planarity:30.0°, maximum distance exceeded, bad a_angle(sp2), bad d_planarity	N[ ] ... O[ ]
1GZWA	CYS60(SG)	4.09	ILE58(CG2)	1GZWA		S[ ] ... C[ ]
1GZWA	CYS60(N)	3.47	VAL59(N)	1GZWA		N[ ] ... N[ ]
1GZWA	CYS60(N)	2.43	VAL59(CA)	1GZWA		N[ ] ... C[ ]
1GZWA	CYS60(N)	2.25	VAL59(O)	1GZWA	[D-A-AA]:29.7° [A-D-DD]:94.4° d_planarity:1.1° a_planarity:1.2°, bad a_angle(sp2)	N[ ] ... O[ ]
1GZWA	CYS60(N)	3.39	VAL59(CB)	1GZWA		N[ ] ... C[ ]
1GZWA	CYS60(N)	3.30	VAL59(CG1)	1GZWA		N[ ] ... C[ ]
1GZWA	CYS60(N)	4.68	VAL59(CG2)	1GZWA		
1GZWA	CYS60(CA)	4.71	VAL59(N)	1GZWA		
1GZWA	CYS60(CA)	3.81	VAL59(CA)	1GZWA		C[ ] ... C[ ]
1GZWA	CYS60(CA)	2.44	VAL59(C)	1GZWA		C[ ] ... C[ ]
1GZWA	CYS60(CA)	2.78	VAL59(O)	1GZWA		C[ ] ... O[ ]
1GZWA	CYS60(CA)	4.64	VAL59(CB)	1GZWA		
1GZWA	CYS60(CA)	4.36	VAL59(CG1)	1GZWA		C[ ] ... C[ ]
1GZWA	CYS60(C)	4.69	VAL59(CA)	1GZWA		
1GZWA	CYS60(C)	3.49	VAL59(C)	1GZWA		C[ ] ... C[ ]
1GZWA	CYS60(C)	3.90	VAL59(O)	1GZWA		C[ ] ... O[ ]
1GZWA	CYS60(C)	4.45	VAL59(CG1)	1GZWA		C[ ] ... C[ ]
1GZWA	CYS60(O)	4.76	VAL59(CA)	1GZWA		
1GZWA	CYS60(O)	3.88	VAL59(C)	1GZWA		O[ ] ... C[ ]
1GZWA	CYS60(O)	4.57	VAL59(O)	1GZWA		
1GZWA	CYS60(O)	4.21	VAL59(CG1)	1GZWA		O[ ] ... C[ ]

1GZWA	CYS60(CB)	4.72	VAL59(CA)	1GZWA		
1GZWA	CYS60(CB)	3.48	VAL59(C)	1GZWA		C[ ] ... C[ ]
1GZWA	CYS60(CB)	3.83	VAL59(O)	1GZWA		C[ ] ... O[ ]
1GZWA	CYS60(SG)	4.94	VAL59(CA)	1GZWA		
1GZWA	CYS60(SG)	3.81	VAL59(C)	1GZWA		S[ ] ... C[ ]
1GZWA	CYS60(SG)	3.86	VAL59(O)	1GZWA		S[ ] ... O[ ]
1GZWA	CYS60(N)	3.58	ASN61(N)	1GZWA		N[ ] ... N[ ]
1GZWA	CYS60(N)	4.78	ASN61(CA)	1GZWA		
1GZWA	CYS60(CA)	2.42	ASN61(N)	1GZWA		C[ ] ... N[ ]
1GZWA	CYS60(CA)	3.77	ASN61(CA)	1GZWA		C[ ] ... C[ ]
1GZWA	CYS60(CA)	4.79	ASN61(C)	1GZWA		
1GZWA	CYS60(CA)	4.76	ASN61(O)	1GZWA		
1GZWA	CYS60(CA)	4.47	ASN61(CB)	1GZWA		C[ ] ... C[ ]
1GZWA	CYS60(C)	2.40	ASN61(CA)	1GZWA		C[ ] ... C[ ]
1GZWA	CYS60(C)	3.63	ASN61(C)	1GZWA		C[ ] ... C[ ]
1GZWA	CYS60(C)	3.94	ASN61(O)	1GZWA		C[ ] ... O[ ]
1GZWA	CYS60(C)	3.13	ASN61(CB)	1GZWA		C[ ] ... C[ ]
1GZWA	CYS60(C)	4.32	ASN61(CG)	1GZWA		C[ ] ... C[ ]
1GZWA	CYS60(C)	4.69	ASN61(ND2)	1GZWA		
1GZWA	CYS60(O)	2.24	ASN61(N)	1GZWA	[D-A-AA]:29.9° [A-D-DD]:92.0° d_planarity:0.4° a_planarity:0.1°, bad a_angle(sp2)	O[ ] ... N[ ]
1GZWA	CYS60(O)	2.71	ASN61(CA)	1GZWA		O[ ] ... C[ ]
1GZWA	CYS60(O)	4.14	ASN61(C)	1GZWA		O[ ] ... C[ ]
1GZWA	CYS60(O)	4.73	ASN61(O)	1GZWA		
1GZWA	CYS60(O)	3.20	ASN61(CB)	1GZWA		O[ ] ... C[ ]
1GZWA	CYS60(O)	4.02	ASN61(CG)	1GZWA		O[ ] ... C[ ]
1GZWA	CYS60(O)	4.83	ASN61(OD1)	1GZWA		
1GZWA	CYS60(O)	4.32	ASN61(ND2)	1GZWA	[D-A-AA]:99.9° [A-D-DD]:68.3° d_planarity:-43.9° a_planarity:2.8°, maximum distance exceeded, bad d_angle(sp2)	O[ ] ... N[ ]
1GZWA	CYS60(CB)	3.21	ASN61(N)	1GZWA		C[ ] ... N[ ]
1GZWA	CYS60(CB)	4.48	ASN61(CA)	1GZWA		C[ ] ... C[ ]
1GZWA	CYS60(SG)	4.74	ASN61(N)	1GZWA	[D-A-AA]:26.0° [A-D-DD]:157.8° d_planarity:69.9°, maximum distance exceeded, bad a_angle(sp3), bad d_planarity	
1GZWA	CYS60(C)	4.72	SER62(N)	1GZWA		
1GZWA	CYS60(O)	4.99	SER62(N)	1GZWA	[D-A-AA]:70.4° [A-D-DD]:162.8° d_planarity:34.1° a_planarity:9.9°, maximum distance exceeded, bad a_angle(sp2)	
1GZWA	CYS60(O)	4.86	THR70(C)	1GZWA		
1GZWA	CYS60(O)	4.34	THR70(O)	1GZWA		O[ ] ... O[ ]
1GZWA	CYS60(C)	4.56	GLU71(CA)	1GZWA		
1GZWA	CYS60(C)	4.82	GLU71(C)	1GZWA		
1GZWA	CYS60(C)	5.00	GLU71(CB)	1GZWA		
1GZWA	CYS60(O)	4.54	GLU71(N)	1GZWA	[D-A-AA]:150.2° [A-D-DD]:31.4° d_planarity:40.3° a_planarity:19.3°, maximum distance exceeded, bad d_angle(sp2)	
1GZWA	CYS60(O)	3.38	GLU71(CA)	1GZWA		O[ ] ... C[ ]
1GZWA	CYS60(O)	3.59	GLU71(C)	1GZWA		O[ ] ... C[ ]
1GZWA	CYS60(O)	4.79	GLU71(O)	1GZWA		
1GZWA	CYS60(O)	3.93	GLU71(CB)	1GZWA		O[ ] ... C[ ]
1GZWA	CYS60(O)	4.50	GLU71(CG)	1GZWA		

1GZWA	CYS60(N)	4.31	GLN72(N)	1GZWA		N[ ] ... N[ ]
1GZWA	CYS60(N)	4.68	GLN72(CA)	1GZWA		
1GZWA	CYS60(N)	3.99	GLN72(C)	1GZWA		N[ ] ... C[ ]
1GZWA	CYS60(N)	2.82	GLN72(O)	1GZWA	**H-bond** [D-A-AA]:158.8° [A-D-DD]:122.2° d_planarity:-5.7° a_planarity:-30.8°	N[ ] ... O[ ]
1GZWA	CYS60(N)	4.91	GLN72(CB)	1GZWA		
1GZWA	CYS60(CA)	4.67	GLN72(N)	1GZWA		
1GZWA	CYS60(CA)	4.81	GLN72(C)	1GZWA		
1GZWA	CYS60(CA)	3.80	GLN72(O)	1GZWA		C[ ] ... O[ ]
1GZWA	CYS60(C)	4.01	GLN72(N)	1GZWA		C[ ] ... N[ ]
1GZWA	CYS60(C)	4.81	GLN72(CA)	1GZWA		
1GZWA	CYS60(C)	4.89	GLN72(C)	1GZWA		
1GZWA	CYS60(C)	4.08	GLN72(O)	1GZWA		C[ ] ... O[ ]
1GZWA	CYS60(C)	4.74	GLN72(CB)	1GZWA		
1GZWA	CYS60(O)	2.85	GLN72(N)	1GZWA	**H-bond** [D-A-AA]:156.3° [A-D-DD]:122.1° d_planarity:-15.5° a_planarity:-31.4°	O[ ] ... N[ ]
1GZWA	CYS60(O)	3.83	GLN72(CA)	1GZWA		O[ ] ... C[ ]
1GZWA	CYS60(O)	4.10	GLN72(C)	1GZWA		O[ ] ... C[ ]
1GZWA	CYS60(O)	3.47	GLN72(O)	1GZWA		O[ ] ... O[ ]
1GZWA	CYS60(O)	3.97	GLN72(CB)	1GZWA		O[ ] ... C[ ]
1GZWA	CYS60(CB)	5.00	GLN72(N)	1GZWA		
1GZWA	CYS60(CB)	4.85	GLN72(C)	1GZWA		
1GZWA	CYS60(CB)	4.05	GLN72(O)	1GZWA		C[ ] ... O[ ]
1GZWA	CYS60(CB)	4.56	GLN72(CB)	1GZWA		
1GZWA	CYS60(SG)	4.97	GLN72(O)	1GZWA		
1GZWA	CYS60(SG)	4.90	GLU74(OE2)	1GZWA	Negative	
1GZWA	CYS60(CB)	4.84	PHE106(CE2)	1GZWA		
1GZWA	CYS60(SG)	3.75	PHE106(CE2)	1GZWA		S[ ] ... C[ ]
1GZWA	CYS60(SG)	3.84	PHE106(CZ)	1GZWA		S[ ] ... C[ ]
1GZWA	CYS60(CA)	4.64	PHE108(CE1)	1GZWA		
1GZWA	CYS60(C)	4.72	PHE108(CD1)	1GZWA		
1GZWA	CYS60(C)	3.94	PHE108(CE1)	1GZWA		C[ ] ... C[ ]
1GZWA	CYS60(C)	4.96	PHE108(CE2)	1GZWA		
1GZWA	CYS60(C)	4.09	PHE108(CZ)	1GZWA		C[ ] ... C[ ]
1GZWA	CYS60(O)	4.82	PHE108(CD1)	1GZWA		
1GZWA	CYS60(O)	3.88	PHE108(CE1)	1GZWA		O[ ] ... C[ ]
1GZWA	CYS60(O)	4.19	PHE108(CZ)	1GZWA		O[ ] ... C[ ]
1GZWA	CYS60(CB)	4.56	PHE108(CG)	1GZWA		
1GZWA	CYS60(CB)	3.95	PHE108(CD1)	1GZWA		C[ ] ... C[ ]
1GZWA	CYS60(CB)	3.96	PHE108(CE1)	1GZWA		C[ ] ... C[ ]
1GZWA	CYS60(CB)	4.58	PHE108(CZ)	1GZWA		

chain1	res1/atm1	distance	res2/atm2	chain2	H-bonding	Charge interaction
1W60A	CYS81(N)	4.90	LYS77(C)	1W60A		
1W60A	CYS81(N)	3.97	LYS77(O)	1W60A	[D-A-AA]:133.0° [A-D-DD]:122.3° d_planarity:-62.3° a_planarity:54.3°, maximum distance exceeded, bad d_planarity	N[ ] ... O[ ]
1W60A	CYS81(CA)	4.91	LYS77(O)	1W60A		

1W60A	CYS81(CB)	4.69	LYS77(O)	1W60A		
1W60A	CYS81(SG)	4.46	LYS77(C)	1W60A		S[ ] ... C[ ]
1W60A	CYS81(SG)	3.43	LYS77(O)	1W60A	Weakly positive	S[ ] ... O[ ]
1W60A	CYS81(N)	4.75	ILE78(CA)	1W60A		
1W60A	CYS81(N)	3.72	ILE78(C)	1W60A		N[ ] ... C[ ]
1W60A	CYS81(N)	2.97	ILE78(O)	1W60A	**H-bond** [D-A-AA]:118.2° [A-D-DD]:102.2° d_planarity:-0.6° a_planarity:70.9°	N[ ] ... O[ ]
1W60A	CYS81(CA)	4.59	ILE78(C)	1W60A		
1W60A	CYS81(CA)	3.58	ILE78(O)	1W60A		C[ ] ... O[ ]
1W60A	CYS81(C)	4.18	ILE78(O)	1W60A		C[ ] ... O[ ]
1W60A	CYS81(CB)	4.89	ILE78(CA)	1W60A		
1W60A	CYS81(CB)	4.28	ILE78(C)	1W60A		C[ ] ... C[ ]
1W60A	CYS81(CB)	3.21	ILE78(O)	1W60A		C[ ] ... O[ ]
1W60A	CYS81(SG)	4.79	ILE78(N)	1W60A	[D-A-AA]:115.8° [A-D-DD]:54.3° d_planarity:9.6°, maximum distance exceeded, bad d_angle(sp2)	
1W60A	CYS81(SG)	4.11	ILE78(CA)	1W60A		S[ ] ... C[ ]
1W60A	CYS81(SG)	4.03	ILE78(C)	1W60A		S[ ] ... C[ ]
1W60A	CYS81(SG)	3.38	ILE78(O)	1W60A		S[ ] ... O[ ]
1W60A	CYS81(N)	4.18	LEU79(N)	1W60A		N[ ] ... N[ ]
1W60A	CYS81(N)	4.04	LEU79(CA)	1W60A		N[ ] ... C[ ]
1W60A	CYS81(N)	3.13	LEU79(C)	1W60A		N[ ] ... C[ ]
1W60A	CYS81(N)	3.47	LEU79(O)	1W60A	[D-A-AA]:63.6° [A-D-DD]:134.1° d_planarity:-86.1° a_planarity:54.7°, bad a_angle(sp2), bad d_planarity	N[ ] ... O[ ]
1W60A	CYS81(CA)	4.41	LEU79(C)	1W60A		C[ ] ... C[ ]
1W60A	CYS81(CA)	4.61	LEU79(O)	1W60A		
1W60A	CYS81(C)	4.70	LEU79(C)	1W60A		
1W60A	CYS81(C)	4.56	LEU79(O)	1W60A		
1W60A	CYS81(N)	2.65	LYS80(N)	1W60A		N[ ] ... N[ ]
1W60A	CYS81(N)	2.43	LYS80(CA)	1W60A		N[ ] ... C[ ]
1W60A	CYS81(N)	2.25	LYS80(O)	1W60A	[D-A-AA]:29.7° [A-D-DD]:94.5° d_planarity:0.8° a_planarity:0.3°, bad a_angle(sp2)	N[ ] ... O[ ]
1W60A	CYS81(N)	3.40	LYS80(CB)	1W60A		N[ ] ... C[ ]
1W60A	CYS81(N)	4.81	LYS80(CG)	1W60A		
1W60A	CYS81(CA)	4.10	LYS80(N)	1W60A		C[ ] ... N[ ]
1W60A	CYS81(CA)	3.80	LYS80(CA)	1W60A		C[ ] ... C[ ]
1W60A	CYS81(CA)	2.44	LYS80(C)	1W60A		C[ ] ... C[ ]
1W60A	CYS81(CA)	2.78	LYS80(O)	1W60A		C[ ] ... O[ ]
1W60A	CYS81(CA)	4.67	LYS80(CB)	1W60A		
1W60A	CYS81(C)	4.79	LYS80(N)	1W60A		
1W60A	CYS81(C)	4.57	LYS80(CA)	1W60A		
1W60A	CYS81(C)	3.25	LYS80(C)	1W60A		C[ ] ... C[ ]
1W60A	CYS81(C)	3.41	LYS80(O)	1W60A		C[ ] ... O[ ]
1W60A	CYS81(O)	4.32	LYS80(C)	1W60A		O[ ] ... C[ ]
1W60A	CYS81(O)	4.23	LYS80(O)	1W60A		O[ ] ... O[ ]
1W60A	CYS81(CB)	4.79	LYS80(N)	1W60A		
1W60A	CYS81(CB)	4.81	LYS80(CA)	1W60A		
1W60A	CYS81(CB)	3.65	LYS80(C)	1W60A		C[ ] ... C[ ]
1W60A	CYS81(CB)	4.16	LYS80(O)	1W60A		C[ ] ... O[ ]
1W60A	CYS81(SG)	4.80	LYS80(N)	1W60A	[D-A-AA]:78.6° [A-D-DD]:83.5° d_planarity:-64.5°, maximum distance exceeded, bad d_angle(sp2), bad d_planarity	

1W60A	CYS81(SG)	4.86	LYS80(CA)	1W60A		
1W60A	CYS81(SG)	4.04	LYS80(C)	1W60A		S[ ] ... C[ ]
1W60A	CYS81(SG)	4.66	LYS80(O)	1W60A	Weakly positive	
1W60A	CYS81(N)	2.66	ALA82(N)	1W60A		N[ ] ... N[ ]
1W60A	CYS81(N)	4.10	ALA82(CA)	1W60A		N[ ] ... C[ ]
1W60A	CYS81(N)	4.77	ALA82(C)	1W60A		
1W60A	CYS81(N)	4.53	ALA82(O)	1W60A	[D-A-AA]:94.0° [A-D-DD]:80.5° d_planarity:62.3° a_planarity:-25.2°, maximum distance exceeded, bad d_angle(sp2), bad d_planarity	
1W60A	CYS81(N)	4.78	ALA82(CB)	1W60A		
1W60A	CYS81(CA)	2.42	ALA82(N)	1W60A		C[ ] ... N[ ]
1W60A	CYS81(CA)	3.80	ALA82(CA)	1W60A		C[ ] ... C[ ]
1W60A	CYS81(CA)	4.51	ALA82(C)	1W60A		
1W60A	CYS81(CA)	4.52	ALA82(O)	1W60A		
1W60A	CYS81(CA)	4.82	ALA82(CB)	1W60A		
1W60A	CYS81(C)	2.43	ALA82(CA)	1W60A		C[ ] ... C[ ]
1W60A	CYS81(C)	3.19	ALA82(C)	1W60A		C[ ] ... C[ ]
1W60A	CYS81(C)	3.49	ALA82(O)	1W60A		C[ ] ... O[ ]
1W60A	CYS81(C)	3.67	ALA82(CB)	1W60A		C[ ] ... C[ ]
1W60A	CYS81(O)	2.25	ALA82(N)	1W60A	[D-A-AA]:29.6° [A-D-DD]:94.3° d_planarity:0.7° a_planarity:0.1°, bad a_angle(sp2)	O[ ] ... N[ ]
1W60A	CYS81(O)	2.77	ALA82(CA)	1W60A		O[ ] ... C[ ]
1W60A	CYS81(O)	3.31	ALA82(C)	1W60A		O[ ] ... C[ ]
1W60A	CYS81(O)	3.84	ALA82(O)	1W60A		O[ ] ... O[ ]
1W60A	CYS81(O)	4.18	ALA82(CB)	1W60A		O[ ] ... C[ ]
1W60A	CYS81(CB)	3.41	ALA82(N)	1W60A		C[ ] ... N[ ]
1W60A	CYS81(CB)	4.68	ALA82(CA)	1W60A		
1W60A	CYS81(SG)	4.94	ALA82(N)	1W60A	[D-A-AA]:26.1° [A-D-DD]:158.1° d_planarity:53.2°, maximum distance exceeded, bad a_angle(sp3)	
1W60A	CYS81(C)	4.11	GLY83(N)	1W60A		C[ ] ... N[ ]
1W60A	CYS81(O)	3.86	GLY83(N)	1W60A	[D-A-AA]:92.7° [A-D-DD]:134.1° d_planarity:71.6° a_planarity:24.1°, maximum distance exceeded, bad d_planarity	O[ ] ... N[ ]
1W60A	CYS81(O)	4.98	GLY83(CA)	1W60A		
1W60A	CYS81(N)	4.88	PHE103(CE2)	1W60A		
1W60A	CYS81(CA)	4.70	PHE103(CE2)	1W60A		
1W60A	CYS81(C)	4.20	PHE103(CD2)	1W60A		C[ ] ... C[ ]
1W60A	CYS81(C)	4.22	PHE103(CE2)	1W60A		C[ ] ... C[ ]
1W60A	CYS81(O)	4.36	PHE103(CD2)	1W60A		O[ ] ... C[ ]
1W60A	CYS81(O)	4.74	PHE103(CE2)	1W60A		
1W60A	CYS81(CB)	4.65	PHE103(CD2)	1W60A		
1W60A	CYS81(CB)	4.31	PHE103(CE2)	1W60A		C[ ] ... C[ ]
1W60A	CYS81(O)	4.48	LYS110(NZ)	1W60A	[D-A-AA]:158.5° [A-D-DD]:113.5° a_planarity:19.6°, maximum distance exceeded	O[ ] ... N[+]
1W60A	CYS81(CA)	4.74	TYR114(OH)	1W60A		
1W60A	CYS81(O)	4.96	TYR114(OH)	1W60A	[D-A-AA]:91.1° [A-D-DD]:108.7° d_planarity:43.3° a_planarity:-50.3°, maximum distance exceeded	
1W60A	CYS81(CB)	4.47	TYR114(CE1)	1W60A		C[ ] ... C[ ]
1W60A	CYS81(CB)	4.14	TYR114(CE2)	1W60A		C[ ] ... C[ ]
1W60A	CYS81(CB)	3.77	TYR114(CZ)	1W60A		C[ ] ... C[ ]
1W60A	CYS81(CB)	3.35	TYR114(OH)	1W60A		C[ ] ... O[ ]
1W60A	CYS81(SG)	4.27	TYR114(CE1)	1W60A		S[ ] ... C[ ]

1W60A	CYS81(SG)	4.88	TYR114(CE2)	1W60A		
1W60A	CYS81(SG)	4.11	TYR114(CZ)	1W60A		S[ ] ... C[ ]
1W60A	CYS81(SG)	3.76	TYR114(OH)	1W60A	[D-A-AA]:62.7° [A-D-DD]:94.4° d_planarity:-71.7°, bad d_planarity	S[ ] ... O[ ]

chain1	res1/atm1	distance	res2/atm2	chain2	H-bonding	Charge interaction
1W60A	CYS162(CB)	4.44	CYS135(CB)	1W60A		C[ ] ... C[ ]
1W60A	CYS162(CB)	3.80	CYS135(SG)	1W60A		C[ ] ... S[ ]
1W60A	CYS162(SG)	3.97	CYS135(CB)	1W60A		S[ ] ... C[ ]
1W60A	CYS162(SG)	3.70	CYS135(SG)	1W60A	Disulphide bridge?	S[ ] ... S[ ]
1W60A	CYS162(SG)	4.00	VAL137(CG2)	1W60A		S[ ] ... C[ ]
1W60A	CYS162(N)	4.30	ILE160(C)	1W60A		N[ ] ... C[ ]
1W60A	CYS162(N)	4.29	ILE160(O)	1W60A	[D-A-AA]:82.0° [A-D-DD]:142.7° d_planarity:77.8° a_planarity:27.0°, maximum distance exceeded, bad a_angle(sp2), bad d_planarity	N[ ] ... O[ ]
1W60A	CYS162(N)	3.57	SER161(N)	1W60A		N[ ] ... N[ ]
1W60A	CYS162(N)	2.42	SER161(CA)	1W60A		N[ ] ... C[ ]
1W60A	CYS162(N)	2.25	SER161(O)	1W60A	[D-A-AA]:29.7° [A-D-DD]:94.3° d_planarity:1.5° a_planarity:0.1°, bad a_angle(sp2)	N[ ] ... O[ ]
1W60A	CYS162(N)	3.30	SER161(CB)	1W60A		N[ ] ... C[ ]
1W60A	CYS162(N)	4.61	SER161(OG)	1W60A	[D-A-AA]:18.5° [A-D-DD]:155.0° d_planarity:40.2°, maximum distance exceeded, bad a_angle(sp3)	
1W60A	CYS162(CA)	4.79	SER161(N)	1W60A		
1W60A	CYS162(CA)	3.80	SER161(CA)	1W60A		C[ ] ... C[ ]
1W60A	CYS162(CA)	2.43	SER161(C)	1W60A		C[ ] ... C[ ]
1W60A	CYS162(CA)	2.77	SER161(O)	1W60A		C[ ] ... O[ ]
1W60A	CYS162(CA)	4.61	SER161(CB)	1W60A		
1W60A	CYS162(C)	4.78	SER161(CA)	1W60A		
1W60A	CYS162(C)	3.56	SER161(C)	1W60A		C[ ] ... C[ ]
1W60A	CYS162(C)	3.96	SER161(O)	1W60A		C[ ] ... O[ ]
1W60A	CYS162(O)	4.89	SER161(CA)	1W60A		
1W60A	CYS162(O)	3.98	SER161(C)	1W60A		O[ ] ... C[ ]
1W60A	CYS162(O)	4.66	SER161(O)	1W60A		
1W60A	CYS162(CB)	4.62	SER161(CA)	1W60A		
1W60A	CYS162(CB)	3.40	SER161(C)	1W60A		C[ ] ... C[ ]
1W60A	CYS162(CB)	3.73	SER161(O)	1W60A		C[ ] ... O[ ]
1W60A	CYS162(SG)	4.62	SER161(C)	1W60A		
1W60A	CYS162(SG)	4.49	SER161(O)	1W60A		S[ ] ... O[ ]
1W60A	CYS162(N)	3.60	ALA163(N)	1W60A		N[ ] ... N[ ]
1W60A	CYS162(N)	4.84	ALA163(CA)	1W60A		
1W60A	CYS162(CA)	2.43	ALA163(N)	1W60A		C[ ] ... N[ ]
1W60A	CYS162(CA)	3.81	ALA163(CA)	1W60A		C[ ] ... C[ ]
1W60A	CYS162(CA)	4.81	ALA163(C)	1W60A		
1W60A	CYS162(CA)	4.80	ALA163(O)	1W60A		
1W60A	CYS162(CA)	4.55	ALA163(CB)	1W60A		
1W60A	CYS162(C)	2.44	ALA163(CA)	1W60A		C[ ] ... C[ ]
1W60A	CYS162(C)	3.66	ALA163(C)	1W60A		C[ ] ... C[ ]
1W60A	CYS162(C)	3.91	ALA163(O)	1W60A		C[ ] ... O[ ]

1W60A	CYS162(C)	3.24	ALA163(CB)	1W60A		C[ ] ... C[ ]
1W60A	CYS162(O)	2.25	ALA163(N)	1W60A	[D-A-AA]:29.7° [A-D-DD]:94.5° d_planarity:0.7° a_planarity:0.3°, bad a_angle(sp2)	O[ ] ... N[ ]
1W60A	CYS162(O)	2.78	ALA163(CA)	1W60A		O[ ] ... C[ ]
1W60A	CYS162(O)	4.16	ALA163(C)	1W60A		O[ ] ... C[ ]
1W60A	CYS162(O)	4.58	ALA163(O)	1W60A		
1W60A	CYS162(O)	3.40	ALA163(CB)	1W60A		O[ ] ... C[ ]
1W60A	CYS162(CB)	3.24	ALA163(N)	1W60A		C[ ] ... N[ ]
1W60A	CYS162(CB)	4.53	ALA163(CA)	1W60A		
1W60A	CYS162(CB)	4.90	ALA163(O)	1W60A		
1W60A	CYS162(SG)	3.73	ALA163(N)	1W60A	[D-A-AA]:60.3° [A-D-DD]:142.6° d_planarity:-71.9°, bad d_planarity	S[ ] ... N[ ]
1W60A	CYS162(SG)	4.97	ALA163(CA)	1W60A		
1W60A	CYS162(SG)	4.49	ALA163(O)	1W60A		S[ ] ... O[ ]
1W60A	CYS162(C)	4.80	LYS164(N)	1W60A		
1W60A	CYS162(N)	4.95	GLY166(O)	1W60A	[D-A-AA]:134.7° [A-D-DD]:46.4° d_planarity:39.4° a_planarity:23.6°, maximum distance exceeded, bad d_angle(sp2)	
1W60A	CYS162(CA)	4.99	GLY166(C)	1W60A		
1W60A	CYS162(CA)	4.08	GLY166(O)	1W60A		C[ ] ... O[ ]
1W60A	CYS162(C)	4.09	GLY166(O)	1W60A		C[ ] ... O[ ]
1W60A	CYS162(SG)	4.80	GLY166(O)	1W60A		
1W60A	CYS162(CA)	4.57	VAL167(CA)	1W60A		
1W60A	CYS162(CA)	4.84	VAL167(CB)	1W60A		
1W60A	CYS162(SG)	4.72	VAL167(CA)	1W60A		
1W60A	CYS162(SG)	4.23	VAL167(CB)	1W60A		S[ ] ... C[ ]
1W60A	CYS162(SG)	4.80	VAL167(CG2)	1W60A		
1W60A	CYS162(C)	4.36	PRO202(CA)	1W60A		C[ ] ... C[ ]
1W60A	CYS162(C)	4.86	PRO202(C)	1W60A		
1W60A	CYS162(C)	4.51	PRO202(CB)	1W60A		
1W60A	CYS162(O)	4.78	PRO202(N)	1W60A		
1W60A	CYS162(O)	3.46	PRO202(CA)	1W60A		O[ ] ... C[ ]
1W60A	CYS162(O)	3.91	PRO202(C)	1W60A		O[ ] ... C[ ]
1W60A	CYS162(O)	3.43	PRO202(CB)	1W60A		O[ ] ... C[ ]
1W60A	CYS162(O)	4.52	PRO202(CG)	1W60A		
1W60A	CYS162(N)	4.53	VAL203(N)	1W60A		
1W60A	CYS162(N)	4.87	VAL203(CA)	1W60A		
1W60A	CYS162(N)	4.06	VAL203(C)	1W60A		N[ ] ... C[ ]
1W60A	CYS162(N)	2.84	VAL203(O)	1W60A	**H-bond** [D-A-AA]:169.4° [A-D-DD]:117.9° d_planarity:-23.5° a_planarity:-7.5°	N[ ] ... O[ ]
1W60A	CYS162(CA)	4.61	VAL203(N)	1W60A		
1W60A	CYS162(CA)	4.85	VAL203(C)	1W60A		
1W60A	CYS162(CA)	3.75	VAL203(O)	1W60A		C[ ] ... O[ ]
1W60A	CYS162(C)	4.34	VAL203(N)	1W60A		C[ ] ... N[ ]
1W60A	CYS162(C)	4.19	VAL203(O)	1W60A		C[ ] ... O[ ]
1W60A	CYS162(O)	3.57	VAL203(N)	1W60A	[D-A-AA]:121.5° [A-D-DD]:129.9° d_planarity:-49.4° a_planarity:-53.7°, maximum distance exceeded	O[ ] ... N[ ]
1W60A	CYS162(O)	4.64	VAL203(CA)	1W60A		
1W60A	CYS162(O)	4.51	VAL203(C)	1W60A		
1W60A	CYS162(O)	3.70	VAL203(O)	1W60A		O[ ] ... O[ ]
1W60A	CYS162(CB)	4.26	VAL203(N)	1W60A		C[ ] ... N[ ]

1W60A	CYS162(CB)	4.85	VAL203(CA)	1W60A		
1W60A	CYS162(CB)	4.71	VAL203(C)	1W60A		
1W60A	CYS162(CB)	3.82	VAL203(O)	1W60A		C[ ] ... O[ ]
1W60A	CYS162(CB)	4.90	VAL203(CB)	1W60A		
1W60A	CYS162(CB)	4.98	VAL203(CG2)	1W60A		
1W60A	CYS162(CB)	4.90	MET229(SD)	1W60A		
1W60A	CYS162(CB)	3.84	MET229(CE)	1W60A		C[ ] ... C[ ]
1W60A	CYS162(SG)	3.73	MET229(CE)	1W60A		S[ ] ... C[ ]

chain1	res1/atm1	distance	res2/atm2	chain2	H-bonding	Charge interaction
1RK4A	CYS191(N)	4.55	PHE114(CE1)	1RK4A		
1RK4A	CYS191(SG)	4.82	PHE114(CG)	1RK4A		
1RK4A	CYS191(SG)	3.58	PHE114(CD1)	1RK4A		S[ ] ... C[ ]
1RK4A	CYS191(SG)	3.69	PHE114(CE1)	1RK4A		S[ ] ... C[ ]
1RK4A	CYS191(SG)	4.99	PHE114(CZ)	1RK4A		
1RK4A	CYS191(CB)	4.56	TYR117(CE2)	1RK4A		
1RK4A	CYS191(SG)	4.07	TYR117(CD2)	1RK4A		S[ ] ... C[ ]
1RK4A	CYS191(SG)	3.81	TYR117(CE2)	1RK4A		S[ ] ... C[ ]
1RK4A	CYS191(N)	4.56	ILE118(CD1)	1RK4A		
1RK4A	CYS191(CA)	4.52	ILE118(CD1)	1RK4A		
1RK4A	CYS191(SG)	4.41	ILE118(CD1)	1RK4A		S[ ] ... C[ ]
1RK4A	CYS191(SG)	4.19	LEU133(CD2)	1RK4A		S[ ] ... C[ ]
1RK4A	CYS191(N)	4.52	ILE186(O)	1RK4A	[D-A-AA]:144.6° [A-D-DD]:150.0° d_planarity:-0.4° a_planarity:16.4°, maximum distance exceeded	
1RK4A	CYS191(N)	4.07	VAL187(C)	1RK4A		N[ ] ... C[ ]
1RK4A	CYS191(N)	2.90	VAL187(O)	1RK4A	**H-bond** [D-A-AA]:158.9° [A-D-DD]:107.6° d_planarity:-3.1° a_planarity:86.6°	N[ ] ... O[ ]
1RK4A	CYS191(CA)	4.85	VAL187(C)	1RK4A		
1RK4A	CYS191(CA)	3.62	VAL187(O)	1RK4A		C[ ] ... O[ ]
1RK4A	CYS191(C)	4.65	VAL187(O)	1RK4A		
1RK4A	CYS191(CB)	4.34	VAL187(C)	1RK4A		C[ ] ... C[ ]
1RK4A	CYS191(CB)	3.20	VAL187(O)	1RK4A		C[ ] ... O[ ]
1RK4A	CYS191(SG)	4.64	VAL187(CA)	1RK4A		
1RK4A	CYS191(SG)	4.18	VAL187(C)	1RK4A		S[ ] ... C[ ]
1RK4A	CYS191(SG)	3.21	VAL187(O)	1RK4A		S[ ] ... O[ ]
1RK4A	CYS191(SG)	4.99	VAL187(CB)	1RK4A		
1RK4A	CYS191(SG)	4.03	VAL187(CG1)	1RK4A		S[ ] ... C[ ]
1RK4A	CYS191(N)	4.87	GLN188(N)	1RK4A		
1RK4A	CYS191(N)	4.81	GLN188(CA)	1RK4A		
1RK4A	CYS191(N)	4.08	GLN188(C)	1RK4A		N[ ] ... C[ ]
1RK4A	CYS191(N)	3.75	GLN188(O)	1RK4A	[D-A-AA]:96.4° [A-D-DD]:95.3° d_planarity:-63.4° a_planarity:79.8°, maximum distance exceeded, bad d_planarity	N[ ] ... O[ ]
1RK4A	CYS191(CA)	4.75	GLN188(C)	1RK4A		
1RK4A	CYS191(CA)	4.15	GLN188(O)	1RK4A		C[ ] ... O[ ]
1RK4A	CYS191(C)	4.89	GLN188(C)	1RK4A		
1RK4A	CYS191(C)	3.96	GLN188(O)	1RK4A		C[ ] ... O[ ]

1RK4A	CYS191(CB)	4.86	GLN188(CA)	1RK4A		
1RK4A	CYS191(CB)	4.73	GLN188(C)	1RK4A		
1RK4A	CYS191(CB)	4.26	GLN188(O)	1RK4A		C[ ] ... O[ ]
1RK4A	CYS191(N)	4.46	VAL189(N)	1RK4A		N[ ] ... N[ ]
1RK4A	CYS191(N)	4.59	VAL189(CA)	1RK4A		
1RK4A	CYS191(N)	3.48	VAL189(C)	1RK4A		N[ ] ... C[ ]
1RK4A	CYS191(N)	3.70	VAL189(O)	1RK4A	[D-A-AA]:70.5° [A-D-DD]:119.4° d_planarity:64.4° a_planarity:43.9°, maximum distance exceeded, bad a_angle(sp2), bad d_planarity	N[ ] ... O[ ]
1RK4A	CYS191(CA)	4.62	VAL189(C)	1RK4A		
1RK4A	CYS191(CA)	4.60	VAL189(O)	1RK4A		
1RK4A	CYS191(C)	4.65	VAL189(C)	1RK4A		
1RK4A	CYS191(C)	4.27	VAL189(O)	1RK4A		C[ ] ... O[ ]
1RK4A	CYS191(N)	2.84	VAL190(N)	1RK4A		N[ ] ... N[ ]
1RK4A	CYS191(N)	2.45	VAL190(CA)	1RK4A		N[ ] ... C[ ]
1RK4A	CYS191(N)	2.24	VAL190(O)	1RK4A	[D-A-AA]:30.1° [A-D-DD]:92.0° d_planarity:3.2° a_planarity:0.9°, bad a_angle(sp2)	N[ ] ... O[ ]
1RK4A	CYS191(N)	3.13	VAL190(CB)	1RK4A		N[ ] ... C[ ]
1RK4A	CYS191(N)	3.63	VAL190(CG1)	1RK4A		N[ ] ... C[ ]
1RK4A	CYS191(N)	4.56	VAL190(CG2)	1RK4A		
1RK4A	CYS191(CA)	4.21	VAL190(N)	1RK4A		C[ ] ... N[ ]
1RK4A	CYS191(CA)	3.81	VAL190(CA)	1RK4A		C[ ] ... C[ ]
1RK4A	CYS191(CA)	2.41	VAL190(C)	1RK4A		C[ ] ... C[ ]
1RK4A	CYS191(CA)	2.72	VAL190(O)	1RK4A		C[ ] ... O[ ]
1RK4A	CYS191(CA)	4.49	VAL190(CB)	1RK4A		C[ ] ... C[ ]
1RK4A	CYS191(CA)	4.69	VAL190(CG1)	1RK4A		
1RK4A	CYS191(C)	4.66	VAL190(N)	1RK4A		
1RK4A	CYS191(C)	4.41	VAL190(CA)	1RK4A		C[ ] ... C[ ]
1RK4A	CYS191(C)	3.03	VAL190(C)	1RK4A		C[ ] ... C[ ]
1RK4A	CYS191(C)	2.98	VAL190(O)	1RK4A		C[ ] ... O[ ]
1RK4A	CYS191(O)	3.86	VAL190(C)	1RK4A		O[ ] ... C[ ]
1RK4A	CYS191(O)	3.45	VAL190(O)	1RK4A		O[ ] ... O[ ]
1RK4A	CYS191(CB)	4.91	VAL190(CA)	1RK4A		
1RK4A	CYS191(CB)	3.72	VAL190(C)	1RK4A		C[ ] ... C[ ]
1RK4A	CYS191(CB)	4.22	VAL190(O)	1RK4A		C[ ] ... O[ ]
1RK4A	CYS191(SG)	4.36	VAL190(C)	1RK4A		S[ ] ... C[ ]
1RK4A	CYS191(SG)	4.96	VAL190(O)	1RK4A		
1RK4A	CYS191(N)	2.90	LYS192(N)	1RK4A		N[ ] ... N[ ]
1RK4A	CYS191(N)	4.33	LYS192(CA)	1RK4A		N[ ] ... C[ ]
1RK4A	CYS191(N)	4.83	LYS192(C)	1RK4A		
1RK4A	CYS191(CA)	2.47	LYS192(N)	1RK4A		C[ ] ... N[ ]
1RK4A	CYS191(CA)	3.84	LYS192(CA)	1RK4A		C[ ] ... C[ ]
1RK4A	CYS191(CA)	4.45	LYS192(C)	1RK4A		C[ ] ... C[ ]
1RK4A	CYS191(CA)	4.93	LYS192(CB)	1RK4A		
1RK4A	CYS191(C)	2.43	LYS192(CA)	1RK4A		C[ ] ... C[ ]
1RK4A	CYS191(C)	3.03	LYS192(C)	1RK4A		C[ ] ... C[ ]
1RK4A	CYS191(C)	3.78	LYS192(O)	1RK4A		C[ ] ... O[ ]
1RK4A	CYS191(C)	3.72	LYS192(CB)	1RK4A		C[ ] ... C[ ]
1RK4A	CYS191(C)	4.95	LYS192(CG)	1RK4A		
1RK4A	CYS191(O)	2.23	LYS192(N)	1RK4A	[D-A-AA]:30.4° [A-D-DD]:93.1° d_planarity:0.0°	O[ ] ... N[ ]

					a_planarity:1.1°, bad a_angle(sp2)	
1RK4A	CYS191(O)	2.73	LYS192(CA)	1RK4A		O[ ] ... C[ ]
1RK4A	CYS191(O)	2.96	LYS192(C)	1RK4A		O[ ] ... C[ ]
1RK4A	CYS191(O)	3.31	LYS192(O)	1RK4A		O[ ] ... O[ ]
1RK4A	CYS191(O)	4.22	LYS192(CB)	1RK4A		O[ ] ... C[ ]
1RK4A	CYS191(CB)	3.25	LYS192(N)	1RK4A		C[ ] ... N[ ]
1RK4A	CYS191(CB)	4.56	LYS192(CA)	1RK4A		
1RK4A	CYS191(SG)	4.96	LYS192(N)	1RK4A	[D-A-AA]:16.5° [A-D-DD]:156.6° d_planarity:52.5°, maximum distance exceeded, bad a_angle(sp3)	
1RK4A	CYS191(N)	4.52	LYS193(N)	1RK4A		
1RK4A	CYS191(CA)	4.52	LYS193(N)	1RK4A		
1RK4A	CYS191(C)	3.38	LYS193(N)	1RK4A		C[ ] ... N[ ]
1RK4A	CYS191(C)	4.53	LYS193(CA)	1RK4A		
1RK4A	CYS191(C)	4.63	LYS193(C)	1RK4A		
1RK4A	CYS191(O)	3.56	LYS193(N)	1RK4A	[D-A-AA]:71.8° [A-D-DD]:122.2° d_planarity:64.2° a_planarity:44.4°, maximum distance exceeded, bad a_angle(sp2), bad d_planarity	O[ ] ... N[ ]
1RK4A	CYS191(O)	4.50	LYS193(CA)	1RK4A		
1RK4A	CYS191(O)	4.28	LYS193(C)	1RK4A		O[ ] ... C[ ]
1RK4A	CYS191(N)	4.86	TYR194(N)	1RK4A		
1RK4A	CYS191(CA)	4.72	TYR194(N)	1RK4A		
1RK4A	CYS191(C)	4.06	TYR194(N)	1RK4A		C[ ] ... N[ ]
1RK4A	CYS191(C)	4.94	TYR194(CA)	1RK4A		
1RK4A	CYS191(C)	4.86	TYR194(C)	1RK4A		
1RK4A	CYS191(O)	3.71	TYR194(N)	1RK4A	[D-A-AA]:97.7° [A-D-DD]:108.3° d_planarity:-54.8° a_planarity:84.9°, maximum distance exceeded	O[ ] ... N[ ]
1RK4A	CYS191(O)	4.40	TYR194(CA)	1RK4A		O[ ] ... C[ ]
1RK4A	CYS191(O)	4.04	TYR194(C)	1RK4A		O[ ] ... C[ ]
1RK4A	CYS191(CA)	4.54	ARG195(N)	1RK4A		
1RK4A	CYS191(CA)	4.47	ARG195(CB)	1RK4A		C[ ] ... C[ ]
1RK4A	CYS191(CA)	4.96	ARG195(CG)	1RK4A		
1RK4A	CYS191(C)	3.92	ARG195(N)	1RK4A		C[ ] ... N[ ]
1RK4A	CYS191(C)	4.53	ARG195(CA)	1RK4A		
1RK4A	CYS191(C)	4.72	ARG195(C)	1RK4A		
1RK4A	CYS191(C)	4.32	ARG195(CB)	1RK4A		C[ ] ... C[ ]
1RK4A	CYS191(O)	2.93	ARG195(N)	1RK4A	**H-bond** [D-A-AA]:138.3° [A-D-DD]:95.1° d_planarity:-18.4° a_planarity:-64.3°	O[ ] ... N[ ]
1RK4A	CYS191(O)	3.39	ARG195(CA)	1RK4A		O[ ] ... C[ ]
1RK4A	CYS191(O)	3.50	ARG195(C)	1RK4A		O[ ] ... C[ ]
1RK4A	CYS191(O)	4.51	ARG195(O)	1RK4A		
1RK4A	CYS191(O)	3.38	ARG195(CB)	1RK4A		O[ ] ... C[ ]
1RK4A	CYS191(O)	4.46	ARG195(CG)	1RK4A		O[ ] ... C[ ]
1RK4A	CYS191(CB)	4.93	ARG195(CB)	1RK4A		
1RK4A	CYS191(C)	4.25	GLY196(N)	1RK4A		C[ ] ... N[ ]
1RK4A	CYS191(C)	4.91	GLY196(CA)	1RK4A		
1RK4A	CYS191(C)	4.90	GLY196(C)	1RK4A		
1RK4A	CYS191(O)	3.08	GLY196(N)	1RK4A	[D-A-AA]:160.9° [A-D-DD]:112.6° d_planarity:-65.6° a_planarity:10.2°, bad d_planarity	O[ ] ... N[ ]
1RK4A	CYS191(O)	3.88	GLY196(CA)	1RK4A		O[ ] ... C[ ]
1RK4A	CYS191(O)	4.01	GLY196(C)	1RK4A		O[ ] ... C[ ]
1RK4A	CYS191(CA)	4.91	PHE197(N)	1RK4A		

1RK4A	CYS191(CA)	4.25	PHE197(O)	1RK4A		C[ ] ... O[ ]
1RK4A	CYS191(CA)	4.74	PHE197(CB)	1RK4A		
1RK4A	CYS191(C)	3.95	PHE197(N)	1RK4A		C[ ] ... N[ ]
1RK4A	CYS191(C)	4.52	PHE197(CA)	1RK4A		
1RK4A	CYS191(C)	4.41	PHE197(C)	1RK4A		C[ ] ... C[ ]
1RK4A	CYS191(C)	3.50	PHE197(O)	1RK4A		C[ ] ... O[ ]
1RK4A	CYS191(C)	4.51	PHE197(CB)	1RK4A		
1RK4A	CYS191(O)	3.18	PHE197(N)	1RK4A	**H-bond** [D-A-AA]:120.9° [A-D-DD]:116.5° d_planarity:-14.9° a_planarity:74.9°	O[ ] ... N[ ]
1RK4A	CYS191(O)	4.05	PHE197(CA)	1RK4A		O[ ] ... C[ ]
1RK4A	CYS191(O)	4.30	PHE197(C)	1RK4A		O[ ] ... C[ ]
1RK4A	CYS191(O)	3.66	PHE197(O)	1RK4A		O[ ] ... O[ ]
1RK4A	CYS191(O)	4.12	PHE197(CB)	1RK4A		O[ ] ... C[ ]
1RK4A	CYS191(CB)	4.86	PHE197(N)	1RK4A		
1RK4A	CYS191(CB)	4.68	PHE197(CA)	1RK4A		
1RK4A	CYS191(CB)	4.48	PHE197(C)	1RK4A		C[ ] ... C[ ]
1RK4A	CYS191(CB)	3.73	PHE197(O)	1RK4A		C[ ] ... O[ ]
1RK4A	CYS191(CB)	4.01	PHE197(CB)	1RK4A		C[ ] ... C[ ]
1RK4A	CYS191(CB)	4.34	PHE197(CG)	1RK4A		C[ ] ... C[ ]
1RK4A	CYS191(CB)	3.80	PHE197(CD1)	1RK4A		C[ ] ... C[ ]
1RK4A	CYS191(CB)	4.75	PHE197(CE1)	1RK4A		
1RK4A	CYS191(SG)	4.97	PHE197(CB)	1RK4A		
1RK4A	CYS191(SG)	4.96	PHE197(CG)	1RK4A		
1RK4A	CYS191(SG)	4.35	PHE197(CD1)	1RK4A		S[ ] ... C[ ]
1RK4A	CYS191(SG)	4.94	PHE197(CE1)	1RK4A		

chain1	res1/atm1	distance	res2/atm2	chain2	H-bonding	Charge interaction
2ALDA	CYS72(N)	4.74	GLY26(O)	2ALDA	[D-A-AA]:117.1° [A-D-DD]:46.0° d_planarity:13.8° a_planarity:69.3°, maximum distance exceeded, bad d_angle(sp2)	
2ALDA	CYS72(CA)	4.76	GLY26(C)	2ALDA		
2ALDA	CYS72(CA)	3.87	GLY26(O)	2ALDA		C[ ] ... O[ ]
2ALDA	CYS72(C)	4.90	GLY26(C)	2ALDA		
2ALDA	CYS72(C)	4.14	GLY26(O)	2ALDA		C[ ] ... O[ ]
2ALDA	CYS72(O)	4.49	GLY26(C)	2ALDA		O[ ] ... C[ ]
2ALDA	CYS72(O)	3.73	GLY26(O)	2ALDA		O[ ] ... O[ ]
2ALDA	CYS72(CB)	4.79	GLY26(O)	2ALDA		
2ALDA	CYS72(CA)	4.62	LYS27(CA)	2ALDA		
2ALDA	CYS72(C)	4.94	LYS27(N)	2ALDA		
2ALDA	CYS72(C)	3.99	LYS27(CA)	2ALDA		C[ ] ... C[ ]
2ALDA	CYS72(C)	4.64	LYS27(C)	2ALDA		
2ALDA	CYS72(C)	4.29	LYS27(CB)	2ALDA		C[ ] ... C[ ]
2ALDA	CYS72(C)	4.69	LYS27(CG)	2ALDA		
2ALDA	CYS72(O)	4.41	LYS27(N)	2ALDA	[D-A-AA]:108.6° [A-D-DD]:35.3° d_planarity:2.9° a_planarity:-66.7°, maximum distance exceeded, bad d_angle(sp2)	O[ ] ... N[ ]
2ALDA	CYS72(O)	3.33	LYS27(CA)	2ALDA		O[ ] ... C[ ]
2ALDA	CYS72(O)	3.67	LYS27(C)	2ALDA		O[ ] ... C[ ]

2ALDA	CYS72(O)	4.86	LYS27(O)	2ALDA		
2ALDA	CYS72(O)	3.88	LYS27(CB)	2ALDA		O[ ] ... C[ ]
2ALDA	CYS72(O)	4.64	LYS27(CG)	2ALDA		
2ALDA	CYS72(C)	4.04	GLY28(N)	2ALDA		C[ ] ... N[ ]
2ALDA	CYS72(C)	4.61	GLY28(O)	2ALDA		
2ALDA	CYS72(O)	2.93	GLY28(N)	2ALDA	**H-bond** [D-A-AA]:149.9° [A-D-DD]:124.2° d_planarity:-16.0° a_planarity:64.1°	O[ ] ... N[ ]
2ALDA	CYS72(O)	3.95	GLY28(CA)	2ALDA		O[ ] ... C[ ]
2ALDA	CYS72(O)	4.09	GLY28(C)	2ALDA		O[ ] ... C[ ]
2ALDA	CYS72(O)	3.82	GLY28(O)	2ALDA		O[ ] ... O[ ]
2ALDA	CYS72(N)	4.74	ARG68(O)	2ALDA	[D-A-AA]:122.3° [A-D-DD]:104.8° d_planarity:-39.9° a_planarity:34.2°, maximum distance exceeded	
2ALDA	CYS72(CB)	4.54	ARG68(O)	2ALDA		
2ALDA	CYS72(SG)	4.68	ARG68(C)	2ALDA		
2ALDA	CYS72(SG)	3.55	ARG68(O)	2ALDA	Weakly positive	S[ ] ... O[ ]
2ALDA	CYS72(N)	4.61	VAL69(CA)	2ALDA		
2ALDA	CYS72(N)	3.69	VAL69(C)	2ALDA		N[ ] ... C[ ]
2ALDA	CYS72(N)	2.82	VAL69(O)	2ALDA	**H-bond** [D-A-AA]:126.6° [A-D-DD]:100.8° d_planarity:-19.6° a_planarity:78.8°	N[ ] ... O[ ]
2ALDA	CYS72(CA)	4.44	VAL69(C)	2ALDA		C[ ] ... C[ ]
2ALDA	CYS72(CA)	3.41	VAL69(O)	2ALDA		C[ ] ... O[ ]
2ALDA	CYS72(C)	4.96	VAL69(C)	2ALDA		
2ALDA	CYS72(C)	3.75	VAL69(O)	2ALDA		C[ ] ... O[ ]
2ALDA	CYS72(O)	4.90	VAL69(O)	2ALDA		
2ALDA	CYS72(CB)	4.30	VAL69(CA)	2ALDA		C[ ] ... C[ ]
2ALDA	CYS72(CB)	4.10	VAL69(C)	2ALDA		C[ ] ... C[ ]
2ALDA	CYS72(CB)	3.25	VAL69(O)	2ALDA		C[ ] ... O[ ]
2ALDA	CYS72(SG)	4.41	VAL69(CA)	2ALDA		S[ ] ... C[ ]
2ALDA	CYS72(SG)	4.54	VAL69(C)	2ALDA		
2ALDA	CYS72(SG)	4.11	VAL69(O)	2ALDA		S[ ] ... O[ ]
2ALDA	CYS72(N)	4.20	ASN70(N)	2ALDA		N[ ] ... N[ ]
2ALDA	CYS72(N)	4.17	ASN70(CA)	2ALDA		N[ ] ... C[ ]
2ALDA	CYS72(N)	3.17	ASN70(C)	2ALDA		N[ ] ... C[ ]
2ALDA	CYS72(N)	3.42	ASN70(O)	2ALDA	[D-A-AA]:67.9° [A-D-DD]:136.8° d_planarity:-86.0° a_planarity:51.7°, bad a_angle(sp2), bad d_planarity	N[ ] ... O[ ]
2ALDA	CYS72(CA)	4.51	ASN70(C)	2ALDA		
2ALDA	CYS72(CA)	4.60	ASN70(O)	2ALDA		
2ALDA	CYS72(C)	4.91	ASN70(C)	2ALDA		
2ALDA	CYS72(C)	4.65	ASN70(O)	2ALDA		
2ALDA	CYS72(N)	2.62	PRO71(N)	2ALDA		N[ ] ... N[ ]
2ALDA	CYS72(N)	2.44	PRO71(CA)	2ALDA		N[ ] ... C[ ]
2ALDA	CYS72(N)	2.29	PRO71(O)	2ALDA	[D-A-AA]:28.6° [A-D-DD]:93.6° d_planarity:4.0° a_planarity:11.7°, bad a_angle(sp2)	N[ ] ... O[ ]
2ALDA	CYS72(N)	3.31	PRO71(CB)	2ALDA		N[ ] ... C[ ]
2ALDA	CYS72(N)	3.93	PRO71(CG)	2ALDA		N[ ] ... C[ ]
2ALDA	CYS72(N)	3.42	PRO71(CD)	2ALDA		N[ ] ... C[ ]
2ALDA	CYS72(CA)	4.08	PRO71(N)	2ALDA		C[ ] ... N[ ]
2ALDA	CYS72(CA)	3.80	PRO71(CA)	2ALDA		C[ ] ... C[ ]
2ALDA	CYS72(CA)	2.43	PRO71(C)	2ALDA		C[ ] ... C[ ]
2ALDA	CYS72(CA)	2.79	PRO71(O)	2ALDA		C[ ] ... O[ ]

2ALDA	CYS72(CA)	4.52	PRO71(CB)	2ALDA		
2ALDA	CYS72(CA)	4.80	PRO71(CD)	2ALDA		
2ALDA	CYS72(C)	4.85	PRO71(N)	2ALDA		
2ALDA	CYS72(C)	4.61	PRO71(CA)	2ALDA		
2ALDA	CYS72(C)	3.26	PRO71(C)	2ALDA		C[ ] ... C[ ]
2ALDA	CYS72(C)	3.51	PRO71(O)	2ALDA		C[ ] ... O[ ]
2ALDA	CYS72(O)	4.23	PRO71(C)	2ALDA		O[ ] ... C[ ]
2ALDA	CYS72(O)	4.17	PRO71(O)	2ALDA		O[ ] ... O[ ]
2ALDA	CYS72(CB)	4.79	PRO71(N)	2ALDA		
2ALDA	CYS72(CB)	4.86	PRO71(CA)	2ALDA		
2ALDA	CYS72(CB)	3.69	PRO71(C)	2ALDA		C[ ] ... C[ ]
2ALDA	CYS72(CB)	4.20	PRO71(O)	2ALDA		C[ ] ... O[ ]
2ALDA	CYS72(SG)	4.15	PRO71(C)	2ALDA		S[ ] ... C[ ]
2ALDA	CYS72(SG)	4.62	PRO71(O)	2ALDA		
2ALDA	CYS72(SG)	4.89	PRO71(CD)	2ALDA		
2ALDA	CYS72(N)	2.80	ILE73(N)	2ALDA		N[ ] ... N[ ]
2ALDA	CYS72(N)	4.25	ILE73(CA)	2ALDA		N[ ] ... C[ ]
2ALDA	CYS72(N)	4.91	ILE73(C)	2ALDA		
2ALDA	CYS72(N)	4.97	ILE73(CB)	2ALDA		
2ALDA	CYS72(CA)	2.44	ILE73(N)	2ALDA		C[ ] ... N[ ]
2ALDA	CYS72(CA)	3.81	ILE73(CA)	2ALDA		C[ ] ... C[ ]
2ALDA	CYS72(CA)	4.76	ILE73(C)	2ALDA		
2ALDA	CYS72(CA)	4.65	ILE73(CB)	2ALDA		
2ALDA	CYS72(CA)	4.61	ILE73(CG1)	2ALDA		
2ALDA	CYS72(CA)	4.69	ILE73(CD1)	2ALDA		
2ALDA	CYS72(C)	2.41	ILE73(CA)	2ALDA		C[ ] ... C[ ]
2ALDA	CYS72(C)	3.42	ILE73(C)	2ALDA		C[ ] ... C[ ]
2ALDA	CYS72(C)	4.18	ILE73(O)	2ALDA		C[ ] ... O[ ]
2ALDA	CYS72(C)	3.48	ILE73(CB)	2ALDA		C[ ] ... C[ ]
2ALDA	CYS72(C)	3.66	ILE73(CG1)	2ALDA		C[ ] ... C[ ]
2ALDA	CYS72(C)	4.82	ILE73(CG2)	2ALDA		
2ALDA	CYS72(C)	3.70	ILE73(CD1)	2ALDA		C[ ] ... C[ ]
2ALDA	CYS72(O)	2.23	ILE73(N)	2ALDA	[D-A-AA]:30.8° [A-D-DD]:90.4° d_planarity:0.2° a_planarity:7.7°, bad a_angle(sp2)	O[ ] ... N[ ]
2ALDA	CYS72(O)	2.68	ILE73(CA)	2ALDA		O[ ] ... C[ ]
2ALDA	CYS72(O)	3.65	ILE73(C)	2ALDA		O[ ] ... C[ ]
2ALDA	CYS72(O)	4.66	ILE73(O)	2ALDA		
2ALDA	CYS72(O)	3.88	ILE73(CB)	2ALDA		O[ ] ... C[ ]
2ALDA	CYS72(O)	3.97	ILE73(CG1)	2ALDA		O[ ] ... C[ ]
2ALDA	CYS72(O)	3.59	ILE73(CD1)	2ALDA		O[ ] ... C[ ]
2ALDA	CYS72(CB)	3.16	ILE73(N)	2ALDA		C[ ] ... N[ ]
2ALDA	CYS72(CB)	4.40	ILE73(CA)	2ALDA		C[ ] ... C[ ]
2ALDA	CYS72(CB)	4.83	ILE73(CB)	2ALDA		
2ALDA	CYS72(CB)	4.30	ILE73(CG1)	2ALDA		C[ ] ... C[ ]
2ALDA	CYS72(CB)	4.24	ILE73(CD1)	2ALDA		C[ ] ... C[ ]
2ALDA	CYS72(SG)	4.85	ILE73(N)	2ALDA	[D-A-AA]:18.0° [A-D-DD]:150.5° d_planarity:57.8°, maximum distance exceeded, bad a_angle(sp3)	
2ALDA	CYS72(C)	4.00	GLY74(N)	2ALDA		C[ ] ... N[ ]
2ALDA	CYS72(O)	3.86	GLY74(N)	2ALDA	[D-A-AA]:87.4° [A-D-DD]:153.9° d_planarity:-74.1° a_planarity:25.5°, maximum distance exceeded,	O[ ] ... N[ ]

					bad a_angle(sp2), bad d_planarity	
2ALDA	CYS72(C)	4.65	PHE299(CZ)	2ALDA		
2ALDA	CYS72(O)	4.31	PHE299(CE1)	2ALDA		O[ ] ... C[ ]
2ALDA	CYS72(O)	4.45	PHE299(CE2)	2ALDA		O[ ] ... C[ ]
2ALDA	CYS72(O)	3.60	PHE299(CZ)	2ALDA		O[ ] ... C[ ]
2ALDA	CYS72(CA)	4.96	ALA331(O)	2ALDA		
2ALDA	CYS72(CB)	4.69	ALA331(CA)	2ALDA		
2ALDA	CYS72(CB)	4.20	ALA331(C)	2ALDA		C[ ] ... C[ ]
2ALDA	CYS72(CB)	3.80	ALA331(O)	2ALDA		C[ ] ... O[ ]
2ALDA	CYS72(CB)	4.11	ALA331(CB)	2ALDA		C[ ] ... C[ ]
2ALDA	CYS72(SG)	4.88	ALA331(CA)	2ALDA		
2ALDA	CYS72(SG)	3.93	ALA331(C)	2ALDA		S[ ] ... C[ ]
2ALDA	CYS72(SG)	3.67	ALA331(O)	2ALDA		S[ ] ... O[ ]
2ALDA	CYS72(SG)	4.55	ALA331(CB)	2ALDA		
2ALDA	CYS72(CB)	4.98	LEU332(N)	2ALDA		
2ALDA	CYS72(SG)	4.24	LEU332(N)	2ALDA	[D-A-AA]:102.9° [A-D-DD]:70.8° d_planarity:49.4°, maximum distance exceeded, bad d_angle(sp2)	S[ ] ... N[ ]
2ALDA	CYS72(SG)	4.01	LEU332(CA)	2ALDA		S[ ] ... C[ ]
2ALDA	CYS72(SG)	4.93	LEU332(C)	2ALDA		
2ALDA	CYS72(SG)	4.88	LEU332(O)	2ALDA		
2ALDA	CYS72(SG)	4.88	LEU332(CB)	2ALDA		
2ALDA	CYS72(SG)	4.46	LEU332(CG)	2ALDA		S[ ] ... C[ ]
2ALDA	CYS72(SG)	3.60	LEU332(CD2)	2ALDA		S[ ] ... C[ ]
2ALDA	CYS72(N)	4.80	SER335(OG)	2ALDA	[D-A-AA]:94.1° [A-D-DD]:51.9° d_planarity:55.2°, maximum distance exceeded, bad d_angle(sp2)	
2ALDA	CYS72(CA)	4.09	SER335(CB)	2ALDA		C[ ] ... C[ ]
2ALDA	CYS72(CA)	4.06	SER335(OG)	2ALDA		C[ ] ... O[ ]
2ALDA	CYS72(C)	5.00	SER335(CB)	2ALDA		
2ALDA	CYS72(O)	4.85	SER335(CB)	2ALDA		
2ALDA	CYS72(CB)	3.93	SER335(CB)	2ALDA		C[ ] ... C[ ]
2ALDA	CYS72(CB)	4.36	SER335(OG)	2ALDA		C[ ] ... O[ ]
2ALDA	CYS72(SG)	4.86	SER335(CA)	2ALDA		
2ALDA	CYS72(SG)	3.48	SER335(CB)	2ALDA		S[ ] ... C[ ]
2ALDA	CYS72(SG)	3.87	SER335(OG)	2ALDA	[D-A-AA]:92.9° [A-D-DD]:63.4°, maximum distance exceeded	S[ ] ... O[ ]

chain1	res1/atm1	distance	res2/atm2	chain2	H-bonding	Charge interaction
2ALDA	CYS239(N)	4.81	GLY194(C)	2ALDA		
2ALDA	CYS239(N)	3.99	GLY194(O)	2ALDA	[D-A-AA]:125.2° [A-D-DD]:124.9° d_planarity:-69.8° a_planarity:19.5°, maximum distance exceeded, bad d_planarity	N[ ] ... O[ ]
2ALDA	CYS239(CA)	4.97	GLY194(O)	2ALDA		
2ALDA	CYS239(N)	4.81	ASP195(N)	2ALDA		
2ALDA	CYS239(N)	3.83	ASP195(CA)	2ALDA		N[ ] ... C[ ]
2ALDA	CYS239(N)	4.44	ASP195(C)	2ALDA		N[ ] ... C[ ]
2ALDA	CYS239(N)	4.58	ASP195(O)	2ALDA	[D-A-AA]:75.8° [A-D-DD]:60.6° d_planarity:89.8° a_planarity:-51.2°, maximum distance exceeded, bad a_angle(sp2), bad d_angle(sp2), bad d_planarity	

2ALDA	CYS239(N)	4.16	ASP195(CB)	2ALDA		N[ ] ... C[ ]
2ALDA	CYS239(CA)	4.13	ASP195(CA)	2ALDA		C[ ] ... C[ ]
2ALDA	CYS239(CA)	4.30	ASP195(C)	2ALDA		C[ ] ... C[ ]
2ALDA	CYS239(CA)	4.07	ASP195(O)	2ALDA		C[ ] ... O[ ]
2ALDA	CYS239(CA)	4.32	ASP195(CB)	2ALDA		C[ ] ... C[ ]
2ALDA	CYS239(CB)	4.62	ASP195(CA)	2ALDA		
2ALDA	CYS239(CB)	4.37	ASP195(C)	2ALDA		C[ ] ... C[ ]
2ALDA	CYS239(CB)	4.16	ASP195(O)	2ALDA		C[ ] ... O[ ]
2ALDA	CYS239(SG)	4.95	ASP195(C)	2ALDA		
2ALDA	CYS239(SG)	4.33	ASP195(O)	2ALDA	Weakly negative	S[ ] ... O[ ]
2ALDA	CYS239(CA)	4.62	HIS196(O)	2ALDA		
2ALDA	CYS239(CB)	4.88	HIS196(N)	2ALDA		
2ALDA	CYS239(CB)	4.32	HIS196(C)	2ALDA		C[ ] ... C[ ]
2ALDA	CYS239(CB)	3.54	HIS196(O)	2ALDA		C[ ] ... O[ ]
2ALDA	CYS239(SG)	4.24	HIS196(C)	2ALDA		S[ ] ... C[ ]
2ALDA	CYS239(SG)	3.81	HIS196(O)	2ALDA	Weakly positive	S[ ] ... O[ ]
2ALDA	CYS239(CB)	4.91	ASP197(N)	2ALDA		
2ALDA	CYS239(CB)	4.78	ASP197(CA)	2ALDA		
2ALDA	CYS239(SG)	4.34	ASP197(N)	2ALDA	[D-A-AA]:97.3° [A-D-DD]:61.9° d_planarity:49.2°, maximum distance exceeded, bad d_angle(sp2)	S[ ] ... N[ ]
2ALDA	CYS239(SG)	3.87	ASP197(CA)	2ALDA		S[ ] ... C[ ]
2ALDA	CYS239(SG)	4.22	ASP197(CB)	2ALDA	Negative	S[ ] ... C[ ]
2ALDA	CYS239(CB)	4.78	PRO235(CA)	2ALDA		
2ALDA	CYS239(CB)	4.66	PRO235(C)	2ALDA		
2ALDA	CYS239(CB)	3.90	PRO235(CB)	2ALDA		C[ ] ... C[ ]
2ALDA	CYS239(CB)	4.81	PRO235(CG)	2ALDA		
2ALDA	CYS239(SG)	4.10	PRO235(CB)	2ALDA		S[ ] ... C[ ]
2ALDA	CYS239(N)	4.94	GLY236(N)	2ALDA		
2ALDA	CYS239(N)	4.56	GLY236(CA)	2ALDA		
2ALDA	CYS239(N)	3.76	GLY236(C)	2ALDA		N[ ] ... C[ ]
2ALDA	CYS239(N)	3.11	GLY236(O)	2ALDA	**H-bond** [D-A-AA]:113.8° [A-D-DD]:106.5° d_planarity:-5.4° a_planarity:86.5°	N[ ] ... O[ ]
2ALDA	CYS239(CA)	4.58	GLY236(C)	2ALDA		
2ALDA	CYS239(CA)	3.79	GLY236(O)	2ALDA		C[ ] ... O[ ]
2ALDA	CYS239(C)	4.65	GLY236(O)	2ALDA		
2ALDA	CYS239(O)	4.38	GLY236(O)	2ALDA		O[ ] ... O[ ]
2ALDA	CYS239(CB)	4.06	GLY236(N)	2ALDA		C[ ] ... N[ ]
2ALDA	CYS239(CB)	4.45	GLY236(CA)	2ALDA		C[ ] ... C[ ]
2ALDA	CYS239(CB)	4.17	GLY236(C)	2ALDA		C[ ] ... C[ ]
2ALDA	CYS239(CB)	3.34	GLY236(O)	2ALDA		C[ ] ... O[ ]
2ALDA	CYS239(N)	4.31	HIS237(N)	2ALDA		N[ ] ... N[ ]
2ALDA	CYS239(N)	4.39	HIS237(CA)	2ALDA		N[ ] ... C[ ]
2ALDA	CYS239(N)	3.31	HIS237(C)	2ALDA		N[ ] ... C[ ]
2ALDA	CYS239(N)	3.59	HIS237(O)	2ALDA	[D-A-AA]:66.8° [A-D-DD]:144.0° d_planarity:-68.9° a_planarity:44.2°, maximum distance exceeded, bad a_angle(sp2), bad d_planarity	N[ ] ... O[ ]
2ALDA	CYS239(CA)	4.68	HIS237(C)	2ALDA		
2ALDA	CYS239(CA)	4.85	HIS237(O)	2ALDA		
2ALDA	CYS239(O)	4.94	HIS237(C)	2ALDA		
2ALDA	CYS239(O)	4.52	HIS237(O)	2ALDA		

2ALDA	CYS239(N)	2.65	ALA238(N)	2ALDA		N[ ] ... N[ ]
2ALDA	CYS239(N)	2.43	ALA238(CA)	2ALDA		N[ ] ... C[ ]
2ALDA	CYS239(N)	2.27	ALA238(O)	2ALDA	[D-A-AA]:29.0° [A-D-DD]:94.4° d_planarity:6.0° a_planarity:12.7°, bad a_angle(sp2)	N[ ] ... O[ ]
2ALDA	CYS239(N)	3.53	ALA238(CB)	2ALDA		N[ ] ... C[ ]
2ALDA	CYS239(CA)	4.10	ALA238(N)	2ALDA		C[ ] ... N[ ]
2ALDA	CYS239(CA)	3.79	ALA238(CA)	2ALDA		C[ ] ... C[ ]
2ALDA	CYS239(CA)	2.42	ALA238(C)	2ALDA		C[ ] ... C[ ]
2ALDA	CYS239(CA)	2.79	ALA238(O)	2ALDA		C[ ] ... O[ ]
2ALDA	CYS239(CA)	4.74	ALA238(CB)	2ALDA		
2ALDA	CYS239(C)	4.89	ALA238(N)	2ALDA		
2ALDA	CYS239(C)	4.36	ALA238(CA)	2ALDA		C[ ] ... C[ ]
2ALDA	CYS239(C)	2.99	ALA238(C)	2ALDA		C[ ] ... C[ ]
2ALDA	CYS239(C)	3.12	ALA238(O)	2ALDA		C[ ] ... O[ ]
2ALDA	CYS239(O)	4.88	ALA238(N)	2ALDA	[D-A-AA]:83.4° [A-D-DD]:66.5° d_planarity:66.3° a_planarity:-38.1°, maximum distance exceeded, bad a_angle(sp2), bad d_angle(sp2), bad d_planarity	
2ALDA	CYS239(O)	4.50	ALA238(CA)	2ALDA		
2ALDA	CYS239(O)	3.41	ALA238(C)	2ALDA		O[ ] ... C[ ]
2ALDA	CYS239(O)	3.80	ALA238(O)	2ALDA		O[ ] ... O[ ]
2ALDA	CYS239(CB)	4.80	ALA238(N)	2ALDA		
2ALDA	CYS239(CB)	4.90	ALA238(CA)	2ALDA		
2ALDA	CYS239(CB)	3.74	ALA238(C)	2ALDA		C[ ] ... C[ ]
2ALDA	CYS239(CB)	4.29	ALA238(O)	2ALDA		C[ ] ... O[ ]
2ALDA	CYS239(N)	3.50	THR240(N)	2ALDA		N[ ] ... N[ ]
2ALDA	CYS239(N)	4.73	THR240(CA)	2ALDA		
2ALDA	CYS239(CA)	2.46	THR240(N)	2ALDA		C[ ] ... N[ ]
2ALDA	CYS239(CA)	3.82	THR240(CA)	2ALDA		C[ ] ... C[ ]
2ALDA	CYS239(CA)	4.60	THR240(C)	2ALDA		
2ALDA	CYS239(CA)	4.89	THR240(CB)	2ALDA		
2ALDA	CYS239(CA)	4.99	THR240(OG1)	2ALDA		
2ALDA	CYS239(C)	2.41	THR240(CA)	2ALDA		C[ ] ... C[ ]
2ALDA	CYS239(C)	3.26	THR240(C)	2ALDA		C[ ] ... C[ ]
2ALDA	CYS239(C)	4.13	THR240(O)	2ALDA		C[ ] ... O[ ]
2ALDA	CYS239(C)	3.67	THR240(CB)	2ALDA		C[ ] ... C[ ]
2ALDA	CYS239(C)	4.08	THR240(OG1)	2ALDA		C[ ] ... O[ ]
2ALDA	CYS239(C)	4.05	THR240(CG2)	2ALDA		C[ ] ... C[ ]
2ALDA	CYS239(O)	2.22	THR240(N)	2ALDA	[D-A-AA]:29.7° [A-D-DD]:94.1° d_planarity:2.4° a_planarity:7.7°, bad a_angle(sp2)	O[ ] ... N[ ]
2ALDA	CYS239(O)	2.74	THR240(CA)	2ALDA		O[ ] ... C[ ]
2ALDA	CYS239(O)	3.34	THR240(C)	2ALDA		O[ ] ... C[ ]
2ALDA	CYS239(O)	3.90	THR240(O)	2ALDA		O[ ] ... O[ ]
2ALDA	CYS239(O)	4.17	THR240(CB)	2ALDA		O[ ] ... C[ ]
2ALDA	CYS239(O)	4.88	THR240(OG1)	2ALDA	[D-A-AA]:43.7° [A-D-DD]:52.6° a_planarity:3.7°, maximum distance exceeded, bad a_angle(sp2), bad d_angle(sp3)	
2ALDA	CYS239(O)	4.52	THR240(CG2)	2ALDA		
2ALDA	CYS239(CB)	3.35	THR240(N)	2ALDA		C[ ] ... N[ ]
2ALDA	CYS239(CB)	4.57	THR240(CA)	2ALDA		
2ALDA	CYS239(CB)	4.89	THR240(C)	2ALDA		
2ALDA	CYS239(SG)	3.46	THR240(N)	2ALDA	[D-A-AA]:71.3° [A-D-DD]:126.8° d_planarity:83.6°, bad d_planarity	S[ ] ... N[ ]

2ALDA	CYS239(SG)	4.48	THR240(CA)	2ALDA		S[ ] ... C[ ]
2ALDA	CYS239(SG)	4.37	THR240(C)	2ALDA		S[ ] ... C[ ]
2ALDA	CYS239(CA)	4.62	GLN241(N)	2ALDA		
2ALDA	CYS239(C)	3.58	GLN241(N)	2ALDA		C[ ] ... N[ ]
2ALDA	CYS239(C)	4.81	GLN241(CA)	2ALDA		
2ALDA	CYS239(C)	4.98	GLN241(C)	2ALDA		
2ALDA	CYS239(C)	4.28	GLN241(O)	2ALDA		C[ ] ... O[ ]
2ALDA	CYS239(O)	3.84	GLN241(N)	2ALDA	[D-A-AA]:68.7° [A-D-DD]:128.9° d_planarity:73.9° a_planarity:49.0°, maximum distance exceeded, bad a_angle(sp2), bad d_planarity	O[ ] ... N[ ]
2ALDA	CYS239(O)	4.88	GLN241(CA)	2ALDA		
2ALDA	CYS239(O)	4.69	GLN241(C)	2ALDA		
2ALDA	CYS239(O)	3.85	GLN241(O)	2ALDA		O[ ] ... O[ ]
2ALDA	CYS239(CB)	4.57	GLN241(N)	2ALDA		
2ALDA	CYS239(CB)	4.50	GLN241(O)	2ALDA		
2ALDA	CYS239(SG)	3.61	GLN241(N)	2ALDA	**H-bond** [D-A-AA]:110.4° [A-D-DD]:105.1° d_planarity:-41.4°	S[ ] ... N[ ]
2ALDA	CYS239(SG)	4.22	GLN241(CA)	2ALDA		S[ ] ... C[ ]
2ALDA	CYS239(SG)	4.53	GLN241(C)	2ALDA		
2ALDA	CYS239(SG)	3.92	GLN241(O)	2ALDA		S[ ] ... O[ ]
2ALDA	CYS239(SG)	3.92	GLN241(CB)	2ALDA		S[ ] ... C[ ]
2ALDA	CYS239(SG)	4.32	GLN241(CG)	2ALDA		S[ ] ... C[ ]
2ALDA	CYS239(O)	4.52	LYS242(NZ)	2ALDA	[D-A-AA]:156.3° [A-D-DD]:117.9° a_planarity:15.7°, maximum distance exceeded	
2ALDA	CYS239(SG)	4.43	PHE243(CE2)	2ALDA		S[ ] ... C[ ]

chain1	res1/atm1	distance	res2/atm2	chain2	H-bonding	Charge interaction
2ALDA	CYS338(SG)	3.97	GLY26(N)	2ALDA	[D-A-AA]:145.6° [A-D-DD]:71.9° d_planarity:-25.2°, bad d_angle(sp2)	S[ ] ... N[ ]
2ALDA	CYS338(SG)	3.78	GLY26(CA)	2ALDA		S[ ] ... C[ ]
2ALDA	CYS338(SG)	3.63	GLY26(C)	2ALDA		S[ ] ... C[ ]
2ALDA	CYS338(SG)	3.73	GLY26(O)	2ALDA		S[ ] ... O[ ]
2ALDA	CYS338(CB)	4.93	LYS27(O)	2ALDA		
2ALDA	CYS338(SG)	3.93	LYS27(N)	2ALDA	[D-A-AA]:154.0° [A-D-DD]:110.0° d_planarity:74.6°, bad d_planarity	S[ ] ... N[ ]
2ALDA	CYS338(SG)	4.63	LYS27(CA)	2ALDA		
2ALDA	CYS338(SG)	4.34	LYS27(C)	2ALDA		S[ ] ... C[ ]
2ALDA	CYS338(SG)	3.98	LYS27(O)	2ALDA	Weakly positive	S[ ] ... O[ ]
2ALDA	CYS338(N)	4.09	LEU283(CG)	2ALDA		N[ ] ... C[ ]
2ALDA	CYS338(N)	4.02	LEU283(CD2)	2ALDA		N[ ] ... C[ ]
2ALDA	CYS338(CA)	4.10	LEU283(CG)	2ALDA		C[ ] ... C[ ]
2ALDA	CYS338(CA)	4.69	LEU283(CD1)	2ALDA		
2ALDA	CYS338(CA)	4.16	LEU283(CD2)	2ALDA		C[ ] ... C[ ]
2ALDA	CYS338(CB)	4.47	LEU283(CG)	2ALDA		C[ ] ... C[ ]
2ALDA	CYS338(CB)	4.88	LEU283(CD1)	2ALDA		
2ALDA	CYS338(CB)	4.09	LEU283(CD2)	2ALDA		C[ ] ... C[ ]
2ALDA	CYS338(N)	4.97	ASN287(CG)	2ALDA		
2ALDA	CYS338(N)	4.43	ASN287(ND2)	2ALDA		N[ ] ... N[ ]

2ALDA	CYS338(CA)	4.73	ASN287(CB)	2ALDA		
2ALDA	CYS338(CA)	4.01	ASN287(CG)	2ALDA		C[ ] ... C[ ]
2ALDA	CYS338(CA)	4.32	ASN287(OD1)	2ALDA		C[ ] ... O[ ]
2ALDA	CYS338(CA)	3.52	ASN287(ND2)	2ALDA		C[ ] ... N[ ]
2ALDA	CYS338(C)	4.27	ASN287(CB)	2ALDA		C[ ] ... C[ ]
2ALDA	CYS338(C)	3.65	ASN287(CG)	2ALDA		C[ ] ... C[ ]
2ALDA	CYS338(C)	3.68	ASN287(OD1)	2ALDA		C[ ] ... O[ ]
2ALDA	CYS338(C)	3.69	ASN287(ND2)	2ALDA		C[ ] ... N[ ]
2ALDA	CYS338(O)	4.52	ASN287(CB)	2ALDA		
2ALDA	CYS338(O)	3.65	ASN287(CG)	2ALDA		O[ ] ... C[ ]
2ALDA	CYS338(O)	3.27	ASN287(OD1)	2ALDA		O[ ] ... O[ ]
2ALDA	CYS338(O)	3.87	ASN287(ND2)	2ALDA	[D-A-AA]:72.6° [A-D-DD]:70.7° d_planarity:57.3° a_planarity:-67.6°, maximum distance exceeded, bad a_angle(sp2), bad d_angle(sp2)	O[ ] ... N[ ]
2ALDA	CYS338(CB)	4.40	ASN287(ND2)	2ALDA		C[ ] ... N[ ]
2ALDA	CYS338(CA)	3.84	LEU297(O)	2ALDA		C[ ] ... O[ ]
2ALDA	CYS338(C)	4.26	LEU297(O)	2ALDA		C[ ] ... O[ ]
2ALDA	CYS338(O)	3.89	LEU297(O)	2ALDA		O[ ] ... O[ ]
2ALDA	CYS338(CB)	4.57	LEU297(C)	2ALDA		
2ALDA	CYS338(CB)	3.61	LEU297(O)	2ALDA		C[ ] ... O[ ]
2ALDA	CYS338(CA)	4.94	THR298(CA)	2ALDA		
2ALDA	CYS338(CA)	4.97	THR298(OG1)	2ALDA		
2ALDA	CYS338(CB)	4.75	THR298(N)	2ALDA		
2ALDA	CYS338(CB)	3.93	THR298(CA)	2ALDA		C[ ] ... C[ ]
2ALDA	CYS338(CB)	4.91	THR298(C)	2ALDA		
2ALDA	CYS338(CB)	3.96	THR298(CB)	2ALDA		C[ ] ... C[ ]
2ALDA	CYS338(CB)	3.50	THR298(OG1)	2ALDA		C[ ] ... O[ ]
2ALDA	CYS338(SG)	4.93	THR298(CA)	2ALDA		
2ALDA	CYS338(SG)	4.40	THR298(CB)	2ALDA		S[ ] ... C[ ]
2ALDA	CYS338(SG)	3.60	THR298(OG1)	2ALDA	**H-bond** [D-A-AA]:72.3° [A-D-DD]:114.7°	S[ ] ... O[ ]
2ALDA	CYS338(CB)	4.57	PHE299(CD1)	2ALDA		
2ALDA	CYS338(CB)	4.82	PHE299(CE1)	2ALDA		
2ALDA	CYS338(SG)	4.77	PHE299(CD1)	2ALDA		
2ALDA	CYS338(SG)	4.51	PHE299(CE1)	2ALDA		
2ALDA	CYS338(N)	4.93	ASN334(CA)	2ALDA		
2ALDA	CYS338(N)	4.11	ASN334(C)	2ALDA		N[ ] ... C[ ]
2ALDA	CYS338(N)	2.95	ASN334(O)	2ALDA	**H-bond** [D-A-AA]:154.0° [A-D-DD]:118.2° d_planarity:-26.4° a_planarity:-79.1°	N[ ] ... O[ ]
2ALDA	CYS338(CA)	3.89	ASN334(O)	2ALDA		C[ ] ... O[ ]
2ALDA	CYS338(CB)	4.85	ASN334(C)	2ALDA		
2ALDA	CYS338(CB)	3.66	ASN334(O)	2ALDA		C[ ] ... O[ ]
2ALDA	CYS338(SG)	4.74	ASN334(C)	2ALDA		
2ALDA	CYS338(SG)	3.76	ASN334(O)	2ALDA		S[ ] ... O[ ]
2ALDA	CYS338(N)	4.87	SER335(N)	2ALDA		
2ALDA	CYS338(N)	4.81	SER335(CA)	2ALDA		
2ALDA	CYS338(N)	4.14	SER335(C)	2ALDA		N[ ] ... C[ ]
2ALDA	CYS338(N)	3.76	SER335(O)	2ALDA	[D-A-AA]:99.2° [A-D-DD]:104.1° d_planarity:-37.4° a_planarity:85.9°, maximum distance exceeded	N[ ] ... O[ ]
2ALDA	CYS338(CA)	4.36	SER335(O)	2ALDA		C[ ] ... O[ ]
2ALDA	CYS338(C)	4.43	SER335(O)	2ALDA		C[ ] ... O[ ]

2ALDA	CYS338(CB)	4.97	SER335(C)	2ALDA		
2ALDA	CYS338(CB)	4.37	SER335(O)	2ALDA		C[ ] ... O[ ]
2ALDA	CYS338(SG)	4.90	SER335(N)	2ALDA	[D-A-AA]:98.2° [A-D-DD]:51.4° d_planarity:19.7°, maximum distance exceeded, bad d_angle(sp2)	
2ALDA	CYS338(SG)	4.15	SER335(CA)	2ALDA		S[ ] ... C[ ]
2ALDA	CYS338(SG)	4.15	SER335(C)	2ALDA		S[ ] ... C[ ]
2ALDA	CYS338(SG)	3.45	SER335(O)	2ALDA		S[ ] ... O[ ]
2ALDA	CYS338(N)	4.27	LEU336(N)	2ALDA		N[ ] ... N[ ]
2ALDA	CYS338(N)	4.33	LEU336(CA)	2ALDA		N[ ] ... C[ ]
2ALDA	CYS338(N)	3.17	LEU336(C)	2ALDA		N[ ] ... C[ ]
2ALDA	CYS338(N)	3.30	LEU336(O)	2ALDA	[D-A-AA]:73.2° [A-D-DD]:123.5° d_planarity:79.1° a_planarity:45.6°, bad a_angle(sp2), bad d_planarity	N[ ] ... O[ ]
2ALDA	CYS338(CA)	4.43	LEU336(C)	2ALDA		C[ ] ... C[ ]
2ALDA	CYS338(CA)	4.31	LEU336(O)	2ALDA		C[ ] ... O[ ]
2ALDA	CYS338(C)	4.59	LEU336(C)	2ALDA		
2ALDA	CYS338(C)	4.10	LEU336(O)	2ALDA		C[ ] ... O[ ]
2ALDA	CYS338(N)	2.81	ALA337(N)	2ALDA		N[ ] ... N[ ]
2ALDA	CYS338(N)	2.47	ALA337(CA)	2ALDA		N[ ] ... C[ ]
2ALDA	CYS338(N)	2.20	ALA337(O)	2ALDA	[D-A-AA]:31.2° [A-D-DD]:91.9° d_planarity:6.9° a_planarity:13.9°, bad a_angle(sp2)	N[ ] ... O[ ]
2ALDA	CYS338(N)	3.35	ALA337(CB)	2ALDA		N[ ] ... C[ ]
2ALDA	CYS338(CA)	4.27	ALA337(N)	2ALDA		C[ ] ... N[ ]
2ALDA	CYS338(CA)	3.85	ALA337(CA)	2ALDA		C[ ] ... C[ ]
2ALDA	CYS338(CA)	2.43	ALA337(C)	2ALDA		C[ ] ... C[ ]
2ALDA	CYS338(CA)	2.70	ALA337(O)	2ALDA		C[ ] ... O[ ]
2ALDA	CYS338(CA)	4.64	ALA337(CB)	2ALDA		
2ALDA	CYS338(C)	4.85	ALA337(N)	2ALDA		
2ALDA	CYS338(C)	4.55	ALA337(CA)	2ALDA		
2ALDA	CYS338(C)	3.17	ALA337(C)	2ALDA		C[ ] ... C[ ]
2ALDA	CYS338(C)	3.06	ALA337(O)	2ALDA		C[ ] ... O[ ]
2ALDA	CYS338(O)	4.36	ALA337(C)	2ALDA		O[ ] ... C[ ]
2ALDA	CYS338(O)	4.17	ALA337(O)	2ALDA		O[ ] ... O[ ]
2ALDA	CYS338(CB)	4.94	ALA337(CA)	2ALDA		
2ALDA	CYS338(CB)	3.70	ALA337(C)	2ALDA		C[ ] ... C[ ]
2ALDA	CYS338(CB)	4.17	ALA337(O)	2ALDA		C[ ] ... O[ ]
2ALDA	CYS338(SG)	4.79	ALA337(C)	2ALDA		
2ALDA	CYS338(N)	2.69	GLN339(N)	2ALDA		N[ ] ... N[ ]
2ALDA	CYS338(N)	4.13	GLN339(CA)	2ALDA		N[ ] ... C[ ]
2ALDA	CYS338(N)	4.69	GLN339(C)	2ALDA		
2ALDA	CYS338(N)	4.82	GLN339(CB)	2ALDA		
2ALDA	CYS338(CA)	2.44	GLN339(N)	2ALDA		C[ ] ... N[ ]
2ALDA	CYS338(CA)	3.84	GLN339(CA)	2ALDA		C[ ] ... C[ ]
2ALDA	CYS338(CA)	4.61	GLN339(C)	2ALDA		
2ALDA	CYS338(CA)	4.77	GLN339(CB)	2ALDA		
2ALDA	CYS338(CA)	4.90	GLN339(CG)	2ALDA		
2ALDA	CYS338(C)	2.47	GLN339(CA)	2ALDA		C[ ] ... C[ ]
2ALDA	CYS338(C)	3.41	GLN339(C)	2ALDA		C[ ] ... C[ ]
2ALDA	CYS338(C)	4.58	GLN339(O)	2ALDA		
2ALDA	CYS338(C)	3.56	GLN339(CB)	2ALDA		C[ ] ... C[ ]
2ALDA	CYS338(C)	3.81	GLN339(CG)	2ALDA		C[ ] ... C[ ]

2ALDA	CYS338(O)	2.21	GLN339(N)	2ALDA	[D-A-AA]:31.6° [A-D-DD]:97.9° d_planarity:11.8° a_planarity:21.3°, bad a_angle(sp2)	O[ ] ... N[ ]
2ALDA	CYS338(O)	2.81	GLN339(CA)	2ALDA		O[ ] ... C[ ]
2ALDA	CYS338(O)	3.89	GLN339(C)	2ALDA		O[ ] ... C[ ]
2ALDA	CYS338(O)	4.94	GLN339(O)	2ALDA		
2ALDA	CYS338(O)	3.86	GLN339(CB)	2ALDA		O[ ] ... C[ ]
2ALDA	CYS338(O)	3.84	GLN339(CG)	2ALDA		O[ ] ... C[ ]
2ALDA	CYS338(CB)	3.53	GLN339(N)	2ALDA		C[ ] ... N[ ]
2ALDA	CYS338(CB)	4.83	GLN339(CA)	2ALDA		
2ALDA	CYS338(SG)	3.90	GLN339(N)	2ALDA	[D-A-AA]:64.5° [A-D-DD]:130.0° d_planarity:68.5°, bad d_planarity	S[ ] ... N[ ]
2ALDA	CYS338(SG)	4.97	GLN339(CA)	2ALDA		
2ALDA	CYS338(SG)	4.44	GLN339(CG)	2ALDA		S[ ] ... C[ ]
2ALDA	CYS338(N)	4.20	GLY340(N)	2ALDA		N[ ] ... N[ ]
2ALDA	CYS338(CA)	4.36	GLY340(N)	2ALDA		C[ ] ... N[ ]
2ALDA	CYS338(C)	3.52	GLY340(N)	2ALDA		C[ ] ... N[ ]
2ALDA	CYS338(C)	4.87	GLY340(CA)	2ALDA		
2ALDA	CYS338(O)	4.30	GLY340(N)	2ALDA	[D-A-AA]:44.3° [A-D-DD]:152.6° d_planarity:-85.1° a_planarity:26.2°, maximum distance exceeded, bad a_angle(sp2), bad d_planarity	O[ ] ... N[ ]

chain1	res1/atm1	distance	res2/atm2	chain2	H-bonding	Charge interaction
3GR4A	CYS49(N)	4.06	ILE47(C)	3GR4A		N[ ] ... C[ ]
3GR4A	CYS49(N)	4.04	ILE47(O)	3GR4A	[D-A-AA]:81.9° [A-D-DD]:140.3° d_planarity:-88.6° a_planarity:30.6°, maximum distance exceeded, bad a_angle(sp2), bad d_planarity	N[ ] ... O[ ]
3GR4A	CYS49(N)	4.92	ILE47(CG2)	3GR4A		
3GR4A	CYS49(SG)	4.70	ILE47(C)	3GR4A		
3GR4A	CYS49(SG)	4.51	ILE47(O)	3GR4A		
3GR4A	CYS49(SG)	4.97	ILE47(CB)	3GR4A		
3GR4A	CYS49(SG)	3.72	ILE47(CG2)	3GR4A		S[ ] ... C[ ]
3GR4A	CYS49(N)	3.44	ILE48(N)	3GR4A		N[ ] ... N[ ]
3GR4A	CYS49(N)	2.44	ILE48(CA)	3GR4A		N[ ] ... C[ ]
3GR4A	CYS49(N)	2.26	ILE48(O)	3GR4A	[D-A-AA]:30.0° [A-D-DD]:94.0° d_planarity:1.0° a_planarity:1.4°, bad a_angle(sp2)	N[ ] ... O[ ]
3GR4A	CYS49(N)	3.47	ILE48(CB)	3GR4A		N[ ] ... C[ ]
3GR4A	CYS49(N)	4.77	ILE48(CG1)	3GR4A		
3GR4A	CYS49(N)	3.50	ILE48(CG2)	3GR4A		N[ ] ... C[ ]
3GR4A	CYS49(CA)	4.66	ILE48(N)	3GR4A		
3GR4A	CYS49(CA)	3.80	ILE48(CA)	3GR4A		C[ ] ... C[ ]
3GR4A	CYS49(CA)	2.43	ILE48(C)	3GR4A		C[ ] ... C[ ]
3GR4A	CYS49(CA)	2.77	ILE48(O)	3GR4A		C[ ] ... O[ ]
3GR4A	CYS49(CA)	4.72	ILE48(CB)	3GR4A		
3GR4A	CYS49(CA)	4.55	ILE48(CG2)	3GR4A		
3GR4A	CYS49(C)	4.70	ILE48(CA)	3GR4A		
3GR4A	CYS49(C)	3.46	ILE48(C)	3GR4A		C[ ] ... C[ ]
3GR4A	CYS49(C)	3.80	ILE48(O)	3GR4A		C[ ] ... O[ ]

3GR4A	CYS49(C)	4.67	ILE48(CG2)	3GR4A		
3GR4A	CYS49(O)	4.98	ILE48(CA)	3GR4A		
3GR4A	CYS49(O)	4.02	ILE48(C)	3GR4A		O[ ] ... C[ ]
3GR4A	CYS49(O)	4.63	ILE48(O)	3GR4A		
3GR4A	CYS49(O)	4.73	ILE48(CG2)	3GR4A		
3GR4A	CYS49(CB)	4.71	ILE48(CA)	3GR4A		
3GR4A	CYS49(CB)	3.51	ILE48(C)	3GR4A		C[ ] ... C[ ]
3GR4A	CYS49(CB)	3.92	ILE48(O)	3GR4A		C[ ] ... O[ ]
3GR4A	CYS49(SG)	4.81	ILE48(N)	3GR4A	[D-A-AA]:96.1° [A-D-DD]:77.9° d_planarity:65.2°, maximum distance exceeded, bad d_angle(sp2), bad d_planarity	
3GR4A	CYS49(SG)	4.73	ILE48(CA)	3GR4A		
3GR4A	CYS49(SG)	3.76	ILE48(C)	3GR4A		S[ ] ... C[ ]
3GR4A	CYS49(SG)	4.08	ILE48(O)	3GR4A		S[ ] ... O[ ]
3GR4A	CYS49(N)	3.47	THR50(N)	3GR4A		N[ ] ... N[ ]
3GR4A	CYS49(N)	4.73	THR50(CA)	3GR4A		
3GR4A	CYS49(CA)	2.40	THR50(N)	3GR4A		C[ ] ... N[ ]
3GR4A	CYS49(CA)	3.76	THR50(CA)	3GR4A		C[ ] ... C[ ]
3GR4A	CYS49(CA)	4.37	THR50(C)	3GR4A		C[ ] ... C[ ]
3GR4A	CYS49(CA)	4.55	THR50(O)	3GR4A		
3GR4A	CYS49(CA)	4.83	THR50(CB)	3GR4A		
3GR4A	CYS49(CA)	4.88	THR50(OG1)	3GR4A		
3GR4A	CYS49(C)	2.41	THR50(CA)	3GR4A		C[ ] ... C[ ]
3GR4A	CYS49(C)	3.08	THR50(C)	3GR4A		C[ ] ... C[ ]
3GR4A	CYS49(C)	3.56	THR50(O)	3GR4A		C[ ] ... O[ ]
3GR4A	CYS49(C)	3.68	THR50(CB)	3GR4A		C[ ] ... C[ ]
3GR4A	CYS49(C)	3.93	THR50(OG1)	3GR4A		C[ ] ... O[ ]
3GR4A	CYS49(C)	4.89	THR50(CG2)	3GR4A		
3GR4A	CYS49(O)	2.23	THR50(N)	3GR4A	[D-A-AA]:29.5° [A-D-DD]:94.3° d_planarity:1.7° a_planarity:0.3°, bad a_angle(sp2)	O[ ] ... N[ ]
3GR4A	CYS49(O)	2.74	THR50(CA)	3GR4A		O[ ] ... C[ ]
3GR4A	CYS49(O)	3.21	THR50(C)	3GR4A		O[ ] ... C[ ]
3GR4A	CYS49(O)	3.95	THR50(O)	3GR4A		O[ ] ... O[ ]
3GR4A	CYS49(O)	4.19	THR50(CB)	3GR4A		O[ ] ... C[ ]
3GR4A	CYS49(O)	4.52	THR50(OG1)	3GR4A	[D-A-AA]:53.5° [A-D-DD]:67.4° a_planarity:28.9°, maximum distance exceeded, bad a_angle(sp2)	
3GR4A	CYS49(CB)	3.34	THR50(N)	3GR4A		C[ ] ... N[ ]
3GR4A	CYS49(CB)	4.56	THR50(CA)	3GR4A		
3GR4A	CYS49(CB)	4.68	THR50(C)	3GR4A		
3GR4A	CYS49(CB)	4.61	THR50(O)	3GR4A		
3GR4A	CYS49(SG)	4.96	THR50(N)	3GR4A	[D-A-AA]:22.1° [A-D-DD]:152.5° d_planarity:51.0°, maximum distance exceeded, bad a_angle(sp3)	
3GR4A	CYS49(CA)	4.95	ILE51(CD1)	3GR4A		
3GR4A	CYS49(C)	3.83	ILE51(N)	3GR4A		C[ ] ... N[ ]
3GR4A	CYS49(C)	4.95	ILE51(CA)	3GR4A		
3GR4A	CYS49(C)	4.59	ILE51(CG1)	3GR4A		
3GR4A	CYS49(C)	4.26	ILE51(CD1)	3GR4A		C[ ] ... C[ ]
3GR4A	CYS49(O)	3.54	ILE51(N)	3GR4A	[D-A-AA]:94.8° [A-D-DD]:128.1° d_planarity:81.3° a_planarity:30.6°, maximum distance exceeded, bad d_planarity	O[ ] ... N[ ]
3GR4A	CYS49(O)	4.60	ILE51(CA)	3GR4A		
3GR4A	CYS49(O)	4.77	ILE51(CB)	3GR4A		

3GR4A	CYS49(O)	3.79	ILE51(CG1)	3GR4A		O[ ] ... C[ ]
3GR4A	CYS49(O)	3.74	ILE51(CD1)	3GR4A		O[ ] ... C[ ]
3GR4A	CYS49(CB)	4.24	ILE51(CD1)	3GR4A		C[ ] ... C[ ]
3GR4A	CYS49(CB)	3.60	MET64(CE)	3GR4A		C[ ] ... C[ ]
3GR4A	CYS49(SG)	3.79	MET64(CE)	3GR4A		S[ ] ... C[ ]
3GR4A	CYS49(N)	4.61	MET69(CG)	3GR4A		
3GR4A	CYS49(CA)	4.90	MET69(CG)	3GR4A		
3GR4A	CYS49(CA)	4.92	MET69(SD)	3GR4A		
3GR4A	CYS49(O)	4.72	MET69(SD)	3GR4A		
3GR4A	CYS49(CB)	4.64	MET69(CB)	3GR4A		
3GR4A	CYS49(CB)	3.93	MET69(CG)	3GR4A		C[ ] ... C[ ]
3GR4A	CYS49(CB)	3.84	MET69(SD)	3GR4A		C[ ] ... S[ ]
3GR4A	CYS49(SG)	4.96	MET69(N)	3GR4A	[D-A-AA]:135.0° [A-D-DD]:51.0° d_planarity:-68.5°, maximum distance exceeded, bad d_angle(sp2), bad d_planarity	
3GR4A	CYS49(SG)	4.19	MET69(CA)	3GR4A		S[ ] ... C[ ]
3GR4A	CYS49(SG)	3.79	MET69(CB)	3GR4A		S[ ] ... C[ ]
3GR4A	CYS49(SG)	3.62	MET69(CG)	3GR4A		S[ ] ... C[ ]
3GR4A	CYS49(SG)	4.21	MET69(SD)	3GR4A		S[ ] ... S[ ]
3GR4A	CYS49(N)	4.07	VAL71(C)	3GR4A		N[ ] ... C[ ]
3GR4A	CYS49(N)	2.85	VAL71(O)	3GR4A	**H-bond** [D-A-AA]:170.4° [A-D-DD]:122.7° d_planarity:-5.3° a_planarity:85.4°	N[ ] ... O[ ]
3GR4A	CYS49(CA)	3.82	VAL71(O)	3GR4A		C[ ] ... O[ ]
3GR4A	CYS49(C)	4.09	VAL71(O)	3GR4A		C[ ] ... O[ ]
3GR4A	CYS49(O)	4.34	VAL71(C)	3GR4A		O[ ] ... C[ ]
3GR4A	CYS49(O)	3.51	VAL71(O)	3GR4A		O[ ] ... O[ ]
3GR4A	CYS49(CB)	4.11	VAL71(O)	3GR4A		C[ ] ... O[ ]
3GR4A	CYS49(SG)	4.76	VAL71(O)	3GR4A		
3GR4A	CYS49(N)	4.91	ALA72(N)	3GR4A		
3GR4A	CYS49(N)	4.83	ALA72(CA)	3GR4A		
3GR4A	CYS49(C)	4.61	ALA72(CA)	3GR4A		
3GR4A	CYS49(C)	4.75	ALA72(C)	3GR4A		
3GR4A	CYS49(O)	4.38	ALA72(N)	3GR4A	[D-A-AA]:140.6° [A-D-DD]:43.2° d_planarity:2.3° a_planarity:-23.5°, maximum distance exceeded, bad d_angle(sp2)	O[ ] ... N[ ]
3GR4A	CYS49(O)	3.46	ALA72(CA)	3GR4A		O[ ] ... C[ ]
3GR4A	CYS49(O)	3.62	ALA72(C)	3GR4A		O[ ] ... C[ ]
3GR4A	CYS49(O)	4.84	ALA72(O)	3GR4A		
3GR4A	CYS49(O)	4.46	ALA72(CB)	3GR4A		O[ ] ... C[ ]
3GR4A	CYS49(N)	4.70	ARG73(N)	3GR4A		
3GR4A	CYS49(CA)	4.89	ARG73(N)	3GR4A		
3GR4A	CYS49(C)	3.86	ARG73(N)	3GR4A		C[ ] ... N[ ]
3GR4A	CYS49(C)	4.58	ARG73(CA)	3GR4A		
3GR4A	CYS49(C)	4.52	ARG73(O)	3GR4A		
3GR4A	CYS49(C)	4.20	ARG73(CB)	3GR4A		C[ ] ... C[ ]
3GR4A	CYS49(O)	2.84	ARG73(N)	3GR4A	**H-bond** [D-A-AA]:142.0° [A-D-DD]:119.9° d_planarity:-1.5° a_planarity:70.1°	O[ ] ... N[ ]
3GR4A	CYS49(O)	3.77	ARG73(CA)	3GR4A		O[ ] ... C[ ]
3GR4A	CYS49(O)	4.27	ARG73(C)	3GR4A		O[ ] ... C[ ]
3GR4A	CYS49(O)	3.83	ARG73(O)	3GR4A		O[ ] ... O[ ]
3GR4A	CYS49(O)	3.77	ARG73(CB)	3GR4A		O[ ] ... C[ ]

3GR4A	CYS49(N)	4.93	LEU361(N)	3GR4A		
3GR4A	CYS49(N)	4.60	LEU361(O)	3GR4A	[D-A-AA]:132.2° [A-D-DD]:52.2° d_planarity:31.9° a_planarity:-18.4°, maximum distance exceeded, bad d_angle(sp2)	
3GR4A	CYS49(CA)	4.75	LEU361(N)	3GR4A		
3GR4A	CYS49(CA)	5.00	LEU361(CA)	3GR4A		
3GR4A	CYS49(CA)	4.80	LEU361(C)	3GR4A		
3GR4A	CYS49(CA)	3.89	LEU361(O)	3GR4A		C[ ] ... O[ ]
3GR4A	CYS49(CA)	4.57	LEU361(CB)	3GR4A		
3GR4A	CYS49(C)	4.96	LEU361(C)	3GR4A		
3GR4A	CYS49(C)	3.85	LEU361(O)	3GR4A		C[ ] ... O[ ]
3GR4A	CYS49(O)	4.96	LEU361(O)	3GR4A		
3GR4A	CYS49(SG)	5.00	LEU361(CB)	3GR4A		
3GR4A	CYS49(SG)	4.44	LEU361(CD1)	3GR4A		S[ ] ... C[ ]
3GR4A	CYS49(CA)	4.75	THR365(CB)	3GR4A		
3GR4A	CYS49(CA)	3.87	THR365(OG1)	3GR4A		C[ ] ... O[ ]
3GR4A	CYS49(CA)	4.91	THR365(CG2)	3GR4A		
3GR4A	CYS49(C)	4.86	THR365(CB)	3GR4A		
3GR4A	CYS49(C)	3.79	THR365(OG1)	3GR4A		C[ ] ... O[ ]
3GR4A	CYS49(O)	4.77	THR365(OG1)	3GR4A	[D-A-AA]:32.2° [A-D-DD]:125.2° a_planarity:67.5°, maximum distance exceeded, bad a_angle(sp2)	
3GR4A	CYS49(CB)	4.26	THR365(CB)	3GR4A		C[ ] ... C[ ]
3GR4A	CYS49(CB)	3.83	THR365(OG1)	3GR4A		C[ ] ... O[ ]
3GR4A	CYS49(CB)	4.28	THR365(CG2)	3GR4A		C[ ] ... C[ ]
3GR4A	CYS49(SG)	4.94	THR365(CB)	3GR4A		
3GR4A	CYS49(SG)	4.83	THR365(OG1)	3GR4A	[D-A-AA]:47.1° [A-D-DD]:85.8°, maximum distance exceeded, bad a_angle(sp3)	
3GR4A	CYS49(SG)	4.34	THR365(CG2)	3GR4A		S[ ] ... C[ ]
3GR4A	CYS49(CB)	4.96	VAL375(CG2)	3GR4A		
3GR4A	CYS49(SG)	4.68	VAL375(CB)	3GR4A		
3GR4A	CYS49(SG)	4.48	VAL375(CG1)	3GR4A		S[ ] ... C[ ]
3GR4A	CYS49(SG)	3.67	VAL375(CG2)	3GR4A		S[ ] ... C[ ]

chain1	res1/atm1	distance	res2/atm2	chain2	H-bonding	Charge interaction
3GR4A	CYS152(CB)	4.60	GLY122(C)	3GR4A		
3GR4A	CYS152(CB)	4.66	GLY122(O)	3GR4A		
3GR4A	CYS152(SG)	3.85	GLY122(N)	3GR4A	[D-A-AA]:133.0° [A-D-DD]:73.1° d_planarity:-14.1°, bad d_angle(sp2)	S[ ] ... N[ ]
3GR4A	CYS152(SG)	3.70	GLY122(CA)	3GR4A		S[ ] ... C[ ]
3GR4A	CYS152(SG)	3.71	GLY122(C)	3GR4A		S[ ] ... C[ ]
3GR4A	CYS152(SG)	4.10	GLY122(O)	3GR4A		S[ ] ... O[ ]
3GR4A	CYS152(N)	4.08	LEU123(C)	3GR4A		N[ ] ... C[ ]
3GR4A	CYS152(N)	2.94	LEU123(O)	3GR4A	**H-bond** [D-A-AA]:151.8° [A-D-DD]:105.2° d_planarity:-15.9° a_planarity:47.4°	N[ ] ... O[ ]
3GR4A	CYS152(CA)	4.49	LEU123(C)	3GR4A		C[ ] ... C[ ]
3GR4A	CYS152(CA)	3.61	LEU123(O)	3GR4A		C[ ] ... O[ ]
3GR4A	CYS152(C)	4.95	LEU123(C)	3GR4A		
3GR4A	CYS152(C)	4.22	LEU123(O)	3GR4A		C[ ] ... O[ ]

3GR4A	CYS152(O)	4.61	LEU123(C)	3GR4A		
3GR4A	CYS152(O)	3.92	LEU123(O)	3GR4A		O[ ] ... O[ ]
3GR4A	CYS152(CB)	4.71	LEU123(N)	3GR4A		
3GR4A	CYS152(CB)	4.92	LEU123(CA)	3GR4A		
3GR4A	CYS152(CB)	3.87	LEU123(C)	3GR4A		C[ ] ... C[ ]
3GR4A	CYS152(CB)	3.32	LEU123(O)	3GR4A		C[ ] ... O[ ]
3GR4A	CYS152(SG)	4.01	LEU123(N)	3GR4A	[D-A-AA]:101.0° [A-D-DD]:111.8° d_planarity:76.7°, maximum distance exceeded, bad d_planarity	S[ ] ... N[ ]
3GR4A	CYS152(SG)	4.76	LEU123(CA)	3GR4A		
3GR4A	CYS152(SG)	4.21	LEU123(C)	3GR4A		S[ ] ... C[ ]
3GR4A	CYS152(SG)	3.69	LEU123(O)	3GR4A		S[ ] ... O[ ]
3GR4A	CYS152(N)	4.70	ILE124(N)	3GR4A		
3GR4A	CYS152(N)	4.47	ILE124(CA)	3GR4A		N[ ] ... C[ ]
3GR4A	CYS152(N)	4.73	ILE124(C)	3GR4A		
3GR4A	CYS152(CA)	4.82	ILE124(N)	3GR4A		
3GR4A	CYS152(CA)	4.44	ILE124(CA)	3GR4A		C[ ] ... C[ ]
3GR4A	CYS152(CA)	4.42	ILE124(CD1)	3GR4A		C[ ] ... C[ ]
3GR4A	CYS152(C)	4.92	ILE124(N)	3GR4A		
3GR4A	CYS152(C)	4.10	ILE124(CA)	3GR4A		C[ ] ... C[ ]
3GR4A	CYS152(C)	4.57	ILE124(C)	3GR4A		
3GR4A	CYS152(C)	4.85	ILE124(CB)	3GR4A		
3GR4A	CYS152(C)	4.87	ILE124(CG1)	3GR4A		
3GR4A	CYS152(C)	4.87	ILE124(CG2)	3GR4A		
3GR4A	CYS152(C)	3.84	ILE124(CD1)	3GR4A		C[ ] ... C[ ]
3GR4A	CYS152(O)	4.45	ILE124(N)	3GR4A	[D-A-AA]:105.1° [A-D-DD]:38.1° d_planarity:28.2° a_planarity:-51.8°, maximum distance exceeded, bad d_angle(sp2)	O[ ] ... N[ ]
3GR4A	CYS152(O)	3.42	ILE124(CA)	3GR4A		O[ ] ... C[ ]
3GR4A	CYS152(O)	3.57	ILE124(C)	3GR4A		O[ ] ... C[ ]
3GR4A	CYS152(O)	4.77	ILE124(O)	3GR4A		
3GR4A	CYS152(O)	4.22	ILE124(CB)	3GR4A		O[ ] ... C[ ]
3GR4A	CYS152(O)	4.62	ILE124(CG1)	3GR4A		
3GR4A	CYS152(O)	4.08	ILE124(CG2)	3GR4A		O[ ] ... C[ ]
3GR4A	CYS152(O)	3.88	ILE124(CD1)	3GR4A		O[ ] ... C[ ]
3GR4A	CYS152(CB)	4.13	ILE124(N)	3GR4A		C[ ] ... N[ ]
3GR4A	CYS152(CB)	4.05	ILE124(CA)	3GR4A		C[ ] ... C[ ]
3GR4A	CYS152(CB)	4.92	ILE124(CB)	3GR4A		
3GR4A	CYS152(CB)	4.52	ILE124(CG1)	3GR4A		
3GR4A	CYS152(CB)	3.67	ILE124(CD1)	3GR4A		C[ ] ... C[ ]
3GR4A	CYS152(SG)	4.85	ILE124(N)	3GR4A	[D-A-AA]:56.1° [A-D-DD]:98.1° d_planarity:51.8°, maximum distance exceeded, bad a_angle(sp3)	
3GR4A	CYS152(N)	3.99	LYS125(N)	3GR4A		N[ ] ... N[ ]
3GR4A	CYS152(N)	4.81	LYS125(CA)	3GR4A		
3GR4A	CYS152(CA)	4.48	LYS125(N)	3GR4A		C[ ] ... N[ ]
3GR4A	CYS152(C)	3.92	LYS125(N)	3GR4A		C[ ] ... N[ ]
3GR4A	CYS152(C)	4.90	LYS125(CA)	3GR4A		
3GR4A	CYS152(C)	4.87	LYS125(CG)	3GR4A		
3GR4A	CYS152(O)	2.81	LYS125(N)	3GR4A	**H-bond** [D-A-AA]:150.3° [A-D-DD]:118.7° d_planarity:-22.5° a_planarity:-24.6°	O[ ] ... N[ ]
3GR4A	CYS152(O)	3.73	LYS125(CA)	3GR4A		O[ ] ... C[ ]

3GR4A	CYS152(O)	4.28	LYS125(CB)	3GR4A		O[ ] ... C[ ]
3GR4A	CYS152(O)	3.71	LYS125(CG)	3GR4A		O[ ] ... C[ ]
3GR4A	CYS152(CB)	4.88	LYS125(N)	3GR4A		
3GR4A	CYS152(N)	4.80	TYR148(C)	3GR4A		
3GR4A	CYS152(N)	3.71	TYR148(O)	3GR4A	[D-A-AA]:149.0° [A-D-DD]:111.3° d_planarity:-89.8° a_planarity:-57.9°, maximum distance exceeded, bad d_planarity	N[ ] ... O[ ]
3GR4A	CYS152(CA)	4.45	TYR148(O)	3GR4A		C[ ] ... O[ ]
3GR4A	CYS152(CB)	4.79	TYR148(O)	3GR4A		
3GR4A	CYS152(SG)	4.97	TYR148(C)	3GR4A		
3GR4A	CYS152(SG)	3.80	TYR148(O)	3GR4A		S[ ] ... O[ ]
3GR4A	CYS152(SG)	4.81	MET149(C)	3GR4A		
3GR4A	CYS152(SG)	4.54	MET149(O)	3GR4A		
3GR4A	CYS152(N)	4.89	GLU150(N)	3GR4A		
3GR4A	CYS152(N)	4.65	GLU150(CA)	3GR4A		
3GR4A	CYS152(N)	3.67	GLU150(C)	3GR4A		N[ ] ... C[ ]
3GR4A	CYS152(N)	4.15	GLU150(O)	3GR4A	[D-A-AA]:59.0° [A-D-DD]:161.2° d_planarity:-40.5° a_planarity:39.7°, maximum distance exceeded, bad a_angle(sp2)	N[ ] ... O[ ]
3GR4A	CYS152(N)	2.77	LYS151(N)	3GR4A		N[ ] ... N[ ]
3GR4A	CYS152(N)	2.44	LYS151(CA)	3GR4A		N[ ] ... C[ ]
3GR4A	CYS152(N)	2.26	LYS151(O)	3GR4A	[D-A-AA]:29.8° [A-D-DD]:94.3° d_planarity:1.3° a_planarity:0.1°, bad a_angle(sp2)	N[ ] ... O[ ]
3GR4A	CYS152(N)	3.74	LYS151(CB)	3GR4A		N[ ] ... C[ ]
3GR4A	CYS152(CA)	4.20	LYS151(N)	3GR4A		C[ ] ... N[ ]
3GR4A	CYS152(CA)	3.82	LYS151(CA)	3GR4A		C[ ] ... C[ ]
3GR4A	CYS152(CA)	2.45	LYS151(C)	3GR4A		C[ ] ... C[ ]
3GR4A	CYS152(CA)	2.78	LYS151(O)	3GR4A		C[ ] ... O[ ]
3GR4A	CYS152(CA)	4.97	LYS151(CB)	3GR4A		
3GR4A	CYS152(C)	4.42	LYS151(CA)	3GR4A		C[ ] ... C[ ]
3GR4A	CYS152(C)	3.08	LYS151(C)	3GR4A		C[ ] ... C[ ]
3GR4A	CYS152(C)	3.15	LYS151(O)	3GR4A		C[ ] ... O[ ]
3GR4A	CYS152(O)	4.27	LYS151(CA)	3GR4A		O[ ] ... C[ ]
3GR4A	CYS152(O)	3.24	LYS151(C)	3GR4A		O[ ] ... C[ ]
3GR4A	CYS152(O)	3.50	LYS151(O)	3GR4A		O[ ] ... O[ ]
3GR4A	CYS152(CB)	4.89	LYS151(N)	3GR4A		
3GR4A	CYS152(CB)	4.90	LYS151(CA)	3GR4A		
3GR4A	CYS152(CB)	3.74	LYS151(C)	3GR4A		C[ ] ... C[ ]
3GR4A	CYS152(CB)	4.26	LYS151(O)	3GR4A		C[ ] ... O[ ]
3GR4A	CYS152(SG)	4.78	LYS151(N)	3GR4A	[D-A-AA]:82.5° [A-D-DD]:100.4° d_planarity:-55.0°, maximum distance exceeded	
3GR4A	CYS152(SG)	4.34	LYS151(C)	3GR4A		S[ ] ... C[ ]
3GR4A	CYS152(SG)	4.98	LYS151(O)	3GR4A	Weakly positive	
3GR4A	CYS152(N)	3.58	ASP153(N)	3GR4A		N[ ] ... N[ ]
3GR4A	CYS152(N)	4.81	ASP153(CA)	3GR4A		
3GR4A	CYS152(CA)	2.42	ASP153(N)	3GR4A		C[ ] ... N[ ]
3GR4A	CYS152(CA)	3.80	ASP153(CA)	3GR4A		C[ ] ... C[ ]
3GR4A	CYS152(CA)	4.85	ASP153(C)	3GR4A		
3GR4A	CYS152(CA)	4.82	ASP153(O)	3GR4A		
3GR4A	CYS152(CA)	4.45	ASP153(CB)	3GR4A		C[ ] ... C[ ]
3GR4A	CYS152(CA)	4.82	ASP153(CG)	3GR4A		

3GR4A	CYS152(CA)	4.99	ASP153(OD1)	3GR4A		
3GR4A	CYS152(C)	2.43	ASP153(CA)	3GR4A		C[ ] ... C[ ]
3GR4A	CYS152(C)	3.71	ASP153(C)	3GR4A		C[ ] ... C[ ]
3GR4A	CYS152(C)	3.99	ASP153(O)	3GR4A		C[ ] ... O[ ]
3GR4A	CYS152(C)	3.08	ASP153(CB)	3GR4A		C[ ] ... C[ ]
3GR4A	CYS152(C)	3.76	ASP153(CG)	3GR4A		C[ ] ... C[ ]
3GR4A	CYS152(C)	4.13	ASP153(OD1)	3GR4A		C[ ] ... O[-]
3GR4A	CYS152(C)	4.49	ASP153(OD2)	3GR4A		C[ ] ... O[-]
3GR4A	CYS152(O)	2.25	ASP153(N)	3GR4A	[D-A-AA]:29.5° [A-D-DD]:94.3° d_planarity:1.0° a_planarity:1.8°, bad a_angle(sp2)	O[ ] ... N[ ]
3GR4A	CYS152(O)	2.77	ASP153(CA)	3GR4A		O[ ] ... C[ ]
3GR4A	CYS152(O)	4.25	ASP153(C)	3GR4A		O[ ] ... C[ ]
3GR4A	CYS152(O)	4.78	ASP153(O)	3GR4A		
3GR4A	CYS152(O)	3.06	ASP153(CB)	3GR4A		O[ ] ... C[ ]
3GR4A	CYS152(O)	4.04	ASP153(CG)	3GR4A		O[ ] ... C[ ]
3GR4A	CYS152(O)	4.71	ASP153(OD1)	3GR4A		
3GR4A	CYS152(O)	4.58	ASP153(OD2)	3GR4A		
3GR4A	CYS152(CB)	3.19	ASP153(N)	3GR4A		C[ ] ... N[ ]
3GR4A	CYS152(CB)	4.50	ASP153(CA)	3GR4A		C[ ] ... C[ ]
3GR4A	CYS152(SG)	4.83	ASP153(N)	3GR4A	[D-A-AA]:20.7° [A-D-DD]:158.7° d_planarity:67.2°, maximum distance exceeded, bad a_angle(sp3), bad d_planarity	
3GR4A	CYS152(C)	4.84	GLU154(N)	3GR4A		
3GR4A	CYS152(N)	4.57	ILE156(O)	3GR4A	[D-A-AA]:142.4° [A-D-DD]:29.1° d_planarity:38.1° a_planarity:76.7°, maximum distance exceeded, bad d_angle(sp2)	
3GR4A	CYS152(CA)	4.50	ILE156(C)	3GR4A		C[ ] ... C[ ]
3GR4A	CYS152(CA)	3.37	ILE156(O)	3GR4A		C[ ] ... O[ ]
3GR4A	CYS152(CA)	4.78	ILE156(CG2)	3GR4A		
3GR4A	CYS152(C)	4.89	ILE156(C)	3GR4A		
3GR4A	CYS152(C)	3.67	ILE156(O)	3GR4A		C[ ] ... O[ ]
3GR4A	CYS152(O)	4.88	ILE156(O)	3GR4A		
3GR4A	CYS152(CB)	4.86	ILE156(C)	3GR4A		
3GR4A	CYS152(CB)	3.83	ILE156(O)	3GR4A		C[ ] ... O[ ]
3GR4A	CYS152(SG)	4.51	ILE156(O)	3GR4A		
3GR4A	CYS152(CA)	4.87	LEU157(CA)	3GR4A		
3GR4A	CYS152(CB)	4.49	LEU157(CA)	3GR4A		C[ ] ... C[ ]
3GR4A	CYS152(CB)	4.02	LEU157(CD1)	3GR4A		C[ ] ... C[ ]
3GR4A	CYS152(SG)	4.20	LEU157(CA)	3GR4A		S[ ] ... C[ ]
3GR4A	CYS152(SG)	4.31	LEU157(C)	3GR4A		S[ ] ... C[ ]
3GR4A	CYS152(SG)	4.90	LEU157(CG)	3GR4A		
3GR4A	CYS152(SG)	3.80	LEU157(CD1)	3GR4A		S[ ] ... C[ ]
3GR4A	CYS152(CB)	4.68	TRP158(N)	3GR4A		
3GR4A	CYS152(SG)	3.46	TRP158(N)	3GR4A	**H-bond** [D-A-AA]:121.5° [A-D-DD]:112.6° d_planarity:-19.9°	S[ ] ... N[ ]
3GR4A	CYS152(SG)	4.24	TRP158(CA)	3GR4A		S[ ] ... C[ ]
3GR4A	CYS152(SG)	4.57	TRP158(C)	3GR4A		
3GR4A	CYS152(SG)	3.97	TRP158(O)	3GR4A		S[ ] ... O[ ]
3GR4A	CYS152(SG)	4.08	TRP158(CB)	3GR4A		S[ ] ... C[ ]

chain1	res1/atm1	distance	res2/atm2	chain2	H-bonding	Charge interaction
3GR4A	CYS326(N)	4.99	VAL292(N)	3GR4A		
3GR4A	CYS326(CA)	4.99	VAL292(N)	3GR4A		
3GR4A	CYS326(CA)	4.42	VAL292(O)	3GR4A		C[ ] ... O[ ]
3GR4A	CYS326(CA)	4.60	VAL292(CB)	3GR4A		
3GR4A	CYS326(C)	4.27	VAL292(O)	3GR4A		C[ ] ... O[ ]
3GR4A	CYS326(SG)	4.97	ARG294(CG)	3GR4A		
3GR4A	CYS326(SG)	4.12	ARG294(CD)	3GR4A		S[ ] ... C[ ]
3GR4A	CYS326(SG)	3.95	ARG294(NE)	3GR4A	[D-A-AA]:145.2° [A-D-DD]:73.8° d_planarity:73.9°, bad d_angle(sp2), bad d_planarity	S[ ] ... N[ ]
3GR4A	CYS326(SG)	3.80	ARG294(CZ)	3GR4A	Positive	S[ ] ... C[ ]
3GR4A	CYS326(SG)	3.71	ARG294(NH1)	3GR4A	[D-A-AA]:176.1° [A-D-DD]:83.3° d_planarity:-77.5°, bad d_angle(sp2), bad d_planarity	S[ ] ... N[+]
3GR4A	CYS326(SG)	4.39	ARG294(NH2)	3GR4A	[D-A-AA]:144.9° [A-D-DD]:55.5° d_planarity:84.0°, maximum distance exceeded, bad d_angle(sp2), bad d_planarity	S[ ] ... N[+]
3GR4A	CYS326(CB)	4.95	GLN310(CD)	3GR4A		
3GR4A	CYS326(CB)	4.82	GLN310(NE2)	3GR4A		
3GR4A	CYS326(SG)	4.70	GLN310(CG)	3GR4A		
3GR4A	CYS326(SG)	3.84	GLN310(CD)	3GR4A		S[ ] ... C[ ]
3GR4A	CYS326(SG)	3.72	GLN310(OE1)	3GR4A		S[ ] ... O[ ]
3GR4A	CYS326(SG)	3.84	GLN310(NE2)	3GR4A	[D-A-AA]:113.4° [A-D-DD]:79.9° d_planarity:65.4°, bad d_angle(sp2), bad d_planarity	S[ ] ... N[ ]
3GR4A	CYS326(CB)	4.99	ILE314(CD1)	3GR4A		
3GR4A	CYS326(N)	4.27	VAL324(C)	3GR4A		N[ ] ... C[ ]
3GR4A	CYS326(N)	4.30	VAL324(O)	3GR4A	[D-A-AA]:80.0° [A-D-DD]:155.1° d_planarity:-86.8° a_planarity:25.9°, maximum distance exceeded, bad a_angle(sp2), bad d_planarity	N[ ] ... O[ ]
3GR4A	CYS326(N)	3.52	ILE325(N)	3GR4A		N[ ] ... N[ ]
3GR4A	CYS326(N)	2.45	ILE325(CA)	3GR4A		N[ ] ... C[ ]
3GR4A	CYS326(N)	2.24	ILE325(O)	3GR4A	[D-A-AA]:30.0° [A-D-DD]:95.2° d_planarity:5.1° a_planarity:1.1°, bad a_angle(sp2)	N[ ] ... O[ ]
3GR4A	CYS326(N)	3.42	ILE325(CB)	3GR4A		N[ ] ... C[ ]
3GR4A	CYS326(N)	4.72	ILE325(CG1)	3GR4A		
3GR4A	CYS326(N)	3.38	ILE325(CG2)	3GR4A		N[ ] ... C[ ]
3GR4A	CYS326(CA)	4.82	ILE325(N)	3GR4A		
3GR4A	CYS326(CA)	3.83	ILE325(CA)	3GR4A		C[ ] ... C[ ]
3GR4A	CYS326(CA)	2.45	ILE325(C)	3GR4A		C[ ] ... C[ ]
3GR4A	CYS326(CA)	2.79	ILE325(O)	3GR4A		C[ ] ... O[ ]
3GR4A	CYS326(CA)	4.60	ILE325(CB)	3GR4A		
3GR4A	CYS326(CA)	4.31	ILE325(CG2)	3GR4A		C[ ] ... C[ ]
3GR4A	CYS326(C)	4.45	ILE325(CA)	3GR4A		C[ ] ... C[ ]
3GR4A	CYS326(C)	3.23	ILE325(C)	3GR4A		C[ ] ... C[ ]
3GR4A	CYS326(C)	3.54	ILE325(O)	3GR4A		C[ ] ... O[ ]
3GR4A	CYS326(C)	4.74	ILE325(CB)	3GR4A		
3GR4A	CYS326(C)	3.98	ILE325(CG2)	3GR4A		C[ ] ... C[ ]
3GR4A	CYS326(O)	4.63	ILE325(CA)	3GR4A		
3GR4A	CYS326(O)	3.74	ILE325(C)	3GR4A		O[ ] ... C[ ]
3GR4A	CYS326(O)	4.32	ILE325(O)	3GR4A		O[ ] ... O[ ]
3GR4A	CYS326(O)	4.79	ILE325(CB)	3GR4A		
3GR4A	CYS326(O)	3.75	ILE325(CG2)	3GR4A		O[ ] ... C[ ]

3GR4A	CYS326(CB)	4.90	ILE325(CA)	3GR4A		
3GR4A	CYS326(CB)	3.68	ILE325(C)	3GR4A		C[ ] ... C[ ]
3GR4A	CYS326(CB)	4.11	ILE325(O)	3GR4A		C[ ] ... O[ ]
3GR4A	CYS326(N)	3.45	ALA327(N)	3GR4A		N[ ] ... N[ ]
3GR4A	CYS326(N)	4.63	ALA327(CA)	3GR4A		
3GR4A	CYS326(CA)	2.42	ALA327(N)	3GR4A		C[ ] ... N[ ]
3GR4A	CYS326(CA)	3.78	ALA327(CA)	3GR4A		C[ ] ... C[ ]
3GR4A	CYS326(CA)	4.81	ALA327(C)	3GR4A		
3GR4A	CYS326(CA)	4.95	ALA327(O)	3GR4A		
3GR4A	CYS326(CA)	4.54	ALA327(CB)	3GR4A		
3GR4A	CYS326(C)	2.43	ALA327(CA)	3GR4A		C[ ] ... C[ ]
3GR4A	CYS326(C)	3.58	ALA327(C)	3GR4A		C[ ] ... C[ ]
3GR4A	CYS326(C)	4.02	ALA327(O)	3GR4A		C[ ] ... O[ ]
3GR4A	CYS326(C)	3.36	ALA327(CB)	3GR4A		C[ ] ... C[ ]
3GR4A	CYS326(O)	2.25	ALA327(N)	3GR4A	[D-A-AA]:29.6° [A-D-DD]:95.7° d_planarity:3.1° a_planarity:0.1°, bad a_angle(sp2)	O[ ] ... N[ ]
3GR4A	CYS326(O)	2.79	ALA327(CA)	3GR4A		O[ ] ... C[ ]
3GR4A	CYS326(O)	3.98	ALA327(C)	3GR4A		O[ ] ... C[ ]
3GR4A	CYS326(O)	4.68	ALA327(O)	3GR4A		
3GR4A	CYS326(O)	3.75	ALA327(CB)	3GR4A		O[ ] ... C[ ]
3GR4A	CYS326(CB)	3.42	ALA327(N)	3GR4A		C[ ] ... N[ ]
3GR4A	CYS326(CB)	4.71	ALA327(CA)	3GR4A		
3GR4A	CYS326(SG)	3.59	ALA327(N)	3GR4A	[D-A-AA]:70.3° [A-D-DD]:136.8° d_planarity:84.8°, bad d_planarity	S[ ] ... N[ ]
3GR4A	CYS326(SG)	4.73	ALA327(CA)	3GR4A		
3GR4A	CYS326(SG)	4.97	ALA327(C)	3GR4A		
3GR4A	CYS326(SG)	4.70	ALA327(O)	3GR4A		
3GR4A	CYS326(C)	4.53	THR328(N)	3GR4A		
3GR4A	CYS326(O)	4.62	THR328(N)	3GR4A	[D-A-AA]:78.4° [A-D-DD]:171.0° d_planarity:83.9° a_planarity:15.8°, maximum distance exceeded, bad a_angle(sp2), bad d_planarity	
3GR4A	CYS326(CA)	4.93	MET330(SD)	3GR4A		
3GR4A	CYS326(C)	4.14	MET330(SD)	3GR4A		C[ ] ... S[ ]
3GR4A	CYS326(O)	4.65	MET330(CG)	3GR4A		
3GR4A	CYS326(O)	3.54	MET330(SD)	3GR4A		O[ ] ... S[ ]
3GR4A	CYS326(O)	4.73	MET330(CE)	3GR4A		
3GR4A	CYS326(CB)	4.37	MET330(SD)	3GR4A		C[ ] ... S[ ]
3GR4A	CYS326(CB)	4.37	MET330(CE)	3GR4A		C[ ] ... C[ ]
3GR4A	CYS326(SG)	4.85	MET330(CG)	3GR4A		
3GR4A	CYS326(SG)	4.21	MET330(SD)	3GR4A		S[ ] ... S[ ]
3GR4A	CYS326(SG)	3.93	MET330(CE)	3GR4A		S[ ] ... C[ ]
3GR4A	CYS326(CB)	3.99	ALA351(CB)	3GR4A		C[ ] ... C[ ]
3GR4A	CYS326(SG)	3.94	ALA351(CB)	3GR4A		S[ ] ... C[ ]
3GR4A	CYS326(N)	4.39	ALA356(CB)	3GR4A		N[ ] ... C[ ]
3GR4A	CYS326(CA)	4.97	ALA356(CB)	3GR4A		
3GR4A	CYS326(CB)	4.29	ALA356(CB)	3GR4A		C[ ] ... C[ ]
3GR4A	CYS326(N)	4.22	CYS358(C)	3GR4A		N[ ] ... C[ ]
3GR4A	CYS326(N)	3.08	CYS358(O)	3GR4A	**H-bond** [D-A-AA]:155.2° [A-D-DD]:126.6° d_planarity:-10.0° a_planarity:65.0°	N[ ] ... O[ ]
3GR4A	CYS326(CA)	4.13	CYS358(O)	3GR4A		C[ ] ... O[ ]

3GR4A	CYS326(C)	4.55	CYS358(O)	3GR4A		
3GR4A	CYS326(O)	4.72	CYS358(C)	3GR4A		
3GR4A	CYS326(O)	4.04	CYS358(O)	3GR4A		O[ ] ... O[ ]
3GR4A	CYS326(CB)	4.26	CYS358(O)	3GR4A		C[ ] ... O[ ]
3GR4A	CYS326(N)	4.90	ILE359(N)	3GR4A		
3GR4A	CYS326(N)	4.63	ILE359(CA)	3GR4A		
3GR4A	CYS326(C)	4.68	ILE359(CA)	3GR4A		
3GR4A	CYS326(C)	4.98	ILE359(C)	3GR4A		
3GR4A	CYS326(O)	4.62	ILE359(N)	3GR4A	[D-A-AA]:136.1° [A-D-DD]:39.2° d_planarity:28.5° a_planarity:-16.1°, maximum distance exceeded, bad d_angle(sp2)	
3GR4A	CYS326(O)	3.61	ILE359(CA)	3GR4A		O[ ] ... C[ ]
3GR4A	CYS326(O)	3.79	ILE359(C)	3GR4A		O[ ] ... C[ ]
3GR4A	CYS326(O)	4.87	ILE359(O)	3GR4A		
3GR4A	CYS326(O)	4.45	ILE359(CB)	3GR4A		O[ ] ... C[ ]
3GR4A	CYS326(O)	4.75	ILE359(CG2)	3GR4A		
3GR4A	CYS326(CB)	4.99	ILE359(CA)	3GR4A		
3GR4A	CYS326(CB)	4.63	ILE359(CG2)	3GR4A		
3GR4A	CYS326(C)	4.43	MET360(N)	3GR4A		C[ ] ... N[ ]
3GR4A	CYS326(C)	4.59	MET360(CB)	3GR4A		
3GR4A	CYS326(O)	3.23	MET360(N)	3GR4A	**H-bond** [D-A-AA]:171.8° [A-D-DD]:113.4° d_planarity:-55.2° a_planarity:11.4°	O[ ] ... N[ ]
3GR4A	CYS326(O)	4.04	MET360(CA)	3GR4A		O[ ] ... C[ ]
3GR4A	CYS326(O)	4.85	MET360(C)	3GR4A		
3GR4A	CYS326(O)	4.68	MET360(O)	3GR4A		
3GR4A	CYS326(O)	3.69	MET360(CB)	3GR4A		O[ ] ... C[ ]

chain1	res1/atm1	distance	res2/atm2	chain2	H-bonding	Charge interaction
3GR4A	CYS358(N)	4.08	THR45(CG2)	3GR4A		N[ ] ... C[ ]
3GR4A	CYS358(CA)	4.85	THR45(C)	3GR4A		
3GR4A	CYS358(CA)	3.80	THR45(CG2)	3GR4A		C[ ] ... C[ ]
3GR4A	CYS358(C)	3.98	THR45(CG2)	3GR4A		C[ ] ... C[ ]
3GR4A	CYS358(O)	4.74	THR45(CG2)	3GR4A		
3GR4A	CYS358(N)	4.69	GLY46(N)	3GR4A		
3GR4A	CYS358(N)	4.48	GLY46(O)	3GR4A	[D-A-AA]:125.2° [A-D-DD]:24.1° d_planarity:7.0° a_planarity:-26.3°, maximum distance exceeded, bad d_angle(sp2)	N[ ] ... O[ ]
3GR4A	CYS358(CA)	4.14	GLY46(N)	3GR4A		C[ ] ... N[ ]
3GR4A	CYS358(CA)	4.43	GLY46(CA)	3GR4A		C[ ] ... C[ ]
3GR4A	CYS358(CA)	4.11	GLY46(C)	3GR4A		C[ ] ... C[ ]
3GR4A	CYS358(CA)	3.19	GLY46(O)	3GR4A		C[ ] ... O[ ]
3GR4A	CYS358(C)	4.68	GLY46(C)	3GR4A		
3GR4A	CYS358(C)	3.53	GLY46(O)	3GR4A		C[ ] ... O[ ]
3GR4A	CYS358(O)	4.74	GLY46(O)	3GR4A		
3GR4A	CYS358(CB)	4.63	GLY46(N)	3GR4A		
3GR4A	CYS358(CB)	4.47	GLY46(CA)	3GR4A		C[ ] ... C[ ]
3GR4A	CYS358(CB)	4.10	GLY46(C)	3GR4A		C[ ] ... C[ ]
3GR4A	CYS358(CB)	3.41	GLY46(O)	3GR4A		C[ ] ... O[ ]

3GR4A	CYS358(SG)	4.32	GLY46(N)	3GR4A	[D-A-AA]:88.0° [A-D-DD]:68.3° d_planarity:-27.0°, maximum distance exceeded, bad d_angle(sp2)	S[ ] ... N[ ]
3GR4A	CYS358(SG)	4.02	GLY46(CA)	3GR4A		S[ ] ... C[ ]
3GR4A	CYS358(SG)	4.19	GLY46(C)	3GR4A		S[ ] ... C[ ]
3GR4A	CYS358(SG)	4.00	GLY46(O)	3GR4A		S[ ] ... O[ ]
3GR4A	CYS358(CB)	4.22	ILE48(CG1)	3GR4A		C[ ] ... C[ ]
3GR4A	CYS358(CB)	3.81	ILE48(CD1)	3GR4A		C[ ] ... C[ ]
3GR4A	CYS358(SG)	4.47	ILE48(CG1)	3GR4A		S[ ] ... C[ ]
3GR4A	CYS358(SG)	4.15	ILE48(CD1)	3GR4A		S[ ] ... C[ ]
3GR4A	CYS358(N)	4.33	VAL324(C)	3GR4A		N[ ] ... C[ ]
3GR4A	CYS358(N)	3.20	VAL324(O)	3GR4A	**H-bond** [D-A-AA]:150.5° [A-D-DD]:124.8° d_planarity:-13.6° a_planarity:44.4°	N[ ] ... O[ ]
3GR4A	CYS358(CA)	4.22	VAL324(O)	3GR4A		C[ ] ... O[ ]
3GR4A	CYS358(C)	4.48	VAL324(O)	3GR4A		C[ ] ... O[ ]
3GR4A	CYS358(O)	4.58	VAL324(C)	3GR4A		
3GR4A	CYS358(O)	3.84	VAL324(O)	3GR4A		O[ ] ... O[ ]
3GR4A	CYS358(CB)	4.49	VAL324(O)	3GR4A		C[ ] ... O[ ]
3GR4A	CYS358(N)	4.90	ILE325(N)	3GR4A		
3GR4A	CYS358(N)	4.56	ILE325(CA)	3GR4A		
3GR4A	CYS358(CA)	4.71	ILE325(CA)	3GR4A		
3GR4A	CYS358(C)	4.31	ILE325(CA)	3GR4A		C[ ] ... C[ ]
3GR4A	CYS358(C)	4.83	ILE325(C)	3GR4A		
3GR4A	CYS358(C)	4.88	ILE325(CB)	3GR4A		
3GR4A	CYS358(C)	4.50	ILE325(CG2)	3GR4A		
3GR4A	CYS358(O)	4.48	ILE325(N)	3GR4A	[D-A-AA]:127.3° [A-D-DD]:38.8° d_planarity:8.3° a_planarity:-37.3°, maximum distance exceeded, bad d_angle(sp2)	O[ ] ... N[ ]
3GR4A	CYS358(O)	3.46	ILE325(CA)	3GR4A		O[ ] ... C[ ]
3GR4A	CYS358(O)	3.77	ILE325(C)	3GR4A		O[ ] ... C[ ]
3GR4A	CYS358(O)	4.98	ILE325(O)	3GR4A		
3GR4A	CYS358(O)	4.29	ILE325(CB)	3GR4A		O[ ] ... C[ ]
3GR4A	CYS358(O)	4.83	ILE325(CG1)	3GR4A		
3GR4A	CYS358(O)	4.06	ILE325(CG2)	3GR4A		O[ ] ... C[ ]
3GR4A	CYS358(CB)	4.47	ILE325(CA)	3GR4A		C[ ] ... C[ ]
3GR4A	CYS358(CB)	4.51	ILE325(CB)	3GR4A		
3GR4A	CYS358(CB)	4.04	ILE325(CG1)	3GR4A		C[ ] ... C[ ]
3GR4A	CYS358(CB)	4.40	ILE325(CG2)	3GR4A		C[ ] ... C[ ]
3GR4A	CYS358(SG)	4.66	ILE325(CG1)	3GR4A		
3GR4A	CYS358(C)	4.22	CYS326(N)	3GR4A		C[ ] ... N[ ]
3GR4A	CYS358(C)	4.72	CYS326(O)	3GR4A		
3GR4A	CYS358(O)	3.08	CYS326(N)	3GR4A	**H-bond** [D-A-AA]:155.2° [A-D-DD]:126.6° d_planarity:-10.0° a_planarity:65.0°	O[ ] ... N[ ]
3GR4A	CYS358(O)	4.13	CYS326(CA)	3GR4A		O[ ] ... C[ ]
3GR4A	CYS358(O)	4.55	CYS326(C)	3GR4A		
3GR4A	CYS358(O)	4.04	CYS326(O)	3GR4A		O[ ] ... O[ ]
3GR4A	CYS358(O)	4.26	CYS326(CB)	3GR4A		O[ ] ... C[ ]
3GR4A	CYS358(N)	4.37	ALA356(CA)	3GR4A		N[ ] ... C[ ]
3GR4A	CYS358(N)	3.34	ALA356(C)	3GR4A		N[ ] ... C[ ]
3GR4A	CYS358(N)	3.60	ALA356(O)	3GR4A	[D-A-AA]:68.0° [A-D-DD]:127.0° d_planarity:70.8° a_planarity:47.9°, maximum distance exceeded, bad a_angle(sp2), bad d_planarity	N[ ] ... O[ ]

3GR4A	CYS358(N)	4.35	ALA356(CB)	3GR4A		N[ ] ... C[ ]
3GR4A	CYS358(CA)	4.57	ALA356(C)	3GR4A		
3GR4A	CYS358(CA)	4.65	ALA356(O)	3GR4A		
3GR4A	CYS358(C)	4.82	ALA356(C)	3GR4A		
3GR4A	CYS358(C)	4.94	ALA356(O)	3GR4A		
3GR4A	CYS358(C)	4.59	ALA356(CB)	3GR4A		
3GR4A	CYS358(O)	4.71	ALA356(CA)	3GR4A		
3GR4A	CYS358(O)	4.49	ALA356(C)	3GR4A		O[ ] ... C[ ]
3GR4A	CYS358(O)	4.86	ALA356(O)	3GR4A		
3GR4A	CYS358(O)	3.77	ALA356(CB)	3GR4A		O[ ] ... C[ ]
3GR4A	CYS358(N)	2.79	ASP357(N)	3GR4A		N[ ] ... N[ ]
3GR4A	CYS358(N)	2.41	ASP357(CA)	3GR4A		N[ ] ... C[ ]
3GR4A	CYS358(N)	2.27	ASP357(O)	3GR4A	[D-A-AA]:28.5° [A-D-DD]:96.4° d_planarity:1.2° a_planarity:2.7°, bad a_angle(sp2)	N[ ] ... O[ ]
3GR4A	CYS358(N)	3.10	ASP357(CB)	3GR4A		N[ ] ... C[ ]
3GR4A	CYS358(N)	4.50	ASP357(CG)	3GR4A		
3GR4A	CYS358(CA)	4.21	ASP357(N)	3GR4A		C[ ] ... N[ ]
3GR4A	CYS358(CA)	3.82	ASP357(CA)	3GR4A		C[ ] ... C[ ]
3GR4A	CYS358(CA)	2.46	ASP357(C)	3GR4A		C[ ] ... C[ ]
3GR4A	CYS358(CA)	2.84	ASP357(O)	3GR4A		C[ ] ... O[ ]
3GR4A	CYS358(CA)	4.44	ASP357(CB)	3GR4A		C[ ] ... C[ ]
3GR4A	CYS358(C)	4.76	ASP357(N)	3GR4A		
3GR4A	CYS358(C)	4.77	ASP357(CA)	3GR4A		
3GR4A	CYS358(C)	3.59	ASP357(C)	3GR4A		C[ ] ... C[ ]
3GR4A	CYS358(C)	4.08	ASP357(O)	3GR4A		C[ ] ... O[ ]
3GR4A	CYS358(O)	4.49	ASP357(N)	3GR4A	[D-A-AA]:95.3° [A-D-DD]:95.7° d_planarity:83.8° a_planarity:-31.8°, maximum distance exceeded, bad d_planarity	O[ ] ... N[ ]
3GR4A	CYS358(O)	4.85	ASP357(CA)	3GR4A		
3GR4A	CYS358(O)	3.99	ASP357(C)	3GR4A		O[ ] ... C[ ]
3GR4A	CYS358(O)	4.75	ASP357(O)	3GR4A		
3GR4A	CYS358(CB)	4.68	ASP357(CA)	3GR4A		
3GR4A	CYS358(CB)	3.44	ASP357(C)	3GR4A		C[ ] ... C[ ]
3GR4A	CYS358(CB)	3.76	ASP357(O)	3GR4A		C[ ] ... O[ ]
3GR4A	CYS358(CB)	4.85	ASP357(CB)	3GR4A		
3GR4A	CYS358(SG)	4.77	ASP357(CA)	3GR4A		
3GR4A	CYS358(SG)	3.65	ASP357(C)	3GR4A		S[ ] ... C[ ]
3GR4A	CYS358(SG)	3.66	ASP357(O)	3GR4A	Weakly negative	S[ ] ... O[ ]
3GR4A	CYS358(SG)	4.53	ASP357(CB)	3GR4A		
3GR4A	CYS358(N)	3.62	ILE359(N)	3GR4A		N[ ] ... N[ ]
3GR4A	CYS358(N)	4.87	ILE359(CA)	3GR4A		
3GR4A	CYS358(CA)	2.37	ILE359(N)	3GR4A		C[ ] ... N[ ]
3GR4A	CYS358(CA)	3.74	ILE359(CA)	3GR4A		C[ ] ... C[ ]
3GR4A	CYS358(CA)	4.60	ILE359(C)	3GR4A		
3GR4A	CYS358(CA)	4.53	ILE359(O)	3GR4A		
3GR4A	CYS358(CA)	4.69	ILE359(CB)	3GR4A		
3GR4A	CYS358(CA)	4.96	ILE359(CG1)	3GR4A		
3GR4A	CYS358(CA)	4.77	ILE359(CG2)	3GR4A		
3GR4A	CYS358(C)	2.43	ILE359(CA)	3GR4A		C[ ] ... C[ ]
3GR4A	CYS358(C)	3.51	ILE359(C)	3GR4A		C[ ] ... C[ ]

3GR4A	CYS358(C)	3.78	ILE359(O)	3GR4A		C[ ] ... O[ ]
3GR4A	CYS358(C)	3.46	ILE359(CB)	3GR4A		C[ ] ... C[ ]
3GR4A	CYS358(C)	4.08	ILE359(CG1)	3GR4A		C[ ] ... C[ ]
3GR4A	CYS358(C)	3.56	ILE359(CG2)	3GR4A		C[ ] ... C[ ]
3GR4A	CYS358(O)	2.25	ILE359(N)	3GR4A	[D-A-AA]:29.6° [A-D-DD]:94.8° d_planarity:1.0° a_planarity:5.4°, bad a_angle(sp2)	O[ ] ... N[ ]
3GR4A	CYS358(O)	2.77	ILE359(CA)	3GR4A		O[ ] ... C[ ]
3GR4A	CYS358(O)	3.94	ILE359(C)	3GR4A		O[ ] ... C[ ]
3GR4A	CYS358(O)	4.49	ILE359(O)	3GR4A		O[ ] ... O[ ]
3GR4A	CYS358(O)	3.79	ILE359(CB)	3GR4A		O[ ] ... C[ ]
3GR4A	CYS358(O)	4.76	ILE359(CG1)	3GR4A		
3GR4A	CYS358(O)	3.59	ILE359(CG2)	3GR4A		O[ ] ... C[ ]
3GR4A	CYS358(CB)	3.01	ILE359(N)	3GR4A		C[ ] ... N[ ]
3GR4A	CYS358(CB)	4.24	ILE359(CA)	3GR4A		C[ ] ... C[ ]
3GR4A	CYS358(CB)	4.63	ILE359(C)	3GR4A		
3GR4A	CYS358(CB)	4.32	ILE359(O)	3GR4A		C[ ] ... O[ ]
3GR4A	CYS358(SG)	4.63	ILE359(N)	3GR4A	[D-A-AA]:20.6° [A-D-DD]:151.3° d_planarity:79.7°, maximum distance exceeded, bad a_angle(sp3), bad d_planarity	
3GR4A	CYS358(C)	4.55	MET360(N)	3GR4A		
3GR4A	CYS358(O)	4.72	MET360(N)	3GR4A	[D-A-AA]:74.7° [A-D-DD]:138.3° d_planarity:57.9° a_planarity:27.6°, maximum distance exceeded, bad a_angle(sp2)	
3GR4A	CYS358(SG)	4.42	HIS464(C)	3GR4A		S[ ] ... C[ ]
3GR4A	CYS358(SG)	3.50	HIS464(O)	3GR4A		S[ ] ... O[ ]
3GR4A	CYS358(N)	4.76	LEU465(O)	3GR4A	[D-A-AA]:127.0° [A-D-DD]:110.3° d_planarity:73.2° a_planarity:-85.4°, maximum distance exceeded, bad d_planarity	
3GR4A	CYS358(SG)	4.61	LEU465(N)	3GR4A	[D-A-AA]:172.8° [A-D-DD]:50.1° d_planarity:24.5°, maximum distance exceeded, bad d_angle(sp2)	
3GR4A	CYS358(SG)	3.84	LEU465(CA)	3GR4A		S[ ] ... C[ ]
3GR4A	CYS358(SG)	3.95	LEU465(C)	3GR4A		S[ ] ... C[ ]
3GR4A	CYS358(SG)	3.70	LEU465(O)	3GR4A		S[ ] ... O[ ]
3GR4A	CYS358(SG)	4.85	LEU465(CB)	3GR4A		
3GR4A	CYS358(SG)	4.45	LEU465(CD2)	3GR4A		S[ ] ... C[ ]
3GR4A	CYS358(SG)	4.90	TYR466(N)	3GR4A	[D-A-AA]:148.4° [A-D-DD]:118.6° d_planarity:45.2°, maximum distance exceeded	

chain1	res1/atm1	distance	res2/atm2	chain2	H-bonding	Charge interaction
1WYIA	CYS41(N)	4.42	GLY10(N)	1WYIA		N[ ] ... N[ ]
1WYIA	CYS41(N)	4.73	GLY10(O)	1WYIA	[D-A-AA]:113.6° [A-D-DD]:34.9° d_planarity:27.3° a_planarity:-36.2°, maximum distance exceeded, bad d_angle(sp2)	
1WYIA	CYS41(CA)	4.19	GLY10(N)	1WYIA		C[ ] ... N[ ]
1WYIA	CYS41(CA)	4.52	GLY10(CA)	1WYIA		
1WYIA	CYS41(CA)	4.46	GLY10(C)	1WYIA		C[ ] ... C[ ]
1WYIA	CYS41(CA)	3.64	GLY10(O)	1WYIA		C[ ] ... O[ ]
1WYIA	CYS41(C)	4.92	GLY10(N)	1WYIA		

1WYIA	CYS41(C)	4.68	GLY10(C)	1WYIA		
1WYIA	CYS41(C)	3.66	GLY10(O)	1WYIA		C[ ] ... O[ ]
1WYIA	CYS41(O)	4.85	GLY10(O)	1WYIA		
1WYIA	CYS41(CB)	4.42	GLY10(O)	1WYIA		C[ ] ... O[ ]
1WYIA	CYS41(CA)	4.65	TRP12(NE1)	1WYIA		
1WYIA	CYS41(CA)	4.81	TRP12(CE2)	1WYIA		
1WYIA	CYS41(CA)	4.84	TRP12(CZ2)	1WYIA		
1WYIA	CYS41(CB)	4.99	TRP12(CD2)	1WYIA		
1WYIA	CYS41(CB)	4.14	TRP12(NE1)	1WYIA		C[ ] ... N[ ]
1WYIA	CYS41(CB)	4.02	TRP12(CE2)	1WYIA		C[ ] ... C[ ]
1WYIA	CYS41(CB)	3.68	TRP12(CZ2)	1WYIA		C[ ] ... C[ ]
1WYIA	CYS41(CB)	4.41	TRP12(CH2)	1WYIA		C[ ] ... C[ ]
1WYIA	CYS41(SG)	4.32	TRP12(NE1)	1WYIA	[D-A-AA]:72.0° [A-D-DD]:146.3° d_planarity:-65.1°, maximum distance exceeded, bad d_planarity	S[ ] ... N[ ]
1WYIA	CYS41(SG)	4.47	TRP12(CE2)	1WYIA		S[ ] ... C[ ]
1WYIA	CYS41(SG)	4.00	TRP12(CZ2)	1WYIA		S[ ] ... C[ ]
1WYIA	CYS41(SG)	4.99	TRP12(CH2)	1WYIA		
1WYIA	CYS41(SG)	3.77	LEU28(CD1)	1WYIA		S[ ] ... C[ ]
1WYIA	CYS41(N)	4.50	VAL39(C)	1WYIA		N[ ] ... C[ ]
1WYIA	CYS41(N)	4.65	VAL39(O)	1WYIA	[D-A-AA]:75.3° [A-D-DD]:153.5° d_planarity:75.7° a_planarity:23.9°, maximum distance exceeded, bad a_angle(sp2), bad d_planarity	
1WYIA	CYS41(SG)	4.87	VAL39(CG1)	1WYIA		
1WYIA	CYS41(N)	3.58	VAL40(N)	1WYIA		N[ ] ... N[ ]
1WYIA	CYS41(N)	2.45	VAL40(CA)	1WYIA		N[ ] ... C[ ]
1WYIA	CYS41(N)	2.25	VAL40(O)	1WYIA	[D-A-AA]:29.9° [A-D-DD]:95.5° d_planarity:0.3° a_planarity:0.7°, bad a_angle(sp2)	N[ ] ... O[ ]
1WYIA	CYS41(N)	3.35	VAL40(CB)	1WYIA		N[ ] ... C[ ]
1WYIA	CYS41(N)	3.34	VAL40(CG1)	1WYIA		N[ ] ... C[ ]
1WYIA	CYS41(N)	4.72	VAL40(CG2)	1WYIA		
1WYIA	CYS41(CA)	4.83	VAL40(N)	1WYIA		
1WYIA	CYS41(CA)	3.84	VAL40(CA)	1WYIA		C[ ] ... C[ ]
1WYIA	CYS41(CA)	2.45	VAL40(C)	1WYIA		C[ ] ... C[ ]
1WYIA	CYS41(CA)	2.79	VAL40(O)	1WYIA		C[ ] ... O[ ]
1WYIA	CYS41(CA)	4.64	VAL40(CB)	1WYIA		
1WYIA	CYS41(CA)	4.50	VAL40(CG1)	1WYIA		
1WYIA	CYS41(C)	4.73	VAL40(CA)	1WYIA		
1WYIA	CYS41(C)	3.50	VAL40(C)	1WYIA		C[ ] ... C[ ]
1WYIA	CYS41(C)	3.90	VAL40(O)	1WYIA		C[ ] ... O[ ]
1WYIA	CYS41(C)	4.56	VAL40(CG1)	1WYIA		
1WYIA	CYS41(O)	5.00	VAL40(CA)	1WYIA		
1WYIA	CYS41(O)	4.06	VAL40(C)	1WYIA		O[ ] ... C[ ]
1WYIA	CYS41(O)	4.73	VAL40(O)	1WYIA		
1WYIA	CYS41(O)	4.43	VAL40(CG1)	1WYIA		O[ ] ... C[ ]
1WYIA	CYS41(CB)	4.71	VAL40(CA)	1WYIA		
1WYIA	CYS41(CB)	3.48	VAL40(C)	1WYIA		C[ ] ... C[ ]
1WYIA	CYS41(CB)	3.86	VAL40(O)	1WYIA		C[ ] ... O[ ]
1WYIA	CYS41(SG)	4.79	VAL40(CA)	1WYIA		
1WYIA	CYS41(SG)	3.72	VAL40(C)	1WYIA		S[ ] ... C[ ]
1WYIA	CYS41(SG)	3.86	VAL40(O)	1WYIA		S[ ] ... O[ ]

1WYIA	CYS41(N)	3.49	ALA42(N)	1WYIA		N[ ] ... N[ ]
1WYIA	CYS41(N)	4.74	ALA42(CA)	1WYIA		
1WYIA	CYS41(CA)	2.44	ALA42(N)	1WYIA		C[ ] ... N[ ]
1WYIA	CYS41(CA)	3.82	ALA42(CA)	1WYIA		C[ ] ... C[ ]
1WYIA	CYS41(CA)	4.66	ALA42(C)	1WYIA		
1WYIA	CYS41(CA)	4.77	ALA42(CB)	1WYIA		
1WYIA	CYS41(C)	2.43	ALA42(CA)	1WYIA		C[ ] ... C[ ]
1WYIA	CYS41(C)	3.39	ALA42(C)	1WYIA		C[ ] ... C[ ]
1WYIA	CYS41(C)	4.27	ALA42(O)	1WYIA		C[ ] ... O[ ]
1WYIA	CYS41(C)	3.56	ALA42(CB)	1WYIA		C[ ] ... C[ ]
1WYIA	CYS41(O)	2.25	ALA42(N)	1WYIA	[D-A-AA]:30.0° [A-D-DD]:93.5° d_planarity:0.1° a_planarity:0.6°, bad a_angle(sp2)	O[ ] ... N[ ]
1WYIA	CYS41(O)	2.76	ALA42(CA)	1WYIA		O[ ] ... C[ ]
1WYIA	CYS41(O)	3.68	ALA42(C)	1WYIA		O[ ] ... C[ ]
1WYIA	CYS41(O)	4.77	ALA42(O)	1WYIA		
1WYIA	CYS41(O)	3.98	ALA42(CB)	1WYIA		O[ ] ... C[ ]
1WYIA	CYS41(CB)	3.45	ALA42(N)	1WYIA		C[ ] ... N[ ]
1WYIA	CYS41(CB)	4.72	ALA42(CA)	1WYIA		
1WYIA	CYS41(SG)	4.97	ALA42(N)	1WYIA	[D-A-AA]:26.9° [A-D-DD]:157.8° d_planarity:50.6°, maximum distance exceeded, bad a_angle(sp3)	
1WYIA	CYS41(CA)	4.64	PRO43(CD)	1WYIA		
1WYIA	CYS41(C)	3.76	PRO43(N)	1WYIA		C[ ] ... N[ ]
1WYIA	CYS41(C)	4.29	PRO43(CG)	1WYIA		C[ ] ... C[ ]
1WYIA	CYS41(C)	3.37	PRO43(CD)	1WYIA		C[ ] ... C[ ]
1WYIA	CYS41(O)	3.72	PRO43(N)	1WYIA		O[ ] ... N[ ]
1WYIA	CYS41(O)	3.91	PRO43(CG)	1WYIA		O[ ] ... C[ ]
1WYIA	CYS41(O)	2.92	PRO43(CD)	1WYIA		O[ ] ... C[ ]
1WYIA	CYS41(CB)	4.83	PRO43(CG)	1WYIA		
1WYIA	CYS41(CB)	4.69	PRO43(CD)	1WYIA		
1WYIA	CYS41(CB)	4.61	LEU55(CD2)	1WYIA		
1WYIA	CYS41(N)	4.35	ILE59(CG2)	1WYIA		N[ ] ... C[ ]
1WYIA	CYS41(CB)	4.59	ILE59(CG2)	1WYIA		
1WYIA	CYS41(SG)	3.89	ILE59(CG2)	1WYIA		S[ ] ... C[ ]
1WYIA	CYS41(N)	4.21	ALA60(C)	1WYIA		N[ ] ... C[ ]
1WYIA	CYS41(N)	3.04	ALA60(O)	1WYIA	**H-bond** [D-A-AA]:158.4° [A-D-DD]:124.7° d_planarity:-8.5° a_planarity:66.2°	N[ ] ... O[ ]
1WYIA	CYS41(CA)	4.05	ALA60(O)	1WYIA		C[ ] ... O[ ]
1WYIA	CYS41(C)	4.41	ALA60(O)	1WYIA		C[ ] ... O[ ]
1WYIA	CYS41(O)	4.64	ALA60(C)	1WYIA		
1WYIA	CYS41(O)	3.87	ALA60(O)	1WYIA		O[ ] ... O[ ]
1WYIA	CYS41(CB)	4.21	ALA60(O)	1WYIA		C[ ] ... O[ ]
1WYIA	CYS41(SG)	4.92	ALA60(O)	1WYIA		
1WYIA	CYS41(N)	4.93	VAL61(N)	1WYIA		
1WYIA	CYS41(N)	4.68	VAL61(CA)	1WYIA		
1WYIA	CYS41(C)	4.63	VAL61(CA)	1WYIA		
1WYIA	CYS41(C)	4.74	VAL61(C)	1WYIA		
1WYIA	CYS41(O)	4.57	VAL61(N)	1WYIA	[D-A-AA]:137.0° [A-D-DD]:36.8° d_planarity:14.9° a_planarity:-24.8°, maximum distance exceeded, bad d_angle(sp2)	
1WYIA	CYS41(O)	3.52	VAL61(CA)	1WYIA		O[ ] ... C[ ]

1WYIA	CYS41(O)	3.62	VAL61(C)	1WYIA		O[ ] ... C[ ]
1WYIA	CYS41(O)	4.80	VAL61(O)	1WYIA		
1WYIA	CYS41(O)	4.34	VAL61(CB)	1WYIA		O[ ] ... C[ ]
1WYIA	CYS41(O)	4.10	VAL61(CG1)	1WYIA		O[ ] ... C[ ]
1WYIA	CYS41(O)	4.82	VAL61(CG2)	1WYIA		
1WYIA	CYS41(N)	4.71	ALA62(N)	1WYIA		
1WYIA	CYS41(CA)	4.94	ALA62(N)	1WYIA		
1WYIA	CYS41(C)	3.90	ALA62(N)	1WYIA		C[ ] ... N[ ]
1WYIA	CYS41(C)	4.59	ALA62(CA)	1WYIA		
1WYIA	CYS41(C)	4.90	ALA62(C)	1WYIA		
1WYIA	CYS41(C)	4.25	ALA62(O)	1WYIA		C[ ] ... O[ ]
1WYIA	CYS41(C)	4.35	ALA62(CB)	1WYIA		C[ ] ... C[ ]
1WYIA	CYS41(O)	2.83	ALA62(N)	1WYIA	**H-bond** [D-A-AA]:144.1° [A-D-DD]:118.4° d_planarity:-20.0° a_planarity:72.2°	O[ ] ... N[ ]
1WYIA	CYS41(O)	3.75	ALA62(CA)	1WYIA		O[ ] ... C[ ]
1WYIA	CYS41(O)	4.12	ALA62(C)	1WYIA		O[ ] ... C[ ]
1WYIA	CYS41(O)	3.57	ALA62(O)	1WYIA		O[ ] ... O[ ]
1WYIA	CYS41(O)	3.85	ALA62(CB)	1WYIA		O[ ] ... C[ ]
1WYIA	CYS41(SG)	4.99	PHE240(CD2)	1WYIA		
1WYIA	CYS41(SG)	4.14	PHE240(CE2)	1WYIA		S[ ] ... C[ ]
1WYIA	CYS41(SG)	4.47	PHE240(CZ)	1WYIA		S[ ] ... C[ ]

chain1	res1/atm1	distance	res2/atm2	chain2	H-bonding	Charge interaction
1WYIA	CYS86(O)	4.63	ILE48(O)	1WYIA		
1WYIA	CYS86(CB)	4.90	ILE48(C)	1WYIA		
1WYIA	CYS86(CB)	4.97	ILE48(CB)	1WYIA		
1WYIA	CYS86(CB)	4.11	ILE48(CG2)	1WYIA		C[ ] ... C[ ]
1WYIA	CYS86(SG)	4.83	ILE48(CG2)	1WYIA		
1WYIA	CYS86(CA)	4.38	ASP49(OD1)	1WYIA		C[ ] ... O[-]
1WYIA	CYS86(C)	4.64	ASP49(OD1)	1WYIA		
1WYIA	CYS86(O)	4.53	ASP49(CA)	1WYIA		
1WYIA	CYS86(O)	4.08	ASP49(OD1)	1WYIA		O[ ] ... O[-]
1WYIA	CYS86(CB)	4.52	ASP49(N)	1WYIA		
1WYIA	CYS86(CB)	4.35	ASP49(CA)	1WYIA		C[ ] ... C[ ]
1WYIA	CYS86(CB)	4.40	ASP49(CB)	1WYIA		C[ ] ... C[ ]
1WYIA	CYS86(CB)	4.55	ASP49(CG)	1WYIA		
1WYIA	CYS86(CB)	4.24	ASP49(OD1)	1WYIA		C[ ] ... O[-]
1WYIA	CYS86(SG)	4.98	ASP49(N)	1WYIA	[D-A-AA]:65.1° [A-D-DD]:79.3° d_planarity:-69.5°, maximum distance exceeded, bad d_angle(sp2), bad d_planarity	
1WYIA	CYS86(SG)	4.92	ASP49(CA)	1WYIA		
1WYIA	CYS86(SG)	4.46	ASP49(CB)	1WYIA		S[ ] ... C[ ]
1WYIA	CYS86(SG)	4.67	ASP49(CG)	1WYIA		
1WYIA	CYS86(SG)	4.78	ASP49(OD1)	1WYIA	Negative	
1WYIA	CYS86(C)	4.74	ARG52(CB)	1WYIA		
1WYIA	CYS86(C)	4.37	ARG52(CG)	1WYIA		C[ ] ... C[ ]
1WYIA	CYS86(O)	3.55	ARG52(CB)	1WYIA		O[ ] ... C[ ]
1WYIA	CYS86(O)	3.33	ARG52(CG)	1WYIA		O[ ] ... C[ ]

1WYIA	CYS86(O)	4.42	ARG52(CD)	1WYIA		O[ ] ... C[ ]
1WYIA	CYS86(N)	4.95	GLY81(O)	1WYIA	[D-A-AA]:128.9° [A-D-DD]:164.2° d_planarity:-41.2° a_planarity:35.6°, maximum distance exceeded	
1WYIA	CYS86(N)	4.97	MET82(CA)	1WYIA		
1WYIA	CYS86(N)	3.97	MET82(C)	1WYIA		N[ ] ... C[ ]
1WYIA	CYS86(N)	2.77	MET82(O)	1WYIA	**H-bond** [D-A-AA]:165.3° [A-D-DD]:123.0° d_planarity:-41.9° a_planarity:71.8°	N[ ] ... O[ ]
1WYIA	CYS86(CA)	4.97	MET82(C)	1WYIA		
1WYIA	CYS86(CA)	3.76	MET82(O)	1WYIA		C[ ] ... O[ ]
1WYIA	CYS86(C)	4.86	MET82(O)	1WYIA		
1WYIA	CYS86(CB)	4.69	MET82(C)	1WYIA		
1WYIA	CYS86(CB)	3.61	MET82(O)	1WYIA		C[ ] ... O[ ]
1WYIA	CYS86(SG)	4.96	MET82(CA)	1WYIA		
1WYIA	CYS86(SG)	4.13	MET82(C)	1WYIA		S[ ] ... C[ ]
1WYIA	CYS86(SG)	3.22	MET82(O)	1WYIA		S[ ] ... O[ ]
1WYIA	CYS86(N)	4.76	ILE83(N)	1WYIA		
1WYIA	CYS86(N)	4.61	ILE83(CA)	1WYIA		
1WYIA	CYS86(N)	3.79	ILE83(C)	1WYIA		N[ ] ... C[ ]
1WYIA	CYS86(N)	3.33	ILE83(O)	1WYIA	**H-bond** [D-A-AA]:102.1° [A-D-DD]:99.3° d_planarity:-21.5° a_planarity:79.3°	N[ ] ... O[ ]
1WYIA	CYS86(CA)	4.58	ILE83(C)	1WYIA		
1WYIA	CYS86(CA)	3.84	ILE83(O)	1WYIA		C[ ] ... O[ ]
1WYIA	CYS86(C)	4.89	ILE83(C)	1WYIA		
1WYIA	CYS86(C)	3.86	ILE83(O)	1WYIA		C[ ] ... O[ ]
1WYIA	CYS86(O)	4.64	ILE83(O)	1WYIA		
1WYIA	CYS86(CB)	4.76	ILE83(CA)	1WYIA		
1WYIA	CYS86(CB)	4.50	ILE83(C)	1WYIA		C[ ] ... C[ ]
1WYIA	CYS86(CB)	3.83	ILE83(O)	1WYIA		C[ ] ... O[ ]
1WYIA	CYS86(SG)	4.85	ILE83(N)	1WYIA	[D-A-AA]:91.1° [A-D-DD]:81.5° d_planarity:31.9°, maximum distance exceeded, bad d_angle(sp2)	
1WYIA	CYS86(SG)	4.85	ILE83(CA)	1WYIA		
1WYIA	CYS86(SG)	4.76	ILE83(O)	1WYIA		
1WYIA	CYS86(N)	4.19	LYS84(N)	1WYIA		N[ ] ... N[ ]
1WYIA	CYS86(N)	4.13	LYS84(CA)	1WYIA		N[ ] ... C[ ]
1WYIA	CYS86(N)	2.96	LYS84(C)	1WYIA		N[ ] ... C[ ]
1WYIA	CYS86(N)	2.90	LYS84(O)	1WYIA	[D-A-AA]:80.6° [A-D-DD]:120.6° d_planarity:-87.0° a_planarity:49.4°, bad a_angle(sp2), bad d_planarity	N[ ] ... O[ ]
1WYIA	CYS86(CA)	4.18	LYS84(C)	1WYIA		C[ ] ... C[ ]
1WYIA	CYS86(CA)	3.85	LYS84(O)	1WYIA		C[ ] ... O[ ]
1WYIA	CYS86(C)	4.33	LYS84(C)	1WYIA		C[ ] ... C[ ]
1WYIA	CYS86(C)	3.66	LYS84(O)	1WYIA		C[ ] ... O[ ]
1WYIA	CYS86(O)	4.79	LYS84(O)	1WYIA		
1WYIA	CYS86(CB)	4.96	LYS84(O)	1WYIA		
1WYIA	CYS86(N)	2.73	ASP85(N)	1WYIA		N[ ] ... N[ ]
1WYIA	CYS86(N)	2.43	ASP85(CA)	1WYIA		N[ ] ... C[ ]
1WYIA	CYS86(N)	2.24	ASP85(O)	1WYIA	[D-A-AA]:29.8° [A-D-DD]:94.4° d_planarity:0.3° a_planarity:0.4°, bad a_angle(sp2)	N[ ] ... O[ ]
1WYIA	CYS86(N)	3.30	ASP85(CB)	1WYIA		N[ ] ... C[ ]
1WYIA	CYS86(N)	4.12	ASP85(CG)	1WYIA		N[ ] ... C[ ]
1WYIA	CYS86(N)	4.84	ASP85(OD1)	1WYIA	[D-A-AA]:48.8° [A-D-DD]:129.6° d_planarity:21.3° a_planarity:53.4°, maximum distance exceeded,	

					bad a_angle(sp2)	
1WYIA	CYS86(N)	4.53	ASP85(OD2)	1WYIA	[D-A-AA]:63.3° [A-D-DD]:116.2° d_planarity:49.1° a_planarity:46.2°, maximum distance exceeded, bad a_angle(sp2)	
1WYIA	CYS86(CA)	4.16	ASP85(N)	1WYIA		C[ ] ... N[ ]
1WYIA	CYS86(CA)	3.80	ASP85(CA)	1WYIA		C[ ] ... C[ ]
1WYIA	CYS86(CA)	2.43	ASP85(C)	1WYIA		C[ ] ... C[ ]
1WYIA	CYS86(CA)	2.76	ASP85(O)	1WYIA		C[ ] ... O[ ]
1WYIA	CYS86(CA)	4.59	ASP85(CB)	1WYIA		
1WYIA	CYS86(C)	4.75	ASP85(N)	1WYIA		
1WYIA	CYS86(C)	4.61	ASP85(CA)	1WYIA		
1WYIA	CYS86(C)	3.33	ASP85(C)	1WYIA		C[ ] ... C[ ]
1WYIA	CYS86(C)	3.55	ASP85(O)	1WYIA		C[ ] ... O[ ]
1WYIA	CYS86(O)	4.47	ASP85(C)	1WYIA		O[ ] ... C[ ]
1WYIA	CYS86(O)	4.55	ASP85(O)	1WYIA		
1WYIA	CYS86(CB)	4.96	ASP85(N)	1WYIA		
1WYIA	CYS86(CB)	4.80	ASP85(CA)	1WYIA		
1WYIA	CYS86(CB)	3.62	ASP85(C)	1WYIA		C[ ] ... C[ ]
1WYIA	CYS86(CB)	4.09	ASP85(O)	1WYIA		C[ ] ... O[ ]
1WYIA	CYS86(SG)	4.95	ASP85(CA)	1WYIA		
1WYIA	CYS86(SG)	4.03	ASP85(C)	1WYIA		S[ ] ... C[ ]
1WYIA	CYS86(SG)	4.49	ASP85(O)	1WYIA		S[ ] ... O[ ]
1WYIA	CYS86(SG)	4.89	ASP85(CB)	1WYIA		
1WYIA	CYS86(N)	2.70	GLY87(N)	1WYIA		N[ ] ... N[ ]
1WYIA	CYS86(N)	4.15	GLY87(CA)	1WYIA		N[ ] ... C[ ]
1WYIA	CYS86(N)	4.95	GLY87(C)	1WYIA		
1WYIA	CYS86(CA)	2.45	GLY87(N)	1WYIA		C[ ] ... N[ ]
1WYIA	CYS86(CA)	3.81	GLY87(CA)	1WYIA		C[ ] ... C[ ]
1WYIA	CYS86(CA)	4.64	GLY87(C)	1WYIA		
1WYIA	CYS86(C)	2.41	GLY87(CA)	1WYIA		C[ ] ... C[ ]
1WYIA	CYS86(C)	3.28	GLY87(C)	1WYIA		C[ ] ... C[ ]
1WYIA	CYS86(C)	4.41	GLY87(O)	1WYIA		C[ ] ... O[ ]
1WYIA	CYS86(O)	2.23	GLY87(N)	1WYIA	[D-A-AA]:30.3° [A-D-DD]:92.1° d_planarity:0.5° a_planarity:0.3°, bad a_angle(sp2)	O[ ] ... N[ ]
1WYIA	CYS86(O)	2.71	GLY87(CA)	1WYIA		O[ ] ... C[ ]
1WYIA	CYS86(O)	3.37	GLY87(C)	1WYIA		O[ ] ... C[ ]
1WYIA	CYS86(O)	4.31	GLY87(O)	1WYIA		O[ ] ... O[ ]
1WYIA	CYS86(CB)	3.58	GLY87(N)	1WYIA		C[ ] ... N[ ]
1WYIA	CYS86(CB)	4.79	GLY87(CA)	1WYIA		
1WYIA	CYS86(N)	4.60	ALA88(N)	1WYIA		
1WYIA	CYS86(CA)	4.37	ALA88(N)	1WYIA		C[ ] ... N[ ]
1WYIA	CYS86(C)	3.27	ALA88(N)	1WYIA		C[ ] ... N[ ]
1WYIA	CYS86(C)	4.63	ALA88(CA)	1WYIA		
1WYIA	CYS86(C)	4.96	ALA88(CB)	1WYIA		
1WYIA	CYS86(O)	3.46	ALA88(N)	1WYIA	[D-A-AA]:71.0° [A-D-DD]:140.4° d_planarity:-75.9° a_planarity:47.3°, bad a_angle(sp2), bad d_planarity	O[ ] ... N[ ]
1WYIA	CYS86(O)	4.68	ALA88(CA)	1WYIA		
1WYIA	CYS86(O)	4.99	ALA88(CB)	1WYIA		
1WYIA	CYS86(CB)	4.67	ALA88(N)	1WYIA		
1WYIA	CYS86(CA)	4.61	LYS18(CE)	1WYIB		

1WYIA	CYS86(CA)	4.66	LYS18(NZ)	1WYIB		
1WYIA	CYS86(SG)	4.81	LYS18(CE)	1WYIB	positive	
1WYIA	CYS86(SG)	4.86	ALA46(C)	1WYIB		
1WYIA	CYS86(SG)	4.36	ALA46(O)	1WYIB		S[ ] ... O[ ]
1WYIA	CYS86(SG)	4.29	ALA46(CB)	1WYIB		S[ ] ... C[ ]
1WYIA	CYS86(N)	4.78	TYR47(OH)	1WYIB	[D-A-AA]:115.4° [A-D-DD]:117.0° d_planarity:85.2° a_planarity:-16.8°, maximum distance exceeded, bad d_planarity	
1WYIA	CYS86(SG)	4.10	TYR47(CD1)	1WYIB		S[ ] ... C[ ]
1WYIA	CYS86(SG)	3.41	TYR47(CE1)	1WYIB		S[ ] ... C[ ]
1WYIA	CYS86(SG)	4.15	TYR47(CZ)	1WYIB		S[ ] ... C[ ]
1WYIA	CYS86(SG)	4.21	TYR47(OH)	1WYIB	[D-A-AA]:129.8° [A-D-DD]:78.0° d_planarity:-34.2°, maximum distance exceeded, bad d_angle(sp2)	S[ ] ... O[ ]

chain1	res1/atm1	distance	res2/atm2	chain2	H-bonding	Charge interaction
1WYIA	CYS126(SG)	3.68	VAL92(CG1)	1WYIA		S[ ] ... C[ ]
1WYIA	CYS126(N)	4.82	LEU93(N)	1WYIA		
1WYIA	CYS126(N)	4.91	LEU93(CA)	1WYIA		
1WYIA	CYS126(N)	3.92	LEU93(C)	1WYIA		N[ ] ... C[ ]
1WYIA	CYS126(N)	2.69	LEU93(O)	1WYIA	**H-bond** [D-A-AA]:178.6° [A-D-DD]:115.8° d_planarity:-5.4° a_planarity:7.6°	N[ ] ... O[ ]
1WYIA	CYS126(CA)	4.74	LEU93(C)	1WYIA		
1WYIA	CYS126(CA)	3.58	LEU93(O)	1WYIA		C[ ] ... O[ ]
1WYIA	CYS126(C)	4.31	LEU93(O)	1WYIA		C[ ] ... O[ ]
1WYIA	CYS126(O)	4.95	LEU93(C)	1WYIA		
1WYIA	CYS126(O)	4.05	LEU93(O)	1WYIA		O[ ] ... O[ ]
1WYIA	CYS126(CB)	4.42	LEU93(C)	1WYIA		C[ ] ... C[ ]
1WYIA	CYS126(CB)	3.47	LEU93(O)	1WYIA		C[ ] ... O[ ]
1WYIA	CYS126(SG)	4.80	LEU93(N)	1WYIA	[D-A-AA]:103.5° [A-D-DD]:102.8° d_planarity:-62.8°, maximum distance exceeded, bad d_planarity	
1WYIA	CYS126(SG)	4.55	LEU93(C)	1WYIA		
1WYIA	CYS126(SG)	3.76	LEU93(O)	1WYIA		S[ ] ... O[ ]
1WYIA	CYS126(N)	4.76	GLY94(N)	1WYIA		
1WYIA	CYS126(N)	4.64	GLY94(CA)	1WYIA		
1WYIA	CYS126(N)	4.65	GLY94(C)	1WYIA		
1WYIA	CYS126(N)	4.73	GLY94(O)	1WYIA	[D-A-AA]:78.8° [A-D-DD]:60.8° d_planarity:-11.9° a_planarity:-76.8°, maximum distance exceeded, bad a_angle(sp2), bad d_angle(sp2)	
1WYIA	CYS126(CA)	4.87	GLY94(CA)	1WYIA		
1WYIA	CYS126(CA)	4.47	GLY94(C)	1WYIA		C[ ] ... C[ ]
1WYIA	CYS126(CA)	4.21	GLY94(O)	1WYIA		C[ ] ... O[ ]
1WYIA	CYS126(C)	4.87	GLY94(CA)	1WYIA		
1WYIA	CYS126(C)	4.51	GLY94(C)	1WYIA		
1WYIA	CYS126(C)	3.93	GLY94(O)	1WYIA		C[ ] ... O[ ]
1WYIA	CYS126(O)	4.08	GLY94(CA)	1WYIA		O[ ] ... C[ ]
1WYIA	CYS126(O)	3.95	GLY94(C)	1WYIA		O[ ] ... C[ ]
1WYIA	CYS126(O)	3.35	GLY94(O)	1WYIA		O[ ] ... O[ ]
1WYIA	CYS126(CB)	4.84	GLY94(N)	1WYIA		

1WYIA	CYS126(CB)	4.38	GLY94(CA)	1WYIA		C[ ] ... C[ ]
1WYIA	CYS126(CB)	3.58	GLY94(C)	1WYIA		C[ ] ... C[ ]
1WYIA	CYS126(CB)	3.38	GLY94(O)	1WYIA		C[ ] ... O[ ]
1WYIA	CYS126(SG)	4.38	GLY94(C)	1WYIA		S[ ] ... C[ ]
1WYIA	CYS126(SG)	4.55	GLY94(O)	1WYIA		
1WYIA	CYS126(CA)	4.96	HIS95(N)	1WYIA		
1WYIA	CYS126(O)	4.99	HIS95(N)	1WYIA	[D-A-AA]:98.6° [A-D-DD]:101.4° d_planarity:31.1° a_planarity:-44.2°, maximum distance exceeded	
1WYIA	CYS126(CB)	3.79	HIS95(N)	1WYIA		C[ ] ... N[ ]
1WYIA	CYS126(CB)	3.78	HIS95(CA)	1WYIA		C[ ] ... C[ ]
1WYIA	CYS126(CB)	4.71	HIS95(C)	1WYIA		
1WYIA	CYS126(CB)	4.78	HIS95(CB)	1WYIA		
1WYIA	CYS126(SG)	4.05	HIS95(N)	1WYIA	[D-A-AA]:68.8° [A-D-DD]:73.2° d_planarity:87.2°, maximum distance exceeded, bad d_angle(sp2), bad d_planarity	S[ ] ... N[ ]
1WYIA	CYS126(SG)	3.89	HIS95(CA)	1WYIA		S[ ] ... C[ ]
1WYIA	CYS126(SG)	4.31	HIS95(CB)	1WYIA		S[ ] ... C[ ]
1WYIA	CYS126(SG)	4.53	HIS95(CG)	1WYIA		
1WYIA	CYS126(SG)	4.12	HIS95(CD2)	1WYIA	Weakly positive	S[ ] ... C[ ]
1WYIA	CYS126(CB)	4.81	SER96(N)	1WYIA		
1WYIA	CYS126(C)	4.54	ARG99(NE)	1WYIA		
1WYIA	CYS126(C)	4.82	ARG99(CZ)	1WYIA		
1WYIA	CYS126(C)	4.18	ARG99(NH2)	1WYIA		C[ ] ... N[+]
1WYIA	CYS126(O)	4.81	ARG99(CD)	1WYIA		
1WYIA	CYS126(O)	3.51	ARG99(NE)	1WYIA	[D-A-AA]:141.5° [A-D-DD]:85.3° d_planarity:-13.7° a_planarity:72.4°, maximum distance exceeded, bad d_angle(sp2)	O[ ] ... N[ ]
1WYIA	CYS126(O)	3.65	ARG99(CZ)	1WYIA		O[ ] ... C[ ]
1WYIA	CYS126(O)	4.93	ARG99(NH1)	1WYIA	[D-A-AA]:158.6° [A-D-DD]:13.9° d_planarity:51.7° a_planarity:33.6°, maximum distance exceeded, bad d_angle(sp2)	
1WYIA	CYS126(O)	2.96	ARG99(NH2)	1WYIA	**H-bond** [D-A-AA]:170.8° [A-D-DD]:110.8° d_planarity:-19.6° a_planarity:23.5°	O[ ] ... N[+]
1WYIA	CYS126(N)	3.82	ILE124(C)	1WYIA		N[ ] ... C[ ]
1WYIA	CYS126(N)	3.51	ILE124(O)	1WYIA	[D-A-AA]:94.9° [A-D-DD]:134.1° d_planarity:-82.7° a_planarity:27.3°, maximum distance exceeded, bad d_planarity	N[ ] ... O[ ]
1WYIA	CYS126(CA)	4.65	ILE124(O)	1WYIA		
1WYIA	CYS126(CB)	4.81	ILE124(O)	1WYIA		
1WYIA	CYS126(SG)	4.79	ILE124(C)	1WYIA		
1WYIA	CYS126(SG)	4.02	ILE124(O)	1WYIA		S[ ] ... O[ ]
1WYIA	CYS126(SG)	4.35	ILE124(CG2)	1WYIA		S[ ] ... C[ ]
1WYIA	CYS126(N)	3.42	ALA125(N)	1WYIA		N[ ] ... N[ ]
1WYIA	CYS126(N)	2.41	ALA125(CA)	1WYIA		N[ ] ... C[ ]
1WYIA	CYS126(N)	2.26	ALA125(O)	1WYIA	[D-A-AA]:29.3° [A-D-DD]:96.0° d_planarity:1.4° a_planarity:1.7°, bad a_angle(sp2)	N[ ] ... O[ ]
1WYIA	CYS126(N)	3.48	ALA125(CB)	1WYIA		N[ ] ... C[ ]
1WYIA	CYS126(CA)	4.69	ALA125(N)	1WYIA		
1WYIA	CYS126(CA)	3.81	ALA125(CA)	1WYIA		C[ ] ... C[ ]
1WYIA	CYS126(CA)	2.45	ALA125(C)	1WYIA		C[ ] ... C[ ]
1WYIA	CYS126(CA)	2.82	ALA125(O)	1WYIA		C[ ] ... O[ ]
1WYIA	CYS126(CA)	4.76	ALA125(CB)	1WYIA		

1WYIA	CYS126(C)	4.70	ALA125(CA)	1WYIA		
1WYIA	CYS126(C)	3.40	ALA125(C)	1WYIA		C[ ] ... C[ ]
1WYIA	CYS126(C)	3.61	ALA125(O)	1WYIA		C[ ] ... O[ ]
1WYIA	CYS126(O)	4.95	ALA125(CA)	1WYIA		
1WYIA	CYS126(O)	3.92	ALA125(C)	1WYIA		O[ ] ... C[ ]
1WYIA	CYS126(O)	4.36	ALA125(O)	1WYIA		O[ ] ... O[ ]
1WYIA	CYS126(CB)	4.77	ALA125(CA)	1WYIA		
1WYIA	CYS126(CB)	3.64	ALA125(C)	1WYIA		C[ ] ... C[ ]
1WYIA	CYS126(CB)	4.16	ALA125(O)	1WYIA		C[ ] ... O[ ]
1WYIA	CYS126(SG)	4.95	ALA125(CA)	1WYIA		
1WYIA	CYS126(SG)	4.07	ALA125(C)	1WYIA		S[ ] ... C[ ]
1WYIA	CYS126(SG)	4.62	ALA125(O)	1WYIA		
1WYIA	CYS126(N)	3.58	ILE127(N)	1WYIA		N[ ] ... N[ ]
1WYIA	CYS126(N)	4.82	ILE127(CA)	1WYIA		
1WYIA	CYS126(CA)	2.40	ILE127(N)	1WYIA		C[ ] ... N[ ]
1WYIA	CYS126(CA)	3.74	ILE127(CA)	1WYIA		C[ ] ... C[ ]
1WYIA	CYS126(CA)	4.68	ILE127(C)	1WYIA		
1WYIA	CYS126(CA)	4.56	ILE127(O)	1WYIA		
1WYIA	CYS126(CA)	4.59	ILE127(CB)	1WYIA		
1WYIA	CYS126(CA)	4.71	ILE127(CG1)	1WYIA		
1WYIA	CYS126(CA)	4.61	ILE127(CG2)	1WYIA		
1WYIA	CYS126(C)	2.38	ILE127(CA)	1WYIA		C[ ] ... C[ ]
1WYIA	CYS126(C)	3.50	ILE127(C)	1WYIA		C[ ] ... C[ ]
1WYIA	CYS126(C)	3.72	ILE127(O)	1WYIA		C[ ] ... O[ ]
1WYIA	CYS126(C)	3.31	ILE127(CB)	1WYIA		C[ ] ... C[ ]
1WYIA	CYS126(C)	3.80	ILE127(CG1)	1WYIA		C[ ] ... C[ ]
1WYIA	CYS126(C)	3.35	ILE127(CG2)	1WYIA		C[ ] ... C[ ]
1WYIA	CYS126(C)	4.79	ILE127(CD1)	1WYIA		
1WYIA	CYS126(O)	2.24	ILE127(N)	1WYIA	[D-A-AA]:29.9° [A-D-DD]:91.3° d_planarity:0.6° a_planarity:1.9°, bad a_angle(sp2)	O[ ] ... N[ ]
1WYIA	CYS126(O)	2.69	ILE127(CA)	1WYIA		O[ ] ... C[ ]
1WYIA	CYS126(O)	3.92	ILE127(C)	1WYIA		O[ ] ... C[ ]
1WYIA	CYS126(O)	4.39	ILE127(O)	1WYIA		O[ ] ... O[ ]
1WYIA	CYS126(O)	3.53	ILE127(CB)	1WYIA		O[ ] ... C[ ]
1WYIA	CYS126(O)	4.39	ILE127(CG1)	1WYIA		O[ ] ... C[ ]
1WYIA	CYS126(O)	3.19	ILE127(CG2)	1WYIA		O[ ] ... C[ ]
1WYIA	CYS126(CB)	3.24	ILE127(N)	1WYIA		C[ ] ... N[ ]
1WYIA	CYS126(CB)	4.45	ILE127(CA)	1WYIA		C[ ] ... C[ ]
1WYIA	CYS126(CB)	4.73	ILE127(O)	1WYIA		
1WYIA	CYS126(SG)	4.82	ILE127(N)	1WYIA	[D-A-AA]:22.4° [A-D-DD]:151.4° d_planarity:58.1°, maximum distance exceeded, bad a_angle(sp3)	
1WYIA	CYS126(C)	4.62	GLY128(N)	1WYIA		
1WYIA	CYS126(O)	4.79	GLY128(N)	1WYIA	[D-A-AA]:74.7° [A-D-DD]:149.5° d_planarity:45.3° a_planarity:18.1°, maximum distance exceeded, bad a_angle(sp2)	
1WYIA	CYS126(N)	4.74	ILE150(CD1)	1WYIA		
1WYIA	CYS126(C)	4.92	ILE150(CD1)	1WYIA		
1WYIA	CYS126(O)	4.52	ILE150(CD1)	1WYIA		
1WYIA	CYS126(N)	4.77	ALA163(N)	1WYIA		
1WYIA	CYS126(N)	4.50	ALA163(O)	1WYIA	[D-A-AA]:128.4° [A-D-DD]:37.2° d_planarity:10.2°	N[ ] ... O[ ]

					a_planarity:-7.4°, maximum distance exceeded, bad d_angle(sp2)	
1WYIA	CYS126(N)	4.90	ALA163(CB)	1WYIA		
1WYIA	CYS126(CA)	4.57	ALA163(N)	1WYIA		
1WYIA	CYS126(CA)	4.76	ALA163(CA)	1WYIA		
1WYIA	CYS126(CA)	4.47	ALA163(C)	1WYIA		C[ ] ... C[ ]
1WYIA	CYS126(CA)	3.45	ALA163(O)	1WYIA		C[ ] ... O[ ]
1WYIA	CYS126(CA)	4.41	ALA163(CB)	1WYIA		C[ ] ... C[ ]
1WYIA	CYS126(C)	4.84	ALA163(C)	1WYIA		
1WYIA	CYS126(C)	3.66	ALA163(O)	1WYIA		C[ ] ... O[ ]
1WYIA	CYS126(O)	4.87	ALA163(O)	1WYIA		
1WYIA	CYS126(CB)	4.15	ALA163(O)	1WYIA		C[ ] ... O[ ]
1WYIA	CYS126(CB)	4.73	ALA163(CB)	1WYIA		
1WYIA	CYS126(SG)	4.83	ALA163(O)	1WYIA		
1WYIA	CYS126(SG)	4.27	ALA163(CB)	1WYIA		S[ ] ... C[ ]
1WYIA	CYS126(CA)	4.06	GLU165(OE1)	1WYIA		C[ ] ... O[-]
1WYIA	CYS126(C)	4.20	GLU165(OE1)	1WYIA		C[ ] ... O[-]
1WYIA	CYS126(O)	4.78	GLU165(OE1)	1WYIA		
1WYIA	CYS126(CB)	4.83	GLU165(CG)	1WYIA		
1WYIA	CYS126(CB)	4.08	GLU165(CD)	1WYIA		C[ ] ... C[ ]
1WYIA	CYS126(CB)	2.94	GLU165(OE1)	1WYIA		C[ ] ... O[-]
1WYIA	CYS126(CB)	4.92	GLU165(OE2)	1WYIA		
1WYIA	CYS126(SG)	4.37	GLU165(CD)	1WYIA		S[ ] ... C[ ]
1WYIA	CYS126(SG)	3.55	GLU165(OE1)	1WYIA	negative	S[ ] ... O[-]
1WYIA	CYS126(SG)	3.90	LEU230(CD2)	1WYIA		S[ ] ... C[ ]

chain1	res1/atm1	distance	res2/atm2	chain2	H-bonding	Charge interaction
1IRIA	CYS404(N)	4.99	HIS336(ND1)	1IRIA		
1IRIA	CYS404(N)	4.97	HIS336(CE1)	1IRIA		
1IRIA	CYS404(C)	4.93	HIS336(CE1)	1IRIA		
1IRIA	CYS404(N)	4.68	ALA337(N)	1IRIA		
1IRIA	CYS404(N)	4.38	ALA337(O)	1IRIA	[D-A-AA]:127.3° [A-D-DD]:37.7° d_planarity:33.4° a_planarity:-22.9°, maximum distance exceeded, bad d_angle(sp2)	N[ ] ... O[ ]
1IRIA	CYS404(N)	4.90	ALA337(CB)	1IRIA		
1IRIA	CYS404(CA)	4.33	ALA337(N)	1IRIA		C[ ] ... N[ ]
1IRIA	CYS404(CA)	4.48	ALA337(CA)	1IRIA		C[ ] ... C[ ]
1IRIA	CYS404(CA)	4.23	ALA337(C)	1IRIA		C[ ] ... C[ ]
1IRIA	CYS404(CA)	3.35	ALA337(O)	1IRIA		C[ ] ... O[ ]
1IRIA	CYS404(CA)	4.14	ALA337(CB)	1IRIA		C[ ] ... C[ ]
1IRIA	CYS404(C)	4.67	ALA337(C)	1IRIA		
1IRIA	CYS404(C)	3.56	ALA337(O)	1IRIA		C[ ] ... O[ ]
1IRIA	CYS404(O)	4.77	ALA337(O)	1IRIA		
1IRIA	CYS404(CB)	4.86	ALA337(C)	1IRIA		
1IRIA	CYS404(CB)	4.21	ALA337(O)	1IRIA		C[ ] ... O[ ]

1IRIA	CYS404(CB)	4.23	ALA337(CB)	1IRIA		C[ ] ... C[ ]
1IRIA	CYS404(SG)	4.82	ALA337(CA)	1IRIA		
1IRIA	CYS404(SG)	4.90	ALA337(O)	1IRIA		
1IRIA	CYS404(SG)	3.62	ALA337(CB)	1IRIA		S[ ] ... C[ ]
1IRIA	CYS404(CB)	4.94	LEU339(CG)	1IRIA		
1IRIA	CYS404(CB)	4.04	LEU339(CD1)	1IRIA		C[ ] ... C[ ]
1IRIA	CYS404(SG)	4.82	LEU339(CG)	1IRIA		
1IRIA	CYS404(SG)	3.70	LEU339(CD1)	1IRIA		S[ ] ... C[ ]
1IRIA	CYS404(SG)	4.81	ILE395(CG1)	1IRIA		
1IRIA	CYS404(SG)	4.36	ILE395(CG2)	1IRIA		S[ ] ... C[ ]
1IRIA	CYS404(SG)	4.13	ILE395(CD1)	1IRIA		S[ ] ... C[ ]
1IRIA	CYS404(N)	3.76	ILE402(C)	1IRIA		N[ ] ... C[ ]
1IRIA	CYS404(N)	3.24	ILE402(O)	1IRIA	[D-A-AA]:104.9° [A-D-DD]:115.8° d_planarity:67.8° a_planarity:25.7°, bad d_planarity	N[ ] ... O[ ]
1IRIA	CYS404(CA)	4.76	ILE402(C)	1IRIA		
1IRIA	CYS404(CA)	4.08	ILE402(O)	1IRIA		C[ ] ... O[ ]
1IRIA	CYS404(CB)	4.98	ILE402(C)	1IRIA		
1IRIA	CYS404(CB)	4.00	ILE402(O)	1IRIA		C[ ] ... O[ ]
1IRIA	CYS404(SG)	4.41	ILE402(C)	1IRIA		S[ ] ... C[ ]
1IRIA	CYS404(SG)	3.37	ILE402(O)	1IRIA		S[ ] ... O[ ]
1IRIA	CYS404(SG)	4.71	ILE402(CB)	1IRIA		
1IRIA	CYS404(SG)	4.12	ILE402(CG2)	1IRIA		S[ ] ... C[ ]
1IRIA	CYS404(N)	3.50	PRO403(N)	1IRIA		N[ ] ... N[ ]
1IRIA	CYS404(N)	2.42	PRO403(CA)	1IRIA		N[ ] ... C[ ]
1IRIA	CYS404(N)	2.26	PRO403(O)	1IRIA	[D-A-AA]:29.3° [A-D-DD]:95.9° d_planarity:2.1° a_planarity:0.9°, bad a_angle(sp2)	N[ ] ... O[ ]
1IRIA	CYS404(N)	3.41	PRO403(CB)	1IRIA		N[ ] ... C[ ]
1IRIA	CYS404(N)	4.71	PRO403(CG)	1IRIA		
1IRIA	CYS404(N)	4.67	PRO403(CD)	1IRIA		
1IRIA	CYS404(CA)	4.71	PRO403(N)	1IRIA		
1IRIA	CYS404(CA)	3.80	PRO403(CA)	1IRIA		C[ ] ... C[ ]
1IRIA	CYS404(CA)	2.43	PRO403(C)	1IRIA		C[ ] ... C[ ]
1IRIA	CYS404(CA)	2.80	PRO403(O)	1IRIA		C[ ] ... O[ ]
1IRIA	CYS404(CA)	4.72	PRO403(CB)	1IRIA		
1IRIA	CYS404(C)	4.78	PRO403(CA)	1IRIA		
1IRIA	CYS404(C)	3.58	PRO403(C)	1IRIA		C[ ] ... C[ ]
1IRIA	CYS404(C)	4.03	PRO403(O)	1IRIA		C[ ] ... O[ ]
1IRIA	CYS404(O)	4.10	PRO403(C)	1IRIA		O[ ] ... C[ ]
1IRIA	CYS404(O)	4.82	PRO403(O)	1IRIA		
1IRIA	CYS404(CB)	4.62	PRO403(CA)	1IRIA		
1IRIA	CYS404(CB)	3.43	PRO403(C)	1IRIA		C[ ] ... C[ ]
1IRIA	CYS404(CB)	3.82	PRO403(O)	1IRIA		C[ ] ... O[ ]
1IRIA	CYS404(SG)	4.96	PRO403(CA)	1IRIA		
1IRIA	CYS404(SG)	3.91	PRO403(C)	1IRIA		S[ ] ... C[ ]
1IRIA	CYS404(SG)	4.02	PRO403(O)	1IRIA		S[ ] ... O[ ]
1IRIA	CYS404(N)	3.54	ASP405(N)	1IRIA		N[ ] ... N[ ]
1IRIA	CYS404(N)	4.85	ASP405(CA)	1IRIA		
1IRIA	CYS404(CA)	2.43	ASP405(N)	1IRIA		C[ ] ... N[ ]
1IRIA	CYS404(CA)	3.81	ASP405(CA)	1IRIA		C[ ] ... C[ ]

1IRIA	CYS404(CA)	4.54	ASP405(C)	1IRIA		
1IRIA	CYS404(CA)	4.96	ASP405(O)	1IRIA		
1IRIA	CYS404(CA)	4.83	ASP405(CB)	1IRIA		
1IRIA	CYS404(CA)	4.70	ASP405(OD1)	1IRIA		
1IRIA	CYS404(C)	2.47	ASP405(CA)	1IRIA		C[ ] ... C[ ]
1IRIA	CYS404(C)	3.37	ASP405(C)	1IRIA		C[ ] ... C[ ]
1IRIA	CYS404(C)	4.07	ASP405(O)	1IRIA		C[ ] ... O[ ]
1IRIA	CYS404(C)	3.63	ASP405(CB)	1IRIA		C[ ] ... C[ ]
1IRIA	CYS404(C)	3.98	ASP405(CG)	1IRIA		C[ ] ... C[ ]
1IRIA	CYS404(C)	3.51	ASP405(OD1)	1IRIA		C[ ] ... O[-]
1IRIA	CYS404(O)	2.26	ASP405(N)	1IRIA	[D-A-AA]:29.3° [A-D-DD]:97.1° d_planarity:3.4° a_planarity:4.1°, bad a_angle(sp2)	O[ ] ... N[ ]
1IRIA	CYS404(O)	2.84	ASP405(CA)	1IRIA		O[ ] ... C[ ]
1IRIA	CYS404(O)	3.71	ASP405(C)	1IRIA		O[ ] ... C[ ]
1IRIA	CYS404(O)	4.66	ASP405(O)	1IRIA		
1IRIA	CYS404(O)	4.12	ASP405(CB)	1IRIA		O[ ] ... C[ ]
1IRIA	CYS404(O)	4.32	ASP405(CG)	1IRIA		O[ ] ... C[ ]
1IRIA	CYS404(O)	3.60	ASP405(OD1)	1IRIA		O[ ] ... O[-]
1IRIA	CYS404(CB)	3.29	ASP405(N)	1IRIA		C[ ] ... N[ ]
1IRIA	CYS404(CB)	4.47	ASP405(CA)	1IRIA		C[ ] ... C[ ]
1IRIA	CYS404(CB)	4.74	ASP405(C)	1IRIA		
1IRIA	CYS404(SG)	4.84	ASP405(N)	1IRIA	[D-A-AA]:24.2° [A-D-DD]:144.3° d_planarity:68.2°, maximum distance exceeded, bad a_angle(sp3), bad d_planarity	
1IRIA	CYS404(C)	3.93	PHE406(N)	1IRIA		C[ ] ... N[ ]
1IRIA	CYS404(C)	4.53	PHE406(CD1)	1IRIA		
1IRIA	CYS404(C)	4.95	PHE406(CE1)	1IRIA		
1IRIA	CYS404(O)	3.89	PHE406(N)	1IRIA	[D-A-AA]:82.7° [A-D-DD]:150.4° d_planarity:-65.0° a_planarity:40.8°, maximum distance exceeded, bad a_angle(sp2), bad d_planarity	O[ ] ... N[ ]
1IRIA	CYS404(O)	4.35	PHE406(CD1)	1IRIA		O[ ] ... C[ ]
1IRIA	CYS404(O)	4.88	PHE406(CE1)	1IRIA		
1IRIA	CYS404(CB)	4.11	PHE406(CD1)	1IRIA		C[ ] ... C[ ]
1IRIA	CYS404(CB)	3.82	PHE406(CE1)	1IRIA		C[ ] ... C[ ]
1IRIA	CYS404(CB)	4.96	PHE406(CZ)	1IRIA		
1IRIA	CYS404(SG)	4.39	PHE406(CE1)	1IRIA		S[ ] ... C[ ]
1IRIA	CYS404(N)	4.71	PRO473(C)	1IRIA		
1IRIA	CYS404(N)	3.71	PRO473(O)	1IRIA	[D-A-AA]:138.7° [A-D-DD]:122.7° d_planarity:-30.3° a_planarity:32.1°, maximum distance exceeded	N[ ] ... O[ ]
1IRIA	CYS404(CA)	4.65	PRO473(O)	1IRIA		
1IRIA	CYS404(O)	4.92	PRO473(C)	1IRIA		
1IRIA	CYS404(O)	4.49	PRO473(O)	1IRIA		O[ ] ... O[ ]
1IRIA	CYS404(CB)	4.54	PRO473(O)	1IRIA		
1IRIA	CYS404(N)	4.39	THR474(CA)	1IRIA		N[ ] ... C[ ]
1IRIA	CYS404(N)	4.92	THR474(CG2)	1IRIA		
1IRIA	CYS404(CA)	4.73	THR474(CA)	1IRIA		
1IRIA	CYS404(CA)	4.89	THR474(CG2)	1IRIA		
1IRIA	CYS404(C)	4.27	THR474(CA)	1IRIA		C[ ] ... C[ ]
1IRIA	CYS404(C)	4.64	THR474(C)	1IRIA		
1IRIA	CYS404(C)	4.55	THR474(CB)	1IRIA		
1IRIA	CYS404(C)	4.83	THR474(CG2)	1IRIA		

1IRIA	CYS404(O)	4.47	THR474(N)	1IRIA	[D-A-AA]:141.1° [A-D-DD]:21.7° d_planarity:25.8° a_planarity:-5.0°, maximum distance exceeded, bad d_angle(sp2)	O[ ] ... N[ ]
1IRIA	CYS404(O)	3.17	THR474(CA)	1IRIA		O[ ] ... C[ ]
1IRIA	CYS404(O)	3.43	THR474(C)	1IRIA		O[ ] ... C[ ]
1IRIA	CYS404(O)	4.63	THR474(O)	1IRIA		
1IRIA	CYS404(O)	3.53	THR474(CB)	1IRIA		O[ ] ... C[ ]
1IRIA	CYS404(O)	4.85	THR474(OG1)	1IRIA	[D-A-AA]:148.5° [A-D-DD]:19.1° a_planarity:-54.9°, maximum distance exceeded, bad d_angle(sp3)	
1IRIA	CYS404(O)	4.08	THR474(CG2)	1IRIA		O[ ] ... C[ ]
1IRIA	CYS404(CB)	4.68	THR474(CA)	1IRIA		
1IRIA	CYS404(CB)	4.57	THR474(CB)	1IRIA		
1IRIA	CYS404(CB)	4.04	THR474(CG2)	1IRIA		C[ ] ... C[ ]
1IRIA	CYS404(SG)	4.91	THR474(CG2)	1IRIA		
1IRIA	CYS404(C)	3.93	ASN475(N)	1IRIA		C[ ] ... N[ ]
1IRIA	CYS404(C)	4.88	ASN475(CA)	1IRIA		
1IRIA	CYS404(C)	4.36	ASN475(O)	1IRIA		C[ ] ... O[ ]
1IRIA	CYS404(C)	4.87	ASN475(CB)	1IRIA		
1IRIA	CYS404(O)	2.74	ASN475(N)	1IRIA	**H-bond** [D-A-AA]:163.1° [A-D-DD]:128.2° d_planarity:-1.0° a_planarity:31.6°	O[ ] ... N[ ]
1IRIA	CYS404(O)	3.81	ASN475(CA)	1IRIA		O[ ] ... C[ ]
1IRIA	CYS404(O)	4.13	ASN475(C)	1IRIA		O[ ] ... C[ ]
1IRIA	CYS404(O)	3.54	ASN475(O)	1IRIA		O[ ] ... O[ ]
1IRIA	CYS404(O)	4.05	ASN475(CB)	1IRIA		O[ ] ... C[ ]
1IRIA	CYS404(O)	4.65	ASN475(CG)	1IRIA		
1IRIA	CYS404(O)	4.65	ASN475(OD1)	1IRIA		

chain1	res1/atm1	distance	res2/atm2	chain2	H-bonding	Charge interaction
2DFDA	CYS71(C)	4.88	ALA10(CB)	2DFDA		
2DFDA	CYS71(O)	4.09	ALA10(CB)	2DFDA		O[ ] ... C[ ]
2DFDA	CYS71(SG)	4.99	ALA10(CA)	2DFDA		
2DFDA	CYS71(SG)	4.19	ALA10(CB)	2DFDA		S[ ] ... C[ ]
2DFDA	CYS71(SG)	4.22	LEU12(CD2)	2DFDA		S[ ] ... C[ ]
2DFDA	CYS71(O)	4.51	ARG34(NH1)	2DFDA	[D-A-AA]:132.8° [A-D-DD]:118.5° d_planarity:-66.0° a_planarity:71.3°, maximum distance exceeded, bad d_planarity	
2DFDA	CYS71(CA)	4.28	THR36(CB)	2DFDA		C[ ] ... C[ ]
2DFDA	CYS71(CA)	4.31	THR36(OG1)	2DFDA		C[ ] ... O[ ]
2DFDA	CYS71(CA)	4.76	THR36(CG2)	2DFDA		
2DFDA	CYS71(C)	4.25	THR36(CB)	2DFDA		C[ ] ... C[ ]
2DFDA	CYS71(C)	3.79	THR36(OG1)	2DFDA		C[ ] ... O[ ]
2DFDA	CYS71(C)	4.73	THR36(CG2)	2DFDA		
2DFDA	CYS71(O)	4.86	THR36(CA)	2DFDA		
2DFDA	CYS71(O)	3.41	THR36(CB)	2DFDA		O[ ] ... C[ ]
2DFDA	CYS71(O)	2.70	THR36(OG1)	2DFDA	**H-bond** [D-A-AA]:144.4° [A-D-DD]:106.8° a_planarity:-25.2°	O[ ] ... O[ ]
2DFDA	CYS71(O)	3.90	THR36(CG2)	2DFDA		O[ ] ... C[ ]
2DFDA	CYS71(CB)	4.55	THR36(CB)	2DFDA		

2DFDA	CYS71(CB)	4.61	THR36(OG1)	2DFDA		
2DFDA	CYS71(SG)	4.29	THR36(CA)	2DFDA		S[ ] ... C[ ]
2DFDA	CYS71(SG)	3.76	THR36(C)	2DFDA		S[ ] ... C[ ]
2DFDA	CYS71(SG)	3.79	THR36(O)	2DFDA		S[ ] ... O[ ]
2DFDA	CYS71(SG)	3.64	THR36(CB)	2DFDA		S[ ] ... C[ ]
2DFDA	CYS71(SG)	3.58	THR36(OG1)	2DFDA	**H-bond** [D-A-AA]:113.3° [A-D-DD]:80.7°	S[ ] ... O[ ]
2DFDA	CYS71(SG)	4.91	THR36(CG2)	2DFDA		
2DFDA	CYS71(CB)	5.00	LEU37(O)	2DFDA		
2DFDA	CYS71(SG)	4.03	LEU37(N)	2DFDA	[D-A-AA]:134.2° [A-D-DD]:96.0° d_planarity:68.6°, maximum distance exceeded, bad d_planarity	S[ ] ... N[ ]
2DFDA	CYS71(SG)	4.43	LEU37(CA)	2DFDA		S[ ] ... C[ ]
2DFDA	CYS71(SG)	3.96	LEU37(C)	2DFDA		S[ ] ... C[ ]
2DFDA	CYS71(SG)	4.14	LEU37(O)	2DFDA		S[ ] ... O[ ]
2DFDA	CYS71(CB)	4.35	TYR38(CB)	2DFDA		C[ ] ... C[ ]
2DFDA	CYS71(CB)	4.84	TYR38(CG)	2DFDA		
2DFDA	CYS71(CB)	4.67	TYR38(CD1)	2DFDA		
2DFDA	CYS71(SG)	4.07	TYR38(N)	2DFDA	[D-A-AA]:125.3° [A-D-DD]:90.7° d_planarity:73.5°, maximum distance exceeded, bad d_planarity	S[ ] ... N[ ]
2DFDA	CYS71(SG)	4.35	TYR38(CA)	2DFDA		S[ ] ... C[ ]
2DFDA	CYS71(SG)	3.67	TYR38(CB)	2DFDA		S[ ] ... C[ ]
2DFDA	CYS71(SG)	4.67	TYR38(CG)	2DFDA		
2DFDA	CYS71(SG)	4.94	TYR38(CD1)	2DFDA		
2DFDA	CYS71(N)	4.99	TYR62(CB)	2DFDA		
2DFDA	CYS71(N)	4.16	TYR62(CG)	2DFDA		N[ ] ... C[ ]
2DFDA	CYS71(N)	3.98	TYR62(CD1)	2DFDA		N[ ] ... C[ ]
2DFDA	CYS71(N)	4.27	TYR62(CD2)	2DFDA		N[ ] ... C[ ]
2DFDA	CYS71(N)	3.88	TYR62(CE1)	2DFDA		N[ ] ... C[ ]
2DFDA	CYS71(N)	4.19	TYR62(CE2)	2DFDA		N[ ] ... C[ ]
2DFDA	CYS71(N)	3.98	TYR62(CZ)	2DFDA		N[ ] ... C[ ]
2DFDA	CYS71(N)	4.56	TYR62(OH)	2DFDA	[D-A-AA]:56.7° [A-D-DD]:78.0° d_planarity:53.1° a_planarity:-82.8°, maximum distance exceeded, bad a_angle(sp2), bad d_angle(sp2)	
2DFDA	CYS71(CA)	4.85	TYR62(CB)	2DFDA		
2DFDA	CYS71(CA)	4.02	TYR62(CG)	2DFDA		C[ ] ... C[ ]
2DFDA	CYS71(CA)	4.28	TYR62(CD1)	2DFDA		C[ ] ... C[ ]
2DFDA	CYS71(CA)	3.70	TYR62(CD2)	2DFDA		C[ ] ... C[ ]
2DFDA	CYS71(CA)	4.21	TYR62(CE1)	2DFDA		C[ ] ... C[ ]
2DFDA	CYS71(CA)	3.63	TYR62(CE2)	2DFDA		C[ ] ... C[ ]
2DFDA	CYS71(CA)	3.88	TYR62(CZ)	2DFDA		C[ ] ... C[ ]
2DFDA	CYS71(CA)	4.49	TYR62(OH)	2DFDA		C[ ] ... O[ ]
2DFDA	CYS71(C)	4.80	TYR62(CE2)	2DFDA		
2DFDA	CYS71(O)	4.94	TYR62(CE2)	2DFDA		
2DFDA	CYS71(CB)	4.00	TYR62(CB)	2DFDA		C[ ] ... C[ ]
2DFDA	CYS71(CB)	3.61	TYR62(CG)	2DFDA		C[ ] ... C[ ]
2DFDA	CYS71(CB)	4.27	TYR62(CD1)	2DFDA		C[ ] ... C[ ]
2DFDA	CYS71(CB)	3.41	TYR62(CD2)	2DFDA		C[ ] ... C[ ]
2DFDA	CYS71(CB)	4.66	TYR62(CE1)	2DFDA		
2DFDA	CYS71(CB)	3.89	TYR62(CE2)	2DFDA		C[ ] ... C[ ]
2DFDA	CYS71(CB)	4.47	TYR62(CZ)	2DFDA		C[ ] ... C[ ]
2DFDA	CYS71(SG)	4.61	TYR62(CB)	2DFDA		

2DFDA	CYS71(SG)	4.37	TYR62(CG)	2DFDA		S[ ] ... C[ ]
2DFDA	CYS71(SG)	3.71	TYR62(CD2)	2DFDA		S[ ] ... C[ ]
2DFDA	CYS71(SG)	4.22	TYR62(CE2)	2DFDA		S[ ] ... C[ ]
2DFDA	CYS71(N)	4.43	GLN67(C)	2DFDA		N[ ] ... C[ ]
2DFDA	CYS71(N)	3.28	GLN67(O)	2DFDA	**H-bond** [D-A-AA]:156.9° [A-D-DD]:122.9° d_planarity:-27.9° a_planarity:15.6°	N[ ] ... O[ ]
2DFDA	CYS71(CA)	4.26	GLN67(O)	2DFDA		C[ ] ... O[ ]
2DFDA	CYS71(CB)	4.95	GLN67(C)	2DFDA		
2DFDA	CYS71(CB)	4.03	GLN67(O)	2DFDA		C[ ] ... O[ ]
2DFDA	CYS71(N)	4.96	LEU68(N)	2DFDA		
2DFDA	CYS71(N)	4.50	LEU68(CA)	2DFDA		N[ ] ... C[ ]
2DFDA	CYS71(N)	3.66	LEU68(C)	2DFDA		N[ ] ... C[ ]
2DFDA	CYS71(N)	2.96	LEU68(O)	2DFDA	**H-bond** [D-A-AA]:114.5° [A-D-DD]:98.8° d_planarity:-38.7° a_planarity:84.3°	N[ ] ... O[ ]
2DFDA	CYS71(CA)	4.45	LEU68(C)	2DFDA		C[ ] ... C[ ]
2DFDA	CYS71(CA)	3.50	LEU68(O)	2DFDA		C[ ] ... O[ ]
2DFDA	CYS71(C)	4.98	LEU68(C)	2DFDA		
2DFDA	CYS71(C)	3.82	LEU68(O)	2DFDA		C[ ] ... O[ ]
2DFDA	CYS71(CB)	4.40	LEU68(CA)	2DFDA		C[ ] ... C[ ]
2DFDA	CYS71(CB)	4.16	LEU68(C)	2DFDA		C[ ] ... C[ ]
2DFDA	CYS71(CB)	3.30	LEU68(O)	2DFDA		C[ ] ... O[ ]
2DFDA	CYS71(CB)	4.55	LEU68(CD1)	2DFDA		
2DFDA	CYS71(SG)	4.78	LEU68(O)	2DFDA		
2DFDA	CYS71(N)	4.31	PRO69(N)	2DFDA		N[ ] ... N[ ]
2DFDA	CYS71(N)	4.31	PRO69(CA)	2DFDA		N[ ] ... C[ ]
2DFDA	CYS71(N)	3.23	PRO69(C)	2DFDA		N[ ] ... C[ ]
2DFDA	CYS71(N)	3.32	PRO69(O)	2DFDA	[D-A-AA]:74.9° [A-D-DD]:121.7° d_planarity:77.7° a_planarity:50.8°, bad a_angle(sp2), bad d_planarity	N[ ] ... O[ ]
2DFDA	CYS71(CA)	4.43	PRO69(C)	2DFDA		C[ ] ... C[ ]
2DFDA	CYS71(CA)	4.28	PRO69(O)	2DFDA		C[ ] ... O[ ]
2DFDA	CYS71(C)	4.60	PRO69(C)	2DFDA		
2DFDA	CYS71(C)	4.05	PRO69(O)	2DFDA		C[ ] ... O[ ]
2DFDA	CYS71(N)	2.81	ASP70(N)	2DFDA		N[ ] ... N[ ]
2DFDA	CYS71(N)	2.46	ASP70(CA)	2DFDA		N[ ] ... C[ ]
2DFDA	CYS71(N)	2.22	ASP70(O)	2DFDA	[D-A-AA]:30.5° [A-D-DD]:93.2° d_planarity:0.1° a_planarity:3.0°, bad a_angle(sp2)	N[ ] ... O[ ]
2DFDA	CYS71(N)	3.33	ASP70(CB)	2DFDA		N[ ] ... C[ ]
2DFDA	CYS71(N)	4.73	ASP70(CG)	2DFDA		
2DFDA	CYS71(CA)	4.23	ASP70(N)	2DFDA		C[ ] ... N[ ]
2DFDA	CYS71(CA)	3.83	ASP70(CA)	2DFDA		C[ ] ... C[ ]
2DFDA	CYS71(CA)	2.43	ASP70(C)	2DFDA		C[ ] ... C[ ]
2DFDA	CYS71(CA)	2.73	ASP70(O)	2DFDA		C[ ] ... O[ ]
2DFDA	CYS71(CA)	4.65	ASP70(CB)	2DFDA		
2DFDA	CYS71(C)	4.79	ASP70(N)	2DFDA		
2DFDA	CYS71(C)	4.46	ASP70(CA)	2DFDA		C[ ] ... C[ ]
2DFDA	CYS71(C)	3.08	ASP70(C)	2DFDA		C[ ] ... C[ ]
2DFDA	CYS71(C)	3.03	ASP70(O)	2DFDA		C[ ] ... O[ ]
2DFDA	CYS71(O)	4.04	ASP70(C)	2DFDA		O[ ] ... C[ ]
2DFDA	CYS71(O)	3.66	ASP70(O)	2DFDA		O[ ] ... O[ ]
2DFDA	CYS71(CB)	4.96	ASP70(N)	2DFDA		

2DFDA	CYS71(CB)	4.89	ASP70(CA)	2DFDA		
2DFDA	CYS71(CB)	3.70	ASP70(C)	2DFDA		C[ ] ... C[ ]
2DFDA	CYS71(CB)	4.22	ASP70(O)	2DFDA		C[ ] ... O[ ]
2DFDA	CYS71(N)	2.77	LEU72(N)	2DFDA		N[ ] ... N[ ]
2DFDA	CYS71(N)	4.21	LEU72(CA)	2DFDA		N[ ] ... C[ ]
2DFDA	CYS71(N)	4.94	LEU72(C)	2DFDA		
2DFDA	CYS71(N)	4.90	LEU72(CB)	2DFDA		
2DFDA	CYS71(CA)	2.44	LEU72(N)	2DFDA		C[ ] ... N[ ]
2DFDA	CYS71(CA)	3.82	LEU72(CA)	2DFDA		C[ ] ... C[ ]
2DFDA	CYS71(CA)	4.72	LEU72(C)	2DFDA		
2DFDA	CYS71(CA)	4.75	LEU72(CB)	2DFDA		
2DFDA	CYS71(CA)	4.87	LEU72(CG)	2DFDA		
2DFDA	CYS71(C)	2.45	LEU72(CA)	2DFDA		C[ ] ... C[ ]
2DFDA	CYS71(C)	3.34	LEU72(C)	2DFDA		C[ ] ... C[ ]
2DFDA	CYS71(C)	4.29	LEU72(O)	2DFDA		C[ ] ... O[ ]
2DFDA	CYS71(C)	3.62	LEU72(CB)	2DFDA		C[ ] ... C[ ]
2DFDA	CYS71(C)	3.99	LEU72(CG)	2DFDA		C[ ] ... C[ ]
2DFDA	CYS71(C)	4.35	LEU72(CD2)	2DFDA		C[ ] ... C[ ]
2DFDA	CYS71(O)	2.26	LEU72(N)	2DFDA	[D-A-AA]:29.9° [A-D-DD]:95.0° d_planarity:4.5° a_planarity:0.7°, bad a_angle(sp2)	O[ ] ... N[ ]
2DFDA	CYS71(O)	2.80	LEU72(CA)	2DFDA		O[ ] ... C[ ]
2DFDA	CYS71(O)	3.46	LEU72(C)	2DFDA		O[ ] ... C[ ]
2DFDA	CYS71(O)	4.10	LEU72(O)	2DFDA		O[ ] ... O[ ]
2DFDA	CYS71(O)	4.17	LEU72(CB)	2DFDA		O[ ] ... C[ ]
2DFDA	CYS71(O)	4.57	LEU72(CG)	2DFDA		
2DFDA	CYS71(O)	4.54	LEU72(CD2)	2DFDA		
2DFDA	CYS71(CB)	3.26	LEU72(N)	2DFDA		C[ ] ... N[ ]
2DFDA	CYS71(CB)	4.50	LEU72(CA)	2DFDA		C[ ] ... C[ ]
2DFDA	CYS71(CB)	4.77	LEU72(CG)	2DFDA		
2DFDA	CYS71(SG)	4.14	LEU72(N)	2DFDA	[D-A-AA]:49.1° [A-D-DD]:119.5° d_planarity:44.3°, maximum distance exceeded, bad a_angle(sp3)	S[ ] ... N[ ]
2DFDA	CYS71(N)	4.61	LYS73(N)	2DFDA		
2DFDA	CYS71(CA)	4.72	LYS73(N)	2DFDA		
2DFDA	CYS71(C)	3.61	LYS73(N)	2DFDA		C[ ] ... N[ ]
2DFDA	CYS71(C)	4.95	LYS73(CA)	2DFDA		
2DFDA	CYS71(C)	4.95	LYS73(O)	2DFDA		
2DFDA	CYS71(O)	3.94	LYS73(N)	2DFDA	[D-A-AA]:65.8° [A-D-DD]:139.7° d_planarity:77.0° a_planarity:38.0°, maximum distance exceeded, bad a_angle(sp2), bad d_planarity	O[ ] ... N[ ]
2DFDA	CYS71(O)	4.63	LYS73(O)	2DFDA		
2DFDA	CYS71(C)	4.33	CYS75(SG)	2DFDA		C[ ] ... S[ ]
2DFDA	CYS71(O)	4.93	CYS75(CB)	2DFDA		
2DFDA	CYS71(O)	3.32	CYS75(SG)	2DFDA		O[ ] ... S[ ]

chain1	res1/atm1	distance	res2/atm2	chain2	H-bonding	Charge interaction
2DFDA	CYS267(C)	4.22	SER262(O)	2DFDA		C[ ] ... O[ ]
2DFDA	CYS267(O)	4.78	SER262(CA)	2DFDA		

2DFDA	CYS267(O)	4.10	SER262(C)	2DFDA		O[ ] ... C[ ]
2DFDA	CYS267(O)	3.22	SER262(O)	2DFDA		O[ ] ... O[ ]
2DFDA	CYS267(O)	4.41	SER262(CB)	2DFDA		O[ ] ... C[ ]
2DFDA	CYS267(O)	4.80	SER262(OG)	2DFDA	[D-A-AA]:153.9° [A-D-DD]:65.4° a_planarity:86.0°, maximum distance exceeded	
2DFDA	CYS267(O)	4.90	GLN263(N)	2DFDA	[D-A-AA]:159.6° [A-D-DD]:84.1° d_planarity:27.2° a_planarity:23.2°, maximum distance exceeded, bad d_angle(sp2)	
2DFDA	CYS267(O)	4.96	GLN263(CA)	2DFDA		
2DFDA	CYS267(N)	4.85	THR265(N)	2DFDA		
2DFDA	CYS267(N)	4.12	THR265(CA)	2DFDA		N[ ] ... C[ ]
2DFDA	CYS267(N)	3.13	THR265(C)	2DFDA		N[ ] ... C[ ]
2DFDA	CYS267(N)	3.24	THR265(O)	2DFDA	[D-A-AA]:73.8° [A-D-DD]:126.4° d_planarity:88.6° a_planarity:57.6°, bad a_angle(sp2), bad d_planarity	N[ ] ... O[ ]
2DFDA	CYS267(N)	3.84	THR265(CB)	2DFDA		N[ ] ... C[ ]
2DFDA	CYS267(N)	2.92	THR265(OG1)	2DFDA	**H-bond** [D-A-AA]:119.6° [A-D-DD]:105.1° d_planarity:-21.4°	N[ ] ... O[ ]
2DFDA	CYS267(CA)	4.39	THR265(C)	2DFDA		C[ ] ... C[ ]
2DFDA	CYS267(CA)	4.28	THR265(O)	2DFDA		C[ ] ... O[ ]
2DFDA	CYS267(CA)	4.81	THR265(CB)	2DFDA		
2DFDA	CYS267(CA)	3.59	THR265(OG1)	2DFDA		C[ ] ... O[ ]
2DFDA	CYS267(C)	4.63	THR265(C)	2DFDA		
2DFDA	CYS267(C)	4.12	THR265(O)	2DFDA		C[ ] ... O[ ]
2DFDA	CYS267(C)	4.13	THR265(OG1)	2DFDA		C[ ] ... O[ ]
2DFDA	CYS267(O)	4.70	THR265(N)	2DFDA	[D-A-AA]:135.6° [A-D-DD]:86.4° d_planarity:-1.6° a_planarity:-17.2°, maximum distance exceeded, bad d_angle(sp2)	
2DFDA	CYS267(O)	4.84	THR265(CA)	2DFDA		
2DFDA	CYS267(O)	4.08	THR265(C)	2DFDA		O[ ] ... C[ ]
2DFDA	CYS267(O)	3.42	THR265(O)	2DFDA		O[ ] ... O[ ]
2DFDA	CYS267(O)	4.87	THR265(CB)	2DFDA		
2DFDA	CYS267(O)	3.70	THR265(OG1)	2DFDA	[D-A-AA]:101.6° [A-D-DD]:138.6° a_planarity:-7.1°, maximum distance exceeded	O[ ] ... O[ ]
2DFDA	CYS267(CB)	4.66	THR265(CB)	2DFDA		
2DFDA	CYS267(CB)	3.31	THR265(OG1)	2DFDA		C[ ] ... O[ ]
2DFDA	CYS267(SG)	4.68	THR265(CB)	2DFDA		
2DFDA	CYS267(SG)	3.61	THR265(OG1)	2DFDA	**H-bond** [D-A-AA]:65.9° [A-D-DD]:131.0°	S[ ] ... O[ ]
2DFDA	CYS267(N)	2.84	GLU266(N)	2DFDA		N[ ] ... N[ ]
2DFDA	CYS267(N)	2.48	GLU266(CA)	2DFDA		N[ ] ... C[ ]
2DFDA	CYS267(N)	2.20	GLU266(O)	2DFDA	[D-A-AA]:31.2° [A-D-DD]:92.8° d_planarity:1.5° a_planarity:1.1°, bad a_angle(sp2)	N[ ] ... O[ ]
2DFDA	CYS267(N)	3.51	GLU266(CB)	2DFDA		N[ ] ... C[ ]
2DFDA	CYS267(N)	3.63	GLU266(CG)	2DFDA		N[ ] ... C[ ]
2DFDA	CYS267(CA)	4.28	GLU266(N)	2DFDA		C[ ] ... N[ ]
2DFDA	CYS267(CA)	3.85	GLU266(CA)	2DFDA		C[ ] ... C[ ]
2DFDA	CYS267(CA)	2.42	GLU266(C)	2DFDA		C[ ] ... C[ ]
2DFDA	CYS267(CA)	2.70	GLU266(O)	2DFDA		C[ ] ... O[ ]
2DFDA	CYS267(CA)	4.79	GLU266(CB)	2DFDA		
2DFDA	CYS267(CA)	4.77	GLU266(CG)	2DFDA		
2DFDA	CYS267(C)	4.86	GLU266(N)	2DFDA		
2DFDA	CYS267(C)	4.43	GLU266(CA)	2DFDA		C[ ] ... C[ ]
2DFDA	CYS267(C)	3.06	GLU266(C)	2DFDA		C[ ] ... C[ ]

2DFDA	CYS267(C)	3.07	GLU266(O)	2DFDA		C[ ] ... O[ ]
2DFDA	CYS267(O)	4.64	GLU266(N)	2DFDA	[D-A-AA]:92.9° [A-D-DD]:75.1° d_planarity:37.5° a_planarity:-35.8°, maximum distance exceeded, bad d_angle(sp2)	
2DFDA	CYS267(O)	4.50	GLU266(CA)	2DFDA		O[ ] ... C[ ]
2DFDA	CYS267(O)	3.42	GLU266(C)	2DFDA		O[ ] ... C[ ]
2DFDA	CYS267(O)	3.66	GLU266(O)	2DFDA		O[ ] ... O[ ]
2DFDA	CYS267(CB)	4.93	GLU266(CA)	2DFDA		
2DFDA	CYS267(CB)	3.71	GLU266(C)	2DFDA		C[ ] ... C[ ]
2DFDA	CYS267(CB)	4.18	GLU266(O)	2DFDA		C[ ] ... O[ ]
2DFDA	CYS267(SG)	4.26	GLU266(C)	2DFDA		S[ ] ... C[ ]
2DFDA	CYS267(SG)	4.88	GLU266(O)	2DFDA		
2DFDA	CYS267(SG)	4.95	GLU266(CG)	2DFDA		
2DFDA	CYS267(N)	3.52	THR268(N)	2DFDA		N[ ] ... N[ ]
2DFDA	CYS267(N)	4.71	THR268(CA)	2DFDA		
2DFDA	CYS267(CA)	2.42	THR268(N)	2DFDA		C[ ] ... N[ ]
2DFDA	CYS267(CA)	3.78	THR268(CA)	2DFDA		C[ ] ... C[ ]
2DFDA	CYS267(CA)	4.52	THR268(C)	2DFDA		
2DFDA	CYS267(CA)	4.82	THR268(CB)	2DFDA		
2DFDA	CYS267(CA)	4.97	THR268(OG1)	2DFDA		
2DFDA	CYS267(CA)	4.95	THR268(CG2)	2DFDA		
2DFDA	CYS267(C)	2.40	THR268(CA)	2DFDA		C[ ] ... C[ ]
2DFDA	CYS267(C)	3.14	THR268(C)	2DFDA		C[ ] ... C[ ]
2DFDA	CYS267(C)	4.15	THR268(O)	2DFDA		C[ ] ... O[ ]
2DFDA	CYS267(C)	3.66	THR268(CB)	2DFDA		C[ ] ... C[ ]
2DFDA	CYS267(C)	4.08	THR268(OG1)	2DFDA		C[ ] ... O[ ]
2DFDA	CYS267(C)	4.00	THR268(CG2)	2DFDA		C[ ] ... C[ ]
2DFDA	CYS267(O)	2.24	THR268(N)	2DFDA	[D-A-AA]:29.7° [A-D-DD]:91.8° d_planarity:3.1° a_planarity:1.3°, bad a_angle(sp2)	O[ ] ... N[ ]
2DFDA	CYS267(O)	2.71	THR268(CA)	2DFDA		O[ ] ... C[ ]
2DFDA	CYS267(O)	3.16	THR268(C)	2DFDA		O[ ] ... C[ ]
2DFDA	CYS267(O)	3.90	THR268(O)	2DFDA		O[ ] ... O[ ]
2DFDA	CYS267(O)	4.15	THR268(CB)	2DFDA		O[ ] ... C[ ]
2DFDA	CYS267(O)	4.84	THR268(OG1)	2DFDA	[D-A-AA]:45.6° [A-D-DD]:53.0° a_planarity:1.3°, maximum distance exceeded, bad a_angle(sp2), bad d_angle(sp3)	
2DFDA	CYS267(O)	4.55	THR268(CG2)	2DFDA		
2DFDA	CYS267(CB)	3.30	THR268(N)	2DFDA		C[ ] ... N[ ]
2DFDA	CYS267(CB)	4.60	THR268(CA)	2DFDA		
2DFDA	CYS267(CB)	4.89	THR268(C)	2DFDA		
2DFDA	CYS267(SG)	4.90	THR268(N)	2DFDA	[D-A-AA]:21.1° [A-D-DD]:157.4° d_planarity:41.8°, maximum distance exceeded, bad a_angle(sp3)	
2DFDA	CYS267(CA)	4.29	TYR269(N)	2DFDA		C[ ] ... N[ ]
2DFDA	CYS267(CA)	4.25	TYR269(O)	2DFDA		C[ ] ... O[ ]
2DFDA	CYS267(C)	3.19	TYR269(N)	2DFDA		C[ ] ... N[ ]
2DFDA	CYS267(C)	4.39	TYR269(CA)	2DFDA		C[ ] ... C[ ]
2DFDA	CYS267(C)	4.41	TYR269(C)	2DFDA		C[ ] ... C[ ]
2DFDA	CYS267(C)	3.94	TYR269(O)	2DFDA		C[ ] ... O[ ]
2DFDA	CYS267(O)	3.37	TYR269(N)	2DFDA	[D-A-AA]:70.7° [A-D-DD]:123.5° d_planarity:86.1° a_planarity:46.5°, bad a_angle(sp2), bad d_planarity	O[ ] ... N[ ]
2DFDA	CYS267(O)	4.36	TYR269(CA)	2DFDA		O[ ] ... C[ ]
2DFDA	CYS267(O)	4.31	TYR269(C)	2DFDA		O[ ] ... C[ ]

2DFDA	CYS267(O)	4.08	TYR269(O)	2DFDA		O[ ] ... O[ ]
2DFDA	CYS267(CB)	4.26	TYR269(N)	2DFDA		C[ ] ... N[ ]
2DFDA	CYS267(CB)	4.98	TYR269(CA)	2DFDA		
2DFDA	CYS267(CB)	4.29	TYR269(C)	2DFDA		C[ ] ... C[ ]
2DFDA	CYS267(CB)	3.26	TYR269(O)	2DFDA		C[ ] ... O[ ]
2DFDA	CYS267(SG)	4.51	TYR269(O)	2DFDA		
2DFDA	CYS267(O)	4.59	PHE270(CB)	2DFDA		
2DFDA	CYS267(CB)	4.45	PHE270(CB)	2DFDA		C[ ] ... C[ ]
2DFDA	CYS267(CB)	4.72	PHE270(CG)	2DFDA		
2DFDA	CYS267(CB)	4.09	PHE270(CD2)	2DFDA		C[ ] ... C[ ]
2DFDA	CYS267(SG)	4.16	PHE270(CD2)	2DFDA		S[ ] ... C[ ]
2DFDA	CYS267(SG)	4.61	PHE270(CE2)	2DFDA		
2DFDA	CYS267(SG)	4.26	ILE302(CA)	2DFDA		S[ ] ... C[ ]
2DFDA	CYS267(SG)	4.63	ILE302(C)	2DFDA		
2DFDA	CYS267(SG)	4.09	ILE302(O)	2DFDA		S[ ] ... O[ ]
2DFDA	CYS267(SG)	4.26	ILE302(CB)	2DFDA		S[ ] ... C[ ]
2DFDA	CYS267(SG)	3.79	ILE302(CG1)	2DFDA		S[ ] ... C[ ]
2DFDA	CYS267(SG)	4.14	ILE302(CG2)	2DFDA		S[ ] ... C[ ]
2DFDA	CYS267(SG)	4.62	ILE302(CD1)	2DFDA		
2DFDA	CYS267(CB)	4.80	LEU305(CB)	2DFDA		
2DFDA	CYS267(CB)	4.88	LEU305(CD1)	2DFDA		
2DFDA	CYS267(SG)	3.95	LEU305(CB)	2DFDA		S[ ] ... C[ ]
2DFDA	CYS267(SG)	4.72	LEU305(CG)	2DFDA		
2DFDA	CYS267(SG)	4.16	LEU305(CD1)	2DFDA		S[ ] ... C[ ]
2DFDA	CYS267(CA)	4.74	LYS306(CG)	2DFDA		
2DFDA	CYS267(CB)	4.94	LYS306(CG)	2DFDA		
2DFDA	CYS267(SG)	4.65	LYS306(N)	2DFDA	[D-A-AA]:105.3° [A-D-DD]:107.0° d_planarity:-68.9°, maximum distance exceeded, bad d_planarity	
2DFDA	CYS267(SG)	4.23	LYS306(CG)	2DFDA	positive	S[ ] ... C[ ]
2DFDA	CYS267(CA)	4.53	ILE309(CD1)	2DFDA		
2DFDA	CYS267(C)	4.54	ILE309(CD1)	2DFDA		
2DFDA	CYS267(CB)	4.15	ILE309(CD1)	2DFDA		C[ ] ... C[ ]

chain1	res1/atm1	Distance	res2/atm2	chain2	H-bonding	Charge interaction
2ZKRn	CYS75(O)	5.00	CYS61(O)	2ZKRn		
2ZKRn	CYS75(C)	4.58	GLN62(CA)	2ZKRn		
2ZKRn	CYS75(C)	4.74	GLN62(C)	2ZKRn		
2ZKRn	CYS75(C)	4.92	GLN62(CG)	2ZKRn		
2ZKRn	CYS75(O)	4.87	GLN62(N)	2ZKRn	[D-A-AA]:125.2° [A-D-DD]:26.9° d_planarity:53.0° a_planarity:24.3°, maximum distance exceeded, bad d_angle(sp2)	
2ZKRn	CYS75(O)	3.63	GLN62(CA)	2ZKRn		O[ ] ... C[ ]
2ZKRn	CYS75(O)	3.65	GLN62(C)	2ZKRn		O[ ] ... C[ ]
2ZKRn	CYS75(O)	4.84	GLN62(O)	2ZKRn		
2ZKRn	CYS75(O)	4.29	GLN62(CB)	2ZKRn		O[ ] ... C[ ]
2ZKRn	CYS75(O)	3.93	GLN62(CG)	2ZKRn		O[ ] ... C[ ]
2ZKRn	CYS75(N)	4.86	ILE63(N)	2ZKRn		

2ZKRn	CYS75(N)	4.88	ILE63(C)	2ZKRn		
2ZKRn	CYS75(N)	3.94	ILE63(O)	2ZKRn	[D-A-AA]:134.8° [A-D-DD]:120.4° d_planarity:-12.7° a_planarity:-3.1°, maximum distance exceeded	N[ ] ... O[ ]
2ZKRn	CYS75(N)	4.69	ILE63(CB)	2ZKRn		
2ZKRn	CYS75(N)	3.31	ILE63(CG2)	2ZKRn		N[ ] ... C[ ]
2ZKRn	CYS75(CA)	4.91	ILE63(N)	2ZKRn		
2ZKRn	CYS75(CA)	4.84	ILE63(O)	2ZKRn		
2ZKRn	CYS75(CA)	5.00	ILE63(CB)	2ZKRn		
2ZKRn	CYS75(CA)	3.55	ILE63(CG2)	2ZKRn		C[ ] ... C[ ]
2ZKRn	CYS75(C)	3.91	ILE63(N)	2ZKRn		C[ ] ... N[ ]
2ZKRn	CYS75(C)	4.75	ILE63(CA)	2ZKRn		
2ZKRn	CYS75(C)	4.56	ILE63(O)	2ZKRn		
2ZKRn	CYS75(C)	4.68	ILE63(CB)	2ZKRn		
2ZKRn	CYS75(C)	3.47	ILE63(CG2)	2ZKRn		C[ ] ... C[ ]
2ZKRn	CYS75(O)	2.81	ILE63(N)	2ZKRn	**H-bond** [D-A-AA]:148.3° [A-D-DD]:117.6° d_planarity:-11.9° a_planarity:84.6°	O[ ] ... N[ ]
2ZKRn	CYS75(O)	3.72	ILE63(CA)	2ZKRn		O[ ] ... C[ ]
2ZKRn	CYS75(O)	4.02	ILE63(C)	2ZKRn		O[ ] ... C[ ]
2ZKRn	CYS75(O)	3.58	ILE63(O)	2ZKRn		O[ ] ... O[ ]
2ZKRn	CYS75(O)	3.95	ILE63(CB)	2ZKRn		O[ ] ... C[ ]
2ZKRn	CYS75(O)	3.01	ILE63(CG2)	2ZKRn		O[ ] ... C[ ]
2ZKRn	CYS75(CB)	4.86	ILE63(CB)	2ZKRn		
2ZKRn	CYS75(CB)	3.39	ILE63(CG2)	2ZKRn		C[ ] ... C[ ]
2ZKRn	CYS75(SG)	4.66	ILE63(CG2)	2ZKRn		
2ZKRn	CYS75(N)	4.43	ILE73(CA)	2ZKRn		N[ ] ... C[ ]
2ZKRn	CYS75(N)	3.39	ILE73(C)	2ZKRn		N[ ] ... C[ ]
2ZKRn	CYS75(N)	3.61	ILE73(O)	2ZKRn	[D-A-AA]:69.7° [A-D-DD]:124.4° d_planarity:67.8° a_planarity:50.0°, maximum distance exceeded, bad a_angle(sp2), bad d_planarity	N[ ] ... O[ ]
2ZKRn	CYS75(N)	4.81	ILE73(CB)	2ZKRn		
2ZKRn	CYS75(N)	3.92	ILE73(CG2)	2ZKRn		N[ ] ... C[ ]
2ZKRn	CYS75(CA)	4.55	ILE73(C)	2ZKRn		
2ZKRn	CYS75(CA)	4.59	ILE73(O)	2ZKRn		
2ZKRn	CYS75(CA)	4.27	ILE73(CG2)	2ZKRn		C[ ] ... C[ ]
2ZKRn	CYS75(C)	4.85	ILE73(C)	2ZKRn		
2ZKRn	CYS75(C)	3.62	ILE73(CG2)	2ZKRn		C[ ] ... C[ ]
2ZKRn	CYS75(O)	4.69	ILE73(CA)	2ZKRn		
2ZKRn	CYS75(O)	4.52	ILE73(C)	2ZKRn		
2ZKRn	CYS75(O)	4.95	ILE73(O)	2ZKRn		
2ZKRn	CYS75(O)	4.45	ILE73(CB)	2ZKRn		O[ ] ... C[ ]
2ZKRn	CYS75(O)	3.04	ILE73(CG2)	2ZKRn		O[ ] ... C[ ]
2ZKRn	CYS75(N)	2.85	VAL74(N)	2ZKRn		N[ ] ... N[ ]
2ZKRn	CYS75(N)	2.43	VAL74(CA)	2ZKRn		N[ ] ... C[ ]
2ZKRn	CYS75(N)	2.23	VAL74(O)	2ZKRn	[D-A-AA]:30.4° [A-D-DD]:98.2° d_planarity:0.2° a_planarity:1.8°, bad a_angle(sp2)	N[ ] ... O[ ]
2ZKRn	CYS75(N)	3.17	VAL74(CB)	2ZKRn		N[ ] ... C[ ]
2ZKRn	CYS75(N)	3.28	VAL74(CG1)	2ZKRn		N[ ] ... C[ ]
2ZKRn	CYS75(N)	3.56	VAL74(CG2)	2ZKRn		N[ ] ... C[ ]
2ZKRn	CYS75(CA)	4.23	VAL74(N)	2ZKRn		C[ ] ... N[ ]
2ZKRn	CYS75(CA)	3.83	VAL74(CA)	2ZKRn		C[ ] ... C[ ]

2ZKRn	CYS75(CA)	2.48	VAL74(C)	2ZKRn		C[ ] ... C[ ]
2ZKRn	CYS75(CA)	2.83	VAL74(O)	2ZKRn		C[ ] ... O[ ]
2ZKRn	CYS75(CA)	4.52	VAL74(CB)	2ZKRn		
2ZKRn	CYS75(CA)	4.50	VAL74(CG1)	2ZKRn		
2ZKRn	CYS75(CA)	4.62	VAL74(CG2)	2ZKRn		
2ZKRn	CYS75(C)	4.74	VAL74(N)	2ZKRn		
2ZKRn	CYS75(C)	4.79	VAL74(CA)	2ZKRn		
2ZKRn	CYS75(C)	3.67	VAL74(C)	2ZKRn		C[ ] ... C[ ]
2ZKRn	CYS75(C)	4.17	VAL74(O)	2ZKRn		C[ ] ... O[ ]
2ZKRn	CYS75(O)	4.42	VAL74(N)	2ZKRn	[D-A-AA]:97.8° [A-D-DD]:96.1° d_planarity:88.5° a_planarity:-26.2°, maximum distance exceeded, bad d_planarity	O[ ] ... N[ ]
2ZKRn	CYS75(O)	4.80	VAL74(CA)	2ZKRn		
2ZKRn	CYS75(O)	4.02	VAL74(C)	2ZKRn		O[ ] ... C[ ]
2ZKRn	CYS75(O)	4.79	VAL74(O)	2ZKRn		
2ZKRn	CYS75(CB)	4.62	VAL74(CA)	2ZKRn		
2ZKRn	CYS75(CB)	3.34	VAL74(C)	2ZKRn		C[ ] ... C[ ]
2ZKRn	CYS75(CB)	3.58	VAL74(O)	2ZKRn		C[ ] ... O[ ]
2ZKRn	CYS75(CB)	4.86	VAL74(CB)	2ZKRn		
2ZKRn	CYS75(CB)	4.64	VAL74(CG1)	2ZKRn		
2ZKRn	CYS75(CB)	4.59	VAL74(CG2)	2ZKRn		
2ZKRn	CYS75(SG)	4.67	VAL74(CA)	2ZKRn		
2ZKRn	CYS75(SG)	3.42	VAL74(C)	2ZKRn		S[ ] ... C[ ]
2ZKRn	CYS75(SG)	3.22	VAL74(O)	2ZKRn		S[ ] ... O[ ]
2ZKRn	CYS75(SG)	4.73	VAL74(CB)	2ZKRn		
2ZKRn	CYS75(SG)	4.88	VAL74(CG1)	2ZKRn		
2ZKRn	CYS75(SG)	3.93	VAL74(CG2)	2ZKRn		S[ ] ... C[ ]
2ZKRn	CYS75(N)	3.65	ALA76(N)	2ZKRn		N[ ] ... N[ ]
2ZKRn	CYS75(N)	4.90	ALA76(CA)	2ZKRn		
2ZKRn	CYS75(CA)	2.43	ALA76(N)	2ZKRn		C[ ] ... N[ ]
2ZKRn	CYS75(CA)	3.82	ALA76(CA)	2ZKRn		C[ ] ... C[ ]
2ZKRn	CYS75(CA)	4.84	ALA76(C)	2ZKRn		
2ZKRn	CYS75(CA)	4.81	ALA76(O)	2ZKRn		
2ZKRn	CYS75(CA)	4.45	ALA76(CB)	2ZKRn		C[ ] ... C[ ]
2ZKRn	CYS75(C)	2.45	ALA76(CA)	2ZKRn		C[ ] ... C[ ]
2ZKRn	CYS75(C)	3.70	ALA76(C)	2ZKRn		C[ ] ... C[ ]
2ZKRn	CYS75(C)	3.97	ALA76(O)	2ZKRn		C[ ] ... O[ ]
2ZKRn	CYS75(C)	3.08	ALA76(CB)	2ZKRn		C[ ] ... C[ ]
2ZKRn	CYS75(O)	2.22	ALA76(N)	2ZKRn	[D-A-AA]:31.0° [A-D-DD]:94.5° d_planarity:1.3° a_planarity:2.3°, bad a_angle(sp2)	O[ ] ... N[ ]
2ZKRn	CYS75(O)	2.75	ALA76(CA)	2ZKRn		O[ ] ... C[ ]
2ZKRn	CYS75(O)	4.20	ALA76(C)	2ZKRn		O[ ] ... C[ ]
2ZKRn	CYS75(O)	4.75	ALA76(O)	2ZKRn		
2ZKRn	CYS75(O)	3.10	ALA76(CB)	2ZKRn		O[ ] ... C[ ]
2ZKRn	CYS75(CB)	3.06	ALA76(N)	2ZKRn		C[ ] ... N[ ]
2ZKRn	CYS75(CB)	4.40	ALA76(CA)	2ZKRn		C[ ] ... C[ ]
2ZKRn	CYS75(SG)	4.59	ALA76(N)	2ZKRn	[D-A-AA]:24.1° [A-D-DD]:163.2° d_planarity:-85.2°, maximum distance exceeded, bad a_angle(sp3), bad d_planarity	
2ZKRn	CYS75(C)	4.76	ARG77(N)	2ZKRn		
2ZKRn	CYS75(C)	4.73	LEU104(CD1)	2ZKRn		

2ZKRn	CYS75(O)	4.35	LEU104(CD1)	2ZKRn		O[ ] ... C[ ]
2ZKRn	CYS75(CB)	4.17	LEU104(O)	2ZKRn		C[ ] ... O[ ]
2ZKRn	CYS75(SG)	4.96	LEU104(O)	2ZKRn		
2ZKRn	CYS75(CA)	4.82	ARG107(CG)	2ZKRn		
2ZKRn	CYS75(CA)	4.58	ARG107(CD)	2ZKRn		
2ZKRn	CYS75(C)	4.71	ARG107(CD)	2ZKRn		
2ZKRn	CYS75(CB)	4.12	ARG107(C)	2ZKRn		C[ ] ... C[ ]
2ZKRn	CYS75(CB)	4.19	ARG107(O)	2ZKRn		C[ ] ... O[ ]
2ZKRn	CYS75(CB)	4.54	ARG107(CB)	2ZKRn		
2ZKRn	CYS75(CB)	3.76	ARG107(CG)	2ZKRn		C[ ] ... C[ ]
2ZKRn	CYS75(CB)	3.97	ARG107(CD)	2ZKRn		C[ ] ... C[ ]
2ZKRn	CYS75(SG)	4.41	ARG107(CA)	2ZKRn		S[ ] ... C[ ]
2ZKRn	CYS75(SG)	3.38	ARG107(C)	2ZKRn		S[ ] ... C[ ]
2ZKRn	CYS75(SG)	2.97	ARG107(O)	2ZKRn		S[ ] ... O[ ]
2ZKRn	CYS75(SG)	4.11	ARG107(CB)	2ZKRn		S[ ] ... C[ ]
2ZKRn	CYS75(SG)	3.97	ARG107(CG)	2ZKRn	Positive	S[ ] ... C[ ]
2ZKRn	CYS75(SG)	4.39	ARG107(CD)	2ZKRn		S[ ] ... C[ ]
2ZKRn	CYS75(CB)	3.78	LEU108(N)	2ZKRn		C[ ] ... N[ ]
2ZKRn	CYS75(CB)	3.78	LEU108(CA)	2ZKRn		C[ ] ... C[ ]
2ZKRn	CYS75(CB)	4.25	LEU108(CB)	2ZKRn		C[ ] ... C[ ]
2ZKRn	CYS75(CB)	4.72	LEU108(CG)	2ZKRn		
2ZKRn	CYS75(SG)	3.52	LEU108(N)	2ZKRn	[D-A-AA]:84.2° [A-D-DD]:78.0° d_planarity:61.1°, bad d_angle(sp2), bad d_planarity	S[ ] ... N[ ]
2ZKRn	CYS75(SG)	3.52	LEU108(CA)	2ZKRn		S[ ] ... C[ ]
2ZKRn	CYS75(SG)	4.37	LEU108(C)	2ZKRn		S[ ] ... C[ ]
2ZKRn	CYS75(SG)	4.32	LEU108(O)	2ZKRn		S[ ] ... O[ ]
2ZKRn	CYS75(SG)	4.55	LEU108(CB)	2ZKRn		
2ZKRn	CYS75(SG)	4.71	PRO138(N)	2ZKRn		
2ZKRn	CYS75(SG)	4.51	PRO138(CA)	2ZKRn		
2ZKRn	CYS75(SG)	4.27	PRO138(CB)	2ZKRn		S[ ] ... C[ ]
2ZKRn	CYS75(SG)	3.73	PRO138(CG)	2ZKRn		S[ ] ... C[ ]
2ZKRn	CYS75(SG)	4.47	PRO138(CD)	2ZKRn		S[ ] ... C[ ]

chain1	res1/atm1	distance	res2/atm2	chain2	H-bonding	Charge interaction
2ZKRn	CYS99(CA)	4.94	ARG77(CZ)	2ZKRn		
2ZKRn	CYS99(CA)	4.88	ARG77(NH1)	2ZKRn		
2ZKRn	CYS99(CA)	4.48	ARG77(NH2)	2ZKRn		C[ ] ... N[+]
2ZKRn	CYS99(C)	4.55	ARG77(NE)	2ZKRn		
2ZKRn	CYS99(C)	3.68	ARG77(CZ)	2ZKRn		C[ ] ... C[ ]
2ZKRn	CYS99(C)	3.71	ARG77(NH1)	2ZKRn		C[ ] ... N[+]
2ZKRn	CYS99(C)	3.46	ARG77(NH2)	2ZKRn		C[ ] ... N[+]
2ZKRn	CYS99(O)	4.96	ARG77(CD)	2ZKRn		
2ZKRn	CYS99(O)	3.89	ARG77(NE)	2ZKRn	[D-A-AA]:114.5° [A-D-DD]:50.6° d_planarity:-66.2° a_planarity:35.5°, maximum distance exceeded, bad d_angle(sp2), bad d_planarity	O[ ] ... N[ ]
2ZKRn	CYS99(O)	3.22	ARG77(CZ)	2ZKRn		O[ ] ... C[ ]
2ZKRn	CYS99(O)	3.68	ARG77(NH1)	2ZKRn	[D-A-AA]:81.7° [A-D-DD]:59.5° d_planarity:62.3°	O[ ] ... N[+]

					a_planarity:46.9°, maximum distance exceeded, bad a_angle(sp2), bad d_angle(sp2), bad d_planarity	
2ZKRn	CYS99(O)	2.84	ARG77(NH2)	2ZKRn	[D-A-AA]:110.2° [A-D-DD]:94.9° d_planarity:84.0° a_planarity:73.0°, bad d_planarity	O[ ] ... N[+]
2ZKRn	CYS99(CB)	4.84	ARG77(NH1)	2ZKRn		
2ZKRn	CYS99(CB)	4.42	ARG77(NH2)	2ZKRn	Positive	C[ ] ... N[+]
2ZKRn	CYS99(SG)	4.01	ARG77(NH2)	2ZKRn	[D-A-AA]:90.4° [A-D-DD]:134.2° d_planarity:31.7°, maximum distance exceeded	S[ ] ... N[+]
2ZKRn	CYS99(CA)	4.50	LEU82(CD1)	2ZKRn		C[ ] ... C[ ]
2ZKRn	CYS99(C)	4.39	LEU82(CD1)	2ZKRn		C[ ] ... C[ ]
2ZKRn	CYS99(O)	4.60	LEU82(CD1)	2ZKRn		
2ZKRn	CYS99(CB)	4.76	LEU82(CG)	2ZKRn		
2ZKRn	CYS99(CB)	3.41	LEU82(CD1)	2ZKRn		C[ ] ... C[ ]
2ZKRn	CYS99(CB)	4.96	LEU82(CD2)	2ZKRn		
2ZKRn	CYS99(SG)	4.65	LEU82(CG)	2ZKRn		
2ZKRn	CYS99(SG)	3.15	LEU82(CD1)	2ZKRn		S[ ] ... C[ ]
2ZKRn	CYS99(O)	4.95	TYR85(CD1)	2ZKRn		
2ZKRn	CYS99(O)	4.46	TYR85(CE1)	2ZKRn		O[ ] ... C[ ]
2ZKRn	CYS99(SG)	4.60	TYR85(CD1)	2ZKRn		
2ZKRn	CYS99(SG)	4.94	TYR85(CE1)	2ZKRn		
2ZKRn	CYS99(N)	4.94	TYR94(O)	2ZKRn	[D-A-AA]:138.7° [A-D-DD]:152.2° d_planarity:-31.0° a_planarity:12.8°, maximum distance exceeded	
2ZKRn	CYS99(N)	4.15	ALA95(C)	2ZKRn		N[ ] ... C[ ]
2ZKRn	CYS99(N)	2.97	ALA95(O)	2ZKRn	**H-bond** [D-A-AA]:159.9° [A-D-DD]:113.0° d_planarity:-12.0° a_planarity:89.8°	N[ ] ... O[ ]
2ZKRn	CYS99(CA)	3.79	ALA95(O)	2ZKRn		C[ ] ... O[ ]
2ZKRn	CYS99(C)	4.86	ALA95(O)	2ZKRn		
2ZKRn	CYS99(CB)	4.53	ALA95(C)	2ZKRn		
2ZKRn	CYS99(CB)	3.40	ALA95(O)	2ZKRn		C[ ] ... O[ ]
2ZKRn	CYS99(N)	4.93	ALA96(N)	2ZKRn		
2ZKRn	CYS99(N)	4.79	ALA96(CA)	2ZKRn		
2ZKRn	CYS99(N)	4.02	ALA96(C)	2ZKRn		N[ ] ... C[ ]
2ZKRn	CYS99(N)	3.35	ALA96(O)	2ZKRn	[D-A-AA]:114.5° [A-D-DD]:97.9° d_planarity:-73.1° a_planarity:88.5°, bad d_planarity	N[ ] ... O[ ]
2ZKRn	CYS99(CA)	4.74	ALA96(C)	2ZKRn		
2ZKRn	CYS99(CA)	3.84	ALA96(O)	2ZKRn		C[ ] ... O[ ]
2ZKRn	CYS99(C)	4.92	ALA96(C)	2ZKRn		
2ZKRn	CYS99(C)	3.79	ALA96(O)	2ZKRn		C[ ] ... O[ ]
2ZKRn	CYS99(O)	4.93	ALA96(O)	2ZKRn		
2ZKRn	CYS99(CB)	4.82	ALA96(CA)	2ZKRn		
2ZKRn	CYS99(CB)	4.69	ALA96(C)	2ZKRn		
2ZKRn	CYS99(CB)	3.96	ALA96(O)	2ZKRn		C[ ] ... O[ ]
2ZKRn	CYS99(N)	4.62	ALA97(N)	2ZKRn		
2ZKRn	CYS99(N)	4.71	ALA97(CA)	2ZKRn		
2ZKRn	CYS99(N)	3.61	ALA97(C)	2ZKRn		N[ ] ... C[ ]
2ZKRn	CYS99(N)	3.75	ALA97(O)	2ZKRn	[D-A-AA]:73.8° [A-D-DD]:121.1° d_planarity:53.0° a_planarity:46.3°, maximum distance exceeded, bad a_angle(sp2)	N[ ] ... O[ ]
2ZKRn	CYS99(CA)	4.77	ALA97(C)	2ZKRn		
2ZKRn	CYS99(CA)	4.68	ALA97(O)	2ZKRn		
2ZKRn	CYS99(C)	4.81	ALA97(C)	2ZKRn		

2ZKRn	CYS99(C)	4.39	ALA97(O)	2ZKRn		C[ ] ... O[ ]
2ZKRn	CYS99(N)	3.05	TYR98(N)	2ZKRn		N[ ] ... N[ ]
2ZKRn	CYS99(N)	2.47	TYR98(CA)	2ZKRn		N[ ] ... C[ ]
2ZKRn	CYS99(N)	2.24	TYR98(O)	2ZKRn	[D-A-AA]:30.5° [A-D-DD]:95.9° d_planarity:3.7° a_planarity:5.5°, bad a_angle(sp2)	N[ ] ... O[ ]
2ZKRn	CYS99(N)	3.02	TYR98(CB)	2ZKRn		N[ ] ... C[ ]
2ZKRn	CYS99(N)	3.56	TYR98(CG)	2ZKRn		N[ ] ... C[ ]
2ZKRn	CYS99(N)	4.70	TYR98(CD1)	2ZKRn		
2ZKRn	CYS99(N)	3.44	TYR98(CD2)	2ZKRn		N[ ] ... C[ ]
2ZKRn	CYS99(N)	4.67	TYR98(CE2)	2ZKRn		
2ZKRn	CYS99(CA)	4.43	TYR98(N)	2ZKRn		C[ ] ... N[ ]
2ZKRn	CYS99(CA)	3.86	TYR98(CA)	2ZKRn		C[ ] ... C[ ]
2ZKRn	CYS99(CA)	2.47	TYR98(C)	2ZKRn		C[ ] ... C[ ]
2ZKRn	CYS99(CA)	2.80	TYR98(O)	2ZKRn		C[ ] ... O[ ]
2ZKRn	CYS99(CA)	4.39	TYR98(CB)	2ZKRn		C[ ] ... C[ ]
2ZKRn	CYS99(CA)	4.66	TYR98(CG)	2ZKRn		
2ZKRn	CYS99(CA)	4.17	TYR98(CD2)	2ZKRn		C[ ] ... C[ ]
2ZKRn	CYS99(C)	4.89	TYR98(N)	2ZKRn		
2ZKRn	CYS99(C)	4.49	TYR98(CA)	2ZKRn		C[ ] ... C[ ]
2ZKRn	CYS99(C)	3.02	TYR98(C)	2ZKRn		C[ ] ... C[ ]
2ZKRn	CYS99(C)	2.81	TYR98(O)	2ZKRn		C[ ] ... O[ ]
2ZKRn	CYS99(O)	3.95	TYR98(C)	2ZKRn		O[ ] ... C[ ]
2ZKRn	CYS99(O)	3.46	TYR98(O)	2ZKRn		O[ ] ... O[ ]
2ZKRn	CYS99(CB)	4.88	TYR98(CA)	2ZKRn		
2ZKRn	CYS99(CB)	3.73	TYR98(C)	2ZKRn		C[ ] ... C[ ]
2ZKRn	CYS99(CB)	4.28	TYR98(O)	2ZKRn		C[ ] ... O[ ]
2ZKRn	CYS99(CB)	4.90	TYR98(CD2)	2ZKRn		
2ZKRn	CYS99(N)	2.83	THR100(N)	2ZKRn		N[ ] ... N[ ]
2ZKRn	CYS99(N)	4.18	THR100(CA)	2ZKRn		N[ ] ... C[ ]
2ZKRn	CYS99(N)	4.69	THR100(C)	2ZKRn		
2ZKRn	CYS99(N)	4.93	THR100(CB)	2ZKRn		
2ZKRn	CYS99(CA)	2.46	THR100(N)	2ZKRn		C[ ] ... N[ ]
2ZKRn	CYS99(CA)	3.84	THR100(CA)	2ZKRn		C[ ] ... C[ ]
2ZKRn	CYS99(CA)	4.44	THR100(C)	2ZKRn		C[ ] ... C[ ]
2ZKRn	CYS99(CA)	4.90	THR100(CB)	2ZKRn		
2ZKRn	CYS99(C)	2.48	THR100(CA)	2ZKRn		C[ ] ... C[ ]
2ZKRn	CYS99(C)	3.11	THR100(C)	2ZKRn		C[ ] ... C[ ]
2ZKRn	CYS99(C)	3.77	THR100(O)	2ZKRn		C[ ] ... O[ ]
2ZKRn	CYS99(C)	3.76	THR100(CB)	2ZKRn		C[ ] ... C[ ]
2ZKRn	CYS99(C)	4.02	THR100(OG1)	2ZKRn		C[ ] ... O[ ]
2ZKRn	CYS99(O)	2.24	THR100(N)	2ZKRn	[D-A-AA]:30.7° [A-D-DD]:96.9° d_planarity:5.8° a_planarity:2.0°, bad a_angle(sp2)	O[ ] ... N[ ]
2ZKRn	CYS99(O)	2.82	THR100(CA)	2ZKRn		O[ ] ... C[ ]
2ZKRn	CYS99(O)	3.10	THR100(C)	2ZKRn		O[ ] ... C[ ]
2ZKRn	CYS99(O)	3.34	THR100(O)	2ZKRn		O[ ] ... O[ ]
2ZKRn	CYS99(O)	4.30	THR100(CB)	2ZKRn		O[ ] ... C[ ]
2ZKRn	CYS99(O)	4.64	THR100(OG1)	2ZKRn	[D-A-AA]:53.3° [A-D-DD]:67.3° a_planarity:19.1°, maximum distance exceeded, bad a_angle(sp2)	
2ZKRn	CYS99(CB)	3.30	THR100(N)	2ZKRn		C[ ] ... N[ ]
2ZKRn	CYS99(CB)	4.70	THR100(CA)	2ZKRn		

2ZKRn	CYS99(SG)	4.48	THR100(N)	2ZKRn	[D-A-AA]:39.7° [A-D-DD]:141.6° d_planarity:38.9°, maximum distance exceeded, bad a_angle(sp3)	S[ ] ... N[ ]
2ZKRn	CYS99(N)	4.47	GLY101(N)	2ZKRn		N[ ] ... N[ ]
2ZKRn	CYS99(CA)	4.61	GLY101(N)	2ZKRn		
2ZKRn	CYS99(C)	3.60	GLY101(N)	2ZKRn		C[ ] ... N[ ]
2ZKRn	CYS99(C)	4.66	GLY101(CA)	2ZKRn		
2ZKRn	CYS99(C)	4.65	GLY101(C)	2ZKRn		
2ZKRn	CYS99(O)	3.82	GLY101(N)	2ZKRn	[D-A-AA]:70.3° [A-D-DD]:116.6° d_planarity:55.3° a_planarity:50.6°, maximum distance exceeded, bad a_angle(sp2)	O[ ] ... N[ ]
2ZKRn	CYS99(O)	4.66	GLY101(CA)	2ZKRn		
2ZKRn	CYS99(O)	4.31	GLY101(C)	2ZKRn		O[ ] ... C[ ]
2ZKRn	CYS99(N)	4.80	LEU102(N)	2ZKRn		
2ZKRn	CYS99(N)	3.85	LEU102(CD1)	2ZKRn		N[ ] ... C[ ]
2ZKRn	CYS99(CA)	4.54	LEU102(N)	2ZKRn		
2ZKRn	CYS99(CA)	4.55	LEU102(CB)	2ZKRn		
2ZKRn	CYS99(CA)	4.82	LEU102(CG)	2ZKRn		
2ZKRn	CYS99(CA)	3.83	LEU102(CD1)	2ZKRn		C[ ] ... C[ ]
2ZKRn	CYS99(C)	3.90	LEU102(N)	2ZKRn		C[ ] ... N[ ]
2ZKRn	CYS99(C)	4.59	LEU102(CA)	2ZKRn		
2ZKRn	CYS99(C)	4.92	LEU102(C)	2ZKRn		
2ZKRn	CYS99(C)	4.42	LEU102(CB)	2ZKRn		C[ ] ... C[ ]
2ZKRn	CYS99(C)	4.32	LEU102(CD1)	2ZKRn		C[ ] ... C[ ]
2ZKRn	CYS99(O)	3.51	LEU102(N)	2ZKRn	[D-A-AA]:98.8° [A-D-DD]:94.5° d_planarity:-52.7° a_planarity:88.9°, maximum distance exceeded	O[ ] ... N[ ]
2ZKRn	CYS99(O)	3.92	LEU102(CA)	2ZKRn		O[ ] ... C[ ]
2ZKRn	CYS99(O)	3.96	LEU102(C)	2ZKRn		O[ ] ... C[ ]
2ZKRn	CYS99(O)	3.77	LEU102(CB)	2ZKRn		O[ ] ... C[ ]
2ZKRn	CYS99(O)	4.76	LEU102(CG)	2ZKRn		
2ZKRn	CYS99(O)	4.36	LEU102(CD1)	2ZKRn		O[ ] ... C[ ]
2ZKRn	CYS99(C)	4.18	LEU103(N)	2ZKRn		C[ ] ... N[ ]
2ZKRn	CYS99(C)	4.88	LEU103(CB)	2ZKRn		
2ZKRn	CYS99(O)	3.08	LEU103(N)	2ZKRn	**H-bond** [D-A-AA]:148.3° [A-D-DD]:118.0° d_planarity:-17.9° a_planarity:74.7°	O[ ] ... N[ ]
2ZKRn	CYS99(O)	3.97	LEU103(CA)	2ZKRn		O[ ] ... C[ ]
2ZKRn	CYS99(O)	3.73	LEU103(CB)	2ZKRn		O[ ] ... C[ ]
2ZKRn	CYS99(CB)	4.51	HIS194(CD2)	2ZKRn		
2ZKRn	CYS99(CB)	4.09	HIS194(NE2)	2ZKRn		C[ ] ... N[ ]
2ZKRn	CYS99(SG)	4.51	HIS194(NE2)	2ZKRn		
2ZKRn	CYS99(N)	4.34	TYR239(CE1)	2ZKRn		N[ ] ... C[ ]
2ZKRn	CYS99(CA)	4.68	TYR239(CE1)	2ZKRn		
2ZKRn	CYS99(CB)	4.43	TYR239(CD1)	2ZKRn		C[ ] ... C[ ]
2ZKRn	CYS99(CB)	4.23	TYR239(CE1)	2ZKRn		C[ ] ... C[ ]
2ZKRn	CYS99(SG)	4.93	TYR239(CD1)	2ZKRn		
2ZKRn	CYS99(SG)	4.80	TYR239(CE1)	2ZKRn		
2ZKRn	CYS99(SG)	4.90	ALA242(CA)	2ZKRn		
2ZKRn	CYS99(SG)	4.32	ALA242(C)	2ZKRn		S[ ] ... C[ ]
2ZKRn	CYS99(SG)	4.46	ALA242(O)	2ZKRn		S[ ] ... O[ ]
2ZKRn	CYS99(SG)	4.18	ALA242(CB)	2ZKRn		S[ ] ... C[ ]
2ZKRn	CYS99(SG)	4.34	HIS243(N)	2ZKRn	[D-A-AA]:130.0° [A-D-DD]:94.0° d_planarity:80.9°, maximum distance exceeded, bad d_planarity	S[ ] ... N[ ]

2ZKRn	CYS99(SG)	4.68	HIS243(CA)	2ZKRn		
2ZKRn	CYS99(CA)	4.83	ILE246(CD1)	2ZKRn		
2ZKRn	CYS99(O)	4.65	ILE246(CD1)	2ZKRn		
2ZKRn	CYS99(SG)	4.83	ILE246(CG1)	2ZKRn		
2ZKRn	CYS99(SG)	4.07	ILE246(CG2)	2ZKRn		S[ ] ... C[ ]
2ZKRn	CYS99(SG)	4.07	ILE246(CD1)	2ZKRn		S[ ] ... C[ ]

chain1	res1/atm1	distance	res2/atm2	chain2	H-bonding	Charge interaction
2ZKQc	CYS97(SG)	4.92	GLY33(O)	2ZKQc		
2ZKQc	CYS97(CB)	3.99	SER35(OG)	2ZKQc		C[ ] ... O[ ]
2ZKQc	CYS97(SG)	4.67	SER35(CA)	2ZKQc		
2ZKQc	CYS97(SG)	3.90	SER35(CB)	2ZKQc		S[ ] ... C[ ]
2ZKQc	CYS97(SG)	2.95	SER35(OG)	2ZKQc	**H-bond** [D-A-AA]:111.3° [A-D-DD]:123.2°	S[ ] ... O[ ]
2ZKQc	CYS97(CB)	4.25	THR53(OG1)	2ZKQc		C[ ] ... O[ ]
2ZKQc	CYS97(SG)	4.81	THR53(OG1)	2ZKQc	[D-A-AA]:61.3° [A-D-DD]:157.6°, maximum distance exceeded	
2ZKQc	CYS97(CB)	4.94	VAL91(CG1)	2ZKQc		
2ZKQc	CYS97(N)	4.99	THR93(C)	2ZKQc		
2ZKQc	CYS97(N)	3.97	THR93(O)	2ZKQc	[D-A-AA]:141.3° [A-D-DD]:132.2° d_planarity:-41.6° a_planarity:13.9°, maximum distance exceeded	N[ ] ... O[ ]
2ZKQc	CYS97(CB)	4.97	THR93(O)	2ZKQc		
2ZKQc	CYS97(N)	4.27	ARG94(CA)	2ZKQc		N[ ] ... C[ ]
2ZKQc	CYS97(N)	3.52	ARG94(C)	2ZKQc		N[ ] ... C[ ]
2ZKQc	CYS97(N)	2.85	ARG94(O)	2ZKQc	**H-bond** [D-A-AA]:113.6° [A-D-DD]:105.4° d_planarity:-27.9° a_planarity:-89.4°	N[ ] ... O[ ]
2ZKQc	CYS97(CA)	4.95	ARG94(CA)	2ZKQc		
2ZKQc	CYS97(CA)	4.41	ARG94(C)	2ZKQc		C[ ] ... C[ ]
2ZKQc	CYS97(CA)	3.52	ARG94(O)	2ZKQc		C[ ] ... O[ ]
2ZKQc	CYS97(C)	4.90	ARG94(C)	2ZKQc		
2ZKQc	CYS97(C)	3.77	ARG94(O)	2ZKQc		C[ ] ... O[ ]
2ZKQc	CYS97(O)	4.53	ARG94(C)	2ZKQc		
2ZKQc	CYS97(O)	3.32	ARG94(O)	2ZKQc		O[ ] ... O[ ]
2ZKQc	CYS97(CB)	4.46	ARG94(CA)	2ZKQc		C[ ] ... C[ ]
2ZKQc	CYS97(CB)	4.39	ARG94(C)	2ZKQc		C[ ] ... C[ ]
2ZKQc	CYS97(CB)	3.64	ARG94(O)	2ZKQc		C[ ] ... O[ ]
2ZKQc	CYS97(N)	4.27	GLY95(N)	2ZKQc		N[ ] ... N[ ]
2ZKQc	CYS97(N)	4.40	GLY95(CA)	2ZKQc		N[ ] ... C[ ]
2ZKQc	CYS97(N)	3.40	GLY95(C)	2ZKQc		N[ ] ... C[ ]
2ZKQc	CYS97(N)	3.55	GLY95(O)	2ZKQc	[D-A-AA]:72.9° [A-D-DD]:133.4° d_planarity:82.7° a_planarity:54.5°, maximum distance exceeded, bad a_angle(sp2), bad d_planarity	N[ ] ... O[ ]
2ZKQc	CYS97(CA)	4.70	GLY95(C)	2ZKQc		
2ZKQc	CYS97(CA)	4.67	GLY95(O)	2ZKQc		
2ZKQc	CYS97(C)	4.94	GLY95(C)	2ZKQc		
2ZKQc	CYS97(C)	4.59	GLY95(O)	2ZKQc		
2ZKQc	CYS97(O)	4.92	GLY95(C)	2ZKQc		
2ZKQc	CYS97(O)	4.59	GLY95(O)	2ZKQc		

2ZKQc	CYS97(N)	2.85	LEU96(N)	2ZKQc		N[ ] ... N[ ]
2ZKQc	CYS97(N)	2.47	LEU96(CA)	2ZKQc		N[ ] ... C[ ]
2ZKQc	CYS97(N)	2.23	LEU96(O)	2ZKQc	[D-A-AA]:31.1° [A-D-DD]:92.9° d_planarity:1.1° a_planarity:1.0°, bad a_angle(sp2)	N[ ] ... O[ ]
2ZKQc	CYS97(N)	3.32	LEU96(CB)	2ZKQc		N[ ] ... C[ ]
2ZKQc	CYS97(N)	4.68	LEU96(CG)	2ZKQc		
2ZKQc	CYS97(CA)	4.30	LEU96(N)	2ZKQc		C[ ] ... N[ ]
2ZKQc	CYS97(CA)	3.84	LEU96(CA)	2ZKQc		C[ ] ... C[ ]
2ZKQc	CYS97(CA)	2.44	LEU96(C)	2ZKQc		C[ ] ... C[ ]
2ZKQc	CYS97(CA)	2.73	LEU96(O)	2ZKQc		C[ ] ... O[ ]
2ZKQc	CYS97(CA)	4.57	LEU96(CB)	2ZKQc		
2ZKQc	CYS97(C)	4.93	LEU96(N)	2ZKQc		
2ZKQc	CYS97(C)	4.59	LEU96(CA)	2ZKQc		
2ZKQc	CYS97(C)	3.21	LEU96(C)	2ZKQc		C[ ] ... C[ ]
2ZKQc	CYS97(C)	3.28	LEU96(O)	2ZKQc		C[ ] ... O[ ]
2ZKQc	CYS97(O)	3.99	LEU96(C)	2ZKQc		O[ ] ... C[ ]
2ZKQc	CYS97(O)	4.29	LEU96(O)	2ZKQc		O[ ] ... O[ ]
2ZKQc	CYS97(CB)	4.89	LEU96(CA)	2ZKQc		
2ZKQc	CYS97(CB)	3.69	LEU96(C)	2ZKQc		C[ ] ... C[ ]
2ZKQc	CYS97(CB)	4.14	LEU96(O)	2ZKQc		C[ ] ... O[ ]
2ZKQc	CYS97(N)	3.39	ALA98(N)	2ZKQc		N[ ] ... N[ ]
2ZKQc	CYS97(N)	4.73	ALA98(CA)	2ZKQc		
2ZKQc	CYS97(CA)	2.43	ALA98(N)	2ZKQc		C[ ] ... N[ ]
2ZKQc	CYS97(CA)	3.81	ALA98(CA)	2ZKQc		C[ ] ... C[ ]
2ZKQc	CYS97(CA)	4.28	ALA98(C)	2ZKQc		C[ ] ... C[ ]
2ZKQc	CYS97(CA)	4.84	ALA98(CB)	2ZKQc		
2ZKQc	CYS97(C)	2.45	ALA98(CA)	2ZKQc		C[ ] ... C[ ]
2ZKQc	CYS97(C)	2.91	ALA98(C)	2ZKQc		C[ ] ... C[ ]
2ZKQc	CYS97(C)	3.62	ALA98(O)	2ZKQc		C[ ] ... O[ ]
2ZKQc	CYS97(C)	3.73	ALA98(CB)	2ZKQc		C[ ] ... C[ ]
2ZKQc	CYS97(O)	2.25	ALA98(N)	2ZKQc	[D-A-AA]:30.2° [A-D-DD]:95.2° d_planarity:1.7° a_planarity:8.2°, bad a_angle(sp2)	O[ ] ... N[ ]
2ZKQc	CYS97(O)	2.79	ALA98(CA)	2ZKQc		O[ ] ... C[ ]
2ZKQc	CYS97(O)	2.75	ALA98(C)	2ZKQc		O[ ] ... C[ ]
2ZKQc	CYS97(O)	3.05	ALA98(O)	2ZKQc		O[ ] ... O[ ]
2ZKQc	CYS97(O)	4.28	ALA98(CB)	2ZKQc		O[ ] ... C[ ]
2ZKQc	CYS97(CB)	3.53	ALA98(N)	2ZKQc		C[ ] ... N[ ]
2ZKQc	CYS97(CB)	4.72	ALA98(CA)	2ZKQc		
2ZKQc	CYS97(CB)	4.74	ALA98(C)	2ZKQc		
2ZKQc	CYS97(SG)	3.58	ALA98(N)	2ZKQc	[D-A-AA]:73.6° [A-D-DD]:119.2° d_planarity:69.4°, bad d_planarity	S[ ] ... N[ ]
2ZKQc	CYS97(SG)	4.47	ALA98(CA)	2ZKQc		S[ ] ... C[ ]
2ZKQc	CYS97(SG)	4.19	ALA98(C)	2ZKQc		S[ ] ... C[ ]
2ZKQc	CYS97(CA)	4.29	ILE99(N)	2ZKQc		C[ ] ... N[ ]
2ZKQc	CYS97(C)	3.24	ILE99(N)	2ZKQc		C[ ] ... N[ ]
2ZKQc	CYS97(C)	4.43	ILE99(CA)	2ZKQc		C[ ] ... C[ ]
2ZKQc	CYS97(C)	4.64	ILE99(C)	2ZKQc		
2ZKQc	CYS97(O)	3.24	ILE99(N)	2ZKQc	[D-A-AA]:78.9° [A-D-DD]:119.5° d_planarity:66.6° a_planarity:56.7°, bad a_angle(sp2), bad d_planarity	O[ ] ... N[ ]
2ZKQc	CYS97(O)	4.17	ILE99(CA)	2ZKQc		O[ ] ... C[ ]

2ZKQc	CYS97(O)	3.98	ILE99(C)	2ZKQc		O[ ] ... C[ ]
2ZKQc	CYS97(O)	4.95	ILE99(O)	2ZKQc		
2ZKQc	CYS97(CB)	4.40	ILE99(N)	2ZKQc		C[ ] ... N[ ]
2ZKQc	CYS97(SG)	3.38	ILE99(N)	2ZKQc	**H-bond** [D-A-AA]:111.9° [A-D-DD]:101.2° d_planarity:-46.2°	S[ ] ... N[ ]
2ZKQc	CYS97(SG)	3.94	ILE99(CA)	2ZKQc		S[ ] ... C[ ]
2ZKQc	CYS97(SG)	4.41	ILE99(C)	2ZKQc		S[ ] ... C[ ]
2ZKQc	CYS97(SG)	3.72	ILE99(CB)	2ZKQc		S[ ] ... C[ ]
2ZKQc	CYS97(SG)	4.42	ILE99(CG1)	2ZKQc		S[ ] ... C[ ]
2ZKQc	CYS97(SG)	4.86	ILE99(CG2)	2ZKQc		
2ZKQc	CYS97(SG)	4.78	ILE99(CD1)	2ZKQc		
2ZKQc	CYS97(CA)	4.77	ALA100(N)	2ZKQc		
2ZKQc	CYS97(CA)	4.85	ALA100(CB)	2ZKQc		
2ZKQc	CYS97(C)	3.97	ALA100(N)	2ZKQc		C[ ] ... N[ ]
2ZKQc	CYS97(C)	4.70	ALA100(CA)	2ZKQc		
2ZKQc	CYS97(C)	4.98	ALA100(C)	2ZKQc		
2ZKQc	CYS97(C)	4.51	ALA100(CB)	2ZKQc		
2ZKQc	CYS97(O)	3.19	ALA100(N)	2ZKQc	**H-bond** [D-A-AA]:121.2° [A-D-DD]:97.9° d_planarity:-50.6° a_planarity:89.8°	O[ ] ... N[ ]
2ZKQc	CYS97(O)	3.68	ALA100(CA)	2ZKQc		O[ ] ... C[ ]
2ZKQc	CYS97(O)	3.79	ALA100(C)	2ZKQc		O[ ] ... C[ ]
2ZKQc	CYS97(O)	4.99	ALA100(O)	2ZKQc		
2ZKQc	CYS97(O)	3.56	ALA100(CB)	2ZKQc		O[ ] ... C[ ]
2ZKQc	CYS97(CB)	4.26	ALA100(N)	2ZKQc		C[ ] ... N[ ]
2ZKQc	CYS97(CB)	4.85	ALA100(CA)	2ZKQc		
2ZKQc	CYS97(CB)	4.10	ALA100(CB)	2ZKQc		C[ ] ... C[ ]
2ZKQc	CYS97(SG)	3.77	ALA100(N)	2ZKQc	**H-bond** [D-A-AA]:92.5° [A-D-DD]:127.1° d_planarity:-8.4°	S[ ] ... N[ ]
2ZKQc	CYS97(SG)	4.78	ALA100(CA)	2ZKQc		
2ZKQc	CYS97(SG)	4.51	ALA100(CB)	2ZKQc		
2ZKQc	CYS97(C)	4.17	GLN101(N)	2ZKQc		C[ ] ... N[ ]
2ZKQc	CYS97(C)	5.00	GLN101(CA)	2ZKQc		
2ZKQc	CYS97(C)	4.44	GLN101(CB)	2ZKQc		C[ ] ... C[ ]
2ZKQc	CYS97(O)	2.97	GLN101(N)	2ZKQc	**H-bond** [D-A-AA]:163.7° [A-D-DD]:118.0° d_planarity:-5.2° a_planarity:16.6°	O[ ] ... N[ ]
2ZKQc	CYS97(O)	3.87	GLN101(CA)	2ZKQc		O[ ] ... C[ ]
2ZKQc	CYS97(O)	3.55	GLN101(CB)	2ZKQc		O[ ] ... C[ ]
2ZKQc	CYS97(N)	4.55	ASP169(OD2)	2ZKQc	[D-A-AA]:148.0° [A-D-DD]:38.8° d_planarity:12.3° a_planarity:32.3°, maximum distance exceeded, bad d_angle(sp2)	
2ZKQc	CYS97(CA)	4.79	ASP169(CG)	2ZKQc		
2ZKQc	CYS97(CA)	3.53	ASP169(OD2)	2ZKQc		C[ ] ... O[-]
2ZKQc	CYS97(C)	4.70	ASP169(CG)	2ZKQc		
2ZKQc	CYS97(C)	3.67	ASP169(OD2)	2ZKQc		C[ ] ... O[-]
2ZKQc	CYS97(O)	4.83	ASP169(OD2)	2ZKQc		
2ZKQc	CYS97(CB)	4.28	ASP169(OD2)	2ZKQc		C[ ] ... O[-]
2ZKQc	CYS97(SG)	4.96	ASP169(CG)	2ZKQc		
2ZKQc	CYS97(SG)	3.77	ASP169(OD2)	2ZKQc	Negative	S[ ] ... O[-]
2ZKQc	CYS97(C)	4.78	ILE188(CG1)	2ZKQc		
2ZKQc	CYS97(C)	4.20	ILE188(CD1)	2ZKQc		C[ ] ... C[ ]
2ZKQc	CYS97(O)	4.64	ILE188(CD1)	2ZKQc		

chain1	res1/atm1	distance	res2/atm2	chain2	H-bonding	Charge interaction
1YY9A	CYS267(N)	5.00	PHE230(CB)	1YY9A		
1YY9A	CYS267(N)	5.00	PHE230(CD2)	1YY9A		
1YY9A	CYS267(SG)	4.96	PHE230(CA)	1YY9A		
1YY9A	CYS267(SG)	3.76	PHE230(CB)	1YY9A		S[ ] ... C[ ]
1YY9A	CYS267(SG)	4.36	PHE230(CG)	1YY9A		S[ ] ... C[ ]
1YY9A	CYS267(SG)	4.54	PHE230(CD2)	1YY9A		
1YY9A	CYS267(N)	4.66	ARG231(N)	1YY9A		
1YY9A	CYS267(N)	4.89	ARG231(CA)	1YY9A		
1YY9A	CYS267(N)	3.89	ARG231(C)	1YY9A		N[ ] ... C[ ]
1YY9A	CYS267(N)	2.71	ARG231(O)	1YY9A	**H-bond** [D-A-AA]:160.7° [A-D-DD]:118.4° d_planarity:-13.2° a_planarity:76.2°	N[ ] ... O[ ]
1YY9A	CYS267(CA)	4.69	ARG231(C)	1YY9A		
1YY9A	CYS267(CA)	3.64	ARG231(O)	1YY9A		C[ ] ... O[ ]
1YY9A	CYS267(C)	4.18	ARG231(O)	1YY9A		C[ ] ... O[ ]
1YY9A	CYS267(O)	4.69	ARG231(C)	1YY9A		
1YY9A	CYS267(O)	3.77	ARG231(O)	1YY9A		O[ ] ... O[ ]
1YY9A	CYS267(CB)	4.34	ARG231(C)	1YY9A		C[ ] ... C[ ]
1YY9A	CYS267(CB)	3.60	ARG231(O)	1YY9A		C[ ] ... O[ ]
1YY9A	CYS267(SG)	4.80	ARG231(N)	1YY9A	[D-A-AA]:106.0° [A-D-DD]:95.5° d_planarity:-85.3°, maximum distance exceeded, bad d_planarity	
1YY9A	CYS267(SG)	4.30	ARG231(C)	1YY9A		S[ ] ... C[ ]
1YY9A	CYS267(SG)	3.78	ARG231(O)	1YY9A	Weakly positive	S[ ] ... O[ ]
1YY9A	CYS267(N)	4.69	ASP232(N)	1YY9A		
1YY9A	CYS267(N)	4.59	ASP232(CA)	1YY9A		
1YY9A	CYS267(CA)	4.71	ASP232(CA)	1YY9A		
1YY9A	CYS267(CA)	4.81	ASP232(CB)	1YY9A		
1YY9A	CYS267(CA)	4.93	ASP232(CG)	1YY9A		
1YY9A	CYS267(CA)	4.58	ASP232(OD1)	1YY9A		
1YY9A	CYS267(C)	4.74	ASP232(CA)	1YY9A		
1YY9A	CYS267(C)	4.99	ASP232(CB)	1YY9A		
1YY9A	CYS267(C)	4.79	ASP232(CG)	1YY9A		
1YY9A	CYS267(C)	4.09	ASP232(OD1)	1YY9A		C[ ] ... O[-]
1YY9A	CYS267(O)	4.82	ASP232(N)	1YY9A	[D-A-AA]:117.3° [A-D-DD]:46.3° d_planarity:6.2° a_planarity:-12.0°, maximum distance exceeded, bad d_angle(sp2)	
1YY9A	CYS267(O)	3.96	ASP232(CA)	1YY9A		O[ ] ... C[ ]
1YY9A	CYS267(O)	4.68	ASP232(C)	1YY9A		
1YY9A	CYS267(O)	4.47	ASP232(CB)	1YY9A		O[ ] ... C[ ]
1YY9A	CYS267(O)	4.29	ASP232(CG)	1YY9A		O[ ] ... C[ ]
1YY9A	CYS267(O)	3.43	ASP232(OD1)	1YY9A		O[ ] ... O[-]
1YY9A	CYS267(CB)	4.54	ASP232(N)	1YY9A		
1YY9A	CYS267(CB)	3.99	ASP232(CA)	1YY9A		C[ ] ... C[ ]
1YY9A	CYS267(CB)	3.67	ASP232(CB)	1YY9A		C[ ] ... C[ ]
1YY9A	CYS267(CB)	3.78	ASP232(CG)	1YY9A		C[ ] ... C[ ]
1YY9A	CYS267(CB)	3.74	ASP232(OD1)	1YY9A		C[ ] ... O[-]

1YY9A	CYS267(CB)	4.50	ASP232(OD2)	1YY9A		C[ ] ... O[-]
1YY9A	CYS267(SG)	4.66	ASP232(N)	1YY9A	[D-A-AA]:75.2° [A-D-DD]:77.2° d_planarity:50.3°, maximum distance exceeded, bad d_angle(sp2)	
1YY9A	CYS267(SG)	4.56	ASP232(CA)	1YY9A		
1YY9A	CYS267(SG)	4.06	ASP232(CB)	1YY9A		S[ ] ... C[ ]
1YY9A	CYS267(SG)	4.60	ASP232(CG)	1YY9A		
1YY9A	CYS267(SG)	4.96	ASP232(OD1)	1YY9A	Negative	
1YY9A	CYS267(O)	4.44	GLU233(N)	1YY9A	[D-A-AA]:155.7° [A-D-DD]:138.5° d_planarity:-35.6° a_planarity:-29.7°, maximum distance exceeded	O[ ] ... N[ ]
1YY9A	CYS267(SG)	4.48	THR239(C)	1YY9A		S[ ] ... C[ ]
1YY9A	CYS267(SG)	4.61	THR239(O)	1YY9A		
1YY9A	CYS267(N)	3.72	CYS240(SG)	1YY9A	[D-A-AA]:144.8° [A-D-DD]:61.6° d_planarity:68.3°, bad d_angle(sp2), bad d_planarity	N[ ] ... S[ ]
1YY9A	CYS267(CA)	4.53	CYS240(CB)	1YY9A		
1YY9A	CYS267(CA)	3.29	CYS240(SG)	1YY9A		C[ ] ... S[ ]
1YY9A	CYS267(C)	4.69	CYS240(SG)	1YY9A		
1YY9A	CYS267(CB)	4.35	CYS240(CA)	1YY9A		C[ ] ... C[ ]
1YY9A	CYS267(CB)	3.92	CYS240(CB)	1YY9A		C[ ] ... C[ ]
1YY9A	CYS267(CB)	3.02	CYS240(SG)	1YY9A		C[ ] ... S[ ]
1YY9A	CYS267(SG)	3.77	CYS240(N)	1YY9A	[D-A-AA]:142.3° [A-D-DD]:55.7° d_planarity:-80.4°, bad d_angle(sp2), bad d_planarity	S[ ] ... N[ ]
1YY9A	CYS267(SG)	3.18	CYS240(CA)	1YY9A		S[ ] ... C[ ]
1YY9A	CYS267(SG)	4.50	CYS240(C)	1YY9A		S[ ] ... C[ ]
1YY9A	CYS267(SG)	3.05	CYS240(CB)	1YY9A		S[ ] ... C[ ]
1YY9A	CYS267(SG)	2.04	CYS240(SG)	1YY9A		S[ ] ... S[ ]
1YY9A	CYS267(CB)	4.38	PRO241(CD)	1YY9A		C[ ] ... C[ ]
1YY9A	CYS267(SG)	4.74	PRO241(N)	1YY9A		
1YY9A	CYS267(SG)	4.09	PRO241(CD)	1YY9A		S[ ] ... C[ ]
1YY9A	CYS267(CA)	4.77	LYS260(CB)	1YY9A		
1YY9A	CYS267(C)	4.55	LYS260(CB)	1YY9A		
1YY9A	CYS267(C)	4.74	LYS260(CG)	1YY9A		
1YY9A	CYS267(C)	4.69	LYS260(CD)	1YY9A		
1YY9A	CYS267(O)	4.78	LYS260(CD)	1YY9A		
1YY9A	CYS267(CB)	4.32	LYS260(CB)	1YY9A		C[ ] ... C[ ]
1YY9A	CYS267(CB)	4.88	LYS260(CG)	1YY9A		
1YY9A	CYS267(CB)	4.49	LYS260(CD)	1YY9A		C[ ] ... C[ ]
1YY9A	CYS267(N)	4.52	TYR261(O)	1YY9A	[D-A-AA]:150.0° [A-D-DD]:37.7° d_planarity:4.9° a_planarity:6.4°, maximum distance exceeded, bad d_angle(sp2)	
1YY9A	CYS267(CA)	4.65	TYR261(C)	1YY9A		
1YY9A	CYS267(CA)	3.48	TYR261(O)	1YY9A		C[ ] ... O[ ]
1YY9A	CYS267(C)	4.98	TYR261(C)	1YY9A		
1YY9A	CYS267(C)	3.76	TYR261(O)	1YY9A		C[ ] ... O[ ]
1YY9A	CYS267(O)	4.98	TYR261(O)	1YY9A		
1YY9A	CYS267(CB)	4.24	TYR261(O)	1YY9A		C[ ] ... O[ ]
1YY9A	CYS267(CA)	4.79	SER262(CA)	1YY9A		
1YY9A	CYS267(N)	4.98	PHE263(N)	1YY9A		
1YY9A	CYS267(N)	4.91	PHE263(O)	1YY9A	[D-A-AA]:125.2° [A-D-DD]:120.2° d_planarity:19.6° a_planarity:-51.4°, maximum distance exceeded	
1YY9A	CYS267(N)	4.51	ALA265(C)	1YY9A		
1YY9A	CYS267(N)	4.62	ALA265(O)	1YY9A	[D-A-AA]:77.2° [A-D-DD]:159.7° d_planarity:79.6°	

					a_planarity:14.4°, maximum distance exceeded, bad a_angle(sp2), bad d_planarity	
1YY9A	CYS267(N)	3.56	THR266(N)	1YY9A		N[ ] ... N[ ]
1YY9A	CYS267(N)	2.40	THR266(CA)	1YY9A		N[ ] ... C[ ]
1YY9A	CYS267(N)	2.27	THR266(O)	1YY9A	[D-A-AA]:29.1° [A-D-DD]:97.2° d_planarity:3.4° a_planarity:1.2°, bad a_angle(sp2)	N[ ] ... O[ ]
1YY9A	CYS267(N)	3.28	THR266(CB)	1YY9A		N[ ] ... C[ ]
1YY9A	CYS267(N)	4.07	THR266(OG1)	1YY9A	[D-A-AA]:47.5° [A-D-DD]:151.6° d_planarity:-88.8°, maximum distance exceeded, bad a_angle(sp3), bad d_planarity	N[ ] ... O[ ]
1YY9A	CYS267(N)	3.15	THR266(CG2)	1YY9A		N[ ] ... C[ ]
1YY9A	CYS267(CA)	4.85	THR266(N)	1YY9A		
1YY9A	CYS267(CA)	3.79	THR266(CA)	1YY9A		C[ ] ... C[ ]
1YY9A	CYS267(CA)	2.46	THR266(C)	1YY9A		C[ ] ... C[ ]
1YY9A	CYS267(CA)	2.85	THR266(O)	1YY9A		C[ ] ... O[ ]
1YY9A	CYS267(CA)	4.54	THR266(CB)	1YY9A		
1YY9A	CYS267(CA)	4.05	THR266(CG2)	1YY9A		C[ ] ... C[ ]
1YY9A	CYS267(C)	4.52	THR266(CA)	1YY9A		
1YY9A	CYS267(C)	3.36	THR266(C)	1YY9A		C[ ] ... C[ ]
1YY9A	CYS267(C)	3.78	THR266(O)	1YY9A		C[ ] ... O[ ]
1YY9A	CYS267(C)	4.81	THR266(CB)	1YY9A		
1YY9A	CYS267(C)	3.89	THR266(CG2)	1YY9A		C[ ] ... C[ ]
1YY9A	CYS267(O)	4.74	THR266(CA)	1YY9A		
1YY9A	CYS267(O)	3.89	THR266(C)	1YY9A		O[ ] ... C[ ]
1YY9A	CYS267(O)	4.59	THR266(O)	1YY9A		
1YY9A	CYS267(O)	4.86	THR266(CB)	1YY9A		
1YY9A	CYS267(O)	3.93	THR266(CG2)	1YY9A		O[ ] ... C[ ]
1YY9A	CYS267(CB)	4.79	THR266(CA)	1YY9A		
1YY9A	CYS267(CB)	3.61	THR266(C)	1YY9A		C[ ] ... C[ ]
1YY9A	CYS267(CB)	4.07	THR266(O)	1YY9A		C[ ] ... O[ ]
1YY9A	CYS267(SG)	4.90	THR266(CA)	1YY9A		
1YY9A	CYS267(SG)	3.90	THR266(C)	1YY9A		S[ ] ... C[ ]
1YY9A	CYS267(SG)	4.28	THR266(O)	1YY9A		S[ ] ... O[ ]
1YY9A	CYS267(N)	3.44	VAL268(N)	1YY9A		N[ ] ... N[ ]
1YY9A	CYS267(N)	4.58	VAL268(CA)	1YY9A		
1YY9A	CYS267(N)	4.83	VAL268(CB)	1YY9A		
1YY9A	CYS267(CA)	2.38	VAL268(N)	1YY9A		C[ ] ... N[ ]
1YY9A	CYS267(CA)	3.73	VAL268(CA)	1YY9A		C[ ] ... C[ ]
1YY9A	CYS267(CA)	4.76	VAL268(C)	1YY9A		
1YY9A	CYS267(CA)	4.87	VAL268(O)	1YY9A		
1YY9A	CYS267(CA)	4.32	VAL268(CB)	1YY9A		C[ ] ... C[ ]
1YY9A	CYS267(C)	2.44	VAL268(CA)	1YY9A		C[ ] ... C[ ]
1YY9A	CYS267(C)	3.61	VAL268(C)	1YY9A		C[ ] ... C[ ]
1YY9A	CYS267(C)	4.02	VAL268(O)	1YY9A		C[ ] ... O[ ]
1YY9A	CYS267(C)	3.26	VAL268(CB)	1YY9A		C[ ] ... C[ ]
1YY9A	CYS267(C)	4.57	VAL268(CG1)	1YY9A		
1YY9A	CYS267(C)	3.90	VAL268(CG2)	1YY9A		C[ ] ... C[ ]
1YY9A	CYS267(O)	2.26	VAL268(N)	1YY9A	[D-A-AA]:28.9° [A-D-DD]:99.0° d_planarity:10.0° a_planarity:4.2°, bad a_angle(sp2)	O[ ] ... N[ ]
1YY9A	CYS267(O)	2.86	VAL268(CA)	1YY9A		O[ ] ... C[ ]

1YY9A	CYS267(O)	4.10	VAL268(C)	1YY9A		O[ ] ... C[ ]
1YY9A	CYS267(O)	4.78	VAL268(O)	1YY9A		
1YY9A	CYS267(O)	3.73	VAL268(CB)	1YY9A		O[ ] ... C[ ]
1YY9A	CYS267(O)	3.93	VAL268(CG2)	1YY9A		O[ ] ... C[ ]
1YY9A	CYS267(CB)	3.29	VAL268(N)	1YY9A		C[ ] ... N[ ]
1YY9A	CYS267(CB)	4.64	VAL268(CA)	1YY9A		
1YY9A	CYS267(SG)	4.88	VAL268(N)	1YY9A	[D-A-AA]:20.8° [A-D-DD]:166.2° d_planarity:25.2°, maximum distance exceeded, bad a_angle(sp3)	
1YY9A	CYS267(C)	4.58	LYS269(N)	1YY9A		
1YY9A	CYS267(O)	4.79	LYS269(N)	1YY9A	[D-A-AA]:72.9° [A-D-DD]:151.3° d_planarity:65.0° a_planarity:12.9°, maximum distance exceeded, bad a_angle(sp2), bad d_planarity	

chain1	res1/atm1	distance	res2/atm2	chain2	H-bonding	Charge interaction
1YY9A	CYS287(N)	4.79	VAL276(CB)	1YY9A		
1YY9A	CYS287(N)	4.86	VAL276(CG1)	1YY9A		
1YY9A	CYS287(N)	4.72	VAL276(CG2)	1YY9A		
1YY9A	CYS287(CB)	4.94	VAL276(CG1)	1YY9A		
1YY9A	CYS287(SG)	4.39	VAL276(CB)	1YY9A		S[ ] ... C[ ]
1YY9A	CYS287(SG)	3.46	VAL276(CG1)	1YY9A		S[ ] ... C[ ]
1YY9A	CYS287(SG)	4.56	VAL276(CG2)	1YY9A		
1YY9A	CYS287(N)	4.83	ARG285(C)	1YY9A		
1YY9A	CYS287(N)	3.67	ALA286(N)	1YY9A		N[ ] ... N[ ]
1YY9A	CYS287(N)	2.44	ALA286(CA)	1YY9A		N[ ] ... C[ ]
1YY9A	CYS287(N)	2.27	ALA286(O)	1YY9A	[D-A-AA]:29.4° [A-D-DD]:97.3° d_planarity:5.0° a_planarity:0.9°, bad a_angle(sp2)	N[ ] ... O[ ]
1YY9A	CYS287(N)	3.19	ALA286(CB)	1YY9A		N[ ] ... C[ ]
1YY9A	CYS287(CA)	4.93	ALA286(N)	1YY9A		
1YY9A	CYS287(CA)	3.85	ALA286(CA)	1YY9A		C[ ] ... C[ ]
1YY9A	CYS287(CA)	2.49	ALA286(C)	1YY9A		C[ ] ... C[ ]
1YY9A	CYS287(CA)	2.87	ALA286(O)	1YY9A		C[ ] ... O[ ]
1YY9A	CYS287(CA)	4.60	ALA286(CB)	1YY9A		
1YY9A	CYS287(C)	4.71	ALA286(CA)	1YY9A		
1YY9A	CYS287(C)	3.25	ALA286(C)	1YY9A		C[ ] ... C[ ]
1YY9A	CYS287(C)	3.20	ALA286(O)	1YY9A		C[ ] ... O[ ]
1YY9A	CYS287(O)	4.94	ALA286(CA)	1YY9A		
1YY9A	CYS287(O)	3.65	ALA286(C)	1YY9A		O[ ] ... C[ ]
1YY9A	CYS287(O)	3.69	ALA286(O)	1YY9A		O[ ] ... O[ ]
1YY9A	CYS287(CB)	4.87	ALA286(CA)	1YY9A		
1YY9A	CYS287(CB)	3.75	ALA286(C)	1YY9A		C[ ] ... C[ ]
1YY9A	CYS287(CB)	4.34	ALA286(O)	1YY9A		C[ ] ... O[ ]
1YY9A	CYS287(SG)	4.44	ALA286(C)	1YY9A		S[ ] ... C[ ]
1YY9A	CYS287(N)	3.64	GLY288(N)	1YY9A		N[ ] ... N[ ]
1YY9A	CYS287(N)	4.96	GLY288(CA)	1YY9A		
1YY9A	CYS287(CA)	2.41	GLY288(N)	1YY9A		C[ ] ... N[ ]
1YY9A	CYS287(CA)	3.79	GLY288(CA)	1YY9A		C[ ] ... C[ ]
1YY9A	CYS287(CA)	4.36	GLY288(C)	1YY9A		C[ ] ... C[ ]

1YY9A	CYS287(CA)	4.26	GLY288(O)	1YY9A		C[ ] ... O[ ]
1YY9A	CYS287(C)	2.44	GLY288(CA)	1YY9A		C[ ] ... C[ ]
1YY9A	CYS287(C)	3.02	GLY288(C)	1YY9A		C[ ] ... C[ ]
1YY9A	CYS287(C)	3.23	GLY288(O)	1YY9A		C[ ] ... O[ ]
1YY9A	CYS287(O)	2.25	GLY288(N)	1YY9A	[D-A-AA]:29.5° [A-D-DD]:95.0° d_planarity:3.1° a_planarity:4.4°, bad a_angle(sp2)	O[ ] ... N[ ]
1YY9A	CYS287(O)	2.79	GLY288(CA)	1YY9A		O[ ] ... C[ ]
1YY9A	CYS287(O)	2.98	GLY288(C)	1YY9A		O[ ] ... C[ ]
1YY9A	CYS287(O)	3.31	GLY288(O)	1YY9A		O[ ] ... O[ ]
1YY9A	CYS287(CB)	3.23	GLY288(N)	1YY9A		C[ ] ... N[ ]
1YY9A	CYS287(CB)	4.43	GLY288(CA)	1YY9A		C[ ] ... C[ ]
1YY9A	CYS287(CB)	4.53	GLY288(C)	1YY9A		
1YY9A	CYS287(CB)	4.01	GLY288(O)	1YY9A		C[ ] ... O[ ]
1YY9A	CYS287(SG)	4.59	GLY288(N)	1YY9A	[D-A-AA]:32.2° [A-D-DD]:147.5° d_planarity:77.0°, maximum distance exceeded, bad a_angle(sp3), bad d_planarity	
1YY9A	CYS287(C)	3.98	ALA289(N)	1YY9A		C[ ] ... N[ ]
1YY9A	CYS287(O)	3.57	ALA289(N)	1YY9A	[D-A-AA]:100.1° [A-D-DD]:130.6° d_planarity:65.8° a_planarity:25.8°, maximum distance exceeded, bad d_planarity	O[ ] ... N[ ]
1YY9A	CYS287(O)	4.66	ALA289(CA)	1YY9A		
1YY9A	CYS287(CA)	4.68	SER291(OG)	1YY9A		
1YY9A	CYS287(C)	4.47	SER291(OG)	1YY9A		C[ ] ... O[ ]
1YY9A	CYS287(CB)	4.58	SER291(C)	1YY9A		
1YY9A	CYS287(CB)	4.49	SER291(O)	1YY9A		C[ ] ... O[ ]
1YY9A	CYS287(CB)	3.99	SER291(OG)	1YY9A		C[ ] ... O[ ]
1YY9A	CYS287(SG)	4.49	SER291(OG)	1YY9A	[D-A-AA]:62.5° [A-D-DD]:128.8°, maximum distance exceeded	S[ ] ... O[ ]
1YY9A	CYS287(CB)	4.48	TYR292(N)	1YY9A		C[ ] ... N[ ]
1YY9A	CYS287(CB)	4.40	TYR292(CA)	1YY9A		C[ ] ... C[ ]
1YY9A	CYS287(CB)	4.38	TYR292(C)	1YY9A		C[ ] ... C[ ]
1YY9A	CYS287(CB)	4.76	TYR292(O)	1YY9A		
1YY9A	CYS287(SG)	4.59	TYR292(N)	1YY9A	[D-A-AA]:75.1° [A-D-DD]:73.4° d_planarity:-79.4°, maximum distance exceeded, bad d_angle(sp2), bad d_planarity	
1YY9A	CYS287(SG)	4.40	TYR292(CA)	1YY9A		S[ ] ... C[ ]
1YY9A	CYS287(SG)	3.80	TYR292(C)	1YY9A		S[ ] ... C[ ]
1YY9A	CYS287(SG)	3.83	TYR292(O)	1YY9A		S[ ] ... O[ ]
1YY9A	CYS287(CB)	4.57	GLU293(N)	1YY9A		
1YY9A	CYS287(SG)	3.95	GLU293(N)	1YY9A	[D-A-AA]:98.7° [A-D-DD]:86.7° d_planarity:68.6°, bad d_angle(sp2), bad d_planarity	S[ ] ... N[ ]
1YY9A	CYS287(SG)	4.13	GLU293(CA)	1YY9A		S[ ] ... C[ ]
1YY9A	CYS287(SG)	4.27	GLU293(CB)	1YY9A	Negative	S[ ] ... C[ ]
1YY9A	CYS287(SG)	4.84	GLU293(CG)	1YY9A		
1YY9A	CYS287(SG)	4.47	LYS301(C)	1YY9A		S[ ] ... C[ ]
1YY9A	CYS287(SG)	4.36	LYS301(O)	1YY9A		S[ ] ... O[ ]
1YY9A	CYS287(N)	4.12	CYS302(SG)	1YY9A	[D-A-AA]:152.3° [A-D-DD]:48.3° d_planarity:-80.5°, maximum distance exceeded, bad d_angle(sp2), bad d_planarity	N[ ] ... S[ ]
1YY9A	CYS287(CA)	4.81	CYS302(CB)	1YY9A		
1YY9A	CYS287(CA)	3.32	CYS302(SG)	1YY9A		C[ ] ... S[ ]
1YY9A	CYS287(C)	4.42	CYS302(SG)	1YY9A		C[ ] ... S[ ]

1YY9A	CYS287(CB)	4.86	CYS302(CA)	1YY9A		
1YY9A	CYS287(CB)	4.16	CYS302(CB)	1YY9A		C[ ] ... C[ ]
1YY9A	CYS287(CB)	3.04	CYS302(SG)	1YY9A		C[ ] ... S[ ]
1YY9A	CYS287(SG)	4.00	CYS302(N)	1YY9A	[D-A-AA]:151.4° [A-D-DD]:58.5° d_planarity:85.4°, bad d_angle(sp2), bad d_planarity	S[ ] ... N[ ]
1YY9A	CYS287(SG)	3.47	CYS302(CA)	1YY9A		S[ ] ... C[ ]
1YY9A	CYS287(SG)	4.82	CYS302(C)	1YY9A		
1YY9A	CYS287(SG)	3.12	CYS302(CB)	1YY9A		S[ ] ... C[ ]
1YY9A	CYS287(SG)	2.05	CYS302(SG)	1YY9A	Disulphide bridge?	S[ ] ... S[ ]

chain1	res1/atm1	distance	res2/atm2	chain2	H-bonding	Charge interaction
1YY9A	CYS446(N)	4.49	THR422(CA)	1YY9A		N[ ] ... C[ ]
1YY9A	CYS446(N)	3.78	THR422(C)	1YY9A		N[ ] ... C[ ]
1YY9A	CYS446(N)	2.68	THR422(O)	1YY9A	**H-bond** [D-A-AA]:147.2° [A-D-DD]:113.9° d_planarity:-2.2° a_planarity:-58.8°	N[ ] ... O[ ]
1YY9A	CYS446(CA)	4.75	THR422(C)	1YY9A		
1YY9A	CYS446(CA)	3.53	THR422(O)	1YY9A		C[ ] ... O[ ]
1YY9A	CYS446(C)	4.94	THR422(C)	1YY9A		
1YY9A	CYS446(C)	3.78	THR422(O)	1YY9A		C[ ] ... O[ ]
1YY9A	CYS446(O)	4.40	THR422(C)	1YY9A		O[ ] ... C[ ]
1YY9A	CYS446(O)	3.39	THR422(O)	1YY9A		O[ ] ... O[ ]
1YY9A	CYS446(CB)	4.86	THR422(C)	1YY9A		
1YY9A	CYS446(CB)	3.67	THR422(O)	1YY9A		C[ ] ... O[ ]
1YY9A	CYS446(SG)	4.58	THR422(O)	1YY9A		
1YY9A	CYS446(N)	4.77	SER423(N)	1YY9A		
1YY9A	CYS446(N)	4.91	SER423(CA)	1YY9A		
1YY9A	CYS446(O)	4.75	SER423(N)	1YY9A	[D-A-AA]:122.6° [A-D-DD]:55.1° d_planarity:11.9° a_planarity:-50.8°, maximum distance exceeded, bad d_angle(sp2)	
1YY9A	CYS446(O)	4.10	SER423(CA)	1YY9A		O[ ] ... C[ ]
1YY9A	CYS446(O)	4.78	SER423(C)	1YY9A		
1YY9A	CYS446(O)	4.88	SER423(CB)	1YY9A		
1YY9A	CYS446(O)	4.68	LEU424(N)	1YY9A	[D-A-AA]:160.4° [A-D-DD]:138.5° d_planarity:-59.4° a_planarity:84.9°, maximum distance exceeded	
1YY9A	CYS446(N)	3.77	ASN444(C)	1YY9A		N[ ] ... C[ ]
1YY9A	CYS446(N)	3.31	ASN444(O)	1YY9A	[D-A-AA]:102.0° [A-D-DD]:133.9° d_planarity:89.3° a_planarity:16.2°, bad d_planarity	N[ ] ... O[ ]
1YY9A	CYS446(CA)	4.97	ASN444(C)	1YY9A		
1YY9A	CYS446(CA)	4.45	ASN444(O)	1YY9A		C[ ] ... O[ ]
1YY9A	CYS446(CB)	4.53	ASN444(O)	1YY9A		
1YY9A	CYS446(SG)	4.78	ASN444(C)	1YY9A		
1YY9A	CYS446(SG)	4.03	ASN444(O)	1YY9A		S[ ] ... O[ ]
1YY9A	CYS446(N)	3.43	LEU445(N)	1YY9A		N[ ] ... N[ ]
1YY9A	CYS446(N)	2.40	LEU445(CA)	1YY9A		N[ ] ... C[ ]
1YY9A	CYS446(N)	2.26	LEU445(O)	1YY9A	[D-A-AA]:29.4° [A-D-DD]:93.2° d_planarity:2.6° a_planarity:1.1°, bad a_angle(sp2)	N[ ] ... O[ ]
1YY9A	CYS446(N)	3.39	LEU445(CB)	1YY9A		N[ ] ... C[ ]
1YY9A	CYS446(N)	3.32	LEU445(CG)	1YY9A		N[ ] ... C[ ]

1YY9A	CYS446(N)	3.26	LEU445(CD1)	1YY9A		N[ ] ... C[ ]
1YY9A	CYS446(N)	4.79	LEU445(CD2)	1YY9A		
1YY9A	CYS446(CA)	4.70	LEU445(N)	1YY9A		
1YY9A	CYS446(CA)	3.77	LEU445(CA)	1YY9A		C[ ] ... C[ ]
1YY9A	CYS446(CA)	2.41	LEU445(C)	1YY9A		C[ ] ... C[ ]
1YY9A	CYS446(CA)	2.75	LEU445(O)	1YY9A		C[ ] ... O[ ]
1YY9A	CYS446(CA)	4.63	LEU445(CB)	1YY9A		
1YY9A	CYS446(CA)	4.32	LEU445(CG)	1YY9A		C[ ] ... C[ ]
1YY9A	CYS446(CA)	4.31	LEU445(CD1)	1YY9A		C[ ] ... C[ ]
1YY9A	CYS446(C)	4.57	LEU445(CA)	1YY9A		
1YY9A	CYS446(C)	3.40	LEU445(C)	1YY9A		C[ ] ... C[ ]
1YY9A	CYS446(C)	3.80	LEU445(O)	1YY9A		C[ ] ... O[ ]
1YY9A	CYS446(C)	4.31	LEU445(CG)	1YY9A		C[ ] ... C[ ]
1YY9A	CYS446(C)	4.12	LEU445(CD1)	1YY9A		C[ ] ... C[ ]
1YY9A	CYS446(O)	4.37	LEU445(CA)	1YY9A		O[ ] ... C[ ]
1YY9A	CYS446(O)	3.53	LEU445(C)	1YY9A		O[ ] ... C[ ]
1YY9A	CYS446(O)	4.17	LEU445(O)	1YY9A		O[ ] ... O[ ]
1YY9A	CYS446(O)	4.63	LEU445(CB)	1YY9A		
1YY9A	CYS446(O)	3.61	LEU445(CG)	1YY9A		O[ ] ... C[ ]
1YY9A	CYS446(O)	3.18	LEU445(CD1)	1YY9A		O[ ] ... C[ ]
1YY9A	CYS446(O)	4.61	LEU445(CD2)	1YY9A		
1YY9A	CYS446(CB)	4.72	LEU445(CA)	1YY9A		
1YY9A	CYS446(CB)	3.49	LEU445(C)	1YY9A		C[ ] ... C[ ]
1YY9A	CYS446(CB)	3.86	LEU445(O)	1YY9A		C[ ] ... O[ ]
1YY9A	CYS446(SG)	4.95	LEU445(CA)	1YY9A		
1YY9A	CYS446(SG)	3.84	LEU445(C)	1YY9A		S[ ] ... C[ ]
1YY9A	CYS446(SG)	3.96	LEU445(O)	1YY9A		S[ ] ... O[ ]
1YY9A	CYS446(N)	3.61	TYR447(N)	1YY9A		N[ ] ... N[ ]
1YY9A	CYS446(N)	4.88	TYR447(CA)	1YY9A		
1YY9A	CYS446(N)	4.39	TYR447(CD1)	1YY9A		N[ ] ... C[ ]
1YY9A	CYS446(N)	4.92	TYR447(CE1)	1YY9A		
1YY9A	CYS446(CA)	2.38	TYR447(N)	1YY9A		C[ ] ... N[ ]
1YY9A	CYS446(CA)	3.78	TYR447(CA)	1YY9A		C[ ] ... C[ ]
1YY9A	CYS446(CA)	4.68	TYR447(C)	1YY9A		
1YY9A	CYS446(CA)	4.33	TYR447(CB)	1YY9A		C[ ] ... C[ ]
1YY9A	CYS446(CA)	4.26	TYR447(CG)	1YY9A		C[ ] ... C[ ]
1YY9A	CYS446(CA)	3.87	TYR447(CD1)	1YY9A		C[ ] ... C[ ]
1YY9A	CYS446(CA)	4.49	TYR447(CE1)	1YY9A		C[ ] ... C[ ]
1YY9A	CYS446(C)	2.49	TYR447(CA)	1YY9A		C[ ] ... C[ ]
1YY9A	CYS446(C)	3.32	TYR447(C)	1YY9A		C[ ] ... C[ ]
1YY9A	CYS446(C)	4.53	TYR447(O)	1YY9A		
1YY9A	CYS446(C)	3.01	TYR447(CB)	1YY9A		C[ ] ... C[ ]
1YY9A	CYS446(C)	3.32	TYR447(CG)	1YY9A		C[ ] ... C[ ]
1YY9A	CYS446(C)	3.23	TYR447(CD1)	1YY9A		C[ ] ... C[ ]
1YY9A	CYS446(C)	4.41	TYR447(CD2)	1YY9A		C[ ] ... C[ ]
1YY9A	CYS446(C)	4.25	TYR447(CE1)	1YY9A		C[ ] ... C[ ]
1YY9A	CYS446(O)	2.26	TYR447(N)	1YY9A	[D-A-AA]:28.7° [A-D-DD]:100.0° d_planarity:6.4° a_planarity:0.7°, bad a_angle(sp2)	O[ ] ... N[ ]
1YY9A	CYS446(O)	2.90	TYR447(CA)	1YY9A		O[ ] ... C[ ]

1YY9A	CYS446(O)	3.43	TYR447(C)	1YY9A		O[ ] ... C[ ]
1YY9A	CYS446(O)	4.63	TYR447(O)	1YY9A		
1YY9A	CYS446(O)	3.01	TYR447(CB)	1YY9A		O[ ] ... C[ ]
1YY9A	CYS446(O)	3.52	TYR447(CG)	1YY9A		O[ ] ... C[ ]
1YY9A	CYS446(O)	3.28	TYR447(CD1)	1YY9A		O[ ] ... C[ ]
1YY9A	CYS446(O)	4.82	TYR447(CD2)	1YY9A		
1YY9A	CYS446(O)	4.43	TYR447(CE1)	1YY9A		O[ ] ... C[ ]
1YY9A	CYS446(CB)	2.90	TYR447(N)	1YY9A		C[ ] ... N[ ]
1YY9A	CYS446(CB)	4.17	TYR447(CA)	1YY9A		C[ ] ... C[ ]
1YY9A	CYS446(CB)	4.51	TYR447(CB)	1YY9A		
1YY9A	CYS446(CB)	3.94	TYR447(CG)	1YY9A		C[ ] ... C[ ]
1YY9A	CYS446(CB)	3.44	TYR447(CD1)	1YY9A		C[ ] ... C[ ]
1YY9A	CYS446(CB)	4.53	TYR447(CD2)	1YY9A		
1YY9A	CYS446(CB)	3.64	TYR447(CE1)	1YY9A		C[ ] ... C[ ]
1YY9A	CYS446(CB)	4.69	TYR447(CE2)	1YY9A		
1YY9A	CYS446(CB)	4.28	TYR447(CZ)	1YY9A		C[ ] ... C[ ]
1YY9A	CYS446(SG)	4.52	TYR447(N)	1YY9A	[D-A-AA]:19.9° [A-D-DD]:151.0° d_planarity:-80.9°, maximum distance exceeded, bad a_angle(sp3), bad d_planarity	
1YY9A	CYS446(SG)	4.86	TYR447(CE1)	1YY9A		
1YY9A	CYS446(CA)	4.44	ALA448(N)	1YY9A		C[ ] ... N[ ]
1YY9A	CYS446(C)	3.15	ALA448(N)	1YY9A		C[ ] ... N[ ]
1YY9A	CYS446(C)	4.48	ALA448(CA)	1YY9A		C[ ] ... C[ ]
1YY9A	CYS446(C)	4.77	ALA448(CB)	1YY9A		
1YY9A	CYS446(O)	3.06	ALA448(N)	1YY9A	[D-A-AA]:83.1° [A-D-DD]:132.5° d_planarity:-46.5° a_planarity:37.6°, bad a_angle(sp2)	O[ ] ... N[ ]
1YY9A	CYS446(O)	4.18	ALA448(CA)	1YY9A		O[ ] ... C[ ]
1YY9A	CYS446(O)	4.35	ALA448(CB)	1YY9A		O[ ] ... C[ ]
1YY9A	CYS446(SG)	4.38	ARG470(CB)	1YY9A		S[ ] ... C[ ]
1YY9A	CYS446(SG)	4.12	ARG470(CG)	1YY9A		S[ ] ... C[ ]
1YY9A	CYS446(SG)	3.96	ARG470(CD)	1YY9A	Positive	S[ ] ... C[ ]
1YY9A	CYS446(N)	4.48	CYS475(SG)	1YY9A	[D-A-AA]:135.6° [A-D-DD]:46.9° d_planarity:61.7°, maximum distance exceeded, bad d_angle(sp2), bad d_planarity	N[ ] ... S[ ]
1YY9A	CYS446(CA)	4.88	CYS475(CB)	1YY9A		
1YY9A	CYS446(CA)	3.64	CYS475(SG)	1YY9A		C[ ] ... S[ ]
1YY9A	CYS446(C)	4.82	CYS475(SG)	1YY9A		
1YY9A	CYS446(CB)	3.94	CYS475(CB)	1YY9A		C[ ] ... C[ ]
1YY9A	CYS446(CB)	3.07	CYS475(SG)	1YY9A		C[ ] ... S[ ]
1YY9A	CYS446(SG)	4.73	CYS475(N)	1YY9A	[D-A-AA]:130.2° [A-D-DD]:50.7° d_planarity:-51.3°, maximum distance exceeded, bad d_angle(sp2)	
1YY9A	CYS446(SG)	3.97	CYS475(CA)	1YY9A		S[ ] ... C[ ]
1YY9A	CYS446(SG)	3.14	CYS475(CB)	1YY9A		S[ ] ... C[ ]
1YY9A	CYS446(SG)	2.07	CYS475(SG)	1YY9A	Disulphide bridge	S[ ] ... S[ ]
1YY9A	CYS446(CB)	4.09	GLN480(O)	1YY9A		C[ ] ... O[ ]
1YY9A	CYS446(CB)	4.56	GLN480(CB)	1YY9A		
1YY9A	CYS446(SG)	4.34	GLN480(O)	1YY9A		S[ ] ... O[ ]
1YY9A	CYS446(SG)	4.07	GLN480(CB)	1YY9A		S[ ] ... C[ ]
1YY9A	CYS446(SG)	4.86	GLN480(CG)	1YY9A		
1YY9A	CYS446(SG)	4.32	GLN480(CD)	1YY9A		S[ ] ... C[ ]
1YY9A	CYS446(SG)	3.77	GLN480(OE1)	1YY9A		S[ ] ... O[ ]

1YY9A	CYS446(SG)	4.97	GLN480(NE2)	1YY9A	[D-A-AA]:109.1° [A-D-DD]:54.1° d_planarity:62.4°, maximum distance exceeded, bad d_angle(sp2), bad d_planarity	

chain1	res1/atm1	distance	res2/atm2	chain2	H-bonding	Charge interaction
1Z2BA	CYS295(N)	4.91	THR271(CG2)	1Z2BA		
1Z2BA	CYS295(CA)	4.63	THR271(CB)	1Z2BA		
1Z2BA	CYS295(CA)	3.79	THR271(CG2)	1Z2BA		C[ ] ... C[ ]
1Z2BA	CYS295(C)	4.77	THR271(CB)	1Z2BA		
1Z2BA	CYS295(C)	4.99	THR271(OG1)	1Z2BA		
1Z2BA	CYS295(C)	3.89	THR271(CG2)	1Z2BA		C[ ] ... C[ ]
1Z2BA	CYS295(O)	4.02	THR271(CB)	1Z2BA		O[ ] ... C[ ]
1Z2BA	CYS295(O)	3.99	THR271(OG1)	1Z2BA	[D-A-AA]:139.1° [A-D-DD]:81.1° a_planarity:-19.8°, maximum distance exceeded	O[ ] ... O[ ]
1Z2BA	CYS295(O)	3.20	THR271(CG2)	1Z2BA		O[ ] ... C[ ]
1Z2BA	CYS295(CB)	4.90	THR271(CB)	1Z2BA		
1Z2BA	CYS295(CB)	4.49	THR271(CG2)	1Z2BA		C[ ] ... C[ ]
1Z2BA	CYS295(SG)	4.61	THR271(O)	1Z2BA		
1Z2BA	CYS295(SG)	4.56	THR271(CB)	1Z2BA		
1Z2BA	CYS295(SG)	4.48	THR271(CG2)	1Z2BA		S[ ] ... C[ ]
1Z2BA	CYS295(SG)	4.98	ALA273(CA)	1Z2BA		
1Z2BA	CYS295(N)	4.57	ILE291(C)	1Z2BA		
1Z2BA	CYS295(N)	3.49	ILE291(O)	1Z2BA	**H-bond** [D-A-AA]:146.0° [A-D-DD]:118.2° d_planarity:-36.7° a_planarity:51.5°	N[ ] ... O[ ]
1Z2BA	CYS295(CA)	4.37	ILE291(O)	1Z2BA		C[ ] ... O[ ]
1Z2BA	CYS295(CB)	4.24	ILE291(O)	1Z2BA		C[ ] ... O[ ]
1Z2BA	CYS295(SG)	4.95	ILE291(C)	1Z2BA		
1Z2BA	CYS295(SG)	4.02	ILE291(O)	1Z2BA		S[ ] ... O[ ]
1Z2BA	CYS295(N)	4.59	THR292(CA)	1Z2BA		
1Z2BA	CYS295(N)	3.60	THR292(C)	1Z2BA		N[ ] ... C[ ]
1Z2BA	CYS295(N)	2.71	THR292(O)	1Z2BA	**H-bond** [D-A-AA]:128.3° [A-D-DD]:99.4° d_planarity:-25.3° a_planarity:78.4°	N[ ] ... O[ ]
1Z2BA	CYS295(CA)	4.37	THR292(C)	1Z2BA		C[ ] ... C[ ]
1Z2BA	CYS295(CA)	3.28	THR292(O)	1Z2BA		C[ ] ... O[ ]
1Z2BA	CYS295(C)	3.97	THR292(O)	1Z2BA		C[ ] ... O[ ]
1Z2BA	CYS295(CB)	4.37	THR292(CA)	1Z2BA		C[ ] ... C[ ]
1Z2BA	CYS295(CB)	3.87	THR292(C)	1Z2BA		C[ ] ... C[ ]
1Z2BA	CYS295(CB)	2.79	THR292(O)	1Z2BA		C[ ] ... O[ ]
1Z2BA	CYS295(SG)	4.51	THR292(CA)	1Z2BA		
1Z2BA	CYS295(SG)	4.56	THR292(C)	1Z2BA		
1Z2BA	CYS295(SG)	3.81	THR292(O)	1Z2BA		S[ ] ... O[ ]
1Z2BA	CYS295(N)	4.23	ASN293(N)	1Z2BA		N[ ] ... N[ ]
1Z2BA	CYS295(N)	4.19	ASN293(CA)	1Z2BA		N[ ] ... C[ ]
1Z2BA	CYS295(N)	3.25	ASN293(C)	1Z2BA		N[ ] ... C[ ]
1Z2BA	CYS295(N)	3.70	ASN293(O)	1Z2BA	[D-A-AA]:59.7° [A-D-DD]:135.3° d_planarity:79.3° a_planarity:47.3°, maximum distance exceeded, bad a_angle(sp2), bad d_planarity	N[ ] ... O[ ]

1Z2BA	CYS295(CA)	4.52	ASN293(C)	1Z2BA		
1Z2BA	CYS295(CA)	4.84	ASN293(O)	1Z2BA		
1Z2BA	CYS295(C)	4.86	ASN293(C)	1Z2BA		
1Z2BA	CYS295(C)	4.84	ASN293(O)	1Z2BA		
1Z2BA	CYS295(N)	2.60	ALA294(N)	1Z2BA		N[ ] ... N[ ]
1Z2BA	CYS295(N)	2.38	ALA294(CA)	1Z2BA		N[ ] ... C[ ]
1Z2BA	CYS295(N)	2.26	ALA294(O)	1Z2BA	[D-A-AA]:28.8° [A-D-DD]:97.9° d_planarity:3.0° a_planarity:0.0°, bad a_angle(sp2)	N[ ] ... O[ ]
1Z2BA	CYS295(N)	3.13	ALA294(CB)	1Z2BA		N[ ] ... C[ ]
1Z2BA	CYS295(CA)	4.03	ALA294(N)	1Z2BA		C[ ] ... N[ ]
1Z2BA	CYS295(CA)	3.77	ALA294(CA)	1Z2BA		C[ ] ... C[ ]
1Z2BA	CYS295(CA)	2.46	ALA294(C)	1Z2BA		C[ ] ... C[ ]
1Z2BA	CYS295(CA)	2.85	ALA294(O)	1Z2BA		C[ ] ... O[ ]
1Z2BA	CYS295(CA)	4.39	ALA294(CB)	1Z2BA		C[ ] ... C[ ]
1Z2BA	CYS295(C)	4.71	ALA294(N)	1Z2BA		
1Z2BA	CYS295(C)	4.41	ALA294(CA)	1Z2BA		C[ ] ... C[ ]
1Z2BA	CYS295(C)	3.00	ALA294(C)	1Z2BA		C[ ] ... C[ ]
1Z2BA	CYS295(C)	2.95	ALA294(O)	1Z2BA		C[ ] ... O[ ]
1Z2BA	CYS295(O)	3.94	ALA294(C)	1Z2BA		O[ ] ... C[ ]
1Z2BA	CYS295(O)	3.58	ALA294(O)	1Z2BA		O[ ] ... O[ ]
1Z2BA	CYS295(CB)	4.61	ALA294(N)	1Z2BA		
1Z2BA	CYS295(CB)	4.76	ALA294(CA)	1Z2BA		
1Z2BA	CYS295(CB)	3.69	ALA294(C)	1Z2BA		C[ ] ... C[ ]
1Z2BA	CYS295(CB)	4.30	ALA294(O)	1Z2BA		C[ ] ... O[ ]
1Z2BA	CYS295(SG)	4.68	ALA294(C)	1Z2BA		
1Z2BA	CYS295(N)	2.66	PHE296(N)	1Z2BA		N[ ] ... N[ ]
1Z2BA	CYS295(N)	4.03	PHE296(CA)	1Z2BA		N[ ] ... C[ ]
1Z2BA	CYS295(N)	4.40	PHE296(C)	1Z2BA		N[ ] ... C[ ]
1Z2BA	CYS295(N)	4.88	PHE296(CB)	1Z2BA		
1Z2BA	CYS295(CA)	2.32	PHE296(N)	1Z2BA		C[ ] ... N[ ]
1Z2BA	CYS295(CA)	3.69	PHE296(CA)	1Z2BA		C[ ] ... C[ ]
1Z2BA	CYS295(CA)	4.38	PHE296(C)	1Z2BA		C[ ] ... C[ ]
1Z2BA	CYS295(CA)	4.65	PHE296(CB)	1Z2BA		
1Z2BA	CYS295(CA)	4.74	PHE296(CG)	1Z2BA		
1Z2BA	CYS295(C)	2.40	PHE296(CA)	1Z2BA		C[ ] ... C[ ]
1Z2BA	CYS295(C)	3.20	PHE296(C)	1Z2BA		C[ ] ... C[ ]
1Z2BA	CYS295(C)	4.27	PHE296(O)	1Z2BA		C[ ] ... O[ ]
1Z2BA	CYS295(C)	3.57	PHE296(CB)	1Z2BA		C[ ] ... C[ ]
1Z2BA	CYS295(C)	3.83	PHE296(CG)	1Z2BA		C[ ] ... C[ ]
1Z2BA	CYS295(C)	4.51	PHE296(CD1)	1Z2BA		
1Z2BA	CYS295(C)	4.11	PHE296(CD2)	1Z2BA		C[ ] ... C[ ]
1Z2BA	CYS295(C)	4.98	PHE296(CE2)	1Z2BA		
1Z2BA	CYS295(O)	2.26	PHE296(N)	1Z2BA	[D-A-AA]:28.4° [A-D-DD]:95.4° d_planarity:1.8° a_planarity:3.1°, bad a_angle(sp2)	O[ ] ... N[ ]
1Z2BA	CYS295(O)	2.79	PHE296(CA)	1Z2BA		O[ ] ... C[ ]
1Z2BA	CYS295(O)	3.52	PHE296(C)	1Z2BA		O[ ] ... C[ ]
1Z2BA	CYS295(O)	4.73	PHE296(O)	1Z2BA		
1Z2BA	CYS295(O)	4.08	PHE296(CB)	1Z2BA		O[ ] ... C[ ]
1Z2BA	CYS295(O)	4.28	PHE296(CG)	1Z2BA		O[ ] ... C[ ]

1Z2BA	CYS295(O)	4.19	PHE296(CD2)	1Z2BA		O[ ] ... C[ ]
1Z2BA	CYS295(CB)	2.94	PHE296(N)	1Z2BA		C[ ] ... N[ ]
1Z2BA	CYS295(CB)	4.24	PHE296(CA)	1Z2BA		C[ ] ... C[ ]
1Z2BA	CYS295(CB)	4.80	PHE296(CB)	1Z2BA		
1Z2BA	CYS295(CB)	4.42	PHE296(CG)	1Z2BA		C[ ] ... C[ ]
1Z2BA	CYS295(CB)	4.42	PHE296(CD1)	1Z2BA		C[ ] ... C[ ]
1Z2BA	CYS295(CB)	4.74	PHE296(CD2)	1Z2BA		
1Z2BA	CYS295(CB)	4.73	PHE296(CE1)	1Z2BA		
1Z2BA	CYS295(SG)	4.65	PHE296(N)	1Z2BA	[D-A-AA]:11.4° [A-D-DD]:152.2° d_planarity:55.3°, maximum distance exceeded, bad a_angle(sp3)	
1Z2BA	CYS295(N)	4.49	GLU297(N)	1Z2BA		N[ ] ... N[ ]
1Z2BA	CYS295(N)	4.70	GLU297(CB)	1Z2BA		
1Z2BA	CYS295(CA)	4.35	GLU297(N)	1Z2BA		C[ ] ... N[ ]
1Z2BA	CYS295(C)	3.18	GLU297(N)	1Z2BA		C[ ] ... N[ ]
1Z2BA	CYS295(C)	4.40	GLU297(CA)	1Z2BA		C[ ] ... C[ ]
1Z2BA	CYS295(C)	4.93	GLU297(C)	1Z2BA		
1Z2BA	CYS295(C)	4.50	GLU297(O)	1Z2BA		C[ ] ... O[ ]
1Z2BA	CYS295(C)	4.55	GLU297(CB)	1Z2BA		
1Z2BA	CYS295(O)	3.18	GLU297(N)	1Z2BA	[D-A-AA]:79.0° [A-D-DD]:140.2° d_planarity:-26.9° a_planarity:43.9°, bad a_angle(sp2)	O[ ] ... N[ ]
1Z2BA	CYS295(O)	4.37	GLU297(CA)	1Z2BA		O[ ] ... C[ ]
1Z2BA	CYS295(O)	4.53	GLU297(C)	1Z2BA		
1Z2BA	CYS295(O)	3.91	GLU297(O)	1Z2BA		O[ ] ... O[ ]
1Z2BA	CYS295(O)	4.79	GLU297(CB)	1Z2BA		
1Z2BA	CYS295(O)	4.80	GLN301(NE2)	1Z2BA	[D-A-AA]:145.8° [A-D-DD]:117.2° d_planarity:-9.2° a_planarity:39.0°, maximum distance exceeded	
1Z2BA	CYS295(CB)	4.83	LEU317(CD2)	1Z2BA		
1Z2BA	CYS295(SG)	4.44	LEU317(CD2)	1Z2BA		S[ ] ... C[ ]
1Z2BA	CYS295(SG)	4.80	VAL375(C)	1Z2BA		
1Z2BA	CYS295(SG)	3.94	VAL375(CB)	1Z2BA		S[ ] ... C[ ]
1Z2BA	CYS295(SG)	4.02	VAL375(CG1)	1Z2BA		S[ ] ... C[ ]
1Z2BA	CYS295(SG)	4.71	VAL375(CG2)	1Z2BA		
1Z2BA	CYS295(SG)	4.66	CYS376(N)	1Z2BA	[D-A-AA]:165.6° [A-D-DD]:98.4° d_planarity:-88.0°, maximum distance exceeded, bad d_planarity	
1Z2BA	CYS295(SG)	4.72	CYS376(C)	1Z2BA		
1Z2BA	CYS295(C)	4.99	MET377(CG)	1Z2BA		
1Z2BA	CYS295(C)	4.94	MET377(SD)	1Z2BA		
1Z2BA	CYS295(O)	4.54	MET377(CG)	1Z2BA		
1Z2BA	CYS295(O)	4.75	MET377(SD)	1Z2BA		
1Z2BA	CYS295(CB)	4.19	MET377(CB)	1Z2BA		C[ ] ... C[ ]
1Z2BA	CYS295(CB)	4.32	MET377(CG)	1Z2BA		C[ ] ... C[ ]
1Z2BA	CYS295(CB)	4.13	MET377(SD)	1Z2BA		C[ ] ... S[ ]
1Z2BA	CYS295(SG)	4.61	MET377(N)	1Z2BA	[D-A-AA]:130.5° [A-D-DD]:82.6° d_planarity:81.6°, maximum distance exceeded, bad d_angle(sp2), bad d_planarity	
1Z2BA	CYS295(SG)	4.65	MET377(CA)	1Z2BA		
1Z2BA	CYS295(SG)	3.48	MET377(CB)	1Z2BA		S[ ] ... C[ ]
1Z2BA	CYS295(SG)	4.21	MET377(CG)	1Z2BA		S[ ] ... C[ ]
1Z2BA	CYS295(SG)	4.45	MET377(SD)	1Z2BA		S[ ] ... S[ ]

chain1	res1/atm1	distance	res2/atm2	chain2	H-bonding	Charge interaction
1Z2BA	CYS347(CB)	4.88	MET313(CA)	1Z2BA		
1Z2BA	CYS347(CB)	4.84	MET313(C)	1Z2BA		
1Z2BA	CYS347(CB)	4.23	MET313(O)	1Z2BA		C[ ] ... O[ ]
1Z2BA	CYS347(SG)	4.66	MET313(CA)	1Z2BA		
1Z2BA	CYS347(SG)	4.37	MET313(C)	1Z2BA		S[ ] ... C[ ]
1Z2BA	CYS347(SG)	3.43	MET313(O)	1Z2BA		S[ ] ... O[ ]
1Z2BA	CYS347(SG)	4.78	MET313(CB)	1Z2BA		
1Z2BA	CYS347(CA)	4.72	PHE343(CD2)	1Z2BA		
1Z2BA	CYS347(CA)	4.84	PHE343(CE2)	1Z2BA		
1Z2BA	CYS347(C)	4.25	PHE343(CD2)	1Z2BA		C[ ] ... C[ ]
1Z2BA	CYS347(C)	4.50	PHE343(CE2)	1Z2BA		C[ ] ... C[ ]
1Z2BA	CYS347(O)	4.46	PHE343(CB)	1Z2BA		O[ ] ... C[ ]
1Z2BA	CYS347(O)	4.27	PHE343(CG)	1Z2BA		O[ ] ... C[ ]
1Z2BA	CYS347(O)	3.60	PHE343(CD2)	1Z2BA		O[ ] ... C[ ]
1Z2BA	CYS347(O)	4.20	PHE343(CE2)	1Z2BA		O[ ] ... C[ ]
1Z2BA	CYS347(CB)	3.87	PHE343(CD2)	1Z2BA		C[ ] ... C[ ]
1Z2BA	CYS347(CB)	3.84	PHE343(CE2)	1Z2BA		C[ ] ... C[ ]
1Z2BA	CYS347(SG)	4.64	PHE343(CE2)	1Z2BA		
1Z2BA	CYS347(N)	4.62	VAL344(C)	1Z2BA		
1Z2BA	CYS347(N)	3.46	VAL344(O)	1Z2BA	[D-A-AA]:157.7° [A-D-DD]:111.7° d_planarity:-79.9° a_planarity:36.9°, bad d_planarity	N[ ] ... O[ ]
1Z2BA	CYS347(CA)	4.22	VAL344(O)	1Z2BA		C[ ] ... O[ ]
1Z2BA	CYS347(C)	4.77	VAL344(O)	1Z2BA		
1Z2BA	CYS347(O)	4.28	VAL344(O)	1Z2BA		O[ ] ... O[ ]
1Z2BA	CYS347(CB)	3.86	VAL344(O)	1Z2BA		C[ ] ... O[ ]
1Z2BA	CYS347(CB)	4.84	VAL344(CG1)	1Z2BA		
1Z2BA	CYS347(N)	3.90	ASP345(C)	1Z2BA		N[ ] ... C[ ]
1Z2BA	CYS347(N)	4.21	ASP345(O)	1Z2BA	[D-A-AA]:67.4° [A-D-DD]:161.2° d_planarity:-16.6° a_planarity:30.1°, maximum distance exceeded, bad a_angle(sp2)	N[ ] ... O[ ]
1Z2BA	CYS347(N)	3.00	TRP346(N)	1Z2BA		N[ ] ... N[ ]
1Z2BA	CYS347(N)	2.44	TRP346(CA)	1Z2BA		N[ ] ... C[ ]
1Z2BA	CYS347(N)	2.25	TRP346(O)	1Z2BA	[D-A-AA]:29.8° [A-D-DD]:95.1° d_planarity:2.2° a_planarity:1.1°, bad a_angle(sp2)	N[ ] ... O[ ]
1Z2BA	CYS347(N)	3.75	TRP346(CB)	1Z2BA		N[ ] ... C[ ]
1Z2BA	CYS347(N)	4.47	TRP346(CG)	1Z2BA		N[ ] ... C[ ]
1Z2BA	CYS347(N)	4.48	TRP346(CD2)	1Z2BA		N[ ] ... C[ ]
1Z2BA	CYS347(N)	3.97	TRP346(CE3)	1Z2BA		N[ ] ... C[ ]
1Z2BA	CYS347(N)	4.93	TRP346(CZ3)	1Z2BA		
1Z2BA	CYS347(CA)	4.35	TRP346(N)	1Z2BA		C[ ] ... N[ ]
1Z2BA	CYS347(CA)	3.82	TRP346(CA)	1Z2BA		C[ ] ... C[ ]
1Z2BA	CYS347(CA)	2.45	TRP346(C)	1Z2BA		C[ ] ... C[ ]
1Z2BA	CYS347(CA)	2.79	TRP346(O)	1Z2BA		C[ ] ... O[ ]
1Z2BA	CYS347(CA)	4.99	TRP346(CB)	1Z2BA		
1Z2BA	CYS347(CA)	4.36	TRP346(CE3)	1Z2BA		C[ ] ... C[ ]
1Z2BA	CYS347(CA)	4.99	TRP346(CZ3)	1Z2BA		
1Z2BA	CYS347(C)	4.82	TRP346(CA)	1Z2BA		
1Z2BA	CYS347(C)	3.69	TRP346(C)	1Z2BA		C[ ] ... C[ ]

1Z2BA	CYS347(C)	4.25	TRP346(O)	1Z2BA		C[ ] ... O[ ]
1Z2BA	CYS347(O)	4.16	TRP346(C)	1Z2BA		O[ ] ... C[ ]
1Z2BA	CYS347(O)	4.93	TRP346(O)	1Z2BA		
1Z2BA	CYS347(CB)	4.65	TRP346(N)	1Z2BA		
1Z2BA	CYS347(CB)	4.43	TRP346(CA)	1Z2BA		C[ ] ... C[ ]
1Z2BA	CYS347(CB)	3.07	TRP346(C)	1Z2BA		C[ ] ... C[ ]
1Z2BA	CYS347(CB)	3.14	TRP346(O)	1Z2BA		C[ ] ... O[ ]
1Z2BA	CYS347(CB)	4.94	TRP346(CD2)	1Z2BA		
1Z2BA	CYS347(CB)	3.94	TRP346(CE3)	1Z2BA		C[ ] ... C[ ]
1Z2BA	CYS347(CB)	4.21	TRP346(CZ3)	1Z2BA		C[ ] ... C[ ]
1Z2BA	CYS347(SG)	3.78	TRP346(C)	1Z2BA		S[ ] ... C[ ]
1Z2BA	CYS347(SG)	3.20	TRP346(O)	1Z2BA		S[ ] ... O[ ]
1Z2BA	CYS347(SG)	4.71	TRP346(CD2)	1Z2BA		
1Z2BA	CYS347(SG)	3.40	TRP346(CE3)	1Z2BA		S[ ] ... C[ ]
1Z2BA	CYS347(SG)	3.21	TRP346(CZ3)	1Z2BA		S[ ] ... C[ ]
1Z2BA	CYS347(SG)	4.43	TRP346(CH2)	1Z2BA		S[ ] ... C[ ]
1Z2BA	CYS347(N)	3.47	PRO348(N)	1Z2BA		N[ ] ... N[ ]
1Z2BA	CYS347(N)	4.64	PRO348(CA)	1Z2BA		
1Z2BA	CYS347(N)	4.16	PRO348(CD)	1Z2BA		N[ ] ... C[ ]
1Z2BA	CYS347(CA)	2.45	PRO348(N)	1Z2BA		C[ ] ... N[ ]
1Z2BA	CYS347(CA)	3.82	PRO348(CA)	1Z2BA		C[ ] ... C[ ]
1Z2BA	CYS347(CA)	4.39	PRO348(C)	1Z2BA		C[ ] ... C[ ]
1Z2BA	CYS347(CA)	4.63	PRO348(O)	1Z2BA		
1Z2BA	CYS347(CA)	4.74	PRO348(CB)	1Z2BA		
1Z2BA	CYS347(CA)	4.17	PRO348(CG)	1Z2BA		C[ ] ... C[ ]
1Z2BA	CYS347(CA)	2.97	PRO348(CD)	1Z2BA		C[ ] ... C[ ]
1Z2BA	CYS347(C)	2.44	PRO348(CA)	1Z2BA		C[ ] ... C[ ]
1Z2BA	CYS347(C)	2.87	PRO348(C)	1Z2BA		C[ ] ... C[ ]
1Z2BA	CYS347(C)	3.16	PRO348(O)	1Z2BA		C[ ] ... O[ ]
1Z2BA	CYS347(C)	3.56	PRO348(CB)	1Z2BA		C[ ] ... C[ ]
1Z2BA	CYS347(C)	3.39	PRO348(CG)	1Z2BA		C[ ] ... C[ ]
1Z2BA	CYS347(C)	2.50	PRO348(CD)	1Z2BA		C[ ] ... C[ ]
1Z2BA	CYS347(O)	2.24	PRO348(N)	1Z2BA		O[ ] ... N[ ]
1Z2BA	CYS347(O)	2.78	PRO348(CA)	1Z2BA		O[ ] ... C[ ]
1Z2BA	CYS347(O)	2.54	PRO348(C)	1Z2BA		O[ ] ... C[ ]
1Z2BA	CYS347(O)	2.43	PRO348(O)	1Z2BA		O[ ] ... O[ ]
1Z2BA	CYS347(O)	4.07	PRO348(CB)	1Z2BA		O[ ] ... C[ ]
1Z2BA	CYS347(O)	4.21	PRO348(CG)	1Z2BA		O[ ] ... C[ ]
1Z2BA	CYS347(O)	3.60	PRO348(CD)	1Z2BA		O[ ] ... C[ ]
1Z2BA	CYS347(CB)	3.45	PRO348(N)	1Z2BA		C[ ] ... N[ ]
1Z2BA	CYS347(CB)	4.77	PRO348(CA)	1Z2BA		
1Z2BA	CYS347(CB)	4.75	PRO348(CG)	1Z2BA		
1Z2BA	CYS347(CB)	3.84	PRO348(CD)	1Z2BA		C[ ] ... C[ ]
1Z2BA	CYS347(SG)	4.58	PRO348(N)	1Z2BA		
1Z2BA	CYS347(SG)	4.38	PRO348(CD)	1Z2BA		S[ ] ... C[ ]
1Z2BA	CYS347(C)	3.73	THR349(N)	1Z2BA		C[ ] ... N[ ]
1Z2BA	CYS347(C)	4.63	THR349(CA)	1Z2BA		
1Z2BA	CYS347(O)	3.41	THR349(N)	1Z2BA	**H-bond** [D-A-AA]:95.1° [A-D-DD]:104.7° d_planarity:37.1° a_planarity:24.7°	O[ ] ... N[ ]

1Z2BA	CYS347(O)	4.03	THR349(CA)	1Z2BA		O[ ] ... C[ ]
1Z2BA	CYS347(O)	4.96	THR349(CB)	1Z2BA		
1Z2BA	CYS347(O)	4.94	THR349(OG1)	1Z2BA	[D-A-AA]:119.4° [A-D-DD]:82.7° a_planarity:5.0°, maximum distance exceeded	
1Z2BA	CYS347(O)	4.94	GLY350(N)	1Z2BA	[D-A-AA]:87.7° [A-D-DD]:143.1° d_planarity:-11.4° a_planarity:52.2°, maximum distance exceeded, bad a_angle(sp2)	
1Z2BA	CYS347(N)	4.18	PRO27(CA)	1Z2BE		N[ ] ... C[ ]
1Z2BA	CYS347(N)	4.65	PRO27(C)	1Z2BE		
1Z2BA	CYS347(N)	4.32	PRO27(O)	1Z2BE	[D-A-AA]:97.5° [A-D-DD]:128.9° d_planarity:-34.6° a_planarity:-39.0°, maximum distance exceeded	N[ ] ... O[ ]
1Z2BA	CYS347(N)	3.56	PRO27(CB)	1Z2BE		N[ ] ... C[ ]
1Z2BA	CYS347(N)	4.75	PRO27(CG)	1Z2BE		
1Z2BA	CYS347(CA)	4.62	PRO27(CA)	1Z2BE		
1Z2BA	CYS347(CA)	3.87	PRO27(CB)	1Z2BE		C[ ] ... C[ ]
1Z2BA	CYS347(CA)	4.68	PRO27(CG)	1Z2BE		
1Z2BA	CYS347(C)	4.75	PRO27(N)	1Z2BE		
1Z2BA	CYS347(C)	4.01	PRO27(CA)	1Z2BE		C[ ] ... C[ ]
1Z2BA	CYS347(C)	3.62	PRO27(CB)	1Z2BE		C[ ] ... C[ ]
1Z2BA	CYS347(C)	4.28	PRO27(CG)	1Z2BE		C[ ] ... C[ ]
1Z2BA	CYS347(C)	4.78	PRO27(CD)	1Z2BE		
1Z2BA	CYS347(O)	4.93	PRO27(N)	1Z2BE		
1Z2BA	CYS347(O)	4.17	PRO27(CA)	1Z2BE		O[ ] ... C[ ]
1Z2BA	CYS347(O)	4.93	PRO27(O)	1Z2BE		
1Z2BA	CYS347(O)	4.21	PRO27(CB)	1Z2BE		O[ ] ... C[ ]

chain1	res1/atm1	distance	res2/atm2	chain2	H-bonding	Charge interaction
1Z2BA	CYS376(SG)	4.32	ILE234(O)	1Z2BA		S[ ] ... O[ ]
1Z2BA	CYS376(CB)	4.89	SER237(O)	1Z2BA		
1Z2BA	CYS376(CB)	4.36	SER237(CB)	1Z2BA		C[ ] ... C[ ]
1Z2BA	CYS376(CB)	4.87	SER237(OG)	1Z2BA		
1Z2BA	CYS376(SG)	4.59	SER237(CA)	1Z2BA		
1Z2BA	CYS376(SG)	4.36	SER237(C)	1Z2BA		S[ ] ... C[ ]
1Z2BA	CYS376(SG)	4.49	SER237(O)	1Z2BA		S[ ] ... O[ ]
1Z2BA	CYS376(SG)	3.57	SER237(CB)	1Z2BA		S[ ] ... C[ ]
1Z2BA	CYS376(SG)	4.46	SER237(OG)	1Z2BA	[D-A-AA]:92.3° [A-D-DD]:43.8°, maximum distance exceeded, bad d_angle(sp3)	S[ ] ... O[ ]
1Z2BA	CYS376(SG)	4.64	ILE238(N)	1Z2BA	[D-A-AA]:120.0° [A-D-DD]:102.6° d_planarity:73.5°, maximum distance exceeded, bad d_planarity	
1Z2BA	CYS376(SG)	4.38	ILE238(CG1)	1Z2BA		S[ ] ... C[ ]
1Z2BA	CYS376(SG)	4.84	ILE238(CD1)	1Z2BA		
1Z2BA	CYS376(N)	4.30	THR271(O)	1Z2BA	[D-A-AA]:159.2° [A-D-DD]:42.7° d_planarity:62.0° a_planarity:41.9°, maximum distance exceeded, bad d_angle(sp2), bad d_planarity	N[ ] ... O[ ]
1Z2BA	CYS376(CA)	4.59	THR271(C)	1Z2BA		
1Z2BA	CYS376(CA)	3.38	THR271(O)	1Z2BA		C[ ] ... O[ ]
1Z2BA	CYS376(C)	4.73	THR271(N)	1Z2BA		
1Z2BA	CYS376(C)	4.85	THR271(CA)	1Z2BA		
1Z2BA	CYS376(C)	4.16	THR271(C)	1Z2BA		C[ ] ... C[ ]

1Z2BA	CYS376(C)	2.98	THR271(O)	1Z2BA		C[ ] ... O[ ]
1Z2BA	CYS376(C)	4.92	THR271(CB)	1Z2BA		
1Z2BA	CYS376(O)	3.56	THR271(N)	1Z2BA	[D-A-AA]:162.1° [A-D-DD]:88.9° d_planarity:-5.2° a_planarity:64.5°, maximum distance exceeded, bad d_angle(sp2)	O[ ] ... N[ ]
1Z2BA	CYS376(O)	3.83	THR271(CA)	1Z2BA		O[ ] ... C[ ]
1Z2BA	CYS376(O)	3.29	THR271(C)	1Z2BA		O[ ] ... C[ ]
1Z2BA	CYS376(O)	2.22	THR271(O)	1Z2BA		O[ ] ... O[ ]
1Z2BA	CYS376(O)	4.12	THR271(CB)	1Z2BA		O[ ] ... C[ ]
1Z2BA	CYS376(O)	4.91	THR271(OG1)	1Z2BA	[D-A-AA]:129.2° [A-D-DD]:49.1° a_planarity:52.2°, maximum distance exceeded, bad d_angle(sp3)	
1Z2BA	CYS376(CB)	4.42	THR271(O)	1Z2BA		C[ ] ... O[ ]
1Z2BA	CYS376(SG)	4.21	THR271(O)	1Z2BA		S[ ] ... O[ ]
1Z2BA	CYS376(CA)	4.81	TYR272(CA)	1Z2BA		
1Z2BA	CYS376(O)	4.39	TYR272(N)	1Z2BA	[D-A-AA]:122.6° [A-D-DD]:90.4° d_planarity:9.2° a_planarity:-50.1°, maximum distance exceeded	O[ ] ... N[ ]
1Z2BA	CYS376(O)	4.64	TYR272(CA)	1Z2BA		
1Z2BA	CYS376(SG)	4.54	TYR272(CD1)	1Z2BA		
1Z2BA	CYS376(N)	4.97	ALA273(N)	1Z2BA		
1Z2BA	CYS376(N)	4.69	ALA273(CB)	1Z2BA		
1Z2BA	CYS376(N)	4.66	CYS295(SG)	1Z2BA	[D-A-AA]:165.6° [A-D-DD]:98.4° d_planarity:-88.0°, maximum distance exceeded, bad d_planarity	
1Z2BA	CYS376(C)	4.72	CYS295(SG)	1Z2BA		
1Z2BA	CYS376(C)	4.63	CYS316(O)	1Z2BA		
1Z2BA	CYS376(N)	3.98	LEU318(N)	1Z2BA		N[ ] ... N[ ]
1Z2BA	CYS376(N)	4.15	LEU318(CA)	1Z2BA		N[ ] ... C[ ]
1Z2BA	CYS376(N)	3.60	LEU318(C)	1Z2BA		N[ ] ... C[ ]
1Z2BA	CYS376(N)	2.82	LEU318(O)	1Z2BA	[D-A-AA]:119.9° [A-D-DD]:105.2° d_planarity:-75.0° a_planarity:-69.5°, bad d_planarity	N[ ] ... O[ ]
1Z2BA	CYS376(N)	4.40	LEU318(CB)	1Z2BA		N[ ] ... C[ ]
1Z2BA	CYS376(CA)	4.27	LEU318(N)	1Z2BA		C[ ] ... N[ ]
1Z2BA	CYS376(CA)	4.46	LEU318(CA)	1Z2BA		C[ ] ... C[ ]
1Z2BA	CYS376(CA)	4.29	LEU318(C)	1Z2BA		C[ ] ... C[ ]
1Z2BA	CYS376(CA)	3.48	LEU318(O)	1Z2BA		C[ ] ... O[ ]
1Z2BA	CYS376(CA)	4.25	LEU318(CB)	1Z2BA		C[ ] ... C[ ]
1Z2BA	CYS376(C)	4.33	LEU318(N)	1Z2BA		C[ ] ... N[ ]
1Z2BA	CYS376(C)	4.92	LEU318(CA)	1Z2BA		
1Z2BA	CYS376(C)	4.62	LEU318(O)	1Z2BA		
1Z2BA	CYS376(C)	4.70	LEU318(CB)	1Z2BA		
1Z2BA	CYS376(CB)	4.13	LEU318(N)	1Z2BA		C[ ] ... N[ ]
1Z2BA	CYS376(CB)	3.90	LEU318(CA)	1Z2BA		C[ ] ... C[ ]
1Z2BA	CYS376(CB)	3.85	LEU318(C)	1Z2BA		C[ ] ... C[ ]
1Z2BA	CYS376(CB)	3.02	LEU318(O)	1Z2BA		C[ ] ... O[ ]
1Z2BA	CYS376(CB)	3.25	LEU318(CB)	1Z2BA		C[ ] ... C[ ]
1Z2BA	CYS376(CB)	4.51	LEU318(CG)	1Z2BA		
1Z2BA	CYS376(CB)	4.73	LEU318(CD1)	1Z2BA		
1Z2BA	CYS376(SG)	4.65	LEU318(O)	1Z2BA		
1Z2BA	CYS376(SG)	4.45	LEU318(CB)	1Z2BA		S[ ] ... C[ ]
1Z2BA	CYS376(N)	4.55	TYR319(N)	1Z2BA		
1Z2BA	CYS376(N)	4.98	TYR319(CA)	1Z2BA		
1Z2BA	CYS376(N)	4.43	ALA374(C)	1Z2BA		N[ ] ... C[ ]

1Z2BA	CYS376(N)	4.43	ALA374(O)	1Z2BA	[D-A-AA]:82.3° [A-D-DD]:135.2° d_planarity:50.7° a_planarity:24.1°, maximum distance exceeded, bad a_angle(sp2)	N[ ] ... O[ ]
1Z2BA	CYS376(N)	3.66	VAL375(N)	1Z2BA		N[ ] ... N[ ]
1Z2BA	CYS376(N)	2.45	VAL375(CA)	1Z2BA		N[ ] ... C[ ]
1Z2BA	CYS376(N)	2.26	VAL375(O)	1Z2BA	[D-A-AA]:29.9° [A-D-DD]:91.5° d_planarity:0.4° a_planarity:0.2°, bad a_angle(sp2)	N[ ] ... O[ ]
1Z2BA	CYS376(N)	3.09	VAL375(CB)	1Z2BA		N[ ] ... C[ ]
1Z2BA	CYS376(N)	3.26	VAL375(CG1)	1Z2BA		N[ ] ... C[ ]
1Z2BA	CYS376(N)	4.54	VAL375(CG2)	1Z2BA		
1Z2BA	CYS376(CA)	4.83	VAL375(N)	1Z2BA		
1Z2BA	CYS376(CA)	3.79	VAL375(CA)	1Z2BA		C[ ] ... C[ ]
1Z2BA	CYS376(CA)	2.39	VAL375(C)	1Z2BA		C[ ] ... C[ ]
1Z2BA	CYS376(CA)	2.71	VAL375(O)	1Z2BA		C[ ] ... O[ ]
1Z2BA	CYS376(CA)	4.42	VAL375(CB)	1Z2BA		C[ ] ... C[ ]
1Z2BA	CYS376(CA)	4.57	VAL375(CG1)	1Z2BA		
1Z2BA	CYS376(C)	4.91	VAL375(CA)	1Z2BA		
1Z2BA	CYS376(C)	3.65	VAL375(C)	1Z2BA		C[ ] ... C[ ]
1Z2BA	CYS376(C)	4.00	VAL375(O)	1Z2BA		C[ ] ... O[ ]
1Z2BA	CYS376(O)	4.47	VAL375(C)	1Z2BA		O[ ] ... C[ ]
1Z2BA	CYS376(O)	4.54	VAL375(O)	1Z2BA		
1Z2BA	CYS376(CB)	4.55	VAL375(CA)	1Z2BA		
1Z2BA	CYS376(CB)	3.24	VAL375(C)	1Z2BA		C[ ] ... C[ ]
1Z2BA	CYS376(CB)	3.44	VAL375(O)	1Z2BA		C[ ] ... O[ ]
1Z2BA	CYS376(SG)	4.54	VAL375(C)	1Z2BA		
1Z2BA	CYS376(SG)	4.27	VAL375(O)	1Z2BA		S[ ] ... O[ ]
1Z2BA	CYS376(N)	2.99	MET377(N)	1Z2BA		N[ ] ... N[ ]
1Z2BA	CYS376(N)	4.37	MET377(CA)	1Z2BA		N[ ] ... C[ ]
1Z2BA	CYS376(N)	4.71	MET377(CB)	1Z2BA		
1Z2BA	CYS376(CA)	2.47	MET377(N)	1Z2BA		C[ ] ... N[ ]
1Z2BA	CYS376(CA)	3.82	MET377(CA)	1Z2BA		C[ ] ... C[ ]
1Z2BA	CYS376(CA)	4.58	MET377(C)	1Z2BA		
1Z2BA	CYS376(CA)	4.90	MET377(O)	1Z2BA		
1Z2BA	CYS376(CA)	4.32	MET377(CB)	1Z2BA		C[ ] ... C[ ]
1Z2BA	CYS376(C)	2.42	MET377(CA)	1Z2BA		C[ ] ... C[ ]
1Z2BA	CYS376(C)	3.08	MET377(C)	1Z2BA		C[ ] ... C[ ]
1Z2BA	CYS376(C)	3.42	MET377(O)	1Z2BA		C[ ] ... O[ ]
1Z2BA	CYS376(C)	3.03	MET377(CB)	1Z2BA		C[ ] ... C[ ]
1Z2BA	CYS376(C)	4.43	MET377(CG)	1Z2BA		C[ ] ... C[ ]
1Z2BA	CYS376(O)	2.22	MET377(N)	1Z2BA	[D-A-AA]:30.6° [A-D-DD]:92.4° d_planarity:6.3° a_planarity:1.2°, bad a_angle(sp2)	O[ ] ... N[ ]
1Z2BA	CYS376(O)	2.71	MET377(CA)	1Z2BA		O[ ] ... C[ ]
1Z2BA	CYS376(O)	2.84	MET377(C)	1Z2BA		O[ ] ... C[ ]
1Z2BA	CYS376(O)	2.73	MET377(O)	1Z2BA		O[ ] ... O[ ]
1Z2BA	CYS376(O)	3.10	MET377(CB)	1Z2BA		O[ ] ... C[ ]
1Z2BA	CYS376(O)	4.27	MET377(CG)	1Z2BA		O[ ] ... C[ ]
1Z2BA	CYS376(CB)	3.31	MET377(N)	1Z2BA		C[ ] ... N[ ]
1Z2BA	CYS376(CB)	4.66	MET377(CA)	1Z2BA		
1Z2BA	CYS376(SG)	4.35	MET377(N)	1Z2BA	[D-A-AA]:44.1° [A-D-DD]:130.9° d_planarity:34.6°, maximum distance exceeded, bad a_angle(sp3)	S[ ] ... N[ ]

1Z2BA	CYS376(C)	3.99	LEU378(N)	1Z2BA		C[ ] ... N[ ]
1Z2BA	CYS376(C)	4.84	LEU378(CD2)	1Z2BA		
1Z2BA	CYS376(O)	3.89	LEU378(N)	1Z2BA	[D-A-AA]:85.6° [A-D-DD]:121.5° d_planarity:37.2° a_planarity:27.2°, maximum distance exceeded, bad a_angle(sp2)	O[ ] ... N[ ]
1Z2BA	CYS376(O)	4.81	LEU378(CA)	1Z2BA		
1Z2BA	CYS376(O)	4.76	LEU378(CG)	1Z2BA		
1Z2BA	CYS376(O)	4.18	LEU378(CD2)	1Z2BA		O[ ] ... C[ ]
1Z2BA	CYS376(SG)	4.77	LEU378(CD2)	1Z2BA		

chain1	res1/atm1	distance	res2/atm2	chain2	H-bonding	Charge interaction
2BTPA	CYS134(N)	4.27	VAL132(C)	2BTPA		N[ ] ... C[ ]
2BTPA	CYS134(N)	4.04	VAL132(O)	2BTPA	[D-A-AA]:92.5° [A-D-DD]:125.3° d_planarity:50.6° a_planarity:19.4°, maximum distance exceeded	N[ ] ... O[ ]
2BTPA	CYS134(CB)	4.94	VAL132(O)	2BTPA		
2BTPA	CYS134(N)	3.67	ALA133(N)	2BTPA		N[ ] ... N[ ]
2BTPA	CYS134(N)	2.41	ALA133(CA)	2BTPA		N[ ] ... C[ ]
2BTPA	CYS134(N)	2.25	ALA133(O)	2BTPA	[D-A-AA]:29.5° [A-D-DD]:97.3° d_planarity:1.4° a_planarity:1.8°, bad a_angle(sp2)	N[ ] ... O[ ]
2BTPA	CYS134(N)	3.03	ALA133(CB)	2BTPA		N[ ] ... C[ ]
2BTPA	CYS134(CA)	4.96	ALA133(N)	2BTPA		
2BTPA	CYS134(CA)	3.84	ALA133(CA)	2BTPA		C[ ] ... C[ ]
2BTPA	CYS134(CA)	2.50	ALA133(C)	2BTPA		C[ ] ... C[ ]
2BTPA	CYS134(CA)	2.86	ALA133(O)	2BTPA		C[ ] ... O[ ]
2BTPA	CYS134(CA)	4.41	ALA133(CB)	2BTPA		C[ ] ... C[ ]
2BTPA	CYS134(C)	4.86	ALA133(CA)	2BTPA		
2BTPA	CYS134(C)	3.73	ALA133(C)	2BTPA		C[ ] ... C[ ]
2BTPA	CYS134(C)	4.24	ALA133(O)	2BTPA		C[ ] ... O[ ]
2BTPA	CYS134(O)	4.80	ALA133(CA)	2BTPA		
2BTPA	CYS134(O)	3.98	ALA133(C)	2BTPA		O[ ] ... C[ ]
2BTPA	CYS134(O)	4.74	ALA133(O)	2BTPA		
2BTPA	CYS134(O)	4.68	ALA133(CB)	2BTPA		
2BTPA	CYS134(CB)	4.55	ALA133(CA)	2BTPA		
2BTPA	CYS134(CB)	3.29	ALA133(C)	2BTPA		C[ ] ... C[ ]
2BTPA	CYS134(CB)	3.55	ALA133(O)	2BTPA		C[ ] ... O[ ]
2BTPA	CYS134(SG)	4.70	ALA133(CA)	2BTPA		
2BTPA	CYS134(SG)	3.91	ALA133(C)	2BTPA		S[ ] ... C[ ]
2BTPA	CYS134(SG)	4.60	ALA133(O)	2BTPA		
2BTPA	CYS134(N)	3.74	GLY135(N)	2BTPA		N[ ] ... N[ ]
2BTPA	CYS134(N)	4.98	GLY135(CA)	2BTPA		
2BTPA	CYS134(CA)	2.48	GLY135(N)	2BTPA		C[ ] ... N[ ]
2BTPA	CYS134(CA)	3.87	GLY135(CA)	2BTPA		C[ ] ... C[ ]
2BTPA	CYS134(CA)	4.40	GLY135(C)	2BTPA		C[ ] ... C[ ]
2BTPA	CYS134(CA)	4.39	GLY135(O)	2BTPA		C[ ] ... O[ ]
2BTPA	CYS134(C)	2.46	GLY135(CA)	2BTPA		C[ ] ... C[ ]
2BTPA	CYS134(C)	2.91	GLY135(C)	2BTPA		C[ ] ... C[ ]

2BTPA	CYS134(C)	3.13	GLY135(O)	2BTPA		C[ ] ... O[ ]
2BTPA	CYS134(O)	2.25	GLY135(N)	2BTPA	[D-A-AA]:30.3° [A-D-DD]:94.0° d_planarity:2.7° a_planarity:1.5°, bad a_angle(sp2)	O[ ] ... N[ ]
2BTPA	CYS134(O)	2.78	GLY135(CA)	2BTPA		O[ ] ... C[ ]
2BTPA	CYS134(O)	2.69	GLY135(C)	2BTPA		O[ ] ... C[ ]
2BTPA	CYS134(O)	2.96	GLY135(O)	2BTPA		O[ ] ... O[ ]
2BTPA	CYS134(CB)	2.96	GLY135(N)	2BTPA		C[ ] ... N[ ]
2BTPA	CYS134(CB)	4.37	GLY135(CA)	2BTPA		C[ ] ... C[ ]
2BTPA	CYS134(SG)	3.67	GLY135(N)	2BTPA	[D-A-AA]:52.9° [A-D-DD]:131.3° d_planarity:66.7°, bad a_angle(sp3), bad d_planarity	S[ ] ... N[ ]
2BTPA	CYS134(SG)	4.78	GLY135(CA)	2BTPA		
2BTPA	CYS134(C)	3.81	ASP136(N)	2BTPA		C[ ] ... N[ ]
2BTPA	CYS134(C)	4.90	ASP136(CA)	2BTPA		
2BTPA	CYS134(C)	4.99	ASP136(C)	2BTPA		
2BTPA	CYS134(O)	3.23	ASP136(N)	2BTPA	[D-A-AA]:108.1° [A-D-DD]:117.1° d_planarity:63.6° a_planarity:10.4°, bad d_planarity	O[ ] ... N[ ]
2BTPA	CYS134(O)	4.12	ASP136(CA)	2BTPA		O[ ] ... C[ ]
2BTPA	CYS134(O)	3.93	ASP136(C)	2BTPA		O[ ] ... C[ ]
2BTPA	CYS134(O)	4.85	ASP136(O)	2BTPA		
2BTPA	CYS134(N)	4.80	ASP137(C)	2BTPA		
2BTPA	CYS134(N)	4.54	ASP137(CB)	2BTPA		
2BTPA	CYS134(N)	4.76	ASP137(OD2)	2BTPA	[D-A-AA]:97.7° [A-D-DD]:134.7° d_planarity:-18.9° a_planarity:33.5°, maximum distance exceeded	
2BTPA	CYS134(C)	4.44	ASP137(N)	2BTPA		C[ ] ... N[ ]
2BTPA	CYS134(C)	4.79	ASP137(CA)	2BTPA		
2BTPA	CYS134(C)	4.69	ASP137(C)	2BTPA		
2BTPA	CYS134(C)	4.41	ASP137(CB)	2BTPA		C[ ] ... C[ ]
2BTPA	CYS134(O)	3.27	ASP137(N)	2BTPA	[D-A-AA]:158.0° [A-D-DD]:88.8° d_planarity:-66.6° a_planarity:8.2°, bad d_angle(sp2), bad d_planarity	O[ ] ... N[ ]
2BTPA	CYS134(O)	3.55	ASP137(CA)	2BTPA		O[ ] ... C[ ]
2BTPA	CYS134(O)	3.55	ASP137(C)	2BTPA		O[ ] ... C[ ]
2BTPA	CYS134(O)	4.69	ASP137(O)	2BTPA		
2BTPA	CYS134(O)	3.25	ASP137(CB)	2BTPA		O[ ] ... C[ ]
2BTPA	CYS134(O)	4.51	ASP137(CG)	2BTPA		
2BTPA	CYS134(O)	4.80	ASP137(OD2)	2BTPA		
2BTPA	CYS134(N)	4.01	ARG138(N)	2BTPA		N[ ] ... N[ ]
2BTPA	CYS134(N)	4.20	ARG138(CA)	2BTPA		N[ ] ... C[ ]
2BTPA	CYS134(N)	3.80	ARG138(CB)	2BTPA		N[ ] ... C[ ]
2BTPA	CYS134(N)	4.32	ARG138(CG)	2BTPA		N[ ] ... C[ ]
2BTPA	CYS134(N)	4.84	ARG138(CD)	2BTPA		
2BTPA	CYS134(CA)	4.41	ARG138(N)	2BTPA		C[ ] ... N[ ]
2BTPA	CYS134(CA)	4.64	ARG138(CA)	2BTPA		
2BTPA	CYS134(CA)	3.87	ARG138(CB)	2BTPA		C[ ] ... C[ ]
2BTPA	CYS134(CA)	4.51	ARG138(CG)	2BTPA		
2BTPA	CYS134(CA)	4.65	ARG138(CD)	2BTPA		
2BTPA	CYS134(C)	3.71	ARG138(N)	2BTPA		C[ ] ... N[ ]
2BTPA	CYS134(C)	4.27	ARG138(CA)	2BTPA		C[ ] ... C[ ]
2BTPA	CYS134(C)	3.64	ARG138(CB)	2BTPA		C[ ] ... C[ ]
2BTPA	CYS134(C)	4.74	ARG138(CG)	2BTPA		
2BTPA	CYS134(C)	5.00	ARG138(CD)	2BTPA		

2BTPA	CYS134(O)	2.72	ARG138(N)	2BTPA	**H-bond** [D-A-AA]:134.9° [A-D-DD]:116.6° d_planarity:-23.4° a_planarity:-69.1°	O[ ] ... N[ ]
2BTPA	CYS134(O)	3.61	ARG138(CA)	2BTPA		O[ ] ... C[ ]
2BTPA	CYS134(O)	4.88	ARG138(C)	2BTPA		
2BTPA	CYS134(O)	3.43	ARG138(CB)	2BTPA		O[ ] ... C[ ]
2BTPA	CYS134(O)	4.75	ARG138(CG)	2BTPA		

chain1	res1/atm1	distance	res2/atm2	chain2	H-bonding	Charge interaction
1Z7XW	CYS37(SG)	4.62	ILE9(CA)	1Z7XW		
1Z7XW	CYS37(SG)	3.99	ILE9(C)	1Z7XW		S[ ] ... C[ ]
1Z7XW	CYS37(SG)	3.69	ILE9(O)	1Z7XW		S[ ] ... O[ ]
1Z7XW	CYS37(SG)	3.94	ILE9(CB)	1Z7XW		S[ ] ... C[ ]
1Z7XW	CYS37(SG)	3.62	ILE9(CG2)	1Z7XW		S[ ] ... C[ ]
1Z7XW	CYS37(SG)	4.47	GLN10(N)	1Z7XW	[D-A-AA]:164.3° [A-D-DD]:84.9° d_planarity:51.0°, maximum distance exceeded, bad d_angle(sp2)	S[ ] ... N[ ]
1Z7XW	CYS37(SG)	4.58	GLN10(CA)	1Z7XW		
1Z7XW	CYS37(SG)	4.71	GLN10(C)	1Z7XW		
1Z7XW	CYS37(N)	4.74	CYS11(N)	1Z7XW		
1Z7XW	CYS37(SG)	4.40	CYS11(N)	1Z7XW	[D-A-AA]:117.4° [A-D-DD]:121.1° d_planarity:-58.2°, maximum distance exceeded	S[ ] ... N[ ]
1Z7XW	CYS37(N)	4.95	GLU12(N)	1Z7XW		
1Z7XW	CYS37(N)	4.28	GLU12(O)	1Z7XW	[D-A-AA]:143.5° [A-D-DD]:31.1° d_planarity:8.2° a_planarity:-46.5°, maximum distance exceeded, bad d_angle(sp2)	N[ ] ... O[ ]
1Z7XW	CYS37(CA)	4.49	GLU12(N)	1Z7XW		C[ ] ... N[ ]
1Z7XW	CYS37(CA)	5.00	GLU12(CA)	1Z7XW		
1Z7XW	CYS37(CA)	4.31	GLU12(C)	1Z7XW		C[ ] ... C[ ]
1Z7XW	CYS37(CA)	3.12	GLU12(O)	1Z7XW		C[ ] ... O[ ]
1Z7XW	CYS37(C)	4.70	GLU12(C)	1Z7XW		
1Z7XW	CYS37(C)	3.50	GLU12(O)	1Z7XW		C[ ] ... O[ ]
1Z7XW	CYS37(O)	4.60	GLU12(C)	1Z7XW		
1Z7XW	CYS37(O)	3.43	GLU12(O)	1Z7XW		O[ ] ... O[ ]
1Z7XW	CYS37(CB)	4.95	GLU12(N)	1Z7XW		
1Z7XW	CYS37(CB)	4.75	GLU12(C)	1Z7XW		
1Z7XW	CYS37(CB)	3.58	GLU12(O)	1Z7XW		C[ ] ... O[ ]
1Z7XW	CYS37(SG)	4.36	GLU12(N)	1Z7XW	[D-A-AA]:98.8° [A-D-DD]:102.8° d_planarity:-5.4°, maximum distance exceeded	S[ ] ... N[ ]
1Z7XW	CYS37(SG)	4.90	GLU12(CA)	1Z7XW		
1Z7XW	CYS37(SG)	4.85	GLU12(C)	1Z7XW		
1Z7XW	CYS37(SG)	3.97	GLU12(O)	1Z7XW		S[ ] ... O[ ]
1Z7XW	CYS37(SG)	4.68	GLU12(CB)	1Z7XW	Negative	
1Z7XW	CYS37(C)	4.81	GLU13(CD)	1Z7XW		
1Z7XW	CYS37(C)	4.29	GLU13(OE1)	1Z7XW		C[ ] ... O[-]
1Z7XW	CYS37(C)	4.84	GLU13(OE2)	1Z7XW		
1Z7XW	CYS37(O)	4.64	GLU13(CA)	1Z7XW		
1Z7XW	CYS37(O)	4.99	GLU13(C)	1Z7XW		
1Z7XW	CYS37(O)	4.45	GLU13(CD)	1Z7XW		O[ ] ... C[ ]
1Z7XW	CYS37(O)	4.21	GLU13(OE1)	1Z7XW		O[ ] ... O[-]

1Z7XW	CYS37(O)	4.42	GLU13(OE2)	1Z7XW		O[ ] ... O[-]
1Z7XW	CYS37(CA)	4.89	LEU14(CD1)	1Z7XW		
1Z7XW	CYS37(C)	4.98	LEU14(CD1)	1Z7XW		
1Z7XW	CYS37(O)	4.29	LEU14(N)	1Z7XW	[D-A-AA]:137.3° [A-D-DD]:115.1° d_planarity:-34.6° a_planarity:-60.3°, maximum distance exceeded	O[ ] ... N[ ]
1Z7XW	CYS37(O)	4.50	LEU14(CB)	1Z7XW		
1Z7XW	CYS37(O)	4.26	LEU14(CG)	1Z7XW		O[ ] ... C[ ]
1Z7XW	CYS37(O)	4.19	LEU14(CD1)	1Z7XW		O[ ] ... C[ ]
1Z7XW	CYS37(CB)	4.24	LEU14(CG)	1Z7XW		C[ ] ... C[ ]
1Z7XW	CYS37(CB)	3.73	LEU14(CD1)	1Z7XW		C[ ] ... C[ ]
1Z7XW	CYS37(SG)	4.63	LEU14(CG)	1Z7XW		
1Z7XW	CYS37(SG)	4.21	LEU14(CD1)	1Z7XW		S[ ] ... C[ ]
1Z7XW	CYS37(N)	4.74	LEU34(C)	1Z7XW		
1Z7XW	CYS37(N)	4.70	LEU34(O)	1Z7XW	[D-A-AA]:84.2° [A-D-DD]:111.9° d_planarity:-40.3° a_planarity:57.9°, maximum distance exceeded, bad a_angle(sp2)	
1Z7XW	CYS37(CB)	4.85	LEU34(CA)	1Z7XW		
1Z7XW	CYS37(CB)	4.56	LEU34(C)	1Z7XW		
1Z7XW	CYS37(CB)	4.80	LEU34(O)	1Z7XW		
1Z7XW	CYS37(CB)	4.69	LEU34(CB)	1Z7XW		
1Z7XW	CYS37(SG)	4.06	LEU34(CA)	1Z7XW		S[ ] ... C[ ]
1Z7XW	CYS37(SG)	3.94	LEU34(C)	1Z7XW		S[ ] ... C[ ]
1Z7XW	CYS37(SG)	4.61	LEU34(O)	1Z7XW		
1Z7XW	CYS37(SG)	4.36	LEU34(CB)	1Z7XW		S[ ] ... C[ ]
1Z7XW	CYS37(SG)	4.68	LEU34(CD2)	1Z7XW		
1Z7XW	CYS37(N)	4.58	ASP35(N)	1Z7XW		
1Z7XW	CYS37(N)	4.45	ASP35(CA)	1Z7XW		N[ ] ... C[ ]
1Z7XW	CYS37(N)	3.29	ASP35(C)	1Z7XW		N[ ] ... C[ ]
1Z7XW	CYS37(N)	3.31	ASP35(O)	1Z7XW	[D-A-AA]:78.4° [A-D-DD]:119.6° d_planarity:65.8° a_planarity:46.7°, bad a_angle(sp2), bad d_planarity	N[ ] ... O[ ]
1Z7XW	CYS37(CA)	4.46	ASP35(C)	1Z7XW		C[ ] ... C[ ]
1Z7XW	CYS37(CA)	4.22	ASP35(O)	1Z7XW		C[ ] ... O[ ]
1Z7XW	CYS37(CB)	4.77	ASP35(N)	1Z7XW		
1Z7XW	CYS37(CB)	4.66	ASP35(C)	1Z7XW		
1Z7XW	CYS37(CB)	4.44	ASP35(O)	1Z7XW		C[ ] ... O[ ]
1Z7XW	CYS37(SG)	3.84	ASP35(N)	1Z7XW	[D-A-AA]:111.0° [A-D-DD]:113.7° d_planarity:-79.4°, bad d_planarity	S[ ] ... N[ ]
1Z7XW	CYS37(SG)	4.63	ASP35(CA)	1Z7XW		
1Z7XW	CYS37(SG)	4.09	ASP35(C)	1Z7XW		S[ ] ... C[ ]
1Z7XW	CYS37(SG)	3.58	ASP35(O)	1Z7XW		S[ ] ... O[ ]
1Z7XW	CYS37(N)	2.87	ASP36(N)	1Z7XW		N[ ] ... N[ ]
1Z7XW	CYS37(N)	2.46	ASP36(CA)	1Z7XW		N[ ] ... C[ ]
1Z7XW	CYS37(N)	2.24	ASP36(O)	1Z7XW	[D-A-AA]:30.1° [A-D-DD]:95.3° d_planarity:3.3° a_planarity:4.7°, bad a_angle(sp2)	N[ ] ... O[ ]
1Z7XW	CYS37(N)	3.79	ASP36(CB)	1Z7XW		N[ ] ... C[ ]
1Z7XW	CYS37(N)	4.43	ASP36(CG)	1Z7XW		N[ ] ... C[ ]
1Z7XW	CYS37(N)	4.35	ASP36(OD1)	1Z7XW	[D-A-AA]:85.7° [A-D-DD]:125.3° d_planarity:40.0° a_planarity:36.1°, maximum distance exceeded, bad a_angle(sp2)	N[ ] ... O[-]
1Z7XW	CYS37(CA)	4.27	ASP36(N)	1Z7XW		C[ ] ... N[ ]
1Z7XW	CYS37(CA)	3.84	ASP36(CA)	1Z7XW		C[ ] ... C[ ]

1Z7XW	CYS37(CA)	2.45	ASP36(C)	1Z7XW		C[ ] ... C[ ]
1Z7XW	CYS37(CA)	2.79	ASP36(O)	1Z7XW		C[ ] ... O[ ]
1Z7XW	CYS37(C)	4.75	ASP36(CA)	1Z7XW		
1Z7XW	CYS37(C)	3.49	ASP36(C)	1Z7XW		C[ ] ... C[ ]
1Z7XW	CYS37(C)	3.83	ASP36(O)	1Z7XW		C[ ] ... O[ ]
1Z7XW	CYS37(O)	4.63	ASP36(C)	1Z7XW		
1Z7XW	CYS37(O)	4.84	ASP36(O)	1Z7XW		
1Z7XW	CYS37(CB)	4.79	ASP36(N)	1Z7XW		
1Z7XW	CYS37(CB)	4.80	ASP36(CA)	1Z7XW		
1Z7XW	CYS37(CB)	3.56	ASP36(C)	1Z7XW		C[ ] ... C[ ]
1Z7XW	CYS37(CB)	3.95	ASP36(O)	1Z7XW		C[ ] ... O[ ]
1Z7XW	CYS37(SG)	4.70	ASP36(N)	1Z7XW	[D-A-AA]:82.1° [A-D-DD]:91.7° d_planarity:49.8°, maximum distance exceeded	
1Z7XW	CYS37(SG)	4.97	ASP36(CA)	1Z7XW		
1Z7XW	CYS37(SG)	3.87	ASP36(C)	1Z7XW		S[ ] ... C[ ]
1Z7XW	CYS37(SG)	4.05	ASP36(O)	1Z7XW		S[ ] ... O[ ]
1Z7XW	CYS37(N)	2.76	GLY38(N)	1Z7XW		N[ ] ... N[ ]
1Z7XW	CYS37(N)	4.20	GLY38(CA)	1Z7XW		N[ ] ... C[ ]
1Z7XW	CYS37(N)	4.97	GLY38(C)	1Z7XW		
1Z7XW	CYS37(CA)	2.44	GLY38(N)	1Z7XW		C[ ] ... N[ ]
1Z7XW	CYS37(CA)	3.81	GLY38(CA)	1Z7XW		C[ ] ... C[ ]
1Z7XW	CYS37(CA)	4.51	GLY38(C)	1Z7XW		
1Z7XW	CYS37(C)	2.44	GLY38(CA)	1Z7XW		C[ ] ... C[ ]
1Z7XW	CYS37(C)	3.10	GLY38(C)	1Z7XW		C[ ] ... C[ ]
1Z7XW	CYS37(C)	4.02	GLY38(O)	1Z7XW		C[ ] ... O[ ]
1Z7XW	CYS37(O)	2.26	GLY38(N)	1Z7XW	[D-A-AA]:29.4° [A-D-DD]:95.1° d_planarity:2.1° a_planarity:0.0°, bad a_angle(sp2)	O[ ] ... N[ ]
1Z7XW	CYS37(O)	2.80	GLY38(CA)	1Z7XW		O[ ] ... C[ ]
1Z7XW	CYS37(O)	3.09	GLY38(C)	1Z7XW		O[ ] ... C[ ]
1Z7XW	CYS37(O)	3.69	GLY38(O)	1Z7XW		O[ ] ... O[ ]
1Z7XW	CYS37(CB)	3.59	GLY38(N)	1Z7XW		C[ ] ... N[ ]
1Z7XW	CYS37(CB)	4.82	GLY38(CA)	1Z7XW		
1Z7XW	CYS37(N)	4.71	LEU39(N)	1Z7XW		
1Z7XW	CYS37(CA)	4.45	LEU39(N)	1Z7XW		C[ ] ... N[ ]
1Z7XW	CYS37(C)	3.30	LEU39(N)	1Z7XW		C[ ] ... N[ ]
1Z7XW	CYS37(C)	4.56	LEU39(CA)	1Z7XW		
1Z7XW	CYS37(C)	4.61	LEU39(CG)	1Z7XW		
1Z7XW	CYS37(C)	4.87	LEU39(CD2)	1Z7XW		
1Z7XW	CYS37(O)	3.45	LEU39(N)	1Z7XW	[D-A-AA]:72.4° [A-D-DD]:127.3° d_planarity:77.4° a_planarity:44.5°, bad a_angle(sp2), bad d_planarity	O[ ] ... N[ ]
1Z7XW	CYS37(O)	4.49	LEU39(CA)	1Z7XW		O[ ] ... C[ ]
1Z7XW	CYS37(O)	4.60	LEU39(CG)	1Z7XW		
1Z7XW	CYS37(O)	4.48	LEU39(CD2)	1Z7XW		O[ ] ... C[ ]
1Z7XW	CYS37(CB)	4.82	LEU39(N)	1Z7XW		
1Z7XW	CYS37(CB)	4.49	LEU39(CG)	1Z7XW		C[ ] ... C[ ]
1Z7XW	CYS37(CB)	4.59	LEU39(CD2)	1Z7XW		
1Z7XW	CYS37(N)	4.57	SER64(O)	1Z7XW	[D-A-AA]:126.9° [A-D-DD]:152.0° d_planarity:-75.7° a_planarity:63.2°, maximum distance exceeded, bad d_planarity	
1Z7XW	CYS37(N)	3.94	ASN65(CG)	1Z7XW		N[ ] ... C[ ]
1Z7XW	CYS37(N)	3.02	ASN65(OD1)	1Z7XW	**H-bond** [D-A-AA]:130.6° [A-D-DD]:105.6° d_planarity:-26.7°	N[ ] ... O[ ]

					a_planarity:-35.9°	
1Z7XW	CYS37(N)	4.22	ASN65(ND2)	1Z7XW		N[ ] ... N[ ]
1Z7XW	CYS37(CA)	4.79	ASN65(CG)	1Z7XW		
1Z7XW	CYS37(CA)	3.70	ASN65(OD1)	1Z7XW		C[ ] ... O[ ]
1Z7XW	CYS37(C)	4.83	ASN65(CG)	1Z7XW		
1Z7XW	CYS37(C)	3.60	ASN65(OD1)	1Z7XW		C[ ] ... O[ ]
1Z7XW	CYS37(O)	4.49	ASN65(OD1)	1Z7XW		O[ ] ... O[ ]
1Z7XW	CYS37(CB)	4.10	ASN65(OD1)	1Z7XW		C[ ] ... O[ ]

chain1	res1/atm1	distance	res2/atm2	chain2	H-bonding	Charge interaction
1Z7XW	CYS247(N)	4.95	GLY242(O)	1Z7XW	[D-A-AA]:134.8° [A-D-DD]:153.2° d_planarity:-21.8° a_planarity:11.3°, maximum distance exceeded	
1Z7XW	CYS247(N)	4.97	MET243(CA)	1Z7XW		
1Z7XW	CYS247(N)	4.10	MET243(C)	1Z7XW		N[ ] ... C[ ]
1Z7XW	CYS247(N)	2.91	MET243(O)	1Z7XW	**H-bond** [D-A-AA]:158.0° [A-D-DD]:110.3° d_planarity:-13.5° a_planarity:-80.3°	N[ ] ... O[ ]
1Z7XW	CYS247(CA)	4.93	MET243(C)	1Z7XW		
1Z7XW	CYS247(CA)	3.68	MET243(O)	1Z7XW		C[ ] ... O[ ]
1Z7XW	CYS247(C)	4.94	MET243(O)	1Z7XW		
1Z7XW	CYS247(CB)	4.43	MET243(C)	1Z7XW		C[ ] ... C[ ]
1Z7XW	CYS247(CB)	3.27	MET243(O)	1Z7XW		C[ ] ... O[ ]
1Z7XW	CYS247(SG)	4.69	MET243(CA)	1Z7XW		
1Z7XW	CYS247(SG)	4.18	MET243(C)	1Z7XW		S[ ] ... C[ ]
1Z7XW	CYS247(SG)	3.23	MET243(O)	1Z7XW		S[ ] ... O[ ]
1Z7XW	CYS247(SG)	4.72	MET243(CB)	1Z7XW		
1Z7XW	CYS247(SG)	3.80	MET243(CE)	1Z7XW		S[ ] ... C[ ]
1Z7XW	CYS247(N)	4.96	ALA244(N)	1Z7XW		
1Z7XW	CYS247(N)	4.91	ALA244(CA)	1Z7XW		
1Z7XW	CYS247(N)	4.29	ALA244(C)	1Z7XW		N[ ] ... C[ ]
1Z7XW	CYS247(N)	4.26	ALA244(O)	1Z7XW	[D-A-AA]:83.5° [A-D-DD]:106.6° d_planarity:-49.0° a_planarity:79.3°, maximum distance exceeded, bad a_angle(sp2)	N[ ] ... O[ ]
1Z7XW	CYS247(CA)	4.88	ALA244(O)	1Z7XW		
1Z7XW	CYS247(CB)	5.00	ALA244(CA)	1Z7XW		
1Z7XW	CYS247(CB)	4.98	ALA244(C)	1Z7XW		
1Z7XW	CYS247(CB)	4.80	ALA244(O)	1Z7XW		
1Z7XW	CYS247(N)	4.38	GLU245(N)	1Z7XW		N[ ] ... N[ ]
1Z7XW	CYS247(N)	4.55	GLU245(CA)	1Z7XW		
1Z7XW	CYS247(N)	3.57	GLU245(C)	1Z7XW		N[ ] ... C[ ]
1Z7XW	CYS247(N)	4.01	GLU245(O)	1Z7XW	[D-A-AA]:60.7° [A-D-DD]:137.3° d_planarity:67.3° a_planarity:42.3°, maximum distance exceeded, bad a_angle(sp2), bad d_planarity	N[ ] ... O[ ]
1Z7XW	CYS247(CA)	4.87	GLU245(C)	1Z7XW		
1Z7XW	CYS247(N)	2.76	LEU246(N)	1Z7XW		N[ ] ... N[ ]
1Z7XW	CYS247(N)	2.39	LEU246(CA)	1Z7XW		N[ ] ... C[ ]
1Z7XW	CYS247(N)	2.27	LEU246(O)	1Z7XW	[D-A-AA]:29.0° [A-D-DD]:95.9° d_planarity:4.6° a_planarity:2.2°, bad a_angle(sp2)	N[ ] ... O[ ]
1Z7XW	CYS247(N)	3.07	LEU246(CB)	1Z7XW		N[ ] ... C[ ]

1Z7XW	CYS247(N)	4.00	LEU246(CG)	1Z7XW		N[ ] ... C[ ]
1Z7XW	CYS247(N)	4.92	LEU246(CD1)	1Z7XW		
1Z7XW	CYS247(CA)	4.20	LEU246(N)	1Z7XW		C[ ] ... N[ ]
1Z7XW	CYS247(CA)	3.78	LEU246(CA)	1Z7XW		C[ ] ... C[ ]
1Z7XW	CYS247(CA)	2.45	LEU246(C)	1Z7XW		C[ ] ... C[ ]
1Z7XW	CYS247(CA)	2.83	LEU246(O)	1Z7XW		C[ ] ... O[ ]
1Z7XW	CYS247(CA)	4.34	LEU246(CB)	1Z7XW		C[ ] ... C[ ]
1Z7XW	CYS247(C)	4.88	LEU246(N)	1Z7XW		
1Z7XW	CYS247(C)	4.51	LEU246(CA)	1Z7XW		
1Z7XW	CYS247(C)	3.08	LEU246(C)	1Z7XW		C[ ] ... C[ ]
1Z7XW	CYS247(C)	2.97	LEU246(O)	1Z7XW		C[ ] ... O[ ]
1Z7XW	CYS247(O)	4.02	LEU246(C)	1Z7XW		O[ ] ... C[ ]
1Z7XW	CYS247(O)	3.63	LEU246(O)	1Z7XW		O[ ] ... O[ ]
1Z7XW	CYS247(CB)	4.94	LEU246(N)	1Z7XW		
1Z7XW	CYS247(CB)	4.83	LEU246(CA)	1Z7XW		
1Z7XW	CYS247(CB)	3.75	LEU246(C)	1Z7XW		C[ ] ... C[ ]
1Z7XW	CYS247(CB)	4.34	LEU246(O)	1Z7XW		C[ ] ... O[ ]
1Z7XW	CYS247(SG)	4.47	LEU246(C)	1Z7XW		S[ ] ... C[ ]
1Z7XW	CYS247(N)	2.88	PRO248(N)	1Z7XW		N[ ] ... N[ ]
1Z7XW	CYS247(N)	4.26	PRO248(CA)	1Z7XW		N[ ] ... C[ ]
1Z7XW	CYS247(N)	4.80	PRO248(C)	1Z7XW		
1Z7XW	CYS247(N)	4.90	PRO248(CB)	1Z7XW		
1Z7XW	CYS247(N)	4.49	PRO248(CG)	1Z7XW		N[ ] ... C[ ]
1Z7XW	CYS247(N)	2.97	PRO248(CD)	1Z7XW		N[ ] ... C[ ]
1Z7XW	CYS247(CA)	2.51	PRO248(N)	1Z7XW		C[ ] ... N[ ]
1Z7XW	CYS247(CA)	3.81	PRO248(CA)	1Z7XW		C[ ] ... C[ ]
1Z7XW	CYS247(CA)	4.34	PRO248(C)	1Z7XW		C[ ] ... C[ ]
1Z7XW	CYS247(CA)	4.83	PRO248(CB)	1Z7XW		
1Z7XW	CYS247(CA)	4.56	PRO248(CG)	1Z7XW		
1Z7XW	CYS247(CA)	3.18	PRO248(CD)	1Z7XW		C[ ] ... C[ ]
1Z7XW	CYS247(C)	2.37	PRO248(CA)	1Z7XW		C[ ] ... C[ ]
1Z7XW	CYS247(C)	2.92	PRO248(C)	1Z7XW		C[ ] ... C[ ]
1Z7XW	CYS247(C)	3.74	PRO248(O)	1Z7XW		C[ ] ... O[ ]
1Z7XW	CYS247(C)	3.61	PRO248(CB)	1Z7XW		C[ ] ... C[ ]
1Z7XW	CYS247(C)	3.65	PRO248(CG)	1Z7XW		C[ ] ... C[ ]
1Z7XW	CYS247(C)	2.61	PRO248(CD)	1Z7XW		C[ ] ... C[ ]
1Z7XW	CYS247(O)	2.26	PRO248(N)	1Z7XW		O[ ] ... N[ ]
1Z7XW	CYS247(O)	2.63	PRO248(CA)	1Z7XW		O[ ] ... C[ ]
1Z7XW	CYS247(O)	2.75	PRO248(C)	1Z7XW		O[ ] ... C[ ]
1Z7XW	CYS247(O)	3.19	PRO248(O)	1Z7XW		O[ ] ... O[ ]
1Z7XW	CYS247(O)	4.11	PRO248(CB)	1Z7XW		O[ ] ... C[ ]
1Z7XW	CYS247(O)	4.45	PRO248(CG)	1Z7XW		O[ ] ... C[ ]
1Z7XW	CYS247(O)	3.68	PRO248(CD)	1Z7XW		O[ ] ... C[ ]
1Z7XW	CYS247(CB)	3.46	PRO248(N)	1Z7XW		C[ ] ... N[ ]
1Z7XW	CYS247(CB)	4.76	PRO248(CA)	1Z7XW		
1Z7XW	CYS247(CB)	3.88	PRO248(CD)	1Z7XW		C[ ] ... C[ ]
1Z7XW	CYS247(N)	4.46	GLY249(N)	1Z7XW		N[ ] ... N[ ]
1Z7XW	CYS247(CA)	4.31	GLY249(N)	1Z7XW		C[ ] ... N[ ]
1Z7XW	CYS247(C)	3.21	GLY249(N)	1Z7XW		C[ ] ... N[ ]

1Z7XW	CYS247(C)	4.43	GLY249(CA)	1Z7XW		C[ ] ... C[ ]
1Z7XW	CYS247(C)	4.56	GLY249(C)	1Z7XW		
1Z7XW	CYS247(O)	3.24	GLY249(N)	1Z7XW	[D-A-AA]:77.8° [A-D-DD]:122.0° d_planarity:68.7° a_planarity:54.2°, bad a_angle(sp2), bad d_planarity	O[ ] ... N[ ]
1Z7XW	CYS247(O)	4.22	GLY249(CA)	1Z7XW		O[ ] ... C[ ]
1Z7XW	CYS247(O)	4.01	GLY249(C)	1Z7XW		O[ ] ... C[ ]
1Z7XW	CYS247(O)	4.95	GLY249(O)	1Z7XW		
1Z7XW	CYS247(N)	4.95	LEU250(N)	1Z7XW		
1Z7XW	CYS247(N)	4.91	LEU250(CB)	1Z7XW		
1Z7XW	CYS247(N)	4.65	LEU250(CG)	1Z7XW		
1Z7XW	CYS247(N)	4.17	LEU250(CD1)	1Z7XW		N[ ] ... C[ ]
1Z7XW	CYS247(CA)	4.41	LEU250(N)	1Z7XW		C[ ] ... N[ ]
1Z7XW	CYS247(CA)	4.84	LEU250(CA)	1Z7XW		
1Z7XW	CYS247(CA)	4.10	LEU250(CB)	1Z7XW		C[ ] ... C[ ]
1Z7XW	CYS247(CA)	4.25	LEU250(CG)	1Z7XW		C[ ] ... C[ ]
1Z7XW	CYS247(CA)	3.86	LEU250(CD1)	1Z7XW		C[ ] ... C[ ]
1Z7XW	CYS247(C)	3.81	LEU250(N)	1Z7XW		C[ ] ... N[ ]
1Z7XW	CYS247(C)	4.46	LEU250(CA)	1Z7XW		C[ ] ... C[ ]
1Z7XW	CYS247(C)	4.81	LEU250(C)	1Z7XW		
1Z7XW	CYS247(C)	4.19	LEU250(CB)	1Z7XW		C[ ] ... C[ ]
1Z7XW	CYS247(C)	4.75	LEU250(CG)	1Z7XW		
1Z7XW	CYS247(C)	4.80	LEU250(CD1)	1Z7XW		
1Z7XW	CYS247(O)	3.26	LEU250(N)	1Z7XW	**H-bond** [D-A-AA]:107.5° [A-D-DD]:94.8° d_planarity:-55.1° a_planarity:-83.8°	O[ ] ... N[ ]
1Z7XW	CYS247(O)	3.69	LEU250(CA)	1Z7XW		O[ ] ... C[ ]
1Z7XW	CYS247(O)	3.74	LEU250(C)	1Z7XW		O[ ] ... C[ ]
1Z7XW	CYS247(O)	4.88	LEU250(O)	1Z7XW		
1Z7XW	CYS247(O)	3.60	LEU250(CB)	1Z7XW		O[ ] ... C[ ]
1Z7XW	CYS247(O)	4.53	LEU250(CG)	1Z7XW		
1Z7XW	CYS247(O)	4.82	LEU250(CD1)	1Z7XW		
1Z7XW	CYS247(CB)	4.52	LEU250(CD1)	1Z7XW		
1Z7XW	CYS247(SG)	3.96	LEU250(CD1)	1Z7XW		S[ ] ... C[ ]
1Z7XW	CYS247(C)	4.16	LEU251(N)	1Z7XW		C[ ] ... N[ ]
1Z7XW	CYS247(C)	4.71	LEU251(CB)	1Z7XW		
1Z7XW	CYS247(C)	4.35	LEU251(CG)	1Z7XW		C[ ] ... C[ ]
1Z7XW	CYS247(C)	4.42	LEU251(CD1)	1Z7XW		C[ ] ... C[ ]
1Z7XW	CYS247(O)	2.98	LEU251(N)	1Z7XW	**H-bond** [D-A-AA]:160.3° [A-D-DD]:117.2° d_planarity:-27.1° a_planarity:87.9°	O[ ] ... N[ ]
1Z7XW	CYS247(O)	3.88	LEU251(CA)	1Z7XW		O[ ] ... C[ ]
1Z7XW	CYS247(O)	3.58	LEU251(CB)	1Z7XW		O[ ] ... C[ ]
1Z7XW	CYS247(O)	3.32	LEU251(CG)	1Z7XW		O[ ] ... C[ ]
1Z7XW	CYS247(O)	3.70	LEU251(CD1)	1Z7XW		O[ ] ... C[ ]
1Z7XW	CYS247(O)	4.63	LEU251(CD2)	1Z7XW		
1Z7XW	CYS247(CB)	4.67	ASP274(C)	1Z7XW		
1Z7XW	CYS247(CB)	4.37	ASP274(O)	1Z7XW		C[ ] ... O[ ]
1Z7XW	CYS247(CB)	4.46	ASP274(CB)	1Z7XW		C[ ] ... C[ ]
1Z7XW	CYS247(SG)	4.61	ASP274(CA)	1Z7XW		
1Z7XW	CYS247(SG)	3.92	ASP274(C)	1Z7XW		S[ ] ... C[ ]
1Z7XW	CYS247(SG)	3.92	ASP274(O)	1Z7XW		S[ ] ... O[ ]
1Z7XW	CYS247(SG)	4.02	ASP274(CB)	1Z7XW	Negative	S[ ] ... C[ ]

1Z7XW	CYS247(SG)	4.01	LEU275(N)	1Z7XW	[D-A-AA]:121.1° [A-D-DD]:85.0° d_planarity:70.9°, maximum distance exceeded, bad d_angle(sp2), bad d_planarity	S[ ] ... N[ ]
1Z7XW	CYS247(SG)	4.15	LEU275(CA)	1Z7XW		S[ ] ... C[ ]
1Z7XW	CYS247(SG)	4.82	LEU275(CB)	1Z7XW		
1Z7XW	CYS247(SG)	4.97	LEU275(CG)	1Z7XW		
1Z7XW	CYS247(SG)	4.00	LEU275(CD1)	1Z7XW		S[ ] ... C[ ]
1Z7XW	CYS247(CA)	4.22	VAL278(CG2)	1Z7XW		C[ ] ... C[ ]
1Z7XW	CYS247(C)	4.60	VAL278(CG2)	1Z7XW		
1Z7XW	CYS247(O)	4.08	VAL278(CG2)	1Z7XW		O[ ] ... C[ ]
1Z7XW	CYS247(CB)	3.79	VAL278(CG2)	1Z7XW		C[ ] ... C[ ]
1Z7XW	CYS247(SG)	4.79	VAL278(CB)	1Z7XW		
1Z7XW	CYS247(SG)	3.70	VAL278(CG2)	1Z7XW		S[ ] ... C[ ]

chain1	res1/atm1	distance	res2/atm2	chain2	H-bonding	Charge interaction
1ZNQO	CYS152(N)	4.54	ALA150(C)	1ZNQO		
1ZNQO	CYS152(N)	4.59	ALA150(O)	1ZNQO	[D-A-AA]:79.7° [A-D-DD]:144.7° d_planarity:57.4° a_planarity:19.2°, maximum distance exceeded, bad a_angle(sp2)	
1ZNQO	CYS152(N)	3.66	SER151(N)	1ZNQO		N[ ] ... N[ ]
1ZNQO	CYS152(N)	2.42	SER151(CA)	1ZNQO		N[ ] ... C[ ]
1ZNQO	CYS152(N)	2.25	SER151(O)	1ZNQO	[D-A-AA]:29.7° [A-D-DD]:93.5° d_planarity:0.4° a_planarity:0.6°, bad a_angle(sp2)	N[ ] ... O[ ]
1ZNQO	CYS152(N)	3.04	SER151(CB)	1ZNQO		N[ ] ... C[ ]
1ZNQO	CYS152(N)	3.38	SER151(OG)	1ZNQO	**H-bond** [D-A-AA]:64.3° [A-D-DD]:124.3° d_planarity:58.9°	N[ ] ... O[ ]
1ZNQO	CYS152(CA)	4.88	SER151(N)	1ZNQO		
1ZNQO	CYS152(CA)	3.79	SER151(CA)	1ZNQO		C[ ] ... C[ ]
1ZNQO	CYS152(CA)	2.42	SER151(C)	1ZNQO		C[ ] ... C[ ]
1ZNQO	CYS152(CA)	2.75	SER151(O)	1ZNQO		C[ ] ... O[ ]
1ZNQO	CYS152(CA)	4.35	SER151(CB)	1ZNQO		C[ ] ... C[ ]
1ZNQO	CYS152(CA)	4.36	SER151(OG)	1ZNQO		C[ ] ... O[ ]
1ZNQO	CYS152(C)	4.30	SER151(CA)	1ZNQO		C[ ] ... C[ ]
1ZNQO	CYS152(C)	2.89	SER151(C)	1ZNQO		C[ ] ... C[ ]
1ZNQO	CYS152(C)	2.76	SER151(O)	1ZNQO		C[ ] ... O[ ]
1ZNQO	CYS152(C)	4.58	SER151(CB)	1ZNQO		
1ZNQO	CYS152(C)	4.10	SER151(OG)	1ZNQO		C[ ] ... O[ ]
1ZNQO	CYS152(O)	3.64	SER151(C)	1ZNQO		O[ ] ... C[ ]
1ZNQO	CYS152(O)	3.11	SER151(O)	1ZNQO		O[ ] ... O[ ]
1ZNQO	CYS152(O)	4.92	SER151(OG)	1ZNQO	[D-A-AA]:42.3° [A-D-DD]:107.9° a_planarity:65.0°, maximum distance exceeded, bad a_angle(sp2)	
1ZNQO	CYS152(CB)	4.88	SER151(CA)	1ZNQO		
1ZNQO	CYS152(CB)	3.74	SER151(C)	1ZNQO		C[ ] ... C[ ]
1ZNQO	CYS152(CB)	4.27	SER151(O)	1ZNQO		C[ ] ... O[ ]
1ZNQO	CYS152(SG)	4.38	SER151(C)	1ZNQO		S[ ] ... C[ ]
1ZNQO	CYS152(SG)	4.99	SER151(CB)	1ZNQO		
1ZNQO	CYS152(N)	2.82	THR153(N)	1ZNQO		N[ ] ... N[ ]

1ZNQO	CYS152(N)	4.22	THR153(CA)	1ZNQO		N[ ] ... C[ ]
1ZNQO	CYS152(N)	4.77	THR153(C)	1ZNQO		
1ZNQO	CYS152(CA)	2.43	THR153(N)	1ZNQO		C[ ] ... N[ ]
1ZNQO	CYS152(CA)	3.80	THR153(CA)	1ZNQO		C[ ] ... C[ ]
1ZNQO	CYS152(CA)	4.50	THR153(C)	1ZNQO		
1ZNQO	CYS152(CA)	4.88	THR153(CB)	1ZNQO		
1ZNQO	CYS152(CA)	4.89	THR153(OG1)	1ZNQO		
1ZNQO	CYS152(C)	2.43	THR153(CA)	1ZNQO		C[ ] ... C[ ]
1ZNQO	CYS152(C)	3.09	THR153(C)	1ZNQO		C[ ] ... C[ ]
1ZNQO	CYS152(C)	3.98	THR153(O)	1ZNQO		C[ ] ... O[ ]
1ZNQO	CYS152(C)	3.72	THR153(CB)	1ZNQO		C[ ] ... C[ ]
1ZNQO	CYS152(C)	4.01	THR153(OG1)	1ZNQO		C[ ] ... O[ ]
1ZNQO	CYS152(C)	4.91	THR153(CG2)	1ZNQO		
1ZNQO	CYS152(O)	2.24	THR153(N)	1ZNQO	[D-A-AA]:29.9° [A-D-DD]:93.7° d_planarity:1.3° a_planarity:1.6°, bad a_angle(sp2)	O[ ] ... N[ ]
1ZNQO	CYS152(O)	2.75	THR153(CA)	1ZNQO		O[ ] ... C[ ]
1ZNQO	CYS152(O)	3.01	THR153(C)	1ZNQO		O[ ] ... C[ ]
1ZNQO	CYS152(O)	3.57	THR153(O)	1ZNQO		O[ ] ... O[ ]
1ZNQO	CYS152(O)	4.26	THR153(CB)	1ZNQO		O[ ] ... C[ ]
1ZNQO	CYS152(O)	4.73	THR153(OG1)	1ZNQO	[D-A-AA]:47.7° [A-D-DD]:62.2° a_planarity:21.2°, maximum distance exceeded, bad a_angle(sp2)	
1ZNQO	CYS152(CB)	3.09	THR153(N)	1ZNQO		C[ ] ... N[ ]
1ZNQO	CYS152(CB)	4.41	THR153(CA)	1ZNQO		C[ ] ... C[ ]
1ZNQO	CYS152(CB)	4.82	THR153(OG1)	1ZNQO		
1ZNQO	CYS152(SG)	3.39	THR153(N)	1ZNQO	**H-bond** [D-A-AA]:64.8° [A-D-DD]:139.4° d_planarity:-57.0°	S[ ] ... N[ ]
1ZNQO	CYS152(SG)	4.59	THR153(CA)	1ZNQO		
1ZNQO	CYS152(SG)	4.87	THR153(CB)	1ZNQO		
1ZNQO	CYS152(SG)	4.08	THR153(OG1)	1ZNQO	[D-A-AA]:102.8° [A-D-DD]:115.6°, maximum distance exceeded	S[ ] ... O[ ]
1ZNQO	CYS152(N)	4.38	THR154(N)	1ZNQO		N[ ] ... N[ ]
1ZNQO	CYS152(CA)	4.48	THR154(N)	1ZNQO		C[ ] ... N[ ]
1ZNQO	CYS152(C)	3.34	THR154(N)	1ZNQO		C[ ] ... N[ ]
1ZNQO	CYS152(C)	4.56	THR154(CA)	1ZNQO		
1ZNQO	CYS152(C)	4.67	THR154(C)	1ZNQO		
1ZNQO	CYS152(O)	3.43	THR154(N)	1ZNQO	[D-A-AA]:75.9° [A-D-DD]:123.6° d_planarity:73.1° a_planarity:46.6°, bad a_angle(sp2), bad d_planarity	O[ ] ... N[ ]
1ZNQO	CYS152(O)	4.40	THR154(CA)	1ZNQO		O[ ] ... C[ ]
1ZNQO	CYS152(O)	4.14	THR154(C)	1ZNQO		O[ ] ... C[ ]
1ZNQO	CYS152(N)	4.97	ASN155(N)	1ZNQO		
1ZNQO	CYS152(N)	4.86	ASN155(OD1)	1ZNQO	[D-A-AA]:117.0° [A-D-DD]:87.1° d_planarity:29.1° a_planarity:25.5°, maximum distance exceeded, bad d_angle(sp2)	
1ZNQO	CYS152(CA)	4.76	ASN155(N)	1ZNQO		
1ZNQO	CYS152(CA)	4.82	ASN155(CB)	1ZNQO		
1ZNQO	CYS152(C)	3.88	ASN155(N)	1ZNQO		C[ ] ... N[ ]
1ZNQO	CYS152(C)	4.65	ASN155(CA)	1ZNQO		
1ZNQO	CYS152(C)	4.48	ASN155(CB)	1ZNQO		C[ ] ... C[ ]
1ZNQO	CYS152(O)	3.24	ASN155(N)	1ZNQO	**H-bond** [D-A-AA]:112.6° [A-D-DD]:98.9° d_planarity:-41.3° a_planarity:79.4°	O[ ] ... N[ ]
1ZNQO	CYS152(O)	3.75	ASN155(CA)	1ZNQO		O[ ] ... C[ ]

1ZNQO	CYS152(O)	3.90	ASN155(C)	1ZNQO		O[ ] ... C[ ]
1ZNQO	CYS152(O)	4.93	ASN155(O)	1ZNQO		
1ZNQO	CYS152(O)	3.65	ASN155(CB)	1ZNQO		O[ ] ... C[ ]
1ZNQO	CYS152(O)	4.76	ASN155(CG)	1ZNQO		
1ZNQO	CYS152(O)	4.84	ASN155(OD1)	1ZNQO		
1ZNQO	CYS152(C)	4.48	CYS156(N)	1ZNQO		C[ ] ... N[ ]
1ZNQO	CYS152(C)	4.97	CYS156(CB)	1ZNQO		
1ZNQO	CYS152(O)	3.37	CYS156(N)	1ZNQO	**H-bond** [D-A-AA]:150.4° [A-D-DD]:118.7° d_planarity:-50.7° a_planarity:37.9°	O[ ] ... N[ ]
1ZNQO	CYS152(O)	4.26	CYS156(CA)	1ZNQO		O[ ] ... C[ ]
1ZNQO	CYS152(O)	3.99	CYS156(CB)	1ZNQO		O[ ] ... C[ ]
1ZNQO	CYS152(CB)	4.73	HIS179(CE1)	1ZNQO		
1ZNQO	CYS152(SG)	4.55	HIS179(ND1)	1ZNQO	[D-A-AA]:122.5° [A-D-DD]:137.6° d_planarity:20.1°, maximum distance exceeded	
1ZNQO	CYS152(SG)	3.50	HIS179(CE1)	1ZNQO		S[ ] ... C[ ]
1ZNQO	CYS152(SG)	4.18	HIS179(NE2)	1ZNQO	Weakly positive	S[ ] ... N[ ]
1ZNQO	CYS152(O)	5.00	SER292(O)	1ZNQO		
1ZNQO	CYS152(CA)	4.94	TYR314(CD2)	1ZNQO		
1ZNQO	CYS152(CA)	4.55	TYR314(CE2)	1ZNQO		
1ZNQO	CYS152(C)	4.22	TYR314(CD2)	1ZNQO		C[ ] ... C[ ]
1ZNQO	CYS152(C)	3.73	TYR314(CE2)	1ZNQO		C[ ] ... C[ ]
1ZNQO	CYS152(C)	4.68	TYR314(CZ)	1ZNQO		
1ZNQO	CYS152(C)	4.86	TYR314(OH)	1ZNQO		
1ZNQO	CYS152(O)	4.86	TYR314(CG)	1ZNQO		
1ZNQO	CYS152(O)	3.80	TYR314(CD2)	1ZNQO		O[ ] ... C[ ]
1ZNQO	CYS152(O)	3.65	TYR314(CE2)	1ZNQO		O[ ] ... C[ ]
1ZNQO	CYS152(O)	4.63	TYR314(CZ)	1ZNQO		
1ZNQO	CYS152(CB)	4.58	TYR314(CD2)	1ZNQO		
1ZNQO	CYS152(CB)	4.12	TYR314(CE2)	1ZNQO		C[ ] ... C[ ]
1ZNQO	CYS152(SG)	4.60	TYR314(CE2)	1ZNQO		
1ZNQO	CYS152(CA)	4.93	ASN316(O)	1ZNQO		
1ZNQO	CYS152(CB)	4.18	ASN316(CA)	1ZNQO		C[ ] ... C[ ]
1ZNQO	CYS152(CB)	4.45	ASN316(C)	1ZNQO		C[ ] ... C[ ]
1ZNQO	CYS152(CB)	3.78	ASN316(O)	1ZNQO		C[ ] ... O[ ]
1ZNQO	CYS152(CB)	3.97	ASN316(CB)	1ZNQO		C[ ] ... C[ ]
1ZNQO	CYS152(SG)	4.93	ASN316(CA)	1ZNQO		
1ZNQO	CYS152(SG)	4.67	ASN316(O)	1ZNQO		
1ZNQO	CYS152(SG)	4.14	ASN316(CB)	1ZNQO		S[ ] ... C[ ]
1ZNQO	CYS152(N)	4.52	TYR320(CG)	1ZNQO		
1ZNQO	CYS152(N)	3.73	TYR320(CD1)	1ZNQO		N[ ] ... C[ ]
1ZNQO	CYS152(N)	3.88	TYR320(CE1)	1ZNQO		N[ ] ... C[ ]
1ZNQO	CYS152(N)	4.77	TYR320(CZ)	1ZNQO		
1ZNQO	CYS152(CA)	4.51	TYR320(CB)	1ZNQO		
1ZNQO	CYS152(CA)	4.35	TYR320(CG)	1ZNQO		C[ ] ... C[ ]
1ZNQO	CYS152(CA)	3.73	TYR320(CD1)	1ZNQO		C[ ] ... C[ ]
1ZNQO	CYS152(CA)	4.36	TYR320(CE1)	1ZNQO		C[ ] ... C[ ]
1ZNQO	CYS152(C)	4.92	TYR320(CD1)	1ZNQO		
1ZNQO	CYS152(CB)	4.48	TYR320(CB)	1ZNQO		C[ ] ... C[ ]
1ZNQO	CYS152(CB)	4.62	TYR320(CG)	1ZNQO		

1ZNQO	CYS152(CB)	4.45	TYR320(CD1)	1ZNQO		C[ ] ... C[ ]
1ZNQO	CYS152(N)	4.36	NAD1001(C4N)	1ZNQO		N[ ] ... C[ ]
1ZNQO	CYS152(N)	3.48	NAD1001(C5N)	1ZNQO		N[ ] ... C[ ]
1ZNQO	CYS152(N)	4.17	NAD1001(C6N)	1ZNQO		N[ ] ... C[ ]
1ZNQO	CYS152(CA)	4.33	NAD1001(C4N)	1ZNQO		C[ ] ... C[ ]
1ZNQO	CYS152(CA)	3.89	NAD1001(C5N)	1ZNQO		C[ ] ... C[ ]
1ZNQO	CYS152(CA)	4.91	NAD1001(C6N)	1ZNQO		
1ZNQO	CYS152(CB)	4.71	NAD1001(C3N)	1ZNQO		
1ZNQO	CYS152(CB)	3.31	NAD1001(C4N)	1ZNQO		C[ ] ... C[ ]
1ZNQO	CYS152(CB)	3.39	NAD1001(C5N)	1ZNQO		C[ ] ... C[ ]
1ZNQO	CYS152(CB)	4.60	NAD1001(C6N)	1ZNQO		
1ZNQO	CYS152(SG)	4.27	NAD1001(C3N)	1ZNQO		S[ ] ... C[ ]
1ZNQO	CYS152(SG)	4.86	NAD1001(O7N)	1ZNQO	[D-A-AA]:91.5° [A-D-DD]:89.5°, maximum distance exceeded	
1ZNQO	CYS152(SG)	3.26	NAD1001(C4N)	1ZNQO		S[ ] ... C[ ]
1ZNQO	CYS152(SG)	3.45	NAD1001(C5N)	1ZNQO		S[ ] ... C[ ]
1ZNQO	CYS152(SG)	4.40	NAD1001(C6N)	1ZNQO	Ligand	S[ ] ... C[ ]

hain1	res1/atm1	distance	res2/atm2	chain2	H-bonding	Charge interaction
1ZNQO	CYS247(SG)	4.58	ILE164(CG2)	1ZNQO		
1ZNQO	CYS247(CB)	4.81	ILE170(CG1)	1ZNQO		
1ZNQO	CYS247(CB)	4.18	ILE170(CG2)	1ZNQO		C[ ] ... C[ ]
1ZNQO	CYS247(SG)	4.68	ILE170(CG1)	1ZNQO		
1ZNQO	CYS247(SG)	5.00	ILE170(CG2)	1ZNQO		
1ZNQO	CYS247(SG)	4.84	ILE170(CD1)	1ZNQO		
1ZNQO	CYS247(N)	4.58	GLU172(O)	1ZNQO	[D-A-AA]:136.0° [A-D-DD]:24.0° d_planarity:8.5° a_planarity:0.7°, maximum distance exceeded, bad d_angle(sp2)	
1ZNQO	CYS247(CA)	4.37	GLU172(C)	1ZNQO		C[ ] ... C[ ]
1ZNQO	CYS247(CA)	3.31	GLU172(O)	1ZNQO		C[ ] ... O[ ]
1ZNQO	CYS247(C)	4.86	GLU172(C)	1ZNQO		
1ZNQO	CYS247(C)	3.64	GLU172(O)	1ZNQO		C[ ] ... O[ ]
1ZNQO	CYS247(O)	4.87	GLU172(O)	1ZNQO		
1ZNQO	CYS247(CB)	4.32	GLU172(C)	1ZNQO		C[ ] ... C[ ]
1ZNQO	CYS247(CB)	3.41	GLU172(O)	1ZNQO		C[ ] ... O[ ]
1ZNQO	CYS247(N)	4.66	GLY173(CA)	1ZNQO		
1ZNQO	CYS247(CA)	4.72	GLY173(N)	1ZNQO		
1ZNQO	CYS247(CA)	4.04	GLY173(CA)	1ZNQO		C[ ] ... C[ ]
1ZNQO	CYS247(CB)	4.59	GLY173(N)	1ZNQO		
1ZNQO	CYS247(CB)	3.95	GLY173(CA)	1ZNQO		C[ ] ... C[ ]
1ZNQO	CYS247(N)	4.02	LEU245(C)	1ZNQO		N[ ] ... C[ ]
1ZNQO	CYS247(N)	4.03	LEU245(O)	1ZNQO	[D-A-AA]:80.7° [A-D-DD]:145.2° d_planarity:-86.2° a_planarity:33.2°, maximum distance exceeded, bad a_angle(sp2), bad d_planarity	N[ ] ... O[ ]
1ZNQO	CYS247(N)	4.78	LEU245(CG)	1ZNQO		
1ZNQO	CYS247(N)	4.90	LEU245(CD1)	1ZNQO		
1ZNQO	CYS247(CB)	4.50	LEU245(CG)	1ZNQO		C[ ] ... C[ ]
1ZNQO	CYS247(CB)	4.51	LEU245(CD1)	1ZNQO		

1ZNQO	CYS247(CB)	4.94	LEU245(CD2)	1ZNQO		
1ZNQO	CYS247(SG)	4.78	LEU245(O)	1ZNQO		
1ZNQO	CYS247(SG)	4.20	LEU245(CG)	1ZNQO		S[ ] ... C[ ]
1ZNQO	CYS247(SG)	4.80	LEU245(CD1)	1ZNQO		
1ZNQO	CYS247(SG)	4.33	LEU245(CD2)	1ZNQO		S[ ] ... C[ ]
1ZNQO	CYS247(N)	3.38	THR246(N)	1ZNQO		N[ ] ... N[ ]
1ZNQO	CYS247(N)	2.44	THR246(CA)	1ZNQO		N[ ] ... C[ ]
1ZNQO	CYS247(N)	2.25	THR246(O)	1ZNQO	[D-A-AA]:29.9° [A-D-DD]:92.5° d_planarity:1.2° a_planarity:0.5°, bad a_angle(sp2)	N[ ] ... O[ ]
1ZNQO	CYS247(N)	3.49	THR246(CB)	1ZNQO		N[ ] ... C[ ]
1ZNQO	CYS247(N)	4.68	THR246(OG1)	1ZNQO	[D-A-AA]:28.0° [A-D-DD]:153.4° d_planarity:31.7°, maximum distance exceeded, bad a_angle(sp3)	
1ZNQO	CYS247(N)	3.55	THR246(CG2)	1ZNQO		N[ ] ... C[ ]
1ZNQO	CYS247(CA)	4.60	THR246(N)	1ZNQO		
1ZNQO	CYS247(CA)	3.80	THR246(CA)	1ZNQO		C[ ] ... C[ ]
1ZNQO	CYS247(CA)	2.41	THR246(C)	1ZNQO		C[ ] ... C[ ]
1ZNQO	CYS247(CA)	2.73	THR246(O)	1ZNQO		C[ ] ... O[ ]
1ZNQO	CYS247(CA)	4.72	THR246(CB)	1ZNQO		
1ZNQO	CYS247(CA)	4.64	THR246(CG2)	1ZNQO		
1ZNQO	CYS247(C)	4.91	THR246(CA)	1ZNQO		
1ZNQO	CYS247(C)	3.68	THR246(C)	1ZNQO		C[ ] ... C[ ]
1ZNQO	CYS247(C)	4.10	THR246(O)	1ZNQO		C[ ] ... O[ ]
1ZNQO	CYS247(O)	4.11	THR246(C)	1ZNQO		O[ ] ... C[ ]
1ZNQO	CYS247(O)	4.80	THR246(O)	1ZNQO		
1ZNQO	CYS247(CB)	4.89	THR246(N)	1ZNQO		
1ZNQO	CYS247(CB)	4.50	THR246(CA)	1ZNQO		
1ZNQO	CYS247(CB)	3.21	THR246(C)	1ZNQO		C[ ] ... C[ ]
1ZNQO	CYS247(CB)	3.42	THR246(O)	1ZNQO		C[ ] ... O[ ]
1ZNQO	CYS247(SG)	4.90	THR246(CA)	1ZNQO		
1ZNQO	CYS247(SG)	4.05	THR246(C)	1ZNQO		S[ ] ... C[ ]
1ZNQO	CYS247(SG)	4.66	THR246(O)	1ZNQO		
1ZNQO	CYS247(N)	3.67	ARG248(N)	1ZNQO		N[ ] ... N[ ]
1ZNQO	CYS247(N)	4.94	ARG248(CA)	1ZNQO		
1ZNQO	CYS247(CA)	2.40	ARG248(N)	1ZNQO		C[ ] ... N[ ]
1ZNQO	CYS247(CA)	3.78	ARG248(CA)	1ZNQO		C[ ] ... C[ ]
1ZNQO	CYS247(CA)	4.50	ARG248(C)	1ZNQO		C[ ] ... C[ ]
1ZNQO	CYS247(CA)	4.68	ARG248(O)	1ZNQO		
1ZNQO	CYS247(CA)	4.79	ARG248(CB)	1ZNQO		
1ZNQO	CYS247(C)	2.44	ARG248(CA)	1ZNQO		C[ ] ... C[ ]
1ZNQO	CYS247(C)	3.28	ARG248(C)	1ZNQO		C[ ] ... C[ ]
1ZNQO	CYS247(C)	3.78	ARG248(O)	1ZNQO		C[ ] ... O[ ]
1ZNQO	CYS247(C)	3.62	ARG248(CB)	1ZNQO		C[ ] ... C[ ]
1ZNQO	CYS247(C)	4.83	ARG248(CG)	1ZNQO		
1ZNQO	CYS247(O)	2.28	ARG248(N)	1ZNQO	[D-A-AA]:28.8° [A-D-DD]:94.9° d_planarity:1.1° a_planarity:0.3°, bad a_angle(sp2)	O[ ] ... N[ ]
1ZNQO	CYS247(O)	2.81	ARG248(CA)	1ZNQO		O[ ] ... C[ ]
1ZNQO	CYS247(O)	3.60	ARG248(C)	1ZNQO		O[ ] ... C[ ]
1ZNQO	CYS247(O)	4.38	ARG248(O)	1ZNQO		O[ ] ... O[ ]
1ZNQO	CYS247(O)	4.10	ARG248(CB)	1ZNQO		O[ ] ... C[ ]
1ZNQO	CYS247(CB)	3.08	ARG248(N)	1ZNQO		C[ ] ... N[ ]

1ZNQO	CYS247(CB)	4.38	ARG248(CA)	1ZNQO		C[ ] ... C[ ]
1ZNQO	CYS247(CB)	4.61	ARG248(C)	1ZNQO		
1ZNQO	CYS247(CB)	4.49	ARG248(O)	1ZNQO		C[ ] ... O[ ]
1ZNQO	CYS247(SG)	4.19	ARG248(N)	1ZNQO	[D-A-AA]:41.9° [A-D-DD]:127.0° d_planarity:52.1°, maximum distance exceeded, bad a_angle(sp3)	S[ ] ... N[ ]
1ZNQO	CYS247(C)	4.03	LEU249(N)	1ZNQO		C[ ] ... N[ ]
1ZNQO	CYS247(O)	3.96	LEU249(N)	1ZNQO	[D-A-AA]:84.3° [A-D-DD]:139.7° d_planarity:87.4° a_planarity:31.4°, maximum distance exceeded, bad a_angle(sp2), bad d_planarity	O[ ] ... N[ ]
1ZNQO	CYS247(O)	4.63	LEU249(CG)	1ZNQO		
1ZNQO	CYS247(O)	4.88	LEU249(CD2)	1ZNQO		
1ZNQO	CYS247(SG)	4.95	LEU249(CG)	1ZNQO		
1ZNQO	CYS247(SG)	4.15	LEU249(CD2)	1ZNQO		S[ ] ... C[ ]
1ZNQO	CYS247(C)	4.12	HIS306(O)	1ZNQO		C[ ] ... O[ ]
1ZNQO	CYS247(O)	4.24	HIS306(C)	1ZNQO		O[ ] ... C[ ]
1ZNQO	CYS247(O)	3.42	HIS306(O)	1ZNQO		O[ ] ... O[ ]
1ZNQO	CYS247(N)	4.96	PHE307(CD1)	1ZNQO		
1ZNQO	CYS247(N)	4.86	PHE307(CE1)	1ZNQO		
1ZNQO	CYS247(CA)	4.98	PHE307(CD1)	1ZNQO		
1ZNQO	CYS247(C)	4.64	PHE307(CA)	1ZNQO		
1ZNQO	CYS247(C)	4.14	PHE307(CD1)	1ZNQO		C[ ] ... C[ ]
1ZNQO	CYS247(C)	4.54	PHE307(CE1)	1ZNQO		
1ZNQO	CYS247(O)	4.34	PHE307(N)	1ZNQO	[D-A-AA]:143.0° [A-D-DD]:49.5° d_planarity:27.2° a_planarity:15.1°, maximum distance exceeded, bad d_angle(sp2)	O[ ] ... N[ ]
1ZNQO	CYS247(O)	3.57	PHE307(CA)	1ZNQO		O[ ] ... C[ ]
1ZNQO	CYS247(O)	3.89	PHE307(C)	1ZNQO		O[ ] ... C[ ]
1ZNQO	CYS247(O)	4.60	PHE307(CB)	1ZNQO		
1ZNQO	CYS247(O)	4.52	PHE307(CG)	1ZNQO		
1ZNQO	CYS247(O)	3.70	PHE307(CD1)	1ZNQO		O[ ] ... C[ ]
1ZNQO	CYS247(O)	4.38	PHE307(CE1)	1ZNQO		O[ ] ... C[ ]
1ZNQO	CYS247(N)	4.68	VAL308(N)	1ZNQO		
1ZNQO	CYS247(N)	4.31	VAL308(C)	1ZNQO		N[ ] ... C[ ]
1ZNQO	CYS247(N)	3.10	VAL308(O)	1ZNQO	**H-bond** [D-A-AA]:167.1° [A-D-DD]:131.4° d_planarity:-4.1° a_planarity:-20.8°	N[ ] ... O[ ]
1ZNQO	CYS247(CA)	4.20	VAL308(O)	1ZNQO		C[ ] ... O[ ]
1ZNQO	CYS247(C)	4.39	VAL308(N)	1ZNQO		C[ ] ... N[ ]
1ZNQO	CYS247(C)	4.39	VAL308(O)	1ZNQO		C[ ] ... O[ ]
1ZNQO	CYS247(O)	3.20	VAL308(N)	1ZNQO	**H-bond** [D-A-AA]:162.2° [A-D-DD]:128.7° d_planarity:-2.8° a_planarity:-33.9°	O[ ] ... N[ ]
1ZNQO	CYS247(O)	4.26	VAL308(CA)	1ZNQO		O[ ] ... C[ ]
1ZNQO	CYS247(O)	4.43	VAL308(C)	1ZNQO		O[ ] ... C[ ]
1ZNQO	CYS247(O)	3.69	VAL308(O)	1ZNQO		O[ ] ... O[ ]
1ZNQO	CYS247(O)	4.87	VAL308(CB)	1ZNQO		
1ZNQO	CYS247(O)	4.29	VAL308(CG1)	1ZNQO		O[ ] ... C[ ]
1ZNQO	CYS247(CB)	4.86	VAL308(O)	1ZNQO		
1ZNQO	CYS247(SG)	4.30	VAL308(O)	1ZNQO		S[ ] ... O[ ]
1ZNQO	CYS247(SG)	4.43	VAL308(CG1)	1ZNQO		S[ ] ... C[ ]
1ZNQO	CYS247(SG)	4.79	LEU310(CD1)	1ZNQO		
1ZNQO	CYS247(CA)	4.78	PHE307(CZ)	1ZNQP		
