# Supplementary material for: Comprehensive Identification and Modified-Site Mapping of S-Nitrosylated Targets in Prostate Epithelial Cells
Source: PLoS One. 2010 Feb 5;5(2):e9075. doi: 10.1371/journal.pone.0009075 (PMC2816712; doi:10.1371/journal.pone.0009075)

(MS/MS spectra for biotinylated peptides in Table S1A)

PDLIM1 (elfin) (K)GHFFVEDQIYcEK(H)
XCorr: 4.46 ΔCn: 0.62

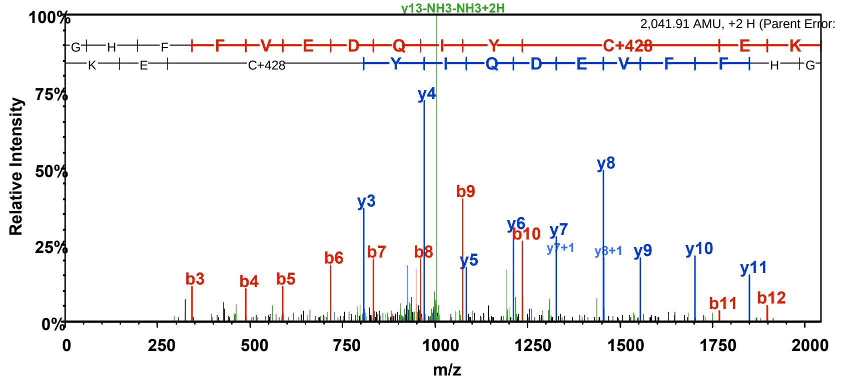

TUBA1C protein (R)TIQFVDWcPTGFK(V)
XCorr: 3.2 ΔCn: 0.48

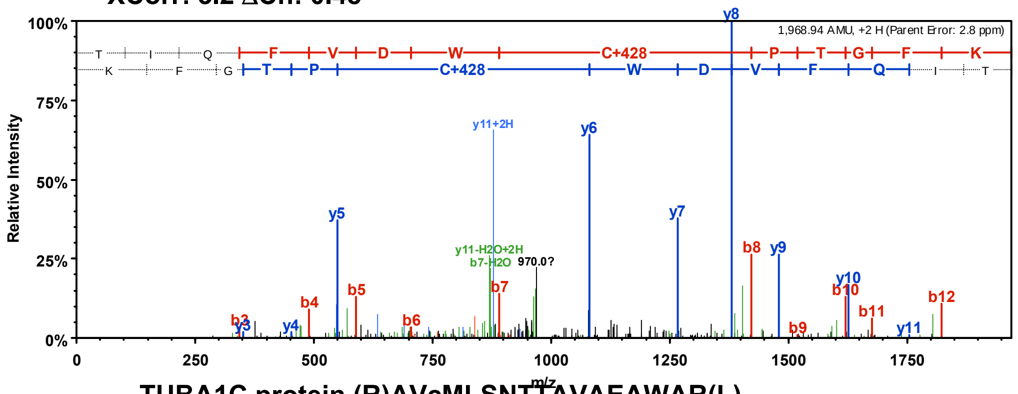

TUBB2C(R)EIVHLQAGGQcGNQIGAK(F)
XCorr: 3.8483 ΔCn:0.479

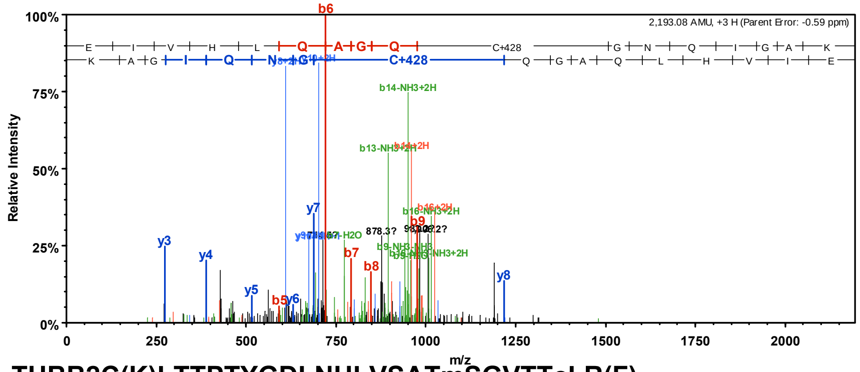

TUBA1C protein (R)AVcMLSNTTAVAEAWAR(L)
XCorr: 2.59 ΔCn: 0.30

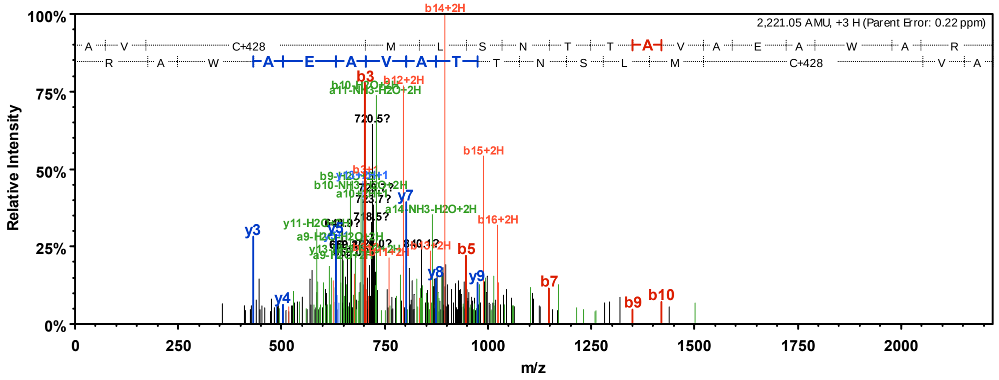

TUBB2C(K)LTTPTYGDLNHLVSATmSGVTTcLR(F)
XCorr: 5.01 ΔCn: 0.581

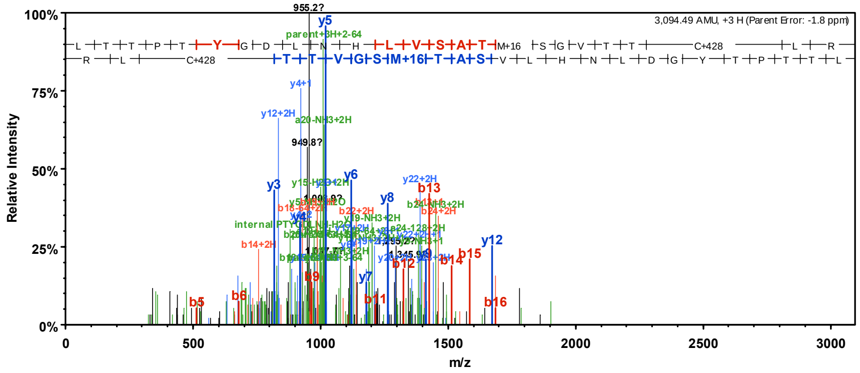

TUBA1C protein (K)AYHEQLTVAEITNAcFEPANQmVK(C)
XCorr: 2.74 ΔCn: 0.15

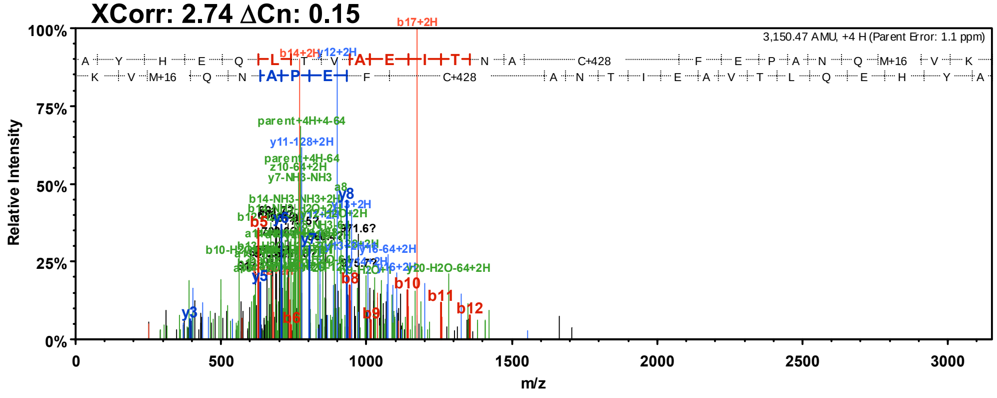

Putative annexin A2-like protein (K)GLGTDEDSLIEIIcSR(T)
XCorr: 4.16 ΔCn: 0.46

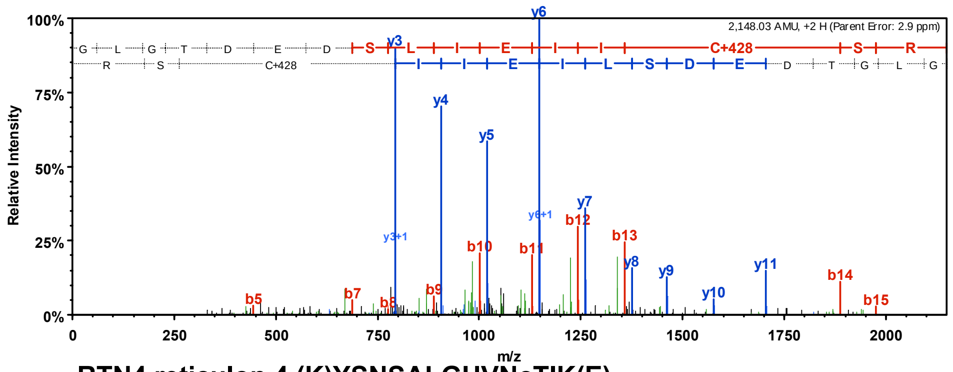

Isoform 1 of Plectin-1 (R)VPLDVAcAR(G)
XCorr: 2.3 ΔCn: 0.36

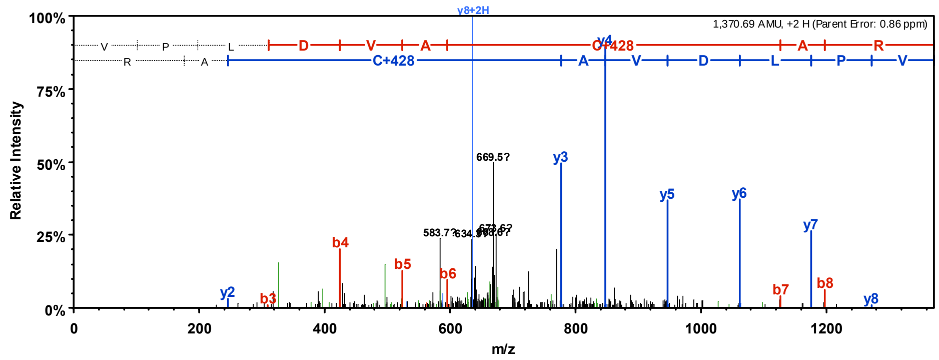

RTN4 reticulon 4 (K)YSNSALGHVNcTIK(E)
XCorr: 3.29 ΔCn: 0.51

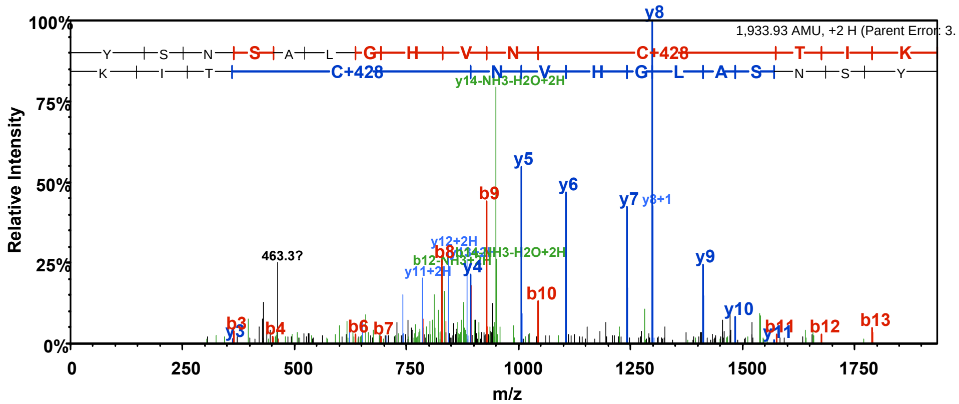

Isoform 1 of Plectin-1 (R)GcLDEETSR(A)
XCorr: 2.59 ΔCn: 0.43

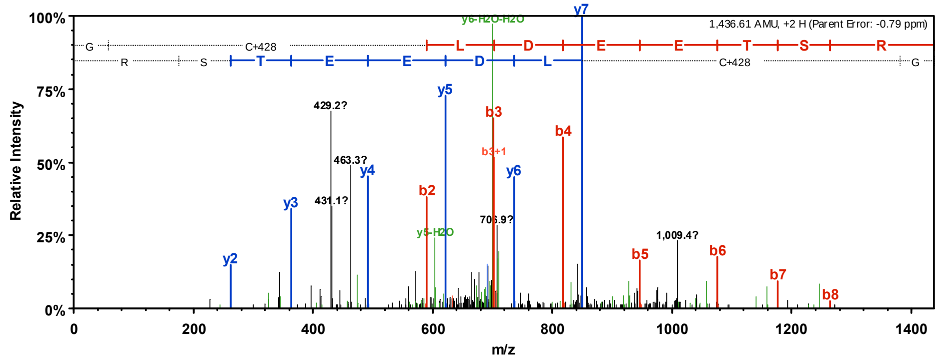

PLEC1, Plectin 1 (R)VPLDVAcAR(G)
XCorr: 2.67 ΔCn: 0.63

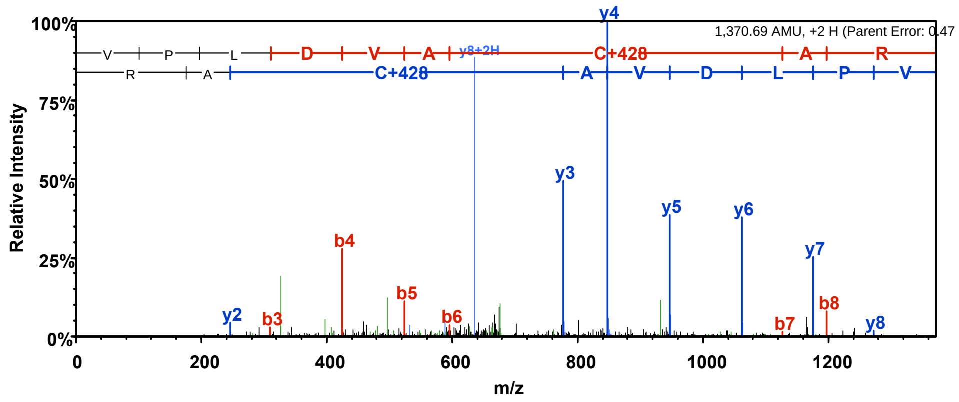

Isoform 1 of Plectin-1 (R)cRPDQLTGLSLLPLSEK(A)
XCorr: 3.64 ΔCn: 0.39

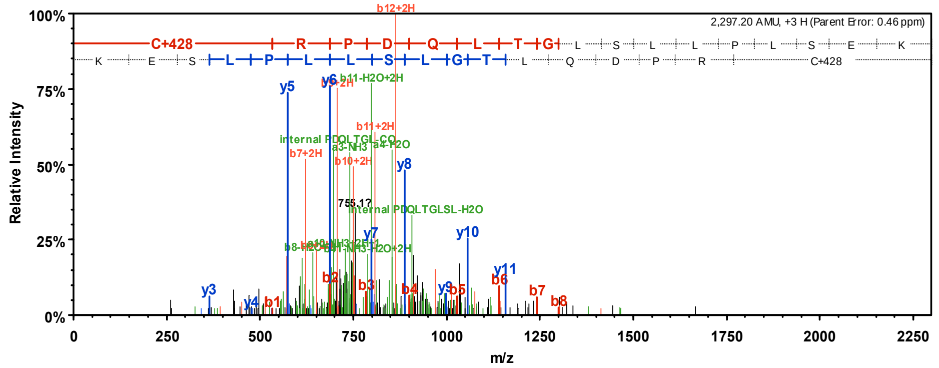

PLEC1 Plectin 1 (R)LQLEAcETR(T)
XCorr: 2.68 ΔCn: 0.48

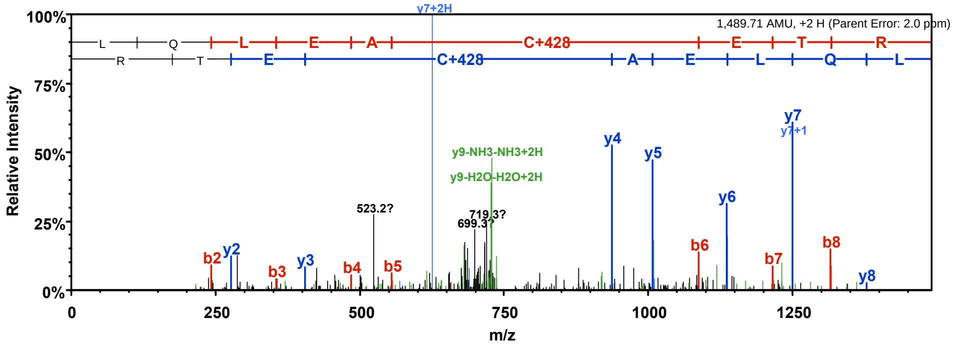

ACTN1 alpha actinin 1 (K)IcDQWDNLGALTQK(R)
XCorr: 4.83 ΔCn: 0.54

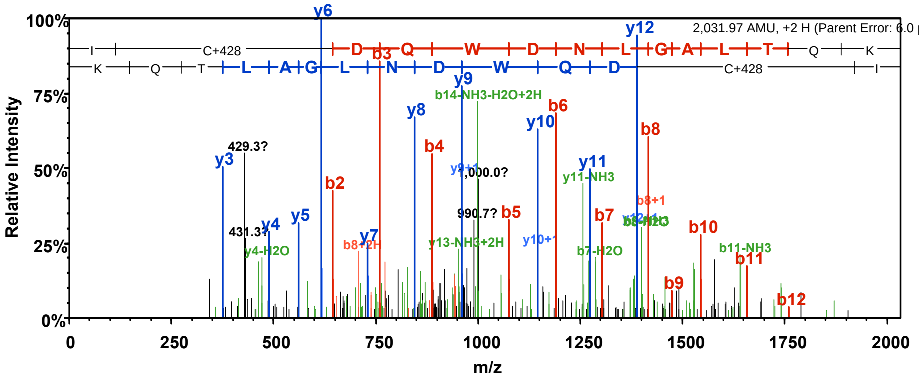

Isoform 1 of Plectin-1 (R)EYGScSHHYQQLLQSLEQGAQEESR(C)
XCorr: 4.00 ΔCn: 0.48

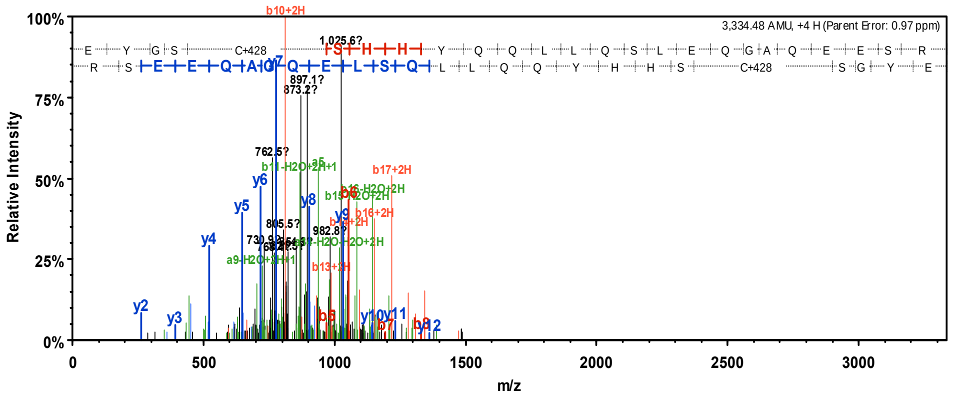

ACTN1 alpha actinin 1 (K)cQLEINFNTLQTK(L)
XCorr: 3.09 ΔCn: 0.57

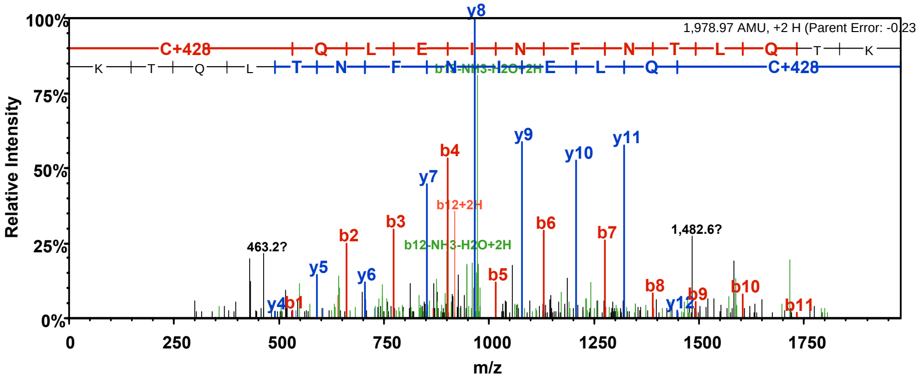

Isoform 1 of Plectin-1 (R)LPLLAVcDYK(Q)
XCorr: 2.4 ΔCn: 0.39

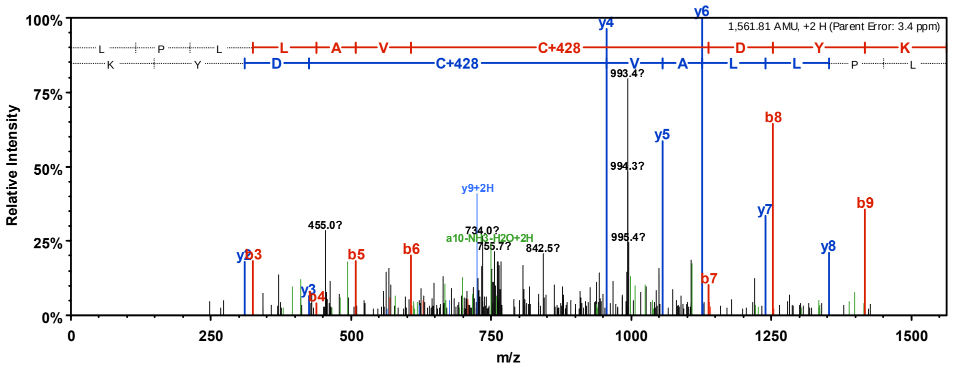

Citrate synthase, mitochondrial (K)LPcVAAK(I)
XCorr: 2.62 ΔCn: 0.35

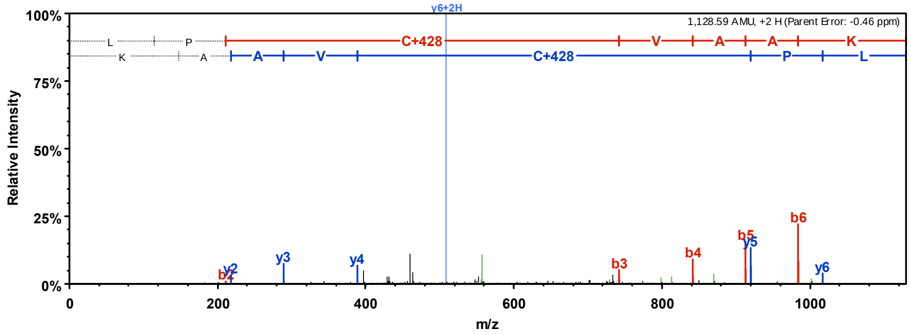

VCP valosin-containing protein (K)GVLFYGPPGcGK(T)
XCorr: 3.39 ΔCn: 0.59

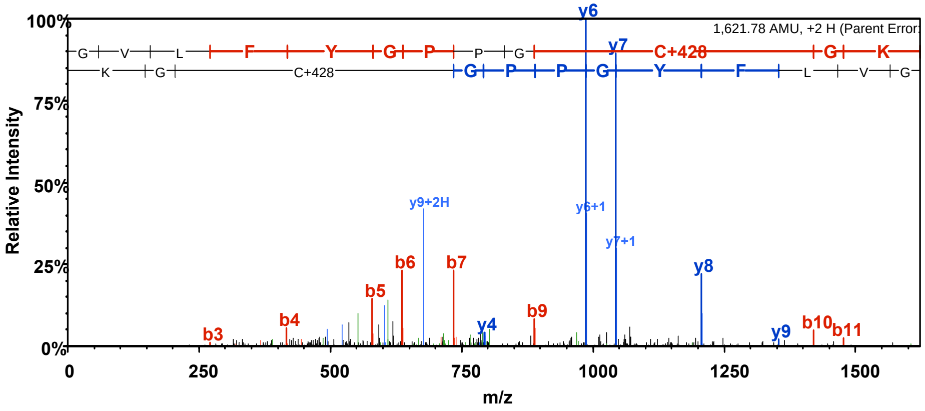

PGK1 phosphoglycerate kinase 1(K)DcVGPEVEK(A)
XCorr: 2.14 ΔCn: 0.39

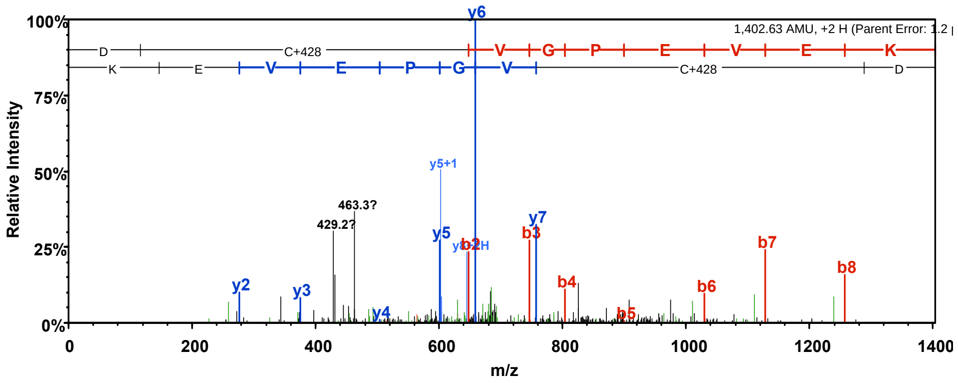

VCP valosin-containing protein (R)LGDVISIQPcPDVK(Y)
XCorr: 3.75 ΔCn: 0.6

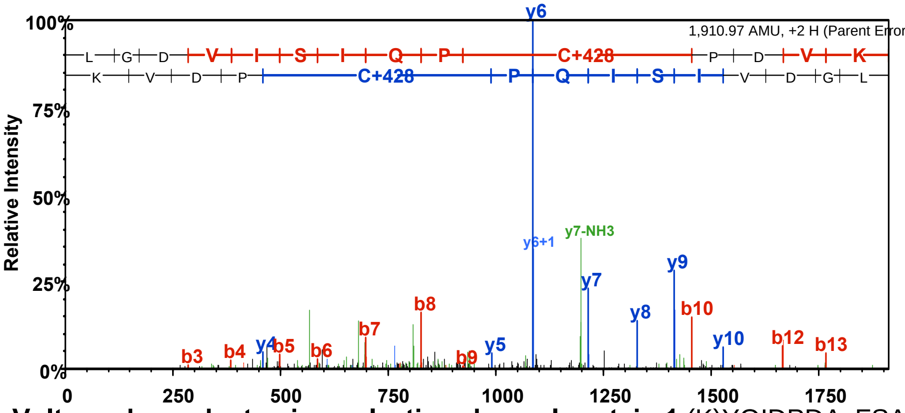

PGK1 phosphoglycerate kinase 1 (K)AcANPAAGSVILLENLR(F)
XCorr: 5.16 ΔCn: 0.57

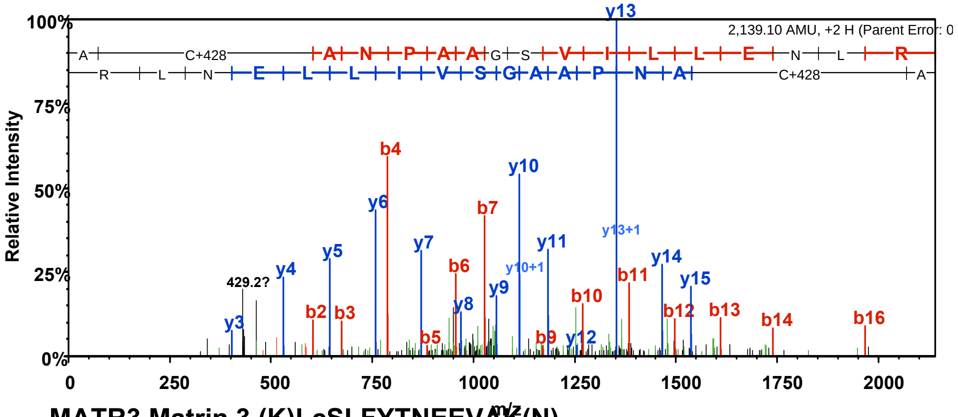

Voltage-dependent anion-selective channel protein 1 (K)YQIDPDAcFSAK
XCorr: 3.23 ΔCn: 0.53

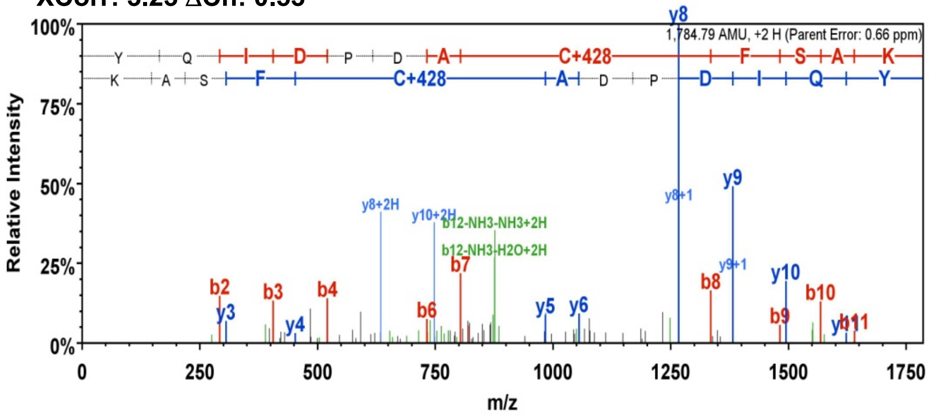

MATR3 Matrin 3 (K)LcSLFYTNEEVAK(N)
XCorr: 3.43 ΔCn: 0.59

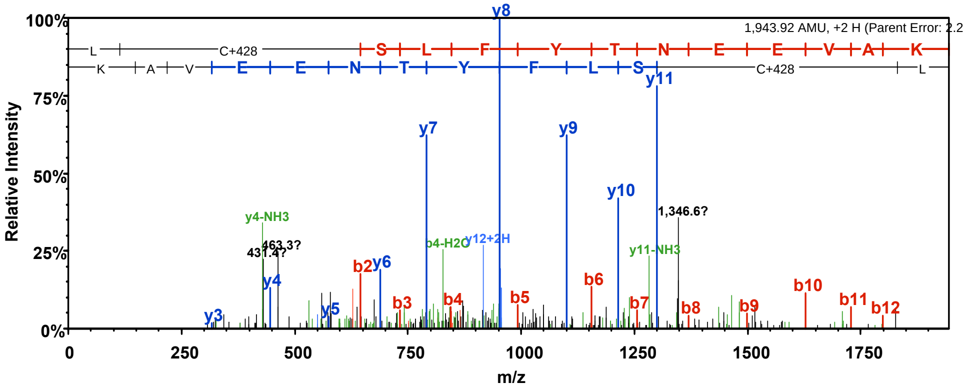

Heterogeneous nuclear ribonucleoprotein R (R)GFcFLEYEDHK(S)
XCorr: 3.53 ΔCn: 0.31

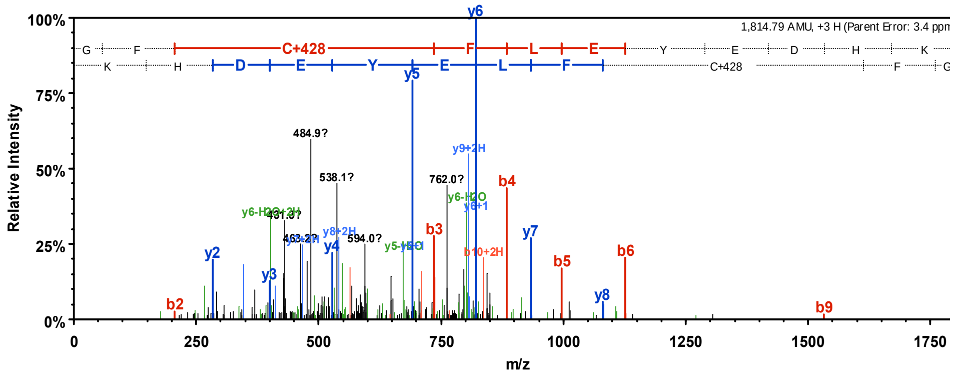

HNRPDL (R)GFcFITYTDEEPVKK(L)
XCorr: 3.62 ΔCn: 0.55

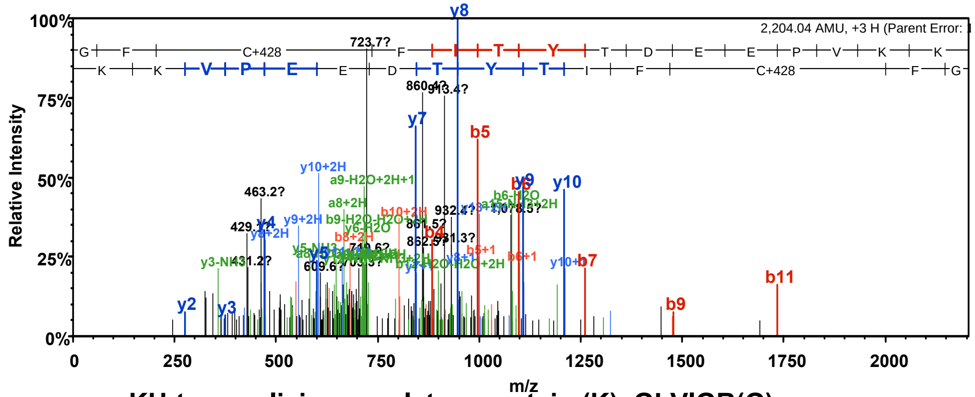

KH-type splicing regulatory protein (K)cGLVIGR(G)
XCorr: 2.02 ΔCn: 0.18

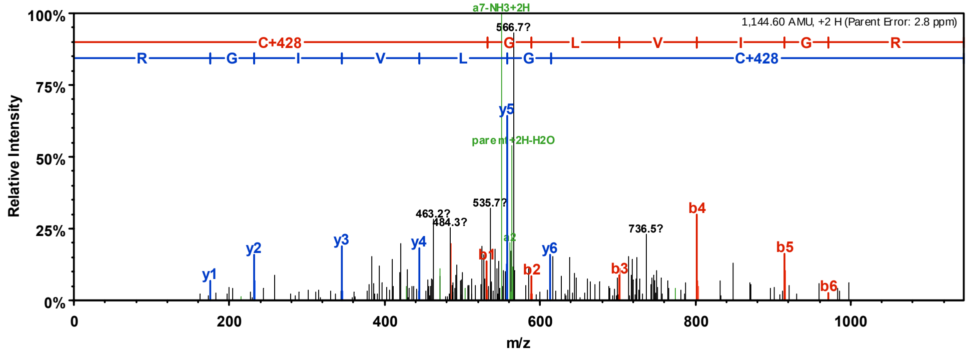

KH-type splicing regulatory protein (K)IQQDSGcK(V)
XCorr: 2.59 ΔCn: 0.33

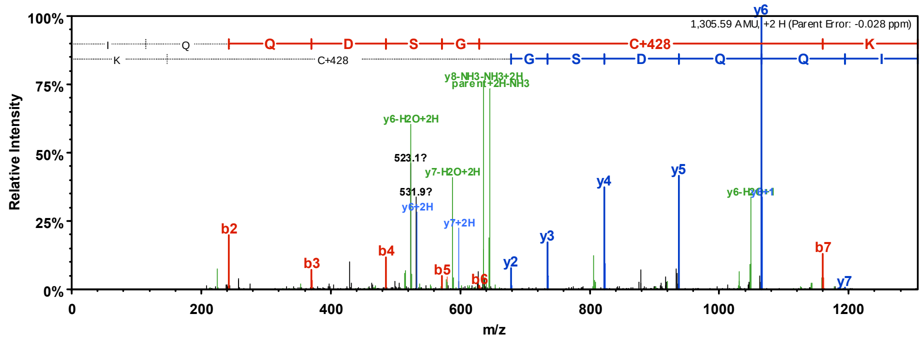

Isoform 1 of Heterogeneous nuclear ribonucleoprotein M
(K)DKFNEcGHVLYADIK(M)
XCorr: 2.79 ΔCn: 0.36

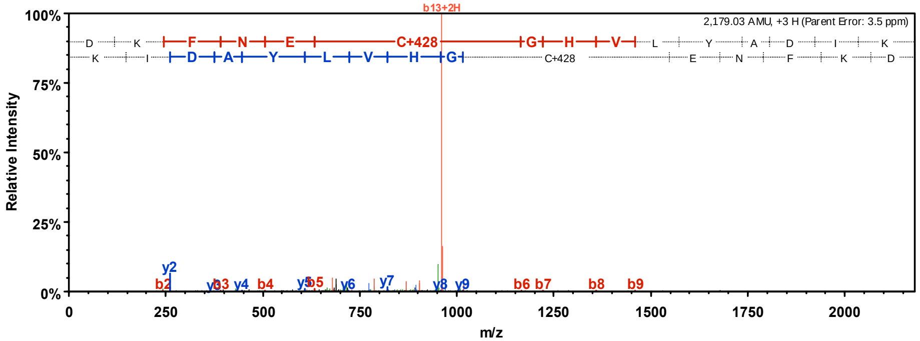

P4HB(K)KEEcPAVR(L)
XCorr: 2.5896 ΔCn: 0.3136

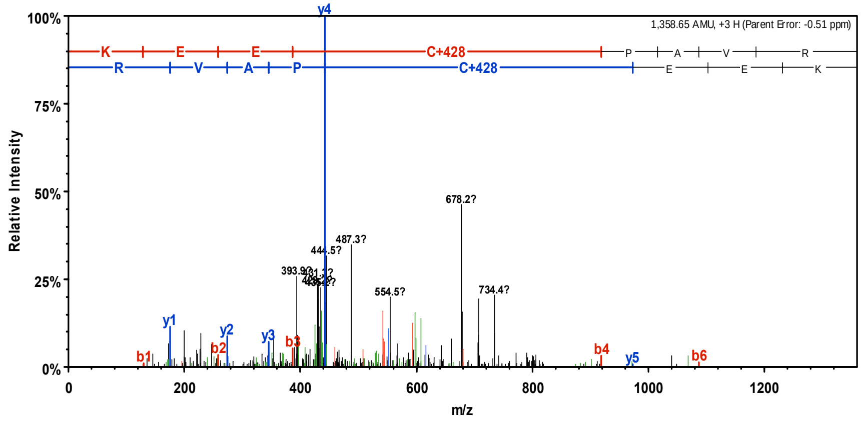

Protein disulfide-isomerase A3 (K)KFIQENIFGIcPHmTEDNKDLIQGK(D)
XCorr: 4.81 ΔCn: 0.53

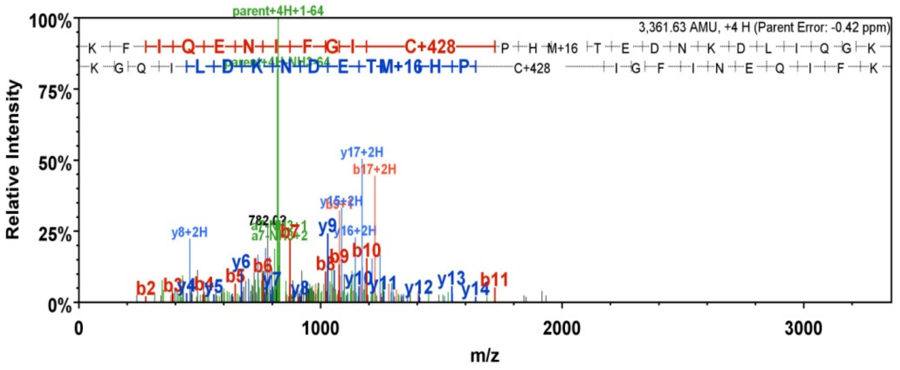

HSPA8 Heat shock 70 kDa protein 8 (K)VcNPIITK(L)
XCorr: 2.56 ΔCn: 0.35

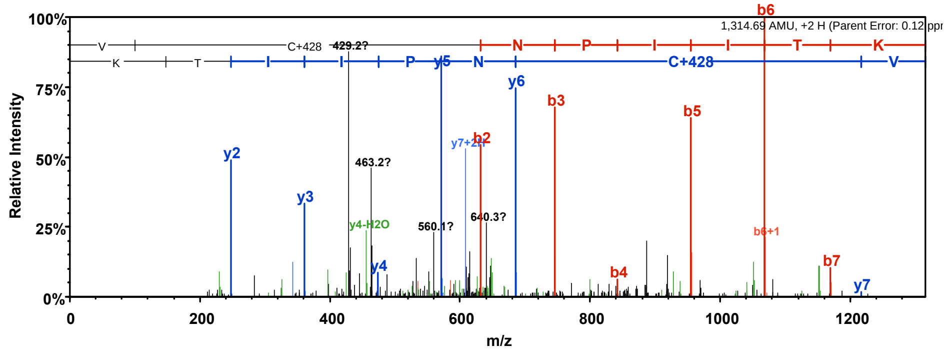

60 kDa heat shock protein, mitochondrial (K)cEFQDAYVLLSEK(K)
XCorr: 4.14 ΔCn: 0.52

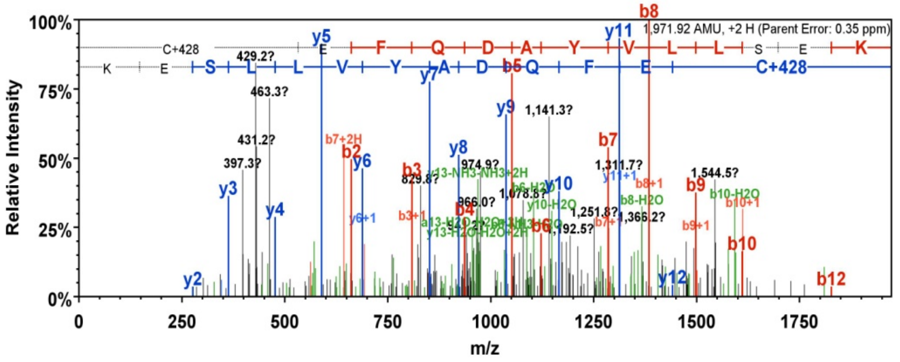

Isoform 1 of Heat shock cognate 71 kDa protein (K)cNEIINWLDK(N)
XCorr: 3.17 ΔCn: 0.31

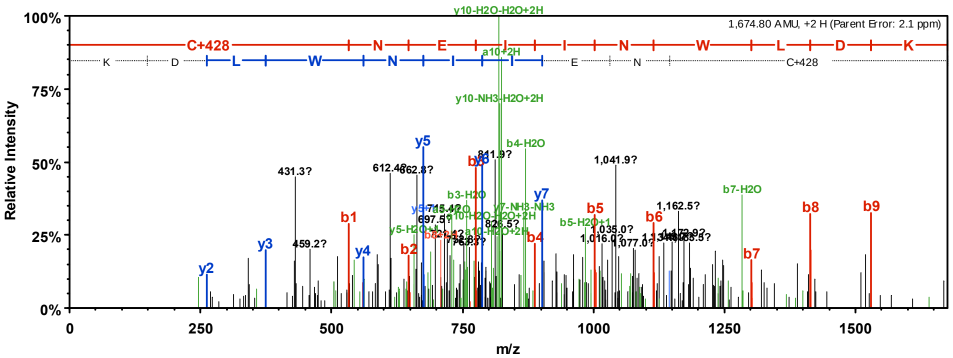

60 kDa heat shock protein, mitochondrial (K)cEFQDAYVLLSEK(K)
XCorr: 2.97 ΔCn: 0.51

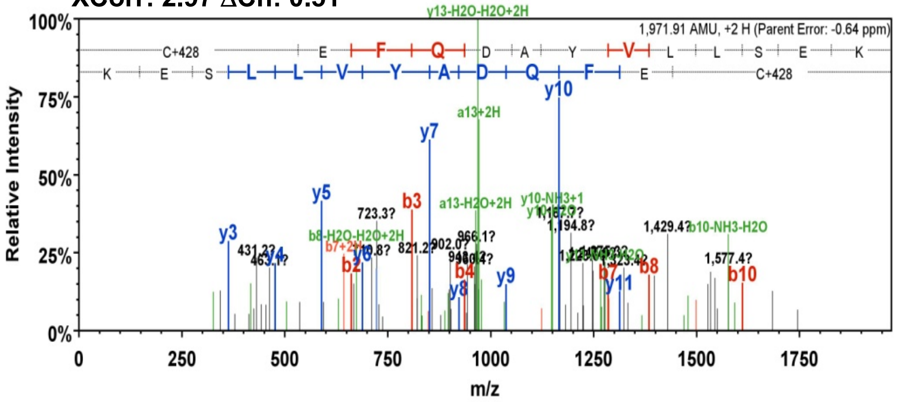

Isoform 1 of Heat shock cognate 71 kDa protein (K)cNEIINWLDKNQTAEKEEFEHQQK(E)
XCorr: 3.21 ΔCn: 0.20

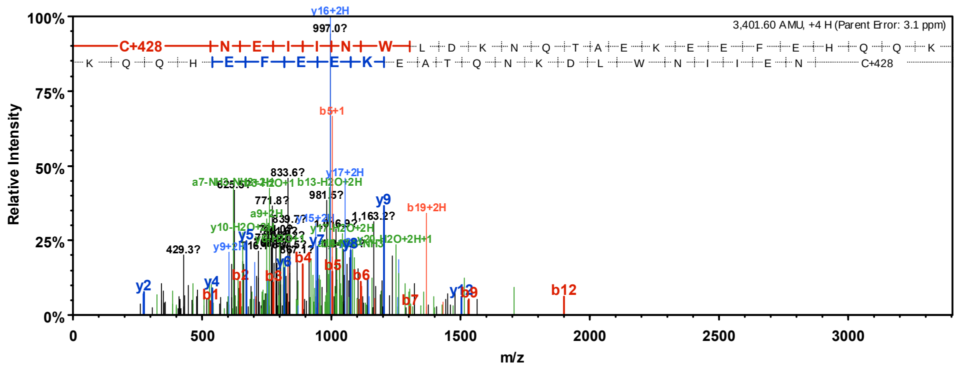

HSPA9 Heat shock 70 kDa protein 9 Mortalin (K)MEEFKDQLPADEcNK(L)
XCorr: 3.04 ΔCn: 0.33

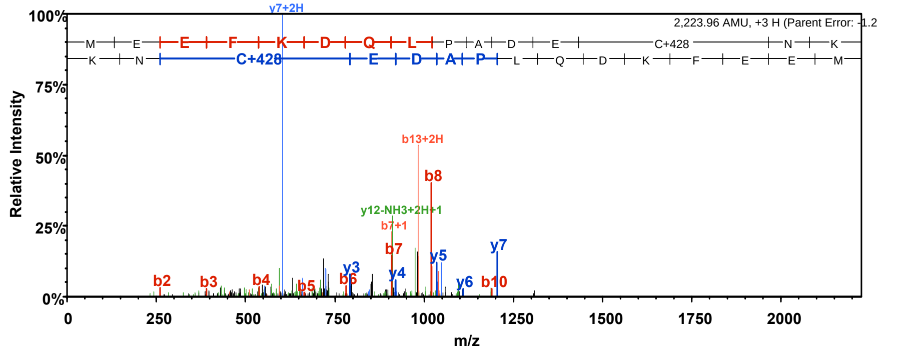

Annexin A1 (K)mYGISLcQAILDETKGDYEK(I)
XCorr: 3.2 ΔCn: 0.58

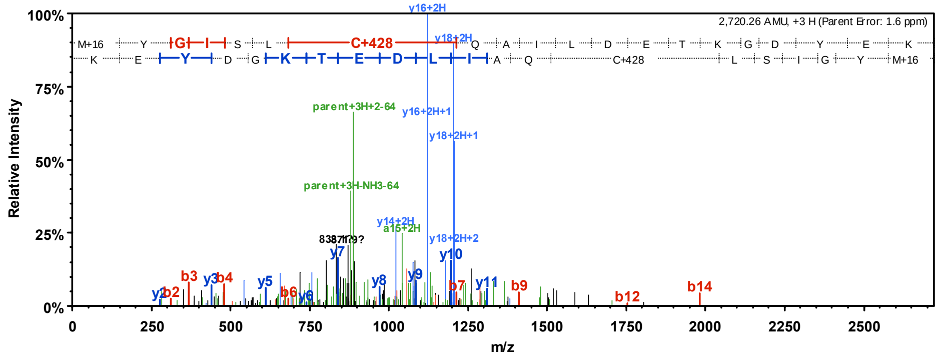

ANXA1 Annexin A1 (K)mYGISLcQAILDETKGDYEK(I)
XCorr: 3.24 ΔCn: 0.58

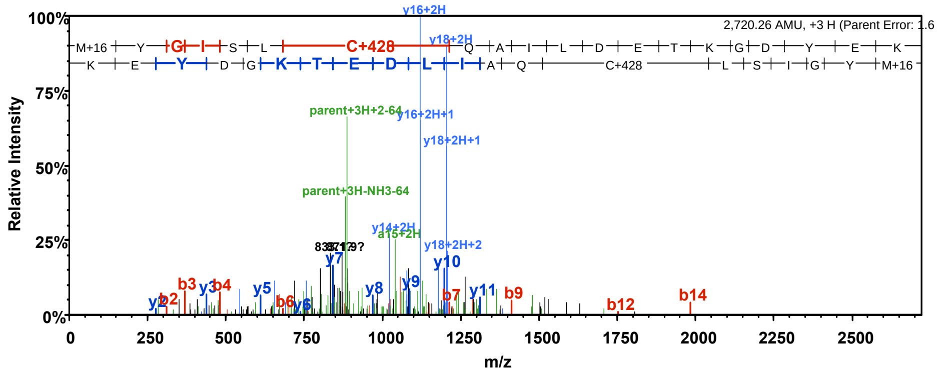

FLNA (K)ATcAPQHGAPGPGPADASK(V)
XCorr: 3.6437 ΔCn: 0.5176

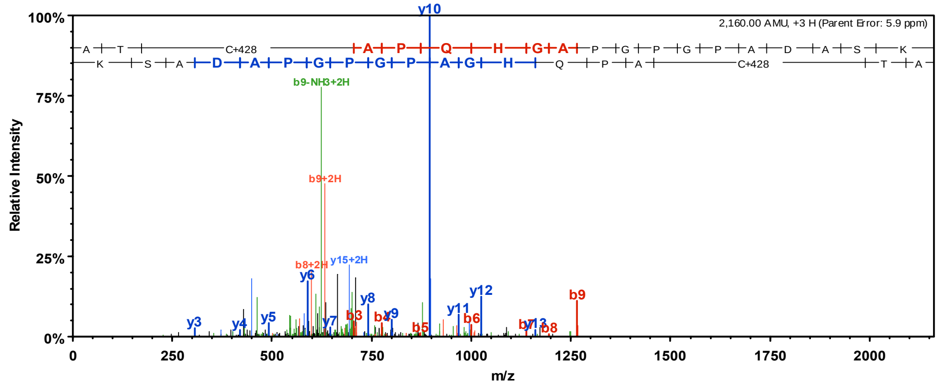

Annexin A1 (K)ILVALcGGN(-)
XCorr: 2.19 ΔCn: 0.48

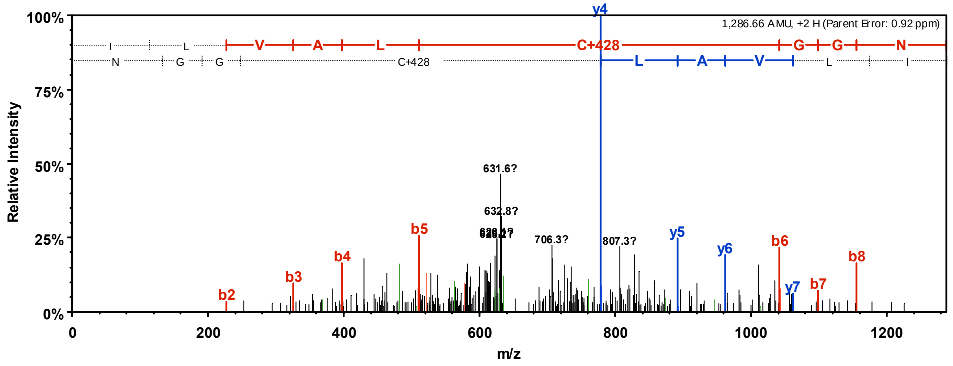

FLNA (K)cAPGVVGPAEADIDFDIIR(N)
XCorr: 3.603 ΔCn: 0.3647

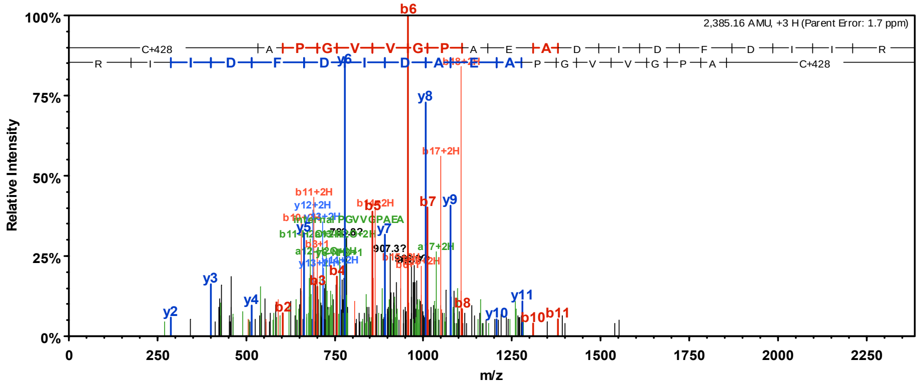

FLNA(K)LQVEPAVDTSGVQcYGPGIEGQGVFR(E)
XCorr: 3.9361 ΔCn: 0.5732

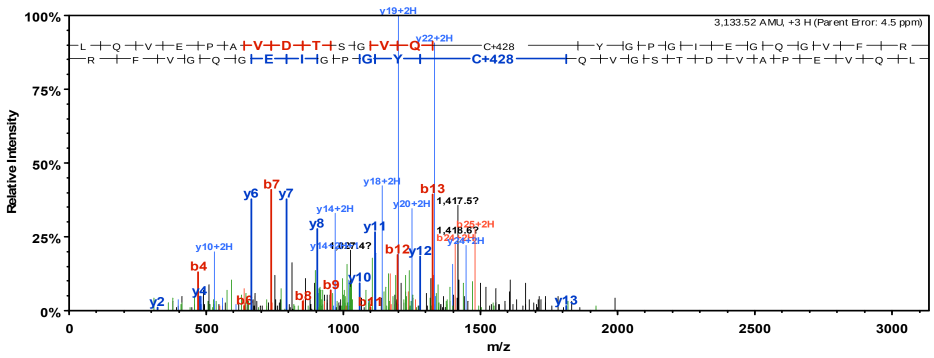

FLNB(K)VAVTEGcQPSR(V)
XCorr: 2.456 ΔCn: 0.4749

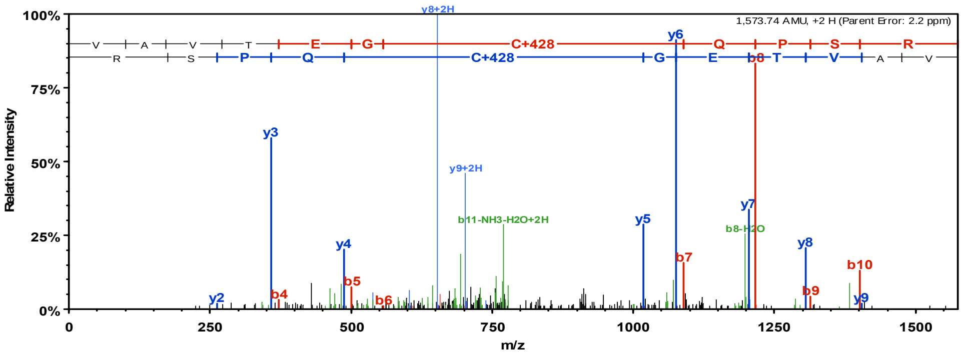

Supporting Information: (MS/MS spectra for biotinylated peptides in Table S1B)

Isoform 2 of Voltage-dependent anion-selective channel protein 2
(K)ScSGVEFSTSGSSNTDTGK(V)
XCorr: 4.56 ΔCn: 0.67

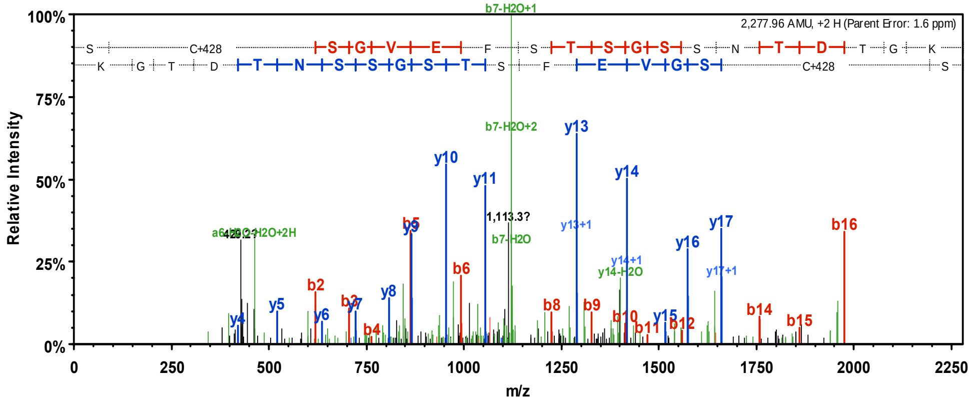

Isoform SERCA2A of Sarcoplasmic/endoplasmic reticulum calcium ATPase 2
(R)cLALATHDNPLR(R)
XCorr: 4.23 ΔCn: 0.51

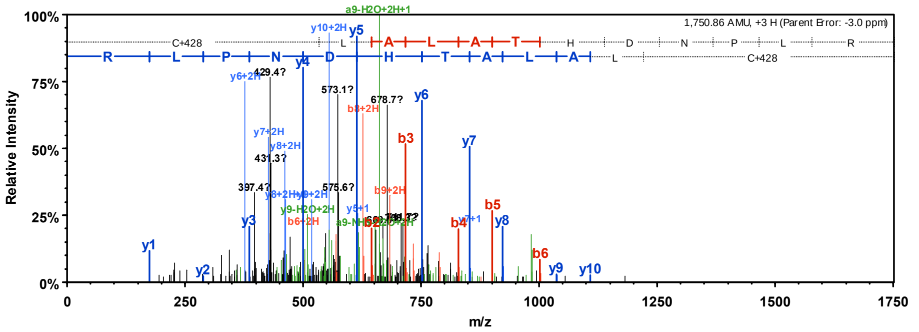

Isoform 1 of Voltage-dependent anion-selective channel protein 3
(K)VcNYGLTFTQK(W)
XCorr: 2.44 ΔCn: 0.21

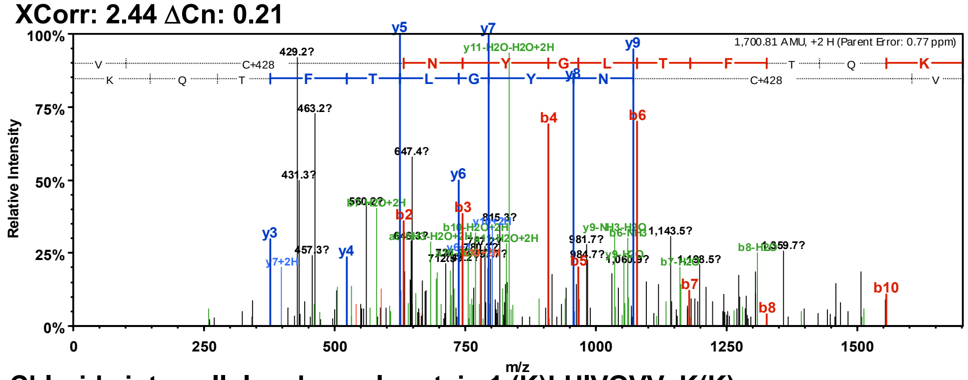

Isoform Mitochondrial of Peroxiredoxin-5, mitochondrial
(K)GVLFGVPGAFTPGcSK(T)
XCorr: 2.78 ΔCn: 0.30

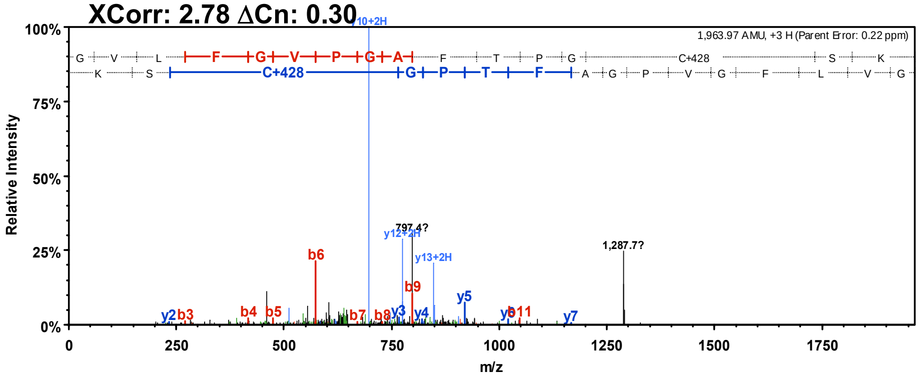

Chloride intracellular channel protein 1 (K)LHIVQVVcK(K)
XCorr: 2.51 ΔCn: 0.46

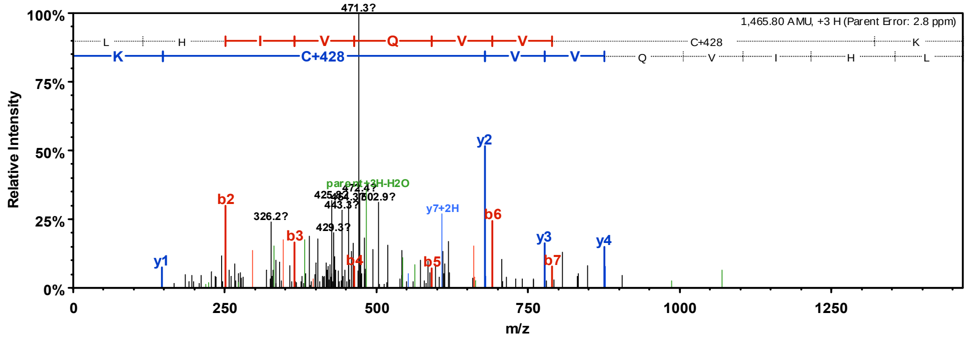

PRDX1(K)HGEVcPAGWKPGSDTIKPDVQK(S)
XCorr: 3.4631 ΔCn: 0.4084

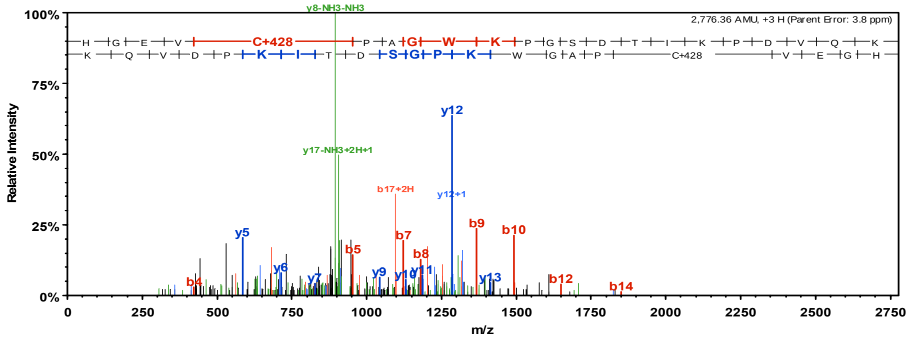

Peroxiredoxin-1 (K)HGEVcPAGWK(P)
XCorr: 3.24 ΔCn: 0.49

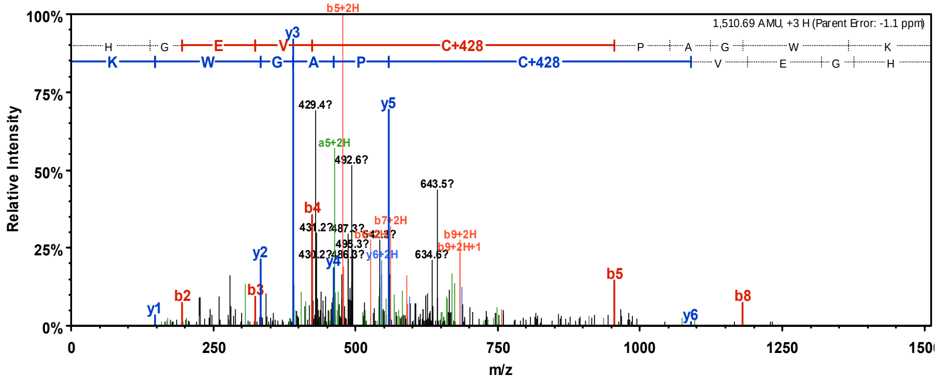

Neuroblast differentiation-associated protein AHNAK
(K)GPFVEAEVPDVDLEcPDAK(L)
XCorr: 2.90 ΔCn: 0.33

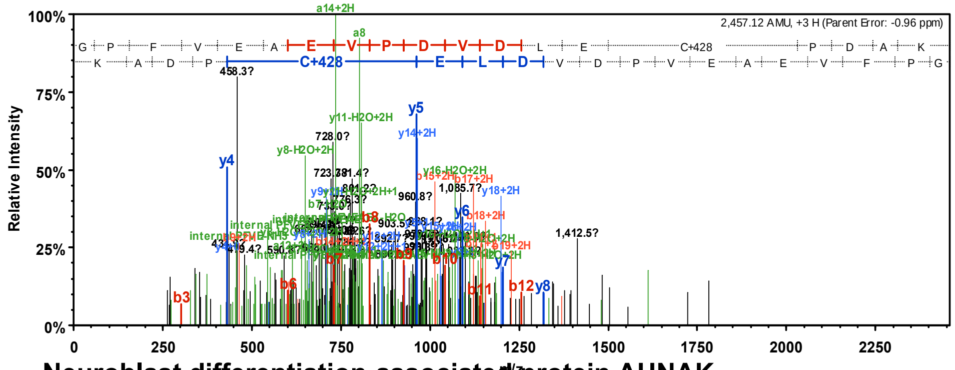

RNH1 ribonuclease/angiogenin inhibitor (K)LGDVGMAELcPGLLHPSSR(L)
XCorr: 3.25 ΔCn: 0.56

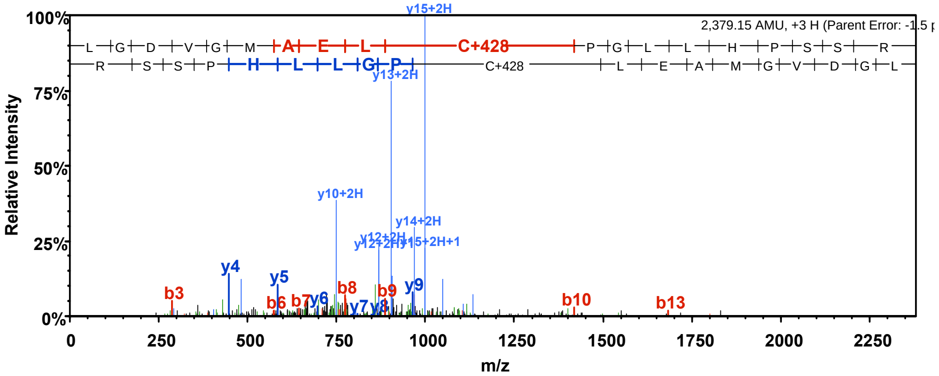

Neuroblast differentiation-associated protein AHNAK
(K)VDVEcPDVNIEGPEGK(W)
XCorr: 3.76 ΔCn: 0.56

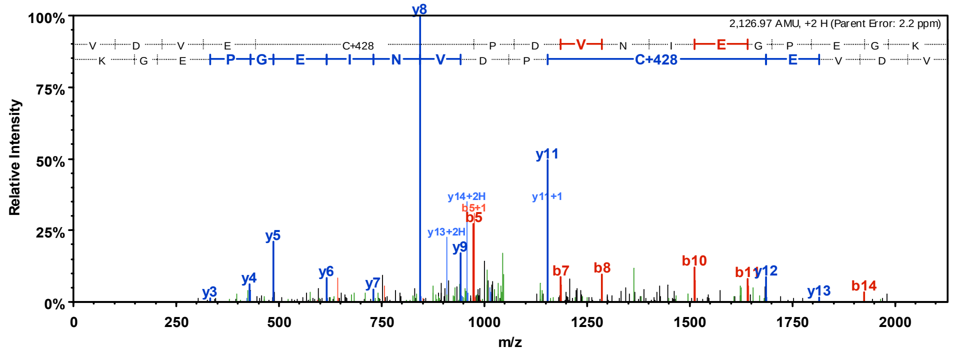

RNH1 ribonuclease/angiogenin inhibitor (R)LDDcGLTEAR(C)
XCorr: 2.88 ΔCn: 0.41

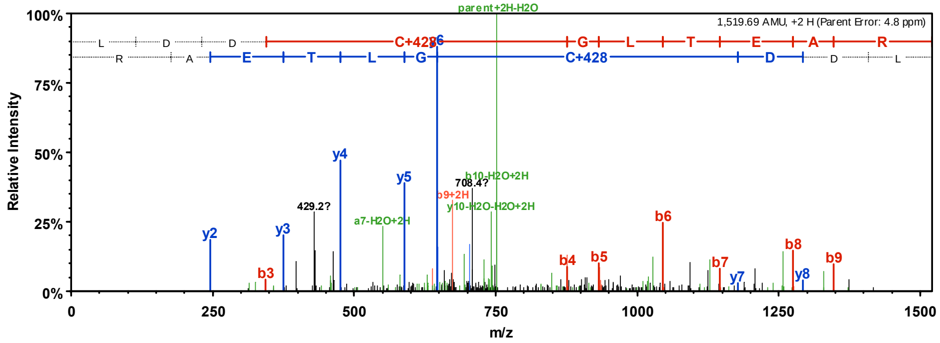

Neuroblast differentiation-associated protein AHNAK
(K)LEGDLTGPSVGVEVPDVELEcPDAK(L)
XCorr: 4.49 ΔCn: 0.48

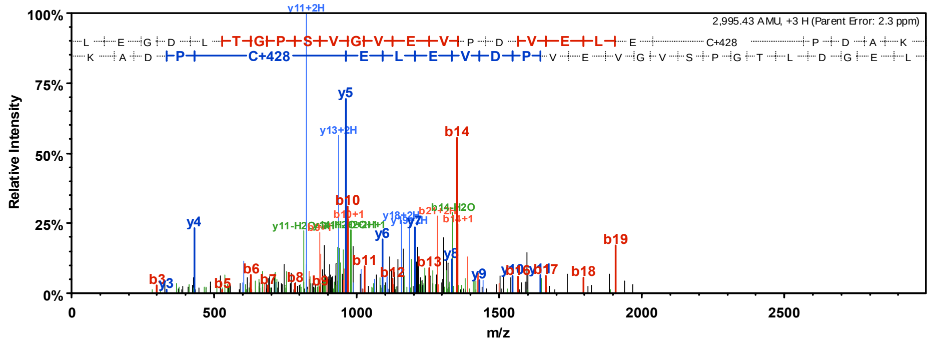

RPLP0 ribosomal protein, large, P0 (K)cFIVGADNVGSK(Q)
XCorr: 2.97 ΔCn: 0.53

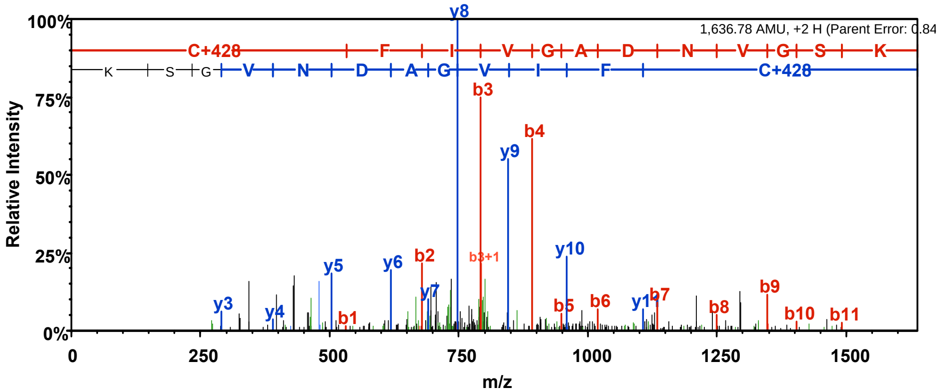

Isoform 1 of Cytoskeleton-associated protein 4 (K)SSSSSSASAAAAAAAAASSSAScSR(R)
XCorr: 3.88 ΔCn: 0.60

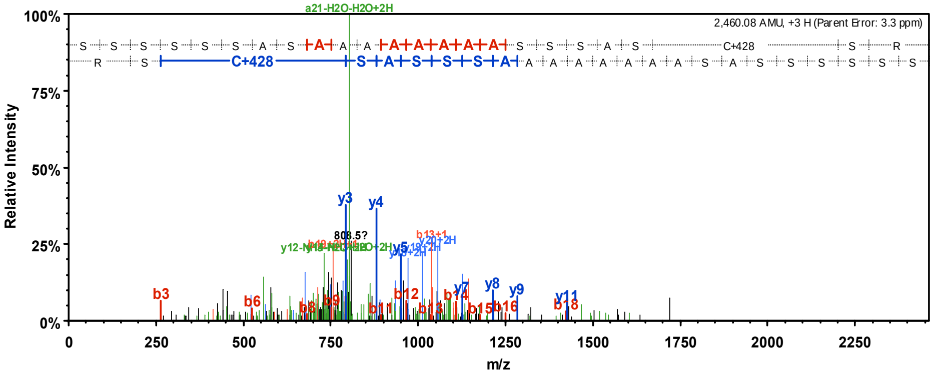

GAPDH glyceraldehyde-3-P dehydrogenase (R)VPTANVSVVDLTcR(L)
XCorr: 4.11 ΔCn: 0.65

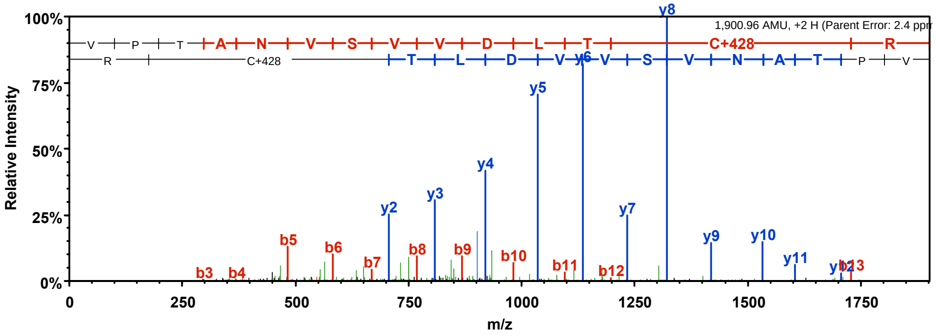

Isoform Alpha-6X1X2B of Integrin alpha-6 (K)ScFEYTANPAGYNPSISIVGTLEAEK(E)
XCorr: 3.53 ΔCn: 0.34

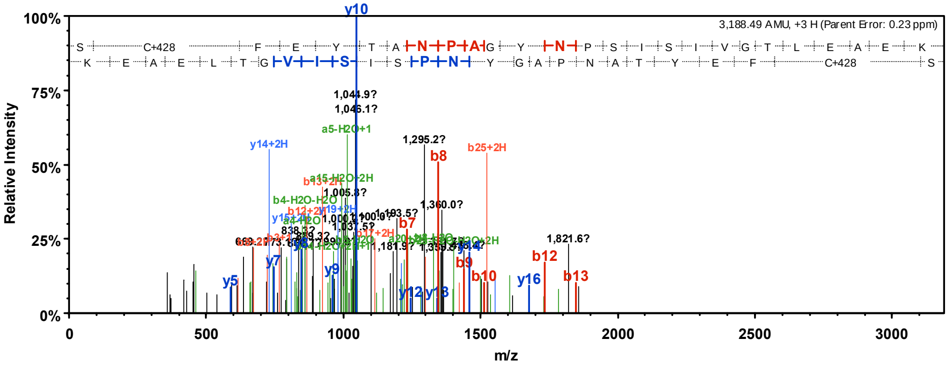

GAPDH glyceraldehyde-3-P dehydrogenase (K)IISNAScTTNcLAPLAK(V)
XCorr: 2.67 ΔCn: 0.3

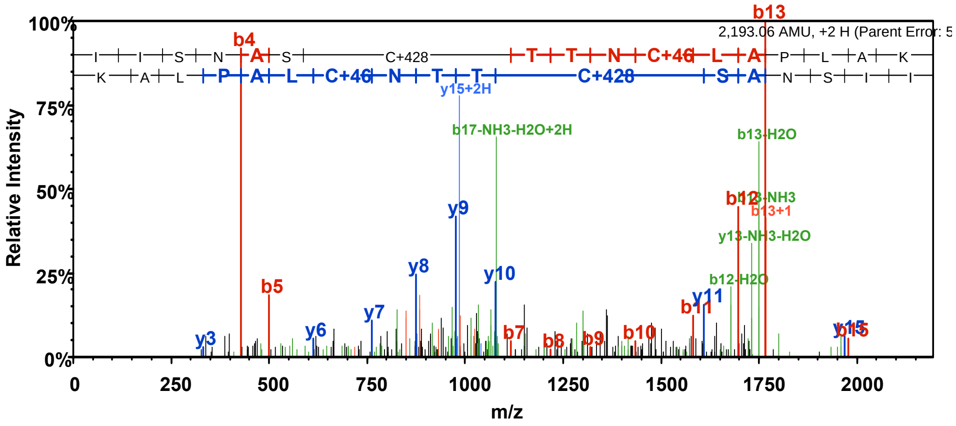

Heme oxygenase 2 (K)GALEGSScPFR(T)
XCorr: 2.33 ΔCn: 0.32

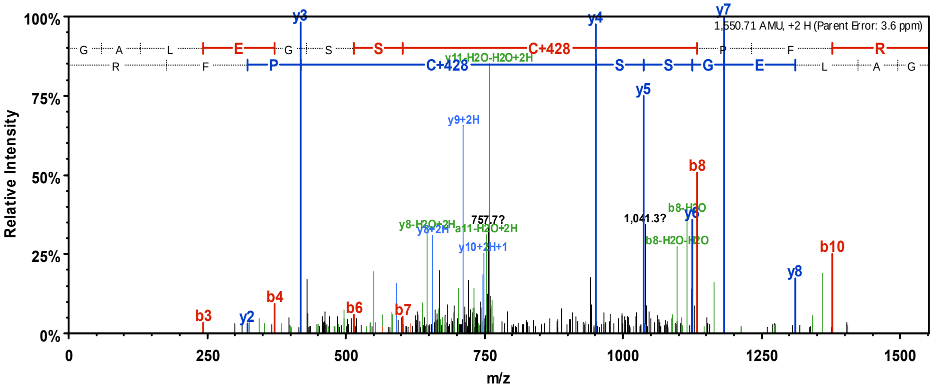

Galectin-1 (R)FNAHGDANTIVcNSK(D)
XCorr: 3.67 ΔCn: 0.66

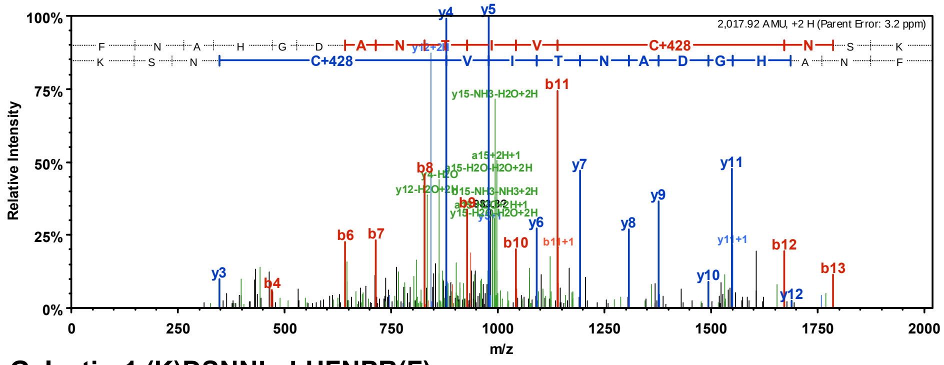

Alpha-actinin-4 (K)AcLISLGYDVENDR(Q)
XCorr: 3.22 ΔCn: 0.66

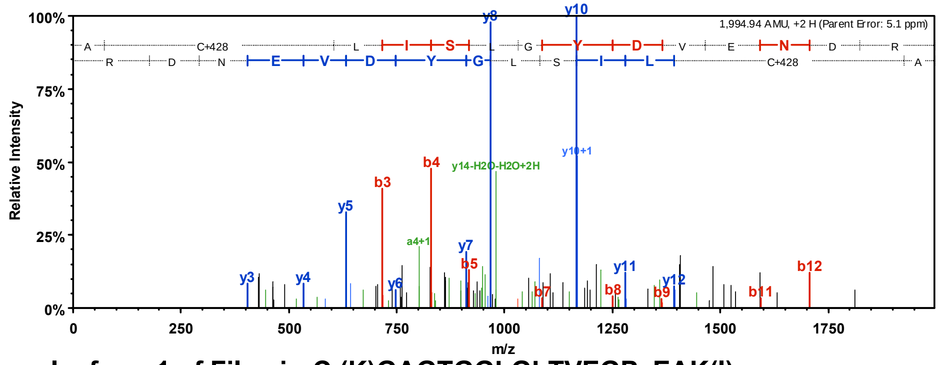

Galectin-1 (K)DSNNLcLHFNPR(F)
XCorr: 3.00 ΔCn: 0.40

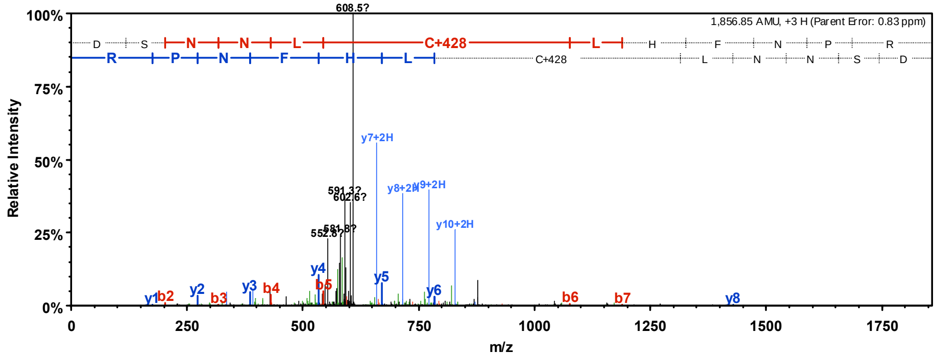

Isoform 1 of Filamin-C (K)GAGTGGLGLTVEGPcEAK(I)
XCorr: 2.54 ΔCn: 0.41

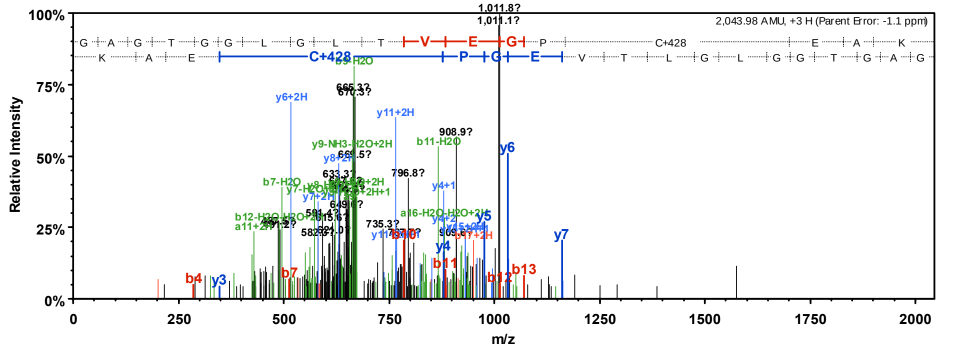

Alpha-actinin-4 (K)IcDQWDALGSLTHSR(R)
XCorr: 5.37 ΔCn: 0.64

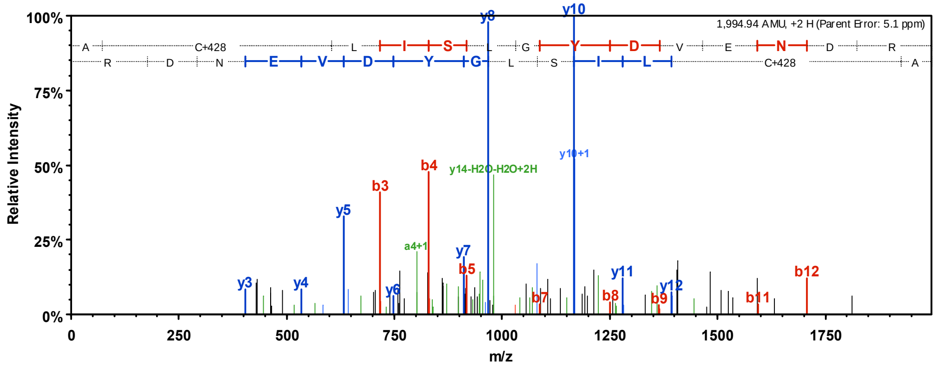

XRCC5(K)ETVYcLNDDDETEVLKEDIIQGFR(Y)
XCorr: 4.81 ΔCn: 0.65

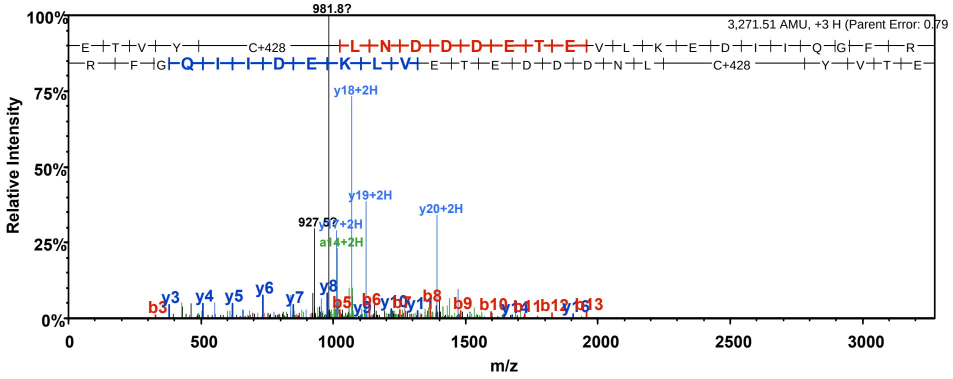

ATP-dependent DNA helicase 2 subunit 2 (R)LFQcLLHR(A)
XCorr: 2.3 ΔCn: 0.35

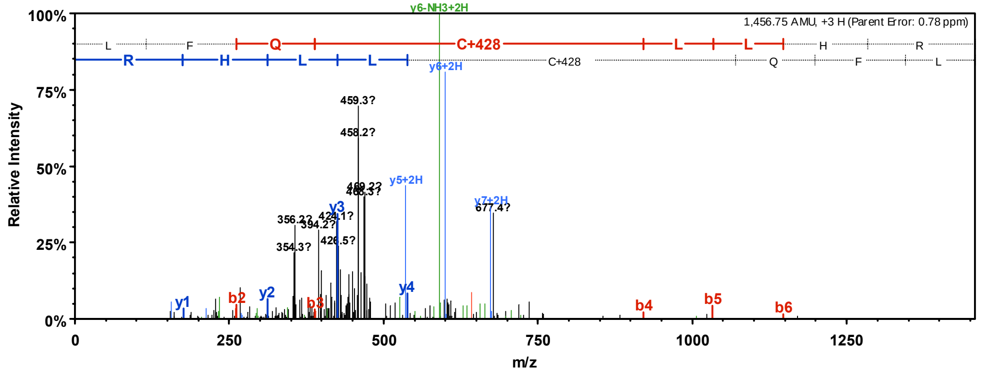

PCNA (K)cAGNEDIITLR(A)
XCorr: 3.68 ΔCn: 0.57

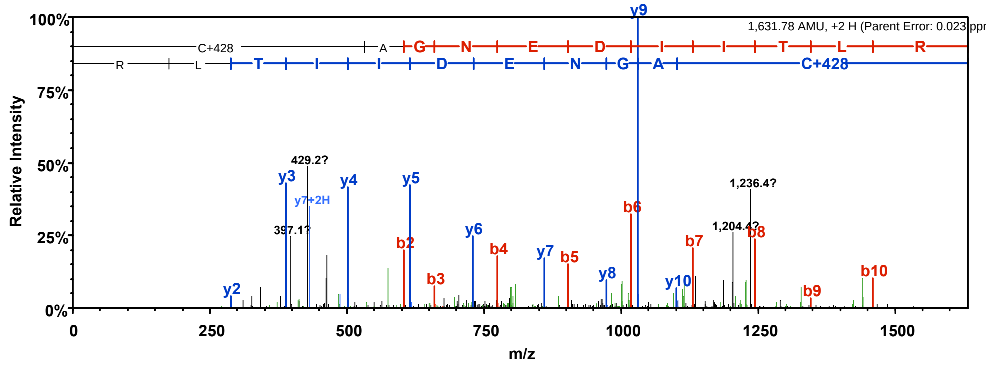

PCNA (R)DLSHIGDAVVIScAK(D)
XCorr: 3.63 ΔCn: 0.57

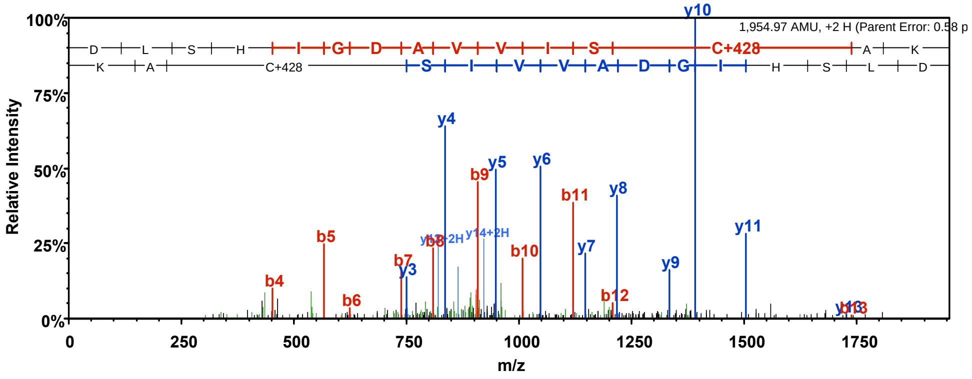

ALDOA aldolase A) (R)ALANSLAcQGK(Y)
XCorr: 3.08 ΔCn: 0.57

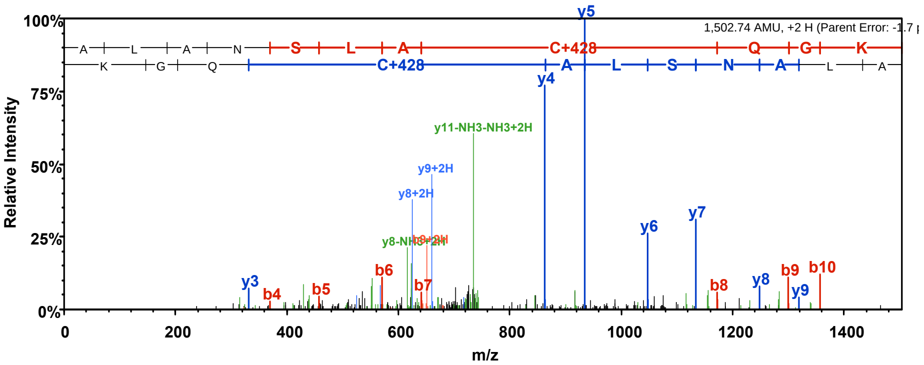

ALDOA aldolase A) (R)VNPcIGGVILFHETLYQK(A)
XCorr: 3.59 ΔCn: 0.46

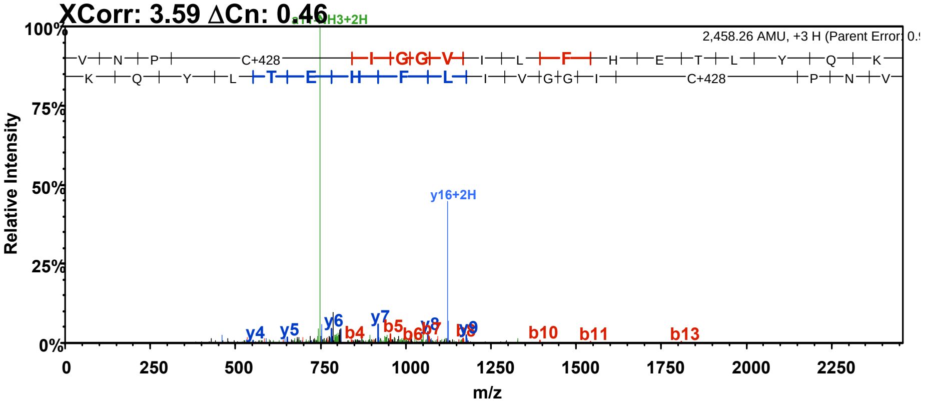

Fructose-bisphosphate aldolase A (R)ALANSLAcQGK(Y)
XCorr: 2.67 ΔCn: 0.43

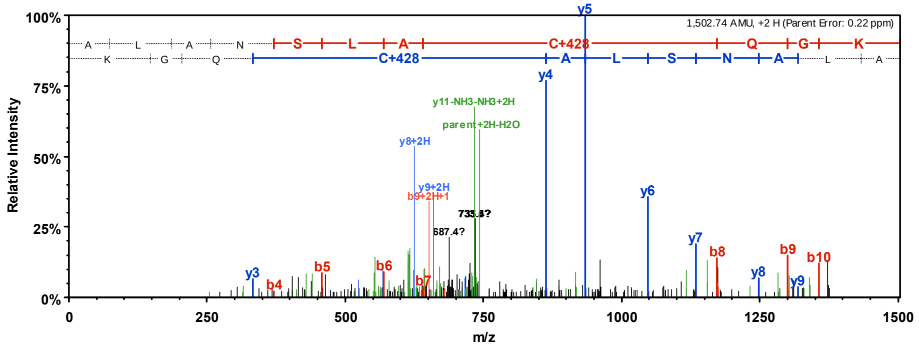

Isoform M1 of Pyruvate kinase isozymes M1/M2 (R)NTGIIcTIGPASR(S)
XCorr: 3.09 ΔCn: 0.35

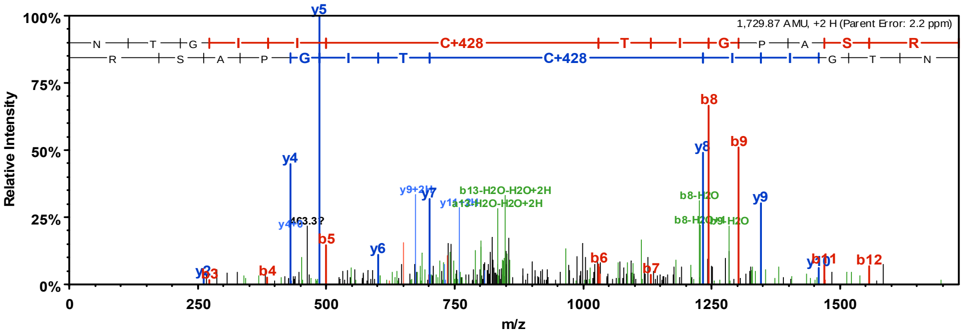

Isoform M1 of Pyruvate kinase isozymes M1/M2 (R)AEGSDVANAVLDGADcIMLSGETAK(G)
XCorr: 3.77 ΔCn: 0.57

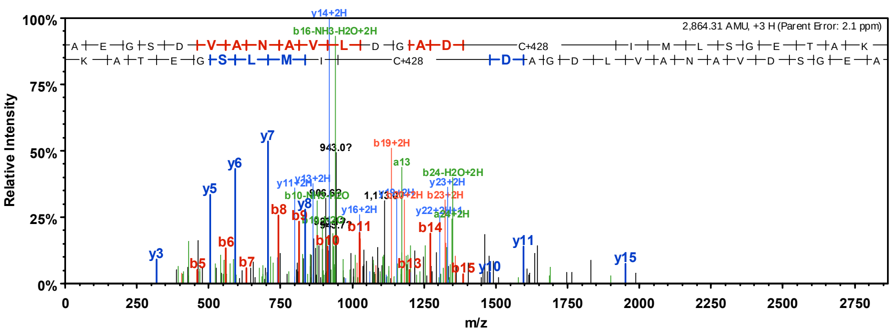

Isoform M1 of Pyruvate kinase isozymes M1/M2 (R)AGKPVIcATQmLESmIK(K)
XCorr: 4.08 ΔCn: 0.57

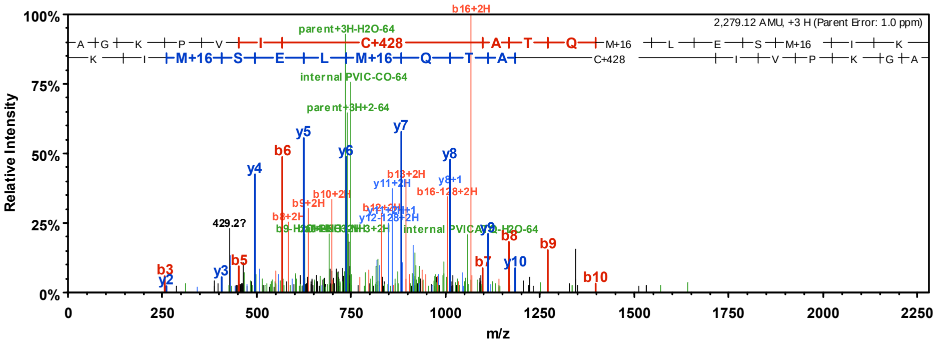

Isoform M1 of Pyruvate kinase isozymes M1/M2 (K)cDENILWLDYK(N)
XCorr: 2.22 ΔCn: 0.44

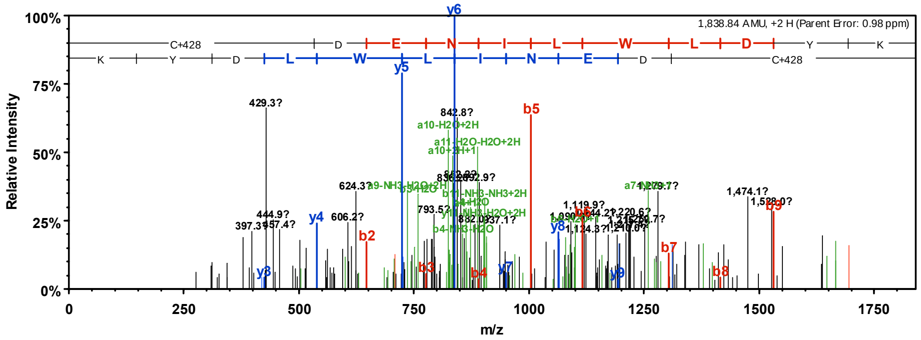

Isoform M1 of Pyruvate kinase isozymes M1/M2 (K)PVIcATQMLESMIK(K)
XCorr: 3.51 ΔCn: 0.60

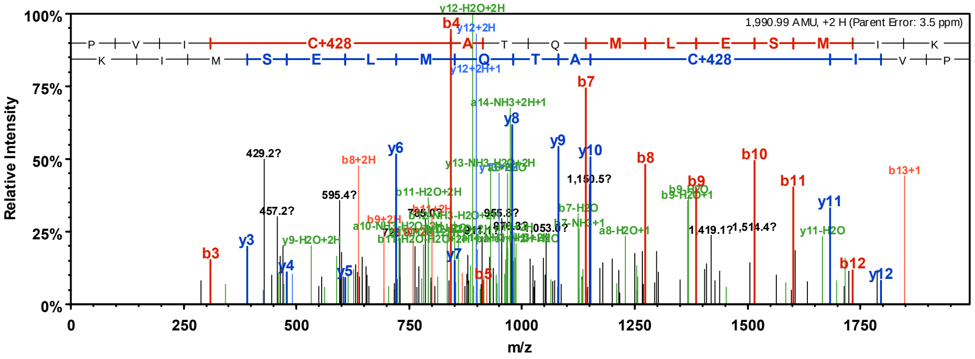

Isoform M1 of Pyruvate kinase isozymes M1/M2 (R)AGKPVIcATQMLESmIK(K)
XCorr: 4.46 ΔCn: 0.57

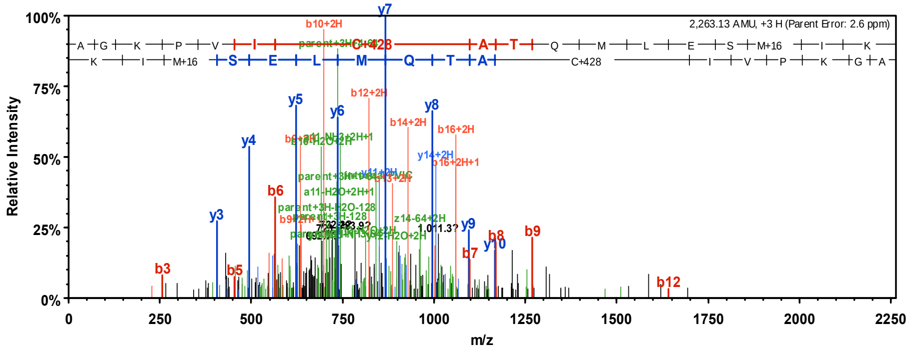

Inosine-5'-monophosphate dehydrogenase 2 (R)DIDFLKEEEHDcFLEEImTK(R)
XCorr: 3.59 ΔCn: 0.36

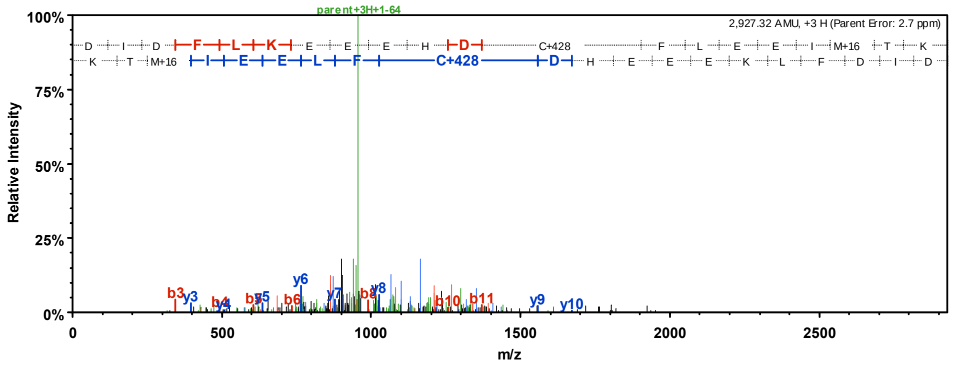

Aconitate hydratase, mitochondrial (K)cTTDHISAAGPWLK(F)
XCorr: 3.86 ΔCn: 0.46

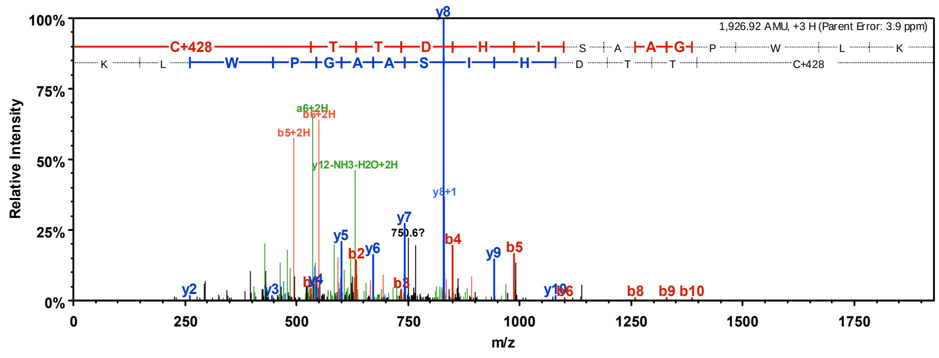

Glucose-6-phosphate isomerase (K)MIPcDFLIPVQTQHPIR(K)
XCorr: 2.84 ΔCn: 0.38

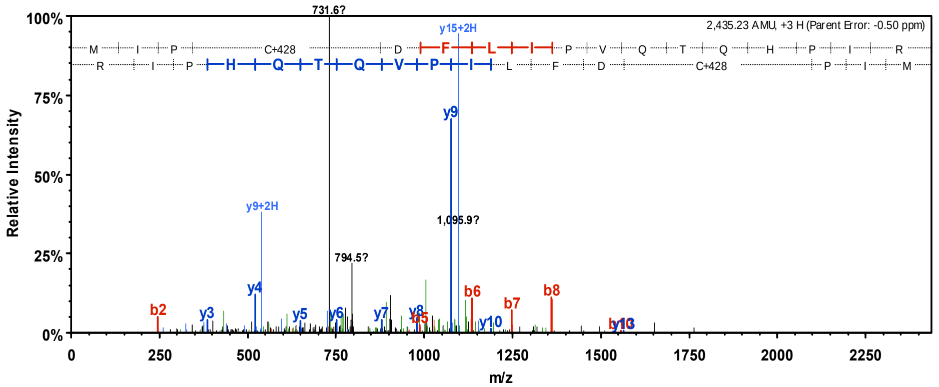

Tubulin alpha-4A chain (R)AVcMLSNTTAIAEAWAR(L)
XCorr: 4.61 ΔCn: 0.55

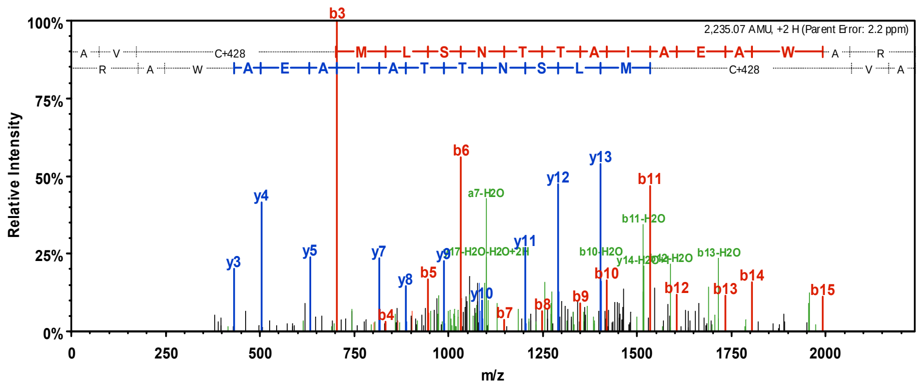

Glucose-6-phosphate isomerase (K)mIPcDFLIPVQTQHPIR(K)
XCorr: 3.14 ΔCn: 0.37

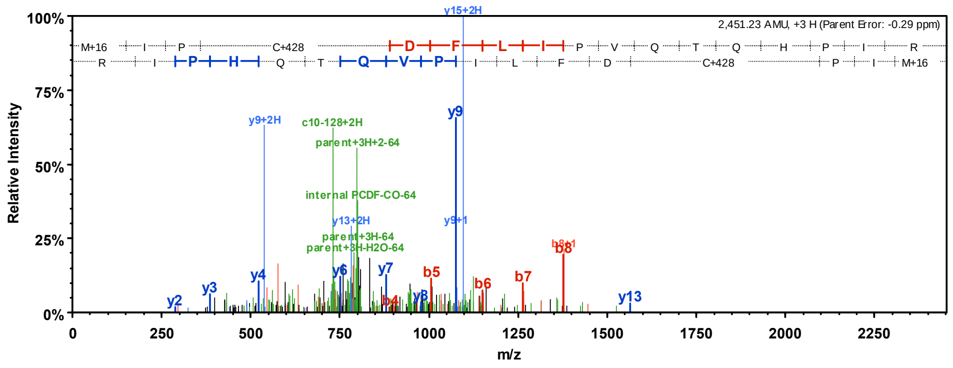

Malate dehydrogenase, mitochondrial (K)GYLGPEQLPDcLK(G)
XCorr: 2.46 ΔCn: 0.50

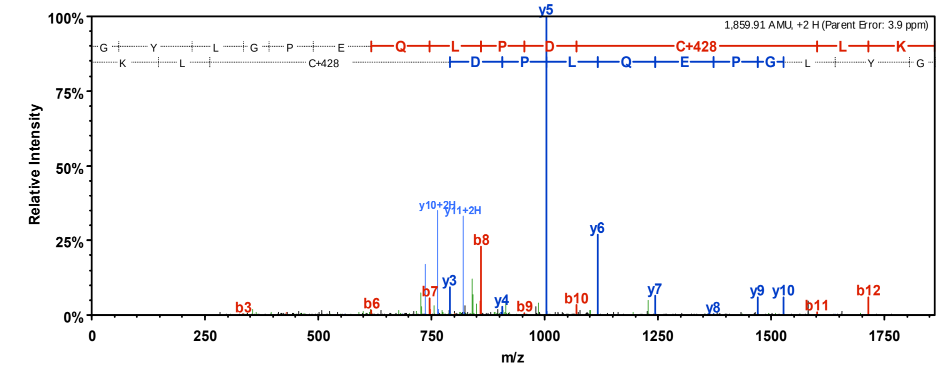

Malate dehydrogenase, mitochondrial (K)SQETEcTYFSTPLLLGK(K)
XCorr: 2.59 ΔCn: 0.49

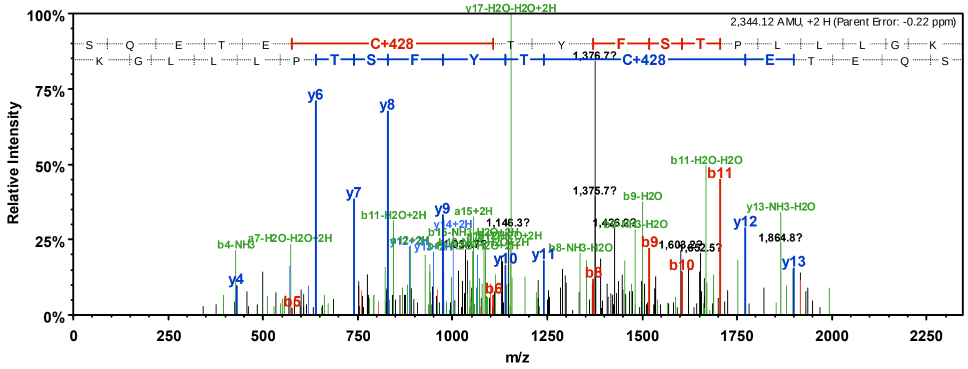

Malate dehydrogenase, mitochondrial (K)VAVLGASGGIGQPLSLLLK(N)
XCorr: 5.27 ΔCn: 0.80

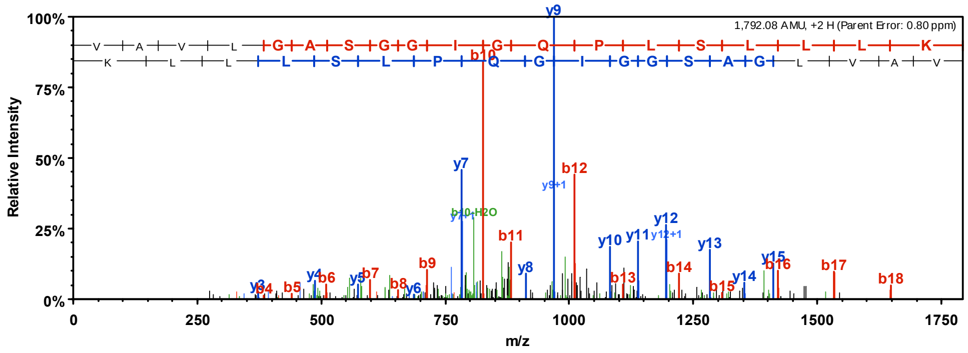

CAST Calpastatin (K)AAAPAPVSEAVcR(T)
XCorr: 2.83 ΔCn: 0.59

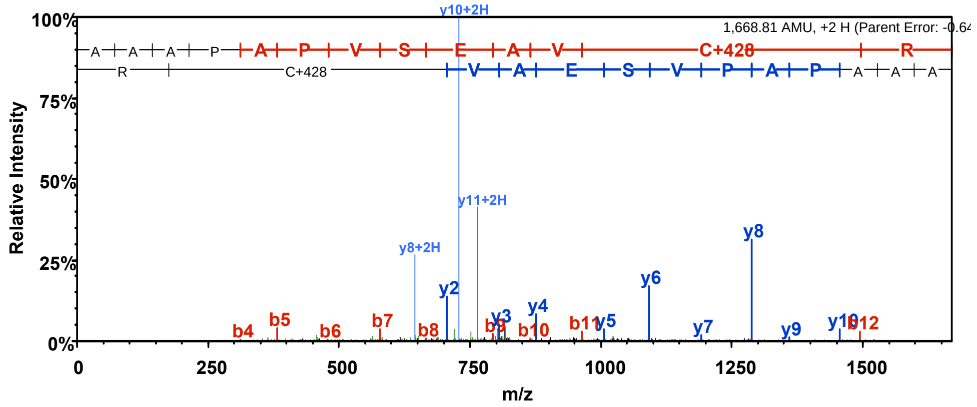

CAST(R)TSMcSIQSAPPEPATLK(G)
XCorr: 2.4946 ΔCn: 0.4552

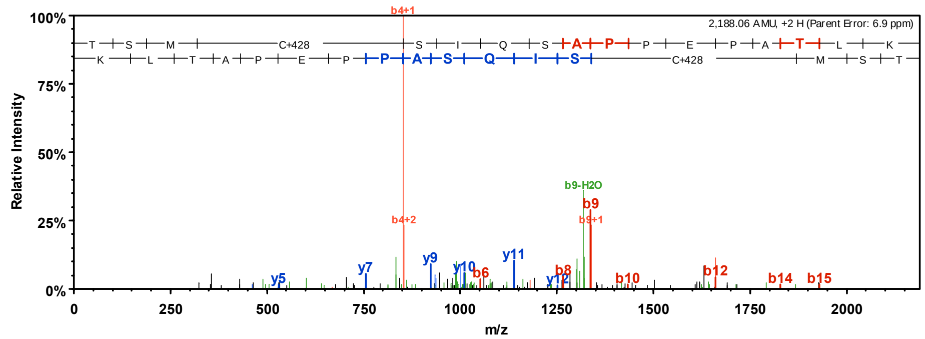

RBM39(K)cPSIAAAIAAVNALHGR(W)
XCorr: 3.845 ΔCn: 0.6294

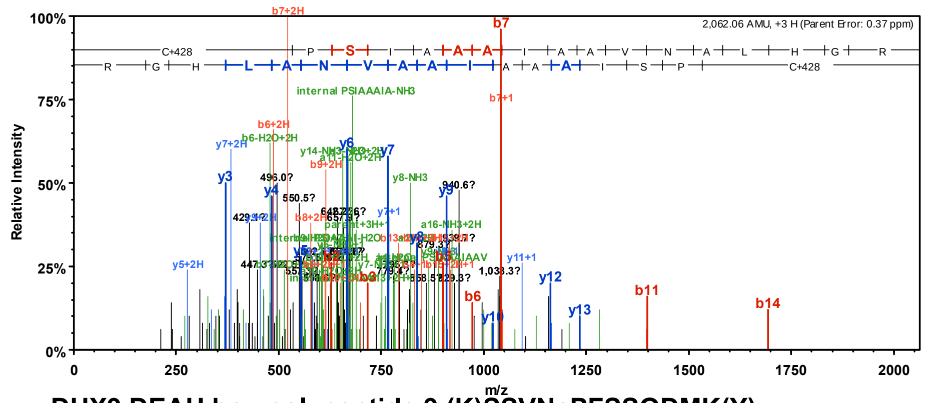

DHX9 DEAH box polypeptide 9 (K)SSVNcPFSSQDMK(Y)
XCorr: 3.02 ΔCn: 0.34

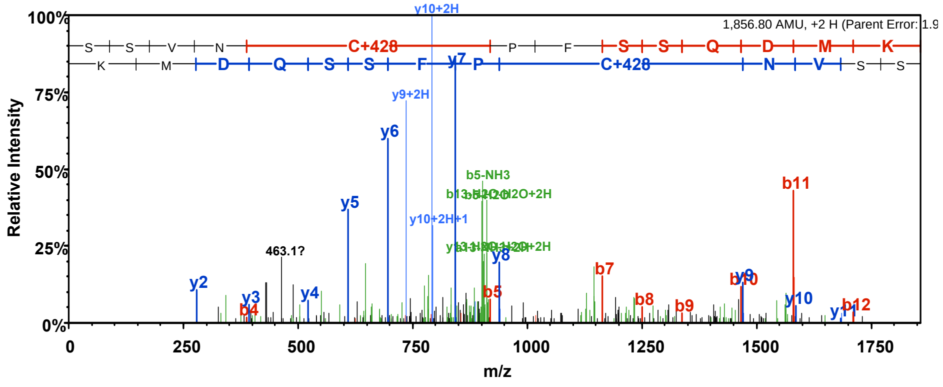

HNRNPA1 (R)SHFEQWGTLTDcVVmR(D)

XCorr: 3.49 ΔCn: 0.51

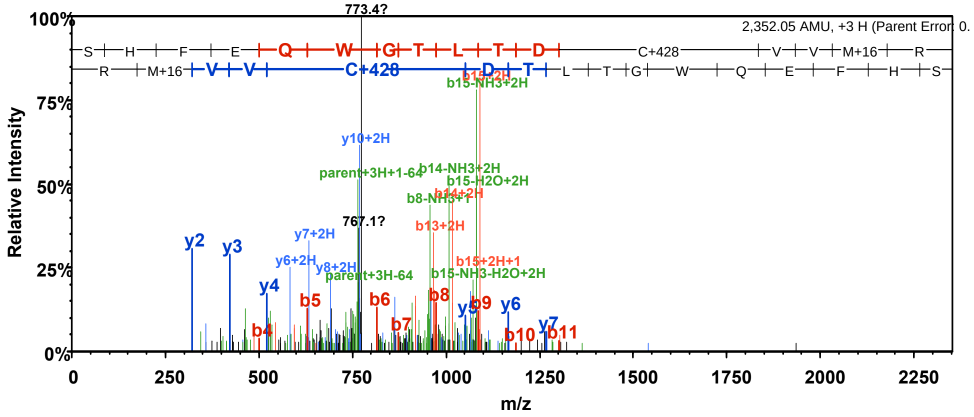

Calponin-2 (K)AGQcVIGLQMGTNK(C)

XCorr: 2.98 ΔCn: 0.40

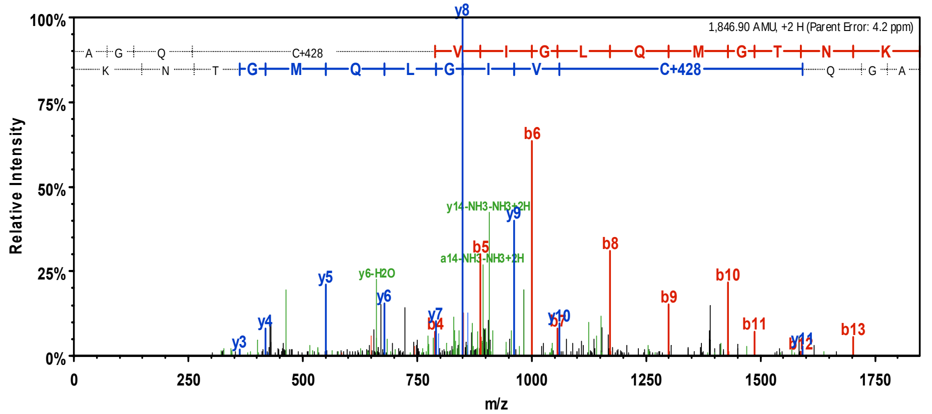

Tubulin alpha-4A chain (K)TIGGGDDSFTTFFcETGAGK(H)

XCorr: 2.76 ΔCn: 0.50

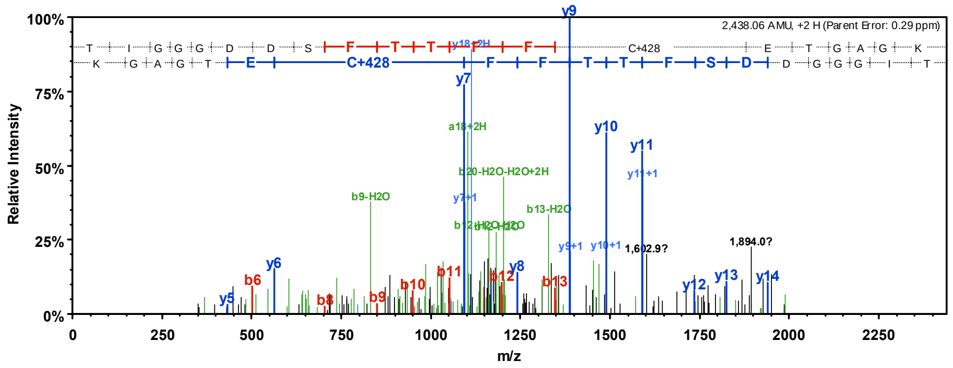

TPM4 (K)cGDLEEELK(N)

XCorr: 3.04 ΔCn: 0.33

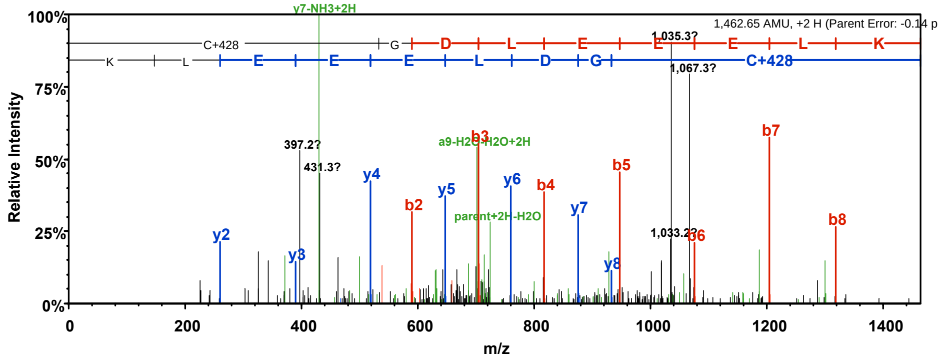

SMARCC2 SWI/SNF complex C2 (R)PNIFLcPEIEPK(L)

XCorr: 2.77 ΔCn: 0.44

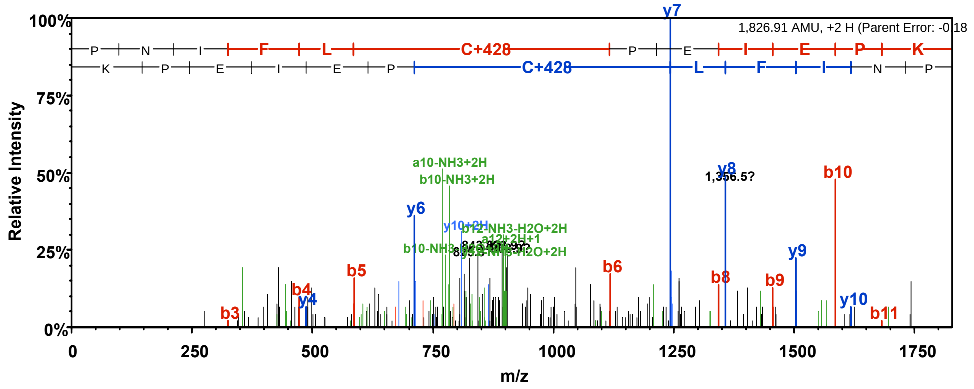

DFNA5(R)NEVLcVLTQK(I)

XCorr:3.0641 ΔCn: 0.2742

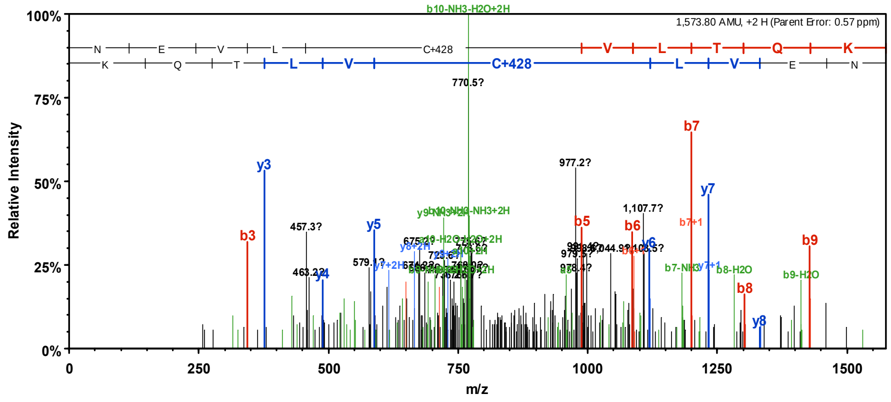

EIF2S3(K)YNIEVVcEYIVK(K)
XCorr: 2.8318 ΔCn: 0.4642

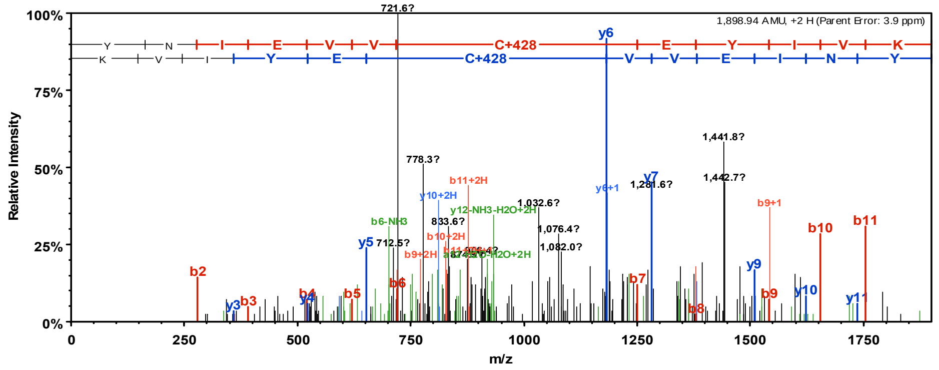

EEF2(K)IWcFGPDGTGPNILTDITK(G)
XCorr: 3.0311 ΔCn: 0.3826

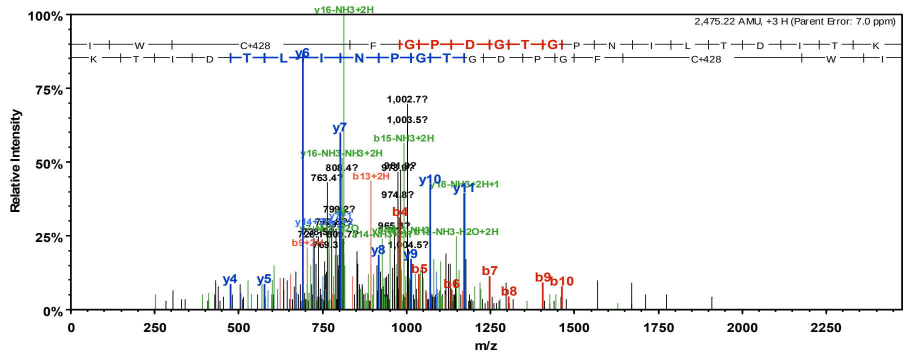

EEF2(R)cELLYEGPPDDEAAMGIK(S)
XCorr: 3.0635 ΔCn: 0.4106

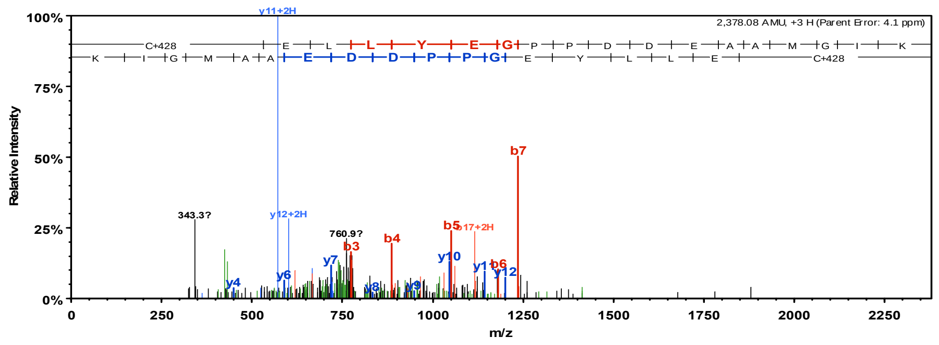

RPL5(R)IEGDmIVcAAYAHELPK(Y)
XCorr: 3.631 ΔCn: 0.5513

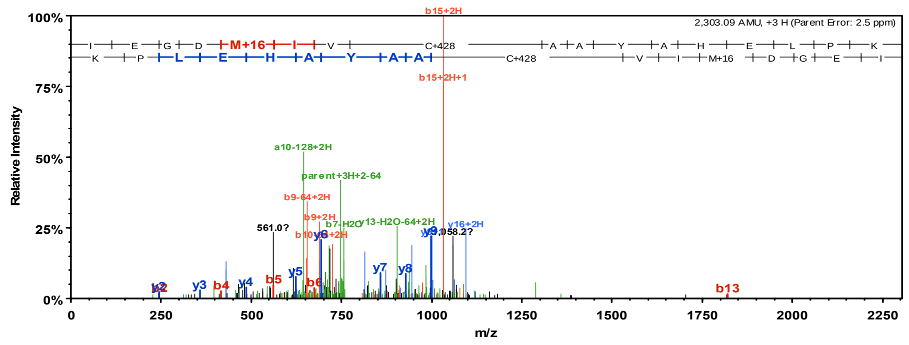

EEF2(K)STLTDSLVcK(A)
XCorr: 2.7941 ΔCn: 0.1928

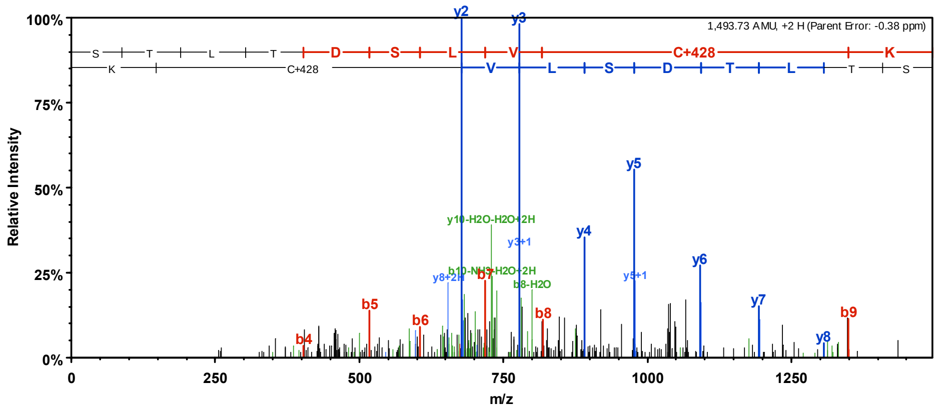

RPL5(K)VGLTNYAAAYcTGLLLAR(R)
XCorr: 2.6861 ΔCn: 0.3463

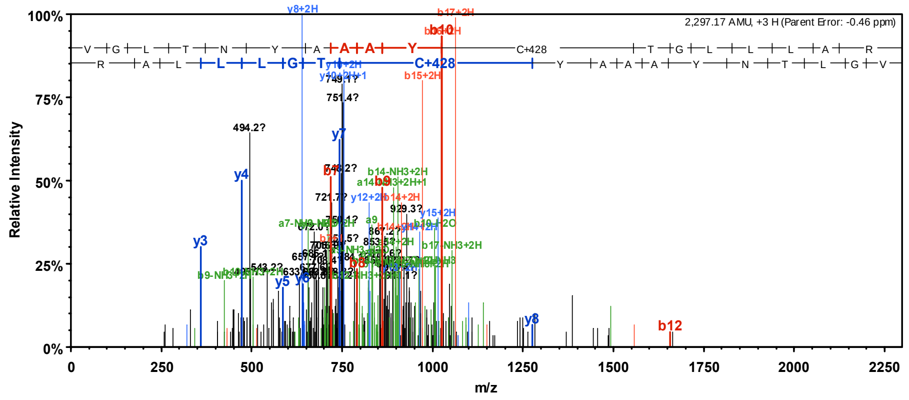

Tu translation elongation factor, mitochondrial precursor
(K)KGDEcELLGHSK(N)
XCorr: 3.05 ΔCn: 0.40

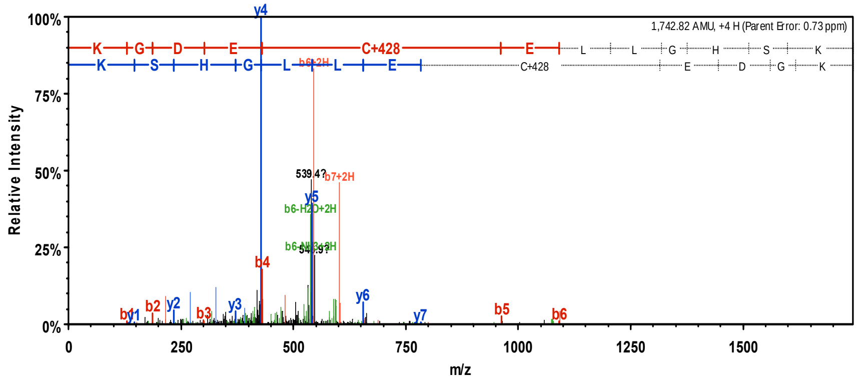

EEF1A1 eukaryotic translation elongation factor 1 alpha 1
(K)SGDAAIVDmVPGKPmcVESFSDYPPLGR(F)
XCorr: 3.61 ΔCn: 0.54

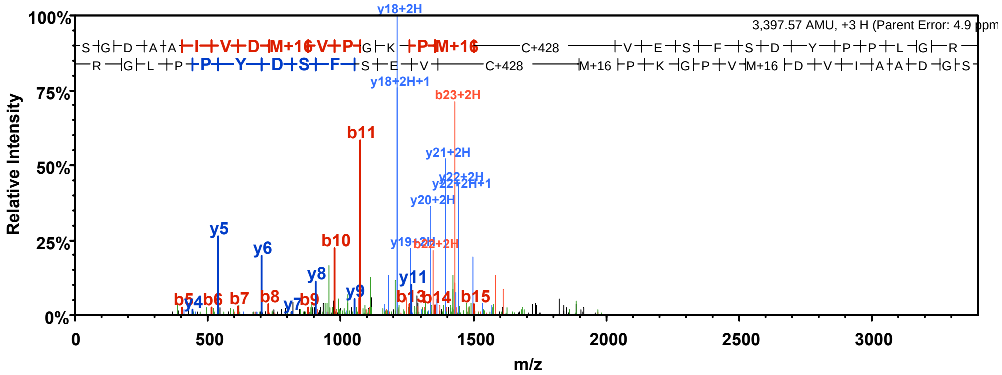

RPL5(K)VGLTNYAAAYcTGLLLAR(R)
XCorr: 2.6861 ΔCn: 0.3463

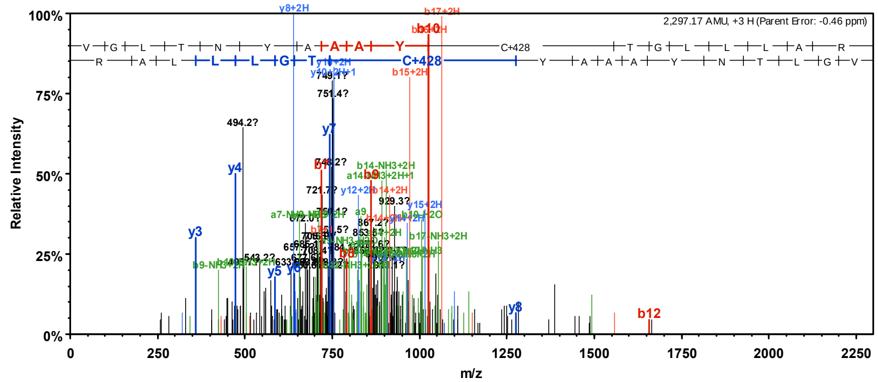

HSPA4(R)KFDEVLVNHFcEEFGK(K)
XCorr: 3.1132 ΔCn: 0.1968

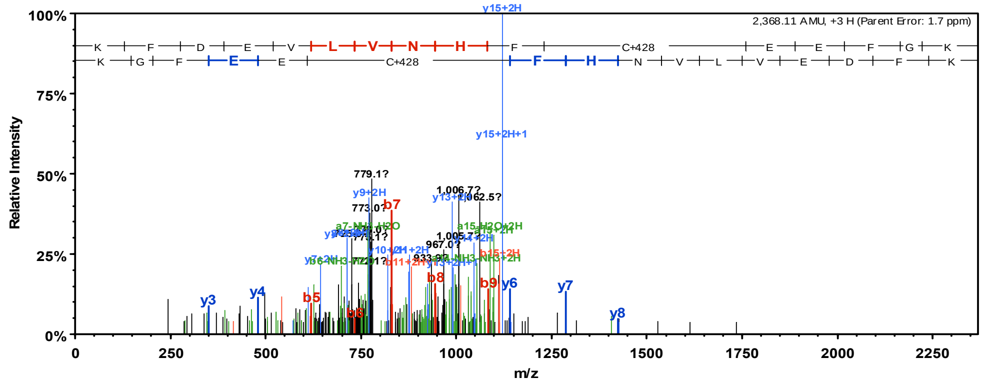

RPN1(K)VAcITEQVLTLVNK(R)
XCorr: 3.9946 ΔCn: 0.4457

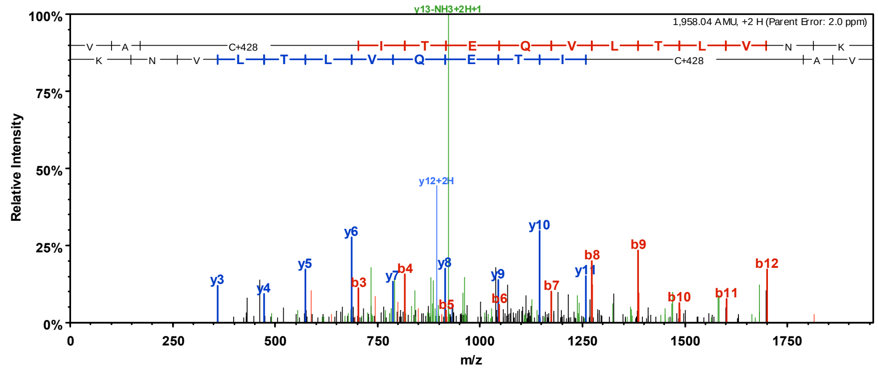

Heat shock protein HSP 90-beta (K)cLELFSELAEDKENYK(K)
XCorr: 3.55 ΔCn: 0.44

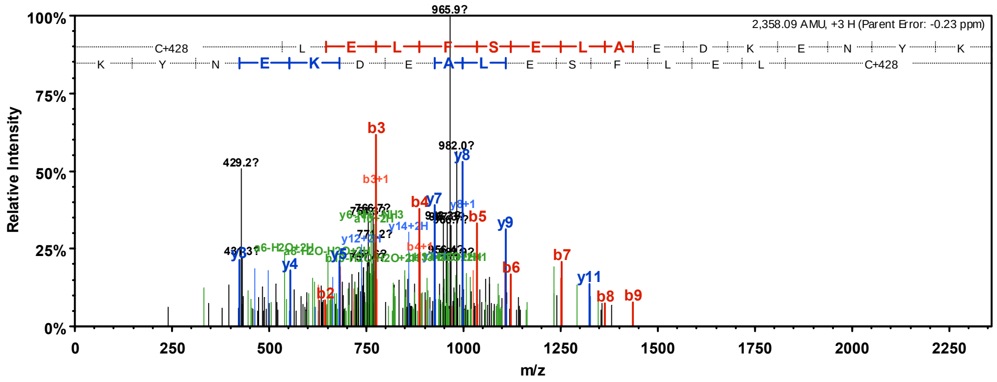

CCT4 (R)SIHDALcVIR(C)
XCorr: 2.98 ΔCn: 0.4

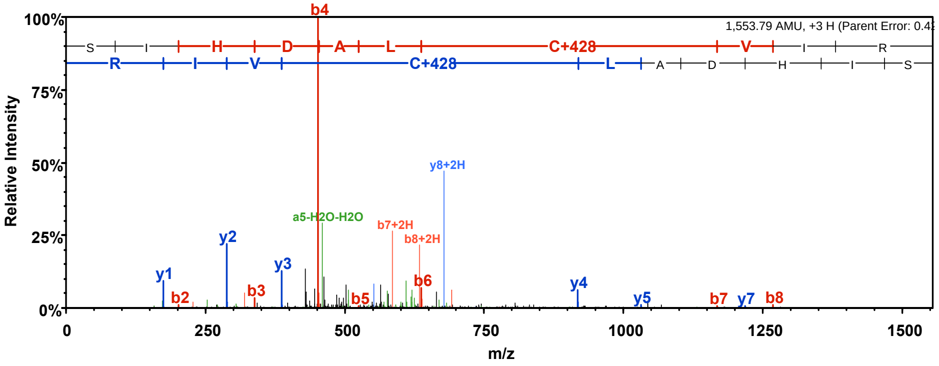

T-complex protein 1 subunit alpha (R)IcDDELILIK(N)
XCorr: 2.31 ΔCn: 0.12

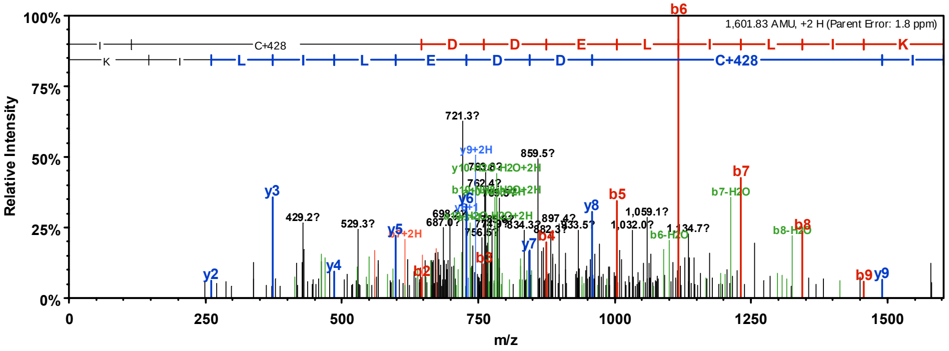

T-complex protein 1 subunit delta (K)TGcNVLLIQK(S)
XCorr: 2.46 ΔCn: 0.11

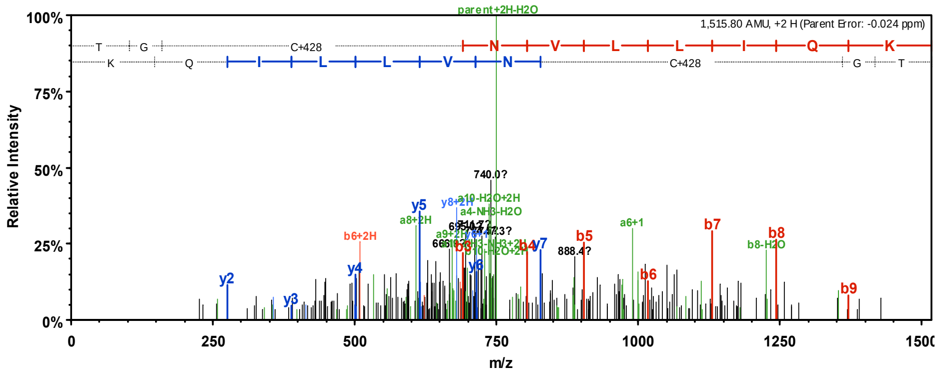

Chaperonin containing TCP1, subunit 3 isoform b (K)IPGGIIEDScVLR(G)
XCorr: 3.25 ΔCn: 0.48

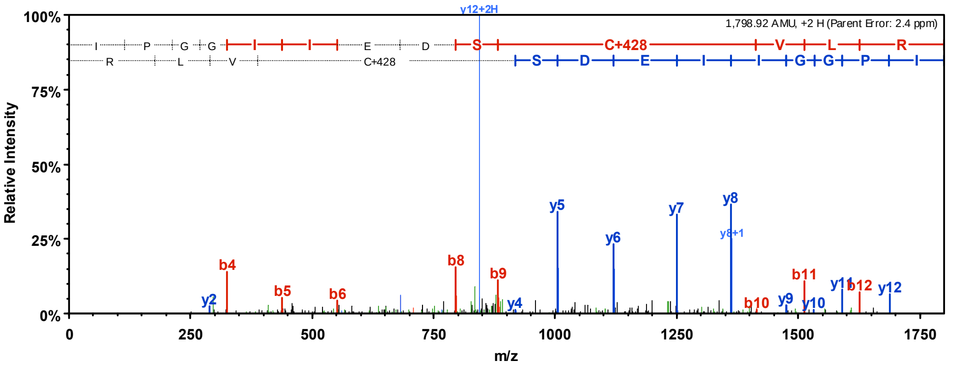

TCP1(K)VLcELADLQDK(E)
XCorr: 3.2281 ΔCn: 0.3898

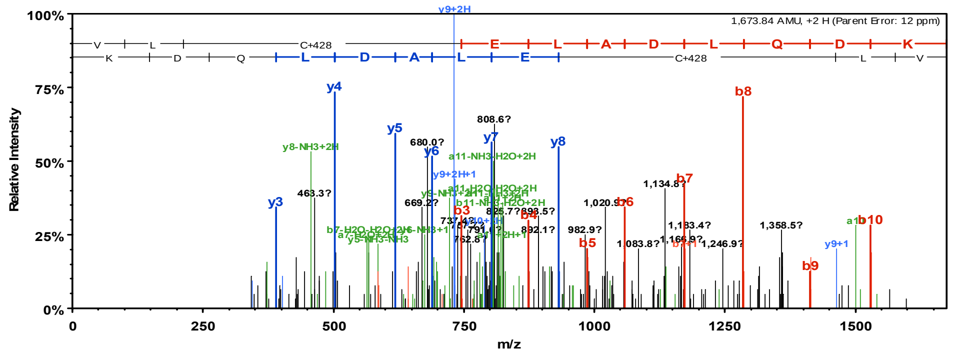

Chaperonin containing TCP1, subunit 3 isoform b
(R)WSSLAcNIALDAVK(M)
XCorr: 2.15 ΔCn: 0.38

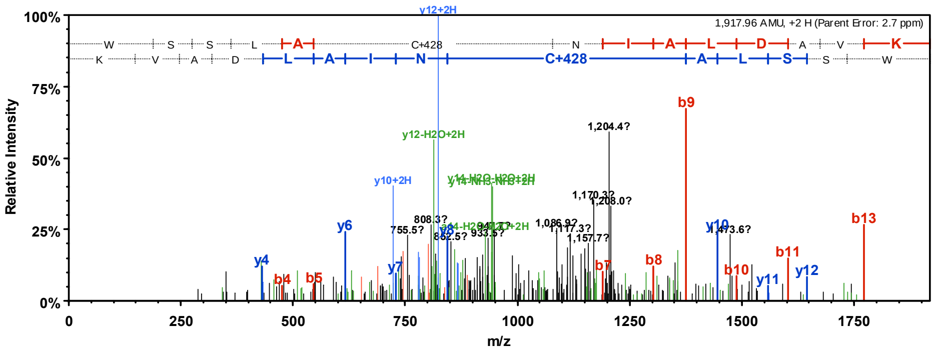

T-complex protein 1 subunit eta (R)YNFFTGcPK(A)
XCorr: 2.20 ΔCn: 0.22

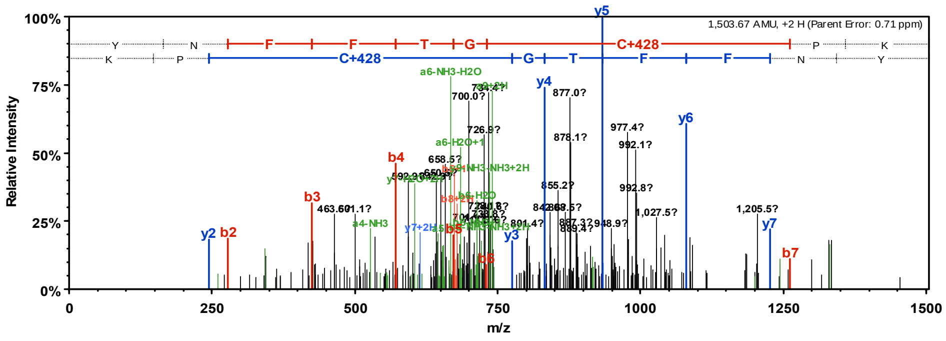

Protein phosphatase 1G (K)cSGDGVGAPR(L)
XCorr: 2.37 ΔCn: 0.27

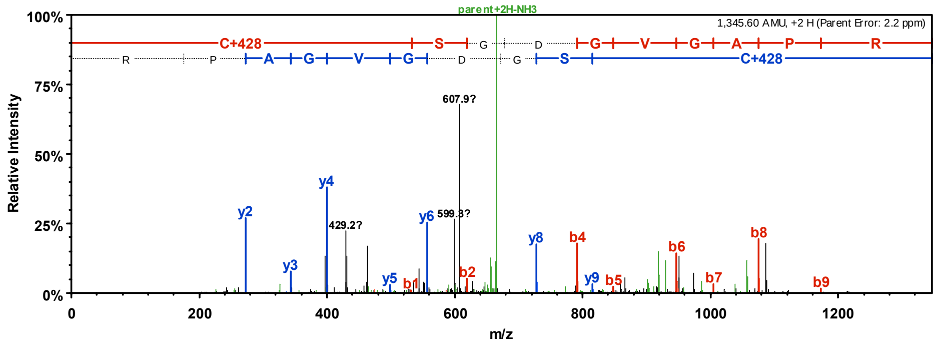

T-complex protein 1 subunit eta (R)QLcDNAGFDATNILNK(L)
XCorr: 3.95 ΔCn: 0.32

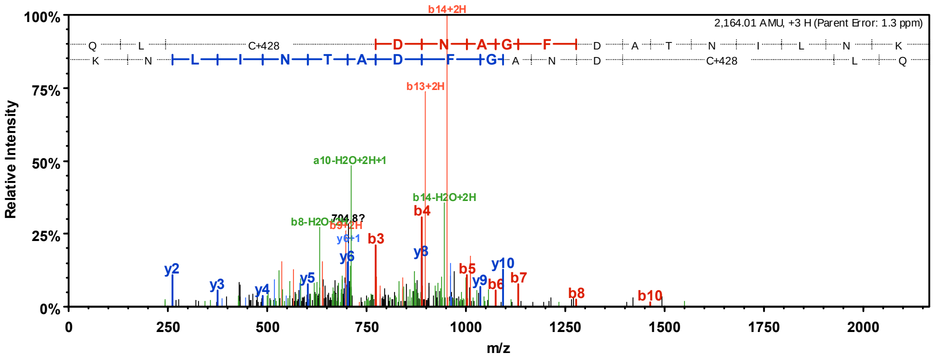

Isoform 1 of Epidermal growth factor receptor (K)NLcYANTINWK(K)
XCorr: 3.47 ΔCn: 0.41

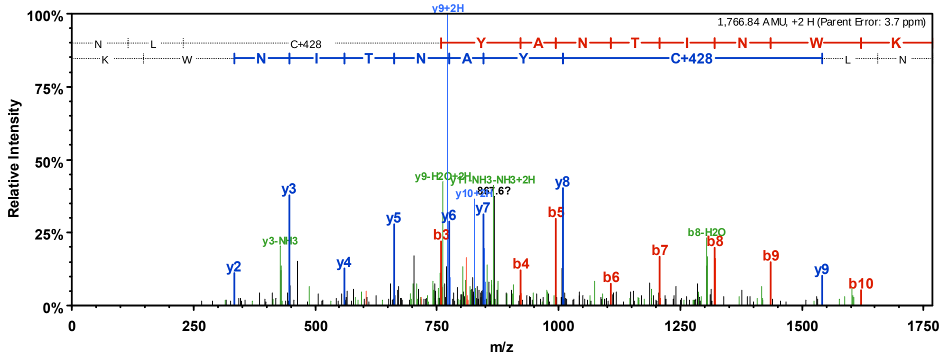

Leucine-rich PPR motif-containing protein, mitochondrial (R)cVANNQVETLEK(L)
XCorr: 4.03 ΔCn: 0.52

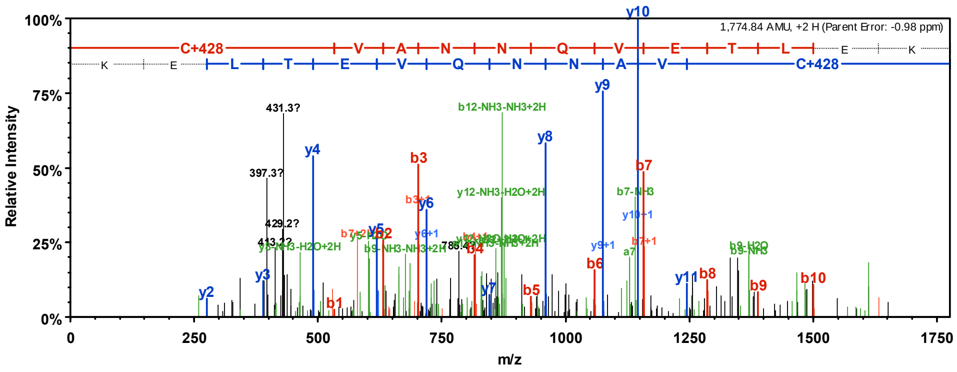

Isoform 1 of Epidermal growth factor receptor (K)YSFGATcVK(K)
XCorr: 2.39 ΔCn: 0.46

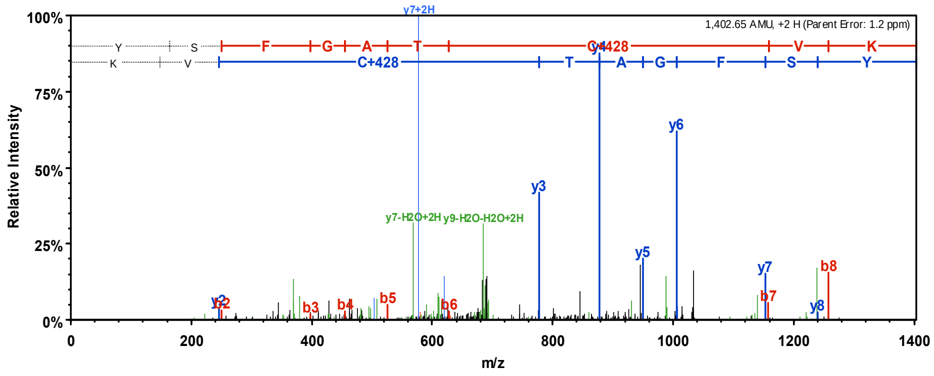

Isoform 3 of Ribosome-binding protein 1 (R)ELcHTQSSHASLR(A)
XCorr: 2.59 ΔCn: 0.44

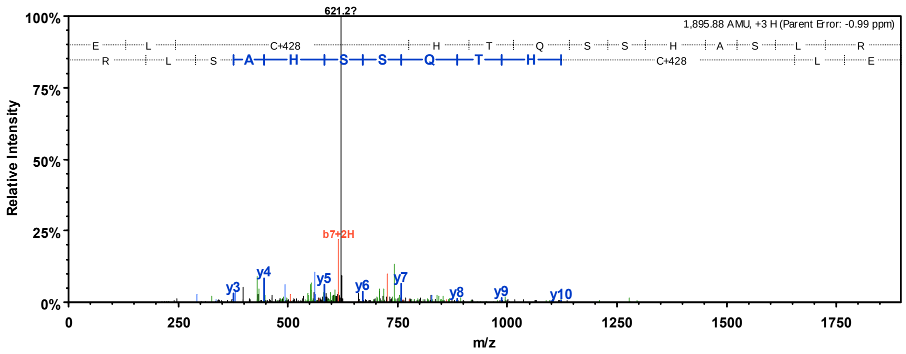

Heterogeneous nuclear ribonucleoprotein L (K)LcFSTAQHAS(-)
XCorr: 2.25 ΔCn: 0.49

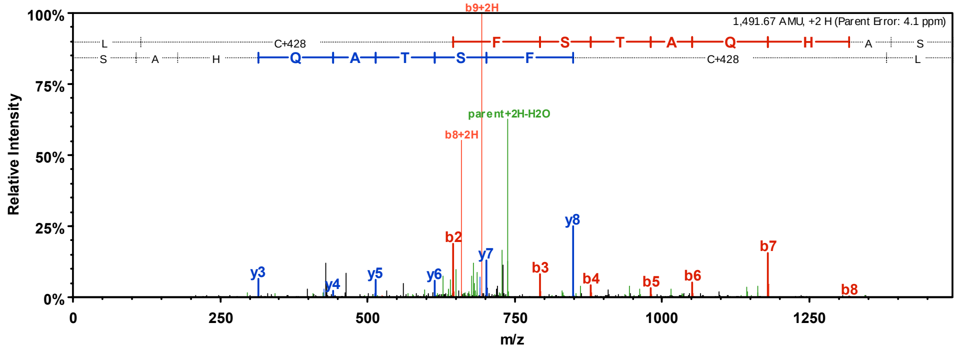

Isoform 3 of Ribosome-binding protein 1 (K)LTAEFEEAQTSAcR(L)
XCorr: 3.07 ΔCn: 0.41

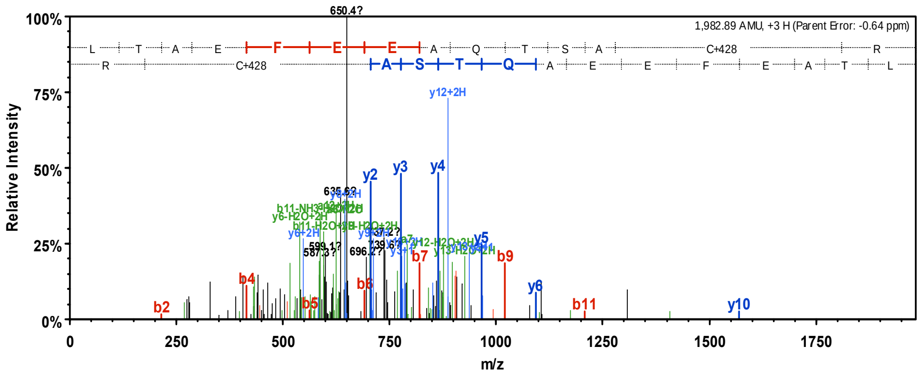

14-3-3 protein theta (R)YLAEVAcGDDR(K)
XCorr: 2.76 ΔCn: 0.51

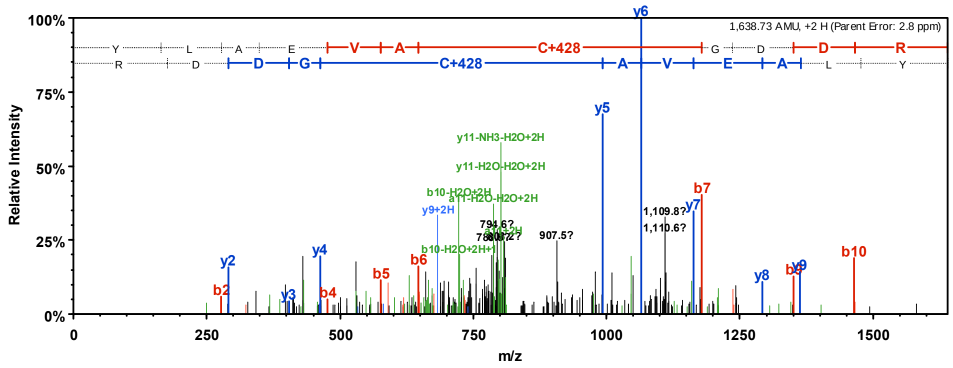

Heterogeneous nuclear ribonucleoprotein H (R)DLNYcFSGMSDHR(Y)
XCorr: 32.63 ΔCn: 0.51

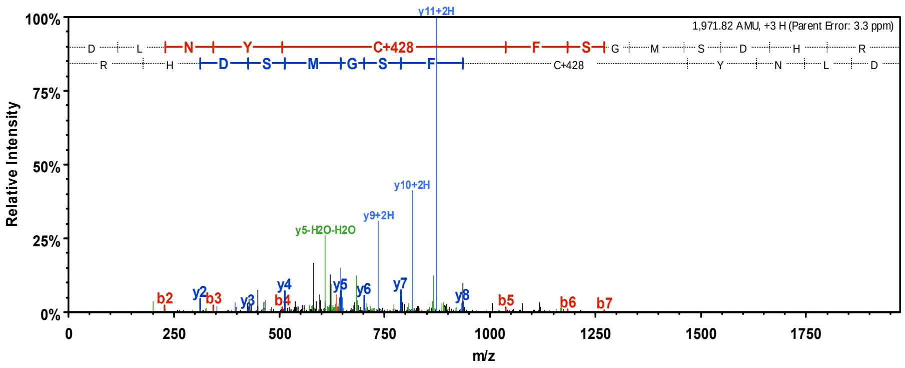

Tubulin alpha-4A chain (R)SIQFVDWcPTGFK(V)
XCorr: 4.07 ΔCn: 0.54

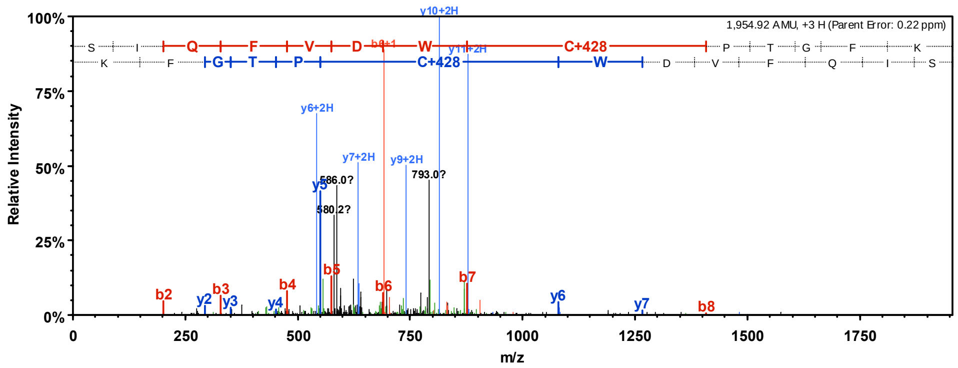

Tubulin alpha-4A chain (R)AVcmLSNTTAIAEAWAR(L)
XCorr: 3.15 ΔCn: 0.38

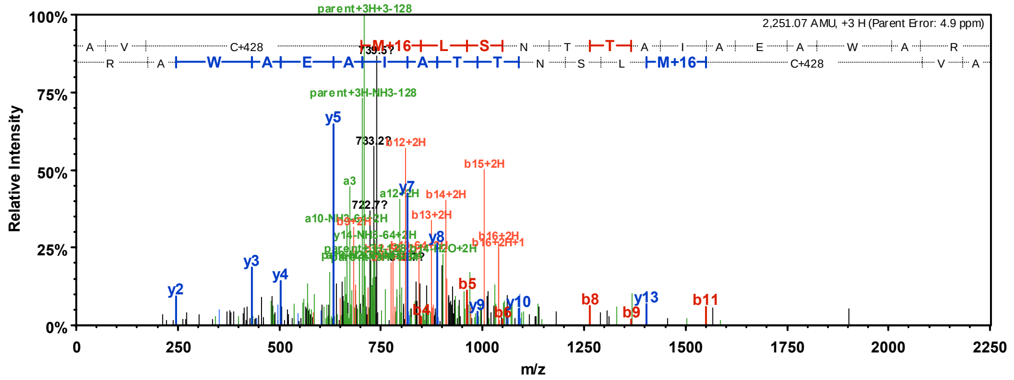

Tubulin alpha-4A chain (K)AYHEQLSVAEITNAcFEPANQmVK(C)
XCorr: 3.66 ΔCn: 0.48

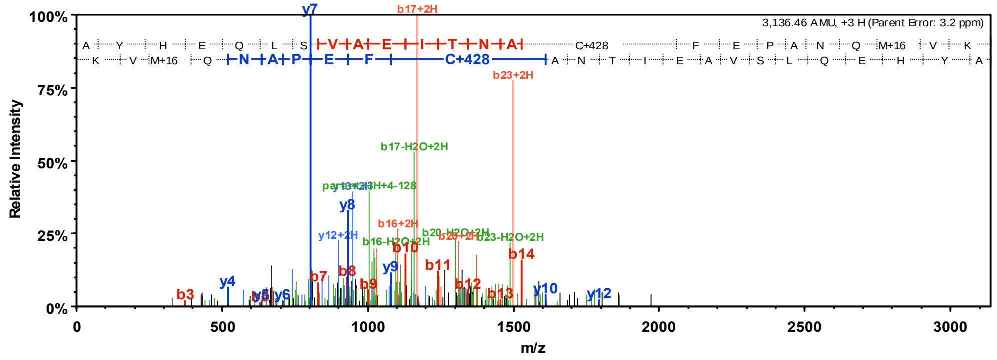

Isoform 1 of Epidermal growth factor receptor (R)AcGADSYEmEEDGVR(K)
XCorr: 4.44 ΔCn: 0.72

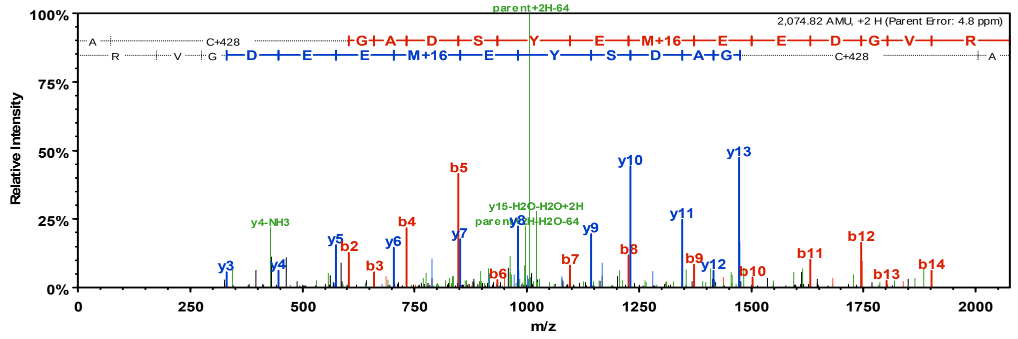

Supplement: Figure S1 — MS/MS spectra for all the biotinylated peptides identified. The MS/MS spectra of biotinylated peptides were evaluated by Scaffold (Proteome Software). (1.67 MB PDF) [file pone.0009075.s005.pdf]
